# Persistent Boryl Radicals as Highly Reducing Photoredox Catalysts for Debrominative Borylations

**DOI:** 10.1021/jacs.5c03864

**Published:** 2025-05-30

**Authors:** Jingjing Wu, Hui Wang, Huaquan Fang, Kevin C. Wang, Deborin Ghosh, Valerio Fasano, Adam Noble, Varinder K. Aggarwal

**Affiliations:** School of Chemistry, 1980 University of Bristol, Cantock’s Close, Bristol BS8 1TS, U.K.

## Abstract

Organic free radicals are commonly perceived to be highly reactive species with short lifetimes, yet there are many examples that defy this convention by displaying remarkable stability. Although these persistent radicals can be relatively unreactive in their ground states, photoexcitation can generate highly reactive excited states. Despite this, they have found limited application as reagents or catalysts in photochemical reactions. Here we report the identification of persistent boryl-bipyridine radicals that function as highly reducing photoredox catalysts. These radicals, which are generated by simply mixing a bipyridine with a diboron reagent, were found to possess excited state reduction potentials that rival the most powerful photoreductants reported to date. We show that this class of doublet state photoredox catalyst can promote borylations of alkyl bromides and various other transformations.

## Graphical abstract

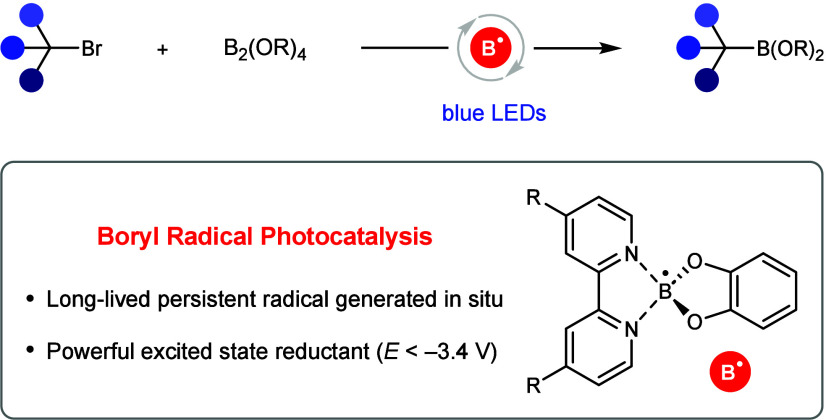

Radical chemistry is dominated by reactions of highly reactive organic free radicals that only exist as transient intermediates.^1^ However, if a radical possesses sufficient steric or electronic stabilization, its reactivity can be attenuated to the extent that it persists for long periods of time.^2^ This phenomenon was first identified by Gomberg in 1900 with the synthesis of the triphenylmethyl radical,^3^ and many other persistent and stable (inert to oxygen) radicals have since been reported (Figure 1a).^2,4^ The long lifetimes of these radicals have led to significant interest in exploiting their unique properties by incorporating them into functional materials, including magnetics, optoelectronics, and photothermal therapeutics.^5^ Conversely, their application as reagents or catalysts in organic synthesis is comparatively rare, with the notable exception of aminoxyl radicals (e.g., TEMPO) which are commonly used as catalysts in oxidation reactions.^6^ The lack of reports detailing their synthetic utility likely results from their low ground state reactivity; however, many persistent radicals undergo photoexcitation upon irradiation with visible-light to generate doublet excited states with enhanced reactivity toward single-electron transfer (SET) with electron donors and acceptors.^7^ Despite this, investigations into photoinduced electron transfer (PET) reactions of persistent radicals have been limited to mechanistic studies rather than their potential applications as photoredox reagents or catalysts in preparative scale synthesis.^7,8^

**Figure 1:**
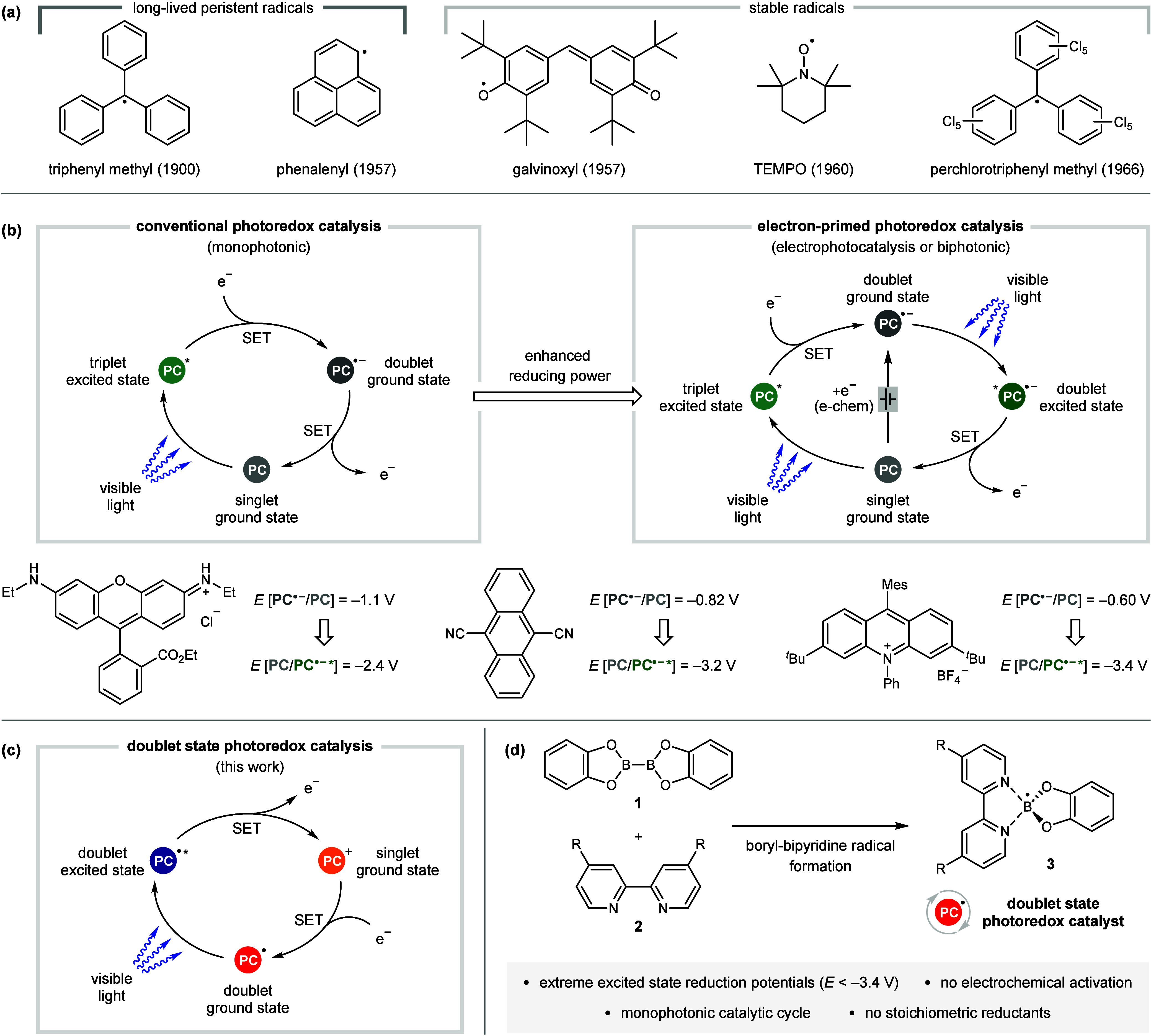
Persistent radicals and their application in photocatalysis.

Over the past decade, photoredox catalysis has become the predominant methodology used for the generation of transient free radicals in organic synthesis.^9^ In their excited state, photoredox catalysts undergo SET with a diverse range of substrates, and the success of these reactions is largely dictated by the catalysts having sufficiently high redox potentials for thermodynamically favored SET.^9a^ Therefore, for substrates with prohibitively high redox potentials, conventional photoredox catalysis fails. A recent strategy that has emerged to overcome this limitation is to perform single-electron oxidation or reduction of an organic photoredox catalyst prior to photoexcitation, termed electron-primed photoredox catalysis (Figure 1b).^10^ This initial SET converts the closed-shell, singlet state catalyst into an open-shell, doublet state (persistent radical) that possesses enhanced excited state redox potentials, thus allowing SET with typically unreactive substrates. However, the crucial SET to form the doublet state necessitates the use of either electrochemistry (electrophotocatalysis)^10a,11^ or biphotonic processes involving an additional PET with stoichiometric oxidants or reductants.^10b,12^ Conversely, to our knowledge, there are no reports detailing the use of a persistent organic radical as the active photoredox catalyst, specifically, one that does not require continual formation via electrochemical or photochemical activation of a singlet precatalyst (Figure 1c).^13^ Such doublet state photoredox catalysts could provide the high redox potentials of electron-primed photoredox catalysis but with simplified experimental setup and increased reaction efficiency by avoiding the need for electrochemistry and requiring only a single photoexcitation. Herein, we report the discovery that simply mixing bis­(catecholato)­diboron (B_2_cat_2_, **1**) with bipyridines **2** generates persistent boryl-bipyridine radicals **3** that display high stability in their ground state but function as exceptionally powerful photoreductants upon irradiation with blue-light (Figure 1d). Furthermore, we demonstrate that the reversibility of the redox cycling between the boryl radicals and their oxidized boronium cation counterparts enables them to function as effective doublet state photoredox catalysts.

Our group,^14^ and others,^15^ recently reported a range of visible-light-mediated, transition metal-free borylations where common functional groups, including carboxylic acids, amines, and alcohols, are transformed into synthetically versatile boronic esters.^16^ These reactions rely on initial activation of the substrates by conversion into redox-active derivatives, which enables formation of photoactive electron donor–acceptor complexes with B_2_cat_2_ and subsequent PET-mediated alkyl radical generation.^17^ This necessity for preinstallation of a redox-active group prevents extension of these methods to substrates lacking a suitable handle for activation. One such class of substrates is the alkyl halides, which are widely exploited in substitution reactions but, apart from the highly reactive alkyl iodides,^18^ engaging them in transition metal-free borylations remains challenging.^19^ This is because of the strong C–halogen bonds and large negative reduction potentials of alkyl bromides and chlorides,^20^ which hinder dehalogenative alkyl radical formation by photolysis or SET. Nonetheless, notable advances have been made in recent years using electrochemical activation or silane-mediated halogen-atom-transfer.^21,22^ In addition, Jiao and co-workers reported pyridine-catalyzed photoinduced dehalogenative borylations, although these required strongly basic conditions for diboron activation.^23^ Our initial proposal endeavored to overcome the challenges associated with dehalogenative borylations by using organic super electron donors (SEDs) as photocatalysts,^24^ inspired by Murphy’s dihydrobipyridine-based SED **4**, which is a powerful photoreductant under UV-light irradiation (Figure 2a).^25^ Although **4** could not be used catalytically, we were encouraged by a report by Shi proposing the related SED **5** could catalyze thermally promoted borylations of *N*-alkyl-pyridinium salts, where **5** was generated in situ by reaction of B_2_cat_2_ with 4,4′-di-*tert*-butyl-2,2′-bipyridine (**2a**) at elevated temperatures.^26^ We reasoned that if **5** could be photoexcited, similarly to **4**, it would provide a valuable photocatalyst for challenging dehalogenative borylations. Gratifyingly, this mechanistic hypothesis led to a successful debrominative borylation protocol, with the reaction of 4-bromopiperidine **6a** with B_2_cat_2_ in the presence of 20 mol % of **2a** under blue-light irradiation giving boronic ester **7a** in 80% yield (Figure 2b). No product was formed in the absence of **2a** or light, which confirms the crucial role of the bipyridine in generating the photoactive species. Finally, using the dimethylamino-bipyridine **2b** led to an improved yield of 92%, likely because the electron-donating amines provide a more strongly reducing catalyst.

**Figure 2:**
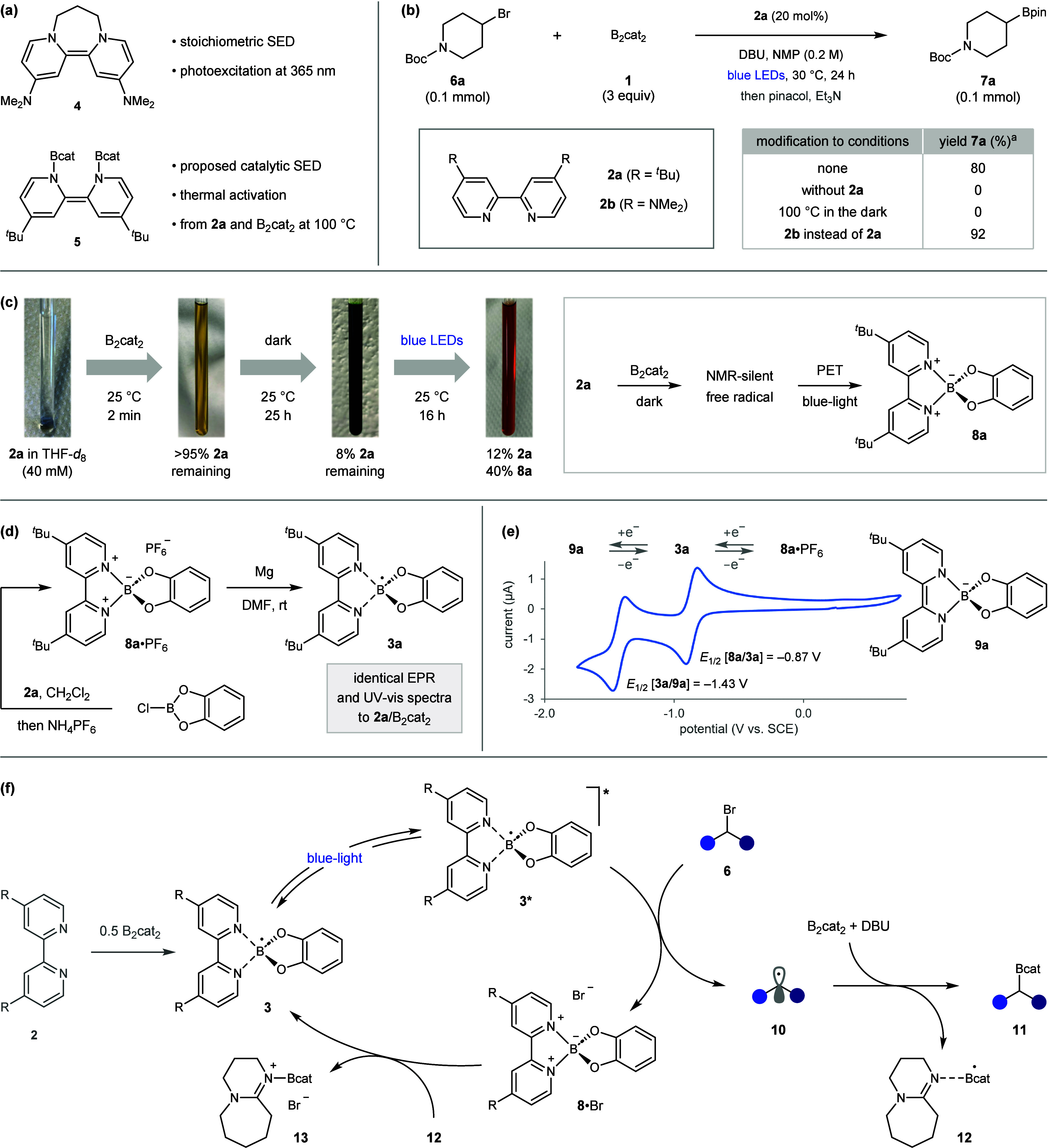
Reaction development and mechanistic investigations. ^*a*^Yields were determined by GC analysis.

Shi’s support for the formation of **5** was provided by ^11^B NMR and DFT, which suggested its generation from **2a** and B_2_cat_2_ was energetically feasible at 100 °C.^26^ However, given that our debrominative borylation proceeds at 30 °C, we questioned their hypothesis and sought to provide additional evidence for the formation of the putative SED **5**. Mixing the two colorless solids, **2a** and B_2_cat_2_, in THF-*d*_8_ resulted in the immediate formation of a yellow solution that became deep green after 25 h (Figure 2c). Surprisingly, ^1^H NMR analysis of this reaction revealed the disappearance of >90% of **2a**, yet no dihydrobipyridine **5** was formed, nor were any other bipyridine-derived species (Figures S6–S10). As the solution was homogeneous throughout the experiment, we suspected that **2a** was transformed into an NMR-silent free radical, which we confirmed using EPR spectroscopy. Subsequent irradiation resulted in the generation of boronium ion **8a**, thus demonstrating the photoredox activity of the free radical and leading us to suspect its structure to be boryl-bipyridine radical **3a**. This was supported through independent synthesis by reduction of boronium **8a**·PF_6_ with magnesium (Figure 2d).^27,28^ These results led us to the conclusion that the photocatalytic species in our reaction was in fact the boryl-bipyridine radical **3**.

To our knowledge, the only report of boryl radical generation by the homolytic cleavage of diboron reagents with 2,2′-bipyridines is from Norman and Russell, who found that B_2_Cl_2_(NMe_2_)_2_ and 2,2′-bipyridine reacted to form a persistent dichloroboryl-bipyridine radical.^28^ Although the stability of related boryl-bipyridine radicals, formed by single-electron reduction of boronium cations, has been described,^29^ there have been no reports detailing their use as reagents or catalysts in redox chemistry.^30^ While Shi postulated that **3a** could be formed from dihydrobipyridine **5** under their reaction conditions, they favored dihydrobipyridine **5** as the active SED.^26^ However, based on the mild conditions under which **3a** is formed, and the lack of experimental support for **5**, it is likely that **3a** was the catalytically active species, and that related boryl-bipyridine radicals are also involved in other borylation reactions.^31^

We subsequently investigated the electrochemical and photophysical properties of radical **3a** to determine its redox properties. Cyclic voltammetry of boronium **8a**·PF_6_ displayed two reversible reduction peaks at −0.87 and −1.43 V versus SCE, highlighting the stability of boryl radical **3a** and its redox cycling between **8a** and dihydrobipyridyl-borate anion **9a** (Figure 2e).^27^ The ground state reduction potential of −0.87 V for **8a** indicates that **3a** is a moderately strong reductant in its ground state, supporting the involvement of related boryl-bipyridine radicals in thermally promoted borylation reactions.^26,31^ The excited state reduction potential of **3a** was then calculated after obtaining an estimate of its excited state energy (*E*_0,0_) of 2.59 eV from the intersection of the normalized absorption and fluorescence spectra.^9a^ This led to an *E*_1/2_[**8a**/3a*] of −3.46 V vs SCE, which makes **3a** a more powerful reductant than elemental lithium (*E* = −3.29 V vs SCE)^12d^ and one of the most strongly reducing photocatalysts reported to date.^32^ Additionally, transient absorption spectroscopy revealed **3a** has an excited state lifetime of 1.2 ns, which is sufficiently long for alkyl bromide activation.^10b^

Based on these studies, we propose the debrominative borylation mechanism shown in Figure 2f. Initial reaction of bipyridine **2** with B_2_cat_2_ generates boryl radical catalyst **3**. Photoexcitation forms the highly reducing doublet excited state 3*, which undergoes SET with alkyl bromide **6** to give alkyl radical **10** and boronium **8**·Br. Borylation of **10** with B_2_cat_2_, facilitated by DBU,^18a^ gives boronic ester **11** and DBU-stabilized boryl radical **12**. Finally, reduction of **8** by **12** is expected to be thermodynamically favored based on the strong reducing ability of Lewis base-stabilized boryl radicals (*E* vs SCE in MeCN is −0.84 V for **8a**, and −1.53 V for DMAc-Bcat radical),^15b^ which regenerates catalyst **3** and forms DBU·BrBcat **13**.

We subsequently investigated the application of the debrominative borylation to a broad range of alkyl bromides (Scheme 1). Cyclic and acyclic secondary substrates were borylated in good to excellent yields (**7a**–**7g**), and primary alkyl bromides also reacted efficiently to afford **7h**–**7s**. Good functional group tolerance was observed, including aromatic rings (**7i–7j**), olefins (**7k**), protected alcohols (**7l**–**7o**), esters (**7p**), nitriles (**7q**) and carbazoles (**7r**). Notably, boronic ester **7o** containing an acetyl-protected primary alcohol was formed in excellent yield, demonstrating the compatibility of base-sensitive functionality. Cyclopropylmethyl bromide underwent ring-opening (**7s**), thus confirming the formation of alkyl radical intermediates. It should be noted that, while the borylations proceeded in higher yields using bipyridine **2b**, synthetically useful yields could also be obtained with commercially available **2a**. Conformationally restricted tertiary alkyl bromides were borylated in high efficiency (**7t**–**7u**), and a 1,1-dibromocyclopropane underwent double debrominative borylation to provide 1,1-bis-boronic ester **7v**. Gratifyingly, our weakly basic conditions were also successful with acyclic tertiary alkyl bromides (**7w**), contrasting the previous reports by Jiao and Studer, where base-mediated elimination prevented borylation of tertiary alkyl halides.^18a,23a^ Finally, we borylated complex natural product-derived alkyl bromides, bearing ketones, acetals, lactones, alcohols, and enones, which provided boronic ester derivatives of (−)-ambroxide (**7x**), epiandrosterone (**7y**), diosgenin (**7z**–**7ab**), tigogenin (**7ac**–**7ad**), and hecogenin (**7ae**) in good yields and high diastereoselectivities. Interestingly, **7ae** was formed with good selectivity for borylation of the secondary over the primary alkyl bromide, however, bis-borylation and enone reduction products were also isolated.

**Scheme 1:**
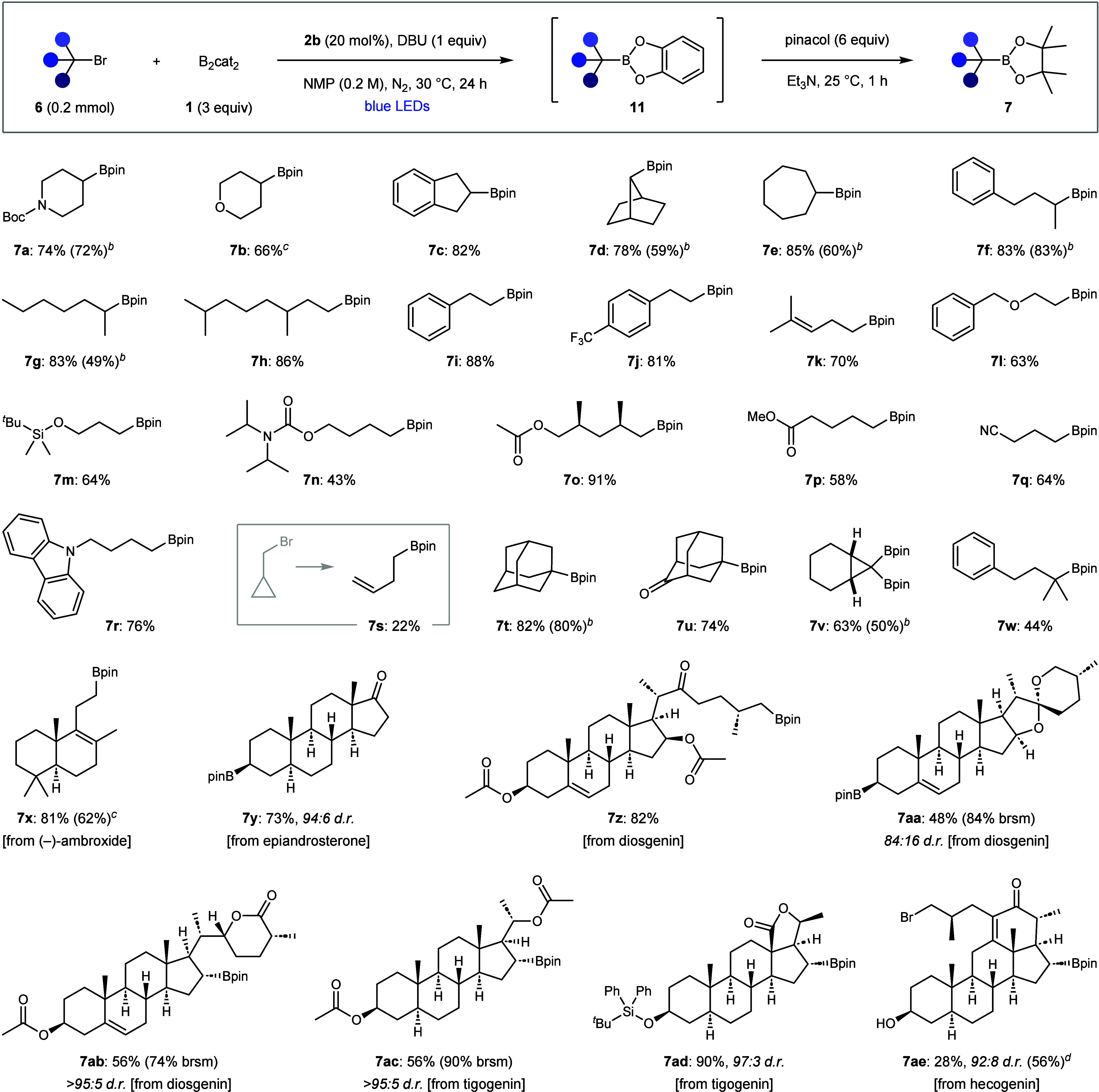
Debrominative Borylation Scope^a^

a Yields are of isolated products (brsm = based on recovered starting material).
b Using **2a** and K_2_CO_3_ as base.
c Using **2a**.
d Total yield, including bis-boronic ester and alkene reduction products.

To further demonstrate the potential of boryl-bipyridine radicals as strongly reducing photoredox catalysts, we investigated their application to other borylation reactions. Catalyst **3b** effectively promoted debrominative aryl radical formation of 2-bromophenyl-thionocarbonate **14**, leading to deoxygenative borylation (Scheme 2a).^14c^ Pleasingly, borylation of alkyl chloride **15** was also possible,^20c,23b^ albeit with low conversion to **7a** (Scheme 2a). We subsequently demonstrated that boryl radicals could catalyze borylations of aryl chlorides **16** (Scheme 2b).^11c,12f,12g,15e–15g,19d,24^ Here, tetramethylphenanthroline **2c** provided improved yields compared to **2b**, thus demonstrating that boryl radical formation was not limited to bipyridines. In addition, bis­(pinacolato)­diboron (B_2_pin_2_) could be used as the borylating agent because of the higher reactivity of aryl radicals,^19^ which meant that only substoichiometric quantities of B_2_cat_2_ were required for the generation of catalyst **3c**. Although conversions in the dechlorinative borylations were modest, the success of these reactions demonstrates that boryl radicals **3** can catalyze highly challenging single-electron reductions (*E* = −2.9 V for **16a**).^12c^ Finally, our optimized conditions effectively promoted the desulfonylation of *N*-tosylindole **18** to form **19** (Scheme 2c), thus highlighting the potential for extending boryl-bipyridine radical photoredox catalysis beyond dehalogenations.

**Scheme 2:**
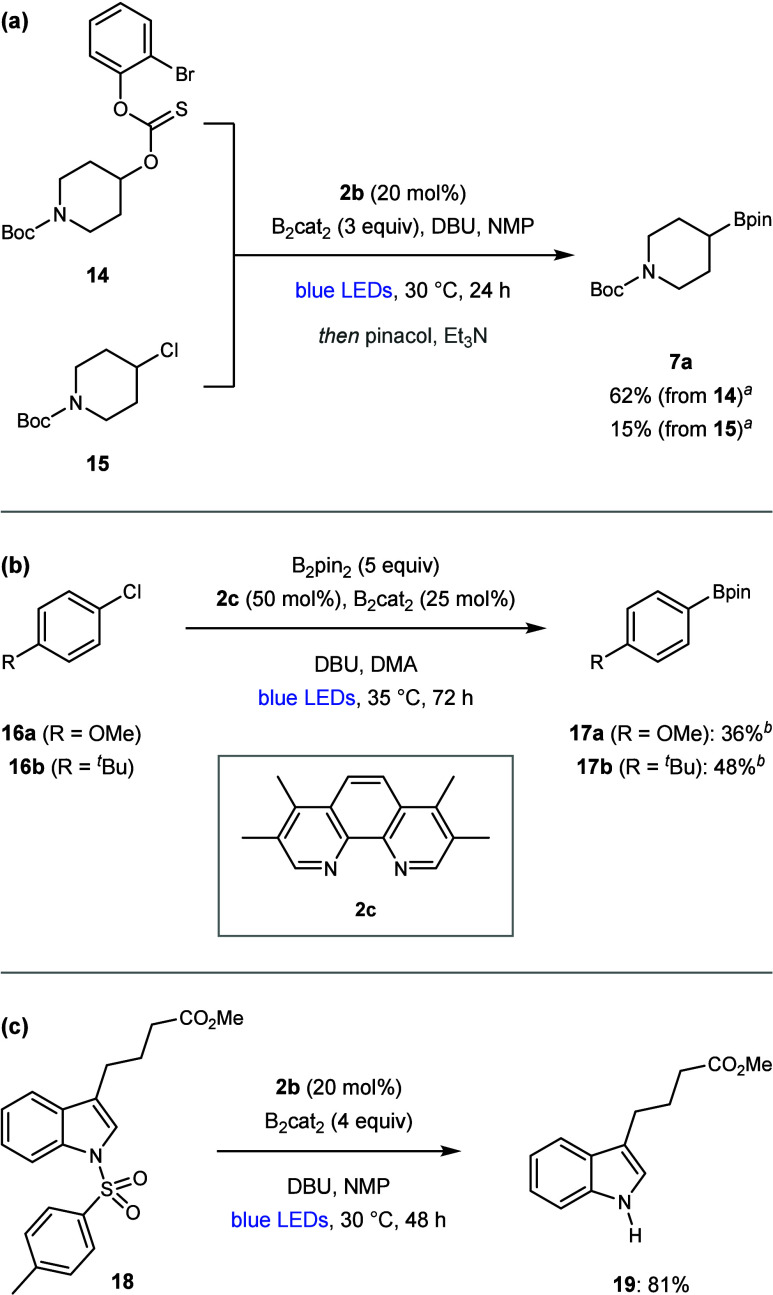
Other Applications

a Yields determined by GC analysis.
b Yield determined by ^1^H NMR analysis.

In conclusion, we have discovered and characterized persistent boryl-bipyridine radicals and demonstrated their ability to act as photoredox catalysts. The radicals are generated by simply mixing 2,2′-bipyridines with B_2_cat_2_ yet were found to be exceptionally powerful excited state reductants. We demonstrated that these doublet state photoredox catalysts allowed the development of mild and operationally simple debrominative borylations of alkyl bromides. Finally, the potential synthetic utility of the boryl-bipyridine radicals as highly reducing photocatalysts was highlighted through their application to deoxygenation, dechlorination, and *N*-desulfonylation reactions.

## Supplementary Material



## Notes

Experimental procedures, characterization data, X-ray crystallography data, cyclic voltammetry data, EPR spectra, NMR spectra for all novel compounds. (PDF)

## †

Frontiers Science Center for Transformative Molecules, Shanghai Key Laboratory for Molecular Engineering of Chiral Drugs, School of Chemistry and Chemical Engineering, Zhangjiang Institute for Advanced Study, Shanghai Jiao Tong University, Shanghai, 200240, China

## §

Key Laboratory of Functional Molecular Solids (Ministry of Education), Anhui Key Laboratory of Molecular Based Materials, College of Chemistry and Materials Science, Anhui Normal University, Wuhu, 241002, China.

## #

Department of Chemistry, University of Milan, Via Golgi 19, 20133 Milano, Italy.
