# Supplementary material for: Persistent Boryl Radicals as Highly Reducing Photoredox Catalysts for Debrominative Borylations
Source: J Am Chem Soc. 2025 May 30;147(23):19450–7. doi: 10.1021/jacs.5c03864 (PMC12164345; doi:10.1021/jacs.5c03864)
Supplement: Supplementary file 1 [file ja5c03864_si_001.pdf]

Supporting Information

Persistent Boryl Radicals as Highly Reducing Photoredox Catalysts for Debrominative Borylations

Jingjing Wu,[†] Hui Wang,[†] Huaquan Fang, Kevin C. Wang, Deborin Ghosh, Valerio Fasano, Adam Noble,* and Varinder K. Aggarwal*

School of Chemistry, University of Bristol, Cantock's Close, Bristol BS8 1TS, United Kingdom

*e-mail: a.noble@bristol.ac.uk, v.aggarwal@bristol.ac.uk

TABLE OF CONTENTS

LIST OF SUPPLEMENTARY SCHEMES, FIGURES AND TABLES 2
LIST OF CHARACTERIZED PRODUCTS 2
1. MATERIALS AND GENERAL METHODS 5
1.1. Glassware, Solvents and Reagents 5
1.2. Chromatography and Instrumentation 5
1.3. Naming of Compounds 5
1.4. Photochemical Equipment 6
2. EXPERIMENTAL DATA 7
2.1. General Procedures 7
2.1.1. General Procedure 1 (GP1): Preparation of Alkyl Bromides from Alcohols 7
2.1.2. General Procedure 2 (GP2): Borylation of Alkyl Bromides 7
2.2. Synthesis of the Bipyridine Catalyst 8
2.3. Alkyl Bromide Synthesis 9
2.4. Optimisation Studies 17
2.5. Borylation Products 18
2.6. Other Applications 46
2.6.1. Deoxygenative Borylation via Aryl Bromide Reduction 46
2.6.2. Borylation of Aryl Bromides 47
2.6.3. Borylation of Alkyl Chlorides 48
2.6.4. Borylation of Aryl Chlorides 49
2.6.5. Desulfonylation of Sulfonamides 51
3. MECHANISTIC STUDIES 53
3.1. NMR Studies 53
3.2. Synthesis of Boronium Ions 59
3.3. Boryl Radical Formation by Reduction of Boronium Ions 61
3.4. Electron Paramagnetic Resonance Spectroscopy 62
3.5. UV-Vis Spectroscopy 68
3.6. Cyclic Voltammetry 69
3.7. Fluorescence Spectroscopy 72
3.8. Approximation of the Excited State Reduction Potential of the Boryl Radical 73
3.9. NMR study of the reaction of boronium **8a** with DBU and K_2CO_3 74
3.10. Quantum Yield Measurement 76
3.11. Time Correlated Single Photon Counting 79
3.12. Transient Absorption Spectroscopy 80
4. X-RAY CRYSTALLOGRAPHY 82
5. SPECTROSCOPIC DATA 85
6. REFERENCES 176

LIST OF SUPPLEMENTARY SCHEMES, FIGURES AND TABLES

Figure S1. Photochemical reaction setup ... 6
Table S1. Optimisation Studies ... 17
Figure S2. Determination of the d.r. of **7y** ... 34
Figure S3. Determination of the d.r. of **7ad** ... 41
Figure S4. Determination of the d.r. of **7ae** ... 44
Scheme S1. Deoxygenative borylations ... 46
Table S2. Different reaction conditions for the borylation of aryl chloride **16a** ... 49
Figure S5. Change in colour of 1:1 dtbbpy/B_2cat_2 solutions in THF-d_8 over time and after irradiation ... 53
Figure S6. ^{1}H NMR spectra of 1:1 dtbbpy/B_2cat_2 in THF-d_8 ... 54
Figure S7. ^{1}H NMR spectra of 1:1 dtbbpy/B_2cat_2 in THF-d_8 after irradiation (6.1– 9.4ppm) ... 55
Figure S8. ^{1}H NMR spectra of 1:1 dtbbpy/B_2cat_2 in THF-d_8 after irradiation (0–10 ppm) ... 56
Figure S9. ^{11}B NMR spectra of 1:1 dtbbpy/B_2cat_2 in THF-d_8 ... 57
Figure S10. ^{11}B NMR spectra of 1:1 dtbbpy/B_2cat_2 in THF-d_8 after irradiation ... 58
Figure S11. EPR spectra of dtbbpy/B_2cat_2 and **8a**•PF_6/Mg in DMF ... 62
Figure S12. EPR spectra of 1:1 dtbbpy/B_2cat_2 in DMF, NMP, and THF ... 63
Table S3. DFT calculations data ... 64
Figure S13. Singly Occupied Molecular Orbital (Isovalue = 0.04) ... 66
Figure S14. UV-Vis absorption spectra in DMF ... 68
Figure S15. Cyclic voltammogram of (dtbbpyBcat)(PF_6) in DMF ... 69
Figure S16. Cyclic voltammogram of (dtbbpyBcat)(PF_6) in MeCN ... 70
Figure S17. Cyclic voltammogram of (bpyBcat)(PF_6) in MeCN ... 71
Figure S18. Normalised absorbance, excitation and emission spectra of dtbbpy/B_2cat_2 in DMF ... 72
Scheme S2. Proposed pathways for regenerating the boryl radical catalyst ... 74
Figure S19. ^{1}H NMR of (dtbbpyBcat)PF_6 and K_2CO_3 in DMF-d_7 ... 75
Figure S20. ^{1}H NMR study of (dtbbpyBcat)PF_6 and DBU in DMF-d_7 ... 75
Figure S21. Actinometry: UV/Vis spectra of ferrioxalate/1,10-phenanthroline solutions ... 76
Figure S22. Actinometry: Moles of Fe^{2+} formed vs. irradiation time ... 77
Figure S23. Fluorescence lifetime measurement using TCSPC ... 79
Figure S24. Transient absorption spectroscopy ... 80
Figure S25. Kinetic analysis of the stimulated emission decay ... 81
Figure S26. Kinetic analysis of the excited state absorption decay ... 81
Figure S27. Kinetic analysis of the ground state bleach decay ... 81
Figure S28. Crystal structure of **8a**•PF_6 ... 82
Figure S29. Crystal structure of **8d**•PF_6 ... 83
Table S4. Crystal data and structure refinement for 8a•PF_6 and 8d•PF_6 ... 84

LIST OF CHARACTERIZED PRODUCTS

N^4,*N*^{4},*N*$^{4'}$,*N*$^{4'}$-Tetramethyl-[2,2'-bipyridine]-4,4'-diamine (**2b**) ... 8
(3-Bromobutyl)benzene (**6f**) ... 9
4-Bromobutyl diisopropylcarbamate (**6n**) ... 9
(2*S*,4*R*)-5-Bromo-2,4-dimethylpentyl acetate (**6o**) ... 10

9-(4-Bromobutyl)-9H-carbazole (**6r**) 10
(3-Bromo-3-methylbutyl)benzene (**6w**) 11
(4a*S*,8a*S*)-8-(2-Bromoethyl)-4,4,7,8a-tetramethyl-1,2,3,4,4a,5,6,8a-octahydronaphthalene (**6x**) 11
Epiandrosterone bromide derivative **6y** 12
(3*S*,8*S*,9*S*,10*R*,13*S*,14*S*,16*S*,17*R*)-17-((2*S*,6*R*)-7-Bromo-6-methyl-3-oxoheptan-2-yl)-10,13-dimethyl-2,3,4,7,8,9,10,11,12,13,14,15,16,17-tetradecahydro-1H-cyclopenta[a]phenanthrene-3,16-diyl diacetate (**6z**) 13
Diosgenin bromide derivative **6aa** 13
(3*S*,8*S*,9*S*,10*R*,13*S*,14*S*,16*R*,17*R*)-16-Bromo-10,13-dimethyl-17-((*S*)-1-((2*R*,5*R*)-5-methyl-6-oxotetrahydro-2H-pyran-2-yl)ethyl)-2,3,4,7,8,9,10,11,12,13,14,15,16,17-tetradecahydro-1H-cyclopenta[a]phenanthren-3-yl acetate (**6ab**) 14
(*S*)-1-((3*S*,5*S*,8*R*,9*S*,10*S*,13*S*,14*S*,16*R*,17*S*)-3-Acetoxy-16-bromo-10,13-dimethylhexadecahydro-1H-cyclopenta[a]phenanthren-17-yl)ethyl acetate (**6ac**) 15
(3*S*,3a*S*,4*R*,5a*S*,5b*R*,7a*S*,9*S*,11a*S*,11b*S*,13a*R*)-4-Bromo-9-((*tert*-butyldiphenylsilyl)oxy)-3,11a-dimethylhexadecahydro-1H,3H-naphtho[2',1':4,5]indeno[1,7a-c]furan-1-one (**6ad**) 15
Hecogenin bromide derivative **6ae** 16
tert-Butyl 4-(4,4,5,5-tetramethyl-1,3,2-dioxaborolan-2-yl)piperidine-1-carboxylate (**7a**) 18
4,4,5,5-Tetramethyl-2-(tetrahydro-2H-pyran-4-yl)-1,3,2-dioxaborolane (**7b**) 18
2-(2,3-Dihydro-1H-inden-2-yl)-4,4,5,5-tetramethyl-1,3,2-dioxaborolane (**7c**) 19
2-((1*R*,4*S*)-Bicyclo[2.2.1]heptan-7-yl)-4,4,5,5-tetramethyl-1,3,2-dioxaborolane (**7d**) 19
2-Cycloheptyl-4,4,5,5-tetramethyl-1,3,2-dioxaborolane (**7e**) 20
4,4,5,5-Tetramethyl-2-(4-phenylbutan-2-yl)-1,3,2-dioxaborolane (**7f**) 21
2-(Heptan-2-yl)-4,4,5,5-tetramethyl-1,3,2-dioxaborolane (**7g**) 21
2-(3,7-Dimethyloctyl)-4,4,5,5-tetramethyl-1,3,2-dioxaborolane (**7h**) 22
4,4,5,5-Tetramethyl-2-phenethyl-1,3,2-dioxaborolane (**7i**) 23
4,4,5,5-Tetramethyl-2-(4-(trifluoromethyl)phenethyl)-1,3,2-dioxaborolane (**7j**) 23
4,4,5,5-Tetramethyl-2-(4-methylpent-3-en-1-yl)-1,3,2-dioxaborolane (**7k**) 24
2-(2-(Benzyloxy)ethyl)-4,4,5,5-tetramethyl-1,3,2-dioxaborolane (**7l**) 25
tert-butyldimethyl(3-(4,4,5,5-tetramethyl-1,3,2-dioxaborolan-2-yl)propoxy)silane (**7m**) 25
4-(4,4,5,5-Tetramethyl-1,3,2-dioxaborolan-2-yl)butyl diisopropylcarbamate (**7n**) 26
(2*S*,4*S*)-2,4-Dimethyl-5-(4,4,5,5-tetramethyl-1,3,2-dioxaborolan-2-yl)pentyl acetate (**7o**) 26
Methyl 5-(4,4,5,5-tetramethyl-1,3,2-dioxaborolan-2-yl)pentanoate (**7p**) 27
4-(4,4,5,5-Tetramethyl-1,3,2-dioxaborolan-2-yl)butanenitrile (**7q**) 28
9-(4-(4,4,5,5-Tetramethyl-1,3,2-dioxaborolan-2-yl)butyl)-9H-carbazole (**7r**) 28
2-(But-3-en-1-yl)-4,4,5,5-tetramethyl-1,3,2-dioxaborolane (**7s**) 29
2-((3r,5r,7r)-Adamantan-1-yl)-4,4,5,5-tetramethyl-1,3,2-dioxaborolane (**7t**) 29
5-(4,4,5,5-Tetramethyl-1,3,2-dioxaborolan-2-yl)adamantan-2-one (**7u**) 30
2,2'-(Bicyclo[4.1.0]heptane-7,7-diyl)bis(4,4,5,5-tetramethyl-1,3,2-dioxaborolane) (**7v**) 31
4,4,5,5-Tetramethyl-2-(2-methyl-4-phenylbutan-2-yl)-1,3,2-dioxaborolane (**7w**) 31
4,4,5,5-Tetramethyl-2-(2-((4a*S*,8a*S*)-2,5,5,8a-tetramethyl-3,4,4a,5,6,7,8,8a-octahydronaphthalen-1-yl)ethyl)-1,3,2-dioxaborolane (**7x**) 32
(3*S*,5*S*,8*R*,9*S*,10*S*,13*S*,14*S*)-10,13-Dimethyl-3-(4,4,5,5-tetramethyl-1,3,2-dioxaborolan-2-yl)hexadecahydro-17*H*-cyclopenta[*a*]phenanthren-17-one (**7y**) 33
(3*S*,8*S*,9*S*,10*R*,13*S*,14*S*,16*S*,17*R*)-10,13-Dimethyl-17-((2*S*,6*S*)-6-methyl-3-oxo-7-(4,4,5,5-tetramethyl-1,3,2-dioxaborolan-2-yl)heptan-2-yl)-2,3,4,7,8,9,10,11,12,13,14,15,16,17-tetradecahydro-1*H*-cyclopenta[*a*]phenanthrene-3,16-diyl diacetate (**7z**) 34
4,4,5,5-Tetramethyl-2-((4*S*,5'*R*,6a*R*,6b*S*,8a*S*,8b*R*,9*S*,10*R*,11a*S*,12a*S*,12b*S*)-5',6a,8a,9-tetramethyl-1,3,3',4,4',5,5',6,6a,6b,6',7,8,8a,8b,9,11a,12,12a,12b-icosahydrospiro[naphtho[2',1':4,5]indeno[2,1-*b*]furan-10,2'-pyran]-4-yl)-1,3,2-dioxaborolane (**7aa**) 35

(3*S*,8*S*,9*S*,10*R*,13*S*,14*S*,16*R*,17*R*)-10,13-Dimethyl-17-((*S*)-1-((2*R*,5*R*)-5-methyl-6-oxotetrahydro-2H-pyran-2-yl)ethyl)-16-(4,4,5,5-tetramethyl-1,3,2-dioxaborolan-2-yl)-2,3,4,7,8,9,10,11,12,13,14,15,16,17-tetradecahydro-1*H*-cyclopenta[*a*]phenanthren-3-yl acetate (**7ab**) ... 36

(3*S*,8*S*,9*S*,10*R*,13*S*,14*S*,16*R*,17*R*)-16-Hydroxy-10,13-dimethyl-17-((*S*)-1-((2*R*,5*R*)-5-methyl-6-oxotetrahydro-2H-pyran-2-yl)ethyl)-2,3,4,7,8,9,10,11,12,13,14,15,16,17-tetradecahydro-1*H*-cyclopenta[*a*]phenanthren-3-yl acetate (**S1ab**) ... 37

(*S*)-1-((3*S*,5*S*,8*R*,9*S*,10*S*,13*S*,14*S*,16*R*,17*S*)-3-Acetoxy-10,13-dimethyl-16-(4,4,5,5-tetramethyl-1,3,2-dioxaborolan-2-yl)hexadecahydro-1*H*-cyclopenta[*a*]phenanthren-17-yl)ethyl acetate (**7ac**) ... 38

(3*S*,5*S*,8*R*,9*S*,10*S*,13*S*,14*S*,16*R*,17*S*)-17-((*S*)-1-Acetoxyethyl)-10,13-dimethylhexadecahydro-1*H*-cyclopenta[*a*]phenanthrene-3,16-diyl diacetate (**S1ac′**) ... 39

(3*S*,3a*S*,4*R*,5a*S*,5b*R*,7a*S*,9*S*,11a*S*,11b*S*,13a*R*)-9-((*tert*-Butyldiphenylsilyl)oxy)-3,11a-dimethyl-4-(4,4,5,5-tetramethyl-1,3,2-dioxaborolan-2-yl)hexadecahydro-1*H*,3*H*-naphtho[2',1':4,5]indeno[1,7a-*c*]furan-1-one (**7ad**) ... 40

(3*S*,3a*R*,4*R*,5a*S*,5b*R*,7a*S*,9*S*,11a*S*,11b*S*,13a*R*)-9-((*tert*-Butyldiphenylsilyl)oxy)-4-hydroxy-3,11a-dimethylhexadecahydro-1*H*,3*H*-naphtho[2',1':4,5]indeno[1,7a-*c*]furan-1-one (**S1ad**) ... 41

(2*R*,2a*R*,2a^1*S*,3*R*,6a*S*,6b*S*,9*S*,10a*S*,12a*R*,12b*S*)-5-((*R*)-3-Bromo-2-methylpropyl)-9-hydroxy-2a^1,3,6b-trimethyl-2-(4,4,5,5-tetramethyl-1,3,2-dioxaborolan-2-yl)-1,2,2a,3,6,6a,6b,7,8,9,10,10a,11,12,12a,12b-hexadecahydrocyclopenta[*ij*]tetraphen-4(2a^1*H*)-one (**7ae**) ... 42

(2*R*,2a*R*,2a^1*S*,3*R*,6a*S*,6b*S*,9*S*,10a*S*,12a*R*,12b*S*)-5-((*R*)-3-Bromo-2-methylpropyl)-2a^1,3,6b-trimethyl-4-oxo-1,2,2a,2a^1,3,4,6,6a,6b,7,8,9,10,10a,11,12,12a,12b-octadecahydrocyclopenta[*ij*]tetraphene-2,9-diyl diacetate (**S1ae′**) ... 43

(2*R*,2a*R*,2a^1*R*,3*R*,5*R*,5a*S*,6a*S*,6b*S*,9*S*,10a*S*,12a*R*,12b*S*)-5-((*R*)-3-Bromo-2-methylpropyl)-9-hydroxy-2a^1,3,6b-trimethyl-2-(4,4,5,5-tetramethyl-1,3,2-dioxaborolan-2-yl)octadecahydrocyclopenta[*ij*]tetraphen-4(2a^1*H*)-one (**7ae-2**) ... 44

(2*R*,2a*R*,2a^1*R*,3R,5*R*,5a*S*,6a*S*,6b*S*,9*S*,10a*S*,12a*R*,12b*S*)-9-Hydroxy-2a^1,3,6b-trimethyl-5-((*S*)-2-methyl-3-(4,4,5,5-tetramethyl-1,3,2-dioxaborolan-2-yl)propyl)-2-(4,4,5,5-tetramethyl-1,3,2-dioxaborolan-2-yl)octadecahydrocyclopenta[*ij*]tetraphen-4(2a^1*H*)-one (**7ae-3**) ... 45

Methyl 4-(1-tosyl-1H-indol-3-yl)butanoate (**18**) ... 51

Methyl 4-(1H-indol-3-yl)butanoate (**19**) ... 52

4,4'-Di-*tert*-butyl-2,2'-bipyridylbenzo[*d*][1,3,2]dioxaboronium hexafluorophosphate (**8a**•PF_6) ... 59

2,2'-Bipyridylbenzo[*d*][1,3,2]dioxaboronium hexafluorophosphate (**8d**•PF_6) ... 60

1. MATERIALS AND GENERAL METHODS

1.1. Glassware, Solvents and Reagents

All manipulations were performed using oven-dried (130 °C for a minimum of 12 h) or flame-dried glassware under an atmosphere of nitrogen, unless otherwise stated.

All anhydrous solvents were commercially supplied from Sigma-Aldrich [*N*-methyl-2-pyrrolidone (NMP), *N*,*N*-dimethylformamide (DMF), *N*,*N*-dimethylacetamide (DMAc)] or dried using an Anhydrous Engineering alumina column drying system [THF, toluene, Et_2O, dichloromethane (DCM), and acetonitrile (MeCN)]. Water is deionised and brine refers to a saturated aqueous solution of NaCl. Reagents were purchased from commercial sources and used as received.

1.2. Chromatography and Instrumentation

Thin layer chromatography (TLC) was performed using aluminium-backed Merck Kieselgel 60 F254 fluorescent treated silica gel plates, which were visualized under UV light and by staining with aqueous basic potassium permanganate followed by heating, an ethanolic solution of phosphomolybdic acid (PMA) followed by heating, Hanessian's stain (CAM stain) followed by heating, or an ethanolic solution of ninhydrin followed by heating.

Flash column chromatography (FCC) was carried out using Sigma-Aldrich silica gel (60 Å, 230-400 mesh, 40-63 µm) or a Biotage Isolera^{TM} flash purification system. In cases where automated column chromatography was employed the solvent gradient and flow rate are indicated.

NMR spectra were recorded at various field strengths, as indicated, using Bruker 400 MHz, Varian VNMR 400 MHz, Varian VNMR 500 MHz, or Bruker Cryo 500 MHz for ^{1}H, ^{11}B, ^{13}C and ^{19}F acquisitions. All NMR spectra were recorder at 25 °C unless otherwise stated. Chemical shifts (δ) are reported in parts per million (ppm) and referenced $CDCl_3$ (^{1}H: 7.26 ppm; ^{13}C: 77.16 ppm). Coupling constants (*J*) are given in Hertz (Hz) and refer to apparent multiplicities (s = singlet, d = doublet, t = triplet, q = quartet, quin = quintet, sex = sextet, h = heptet, m = multiplet, br s = broad signal, dd = doublet of doublets, etc.). The ^{1}H NMR spectra are reported as follows: chemical shift (multiplicity, coupling constants, number of protons)

High resolution mass spectra (HRMS) were recorded on a Bruker Daltonics MicrOTOF II by Electrospray Ionisation (ESI), a Thermo Scientific Orbitrap by Atmospheric Pressure Chemical Ionisation (ESI, APCI), or a Thermo Scientific QExactive (EI).

Infrared (IR) spectra were recorded as a thin film on a Perkin Elmer Spectrum One FT-IR equipped with an ATR sampling accessory. Selected absorption maxima (ν_{max}) are reported in wavenumbers (cm^{-1}).

Gas chromatography (GC) was performed on an Agilent Technologies 6890N Network GC System using an Agilent HP-5 column (15 m × 0.25 mm × 0.25 µm).

1.3. Naming of Compounds

Compound names are those generated by ChemDraw Professional 20.0 software (PerkinElmer), following the IUPAC nomenclature.

1.4. Photochemical Equipment

The blue LEDs were either the Penn OC Photoreactor M1 (purchased from www.pennoc.com, discontinued) or the Penn PhD Photoreactor M2 (purchased from www.sigmaaldrich.com) equipped with 450 nm LEDs.

Reaction mixtures were prepared under a nitrogen atmosphere in 7 mL vials before sealing with parafilm and placing in the photoreactor (Figure S1). The LED intensity was set to 100% and the stirring speed set to 500 rpm. The fan speed was set to 4500 rpm to maintain a temperature of 30–35°C.

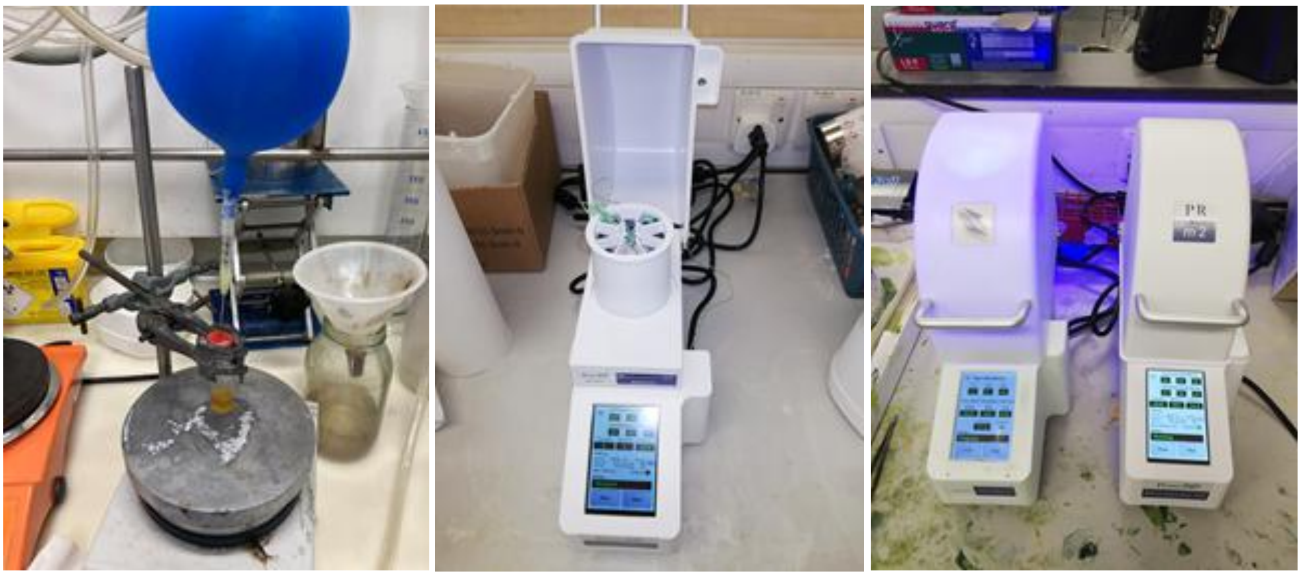

Figure S1. Photochemical reaction setup

2. EXPERIMENTAL DATA

2.1. General Procedures

2.1.1. General Procedure 1 (GP1): Preparation of Alkyl Bromides from Alcohols

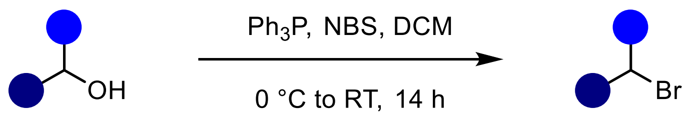

S1 **6**

A solution of alcohol **S1** (1.11 equiv) and Ph_3P (1.57 g, 6.00 mmol, 1.20 equiv) in dry DCM (6.0 mL, 0.83 M) was cooled to 0 °C before *N*-bromosuccinimide (NBS, 890 mg, 5.00 mmol, 1.00 equiv) was added slowly over 5 min. The reaction was stirred at 0 °C for 30 min before removing from the ice bath and stirring at room temperature (RT) for 14 h. The reaction was concentrated under reduced pressure, and the residue was purified by flash column chromatography to give alkyl bromide **6**.

2.1.2. General Procedure 2 (GP2): Borylation of Alkyl Bromides

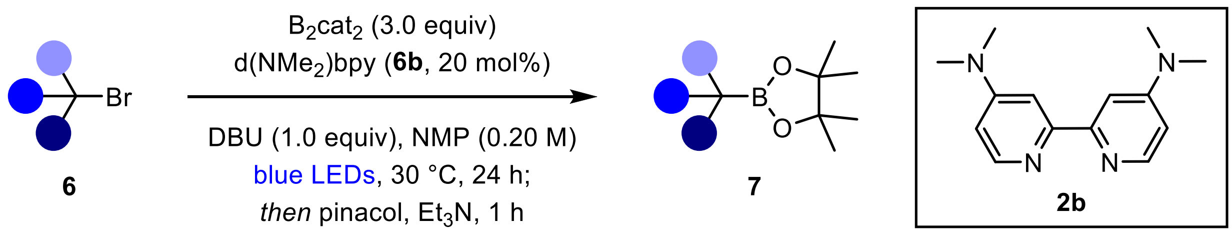

Alkyl bromide **6** (0.20 mmol, 1.0 equiv), B_2cat_2 (143 mg, 0.600 mmol, 3.00 equiv), and d(NMe_2)bpy catalyst **2b** (10 mg, 0.040 mmol, 20 mol%) were added into an oven-dried 7 mL vial containing a small magnetic stirrer bar. The vial was sealed with a septum and anhydrous NMP (1.0 mL, 0.20 M) was added. The headspace of the vial was purged with a gentle stream of N_2 for approximately 1 min before DBU (30 µL, 0.20 mmol, 1.0 equiv) was added. The vial was tightly sealed with parafilm, then placed in the photoreactor before stirring under irradiation for 24 h (see Figure S1 for experimental setup). The vial was removed from the photoreactor, a solution of pinacol (142 mg, 1.20 mmol, 6.00 equiv) in Et_3N (0.30 mL) was added to the reaction and it was stirred at RT for another 1 h. Water (10 mL) was added and the reaction mixture was extracted with EtOAc (30 + 15 mL). The combined organic extracts were washed with water (20 mL) and brine (30 mL), dried over $MgSO_4$, filtered, and concentrated under reduced pressure. The crude product was purified by flash column chromatography.

Modifications to **GP2** using dtbbpy (**2a**): Whilst the use of bipyridine catalyst **2b** generally gave the highest yields, the debrominative borylation reactions can also be performed with commercially available 4,4′-di-*tert*-butyl-2,2′-dipyridyl (dtbbpy, **2a**) as the catalyst.

GP2-modification 1: Bipyridine catalyst **2b** was replaced by dtbbpy (**2a**, 11 mg, 0.040 mmol, 20 mol%).

GP2-modification 2: Bipyridine catalyst **2b** was replaced by dtbbpy (**2a**, 11 mg, 0.040 mmol, 20 mol%), and DBU was replaced with K_2CO_3 (19 mg, 0.14 mmol, 0.70 equiv).

2.2. Synthesis of the Bipyridine Catalyst

***N*[4],*N*[4],*N*[4'],*N*[4']-Tetramethyl-[2,2'-bipyridine]-4,4'-diamine (2b)**

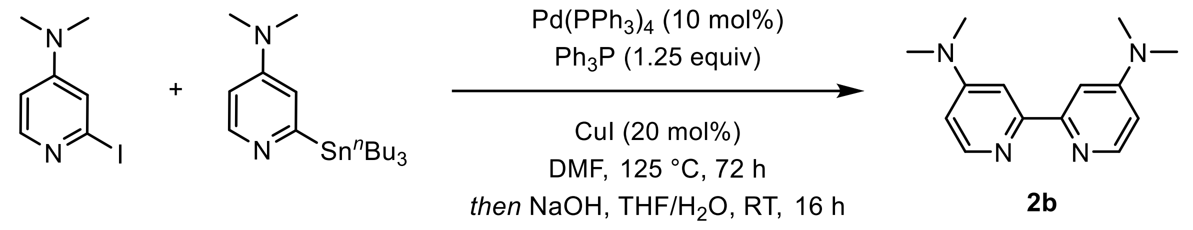

Bipyridine catalyst **2b** was synthesized according to a modified literature procedure.[1] 2-Iodo-4-*N*,*N*-dimethylamino-pyridine (1.0 g, 4.0 mmol, 1.0 equiv) was added to a Schlenk tube and dried under vacuum for 10 min. $Pd(PPh_3)_4$ (462 mg, 0.400 mmol, 10.0 mol%), PPh_3 (1.31 g, 5.00 mmol, 1.25 equiv.), and DMF (5.0 mL) were added and the mixture was degassed by three freeze-pump-thaw cycles before stirring at RT for 20 min. Concurrently, to a second Schlenk tube, *N,N*-dimethyl-2-(tributylstannyl)pyridin-4-amine (1.8 g, 4.4 mmol, 1.1 equiv.) was added and dried under vacuum for 10 min before the addition of CuI (152 mg, 0.800 mmol, 20.0 mol%) and DMF (5.0 mL). This mixture was degassed by three freeze-pump-thaw cycles, then stirred at RT for 20 min before transferring to the first Schenk tube via syringe. The reaction mixture was heated to 125 °C and stirred vigorously for 72 h. After cooling to RT, THF (50 mL) and aqueous NaOH (30 wt%, 25 mL) were added and the resulting biphasic mixture was stirring for 30 min. Additional, THF (100 mL) and aqueous NaOH (30 wt%, 25 mL) were added and the mixture was stirred for a further 16 h. The phases were separated, and the aqueous phase was extracted with Et_2O (3 × 25 mL). The combined organic phases were extracted with aqueous HCl (2 × 50 mL, 3 M), and the combined acidic aqueous extracts were washed with Et_2O (2 × 50 mL). Solid $NaHCO_3$ was slowly added into the aqueous phase until no further CO_2 generation was observed. The aqueous solution was then extracted with Et_2O (3 × 50 mL) and DCM (2 × 50 mL). The combined organic phases were dried over Na_2SO_4, filtered, and concentrated under reduced pressure. The crude product was purified by flash column chromatography (100:2:1 to 50:2:1 DCM/EtOH/NEt_3) and subsequently recrystallized from EtOAc (100%) to give **2b** (514 mg, 53%) as a pale-yellow solid.

NMR Spectroscopy (*see spectra*):

> **^{1}H NMR** (400 MHz, $CDCl_3$): δ_H 8.30 (dd, *J* = 5.9, 1.2 Hz, 2H), 7.69 (dd, *J* = 2.9, 1.1 Hz, 2H), 6.51 (dt, *J* = 5.9, 2.1 Hz, 2H), 3.08 (s, 12H);
>
> **^{1}H NMR** (500 MHz, DMF-d_7) δ_H 8.26 (d, *J* = 5.8 Hz, 2H), 7.82 (d, *J* = 2.7 Hz, 2H), 6.68 (dd, *J* = 5.8, 2.7 Hz, 2H), 3.09 (s, 12H);
>
> **^{13}C NMR** (101 MHz, $CDCl_3$): δ_C 156.9, 155.4, 149.1, 106.6, 104.2, 39.5;
>
> **^{13}C NMR** (126 MHz, DMF-d_7): δ_C 157.7, 156.3, 150.1, 107.8, 104.4, 39.6.

All recorded spectroscopic data matched those previously reported in the literature.[1]

2.3. Alkyl Bromide Synthesis

(3-Bromobutyl)benzene (6f)

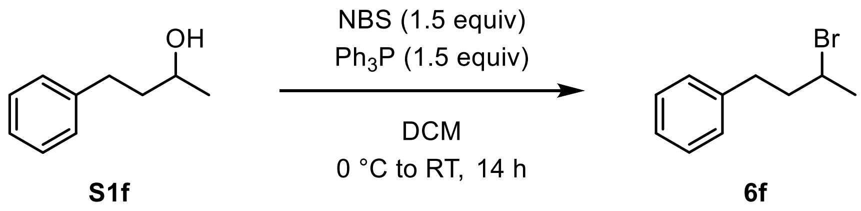

Following **GP1** with modified stoichiometry, using alcohol **S1f** (600 mg, 4.00 mmol), Ph_3P (1.57 g, 6.00 mmol, 1.50 equiv) and NBS (1.07 g, 6.00 mmol, 1.50 equiv) in DCM (6.0 mL). Purification by flash column chromatography (Biotage, Sfär silica, 25 g, 100:0 to 95:5 petroleum ether/Et_2O) gave **6f** (735 mg, 87%) as a colourless oil.

NMR Spectroscopy (*see spectra*):

> **^{1}H NMR** (400 MHz, $CDCl_3$): δ_H 7.34 – 7.28 (m, 2H), 7.25 – 7.19 (m, 3H), 4.15 – 4.05 (m, 1H), 2.88 (ddd, J = 14.1, 9.0, 5.3 Hz, 1H), 2.77 (ddd, J = 14.0, 8.9, 7.2 Hz, 1H), 2.22 – 2.01 (m, 2H), 1.75 (d, J = 6.7 Hz, 3H);
>
> **^{13}C NMR** (101 MHz, $CDCl_3$): δ_C 141.0, 128.7, 128.6, 126.2, 51.0, 42.8, 34.1, 26.7.

All recorded spectroscopic data matched those previously reported in the literature.[2]

4-Bromobutyl diisopropylcarbamate (6n)

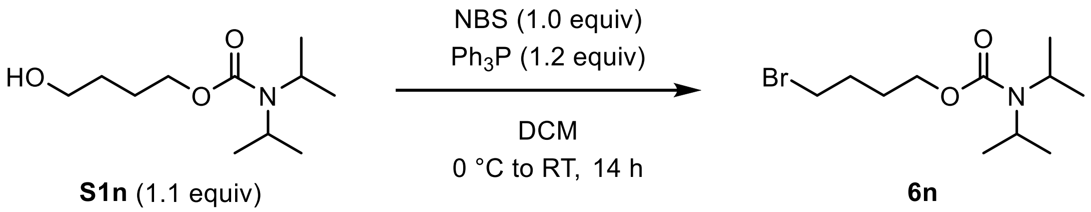

Following **GP1**, using alcohol **S1n** (1.12 g, 5.55 mmol, 1.11 equiv), Ph_3P (1.57 g, 5.00 mmol, 1.20 equiv) and NBS (890 mg, 5.00 mmol, 1.00 equiv) in DCM (6 mL). Purification by flash column chromatography (10:1 petroleum ether/Et_2O) gave **6n** (1.12 g, 86%) as a colourless oil.

NMR Spectroscopy (*see spectra*):

> **^{1}H NMR** (400 MHz, $CDCl_3$): δ_H 4.11 (t, J = 6.3 Hz, 2H), 4.09 – 3.62 (brs, 2H), 3.45 (t, J = 6.6 Hz, 2H), 2.02 – 1.91 (m, 2H), 1.87 – 1.77 (m, 2H), 1.20 (d, J = 6.8 Hz, 12H);
>
> **^{13}C NMR** (101 MHz, $CDCl_3$): δ_C 155.7, 63.6, 44.1, 33.3, 29.6, 27.8, 22.1.

IR (film): ν_{max} 2967, 1684, 1435, 1309, 1289, 1218, 1155, 1133, 1068, 1047, 771, 607, 560.

MS (EI): m/z (relative intensity) 281.1 (15) $[M(^{81}Br)]^+$, 279.1 (15) $[M(^{79}Br)]^+$, 266.1 (80) $[M(^{81}Br)–CH_3]^+$, 264.1 (80) $[M(^{79}Br)–CH_3]^+$, 135.0 (100).

(2*S*,4*R*)-5-Bromo-2,4-dimethylpentyl acetate (6o)

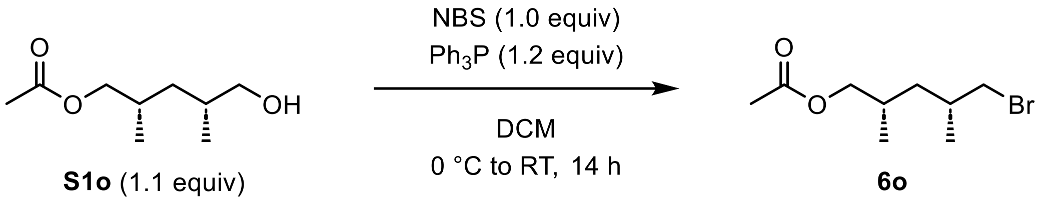

Following **GP1**, using alcohol **S1o** (967 mg, 5.55 mmol, 1.11 equiv), Ph_3P (1.57 g, 6.00 mmol, 1.20 equiv) and NBS (890 mg, 5.00 mmol, 1.00 equiv) in DCM (6 mL). Purification by flash column chromatography (30:1 petroleum ether/Et_2O) gave **6o** (972 mg, 82%) as a colourless oil.

NMR Spectroscopy (*see spectra*):

^{1}H NMR (400 MHz, $CDCl_3$): δ_H 3.96 (dd, *J* = 10.8, 5.7 Hz, 1H), 3.89 (dd, *J* = 10.8, 6.5 Hz, 1H), 3.41 (dd, *J* = 9.9, 4.5 Hz, 1H), 3.34 (dd, *J* = 9.9, 5.8 Hz, 1H), 2.08 (s, 3H), 1.98 – 1.84 (m, 2H), 1.54 (dt, *J* = 13.7, 6.8 Hz, 1H), 1.11 (dd, *J* = 14.3, 6.9 Hz, 1H), 1.06 (d, *J* = 6.6 Hz, 3H), 0.97 (d, *J* = 6.7 Hz, 3H);

^{13}C NMR (101 MHz, $CDCl_3$): δ_C 171.3, 69.1, 41.3, 38.9, 32.5, 30.1, 21.1, 19.5, 17.6.

IR (film): ν_{max} 2963, 1736, 1460, 1366, 1229, 1036, 984, 651, 606.

MS (EI): m/z (relative intensity) 178.0 (10) [M(^{81}Br)–OAc]$^+$, 176.0 (10) [M(^{79}Br)–OAc]$^+$, 97.1 (100).

9-(4-Bromobutyl)-9H-carbazole (6r)

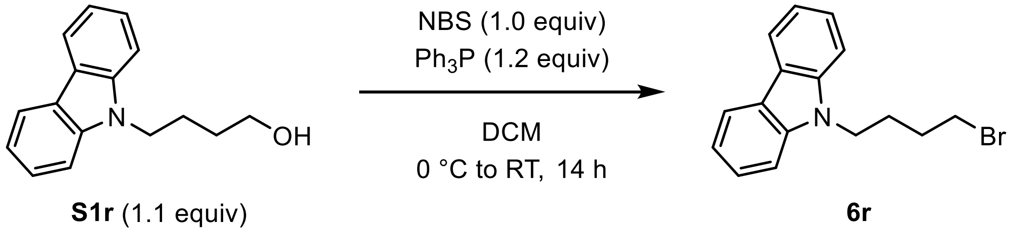

Following **GP1**, using alcohol **S1r** (1.33 g, 5.55 mmol, 1.11 equiv), Ph_3P (1.57 g, 6.00 mmol, 1.20 equiv) and NBS (890 mg, 5.00 mmol, 1.00 equiv) in DCM (6 mL). Purification by flash column chromatography (15:1 petroleum ether/Et_2O) gave **6r** (1.07 g, 71%) as a white solid.

NMR Spectroscopy (*see spectra*):

^{1}H NMR (400 MHz, $CDCl_3$): δ_H 8.10 (dd, *J* = 8.3, 1.0 Hz, 2H), 7.47 (ddd, *J* = 8.3, 7.0, 1.0 Hz, 2H), 7.40 (dd, *J* = 8.3, 1.0 Hz, 2H), 7.23 (ddd, *J* = 8.0, 7.0, 1.0 Hz, 2H), 4.35 (t, *J* = 6.9 Hz, 2H), 3.37 (t, *J* = 6.5 Hz, 2H), 2.11 – 2.02 (m, 2H), 1.96 – 1.87 (m, 2H);

^{13}C NMR (101 MHz, $CDCl_3$): δ_C 140.4, 125.9, 123.0, 120.6, 119.1, 108.7, 42.3, 33.3, 30.4, 27.8.

IR (film): ν_{max} 2928, 2870, 1593, 1485, 1466, 1452, 1327, 1247, 1225, 1196, 752, 726, 653.

All recorded spectroscopic data matched those previously reported in the literature.[3]

(3-Bromo-3-methylbutyl)benzene (6w)

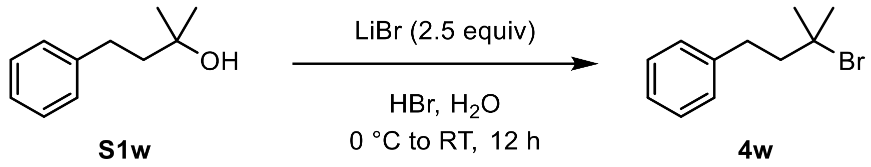

Alkyl bromide **6w** was prepared following a literature procedure.[4] A solution of LiBr (2.17 g, 25.0 mmol, 2.50 equiv) in HBr (48% aqueous, 20 mL) was cooled to 0 °C. Alcohol **S1w** (1.64 g, 10.0 mmol, 1.00 equiv) was added and the reaction was allowed to warm to RT and stirred for 12 h. The reaction mixture was extracted with DCM (2 × 20 mL) and the combined organic extracts were washed with water (20 mL), saturated aqueous $NaHCO_3$ (20 mL) and brine (20 mL), then dried over $MgSO_4$, filtered, and concentrated under reduced pressure. Purification by flash column chromatography (30:1 petroleum ether/Et_2O) gave **6w** (1.64 g, 72%) as a colourless oil.

NMR Spectroscopy (*see spectra*):

^{1}H NMR (400 MHz, $CDCl_3$): δ_H 7.35 – 7.28 (m, 2H), 7.25 – 7.18 (m, 3H), 2.86 (ddd, *J* = 17.0, 8.4, 4.7 Hz, 2H), 2.09 (ddd, *J* = 17.0, 8.0, 4.0 Hz, 2H), 1.83 (d, *J* = 1.0 Hz, 6H);

^{13}C NMR (101 MHz, $CDCl_3$): δ_C 141.7, 128.6, 128.5, 126.1, 67.7, 49.5, 34.4, 33.0.

IR (film): ν_{max} 3027, 2966, 1604, 1496, 1454, 1370, 1191, 1153, 1104, 1071, 745, 697.

All recorded spectroscopic data matched those previously reported in the literature.[5]

(4a*S*,8a*S*)-8-(2-Bromoethyl)-4,4,7,8a-tetramethyl-1,2,3,4,4a,5,6,8a-octahydronaphthalene (6x)

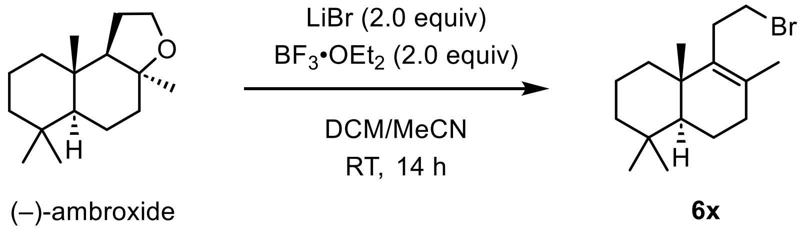

Alkyl bromide **6x** was prepared following a literature procedure.[6] To a solution of (–)-ambroxide (1.18 g, 5.00 mmol) and LiBr (869 mg, 10.0 mmol, 2.00 equiv) in DCM (10 mL) and MeCN (2 mL) at RT, BF_3•OEt_2 (1.25 mL, 10.0 mmol, 2.00 equiv) was added dropwise over 5 min. The reaction was stirred at RT for 14 h before quenching with saturated aqueous $NaHCO_3$ (50 mL) and extracting with EtOAc (60 + 30 mL). The combined organic extracts were washed with water (30 mL) and brine (30 mL), dried over $MgSO_4$, filtered, and concentrated under reduced pressure. Purification by flash column chromatography (Biotage Sfär silica, 50 g × 2, pure pentane) gave **6x** (680 mg, 45%) as a white solid.

NMR Spectroscopy (*see spectra*):

^{1}H NMR (400 MHz, $CDCl_3$): δ_H 3.36 – 3.23 (m, 2H), 2.63 (td, *J* = 13.1, 5.6 Hz, 1H), 2.47 (td, *J* = 12.9, 5.6

Hz, 1H), 2.04 – 1.90 (m, 2H), 1.86 – 1.78 (m, 1H), 1.70 – 1.60 (m, 2H), 1.59 (s, 3H), 1.50 (ddd, *J* = 13.9, 7.2, 3.9 Hz, 1H), 1.44 – 1.33 (m, 2H), 1.19 – 1.03 (m, 3H), 0.94 (s, 3H), 0.88 (s, 3H), 0.83 (s, 3H);

^{13}C NMR (126 MHz, $CDCl_3$): δ_C 138.7, 129.7, 51.9, 41.8, 38.8, 37.3, 33.8, 33.4, 33.4, 32.9, 32.0, 21.8, 20.1, 19.9, 19.1, 19.0.

All recorded spectroscopic data matched those previously reported in the literature.[6]

Epiandrosterone bromide derivative 6y

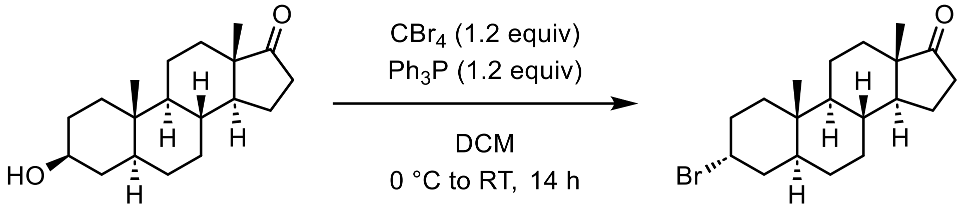

S1y **6y**

Following a modified **GP1** with CBr_4 in place of NBS, using epiandrosterone (**S1y**, 581 mg, 2.00 mmol), Ph_3P (670 mg, 2.40 mmol, 1.20 equiv) and CBr_4 (796 mg, 2.40 mmol, 1.20 equiv) in DCM (10 mL). Purification by flash column chromatography (6:1 petroleum ether/Et_2O) gave **6y** (580 mg, 82%) as a white solid.

NMR Spectroscopy (*see spectra*):

^{1}H NMR (400 MHz, $CDCl_3$): δ_H 4.73 (p, *J* = 2.8 Hz, 1H), 2.44 (ddd, *J* = 19.2, 8.9, 1.2 Hz, 1H), 2.08 (dt, *J* = 19.2, 9.0 Hz, 1H), 1.99 – 1.89 (m, 3H), 1.84 – 1.63 (m, 6H), 1.61 – 1.43 (m, 4H), 1.36 – 1.21 (m, 5H), 1.14 – 1.00 (m, 1H), 0.93 – 0.87 (m, 1H), 0.86 (s, 3H), 0.82 (s, 3H);

^{13}C NMR (101 MHz, $CDCl_3$): δ_C 221.4, 55.8, 54.2, 51.6, 47.9, 40.3, 37.4, 36.6, 36.0, 35.2, 33.0, 31.7, 31.1, 30.8, 27.7, 21.9, 20.2, 14.0, 12.5.

IR (film): ν_{max} 2930, 2851, 1736, 1443, 1371, 1250, 1205, 1053, 1012, 953, 692.

All recorded spectroscopic data matched those previously reported in the literature.[7]

(3*S*,8*S*,9*S*,10*R*,13*S*,14*S*,16*S*,17*R*)-17-((2*S*,6*R*)-7-Bromo-6-methyl-3-oxoheptan-2-yl)-10,13-dimethyl-2,3,4,7,8,9,10,11,12,13,14,15,16,17-tetradecahydro-1H-cyclopenta[a]phenanthrene-3,16-diyl diacetate (6z)

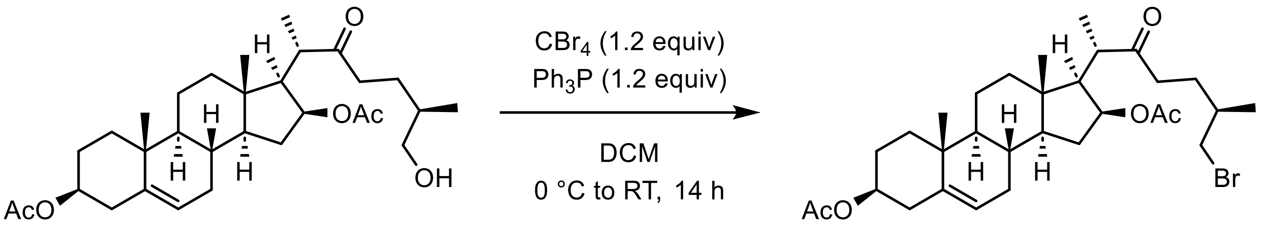

S1z **6z**

Following a modified **GP1** with CBr_4 in place of NBS, using alcohol **S1z** (181 mg, 0.350 mmol), Ph_3P (110 mg, 0.420 mmol, 1.20 equiv) and CBr_4 (139 mg, 0.420 mmol, 1.20 equiv) in DCM (10 mL). Purification by flash column chromatography (8:1 petroleum ether/Et_2O) gave **6z** (162 mg, 80%) as a white solid.

NMR Spectroscopy (*see spectra*):

^{1}H NMR (400 MHz, $CDCl_3$): δ_H 5.35 (d, *J* = 5.4 Hz, 1H), 5.02 – 4.93 (m, 1H),, 4.59 (tdd, *J* = 10.7, 6.3, 4.3 Hz, 1H), 3.37 (dd, *J* = 10.0, 4.8 Hz, 1H), 3.30 (dd, *J* = 10.0, 5.9 Hz, 1H), 3.00 – 2.88 (m, 1H), 2.61 (ddd, *J* = 17.5, 9.6, 5.9 Hz, 1H), 2.49 – 2.21 (m, 4H), 2.02 (s, 3H), 1.96 (s, 3H), 1.95 – 1.66 (m, 7H), 1.59 – 1.40 (m, 6H), 1.27 (td, *J* = 12.5, 4.8 Hz, 1H), 1.14 (d, *J* = 7.1 Hz, 3H), 1.18 – 1.09 (m, 1H), 1.06 – 0.94 (m, 3H), 1.02 (s, 3H), 1.00 (d, *J* = 6.6 Hz, 3H), 0.86 (s, 3H);

^{13}C NMR (101 MHz, $CDCl_3$): δ_C 212.6, 170.6, 169.8, 139.8, 122.4, 75.8, 73.9, 55.2, 54.1, 49.9, 43.7, 42.0, 40.9, 39.8, 38.3, 38.2, 37.0, 36.7, 35.0, 34.7, 31.7, 31.4, 28.6, 27.8, 21.5, 21.3, 20.8, 19.4, 18.7, 16.9, 13.4.

All recorded spectroscopic data matched those previously reported in the literature.[8]

Diosgenin bromide derivative 6aa

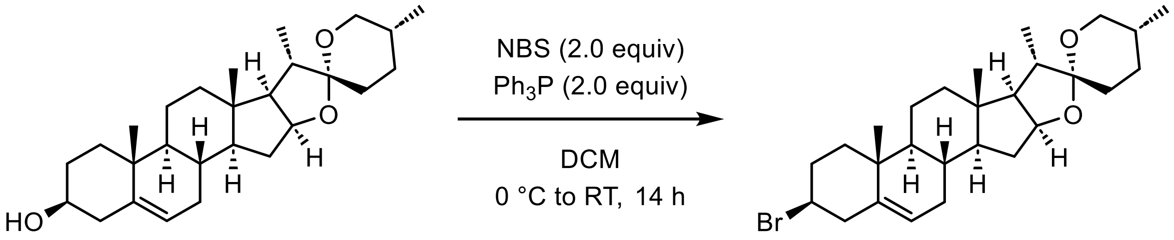

S1aa (diosgenin) **6aa**

Following **GP1** with modified stoichiometry, using diosgenin (**S1aa**, 622 mg, 1.50 mmol), Ph_3P (787 mg, 3.00 mmol, 2.00 equiv) and NBS (534 mg, 3.00 mmol, 2.00 equiv) in DCM (10 mL). Purification by flash column chromatography (Biotage, Sfär silica, 25 g, 100:0 to 93:7 petroleum ether/Et_2O) gave **6aa** (610 mg, 85%) as a white solid.

NMR Spectroscopy (*see spectra*):

^{1}H NMR (500 MHz, $CDCl_3$): δ_H 5.36 (d, *J* = 4.8 Hz, 1H), 4.40 (q, *J* = 7.4 Hz, 1H), 3.91 (tt, *J* = 12.2, 4.4 Hz,

1H), 3.47 (ddd, J = 10.9, 4.4, 2.0 Hz, 1H), 3.37 (t, J = 10.9 Hz, 1H), 2.77 – 2.71 (m, 1H), 2.58 (ddd, J = 13.6, 4.7, 2.3 Hz, 1H), 2.20 – 2.14 (m, 1H), 2.07 – 1.93 (m, 3H), 1.89 – 1.83 (m, 2H), 1.80 – 1.70 (m, 2H), 1.67 – 1.56 (m, 5H), 1.54 – 1.37 (m, 4H), 1.28 (td, J = 12.9, 12.3, 6.4 Hz, 1H), 1.20 – 1.08 (m, 3H), 1.05 (s, 3H), 0.97 (d, J = 6.9 Hz, 3H), 0.96 – 0.93 (m, 1H), 0.79 (d, J = 5.8 Hz, 3H), 0.78 (s, 3H);

^{13}C NMR (126 MHz, $CDCl_3$): δ_C 141.7, 122.2, 109.4, 80.9, 67.0, 62.2, 56.6, 52.6, 50.2, 44.4, 41.8, 40.4, 39.8, 36.7, 34.5, 32.1, 32.0, 31.5, 31.4, 30.4, 28.9, 20.8, 19.4, 17.3, 16.4, 14.7.

IR (film): ν_{max} 2950, 2900, 2869, 2846, 1454, 1376, 1261, 1051, 981, 750.

HRMS (APCI$^+$): m/z calculated for $C_{27}H_{41}O_2Br$ $[M+H]^+$ 477.2363, found 477.2364.

(3*S*,8*S*,9*S*,10*R*,13*S*,14*S*,16*R*,17*R*)-16-Bromo-10,13-dimethyl-17-((*S*)-1-((2*R*,5*R*)-5-methyl-6-oxotetrahydro-2H-pyran-2-yl)ethyl)-2,3,4,7,8,9,10,11,12,13,14,15,16,17-tetradecahydro-1H-cyclopenta[a]phenanthren-3-yl acetate (6ab)

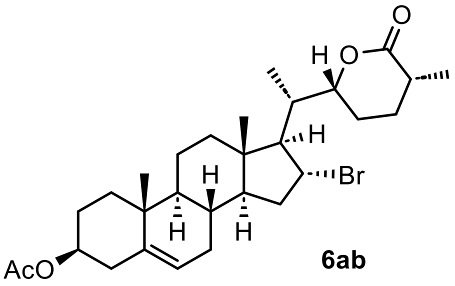

6ab

Alkyl bromide **6ab** was provided by Prof. Weisheng Tian from Shanghai Institute of Organic Chemistry.

NMR Spectroscopy (*see spectra*):

^{1}H NMR (500 MHz, $CDCl_3$): δ_H 5.37 (d, J = 5.3 Hz, 1H), 4.69 (dt, J = 11.4, 3.0 Hz, 1H), 4.64 – 4.55 (m, 1H), 4.11 (dd, J = 8.5, 6.3 Hz, 1H), 2.70 – 2.62 (m, 1H), 2.37 – 2.25 (m, 2H), 2.18 – 2.04 (m, 4H), 2.03 (s, 3H), 2.01 – 1.90 (m, 3H), 1.89 – 1.76 (m, 3H), 1.68 – 1.50 (m, 6H), 1.50 – 1.39 (m, 2H), 1.36 – 1.30 (m, 1H), 1.22 (d, J = 6.8 Hz, 3H), 1.17 – 1.10 (m, 1H), 1.07 – 1.02 (m, 1H), 1.01 (s, 3H), 0.98 (d, J = 6.7 Hz, 3H), 0.73 (s, 3H);

^{13}C NMR (126 MHz, $CDCl_3$): δ_C 176.8, 170.6, 139.9, 122.1, 79.9, 73.9, 64.0, 54.0, 53.3, 49.7, 45.3, 39.7, 39.6, 38.8, 38.2, 37.0, 36.7, 33.3, 31.8, 30.8, 27.8, 25.4, 21.6, 20.9, 20.5, 19.4, 16.6, 13.6, 12.7.

All recorded spectroscopic data matched those previously reported in the literature.[9]

(*S*)-1-((3*S*,5*S*,8*R*,9*S*,10*S*,13*S*,14*S*,16*R*,17*S*)-3-Acetoxy-16-bromo-10,13-dimethylhexadecahydro-1H-cyclopenta[a]phenanthren-17-yl)ethyl acetate (6ac)

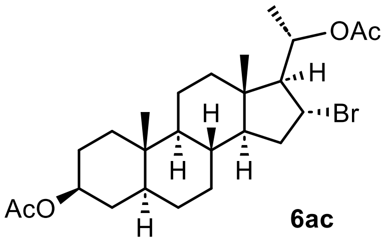

6ac

Alkyl bromide **6ac** was provided by Prof. Weisheng Tian from Shanghai Institute of Organic Chemistry.

NMR Spectroscopy (*see spectra*):

^{1}H NMR (500 MHz, $CDCl_3$): δ_H 5.11 – 5.05 (m, 1H), 4.68 (tt, *J* = 11.3, 4.9 Hz, 1H), 4.28 (ddd, *J* = 8.4, 6.5, 1.4 Hz, 1H), 2.13 – 2.07 (m, 2H), 2.06 (s, 3H), 2.01 (s, 3H), 2.00 – 1.92 (m, 1H), 1.86 – 1.78 (m, 2H), 1.71 (dt, *J* = 13.3, 3.5 Hz, 1H), 1.63 – 1.57 (m, 3H), 1.57 – 1.51 (m, 2H), 1.47 (td, *J* = 12.6, 3.9 Hz, 1H), 1.42 – 1.34 (m, 2H), 1.32 – 1.28 (m, 1H), 1.30 (d, *J* = 6.5 Hz, 3H), 1.28 – 1.14 (m, 4H), 1.05 – 0.95 (m, 2H), 0.81 (s, 3H), 0.75 (td, *J* = 11.1, 4.4 Hz, 1H), 0.63 (s, 3H);

^{13}C NMR (126 MHz, $CDCl_3$): δ_C 170.8, 170.8, 73.7, 70.4, 67.1, 54.0, 52.9, 50.2, 44.7, 44.3, 39.2, 38.8, 36.8, 35.6, 34.3, 34.1, 31.9, 28.5, 27.5, 21.6, 20.9, 20.7, 13.3, 12.3.

All recorded spectroscopic data matched those previously reported in the literature.[10]

(3*S*,3a*S*,4*R*,5a*S*,5b*R*,7a*S*,9*S*,11a*S*,11b*S*,13a*R*)-4-Bromo-9-((*tert*-butyldiphenylsilyl)oxy)-3,11a-dimethylhexadecahydro-1H,3H-naphtho[2',1':4,5]indeno[1,7a-c]furan-1-one (6ad)

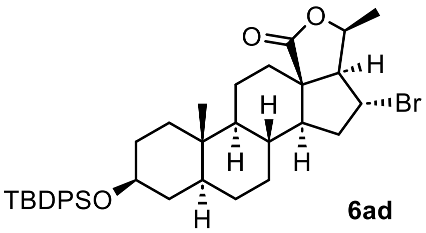

6ad

Alkyl bromide **6ad** was provided by Prof. Weisheng Tian from Shanghai Institute of Organic Chemistry.

NMR Spectroscopy (*see spectra*):

^{1}H NMR (500 MHz,$CDCl_3$): δ_H 7.68 (d, *J* = 7.1 Hz, 4H), 7.44 – 7.39 (m, 2H), 7.37 (t, *J* = 6.8 Hz, 4H), 4.60 (p, *J* = 6.3 Hz, 1H), 4.48 (dd, *J* = 7.6, 3.0 Hz, 1H), 3.59 (tt, *J* = 10.9, 5.6 Hz, 1H), 2.71 (t, *J* = 4.0 Hz, 1H), 2.29 (qd, *J* = 11.0, 3.8 Hz, 1H), 2.17 (dd, *J* = 14.1, 5.2 Hz, 1H), 2.10 – 2.03 (m, 1H), 1.85 (td, *J* = 12.1, 10.8, 5.1 Hz, 1H), 1.74 (td, *J* = 13.8, 7.5 Hz, 1H), 1.67 – 1.49 (m, 7H), 1.46 (d, *J* = 6.8 Hz, 3H),1.46 – 1.42 (m, 2H), 1.26 (td, *J* = 12.9, 3.7 Hz, 1H), 1.20 – 1.15 (m, 1H), 1.05 (s, 9H), 0.97 – 0.83 (m, 2H),0.86 (s, 3H), 0.78 (t, *J* = 13.1 Hz, 1H), 0.67 (td, *J* = 10.3, 5.2 Hz, 1H);

^{13}C NMR (126 MHz, $CDCl_3$): δ_C 178.0, 135.9, 135.1, 135.0, 129.5, 127.6, 127.6, 74.6, 72.8, 63.4, 56.8,

53.7, 52.0, 45.8, 44.9, 40.2, 38.4, 37.3, 35.8, 33.2, 32.2, 31.8, 31.5, 28.4, 27.1, 20.6, 19.3, 15.8, 12.5.

All recorded spectroscopic data matched those previously reported in the literature.[10]

Hecogenin bromide derivative 6ae

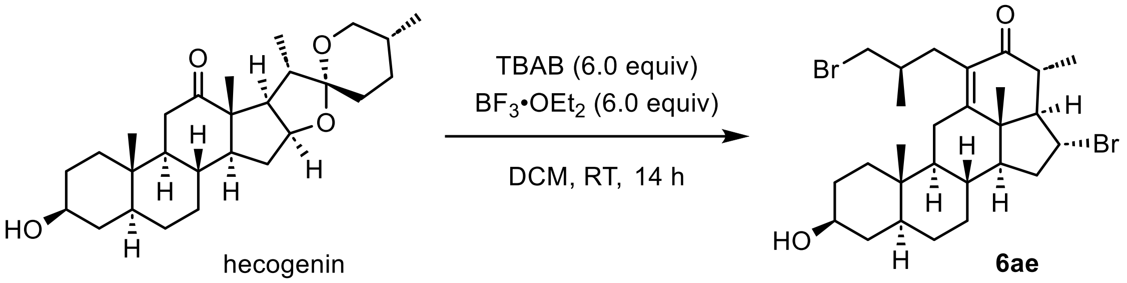

Alkyl bromide **6ae** was prepared following a literature procedure.[11] To a solution of hecogenin (301 mg, 0.700 mmol) and tetrabutylammonium bromide (TBAB, 1.35 g, 4.20 mmol, 6.00 equiv) in DCM (7 mL) at RT, BF_3•OEt_2 (0.52 mL, 4.2 mmol, 6.0 equiv) was added dropwise over 5 min. The reaction was stirred at RT for 14 h before quenching with saturated aqueous $NaHCO_3$ (20 mL) and extracting with EtOAc (40 + 20 mL). The combined organic extracts were washed with water (30 mL) and brine (30 mL), dried over $MgSO_4$, filtered, and concentrated under reduced pressure. Purification by flash column chromatography (Biotage Sfär silica, 25 g, 100:0 to 40:60 petroleum ether/Et_2O) gave **6ae** (252 mg, 65%) as a white foam.

NMR Spectroscopy (*see spectra*):

^{1}H NMR (500 MHz, $CDCl_3$): δ_H 4.20 (td, *J* = 9.6, 3.0 Hz, 1H), 3.61 (tt, *J* = 10.9, 4.6 Hz, 1H), 3.38 (dd, *J* = 9.8, 4.7 Hz, 1H), 3.31 (dd, *J* = 9.8, 5.5 Hz, 1H), 2.72 (dd, *J* = 16.1, 4.5 Hz, 1H), 2.46 (dt, *J* = 13.5, 6.7 Hz, 1H), 2.40 (dd, *J* = 13.2, 5.9 Hz, 1H), 2.35 – 2.27 (m, 1H), 2.22 (ddd, *J* = 15.0, 7.5, 3.0 Hz, 1H), 2.16 – 2.01 (m, 3H), 1.93 – 1.82 (m, 2H), 1.78 (dt, *J* = 13.0, 3.5 Hz, 1H), 1.71 (dq, *J* = 12.6, 3.3 Hz, 1H), 1.65 – 1.59 (m, 3H), 1.43 (qd, *J* = 13.5, 3.9 Hz, 1H), 1.36 – 1.26 (m, 3H), 1.33 (d, *J* = 6.8 Hz, 3H), 1.21 – 1.13 (m, 1H), 1.07 – 0.97 (m, 3H), 0.92 (d, *J* = 6.7 Hz, 3H), 0.89 (s, 3H), 0.88 (s, 3H);

^{13}C NMR (126 MHz, $CDCl_3$): δ_C 200.9, 164.0, 130.7, 71.0, 61.0, 55.4, 52.6, 47.7, 45.0, 44.9, 42.4, 42.0, 38.6, 38.1, 36.8, 36.3, 34.9, 33.8, 31.5, 30.2, 28.3, 25.9, 18.7, 15.2, 13.6, 12.4.

IR (film): ν_{max} 3300 (brs), 2967, 2928, 2859, 1735, 1658, 1445, 1372, 1239, 1043.

HRMS (ESI$^+$): m/z calculated for $C_{27}H_{40}Br_2NaO_2$ [M+Na]$^+$ 577.12873, found 577.12871.

2.4. Optimisation Studies

Table S1. Optimisation Studies

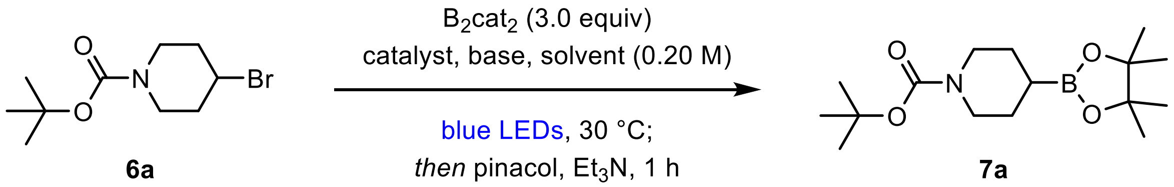

Entry	Catalyst (mol%)	Solvent	Base (equiv)	Time (h)	Conversion (%)	Yield (%)
1	-	DMAc	-	24	0	0
2	**2a** (50)	DMAc	-	24	80	55
3	**2a** (20)	DMAc	-	24	38	35
4	**2a** (20)	DMAc	K_2CO_3 (0.70)	24	88	83
5	**2a** (20)	DMAc	K_2CO_3 (0.70)	36	90	83
6	**2a** (20)	DMF	K_2CO_3 (0.70)	24	85	82
7	**2a** (20)	MeCN	K_2CO_3 (0.70)	24	26	16
8	**2a** (20)	NMP	K_2CO_3 (0.70)	24	94	92
9	**2a** (20)	NMP	DBU (1.0)	24	100	80
10	**2b** (20)	NMP	K_2CO_3 (0.70)	24	95	92
11	**2b** (20)	NMP	DBU (1.0)	24	100	92
12[a]	**2a** (20)	NMP	K_2CO_3 (0.70)	24	0	0
13[b]	**2a** (20)	NMP	K_2CO_3 (0.70)	24	0	0

Conditions: Reactions performed using 0.10 mmol of **6a** in 0.50 mL of solvent. Yields and conversions were determined by GC analysis using 1,3,5-trimethoxybenzene as the internal standard. [a] Reaction performed in the dark. [b] Reaction performed in the dark at 100 °C.

2.5. Borylation Products

tert-Butyl 4-(4,4,5,5-tetramethyl-1,3,2-dioxaborolan-2-yl)piperidine-1-carboxylate (7a)

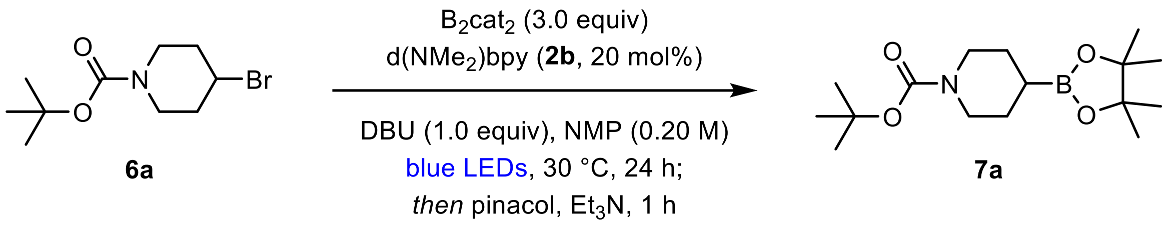

Following **GP2**, using *tert*-butyl 4-bromopiperidine-1-carboxylate (**6a**, 55 mg, 0.20 mmol), B_2cat_2 (143 mg, 0.600 mmol, 3.00 equiv), d(NMe_2)bpy catalyst **2b** (10 mg, 0.040 mmol, 20 mol%) and DBU (30 μL, 0.20 mmol, 1.0 equiv) in NMP (1.0 mL). Purification by flash column chromatography (Biotage, Sfär silica, 10 g, 100:0 to 90:10 petroleum ether/EtOAc) gave **7a** (46 mg, 74%) as a colourless oil.

Note: The borylation reaction was also performed following **GP2-modification 2**, using dtbbpy (**2a**, 11 mg, 0.040 mmol, 20 mol%) and K_2CO_3 (19 mg, 0.14 mmol, 0.70 equiv). Purification by flash column chromatography (Biotage, Sfär silica, 10 g, 100:0 to 90:10 petroleum ether/EtOAc) gave **7a** (45 mg, 72%) as a colourless oil.

NMR Spectroscopy (*see spectra*):

^{1}**H NMR** (500 MHz, $CDCl_3$): δ_H 3.77 (d, *J* = 13.0 Hz, 2H), 2.91 (t, *J* = 11.3 Hz, 2H), 1.62 (d, *J* = 13.2 Hz, 2H), 1.52 – 1.44 (m, 2H), 1.43 (s, 9H), 1.22 (s, 12H), 1.10 (td, *J* = 10.8, 4.7 Hz, 1H);

^{13}**C NMR** (126 MHz, $CDCl_3$): δ_C 155.0, 83.3, 79.2, 45.3, 44.6, 28.6, 27.1, 24.9, 19.9 (br) (the carbon attached to boron is broad due to quadrupolar relaxation).

All recorded spectroscopic data matched those previously reported in the literature.[12]

4,4,5,5-Tetramethyl-2-(tetrahydro-2H-pyran-4-yl)-1,3,2-dioxaborolane (7b)

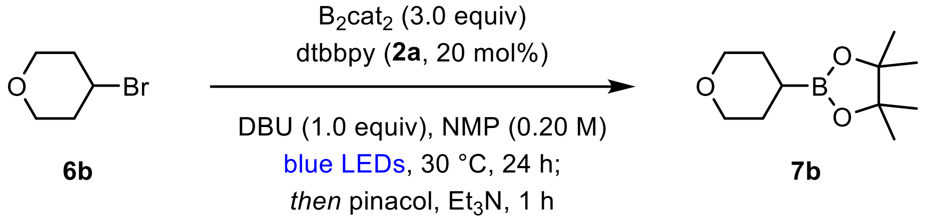

Following **GP2-modification 1**, using 4-bromotetrahydro-2H-pyran (**6b**, 33 mg, 0.20 mmol), B_2cat_2 (143 mg, 0.600 mmol, 3.00 equiv), dtbbpy catalyst **2a** (11 mg, 0.040 mmol, 20 mol%) and DBU (30 μL, 0.20 mmol, 1.0 equiv) in NMP (1.0 mL). Purification by flash column chromatography (Biotage, Sfär silica, 10 g, 100:0 to 90:10 petroleum ether/EtOAc) gave **7b** (28 mg, 66%) as a colourless oil.

NMR Spectroscopy (*see spectra*):

^{1}**H NMR** (500 MHz, $CDCl_3$): δ_H 3.81 (dt, *J* = 11.1, 3.9 Hz, 2H), 3.46 (dt, *J* = 12.0, 6.3 Hz, 2H), 1.61 (td, *J* = 7.4, 6.7, 3.5 Hz, 4H), 1.23 (s, 12H), 1.21 – 1.16 (m, 1H);

^{13}C NMR (126 MHz, $CDCl_3$): δ_C 83.3, 69.0, 27.8, 24.9, 19.0 (br) (the carbon attached to boron is broad due to quadrupolar relaxation).

All recorded spectroscopic data matched those previously reported in the literature.[13]

2-(2,3-Dihydro-1H-inden-2-yl)-4,4,5,5-tetramethyl-1,3,2-dioxaborolane (7c)

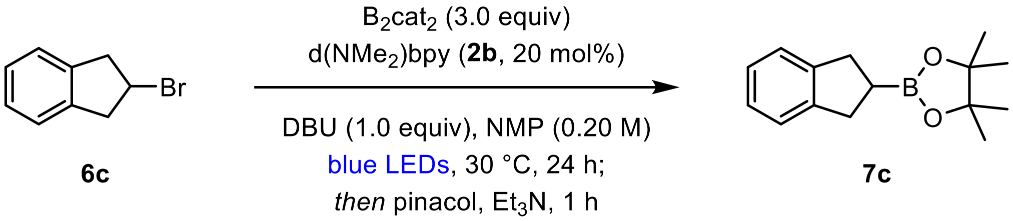

Following **GP2**, using 2-bromo-2,3-dihydro-1H-indene (**6c**, 39 mg, 0.20 mmol), B_2cat_2 (143 mg, 0.600 mmol, 3.00 equiv), d(NMe_2)bpy catalyst **2b** (10 mg, 0.040 mmol, 20 mol%) and DBU (30 µL, 0.20 mmol, 1.0 equiv) in NMP (1.0 mL). Purification by flash column chromatography on silica gel (20:1 pentane/Et_2O) yielded **7c** (40 mg, 82%) as a colourless oil.

NMR Spectroscopy (*see spectra*):

^{1}H NMR (400 MHz, $CDCl_3$): δ_H 7.21 (dd, J = 5.2, 3.4 Hz, 2H), 7.12 (dd, J = 5.5, 3.2 Hz, 2H), 3.07 (dd, J = 15.3, 9.5 Hz, 2H), 2.98 (dd, J = 15.4, 10.2 Hz, 2H), 1.89 (p, J = 9.9 Hz, 1H), 1.27 (s, 12H);

^{13}C NMR (101 MHz, $CDCl_3$): δ_C 144.5, 126.0, 124.3, 83.4, 35.3, 24.9, 21.7 (br) (the carbon attached to boron is broad due to quadrupolar relaxation);

^{11}B NMR (128 MHz, $CDCl_3$): δ_B 34.3.

IR (film): ν_{max} 2977, 2933, 1739, 1379, 1371, 1315, 1262, 1216, 1141, 971, 857, 741.

All recorded spectroscopic data matched those previously reported in the literature.[14]

2-((1*R*,4*S*)-Bicyclo[2.2.1]heptan-7-yl)-4,4,5,5-tetramethyl-1,3,2-dioxaborolane (7d)

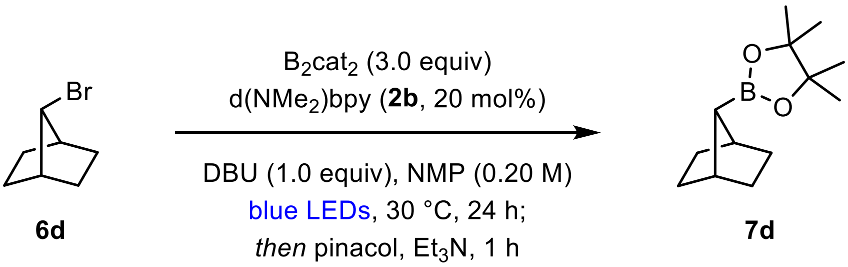

Following **GP2**, using 7-bromobicyclo[2.2.1]heptane (**6d**, 90% purity, 39 mg, 0.20 mmol), B_2cat_2 (143 mg, 0.600 mmol, 3.00 equiv), d(NMe_2)bpy catalyst **2b** (10 mg, 0.040 mmol, 20 mol%) and DBU (30 µL, 0.20 mmol, 1.0 equiv) in NMP (1.0 mL). Purification by flash column chromatography on silica gel (40:1 pentane/Et_2O) gave **7d**

(35 mg, 79%) as a colourless oil.

Note: The borylation reaction was also performed following **GP2-modification 2**, using dtbbpy (**2a**, 11 mg, 0.040 mmol, 20 mol%) and K_2CO_3 (19 mg, 0.14 mmol, 0.70 equiv). Purification by flash column chromatography (Biotage, Sfär silica, 10 g, 100:0 to 92:8 petroleum ether/Et_2O) gave **7d** (26 mg, 59%) as a colourless oil.

NMR Spectroscopy (*see spectra*):

^{1}**H NMR** (500 MHz, $CDCl_3$): δ_H 2.38 – 2.33 (m, 2H), 1.61 – 1.56 (m, 2H), 1.50 – 1.46 (m, 2H), 1.22 (s, 12H), 1.18 (d, *J* = 7.1 Hz, 2H), 1.14 (dd, *J* = 7.4, 2.0 Hz, 2H), 0.91 (s, 1H);

^{13}**C NMR** (126 MHz, $CDCl_3$): δ_C 82.9, 39.2, 34.8 (br), 31.7, 29.6, 24.9 (the carbon attached to boron is broad due to quadrupolar relaxation).

^{11}**B NMR** (128 MHz, $CDCl_3$): δ_B 34.2.

IR (film): ν_{max} 2988, 2947, 2868, 1414, 1380, 1311, 1138, 853.

HRMS (EI$^+$): calculated for $C_{13}H_{23}BO_2$ $[M–CH_3]^+$ 207.1551, found 207.1550.

2-Cycloheptyl-4,4,5,5-tetramethyl-1,3,2-dioxaborolane (7e)

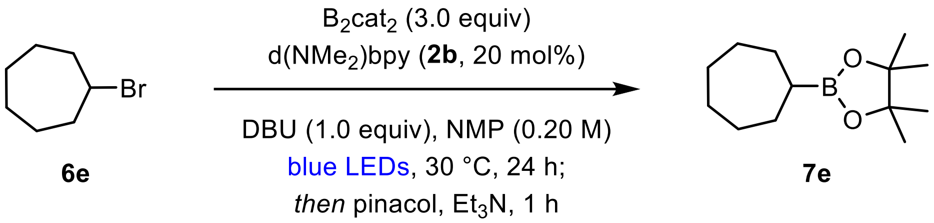

Following **GP2**, using bromocycloheptane (**6e**, 97% purity, 37 mg, 0.20 mmol), B_2cat_2 (143 mg, 0.600 mmol, 3.00 equiv), d(NMe_2)bpy catalyst **2b** (10 mg, 0.040 mmol, 20 mol%) and DBU (30 µL, 0.20 mmol, 1.0 equiv) in NMP (1.0 mL). Purification by flash column chromatography on silica gel (40:1 pentane/Et_2O) gave **7e** (38 mg, 85%) as a colourless oil.

Note: The borylation reaction was also performed following **GP2-modification 2**, using dtbbpy (**2a**, 11 mg, 0.040 mmol, 20 mol%) and K_2CO_3 (19 mg, 0.14 mmol, 0.70 equiv). Purification by flash column chromatography (Biotage, Sfär silica, 10 g, 100:0 to 93:7 petroleum ether/Et_2O) gave **7e** (27 mg, 60%) as a colourless oil.

NMR Spectroscopy (*see spectra*):

^{1}**H NMR** (500 MHz, $CDCl_3$): δ_H 1.78 – 1.70 (m, 2H), 1.69 – 1.61 (m, 2H), 1.59 – 1.51 (m, 2H), 1.51 – 1.41 (m, 6H), 1.22 (s, 12H), 1.10 – 1.03 (m, 2H);

^{13}**C NMR** (126 MHz, $CDCl_3$): δ_C 82.9, 29.7, 29.1, 28.5, 24.9, 23.3 (br) (the carbon attached to boron is broad due to quadrupolar relaxation).

IR (film): ν_{max} 2971, 2920, 1739, 1446, 1378, 1311, 1217, 1145, 970, 852.

All recorded spectroscopic data matched those previously reported in the literature.[12]

4,4,5,5-Tetramethyl-2-(4-phenylbutan-2-yl)-1,3,2-dioxaborolane (7f)

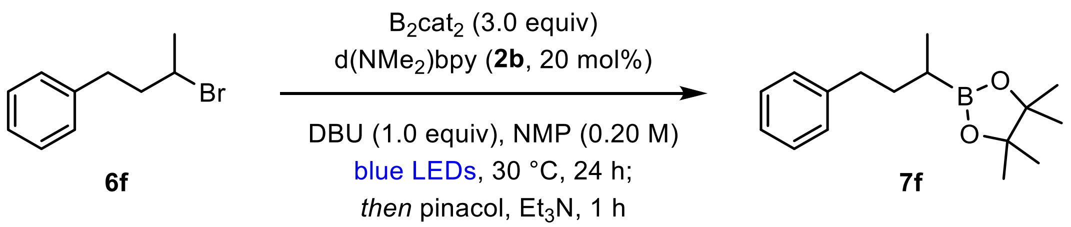

Following **GP2**, using **6f** (42 mg, 0.20 mmol), B_2cat_2 (143 mg, 0.600 mmol, 3.00 equiv), d(NMe_2)bpy catalyst **2b** (10 mg, 0.040 mmol, 20 mol%) and DBU (30 µL, 0.20 mmol, 1.0 equiv) in NMP (1.0 mL). Purification by flash column chromatography (Biotage, Sfär silica, 10 g, 100:0 to 93:7 petroleum ether/Et_2O) gave **7f** (43 mg, 83%) as a colourless oil.

Note: The borylation reaction was also performed following **GP2-modification 2**, using dtbbpy (**2a**, 11 mg, 0.040 mmol, 20 mol%) and K_2CO_3 (19 mg, 0.14 mmol, 0.70 equiv). Purification by flash column chromatography (Biotage, Sfär silica, 10 g, 100:0 to 93:7 petroleum ether/Et_2O) gave **7f** (43 mg, 83%) as a colourless oil.

NMR Spectroscopy (*see spectra*):

^{1}**H NMR** (500 MHz, $CDCl_3$): δ_H 7.29 – 7.25 (m, 2H), 7.22 – 7.14 (m, 3H), 2.63 (ddd, *J* = 9.2, 6.6, 2.1 Hz, 2H), 1.84 – 1.75 (m, 1H), 1.64 – 1.56 (m, 1H), 1.26 (s, 12H), 1.09 (dt, *J* = 13.1, 6.8 Hz, 1H), 1.03 (d, *J* = 6.9 Hz, 3H);

^{13}**C NMR** (126 MHz, $CDCl_3$): δ_C 143.2, 128.6, 128.3, 125.6, 83.0, 35.5, 35.4, 24.9, 24.9, 16.9 (br), 15.6 (the carbon attached to boron is broad due to quadrupolar relaxation).

IR (film): ν_{max} 3027, 2971, 2927, 1739, 1455, 1370, 1366, 1316, 1228, 1217, 1143, 747, 698.

All recorded spectroscopic data matched those previously reported in the literature.[15]

2-(Heptan-2-yl)-4,4,5,5-tetramethyl-1,3,2-dioxaborolane (7g)

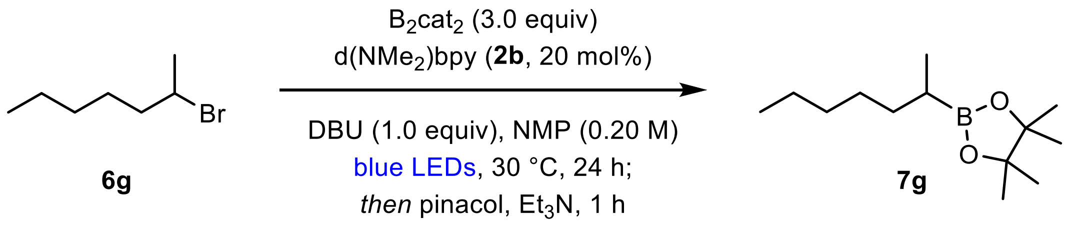

Following **GP2**, using 2-bromoheptane (**6g**, 88% purity, 41 mg, 0.20 mmol), B_2cat_2 (143 mg, 0.600 mmol, 3.00 equiv), d(NMe_2)bpy catalyst **2b** (10 mg, 0.040 mmol, 20 mol%) and DBU (30 µL, 0.20 mmol, 1.0 equiv) in NMP (1.0 mL). Purification by flash column chromatography (Biotage, Sfär silica, 10 g, 100:0 to 92:8 petroleum ether/Et_2O) gave **7g** (38 mg, 84%) as a colourless oil.

Note: The borylation reaction was also performed following **GP2-modification 2**, using dtbbpy (**2a**, 11 mg, 0.040 mmol, 20 mol%) and K_2CO_3 (19 mg, 0.14 mmol, 0.70 equiv). Purification by flash column chromatography

(Biotage, Sfär silica, 10 g, 100:0 to 92:8 petroleum ether/Et_2O) gave **7g** (22 mg, 49%) as a colourless oil.

NMR Spectroscopy (*see spectra*):

^{1}H NMR (500 MHz, $CDCl_3$): δ_H 1.33 – 1.23 (m, 8H), 1.23 (s, 12H), 1.02 – 0.96 (m, 1H), 0.95 (d, *J* = 6.0 Hz, 3H), 0.87 (t, *J* = 7.0 Hz, 3H);

^{13}C NMR (101 MHz, $CDCl_3$): δ_C 82.9, 33.4, 32.2, 28.8, 24.9, 24.9, 22.8, 15.7, 14.2 (the carbon attached to boron was not observed due to quadrupolar relaxation);

^{11}B NMR (128 MHz, $CDCl_3$): δ_B 34.5.

IR (film): ν_{max} 2956, 2926, 1739, 1462, 1371, 1314, 1228, 1217, 1144, 968, 860.

All recorded spectroscopic data matched those previously reported in the literature.[12]

2-(3,7-Dimethyloctyl)-4,4,5,5-tetramethyl-1,3,2-dioxaborolane (7h)

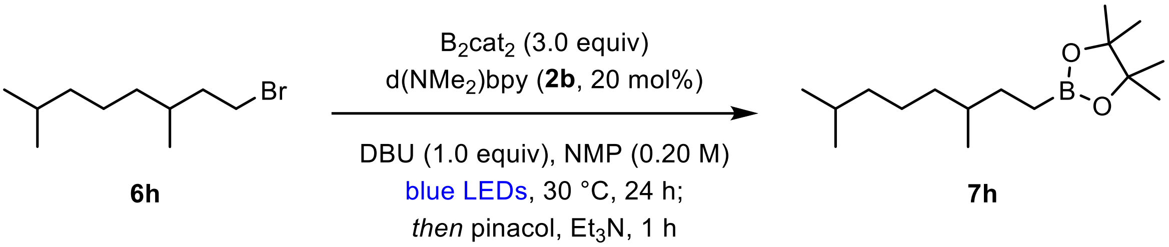

Following **GP2**, using 1-bromo-3,7-dimethyloctane (**6h**, 44 mg, 0.20 mmol), B_2cat_2 (143 mg, 0.600 mmol, 3.00 equiv), d(NMe_2)bpy catalyst **2b** (10 mg, 0.040 mmol, 20 mol%) and DBU (30 µL, 0.20 mmol, 1.0 equiv) in NMP (1.0 mL). Purification by flash column chromatography on silica gel (40:1 pentane/Et_2O) gave **7h** (46 mg, 86%) as a colourless oil.

NMR Spectroscopy (*see spectra*):

^{1}H NMR (400 MHz, $CDCl_3$): δ_H 1.56 – 1.45 (m, 1H), 1.44 – 1.36 (m, 1H), 1.36 – 1.15 (m, 17H), 1.14 – 1.09 (m, 2H), 1.07 – 0.99 (m, 1H), 0.85 (d, *J* = 7.2 Hz, 6H), 0.83 (d, *J* = 6.4 Hz, 3H), 0.81 – 0.66 (m, 2H);

^{13}C NMR (101 MHz, $CDCl_3$): δ_C 83.0, 39.5, 37.0, 35.1, 31.1, 28.1, 25.0, 25.0, 22.9, 22.8, 19.4 (the carbon attached to boron was not observed due to quadrupolar relaxation);

^{11}B NMR (128 MHz, $CDCl_3$): δ_B 34.4.

IR (film): ν_{max} 2954, 2926, 1739, 1464, 1370, 1318, 1216, 1145, 968, 847.

All recorded spectroscopic data matched those previously reported in the literature.[16]

4,4,5,5-Tetramethyl-2-phenethyl-1,3,2-dioxaborolane (7i)

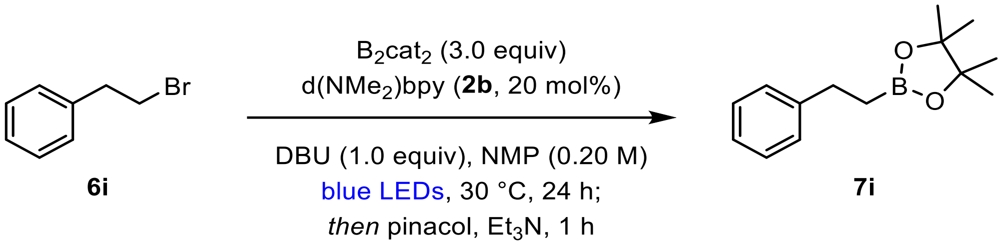

Following **GP2**, using (2-bromoethyl)benzene (**6i**, 37 mg, 0.20 mmol), B_2cat_2 (143 mg, 0.600 mmol, 3.00 equiv), d(NMe_2)bpy catalyst **2b** (10 mg, 0.040 mmol, 20 mol%) and DBU (30 µL, 0.20 mmol, 1.0 equiv) in NMP (1.0 mL). Purification by flash column chromatography on silica gel (30:1 pentane/Et_2O) gave **7i** (41 mg, 88%) as a colourless oil.

NMR Spectroscopy (*see spectra*):

^{1}**H NMR** (500 MHz, $CDCl_3$): δ_H 7.26 (t, *J* = 7.3 Hz, 2H), 7.22 (d, *J* = 7.5 Hz, 2H), 7.18 – 7.13 (m, 1H), 2.76 (t, *J* = 8.1 Hz, 2H), 1.22 (s, 12H), 1.15 (t, *J* = 8.1 Hz, 2H);

^{13}**C NMR** (126 MHz, $CDCl_3$): δ_C 144.5, 128.3, 128.1, 125.6, 83.2, 30.1, 25.0, 13.2 (br) (the carbon attached to boron is broad due to quadrupolar relaxation).

IR (film): ν_{max} 2971, 1739, 1371, 1321, 1230, 1217, 1144, 968, 849, 751, 698.

All recorded spectroscopic data matched those previously reported in the literature.[12]

4,4,5,5-Tetramethyl-2-(4-(trifluoromethyl)phenethyl)-1,3,2-dioxaborolane (7j)

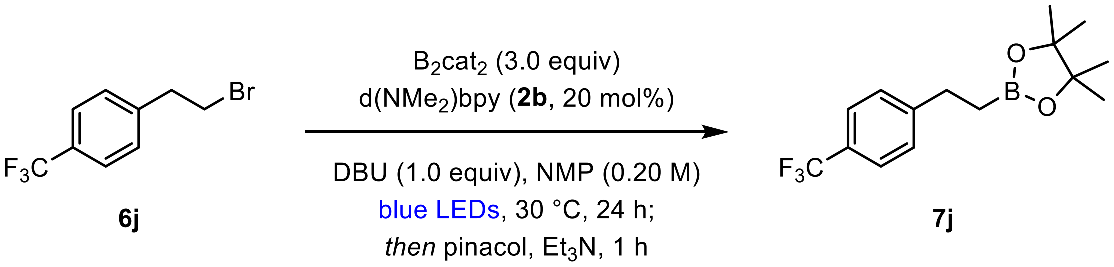

Following **GP2**, using 1-(2-bromoethyl)-4-(trifluoromethyl)benzene (**6j**, 51 mg, 0.20 mmol), B_2cat_2 (143 mg, 0.600 mmol, 3.00 equiv), d(NMe_2)bpy catalyst **2b** (10 mg, 0.040 mmol, 20 mol%) and DBU (30 µL, 0.20 mmol, 1.0 equiv) in NMP (1.0 mL). Purification by flash column chromatography on silica gel (50:1 pentane/Et_2O) gave **7j** (49 mg, 82%) as a white solid.

Melting point: 39–40 °C.

NMR Spectroscopy (*see spectra*):

^{1}**H NMR** (400 MHz, $CDCl_3$): δ_H 7.51 (d, *J* = 8.0 Hz, 2H), 7.32 (d, *J* = 9.4 Hz, 2H), 2.80 (t, *J* = 8.1 Hz, 2H), 1.22 (s, 12H), 1.15 (t, *J* = 8.1 Hz, 2H);

^{13}**C NMR** (101 MHz, $CDCl_3$): δ_C 148.7, 128.5, 127.9 (q, $^{2}J_{C\text{-}F}$ = 32.2 Hz), 125.3 (q, $^{3}J_{C\text{-}F}$ = 3.7 Hz), 124.6 (q,

$^{1}J_{C\text{-}F}$ = 272.0 Hz), 83.4, 30.0, 24.9 (the carbon attached to boron was not observed due to quadrupolar relaxation);

^{19}F NMR (377 MHz, $CDCl_3$): δ_F –62.2;

^{11}B NMR (128 MHz, $CDCl_3$): δ_B 33.7.

IR (film): ν_{max} 2982, 2940, 1739, 1417, 1373, 1325, 1161, 1143, 1112, 1068, 1018, 853, 839.

All recorded spectroscopic data matched those previously reported in the literature.[17]

4,4,5,5-Tetramethyl-2-(4-methylpent-3-en-1-yl)-1,3,2-dioxaborolane (7k)

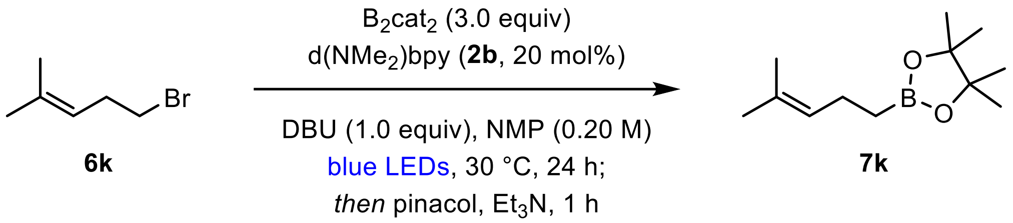

Following **GP2**, using 5-bromo-2-methylpent-2-ene (**6k**, 33 mg, 0.20 mmol), B_2cat_2 (143 mg, 0.600 mmol, 3.00 equiv), d(NMe_2)bpy catalyst **2b** (10 mg, 0.040 mmol, 20 mol%) and DBU (30 μL, 0.20 mmol, 1.0 equiv) in NMP (1.0 mL). Purification by flash column chromatography on silica gel (50:1 pentane/Et_2O) gave **7k** (30 mg, 71%) as a colourless oil.

NMR Spectroscopy (*see spectra*):

^{1}H NMR (400 MHz, $CDCl_3$): δ_H 5.11 (dddd, *J* = 7.2, 5.7, 2.9, 1.4 Hz, 1H), 2.08 (q, *J* = 7.3 Hz, 2H), 1.65 (q, *J* = 1.3 Hz, 3H), 1.59 (d, *J* = 1.3 Hz, 3H), 1.23 (s, 12H), 0.81 (t, *J* = 7.8 Hz, 2H);

^{13}C NMR (101 MHz, $CDCl_3$): δ_C 130.5, 126.9, 83.0, 25.8, 24.9, 22.6, 17.8 (the carbon attached to boron was not observed due to quadrupolar relaxation);

^{11}B NMR (128 MHz, $CDCl_3$): δ_B 34.0.

IR (film): ν_{max} 2971, 2928, 1739, 1370, 1323, 1217, 1144, 968, 850.

All recorded spectroscopic data matched those previously reported in the literature.[18]

2-(2-(Benzyloxy)ethyl)-4,4,5,5-tetramethyl-1,3,2-dioxaborolane (7l)

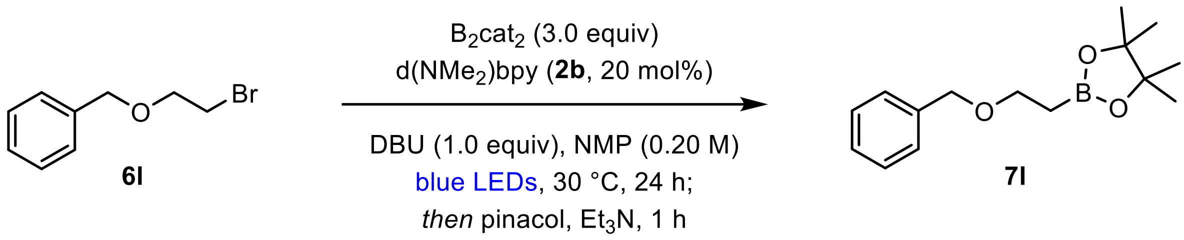

Following **GP2**, using ((2-bromoethoxy)methyl)benzene (**6l**, 43 mg, 0.20 mmol), B_2cat_2 (143 mg, 0.600 mmol, 3.00 equiv), d(NMe$_2$)bpy catalyst **2b** (10 mg, 0.040 mmol, 20 mol%) and DBU (30 µL, 0.20 mmol, 1.0 equiv) in NMP (1.0 mL). Purification by flash column chromatography on silica gel (50:1 pentane/Et_2O) gave **7l** (33 mg, 63%) as a colourless oil.

NMR Spectroscopy (*see spectra*):

^{1}H NMR (400 MHz, $CDCl_3$): δ_H 7.37 – 7.29 (m, 4H), 7.28 – 7.23 (m, 1H), 4.51 (s, 2H), 3.63 (dd, *J* = 8.2, 7.5 Hz, 2H), 1.24 (s, 12H), 1.23 – 1.20 (m, 2H);

^{13}C NMR (101 MHz, $CDCl_3$): δ_C 139.0, 128.4, 127.7, 127.5, 83.3, 72.7, 67.2, 25.0 (the carbon attached to boron was not observed due to quadrupolar relaxation);

^{11}B NMR (128 MHz, $CDCl_3$): δ_B 33.6.

IR (film): ν_{max} 2978, 2860, 1371, 1321, 1259, 1214, 1145, 1097, 967, 847, 735, 698.

All recorded spectroscopic data matched those previously reported in the literature.[19]

tert-butyldimethyl(3-(4,4,5,5-tetramethyl-1,3,2-dioxaborolan-2-yl)propoxy)silane (7m)

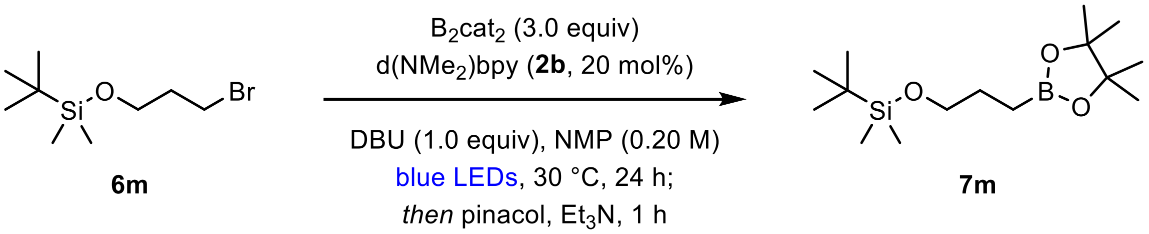

Following **GP2**, using (3-bromopropoxy)(*tert*-butyl)dimethylsilane (**6m**, 51 mg, 0.20 mmol), B_2cat_2 (143 mg, 0.600 mmol, 3.00 equiv), d(NMe$_2$)bpy catalyst **2b** (10 mg, 0.040 mmol, 20 mol%) and DBU (30 µL, 0.20 mmol, 1.0 equiv) in NMP (1.0 mL). Purification by flash column chromatography on silica gel (50:1 pentane/Et_2O) gave **7m** (39 mg, 65%) as a colourless oil.

NMR Spectroscopy (*see spectra*):

^{1}H NMR (400 MHz, $CDCl_3$): δ_H 3.56 (t, *J* = 6.9 Hz, 2H), 1.65 – 1.56 (m, 2H), 1.23 (s, 12H), 0.88 (s, 9H), 0.76 (t, *J* = 7.9 Hz, 2H), 0.03 (s, 6H);

^{13}C NMR (101 MHz, $CDCl_3$): δ_C 83.1, 65.3, 27.4, 26.2, 25.0, 18.5, –5.1 (the carbon attached to boron was

not observed due to quadrupolar relaxation).

IR (film): v_{max} 2954, 2930, 2858, 1472, 1371, 1319, 1254, 1146, 1097, 835, 775.

All recorded spectroscopic data matched those previously reported in the literature.[20]

4-(4,4,5,5-Tetramethyl-1,3,2-dioxaborolan-2-yl)butyl diisopropylcarbamate (7n)

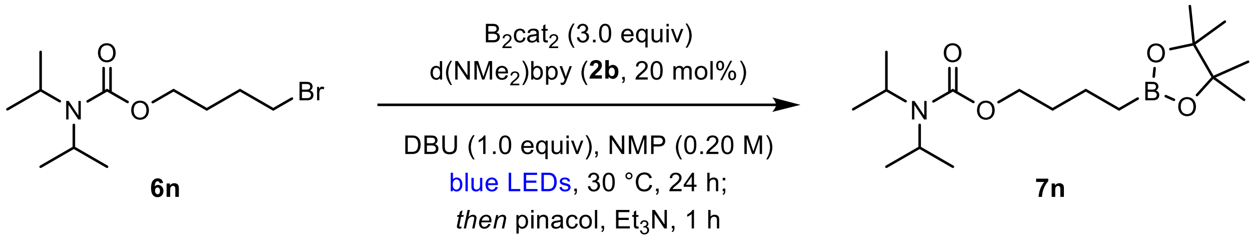

Following **GP2**, using **6n** (56 mg, 0.20 mmol), B_2cat_2 (143 mg, 0.600 mmol, 3.00 equiv), d(NMe_2)bpy catalyst **2b** (10 mg, 0.040 mmol, 20 mol%) and DBU (30 µL, 0.20 mmol, 1.0 equiv) in NMP (1.0 mL). Purification by flash column chromatography on silica gel (6:1 pentane/Et_2O) gave **7n** (28 mg, 43%) as a colourless oil.

NMR Spectroscopy (*see spectra*):

^{1}**H NMR** (400 MHz, $CDCl_3$): δ_H 4.04 (t, J = 6.5 Hz, 2H), 3.87 (br. m, 2H), 1.68 – 1.58 (m, 2H), 1.53 – 1.44 (m, 2H), 1.21 (s, 12H), 1.18 (d, J = 6.8 Hz, 12H), 0.79 (t, J = 7.7 Hz, 2H);

^{13}**C NMR** (101 MHz, $CDCl_3$): δ_C 156.1, 83.0, 64.7, 45.9 (br), 31.7, 24.9, 21.2, 20.9 (the carbon attached to boron was not observed due to quadrupolar relaxation);

^{11}**B NMR** (128 MHz, $CDCl_3$): δ_B 33.9.

IR (film): v_{max} 2970, 2938, 1739, 1688, 1435, 1370, 1309, 1290, 1217, 1144, 1069, 1048, 920, 847, 773, 732.

HRMS (ESI$^+$): calculated for $C_{17}H_{34}BNO_4Na$ $[M+Na]^+$ 350.2474, found 350.2476.

(2*S*,4*S*)-2,4-Dimethyl-5-(4,4,5,5-tetramethyl-1,3,2-dioxaborolan-2-yl)pentyl acetate (7o)

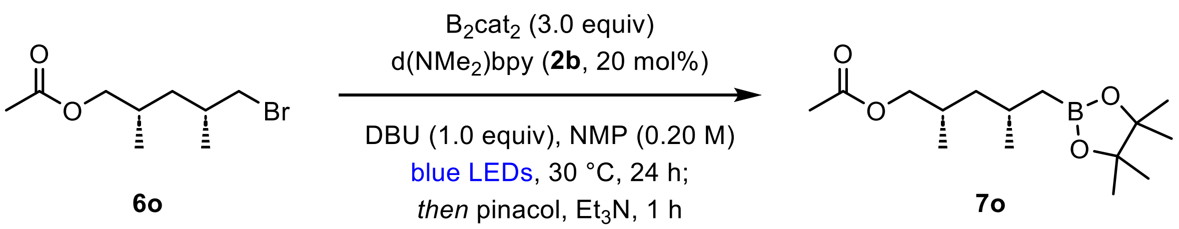

Following **GP2**, using **6o** (55 mg, 0.20 mmol), B_2cat_2 (143 mg, 0.600 mmol, 3.00 equiv), d(NMe_2)bpy catalyst **2b** (10 mg, 0.040 mmol, 20 mol%) and DBU (30 µL, 0.20 mmol, 1.0 equiv) in NMP (1.0 mL). Purification by flash column chromatography on silica gel (10:1 pentane/Et_2O) gave **7o** (52 mg, 91%) as a colourless oil.

NMR Spectroscopy (*see spectra*):

^{1}**H NMR** (400 MHz, $CDCl_3$): δ_H 3.96 (dd, *J* = 10.7, 5.4 Hz, 1H), 3.78 (dd, *J* = 10.7, 7.1 Hz, 1H), 2.03 (s, 3H), 1.90 – 1.72 (m, 2H), 1.31 – 1.25 (m, 1H), 1.23 (s, 12H), 1.03 (dt, *J* = 13.6, 7.3 Hz, 1H), 0.92 (d, *J* = 6.6 Hz, 3H), 0.90 (d, *J* = 6.7 Hz, 3H), 0.81 (dd, *J* = 15.4, 5.4 Hz, 1H), 0.60 (dd, *J* = 15.4, 8.4 Hz, 1H);

^{13}**C NMR** (126 MHz, $CDCl_3$): δ_C 171.4, 83.0, 69.8, 43.7, 30.4, 26.9, 25.0, 24.9, 23.0, 21.1, 19.5 (br),17.6 (the carbon attached to boron is broad due to quadrupolar relaxation);

^{11}**B NMR** (128 MHz, $CDCl_3$): δ_B 33.9.

IR (film): ν_{max} 2971, 2927, 1739, 1370, 1315, 1229, 1217, 1144, 1035, 970, 848.

All recorded spectroscopic data matched those previously reported in the literature.[21]

Methyl 5-(4,4,5,5-tetramethyl-1,3,2-dioxaborolan-2-yl)pentanoate (7p)

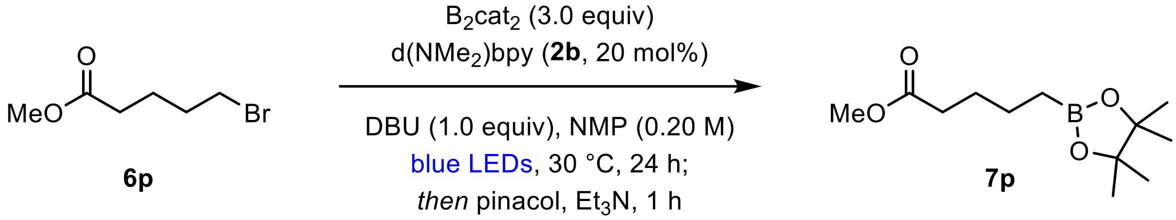

Following **GP2**, using methyl 5-bromopentanoate (**6p**, 39 mg, 0.20 mmol), B_2cat_2 (143 mg, 0.600 mmol, 3.00 equiv), d(NMe_2)bpy catalyst **2b** (10 mg, 0.040 mmol, 20 mol%) and DBU (30 µL, 0.20 mmol, 1.0 equiv) in NMP (1.0 mL). Purification by flash column chromatography on silica gel (50:1 pentane/Et_2O) gave **7p** (28 mg, 58%) as a colourless oil.

NMR Spectroscopy (*see spectra*):

^{1}**H NMR** (400 MHz, $CDCl_3$): δ_H 3.59 (s, 3H), 2.24 (t, *J* = 7.5 H, 2H), 1.65 – 1.50 (m, 2H), 1.42 – 1.33 (m, 2H), 1.18 (s, 12H), 0.72 (t, *J* = 7.7 H, 2H);

^{13}**C NMR** (101 MHz, $CDCl_3$): δ_C 174.4, 83.1, 51.5, 34.1, 27.7, 25.0, 23.8 (the carbon attached to boron was not observed due to quadrupolar relaxation);

^{11}**B NMR** (128 MHz, $CDCl_3$): δ_B 34.1.

IR (film): ν_{max} 2978, 2937, 1738, 1371, 1318, 1216, 1144, 968, 847.

All recorded spectroscopic data matched those previously reported in the literature.[22]

4-(4,4,5,5-Tetramethyl-1,3,2-dioxaborolan-2-yl)butanenitrile (7q)

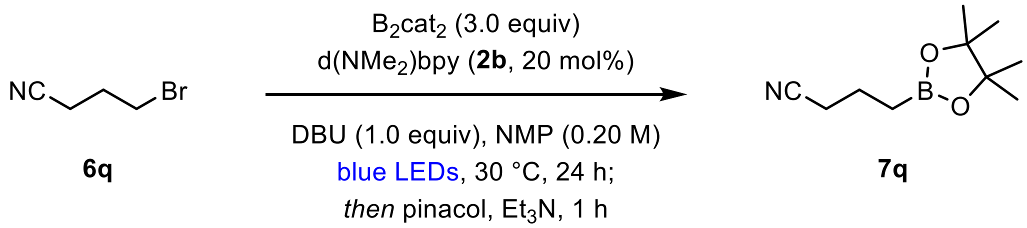

Following **GP2**, using 4-bromobutanenitrile (**6q**, 30 mg, 0.20 mmol), B_2cat_2 (143 mg, 0.600 mmol, 3.00 equiv), d(NMe_2)bpy catalyst **2b** (10 mg, 0.040 mmol, 20 mol%) and DBU (30 μL, 0.20 mmol, 1.0 equiv) in NMP (1.0 mL). Purification by flash column chromatography on silica gel (6:1 pentane/Et_2O) gave **7q** (25 mg, 64%) as a colourless oil.

NMR Spectroscopy (*see spectra*):

^{1}**H NMR** (400 MHz, $CDCl_3$): δ_H 2.34 (t, *J* = 7.2 Hz, 2H), 1.83 – 1.69 (m, 2H), 1.22 (s, 12H), 0.92 (t, *J* = 7.7 Hz, 2H);

^{13}**C NMR** (101 MHz, $CDCl_3$): δ_C 119.9, 83.5, 24.9, 20.5, 19.2 (the carbon attached to boron was not observed due to quadrupolar relaxation);

^{11}**B NMR** (128 MHz, $CDCl_3$): δ_B 33.5.

IR (film): ν_{max} 2979, 2941, 1739, 1373, 1320, 1233, 1217, 1142, 968, 846.

HRMS (ESI$^+$): calculated for $C_{10}H_{18}BNO_2Na$ $[M+Na]^+$ 218.1325, found 218.1323.

All recorded spectroscopic data matched those previously reported in the literature.[23]

9-(4-(4,4,5,5-Tetramethyl-1,3,2-dioxaborolan-2-yl)butyl)-9H-carbazole (7r)

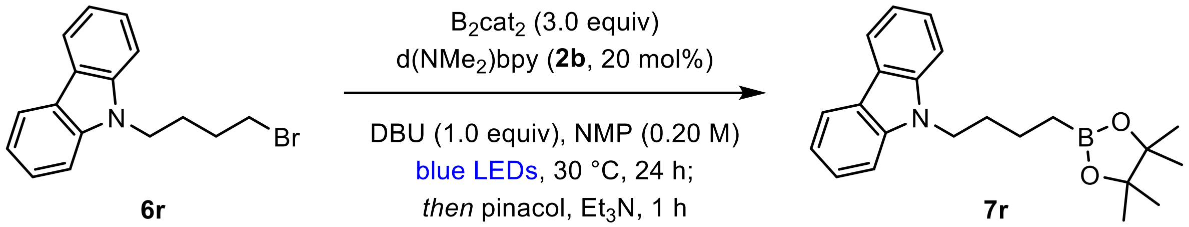

Following **GP2**, using **6r** (60 mg, 0.20 mmol), B_2cat_2 (143 mg, 0.600 mmol, 3.00 equiv), d(NMe_2)bpy catalyst **2b** (10 mg, 0.040 mmol, 20 mol%) and DBU (30 μL, 0.20 mmol, 1.0 equiv) in NMP (1.0 mL). Purification by flash column chromatography on silica gel (16:1 hexane/EtOAc to 1:1 hexane/DCM) gave **7r** (53 mg, 76%) as a colourless oil.

NMR Spectroscopy (*see spectra*):

^{1}**H NMR** (400 MHz, $CDCl_3$): δ_H 8.11 (dt, *J* = 7.8, 1.0 Hz, 2H), 7.55 – 7.38 (m, 4H), 7.29 – 7.18 (m, 2H), 4.31 (t, *J* = 7.5 Hz,, 2H), 1.96 – 1.85 (m, 2H), 1.58 (p, *J* = 7.6 Hz, 2H), 1.24 (s, 12H), 0.88 (t, *J* = 7.6 Hz, 2H);

^{13}C NMR (101 MHz, $CDCl_3$): δ_C 140.5, 125.6, 122.9, 120.4, 118.8, 108.8, 83.2, 43.0, 31.4, 24.9, 21.9 (the carbon attached to boron was not observed due to quadrupolar relaxation);

^{11}B NMR (128 MHz, $CDCl_3$): δ_B 34.2.

IR (film): ν_{max} 2976, 2930, 1597, 1484, 1462, 1453, 1371, 1324, 1242, 1142, 967, 845, 748, 742.

All recorded spectroscopic data matched those previously reported in the literature.[24]

2-(But-3-en-1-yl)-4,4,5,5-tetramethyl-1,3,2-dioxaborolane (7s)

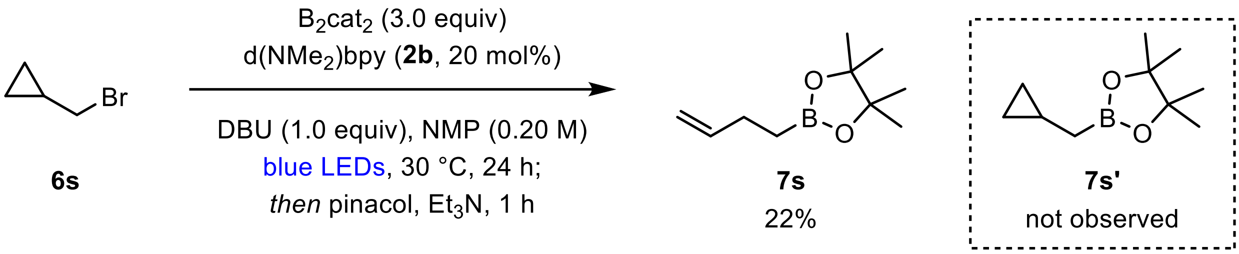

Following **GP2**, using (bromomethyl)cyclopropane (**6s**, 29 μL, 0.30 mmol), B_2cat_2 (214 mg, 0.900 mmol, 3.00 equiv), d(NMe_2)bpy catalyst **2b** (15 mg, 0.060 mmol, 20 mol%) and DBU (45 μL, 0.30 mmol, 1.0 equiv) in NMP (1.5 mL). Purification by flash column chromatography (Biotage, Sfär silica, 10 g, 100:0 to 95:5 pentane/Et_2O) gave **7s** (12 mg, 22%) as a colourless oil.

NMR Spectroscopy (*see spectra*):

^{1}H NMR (500 MHz, $CDCl_3$): δ_H 5.88 (ddt, J = 16.6, 10.2, 6.2 Hz, 1H), 4.99 (dd, J = 17.1, 1.8 Hz, 1H), 4.93 – 4.86 (m, 1H), 2.21 – 2.11 (m, 2H), 1.24 (s, 12H), 0.88 (t, J = 7.8 Hz, 2H);

^{13}C NMR (126 MHz, $CDCl_3$): δ_C 140.8, 113.3, 83.2, 28.1, 25.0, 10.4 (br) (the carbon attached to boron become broad due to quadrupolar relaxation).

All recorded spectroscopic data matched those previously reported in the literature.[25]

2-((3r,5r,7r)-Adamantan-1-yl)-4,4,5,5-tetramethyl-1,3,2-dioxaborolane (7t)

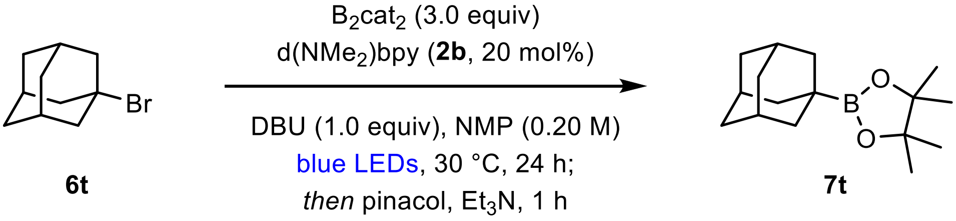

Following **GP2**, using 1-bromoadamantane (**6t**, 43 mg, 0.20 mmol), B_2cat_2 (143 mg, 0.600 mmol, 3.00 equiv), d(NMe_2)bpy catalyst **2b** (10 mg, 0.040 mmol, 20 mol%) and DBU (30 μL, 0.20 mmol, 1.0 equiv) in NMP (1.0 mL). Purification by flash column chromatography on silica gel (50:1 pentane/Et_2O) gave **7t** (43 mg, 82%) as a white solid.

Note: The borylation reaction was also performed following **GP2-modification 2**, using dtbbpy (**2a**, 11 mg, 0.040 mmol, 20 mol%) and K_2CO_3 (19 mg, 0.14 mmol, 0.70 equiv). Purification by flash column chromatography on silica gel (50:1 pentane/Et_2O) gave **7t** (42 mg, 80%) as a white solid.

NMR Spectroscopy (*see spectra*):

^{1}**H NMR** (400 MHz, $CDCl_3$): δ_H 1.86 – 1.81 (m, 3H), 1.76 – 1.73 (m, 12H), 1.20 (s, 12H);

^{13}**C NMR** (101 MHz, $CDCl_3$): δ_C 82.7, 38.1, 37.7, 27.7, 24.8 (the carbon attached to boron was not observed due to quadrupolar relaxation);

^{11}**B NMR** (128 MHz, $CDCl_3$): δ_B 33.6.

All recorded spectroscopic data matched those previously reported in the literature.[22]

5-(4,4,5,5-Tetramethyl-1,3,2-dioxaborolan-2-yl)adamantan-2-one (7u)

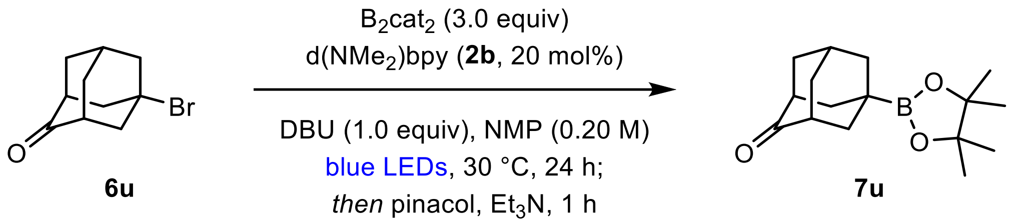

Following **GP2**, using 5-bromoadamantan-2-one (**6u**, 46 mg, 0.20 mmol), B_2cat_2 (143 mg, 0.600 mmol, 3.00 equiv), d(NMe_2)bpy catalyst **2b** (10 mg, 0.040 mmol, 20 mol%) and DBU (30 µL, 0.20 mmol, 1.0 equiv) in NMP (1.0 mL). Purification by flash column chromatography on silica gel (10:1 pentane/Et_2O) gave **7u** (41 mg, 74%) as a white solid.

NMR Spectroscopy (*see spectra*):

^{1}**H NMR** (400 MHz, $CDCl_3$): δ_H 2.51 (t, J = 3.0 Hz, 2H), 2.14 – 2.04 (m, 4H), 2.04 – 1.95 (m, 5H), 1.93 (t, J = 2.4 Hz, 2H), 1.21 (s, 12H);

^{13}**C NMR** (101 MHz, $CDCl_3$): δ_C 218.9, 83.4, 46.7, 40.6, 39.2, 37.0, 27.0, 24.8 (the carbon attached to boron was not observed due to quadrupolar relaxation);

^{11}**B NMR** (128 MHz, $CDCl_3$): δ_B 33.4.

IR (film): ν_{max} 2971, 2925, 1738, 1725, 1440, 1374, 1366, 1217, 1062, 889.

HRMS (ESI$^+$): calculated for $C_{16}H_{25}BO_3Na$ $[M+Na]^+$ 299.1792, found 299.1805.

2,2'-(Bicyclo[4.1.0]heptane-7,7-diyl)bis(4,4,5,5-tetramethyl-1,3,2-dioxaborolane) (7v)

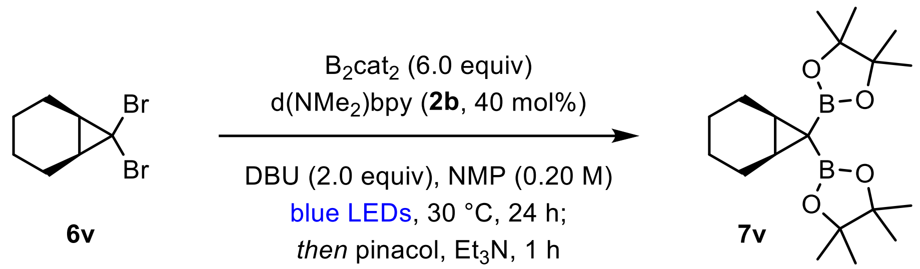

Following **GP2**, using 7,7-dibromobicyclo[4.1.0]heptane (**6v**, 97% purity, 53 mg, 0.20 mmol), B_2cat_2 (286 mg, 1.20 mmol, 6.00 equiv), d(NMe_2)bpy catalyst **2b** (19 mg, 0.080 mmol, 40 mol%) and DBU (60 µL, 0.40 mmol, 2.0 equiv) in NMP (1.0 mL). Purification by flash column chromatography (Biotage, Sfär silica, 10 g, 100:0 to 92:8 petroleum ether/Et_2O) gave **7v** (44 mg, 63%) as a colourless oil.

Note: The borylation reaction was also performed following **GP2-modification 2**, using dtbbpy (**2a**, 22 mg, 0.080 mmol, 40 mol%) and K_2CO_3 (39 mg, 0.28 mmol, 1.4 equiv). Purification by flash column chromatography (Biotage, Sfär silica, 10 g, 100:0 to 94:6 hexane/EtOAc) gave **7v** (35 mg, 50%) as a colourless oil.

NMR Spectroscopy (*see spectra*):

^{1}**H NMR** (400 MHz, $CDCl_3$): δ_H 1.91 – 1.76 (m, 4H), 1.33 – 1.28 (m, 2H), 1.28 (s, 12H), 1.25 – 1.17 (m, 3H), 1.15 (s, 12H), 1.15 – 1.10 (m, 1H);

^{13}**C NMR** (101 MHz, $CDCl_3$): δ_C 82.7, 25.0, 24.9, 23.0, 21.9, 19.3 (the carbon attached to boron was not observed due to quadrupolar relaxation).

IR (film): ν_{max} 2977, 2828, 2869, 2853, 1379, 1360, 1324, 1290, 1140, 1089, 853.

HRMS (ESI^+): calculated for $C_{19}H_{35}B_2O_4$ $[M+H]^+$ 349.2723, found 349.2729; calculated for $C_{33}H_{53}BO_4Na$ $[M+Na]^+$ 371.2542, found 371.2551.

4,4,5,5-Tetramethyl-2-(2-methyl-4-phenylbutan-2-yl)-1,3,2-dioxaborolane (7w)

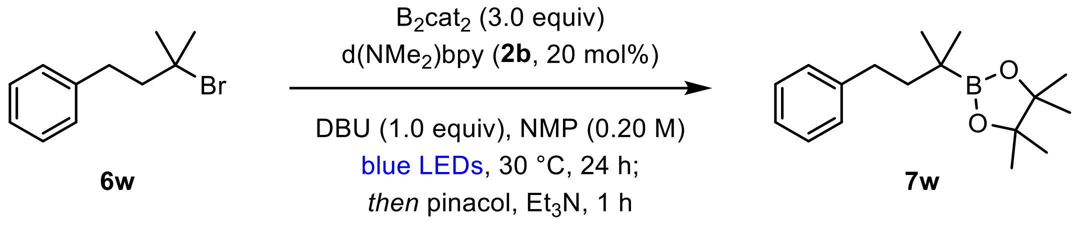

Following **GP2**, using **6w** (45 mg, 0.20 mmol), B_2cat_2 (143 mg, 0.600 mmol, 3.00 equiv), d(NMe_2)bpy catalyst **2b** (10 mg, 0.040 mmol, 20 mol%) and DBU (30 µL, 0.20 mmol, 1.0 equiv) in NMP (1.0 mL). Purification by flash column chromatography on silica gel (50:1 pentane/Et_2O) gave **7w** (24 mg, 44%) as a colourless oil.

NMR Spectroscopy (*see spectra*):

^{1}**H NMR** (400 MHz, $CDCl_3$): δ_H 7.30 – 7.14 (m, 5H), 2.64 – 2.52 (m, 2H), 1.66 – 1.56 (m, 2H), 1.27 (s, 12H),

1.02 (s, 6H);

^{13}C NMR (101 MHz, $CDCl_3$): δ_C 143.8, 128.5, 128.4, 125.6, 83.1, 43.7, 33.2, 24.9, 24.9 (the carbon attached to boron was not observed due to quadrupolar relaxation);

^{11}B NMR (128 MHz, $CDCl_3$): δ_B 34.9.

All recorded spectroscopic data matched those previously reported in the literature.[26]

4,4,5,5-Tetramethyl-2-(2-((4a*S*,8a*S*)-2,5,5,8a-tetramethyl-3,4,4a,5,6,7,8,8a-octahydronaphthalen-1-yl)ethyl)-1,3,2-dioxaborolane (7x)

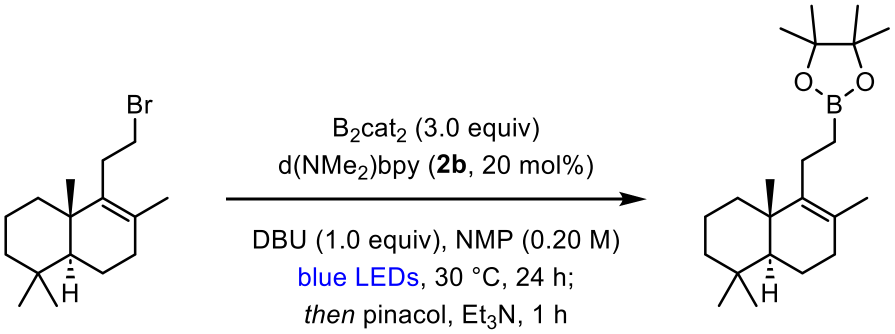

6x **7x**

Following **GP2**, using **6x** (60 mg, 0.20 mmol), B_2cat_2 (143 mg, 0.600 mmol, 3.00 equiv), d(NMe_2)bpy catalyst **2b** (10 mg, 0.040 mmol, 20 mol%) and DBU (30 μL, 0.20 mmol, 1.0 equiv) in NMP (1.0 mL). Purification by flash column chromatography (Biotage, Sfär silica, 10 g, 100:0 to 95:5 petroleum ether/Et_2O) gave **7x** (56 mg, 81%) as a colourless oil.

Note: The borylation reaction was also performed following **GP2-modification 2**, using dtbbpy (**2a**, 11 mg, 0.040 mmol, 20 mol%) and K_2CO_3 (19 mg, 0.14 mmol, 0.70 equiv). Purification by flash column chromatography (Biotage, Sfär silica, 10 g, 100:0 to 95:5 petroleum ether/Et_2O) gave **7x** (43 mg, 62%) as a colourless oil.

NMR Spectroscopy (*see spectra*):

^{1}H NMR (500 MHz, $CDCl_3$): δ_H 2.14 – 2.06 (m, 1H), 2.06 – 1.88 (m, 3H), 1.85 – 1.80 (m, 1H), 1.65 – 1.53 (m, 2H), 1.55 (s, 3H), 1.48 – 1.42 (m, 1H), 1.41 – 1.33 (m, 2H), 1.24 (s, 12H), 1.19 – 1.08 (m, 3H), 0.92 (s, 3H), 0.86 (s, 3H), 0.86 – 0.83 (m, 2H), 0.81 (s, 3H);

^{13}C NMR (126 MHz, $CDCl_3$): δ_C 143.0, 124.8, 83.0, 52.1, 42.0, 39.2, 37.0, 33.8, 33.5, 25.0, 21.9, 21.6, 20.2, 19.6, 19.2, 12.9 (br) (the carbon attached to boron is broad due to quadrupolar relaxation).

IR (film): ν_{max} 2979, 2937, 2865, 1468, 1369, 1313, 1274, 1146, 967, 850.

HRMS (EI^+): calculated for $C_{22}H_{39}BO_2$ $[M]^+$ 346.3038, found 346.3034.

(3*S*,5*S*,8*R*,9*S*,10*S*,13*S*,14*S*)-10,13-Dimethyl-3-(4,4,5,5-tetramethyl-1,3,2-dioxaborolan-2-yl)hexadecahydro-17*H*-cyclopenta[*a*]phenanthren-17-one (7y)

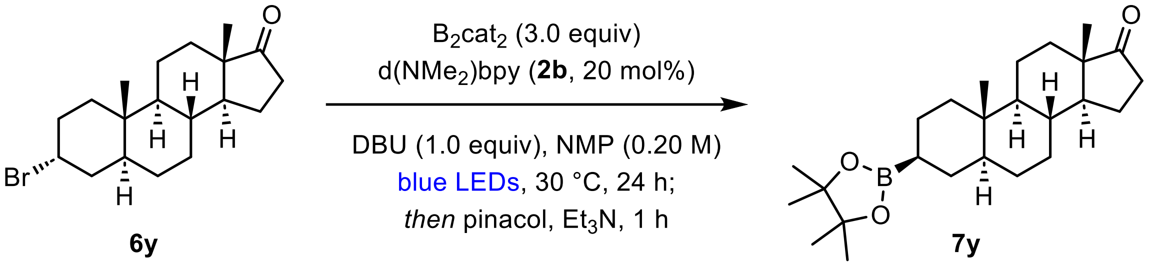

Following **GP2**, using **6y** (71 mg, 0.20 mmol), B_2cat_2 (143 mg, 0.600 mmol, 3.00 equiv), d(NMe_2)bpy catalyst **2b** (10 mg, 0.040 mmol, 20 mol%) and DBU (30 µL, 0.20 mmol, 1.0 equiv) in NMP (1.0 mL). Purification by flash column chromatography on silica gel (50:1 pentane/Et_2O) gave **7y** (59 mg, 74%) as a white solid. The d.r. of **7y** was determined to be 94:6 by ^1H NMR analysis after stereospecific oxidation of the boronic ester group to the corresponding alcohol.

NMR Spectroscopy (*see spectra*):

^{1}H NMR (400 MHz, $CDCl_3$): δ_H 2.40 (ddd, *J* = 19.1, 8.9, 1.1 Hz, 1H), 2.03 (dt, *J* = 19.1, 9.0 Hz, 1H), 1.90 (dddd, *J* = 12.2, 8.9, 5.7, 1.1 Hz, 1H), 1.80 – 1.72 (m, 2H), 1.67 (tt, *J* = 12.9, 3.1 Hz, 2H), 1.61 – 1.42 (m, 3H), 1.42 – 1.35 (m, 1H), 1.35 – 1.14 (m, 20H), 1.11 – 1.01 (m, 1H), 1.01 – 0.87 (m, 3H), 0.83 (s, 3H), 0.79 (s, 3H), 0.71 (ddd, *J* = 11.7, 10.3, 4.2 Hz, 1H);

^{13}C NMR (101 MHz, $CDCl_3$): δ_C 221.6, 82.9, 54.9, 51.7, 48.0, 47.9, 39.5, 36.4, 36.0, 35.2, 31.7, 31.1, 30.2, 28.7, 24.9, 24.9, 23.5, 21.9, 20.2, 13.9, 12.5 (the carbon attached to boron was not observed due to quadrupolar relaxation);

^{11}B NMR (128 MHz, $CDCl_3$): δ_B 32.7.

IR (film): ν_{max} 2972, 2914, 2853, 1733, 1447, 1380, 1319, 1216, 1143, 1054, 1014, 856.

All recorded spectroscopic data matched those previously reported in the literature.[21]

Determination of the d.r. of **7y**:

The d.r. of boronic ester **7y** could not be determined by ^1H NMR analysis due to overlapping signals. Therefore, a small amount of **7y** was oxidized to corresponding alcohol **S1y** and subsequent ^1H NMR analysis indicated a d.r. of 94:6.

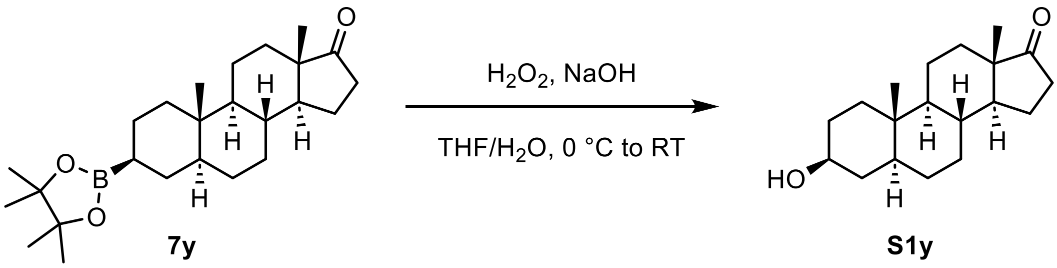

To a solution of **7y** (10 mg, 0.025 mmol) in THF (0.5 mL), a premixed solution of 3 M aqueous NaOH/30% aqueous H_2O_2 (2:1, 0.50 mL) was added dropwise at 0 °C. The reaction was removed from the ice bath and stirred at RT for 1 h. The reaction was quenched with 10% aqueous $Na_2S_2O_3$ (5 mL), diluted with water (5 mL), and extracted with EtOAc (2 × 20 mL). The combined organic extracts were washed with brine (10 mL), dried over $MgSO_4$, filtered, and concentrated under reduced pressure to give **S1y** (6.9 mg, 95%). Comparison of ^{1}H NMR spectra of the oxidation product to those of commercially available epiandrosterone (**S1y**) and androsterone (**S1y′**) showed the d.r. to be 94:6 in favour of **S1y** (Figure S2).

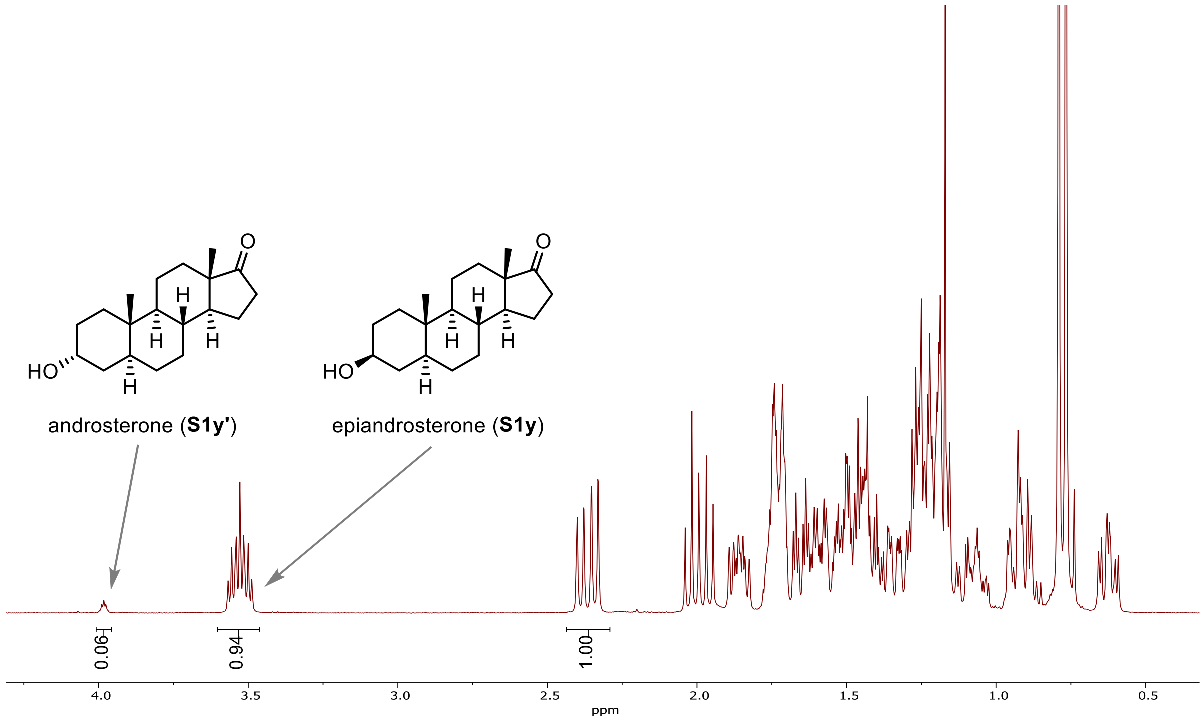

Figure S2. Determination of the d.r. of **7y**

(3*S*,8*S*,9*S*,10*R*,13*S*,14*S*,16*S*,17*R*)-10,13-Dimethyl-17-((2*S*,6*S*)-6-methyl-3-oxo-7-(4,4,5,5-tetramethyl-1,3,2-dioxaborolan-2-yl)heptan-2-yl)-2,3,4,7,8,9,10,11,12,13,14,15,16,17-tetradecahydro-1*H*-cyclopenta[*a*]phenanthrene-3,16-diyl diacetate (7z)

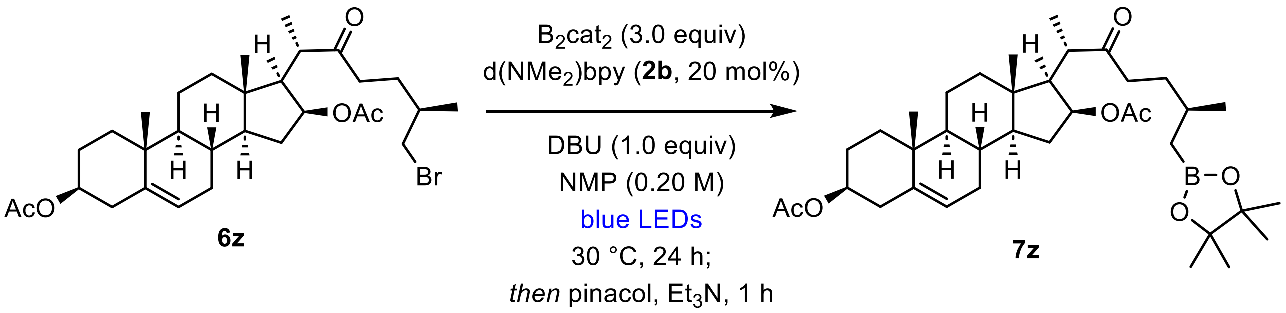

Following **GP2**, using **6z** (116 mg, 0.200 mmol), B_2cat_2 (143 mg, 0.600 mmol, 3.00 equiv), d(NMe_2)bpy catalyst **2b** (10 mg, 0.040 mmol, 20 mol%) and DBU (30 µL, 0.20 mmol, 1.0 equiv) in NMP (1.0 mL). Purification by flash column chromatography on silica gel (50:1 pentane/Et_2O) gave **7z** (103 mg, 82%) as a white solid.

NMR Spectroscopy (*see spectra*):

^{1}H NMR (400 MHz, $CDCl_3$): δ_H 5.36 (d, *J* = 5.0 Hz, 1H), 5.04 – 4.91 (m, 1H), 4.65 – 4.53 (m, 1H), 3.03 – 2.87 (m, 1H), 2.57 (ddd, *J* = 17.3, 10.8, 5.3 Hz, 1H), 2.41 (dd, *J* = 8.1, 6.4 Hz, 1H), 2.36 – 2.25 (m, 3H), 2.03 (s, 3H), 1.95 (s, 3H), 1.95 – 1.80 (m, 5H), 1.72 – 1.61 (m, 1H), 1.60 – 1.56 (m, 1H), 1.54 – 1.36 (m, 5H), 1.29 (dd, *J* = 12.6, 4.9 Hz, 1H), 1.24 (s, 12H), 1.18 – 1.14 (m, 1H), 1.13 (d, *J* = 7.1 Hz, 3H), 1.08 – 0.95 (m, 4H), 1.02 (s, 3H), 0.91 (d, *J* = 6.7 Hz, 3H), 0.86 (s, 3H), 0.85 – 0.79 (m, 1H), 0.67 (dd, *J* = 15.4, 8.5 Hz, 1H);

^{13}C NMR (101 MHz, $CDCl_3$): δ_C 213.5, 170.6, 169.9, 139.7, 122.4, 83.1, 75.8, 74.0, 55.2, 54.1, 49.9, 43.7, 42.0, 39.8, 39.3, 38.2, 37.0, 36.7, 35.0, 33.0, 31.7, 31.4, 29.8, 29.4, 27.9, 25.0, 24.9, 22.2, 21.5, 21.3, 20.9, 19.4, 16.9, 13.4 (the carbon attached to boron was not observed due to quadrupolar relaxation);

^{11}B NMR (128 MHz, $CDCl_3$): δ_B 34.7.

IR (film): ν_{max} 3026, 2971, 1738, 1436, 1366, 1229, 1217, 1206, 1145, 1092, 1035, 903, 772.

HRMS (ESI^+): m/z calculated for $C_{37}H_{59}BNaO_7$ $[M+Na]^+$ 649.4253, found 649.4230.

4,4,5,5-Tetramethyl-2-((4*S*,5'*R*,6a*R*,6b*S*,8a*S*,8b*R*,9*S*,10*R*,11a*S*,12a*S*,12b*S*)-5',6a,8a,9-tetramethyl-1,3,3',4,4',5,5',6,6a,6b,6',7,8,8a,8b,9,11a,12,12a,12b-icosahydrospiro[naphtho[2',1':4,5]indeno[2,1-*b*]furan-10,2'-pyran]-4-yl)-1,3,2-dioxaborolane (7aa)

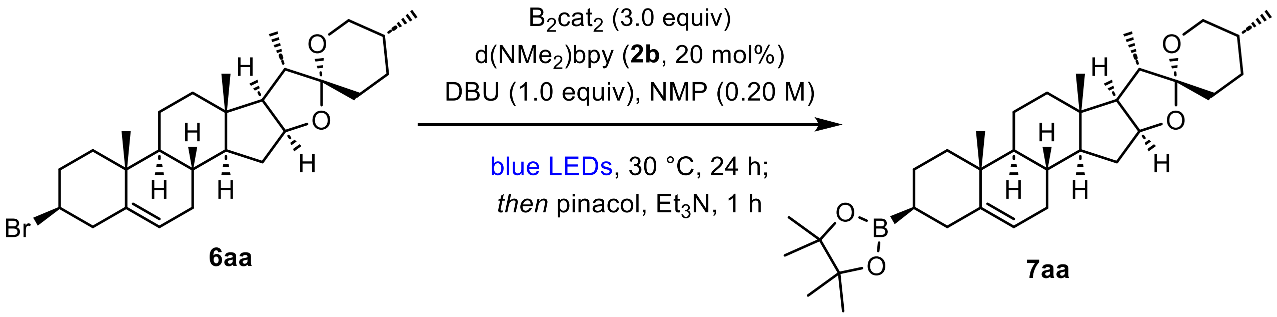

Following **GP2**, using **6aa** (96 mg, 0.20 mmol), B_2cat_2 (143 mg, 0.600 mmol, 3.00 equiv), $d(NMe_2)bpy$ catalyst **2b** (10 mg, 0.040 mmol, 20 mol%) and DBU (30 µL, 0.20 mmol, 1.0 equiv) in NMP (1.0 mL). Purification by flash column chromatography (Biotage, Sfär silica, 10 g, 100:0 to 93:7 petroleum ether/Et_2O) gave **7aa** (50 mg, 48%) as a white solid, and unreacted starting material **6aa** (41 mg, 43%). The d.r. of **7aa** was determined to be 84:16 by ^{1}H NMR analysis.

NMR Spectroscopy (*see spectra*):

^{1}H NMR (500 MHz, $CDCl_3$, for all isomers): δ_H 5.33 (d, *J* = 5.1 Hz, 0.14 H), 5.26 (d, *J* = 5.1 Hz, 0.86H), 4.40 (q, *J* = 7.4 Hz, 1H), 3.51 – 3.43 (m, 1H), 3.37 (t, *J* = 10.9 Hz, 1H), 2.19 (t, *J* = 13.9 Hz, 1H), 2.06 – 1.93 (m, 3H), 1.89 – 1.82 (m, 2H), 1.78 – 1.57 (m, 7H), 1.53 – 1.36 (m, 5H), 1.31 – 1.25 (m, 2H), 1.23 (s, 12H), 1.17 – 1.02 (m, 3H), 1.02 – 0.98 (m, 1H), 1.00 (s, 3H), 0.97 (d, *J* = 6.9 Hz, 3H), 0.90 – 0.86 (m, 1H), 0.79 (d, *J* = 6.4 Hz, 3H), 0.78 (s, 3H);

^{13}C NMR (126 MHz, $CDCl_3$): δ_C 144.0, 118.4, 109.4, 83.0, 81.0, 67.0, 62.3, 56.8, 50.7, 41.8, 41.0, 40.4,

40.0, 37.6, 34.0, 32.2, 32.0, 31.5, 31.5, 30.5, 29.0, 24.9, 24.9, 24.2, 20.7, 19.7, 17.3, 16.4, 14.7 (the carbon attached to boron was not observed due to quadrupolar relaxation).

IR (film): ν_{max} 2975, 2951, 2926, 2869, 1458, 1384, 1312, 1145, 981, 899.

HRMS (ESI$^+$): calculated for $C_{33}H_{54}BO_4$ [M+H]$^+$ 525.4115, found 525.4118; calculated for $C_{33}H_{53}BO_4Na$ [M+Na]$^+$ 547.3935, found 547.3948.

All recorded spectroscopic data matched those previously reported in the literature.[27]

(3*S*,8*S*,9*S*,10*R*,13*S*,14*S*,16*R*,17*R*)-10,13-Dimethyl-17-((*S*)-1-((2*R*,5*R*)-5-methyl-6-oxotetrahydro-2H-pyran-2-yl)ethyl)-16-(4,4,5,5-tetramethyl-1,3,2-dioxaborolan-2-yl)-2,3,4,7,8,9,10,11,12,13,14,15,16,17-tetradecahydro-1*H*-cyclopenta[*a*]phenanthren-3-yl acetate (7ab)

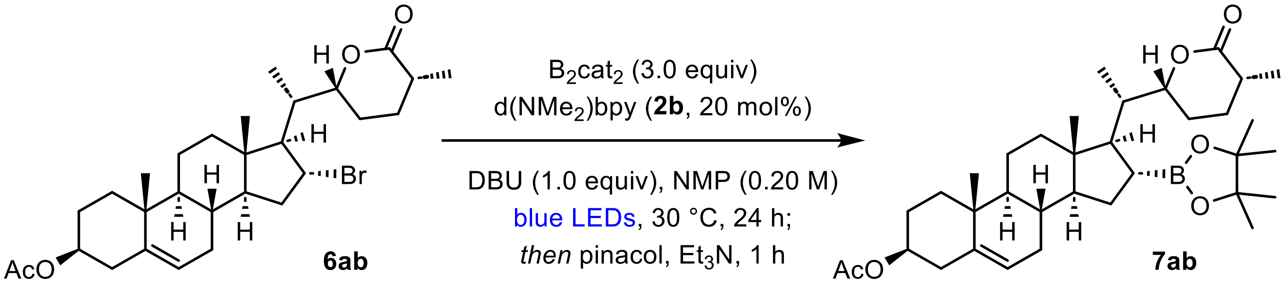

Following **GP2**, using **6ab** (107 mg, 0.200 mmol), B_2cat_2 (143 mg, 0.600 mmol, 3.00 equiv), d(NMe$_2$)bpy catalyst **2b** (10 mg, 0.040 mmol, 20 mol%) and DBU (30 µL, 0.20 mmol, 1.0 equiv) in NMP (1.0 mL). Purification by flash column chromatography (Biotage, Sfär silica, 10 g, 100:0 to 70:30 petroleum ether/EtOAc) gave **7ab** (65 mg, 56%) as a white solid, and unreacted starting material **6ab** (26 mg, 24%). The d.r. of **7ab** was determined to be >97:3 by ^{1}H NMR analysis after stereospecific oxidation of the boronic ester group to the corresponding alcohol.

NMR Spectroscopy (*see spectra*):

^{1}H NMR (500 MHz, $CDCl_3$): δ_H 5.35 (d, *J* = 5.0 Hz, 1H), 4.62 – 4.54 (m, 1H), 4.28 (dt, *J* = 11.8, 3.3 Hz, 1H), 2.61 – 2.53 (m, 1H), 2.34 – 2.26 (m, 2H), 2.02 (s, 3H), 2.01 – 1.80 (m, 7H), 1.70 – 1.58 (m, 2H), 1.57 – 1.35 (m, 8H), 1.31 – 1.27 (m, 1H), 1.24 (s, 6H), 1.22 (s, 6H), 1.20 (d, *J* = 6.8 Hz, 3H), 1.15 – 1.07 (m, 2H), 1.04 – 0.98 (m, 1H), 1.00 (s, 3H), 0.97 (d, *J* = 6.7 Hz, 3H), 0.96 – 0.92 (m, 1H) 0.73 (s, 3H);

^{13}C NMR (126 MHz, $CDCl_3$): δ_C 176.7, 170.7, 139.9, 122.5, 83.1, 81.0, 74.1, 56.1, 53.4, 49.8, 43.9, 40.5, 39.7, 38.2, 37.2, 36.7, 33.2, 32.0, 31.8, 29.0, 27.9, 25.8, 25.1, 24.7, 23.4 (br), 21.6, 21.2, 19.4, 19.3, 16.5, 13.5, 12.0 (the carbon attached to boron is broad due to quadrupolar relaxation).

IR (film): ν_{max} 2971, 2937, 2873, 2853, 1734, 1374, 1257, 1242, 950.

HRMS (ESI$^+$): calculated for $C_{35}H_{55}BO_6Na$ [M + Na]$^+$ 605.3990, found 605.3967.

Determination of the d.r. of **7ab**:

The d.r. of boronic ester **7ab** could not be determined by ^{1}H NMR analysis due to overlapping signals. Therefore, a small amount of **7ab** was oxidized to alcohol **S1ab** and subsequent ^{1}H NMR analysis indicated a d.r. of >97:3.

(3*S*,8*S*,9*S*,10*R*,13*S*,14*S*,16*R*,17*R*)-16-Hydroxy-10,13-dimethyl-17-((*S*)-1-((2*R*,5*R*)-5-methyl-6-oxotetrahydro-2H-pyran-2-yl)ethyl)-2,3,4,7,8,9,10,11,12,13,14,15,16,17-tetradecahydro-1*H*-cyclopenta[*a*]phenanthren-3-yl acetate (S1ab)

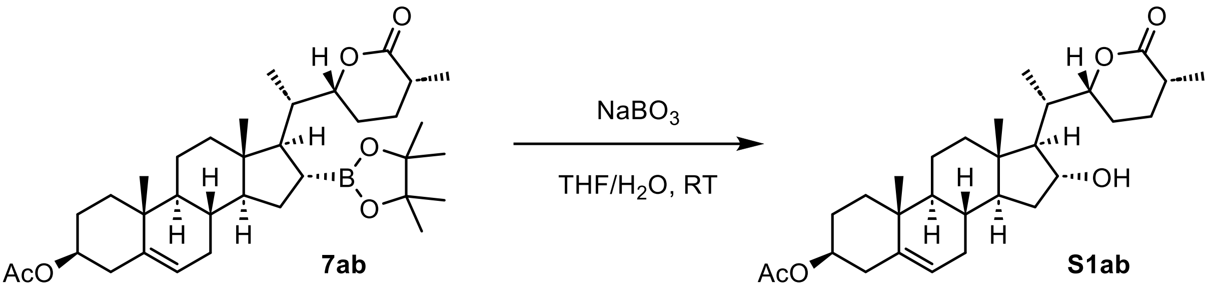

To a solution of **7ab** (12 mg, 20 µmol) in THF (0.8 mL) and water (0.4 mL) at RT was added sodium perborate tetrahydrate (31 mg, 0.20 mmol, 10 equiv). The reaction was stirred at RT for 12 h before being quenched with 10% aqueous $Na_2S_2O_3$ (1 mL), diluted with water (5 mL), and extracted with EtOAc (2 × 10 mL). The combined organic extracts were washed with brine (5 mL), dried over $MgSO_4$, filtered, and concentrated under reduced pressure. Purification by flash column chromatography (Biotage, Sfär silica, 5 g, 100:0 to 50:50 petroleum ether/EtOAc) gave **S1ab** (8 mg, 85%) as a white solid. ^{1}H NMR analysis showed the d.r. of **S1ab** to be >97:3.

NMR Spectroscopy (*see spectra*):

^{1}H NMR (500 MHz, $CDCl_3$): δ_H 5.36 (d, *J* = 5.0 Hz, 1H), 4.65 (dt, *J* = 11.5, 3.4 Hz, 1H), 4.60 (dt, *J* = 11.9, 6.1 Hz, 1H), 4.10 (t, *J* = 7.1 Hz, 1H), 2.63 (h, *J* = 7.2 Hz, 1H), 2.35 – 2.26 (m, 2H), 2.16 – 2.04 (m, 2H), 2.03 (s, 3H), 2.01 – 1.91 (m, 3H), 1.89 – 1.82 (m, 2H), 1.72 (td, *J* = 13.1, 8.1 Hz, 1H), 1.66 – 1.37 (m, 9H), 1.32 – 1.26 (m, 1H), 1.21 (d, *J* = 6.8 Hz, 3H), 1.17 – 1.09 (m, 2H), 1.02 – 0.98 (m, 1H), 1.01 (s, 3H), 0.99 (d, *J* = 6.6 Hz, 3H), 0.73 (s, 3H);

^{13}C NMR (126 MHz, $CDCl_3$): δ_C 177.2, 170.7, 140.0, 122.3, 80.8, 74.0, 61.8, 53.8, 50.0, 44.4, 39.8, 38.2, 38.1, 37.9, 37.0, 36.7, 33.4, 31.9, 31.4, 27.9, 25.9, 21.6, 20.8, 19.9, 19.4, 16.5, 13.3, 13.3.

IR (film): ν_{max} 3519, 3006, 2986, 2970, 2935, 2902, 2883, 2853, 2822, 1728, 1712, 1275, 1260, 1242, 1038, 1004, 764, 750.

HRMS (ESI$^+$): calculated for $C_{29}H_{44}O_5Na$ $[M + Na]^+$ 495.3081, found 495.3080.

(*S*)-1-((3*S*,5*S*,8*R*,9*S*,10*S*,13*S*,14*S*,16*R*,17*S*)-3-Acetoxy-10,13-dimethyl-16-(4,4,5,5-tetramethyl-1,3,2-dioxaborolan-2-yl)hexadecahydro-1*H*-cyclopenta[*a*]phenanthren-17-yl)ethyl acetate (7ac)

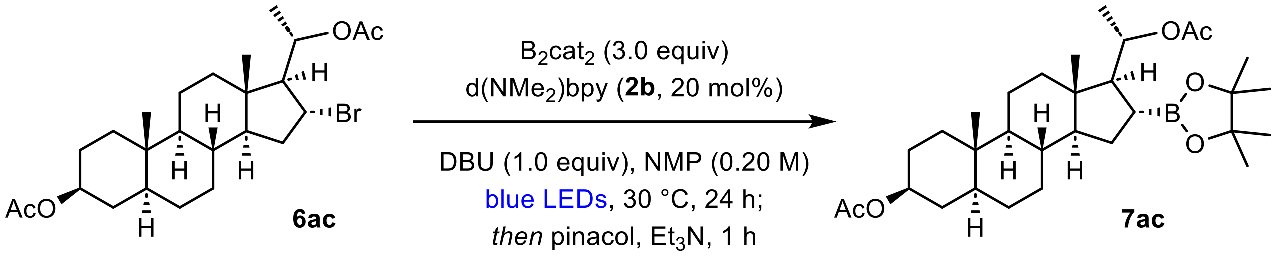

Following **GP2**, using **6ac** (97 mg, 0.20 mmol), B_2cat_2 (143 mg, 0.600 mmol, 3.00 equiv), d(NMe_2)bpy catalyst **2b** (10 mg, 0.040 mmol, 20 mol%) and DBU (30 µL, 0.20 mmol, 1.0 equiv) in NMP (1.0 mL). Purification by flash column chromatography (Biotage, Sfär silica, 10 g, 100:0 to 80:20 petroleum ether/EtOAc) gave **7ac** (59 mg, 56%) as a white solid, and unreacted starting material **6ac** (37 mg, 38%). The d.r. of **7ac** was determined to be >97:3 by ^{1}H NMR analysis after stereospecific oxidation of the boronic ester group and acetyl protection of the resulting alcohol.

NMR Spectroscopy (*see spectra*):

^{1}H NMR (500 MHz, $CDCl_3$): δ_H 4.83 (dq, *J* = 8.8, 6.2 Hz, 1H), 4.66 (tt, *J* = 10.9, 4.9 Hz, 1H), 2.00 (s, 3H), 1.99 (s, 3H), 1.82 – 1.74 (m, 2H), 1.72 – 1.65 (m, 2H), 1.62 (dd, *J* = 12.9, 3.4 Hz, 1H), 1.60 – 1.54 (m, 1H), 1.52 – 1.42 (m, 3H), 1.37 – 1.27 (m, 3H), 1.25 – 1.21 (m, 2H), 1.23 (s, 12H), 1.19 (d, *J* = 6.1 Hz, 4H), 1.16 – 1.08 (m, 2H), 0.99 (td, *J* = 13.9, 4.1 Hz, 1H), 0.94 – 0.87 (m, 1H), 0.86 – 0.81 (m, 1H), 0.79 (s, 3H), 0.71 – 0.66 (m, 1H), 0.65 (s, 3H);

^{13}C NMR (126 MHz, $CDCl_3$): δ_C 170.8, 170.7, 82.8, 73.8, 73.3, 58.0, 55.9, 54.0, 44.7, 42.8, 38.9, 36.9, 35.6, 35.3, 34.1, 31.9, 28.6, 27.7, 27.6, 25.2, 24.5, 21.7, 21.6, 21.1, 20.9, 13.0, 12.3 (the carbon attached to boron was not observed due to quadrupolar relaxation).

IR (film): ν_{max} 2975, 2928, 2869, 2854, 1734, 1376, 1239, 1146, 1027, 750.

HRMS (ESI$^+$): calculated for $C_{31}H_{51}BO_6Na$ [M + Na]$^+$ 553.3676, found 553.3665.

<u>Determination of the d.r. of **7ac**:</u>

The d.r. of boronic ester **7ac** could not be determined by ^{1}H NMR analysis due to overlapping signals. Therefore, a small amount of **7ac** was oxidized to an alcohol and subsequently acylated to generated acetate **S1ac′**. ^{1}H NMR analysis of **S1ac′** indicated a d.r. of >97:3.

(3*S*,5*S*,8*R*,9*S*,10*S*,13*S*,14*S*,16*R*,17*S*)-17-((*S*)-1-Acetoxyethyl)-10,13-dimethylhexadecahydro-1*H*-cyclopenta[*a*]phenanthrene-3,16-diyl diacetate (S1ac′)

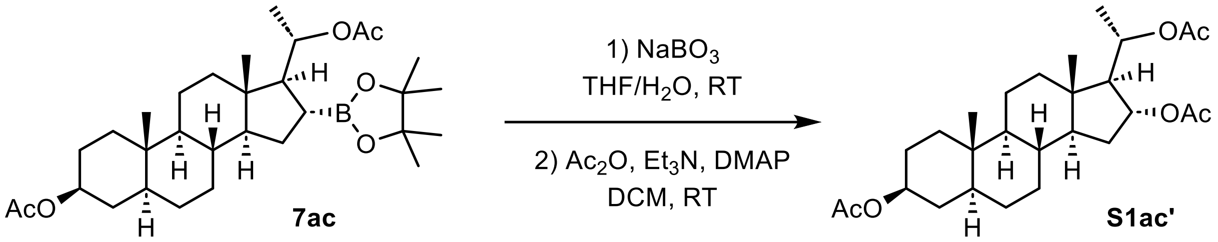

To a solution of **7ac** (11 mg, 20 µmol) in THF (0.8 mL) and water (0.4 mL) at RT was added sodium perborate tetrahydrate (31 mg, 0.20 mmol, 10 equiv). The reaction was stirred at RT for 14 h before being quenched with 10% aqueous $Na_2S_2O_3$ (1 mL), diluted with water (5 mL), and extracted with EtOAc (2 × 10 mL). The combined organic extracts were washed with brine (5 mL), dried over $MgSO_4$, filtered, and concentrated under reduced pressure.

To a solution of the crude alcohol (20 µmol), Et_3N (0.10 mL, 0.72 mmol, 36 equiv) and DMAP (0.5 mg, 4 µmol, 0.2 equiv) in DCM (3 mL) at RT was added Ac_2O (38 µL, 0.40 mmol, 20 equiv). The reaction was stirred at RT for 14 h before being quenched by the addition of 10% aqueous $NaHCO_3$ (5 mL), diluted with water (5 mL), and extracted with EtOAc (20 + 10 mL). The combined organic extracts were washed with brine (10 mL), dried over $MgSO_4$, filtered, and concentrated under reduced pressure. Purification by flash column chromatography (Biotage, Sfär silica, 5 g, 100:0 to 70:30 petroleum ether/EtOAc) gave **S1ac′** (9 mg, 97% over 2 steps) as a white solid. ^{1}H NMR analysis showed the d.r. of **S1ac′** to be >97:3.

NMR Spectroscopy (*see spectra*):

^{1}H NMR (500 MHz, $CDCl_3$): δ_H 5.19 (t, *J* = 7.3 Hz, 1H), 5.07 – 4.98 (m, 1H), 4.68 (tt, *J* = 11.0, 4.9 Hz, 1H), 2.03 (s, 3H), 2.01 (s, 3H), 1.95 (s, 3H), 1.86 – 1.77 (m, 2H), 1.75 – 1.68 (m, 3H), 1.63 – 1.57 (m, 2H), 1.56 – 1.44 (m, 3H), 1.40 – 1.31 (m, 3H), 1.27 – 1.23 (m, 3H), 1.23 (d, *J* = 6.3 Hz, 3H), 1.16 (tt, *J* = 12.2, 3.3 Hz, 1H), 1.02 (td, *J* = 13.6, 3.8 Hz, 1H), 0.97 – 0.88 (m, 1H)), 0.81 (s, 3H), 0.75 – 0.69 (m, 1H), 0.68 (s, 3H);

^{13}C NMR (126 MHz, $CDCl_3$): δ_C 170.8, 170.6, 170.5, 77.0, 73.7, 70.4, 61.6, 54.2, 53.5, 44.8, 42.9, 39.0, 36.8, 35.6, 34.6, 34.4, 34.1, 31.9, 28.5, 27.6, 21.6, 21.5, 21.5, 20.8, 20.7, 14.0, 12.3.

IR (film): ν_{max} 2979, 2933, 2850, 1731, 1445, 1371, 1275, 1235, 1029, 764, 750.

HRMS (ESI$^+$): calculated for $C_{27}H_{42}O_6Na$ [M + Na]$^+$ 485.2874, found 485.2894.

(3*S*,3a*S*,4*R*,5a*S*,5b*R*,7a*S*,9*S*,11a*S*,11b*S*,13a*R*)-9-((*tert*-Butyldiphenylsilyl)oxy)-3,11a-dimethyl-4-(4,4,5,5-tetramethyl-1,3,2-dioxaborolan-2-yl)hexadecahydro-1*H*,3*H*-naphtho[2',1':4,5]indeno[1,7a-*c*]furan-1-one (7ad)

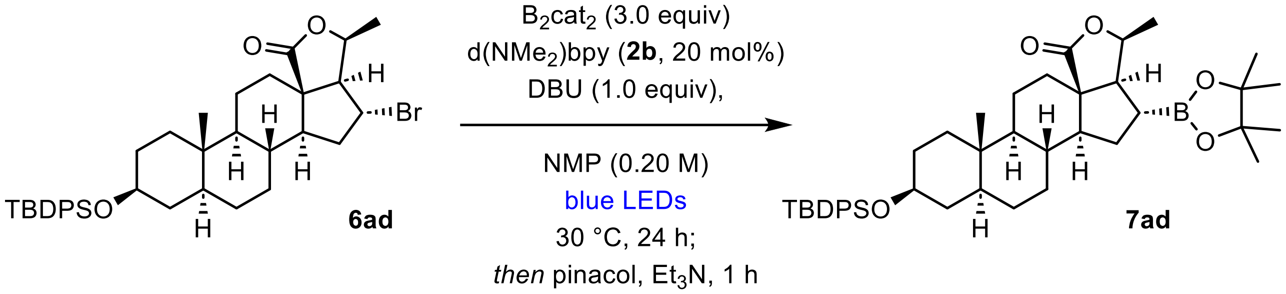

Following **GP2**, using **6ad** (130 mg, 0.200 mmol), B_2cat_2 (143 mg, 0.600 mmol, 3.00 equiv), d(NMe$_2$)bpy catalyst **2b** (10 mg, 0.040 mmol, 20 mol%) and DBU (30 μL, 0.20 mmol, 1.0 equiv) in NMP (1.0 mL). Purification by flash column chromatography (Biotage, Sfär silica, 10 g, 100:0 to 93:7 petroleum ether/EtOAc) gave **7ad** (126 mg, 90%) as a white foam. The d.r. of **7ad** was determined to be 97:3 by ^{1}H NMR analysis after stereospecific oxidation of the boronic ester group to the corresponding alcohol.

NMR Spectroscopy (*see spectra*):

^{1}**H NMR** (500 MHz, $CDCl_3$): δ_H 7.68 (d, *J* = 6.9 Hz, 4H), 7.43 – 7.38 (m, 2H), 7.36 (t, *J* = 7.2 Hz, 4H), 4.63 (dt, *J* = 10.8, 5.4 Hz, 1H), 3.57 (h, *J* = 6.3 Hz, 1H), 2.26 – 2.18 (m, 2H), 2.04 (dt, *J* = 12.5, 3.4 Hz, 1H), 1.79 (dd, *J* = 12.5, 6.3 Hz, 1H), 1.69 (dd, *J* = 12.8, 3.5 Hz, 1H), 1.65 – 1.51 (m, 5H), 1.46 – 1.40 (m, 2H), 1.40 – 1.33 (m, 2H), 1.31 (d, *J* = 6.5 Hz, 3H), 1.28 – 1.23 (m, 2H), 1.23 – 1.21 (m, 1H), 1.21 (s, 6H), 1.20 (s, 6H), 1.16 – 1.10 (m, 1H), 1.04 (s, 9H), 0.93 – 0.87 (m, 1H), 0.85 (s, 3H), 0.82 – 0.70 (m, 2H), 0.56 (td, *J* = 9.8, 6.8 Hz, 1H);

^{13}**C NMR** (126 MHz, $CDCl_3$): δ_C 179.4, 135.9, 135.1, 135.1, 129.5, 127.5, 127.5, 83.5, 75.7, 73.0, 57.0, 55.1, 54.0, 52.9, 45.0, 38.5, 37.4, 35.8, 34.4, 32.5, 31.8, 31.5, 29.2, 28.6, 27.1, 25.2, 24.9, 24.7, 20.8, 19.3, 18.0 (br), 16.3, 12.6 (the carbon attached to boron is broad due to quadrupolar relaxation).

IR (film): ν_{max} 3069, 3049, 2975, 2930, 2853, 1761, 1372, 1144, 1111, 1081, 1065, 702.

HRMS (ESI$^+$): calculated for $C_{43}H_{61}BO_5Si$ $[M + H]^+$ 719.4281, found 719.4261.

Determination of the d.r. of **7ad**:

The d.r. of boronic ester **7ad** could not be determined by ^{1}H NMR analysis due to overlapping signals. Therefore, a small amount of **7ad** was oxidized to the corresponding alcohol **S1ad** and subsequent ^{1}H NMR analysis indicated a d.r. of 97:3.

(3*S*,3a*R*,4*R*,5a*S*,5b*R*,7a*S*,9*S*,11a*S*,11b*S*,13a*R*)-9-((*tert*-Butyldiphenylsilyl)oxy)-4-hydroxy-3,11a-dimethylhexadecahydro-1*H*,3*H*-naphtho[2',1':4,5]indeno[1,7a-*c*]furan-1-one (S1ad)

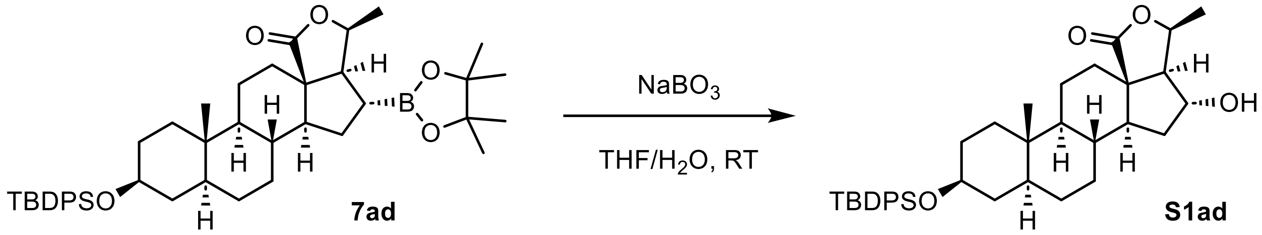

To a solution of **7ad** (11 mg, 15 µmol) in THF (0.8 mL) and water (0.4 mL) at RT was added sodium perborate tetrahydrate (23 mg, 0.15 mmol, 10 equiv). The reaction was stirred at RT for 16 h before being quenched with 10% aqueous $Na_2S_2O_3$ (1 mL), diluted with water (5 mL), and extracted with EtOAc (2 × 10 mL). The combined organic extracts were washed with brine (5 mL), dried over $MgSO_4$, filtered, and concentrated under reduced pressure to give crude **S1ad** (8 mg, 91%) as a white foam. ^1H NMR analysis showed the d.r. of **S1ad** to be 97:3 (Figure S3).

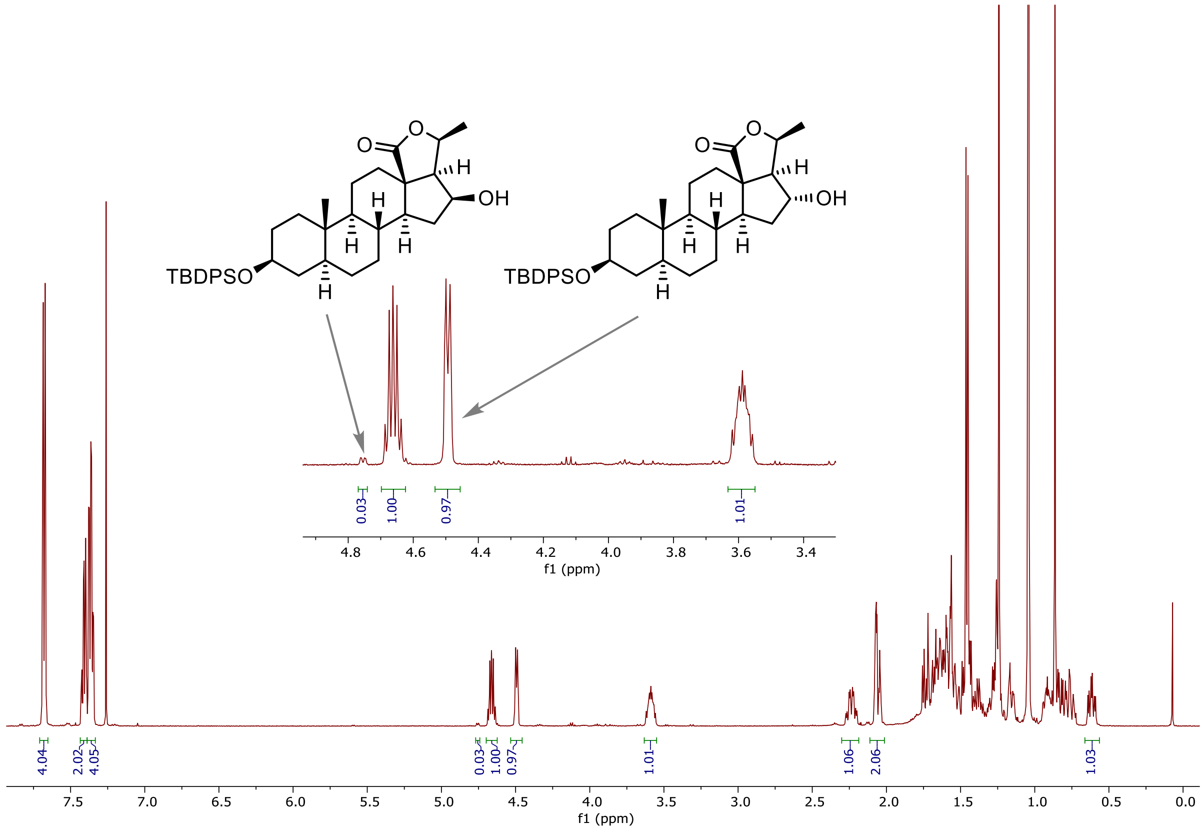

Figure S3. Determination of the d.r. of **7ad**

(2*R*,2a*R*,2a^1*S*,3*R*,6a*S*,6b*S*,9*S*,10a*S*,12a*R*,12b*S*)-5-((*R*)-3-Bromo-2-methylpropyl)-9-hydroxy-2a^1,3,6b-trimethyl-2-(4,4,5,5-tetramethyl-1,3,2-dioxaborolan-2-yl)-1,2,2a,3,6,6a,6b,7,8,9,10,10a,11,12,12a,12b-hexadecahydrocyclopenta[*ij*]tetraphen-4(2a^1*H*)-one (7ae)

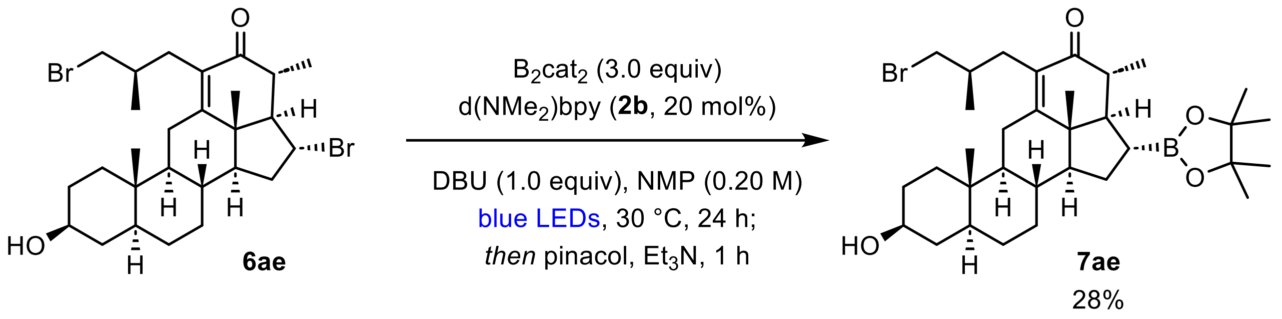

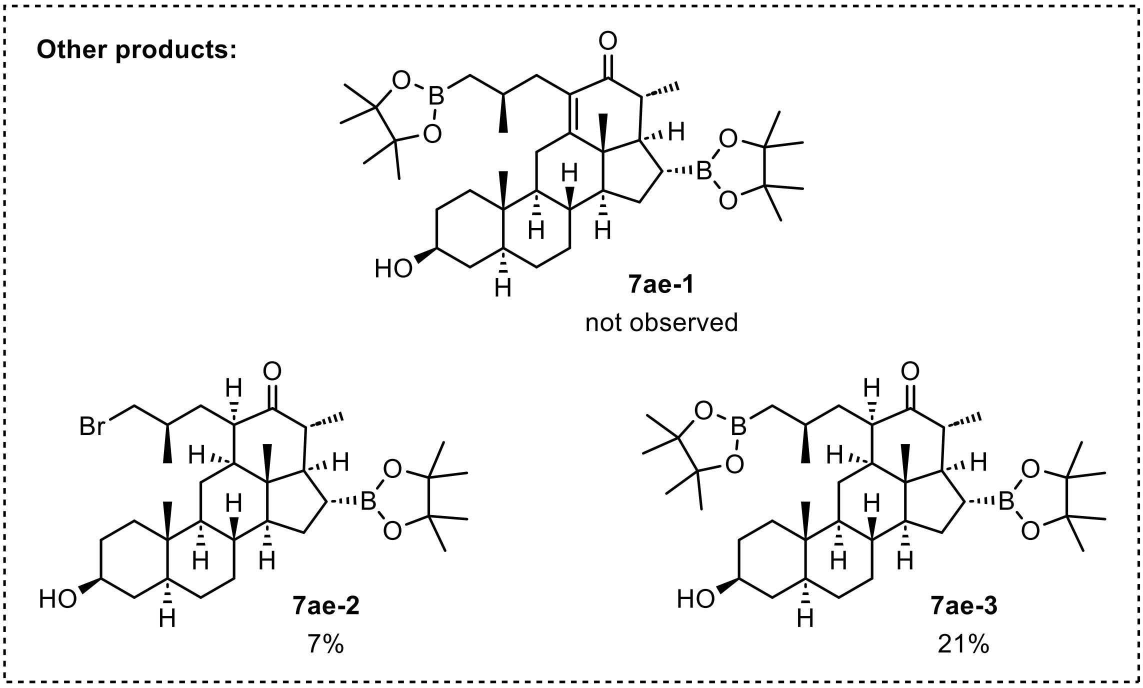

Following **GP2**, using **6ae** (90 mg, 0.16 mmol), B_2cat_2 (114 mg, 0.48 mmol, 3.00 equiv), d(NMe_2)bpy catalyst **2b** (8 mg, 0.03 mmol, 0.2 equiv) and DBU (24 µL, 0.16 mmol, 1.0 equiv) in NMP (0.80 mL). Purification by flash column chromatography (Biotage, Sfär silica, 10 g, 100:0 to 80:20 petroleum ether/acetone, then again with 100:0 to 25:75 petroleum ether/Et_2O) gave **7ae** (27 mg, 28%), **7ae-2** (7 mg, 7%) and **7ae-3** (22 mg, 21%) as colourless oils. The d.r. of **7ae** was determined to be 93:7 by ^{1}H NMR analysis after stereospecific oxidation of the boronic ester group and acetyl protection of the resulting alcohol.

NMR Spectroscopy (*see spectra*):

^{1}H NMR (500 MHz, $CDCl_3$): δ_H 3.61 (dt, *J* = 11.0, 5.9 Hz, 1H), 3.47 (dd, *J* = 10.6, 4.7 Hz, 1H), 3.38 (dd, *J* = 10.6, 5.9 Hz, 1H), 2.69 (dd, *J* = 16.1, 4.6 Hz, 1H), 2.36 (dd, *J* = 13.2, 6.2 Hz, 1H), 2.26 (dq, *J* = 13.5, 6.7 Hz, 1H), 2.12 (dd, *J* = 13.3, 7.8 Hz, 1H), 2.09 – 2.02 (m, 1H), 1.94 – 1.81 (m, 2H), 1.79 – 1.72 (m, 2H), 1.70 – 1.50 (m, 5H), 1.45 – 1.40 (m, 1H), 1.36 – 1.27 (m, 5H), 1.23 (s, 12H), 1.17 – 1.12 (m, 1H), 1.10 (d, *J* = 6.7 Hz, 3H), 1.05 – 0.95 (m, 4H), 0.92 (d, *J* = 6.7 Hz, 3H), 0.89 (s, 3H), 0.85 (s, 3H);

^{13}C NMR (126 MHz, $CDCl_3$): δ_C 202.3, 166.2, 130.2, 83.3, 71.1, 55.7, 54.3, 54.0, 51.7, 45.1, 44.8, 42.8,

38.3, 37.0, 36.3, 35.5, 34.6, 31.6, 31.6, 29.4, 28.5, 27.2, 26.3, 24.9, 24.8, 24.8, 17.7, 14.6, 14.3, 12.5 (the carbon attached to boron was not observed due to quadrupolar relaxation).

IR (film): ν_{max} 3448(brs), 2924, 2853, 1697, 1659, 1454, 1373, 1319, 1144, 857, 749.

HRMS (APCI$^+$): calculated for $C_{33}H_{52}O_4B$ [M–Br]$^+$ 523.3953, found 523.3938.

Determination of the d.r. of **7ae**:

The d.r. of boronic ester **7ae** could not be determined by ^{1}H NMR analysis due to overlapping signals. Therefore, a small amount of **7ae** was oxidized to an alcohol and subsequently acylated to generated acetate **S1ae′**. ^{1}H NMR analysis of **S1ae′** indicated a d.r. of 93:7.

(2*R*,2a*R*,2a^1*S*,3*R*,6a*S*,6b*S*,9*S*,10a*S*,12a*R*,12b*S*)-5-((*R*)-3-Bromo-2-methylpropyl)-2a^1,3,6b-trimethyl-4-oxo-1,2,2a,2a^1,3,4,6,6a,6b,7,8,9,10,10a,11,12,12a,12b-octadecahydrocyclopenta[*ij*]tetraphene-2,9-diyl diacetate (S1ae′)

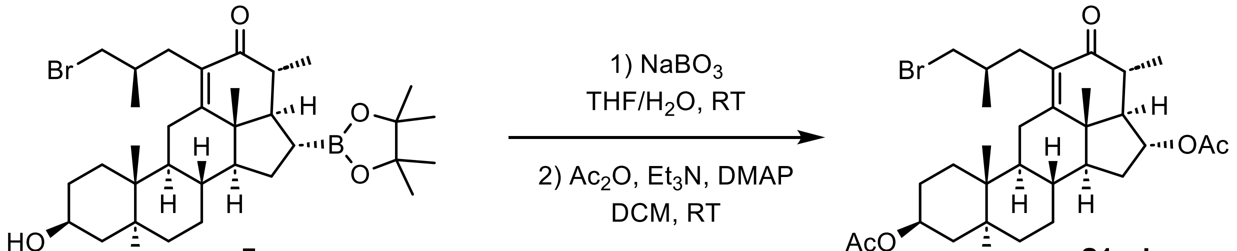

7ae **S1ae'**

To a solution of **7ae** (6 mg, 10 µmol) in THF (0.8 mL) and water (0.4 mL) at RT was added sodium perborate tetrahydrate (31 mg, 0.20 mmol, 20 equiv). The reaction was stirred at RT for 16 h before being quenched with 10% aqueous $Na_2S_2O_3$ (1 mL), diluted with water (5 mL), and extracted with EtOAc (2 × 10 mL). The combined organic extracts were washed with brine (5 mL), dried over $MgSO_4$, filtered, and concentrated under reduced pressure.

To a solution of the crude alcohol (10 µmol), Et_3N (0.10 mL, 0.72 mmol, 72 equiv) and DMAP (0.5 mg, 4 µmol, 0.4 equiv) in DCM (3 mL) at RT was added Ac_2O (38 µL, 0.40 mmol, 40 equiv). The reaction was stirred at RT for 14 h before being quenched by the addition of 10% aqueous $NaHCO_3$ (5 mL), diluted with water (5 mL), and extracted with EtOAc (20 + 10 mL). The combined organic extracts were washed with brine (10 mL), dried over $MgSO_4$, filtered, and concentrated under reduced pressure. Purification by flash column chromatography (Biotage, Sfär silica, 5 g, 100:0 to 70:30 petroleum ether/EtOAc) gave **S1ae′** (5 mg, 87% over 2 steps) as a white solid. ^{1}H NMR showed the dr of **S1ae′** to be 93:7 (Figure S4).

NMR Spectroscopy (*see spectra*):

^{1}H NMR (500 MHz, $CDCl_3$): δ_H 5.17 (td, *J* = 9.1, 2.4 Hz, 1H), 4.69 (tt, *J* = 11.0, 4.8 Hz, 1H), 3.47 (dd, *J* = 10.5, 4.8 Hz, 1H), 3.39 (dd, *J* = 10.6, 5.6 Hz, 1H), 2.71 (dd, *J* = 16.2, 4.6 Hz, 1H), 2.47 – 2.38 (m, 2H), 2.18 – 2.05 (m, 3H), 2.04 (s, 3H), 2.03 (s, 3H), 1.95 – 1.75 (m, 4H), 1.74 – 1.68 (m, 1H), 1.68 – 1.47 (m, 4H),

1.43 – 1.18 (m, 4H), 1.12 (d, *J* = 6.6 Hz, 3H), 1.08 (dd, *J* = 13.9, 3.9 Hz, 1H), 1.05 – 0.95 (m, 3H), 0.93 (s, 3H), 0.92 (d, *J* = 6.6 Hz, 3H), 0.92 (s, 3H);

^{13}C NMR (126 MHz, $CDCl_3$): δ_C 201.2, 171.1, 170.8, 164.6, 130.7, 76.2, 73.4, 56.1, 55.6, 52.7, 51.5, 44.9, 43.5, 41.5, 36.6, 36.3, 35.4, 34.1, 34.0, 33.7, 31.5, 29.3, 28.2, 27.5, 25.7, 21.6, 21.4, 17.6, 15.7, 13.4, 12.4.

IR (film): ν_{max} 2967, 2929, 2854, 1732, 1661, 1445, 1375, 1241, 1038, 764, 750.

MS (APCI$^+$): m/z 497.3 [M–Br]$^+$.

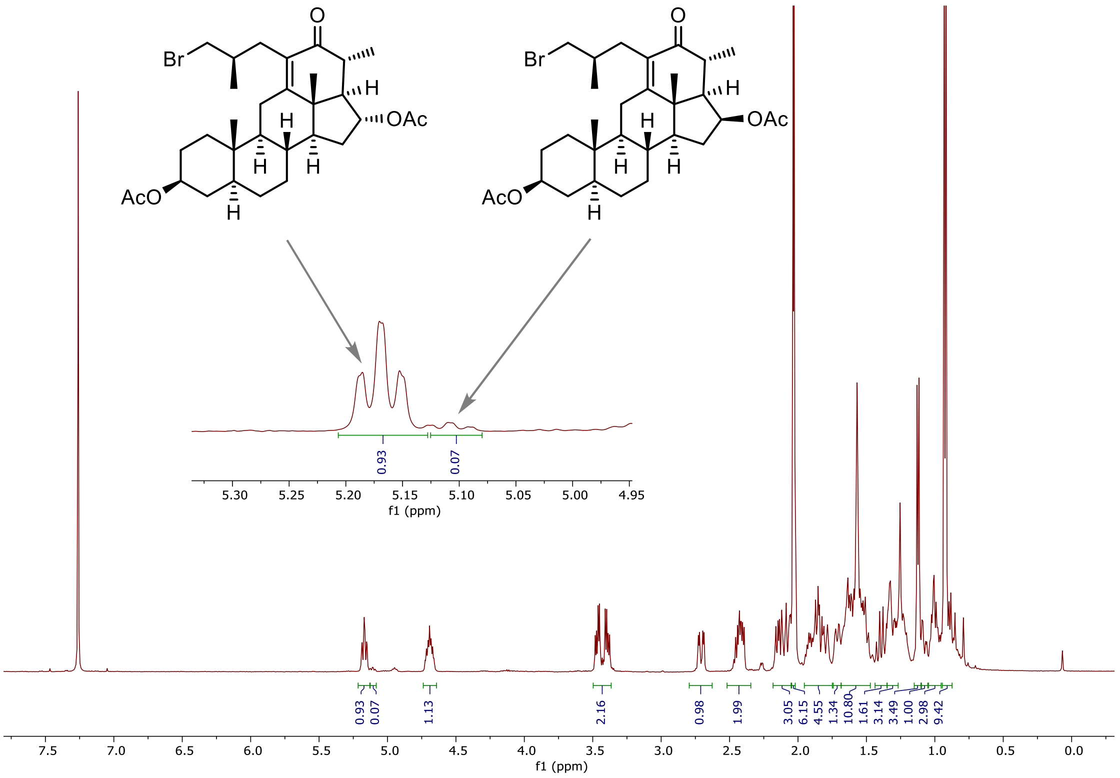

Figure S4. Determination of the d.r. of **7ae**

(2*R*,2a*R*,2a^1*R*,3*R*,5*R*,5a*S*,6a*S*,6b*S*,9*S*,10a*S*,12a*R*,12b*S*)-5-((*R*)-3-Bromo-2-methylpropyl)-9-hydroxy-2a^1,3,6b-trimethyl-2-(4,4,5,5-tetramethyl-1,3,2-dioxaborolan-2-yl)octadecahydrocyclopenta[*ij*]tetraphen-4(2a^1*H*)-one (7ae-2)

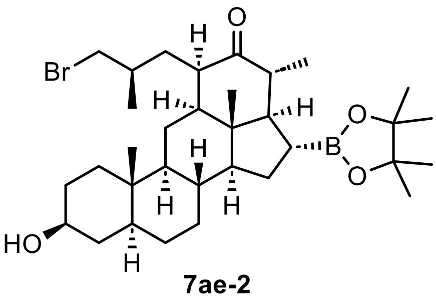

7ae-2

NMR Spectroscopy (*see spectra*):

^{1}H NMR (500 MHz, $CDCl_3$): δ_H 3.59 (tt, *J* = 9.8, 3.8 Hz, 1H), 3.44 (ddd, *J* = 10.9, 4.5, 1.5 Hz, 1H), 3.39

(ddd, *J* = 11.1, 5.6, 1.4 Hz, 1H), 2.45 – 2.36 (m, 1H), 2.18 (t, *J* = 9.3 Hz, 1H), 1.97 – 1.90 (m, 1H), 1.85 – 1.79 (m, 1H), 1.76 – 1.50 (m, 9H), 1.46 – 1.24 (m, 7H), 1.22 (s, 12H), 1.14 – 1.07 (m, 3H), 1.06 – 1.02 (m, 1H), 1.01 (d, *J* = 6.6 Hz, 3H), 0.98 (d, *J* = 6.5 Hz, 3H), 0.94 – 0.88 (m, 2H), 0.85 (s, 3H), 0.81 (s, 3H), 0.75 (td, *J* = 9.7, 8.6, 3.2 Hz, 1H);

^{13}C NMR (126 MHz, $CDCl_3$): δ_C 214.2, 83.2, 71.3, 58.5, 55.4, 54.8, 51.2, 50.4, 46.9, 45.2, 43.9, 38.3, 37.3, 36.2, 35.7, 34.3, 32.1, 31.7, 30.6, 29.4, 28.8, 25.0, 24.9, 24.8, 19.2, 13.8, 12.6, 10.5 (the carbon attached to boron was not observed due to quadrupolar relaxation).

IR (film): ν_{max} 3452(brs), 2976, 2928, 2856, 1698, 1449, 1372, 1318, 1143, 1039, 856, 735.

HRMS (APCI$^+$): calculated for $C_{33}H_{54}O_4B$ [M–Br]$^+$ 525.4110, found 525.4090.

(2*R*,2a*R*,2a^1*R*,3R,5*R*,5a*S*,6a*S*,6b*S*,9*S*,10a*S*,12a*R*,12b*S*)-9-Hydroxy-2a^1,3,6b-trimethyl-5-((*S*)-2-methyl-3-(4,4,5,5-tetramethyl-1,3,2-dioxaborolan-2-yl)propyl)-2-(4,4,5,5-tetramethyl-1,3,2-dioxaborolan-2-yl)octadecahydrocyclopenta[*ij*]tetraphen-4(2a^1*H*)-one (7ae-3)

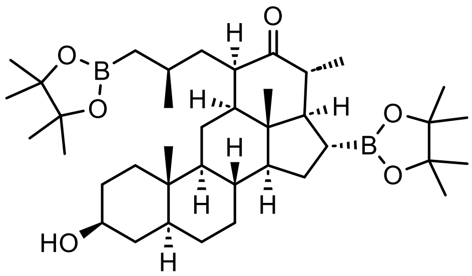

7ae-3

NMR Spectroscopy (*see spectra*):

^{1}H NMR (500 MHz, $CDCl_3$): δ_H 3.59 (dt, *J* = 11.1, 5.9 Hz, 1H), 2.39 (dq, *J* = 12.8, 6.3 Hz, 1H), 2.18 (t, *J* = 8.4 Hz, 1H), 1.81 (d, *J* = 12.4 Hz, 1H), 1.77 – 1.61 (m, 8H), 1.60 – 1.54 (m, 2H), 1.50 (td, *J* = 11.1, 3.5 Hz, 1H), 1.46 – 1.36 (m, 1H), 1.34 – 1.26 (m, 4H), 1.21 (s, 24H), 1.13 – 1.06 (m, 2H), 1.05 – 0.99 (m, 2H), 0.98 – 0.93 (m, 2H), 0.96 (d, *J* = 6.3 Hz, 3H), 0.91 (d, *J* = 6.2 Hz, 3H), 0.84 (s, 3H), 0.83 – 0.78 (m, 2H), 0.81 (s, 3H), 0.73 (td, *J* = 10.3, 3.8 Hz, 1H), 0.51 (dd, *J* = 15.3, 8.5 Hz, 1H);

^{13}C NMR (126 MHz, $CDCl_3$): δ_C 214.3, 83.2, 82.9, 71.3, 58.7, 55.4, 54.8, 50.5, 49.2, 46.9, 45.3, 44.0, 38.4, 37.5, 36.2, 36.1, 35.8, 32.2, 31.6, 29.5, 28.9, 28.7, 25.1, 25.0, 24.9, 24.9, 24.8, 23.4, 13.8, 12.6, 10.6 (the carbon attached to boron was not observed due to quadrupolar relaxation).

IR (film): ν_{max} 3450(brs), 2976, 2926, 2855, 1700, 1452, 1371, 1317, 1143, 1041, 968, 857, 737.

HRMS (ESI$^+$): calculated for $C_{39}H_{66}B_2O_6Na$ [M+Na]$^+$ 675.4951, found 675.4926.

2.6. Other Applications

2.6.1. Deoxygenative Borylation via Aryl Bromide Reduction

Under our previously reported catalyst-free photoinduced deoxygenative borylations of 2-iodophenyl thionocarbonates **S2**, the corresponding 2-bromophenyl thionocarbonate **14** failed to yield more than trace product (Scheme S1a).[21] Using the bipyridine-catalysed conditions, 2-bromophenyl thionocarbonate **14** was converted to boronic ester **7a** in 62% yield (Scheme S1b).

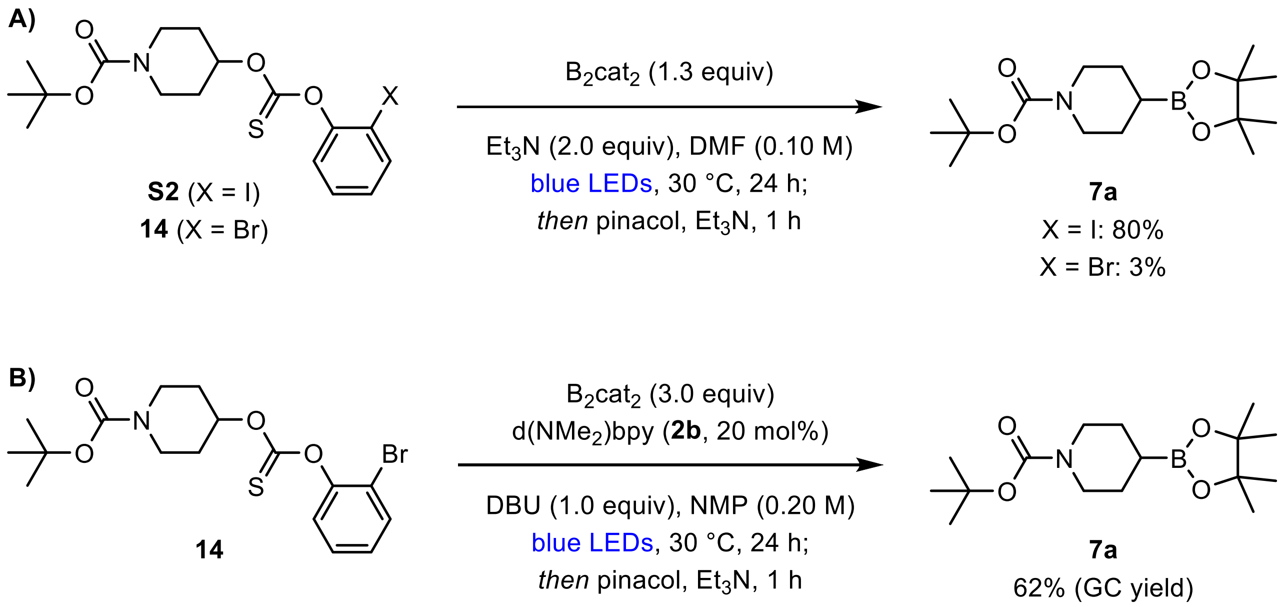

Scheme S1. Deoxygenative borylations

Conditions for the conversion of **14** to **7a**:

Following **GP2**, using **14** (42 mg, 0.10 mmol), B_2cat_2 (72 mg, 0.30 mmol, 3.0 equiv), $d(NMe_2)bpy$ catalyst **2b** (5 mg, 0.02 mmol, 0.2 equiv) and DBU (15 µL, 0.20 mmol, 1.0 equiv) in NMP (0.5 mL). The yield of **7a** was determined by GC analysis using 1,3,5-trimethoxybenzene as the internal standard to be 62%.

2.6.2. Borylation of Aryl Bromides

After demonstrating successful debrominative aryl radical formation in the reaction of 2-bromophenyl thionocarbonates **14**, we also applied modified conditions to the direct debrominative borylation of 4-bromotoluene (**S3**).

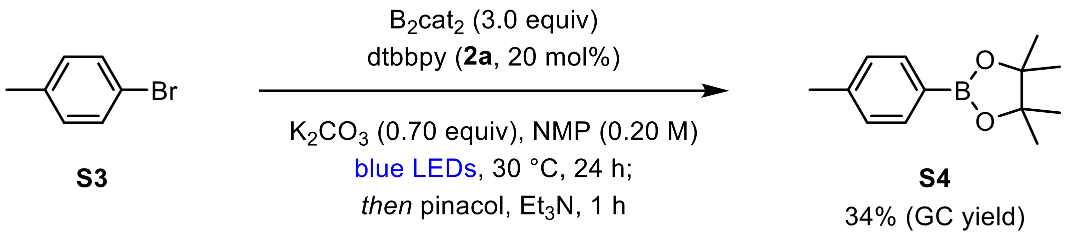

S3

S4
34% (GC yield)
[49% with DMF as solvent]

Following **GP2-modification 2**, using **S3** (34 mg, 0.20 mmol), B_2cat_2 (143 mg, 0.600 mmol, 3.00 equiv), dtbbpy (**2a**, 11 mg, 0.040 mmol, 20 mol%), and K_2CO_3 (19 mg, 0.14 mmol, 0.70 equiv). The yield of **S4** was determined by GC analysis using 1,3,5-trimethoxybenzene as the internal standard to be 34%.

Note: When the reaction was performed with DMF (1.0 mL) as the solvent instead of NMP, the yield of **S4** increased to 49% (GC yield).

2.6.3. Borylation of Alkyl Chlorides

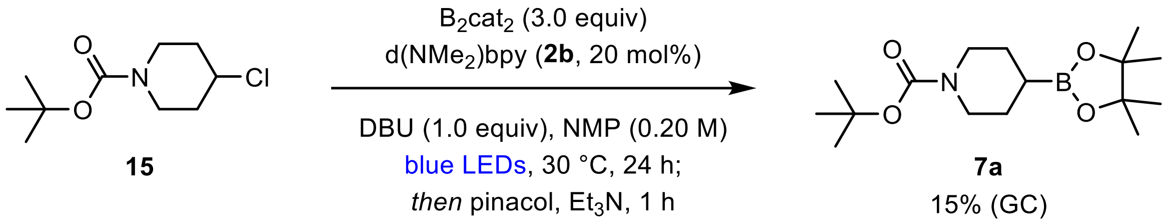

Following **GP2**, using *tert*-butyl 4-chloropiperidine-1-carboxylate (**15**, 44 mg, 0.20 mmol), B_2cat_2 (143 mg, 0.600 mmol, 3.00 equiv.), d(NMe_2)bpy catalyst **2b** (10 mg, 0.040 mmol, 20 mol%), and DBU (30 µL, 0.20 mmol, 1.0 equiv.). The yield was determined by GC analysis using 1,3,5-trimethoxybenzene as the internal standard to be 15%. Approximately 85% unreacted **15** remained.

2.6.4. Borylation of Aryl Chlorides

4-Chloroanisole:

1-Chloro-4-methoxybenzene (**16a**, 14 mg, 0.10 mmol), B_2cat_2 (4.8-71 mg, 0.020-0.30 mmol, 0.20-3.0 equiv.), B_2pin_2 (76-127 mg, 0.30-0.50 mmol, 3.0-5.0 equiv.), and d(Me_2N)bpy (**2b**, 4.8-12 mg, 0.020-0.050 mmol, 20-50 mol%) or 3,4,7,8-tetramethyl-1,10-phenanthroline (**2c**, 12 mg, 0.050 mmol, 50 mol%) were added into an oven-dried 7 mL vial containing a small magnetic stirrer bar. The vial was sealed with a septum and anhydrous DMA (0.5 mL, 0.20 M) was added. The headspace of the vial was purged with a gentle stream of N_2 for approximately 1 min before DBU (15 µL, 0.10 mmol, 1.0 equiv) was added. The vial was tightly sealed with parafilm, then placed in the photoreactor before stirring under irradiation for 24-72 h (see Figure S1 for experimental setup). The vial was removed from the photoreactor, a solution of pinacol (71 mg, 0.60 mmol, 6.0 equiv) in Et_3N (0.15 mL) was added to the reaction and it was stirred at RT for another 1 h. The yield was determined by GC analysis using mesitylene as the internal standard after calibration against a commercial sample of **17a**. The results are shown in Table S2.

Table S2. Different reaction conditions for the borylation of aryl chloride **16a**

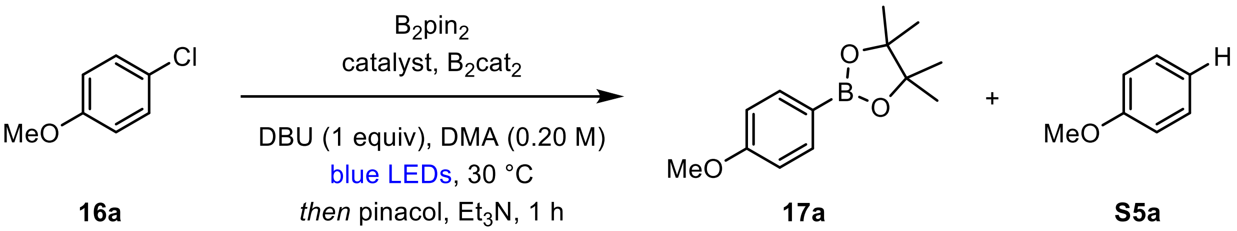

(0.2 mmol)

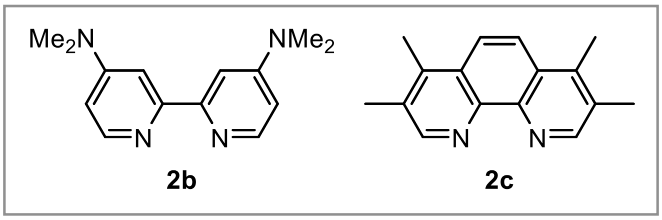

Entry	catalyst (mol%)	B_2cat_2 equiv.	B_2pin_2 equiv	time	**17a**	**S5a**	**16a**
1	**2b** (20)	3	0	24 h	14%	6%	78%
2	**2b** (20)	0.2	3	24 h	20%	8%	66%
3	**2b** (50)	0.5	3	72 h	32%	13%	55%
4	**2b** (50)	0.25	5	72 h	15%	4%	72%
5	**2c** (50)	0.25	5	72 h	35%	15%	58%
6[a]	**2c** (50)	0.25	5	72 h	36%[b]	ND	ND

Yields and conversions were determined by GC analysis using mesitylene as an internal standard. [a] The fan speed on the photoreactor was reduced from 4500 rpm to 2800 ppm, leading to a temperature increase to 35-40 °C. [b] Yield determined by ^1H NMR analysis. ND = not determined.

1-*tert*-Butyl-4-chlorobenzene:

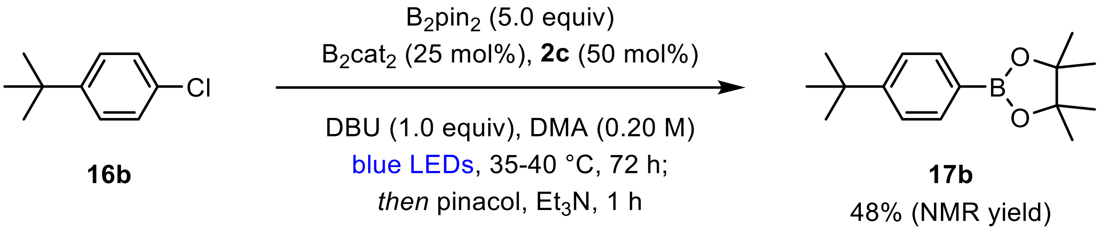

1-*tert*-Butyl-4-chlorobenzene (**16b**, 21 mg, 0.10 mmol), B_2cat_2 (5.9 mg, 0.025 mmol, 25 mol%), B_2pin_2 (127 mg, 0.500 mmol, 5.00 equiv.), and 3,4,7,8-tetramethyl-1,10-phenanthroline (**2c**, 12 mg, 0.050 mmol, 50 mol%) were added into an oven-dried 7 mL vial containing a small magnetic stirrer bar. The vial was sealed with a septum and anhydrous DMA (0.5 mL, 0.20 M) was added. The headspace of the vial was purged with a gentle stream of N_2 for approximately 1 min before DBU (15 µL, 0.10 mmol, 1.0 equiv) was added. The vial was tightly sealed with parafilm, then placed in the photoreactor before stirring under irradiation for 72 h (see Figure S1 for experimental setup, except with the fan speed set to 2800 rpm to maintain a higher temperature of 35-40 °C). The vial was removed from the photoreactor, a solution of pinacol (71 mg, 0.60 mmol, 6.0 equiv) in Et_3N (0.15 mL) was added to the reaction and it was stirred at RT for another 1 h. Water (5 mL) was added and the reaction mixture was extracted with EtOAc (15 + 10 mL). The combined organic extracts were washed with water (10 mL) and brine (15 mL), dried over $MgSO_4$, filtered, and concentrated under reduced pressure. The yield was determined by ^1H NMR analysis using mesitylene as the internal standard to be 48%.

2.6.5. Desulfonylation of Sulfonamides

Methyl 4-(1-tosyl-1H-indol-3-yl)butanoate (18)

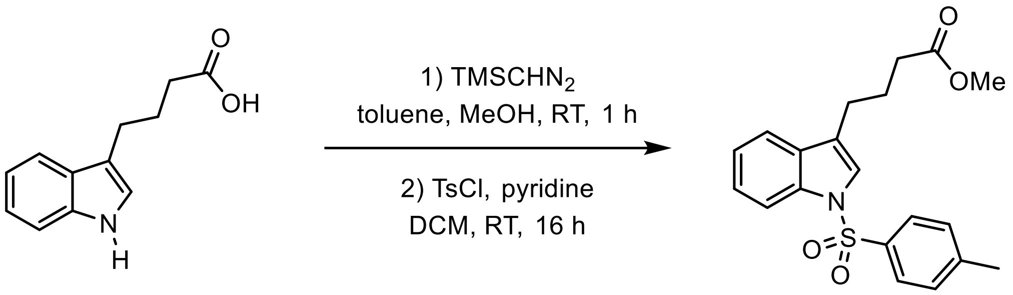

S6 **18**

To a solution of **S6** (305 mg, 1.50 mmol) in toluene (6 mL) and MeOH (2 mL) at RT, trimethylsilyldiazomethane (2 M in Et_2O, 1.0 mL, 2.0 mmol, 1.3 equiv) was added dropwise over 2 min. The reaction was stirred at RT for 1 h before concentrating under reduced pressure. The crude product was dried under high vacuum and used in the next step without further purification.

To a solution of the crude product and pyridine (0.50 mL) in DCM (6 mL) at RT, a solution of *p*-toluenesulfonyl chloride (0.38 g, 2.0 mmol, 1.3 equiv) in DCM (4 mL) was slowly added. The solution was stirred at RT for 16 h before being washed with aqueous 1 M aqueous HCl (10 mL) and extracted with EtOAc (30 + 15 mL). The combined organic extracts were washed with water (20 mL), brine (20 mL), dried over $MgSO_4$, filtered, and concentrated under reduced pressure. Purification by flash column chromatography (Biotage, Sfär silica, 25 g, 100:0 to 92:8 toluene/EtOAc) gave **18** (506 mg, 91%) as an off white solid.

NMR Spectroscopy (*see spectra*):

^{1}**H NMR** (500 MHz, $CDCl_3$): δ_H 7.98 (dt, *J* = 8.3, 1.0 Hz, 1H), 7.74 (d, *J* = 8.4 Hz, 2H), 7.48 (d, *J* = 7.8 Hz, 1H), 7.34 – 7.28 (m, 2H), 7.25 – 7.18 (m, 3H), 3.68 (s, 3H), 2.70 (t, *J* = 7.6 Hz, 2H), 2.35 (t, *J* = 7.4 Hz, 2H), 2.33 (s, 3H), 2.01 (p, *J* = 7.4 Hz, 2H);

^{13}**C NMR** (126 MHz, $CDCl_3$): δ_C 173.9, 144.9, 135.5, 135.4, 131.0, 129.9, 126.9, 124.8, 123.2, 123.1, 122.4, 119.6, 113.9, 51.7, 33.5, 24.4, 24.3, 21.7.

IR (film): ν_{max} 2951, 1725, 1596, 1446, 1358, 1169, 1118.

HRMS (ESI): calculated for $C_{20}H_{21}NO_4S$ $[M+Na]^+$ 394.1084, found 394.1097.

Methyl 4-(1H-indol-3-yl)butanoate (19)

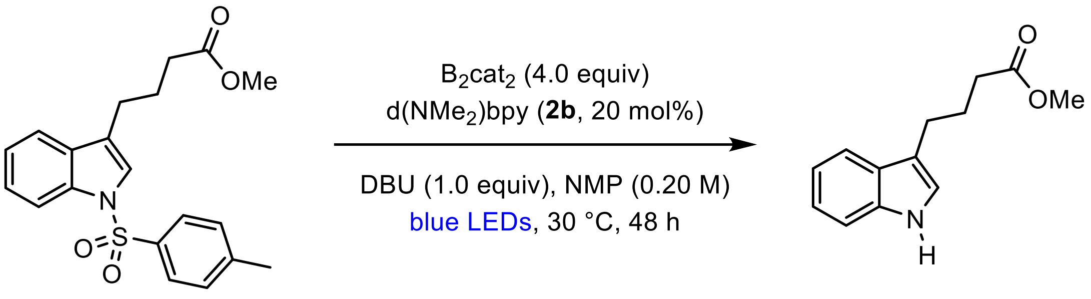

18 **19**

N-Tosyl indole **18** (37 mg, 0.10 mmol), B_2cat_2 (95 mg, 0.40 mmol, 4.0 equiv), and d(NMe_2)bpy catalyst **2b** (5 mg, 0.04 mmol, 0.2 equiv) were added into an oven-dried 7 mL vial containing a small magnetic stirrer bar. The vial was sealed with a septum and anhydrous NMP (1.0 mL, 0.10 M) was added. The headspace of the vial was purged with a gentle stream of N_2 for approximately 1 min before DBU (15 µL, 0.10 mmol, 1.0 equiv) was added. The vial was tightly sealed with parafilm, then placed in the photoreactor before stirring under irradiation for 48 h (see Figure S1 for experimental setup). Water (5 mL) was added and the reaction mixture was extracted with EtOAc (20 + 10 mL). The combined organic extracts were washed with brine (5 mL), dried over $MgSO_4$, filtered, and concentrated under reduced pressure. The crude product was purified by flash column chromatography (Biotage, Sfär silica, 10 g, toluene/EtOAc = 100:0 to 92:8) to give **19** (18 mg, 81% yield) as a white solid.

Note: When the reaction was performed with 3 equivalents of B_2cat_2, product **19** was formed in 64% yield after 48 h irradiation.

NMR Spectroscopy (*see spectra*):

^{1}H NMR (400 MHz, $CDCl_3$): δ_H 7.96 (brs, 1H), 7.62 (d, *J* = 7.8 Hz, 1H), 7.36 (d, *J* = 8.1 Hz, 1H), 7.20 (ddd, *J* = 8.2, 7.0, 1.2 Hz, 1H), 7.12 (ddd, *J* = 8.0, 7.0, 1.1 Hz, 1H), 6.99 (dt, *J* = 2.2, 1.0 Hz, 1H), 3.67 (s, 3H), 2.82 (td, *J* = 7.4, 0.9 Hz, 2H), 2.40 (t, *J* = 7.4 Hz, 2H), 2.07 (p, *J* = 7.5 Hz, 2H);

^{13}C NMR (101 MHz, $CDCl_3$): δ_C 174.3, 136.5, 127.6, 122.1, 121.6, 119.3, 119.0, 115.8, 111.2, 51.6, 33.8, 25.5, 24.6.

All recorded spectroscopic data matched those previously reported in the literature.[28]

3. MECHANISTIC STUDIES

3.1. NMR Studies

Initial investigations into studying the reaction of B_2cat_2 with dtbbpy (**2a**) by ^1H NMR were carried out in DMF-d_4; however, trace water impurities in this solvent complicated analysis due to considerable hydrolysis of B_2cat_2. Similarly, trace water impurities also complicated ^1H NMR spectra measured in CD_3CN. In addition, upon mixing B_2cat_2 and dtbbpy in CD_3CN, a deep red/black solid precipitated, which was tentatively assigned as a dtbbpy•B_2cat_2 complex.[29] To avoid these issues with water impurities and solubility, ^1H NMR investigations were carried out using THF-d_8 from sealed ampules and samples were prepared in a glovebox.

Sample preparation:

In a glovebox, to a 7.0 mL vial equipped was added 4,4'-di-*tert*-butyl-2,2'-bipyridine (**2a**, 6.4 mg, 0.024 mmol, 1.0 equiv), B_2cat_2 (5.7 mg, 0.024 mmol, 1.0 equiv), and 1,3,5-trimethoxybenzene (1.4 mg, 0.0083 mmol, 0.35 equiv) as an internal standard. The mixture was dissolved in THF-d_8 (0.6 mL) and immediately transferred to an NMR tube.

Upon dissolving the mixture of colourless solids in THF-d_8, a dark yellow solution was formed immediately, which became dark green over a period of 25 h (Figure S5).

The tube was sealed and removed from the glovebox for NMR analysis (Figures S6-S10). The NMR tube was stored at RT and analysed at various time intervals over a period of 25 h. The NMR tube was then irradiated with blue LEDs (450 nm) for 16 h before further analysis.

(a) 2 min after mixing (b) 25 h after mixing (c) after 16 h irradiation

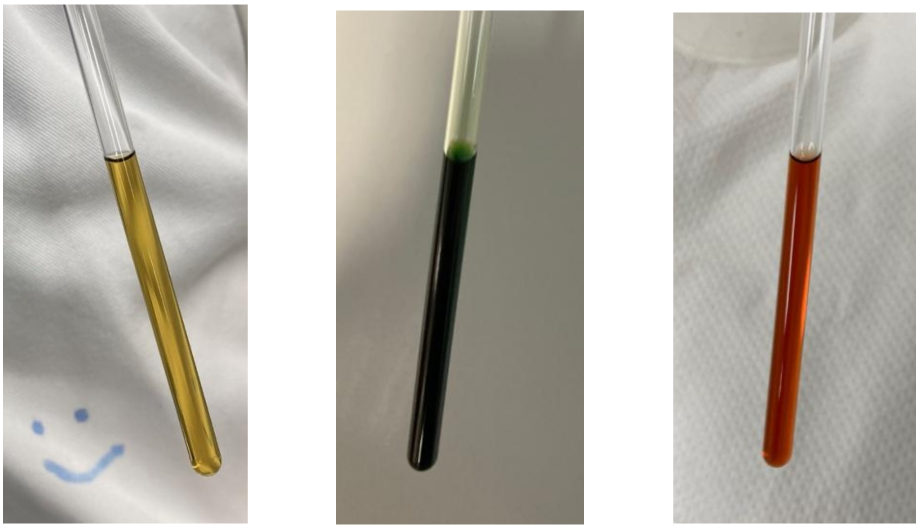

Figure S5. Change in colour of 1:1 dtbbpy/B_2cat_2 solutions in THF-d_8 over time and after irradiation

^{1}H NMR:

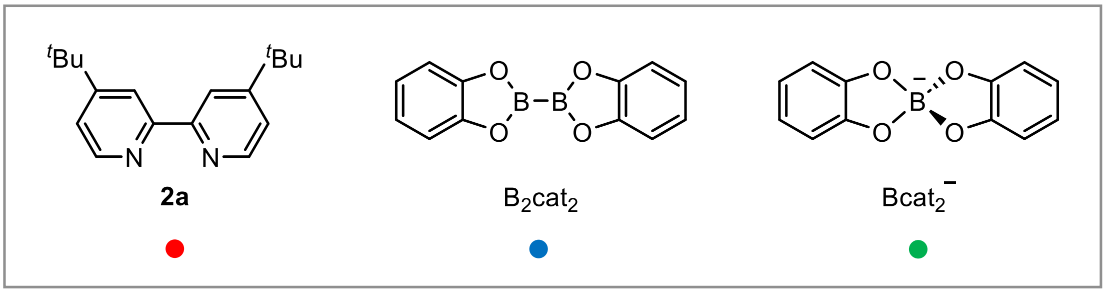

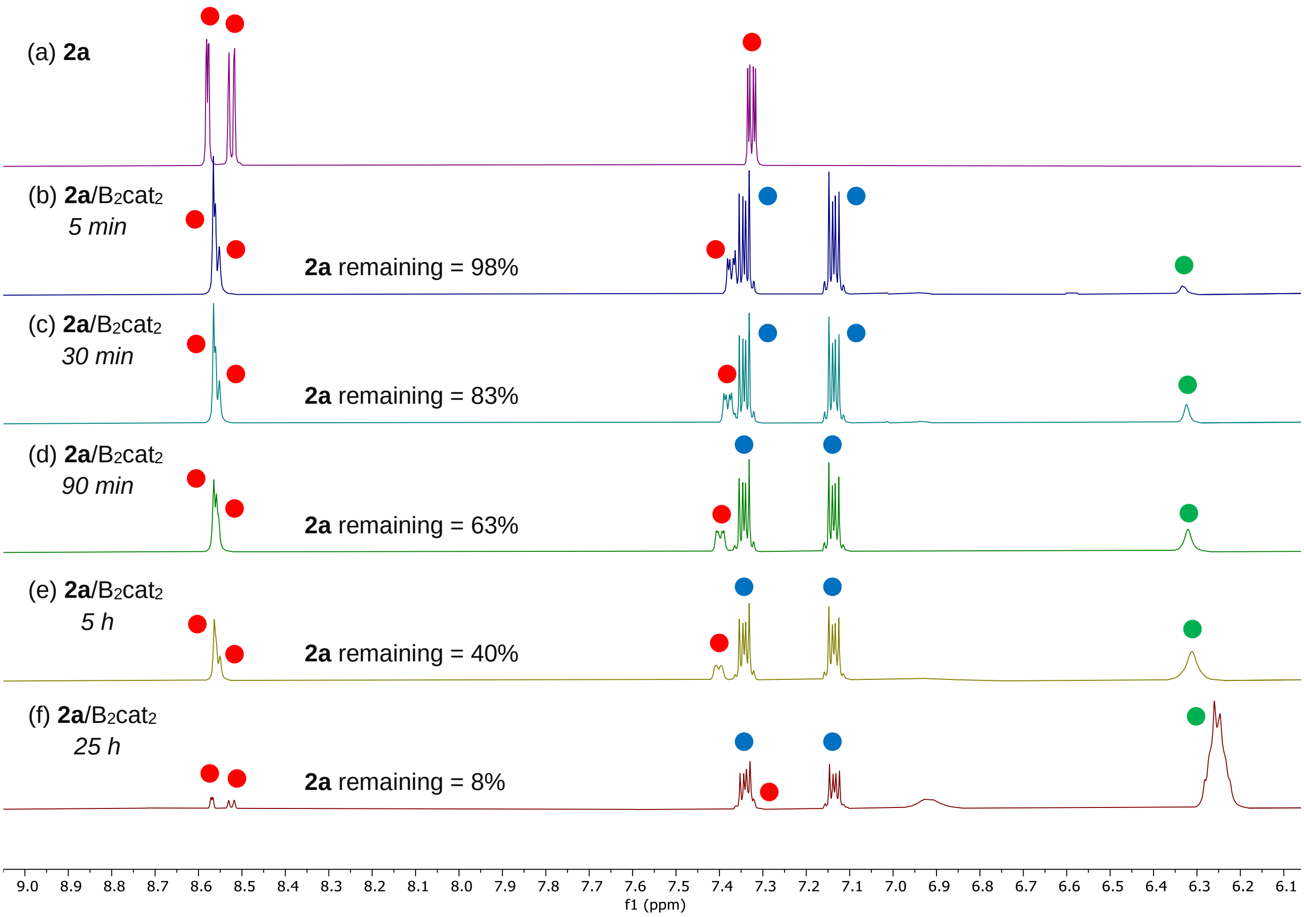

Figure S6. ^{1}H NMR spectra of 1:1 dtbbpy/B_2cat_2 in THF-d_8

The times shown in spectra (b)–(f) indicate the period in between dissolving dtbbpy (**2a**) and B_2cat_2 in THF-d_8 and recording the ^{1}H NMR spectrum. The amount of **2a** remaining was determined using 1,3,5-trimethoxybenzene (peaks not seen in the above chemical shift range) as an internal standard.

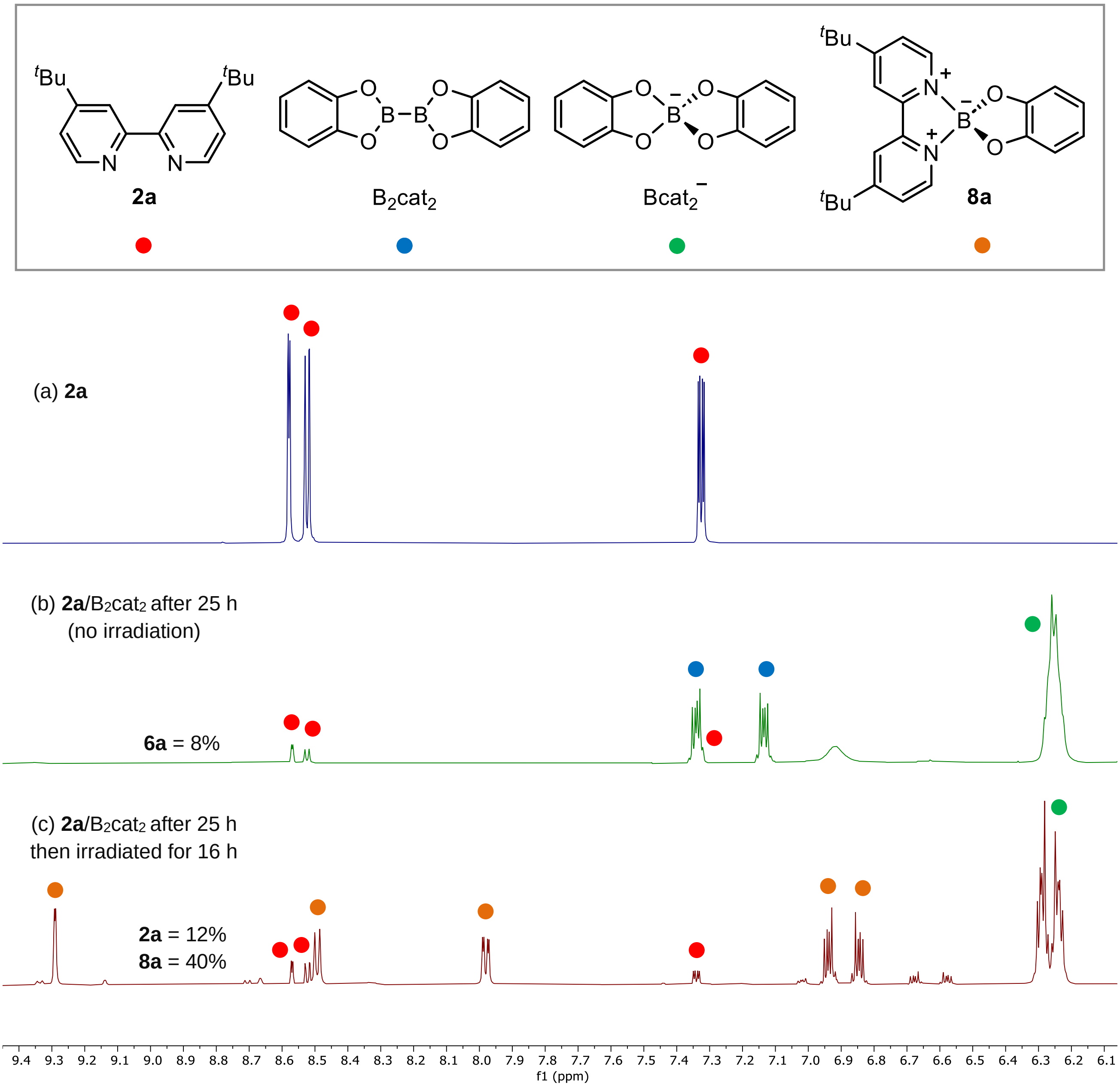

Figure S7. ^{1}H NMR spectra of 1:1 dtbbpy/B_2cat_2 in THF-d_8 after irradiation (6.1– 9.4ppm)
Spectrum (b) was recorded 25 h after dissolving dtbbpy (**2a**) and B_2cat_2 in THF-d_8. This sample was then irradiated in the NMR tube with blue LEDs (450 nm) for 16 h before recording spectrum (c). The amount of **2a** remaining and **8a** formed were determined using 1,3,5-trimethoxybenzene (peaks not seen in the above chemical shift range) as an internal standard.

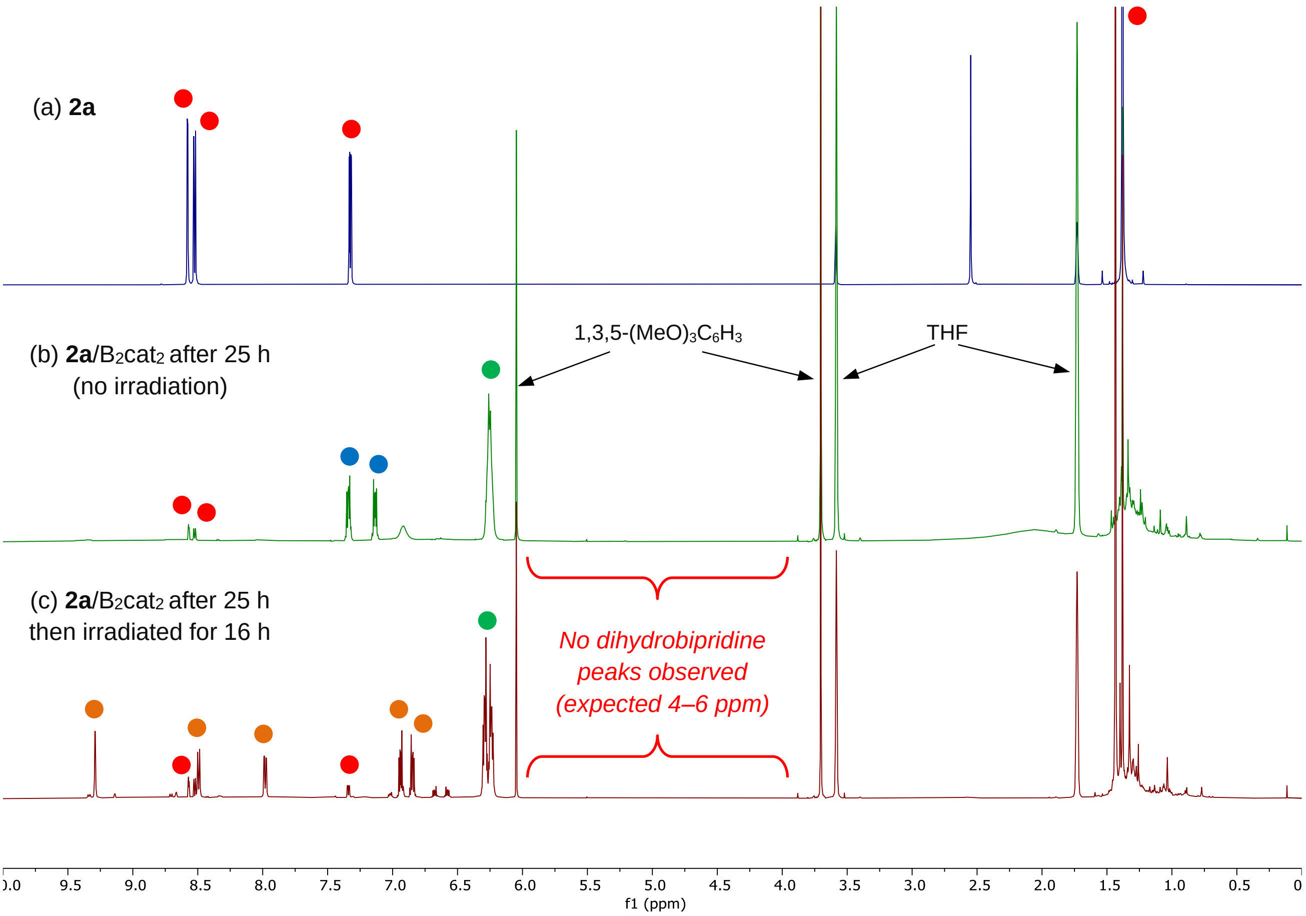

Figure S8. ^{1}H NMR spectra of 1:1 dtbbpy/B_2cat_2 in THF-d_8 after irradiation (0–10 ppm)

Spectrum (b) was recorded 25 h after dissolving dtbbpy (**2a**) and B_2cat_2 in THF-d_8. This sample was then irradiated in the NMR tube with blue LEDs (450 nm) for 16 h before recording spectrum (c). The amount of **2a** remaining and **8a** formed were determined using 1,3,5-trimethoxybenzene (peaks not seen in the above chemical shift range) as an internal standard.

^{11}B NMR:

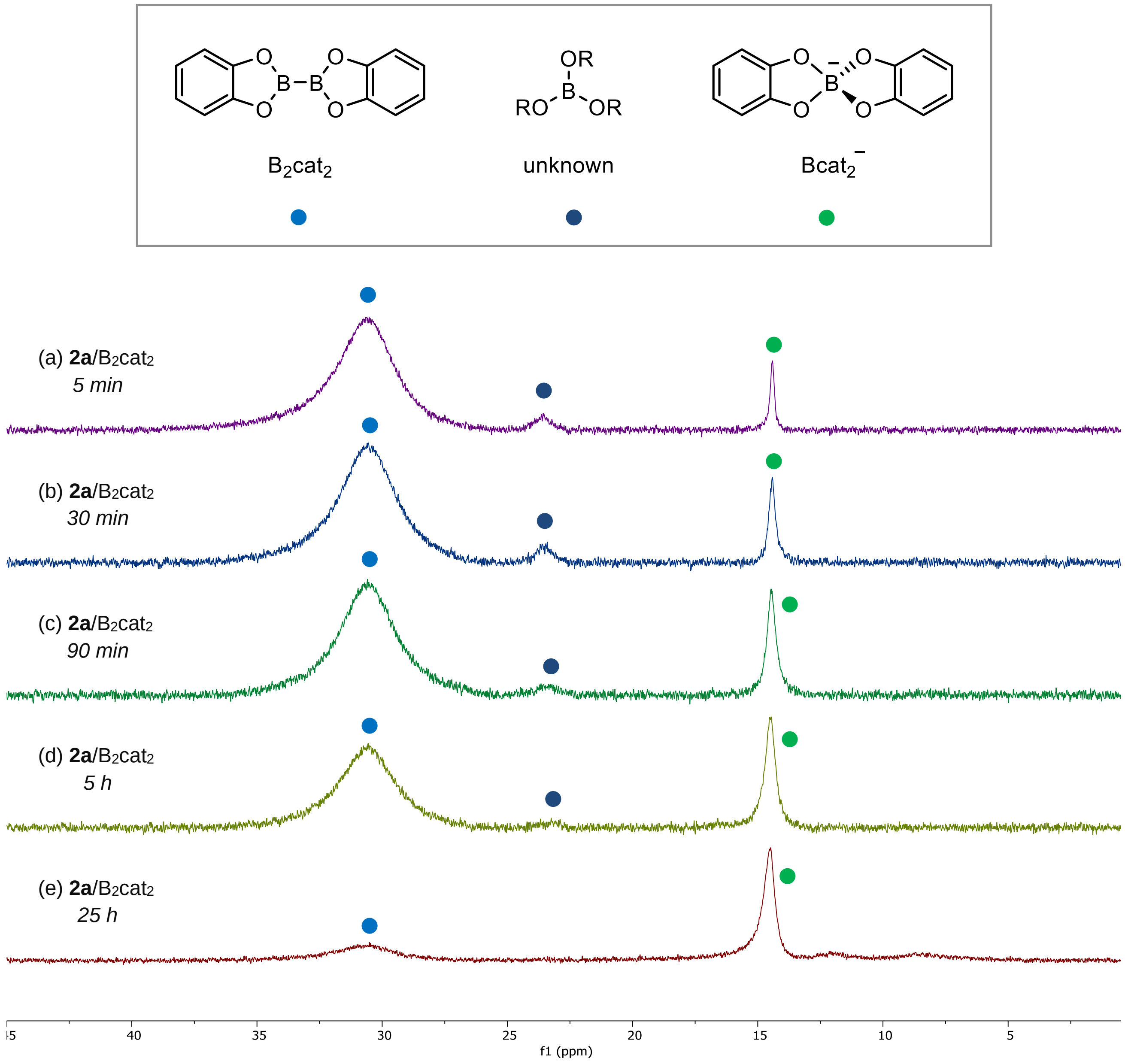

Figure S9. ^{11}B NMR spectra of 1:1 dtbbpy/B_2cat_2 in THF-d_8

The times shown in spectra (a)–(e) indicate the period in between dissolving dtbbpy (**2a**) and B_2cat_2 in THF-d_8 and recording the ^{11}B NMR spectrum.

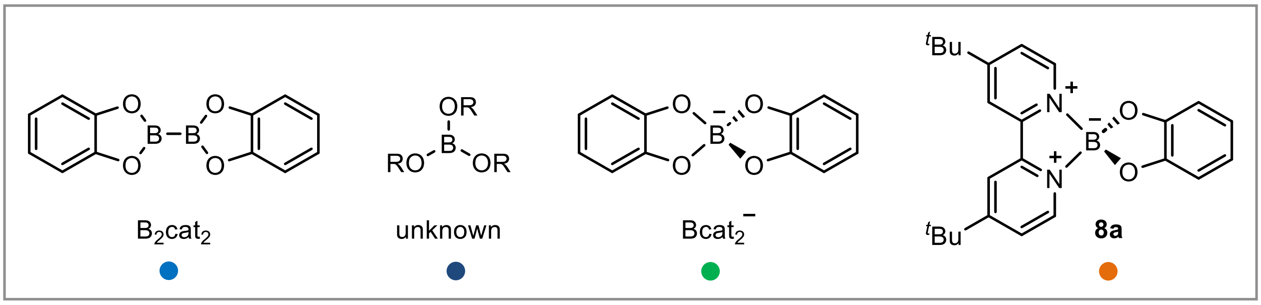

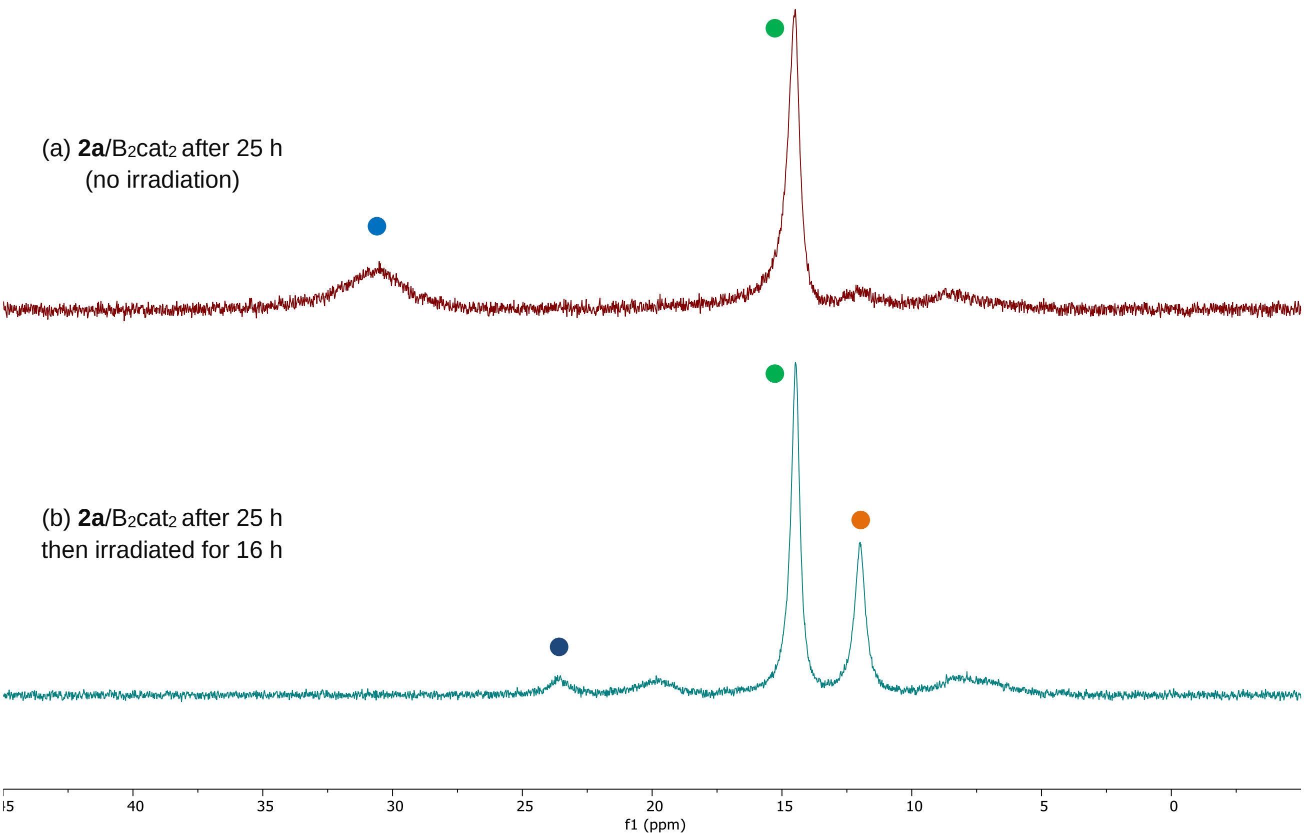

Figure S10. ^{11}B NMR spectra of 1:1 dtbbpy/B_2cat_2 in THF-d_8 after irradiation

Spectrum (b) was recorded 25 h after dissolving dtbbpy (**2a**) and B_2cat_2 in THF-d_8. This sample was then irradiated in the NMR tube with blue LEDs (450 nm) for 16 h before recording spectrum (c).

3.2. Synthesis of Boronium Ions

4,4'-Di-*tert*-butyl-2,2'-bipyridylbenzo[*d*][1,3,2]dioxaboronium hexafluorophosphate (8a•PF_6)

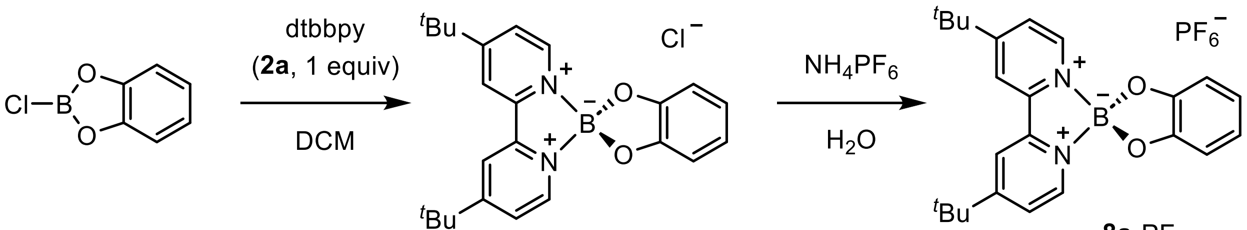

Boronium **8a**•PF_6 was prepared following a modified literature procedure.[30]

To a flame-dried Schlenk flask was added 4,4'-di-*tert*-butyl-2,2'-bipyridine (**2a**, 805 mg, 3.00 mmol), after which the flask was evacuated and back-filled with nitrogen three times. Subsequently, anhydrous DCM (3.0 mL) was added via syringe. Then 2-chlorobenzo[*d*][1,3,2]dioxaborole (ClBcat, 463 mg, 3.00 mmol.) in DCM (3.0 mL) was added dropwise to the solution and an orange precipitate was formed immediately. After addition, the reaction was stirred for 10 minutes, the solid was filtered, washed with DCM (2 × 10 mL) and dried under vacuum to give **8a**•Cl, which was dissolved in H_2O (10 mL). A solution of ammonium hexafluorophosphate (489 mg, 3.00 mmol) in H_2O (5.0 mL) was added to the stirred solution of **8a**•Cl. A fine yellow precipitate was formed immediately. After 5 min, the yellow solid was collected by filtration and washed with water (10 mL) and pentane (10 mL) to provide **8a**•PF_6 (1.50 g, 94%) as a yellow solid. Crystals for X-ray analysis were obtained as fine needles by crystallizing **8a**•PF_6 from cooled acetone (co-crystallized with acetone).

NMR Spectroscopy (*see spectra*):

^{1}**H NMR** (400 MHz, acetone-*d*$_6$) δ_H 9.19 (dd, *J* = 1.8, 0.7 Hz, 2H), 8.92 (dd, *J* = 6.1, 0.7 Hz, 2H), 8.32 (dd, *J* = 6.1, 1.8 Hz, 2H), 6.98 – 6.92 (m, 2H), 6.92 – 6.87 (m, 2H), 1.55 (s, 18H);

^{13}**C NMR** (101 MHz, acetone-*d*$_6$) δ_C 174.9, 151.5, 145.8, 144.6, 127.8, 121.6, 121.5, 111.5, 38.0;

^{31}**P NMR** (162 MHz, acetone-*d*$_6$) δ_P 144.25 (hept, *J* = 707.7 Hz);

^{19}**F NMR** (377 MHz, acetone-*d*$_6$) δ_F -72.50 (d, *J* = 707.9 Hz);

^{11}**B NMR** (128 MHz, acetone-*d*$_6$) δ_B 12.2.

IR (film): ν_{max} 2970, 1739, 1707, 1635, 1556, 1481, 1436, 1363, 1232, 1142, 1097, 1037, 833, 747, 557.

HRMS (Nanospray): calculated for $C_{24}H_{28}BN_2O_2$ [M – PF_6^-]$^+$ 387.2238, found 387.2246.

2,2'-Bipyridylbenzo[*d*][1,3,2]dioxaboronium hexafluorophosphate (8d•PF_6)

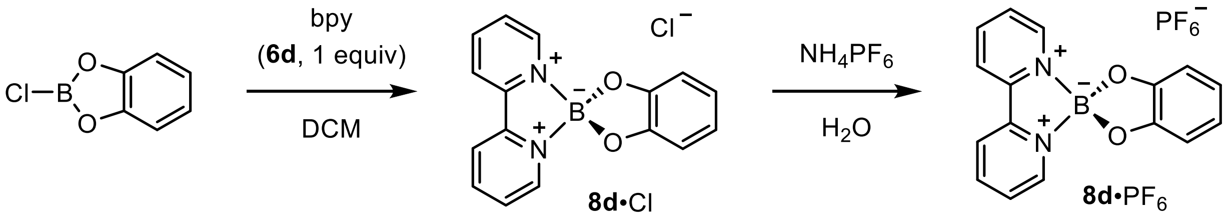

Boronium **8d**•PF_6 was prepared following a modified literature procedure.[30]

To a flame-dried Schlenk flask was added 2,2'-bipyridine (**2d**, 469 mg, 3.00 mmol), after which the flask was evacuated and back-filled with nitrogen three times. Subsequently, anhydrous DCM (3.0 mL) was added via syringe. Then 2-chlorobenzo[*d*][1,3,2]dioxaborole (ClBcat, 463 mg, 3.00 mmol.) in DCM (3.0 mL) was added dropwise to the solution and an orange precipitate was formed immediately. After addition, the reaction was stirred for 10 minutes, the solid was filtered, washed with DCM (2 × 10 mL) and dried under vacuum to give **8d**•Cl, which was dissolved in H_2O (10 mL). A solution of ammonium hexafluorophosphate (489 mg, 3.00 mmol) in H_2O (5.0 mL) was added to the stirred solution of **8d**•Cl. A fine yellow precipitate was formed immediately. After 5 min, the yellow solid was collected by filtration and washed with water (10 mL) and pentane (10 mL) to provide **8d**•PF_6 (1.01 g, 80%) as a yellow solid. Crystals for X-ray analysis were obtained as fine needles by crystallization from boiling water.

NMR Spectroscopy (*see spectra*):

> **^{1}H NMR** (400 MHz, acetone-*d*$_6$) δ_H 9.10 (dt, *J* = 8.0, 1.1 Hz, 2H), 9.07 (d, *J* = 5.5 Hz, 2H), 9.00 (td, *J* = 7.9, 1.4 Hz, 2H), 8.36 (ddd, *J* = 7.7, 5.6, 1.1 Hz, 2H), 7.01 – 6.96 (m, 2H), 6.94 – 6.88 (m, 2H);
>
> **^{13}C NMR** (101 MHz, acetone-*d*$_6$) δ_C 151.4, 148.4, 145.9, 143.9, 128.8, 124.2, 121.8, 111.6;
>
> **^{31}P NMR** (162 MHz, acetone-*d*$_6$) δ_P 144.24 (hept, *J* = 707.7 Hz);
>
> **^{19}F NMR** (377 MHz, acetone-*d*$_6$) δ_F -72.50 (d, *J* = 707.9 Hz);
>
> **^{11}B NMR** (128 MHz, acetone-*d*$_6$) δ_B 12.5.

IR (film): ν_{max} 3082, 1738, 1636, 1513, 1482, 1365, 1232, 1148, 1036, 836, 779, 728, 557.

HRMS (Nanospray): calculated for $C_{16}H_{12}BN_2O_2$ [M – PF_6^-]$^+$ 275.0986, found 275.0994.

3.3. Boryl Radical Formation by Reduction of Boronium Ions

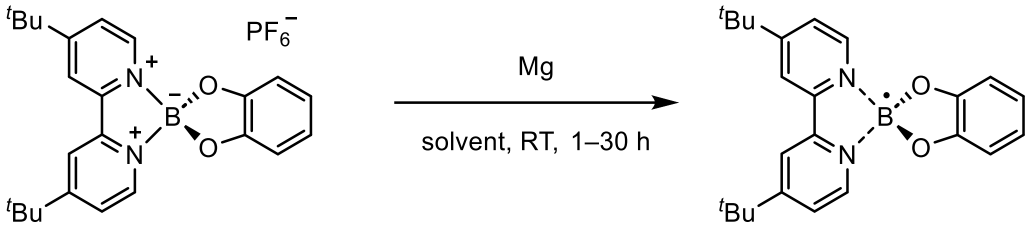

8a•PF_6 **3a**

DMF: In glovebox, to a 7.0 mL vial equipped with a magnetic stir bar was added 4,4'-di-*tert*-butyl-2,2'-bipyridylbenzo[d][1,3,2]dioxaboronium hexafluorophosphate (**8a**•PF_6, 21.3 mg, 0.0400 mmol) and Mg (9.7 mg, 0.40 mmol, 10 equiv.). Subsequently, anhydrous DMF (1.0 mL) was added, and the mixture was allowed to stir at RT. After 30 min, the reaction solution slowly changed to blue, after 1 h the solution was changed to deep blue, which suggested that the boryl radical was formed in the reaction. After this time, samples were submitted for analysis by EPR and UV-Vis spectroscopy (see Sections 3.4 and 3.5).

NMP: When the above reaction was performed in NMP, a similar colour change was observed to blue after 30 min and deep blue after 1 h.

THF: When the above reaction was performed in THF, a slow colour change was observed to blue after 12 h and deep blue after 30 h.

3.4. Electron Paramagnetic Resonance Spectroscopy

Spectrometer Details: Electron paramagnetic resonance (EPR) spectra were recorded using an Active Spectrum X-band Micro-ESR Spectrometer.

Run Details: O.D. 2 mm, L 250 mm Wilmad® quartz (CFQ) EPR tubes, 40 scans, 75 ms delay, field sweep 3400–3600 G, microwave power 15–50 mW (as indicated on spectra), modulation amplitude 40%, microwave frequency 9.787272 GHz, reflected power –15.5 dBm, temperature 24 °C.

Sample preparation:

*A) dtbbpy (**2a**) with B_2cat_2*

In a glovebox, to a 7.0 mL vial equipped with a magnetic stir bar was added 4,4'-di-*tert*-butyl-2,2'-bipyridine (**2a**, 10.8 mg, 0.0402 mmol) and B_2cat_2 (9.5 mg, 0.040 mmol, 1.0 equiv). Subsequently, anhydrous DMF (1.0 mL) was added, and the mixture was allowed to stir at room temperature for 1 h. Then the reaction was then transferred into the EPR tube. The tube was removed from the glovebox and ready for EPR measurement (Figure S11).

*B) (dtbbpyBcat)(PF_6) (**8a**•PF_6) with Mg*

In a glovebox, to a 7.0 mL vial equipped with a magnetic stir bar was added **8a**•PF_6 (21.3 mg, 0.0400 mmol) and Mg (9.6 mg, 0.40 mmol, 10 equiv). Subsequently, anhydrous DMF (1.0 mL) was added, and the mixture was allowed to stir at room temperature for 30 h. Then the reaction was filtered to remove the excess Mg metal and carefully transferred into the EPR tube. The tube was removed from the glovebox and ready for EPR measurement (Figure S11).

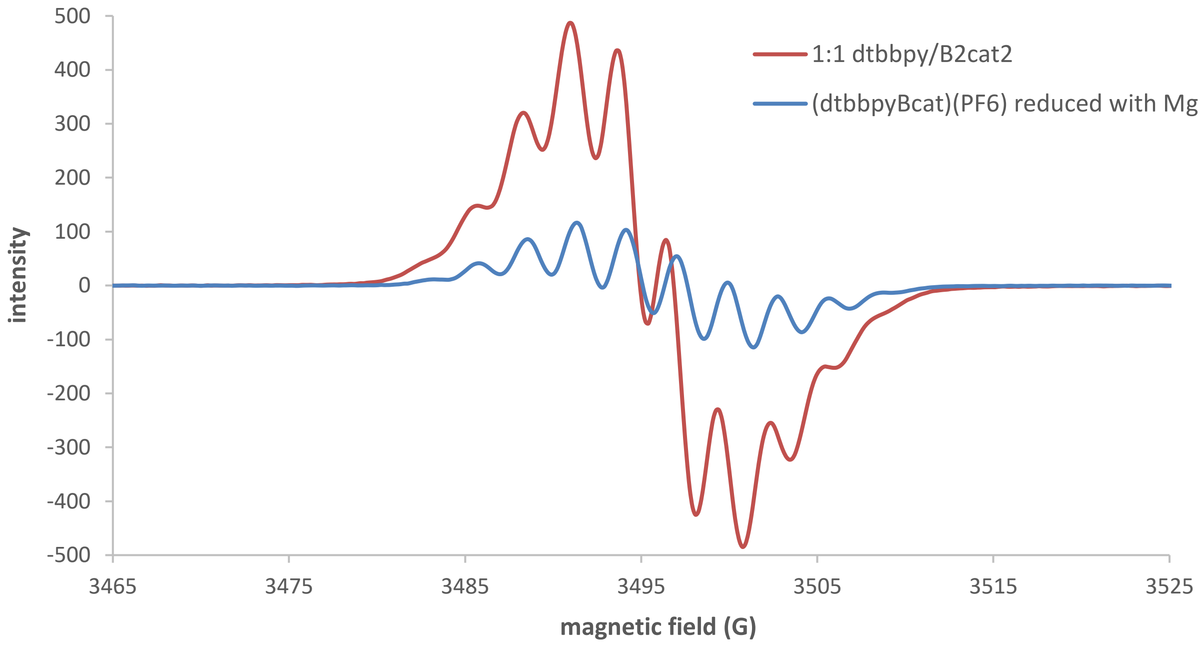

Figure S11. EPR spectra of dtbbpy/B_2cat_2 and **8a**•PF_6/Mg in DMF (microwave power = 10 mW for dtbbpy/B_2cat_2 and 15 mW for (dtbbpyBcat)(PF_6)/Mg)

EPR spectra were also recorded for 1:1 mixtures of dtbbpy (**2a**) and B_2cat_2 in NMP (0.04 M) and THF (0.04 M). The samples were prepared following the analogous procedure to that described above for DMF (0.04 M). The spectra in Figure S12 show that the same radical species is formed in DMF, NMP and THF.

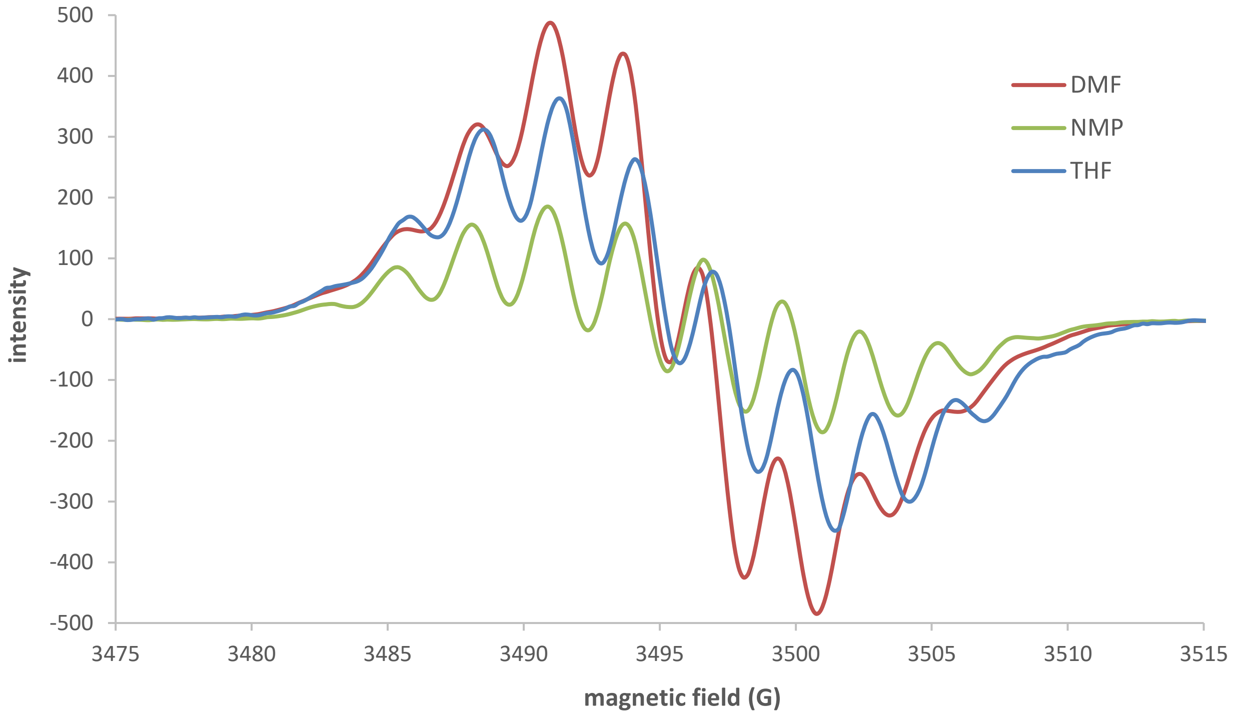

Figure S12. EPR spectra of 1:1 dtbbpy/B_2cat_2 in DMF, NMP, and THF (microwave power = 10 mW for DMF, 25 mW for NMP and 50 mW for THF)

DFT Calculations:

Calculations were performed using the Gaussian09[31] suite of programmes. Geometry optimisation was performed with the uM062X/6-311g(d,p) level of theory. The analysis also provided zero-point vibrational energy corrections and thermal corrections to various thermodynamic properties. EPR calculations were carried out at the uM062X/EPR-III level of theory.[32]

Table S3. DFT calculations data

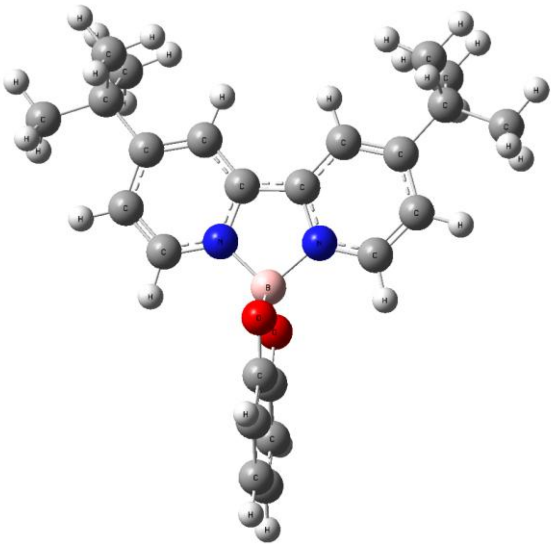

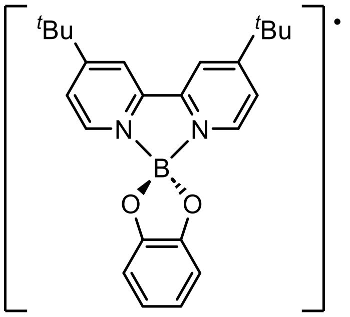

3a

C	-2.95134	-2.22376	0.54645
C	-1.52507	-2.19758	0.46599
C	-0.86718	-0.99868	0.40934
C	-2.90523	0.19235	0.50607
C	-3.61738	-1.01823	0.56511
H	-0.94496	-3.10728	0.44912
H	0.21288	-0.92600	0.34702
H	-4.69872	-0.97958	0.62527
C	-2.43215	3.70931	0.43164
C	-3.67714	4.27555	0.48846
C	-4.83534	3.44209	0.55673
C	-4.64703	2.07768	0.56789
C	-3.35176	1.53439	0.51038
H	-1.52331	4.29810	0.37734
H	-3.75703	5.35149	0.47928
H	-5.48998	1.39877	0.61803
C	1.08176	2.26875	-0.49177
C	1.18093	2.29113	0.90398
C	2.14281	2.62621	-1.29451
C	2.34463	2.67044	1.53638
C	3.33111	3.01385	-0.65825

H	2.04997	2.60396	-2.37281
C	3.42993	3.03521	0.72602
H	2.40617	2.68208	2.61706
H	4.18455	3.30041	-1.26071
H	4.35932	3.33851	1.19233
O	-0.15925	1.86008	-0.86451
O	0.00520	1.89770	1.46115
B	-0.90178	1.60445	0.36150
N	-2.26243	2.37757	0.44012
N	-1.52839	0.16971	0.42737
C	-6.25113	4.01840	0.61146
C	-6.25011	5.55064	0.60661
H	-5.71533	5.95527	1.46950
H	-5.79719	5.95043	-0.30398
H	-7.27988	5.91198	0.65282
C	-7.04341	3.53017	-0.61313
H	-7.12544	2.44146	-0.63036
H	-8.05505	3.94486	-0.59269
H	-6.55878	3.84755	-1.53947
C	-6.94635	3.53895	1.89663
H	-6.38818	3.85684	2.78049
H	-7.95419	3.95900	1.95522
H	-7.03320	2.45092	1.92508
C	-3.73865	-3.53399	0.60789
C	-2.81982	-4.75968	0.57666
H	-2.13842	-4.77257	1.43092
H	-3.42628	-5.66717	0.61967
H	-2.22913	-4.79650	-0.34198
C	-4.55680	-3.57479	1.90949
H	-5.12157	-4.50946	1.96585
H	-3.90082	-3.51502	2.78118
H	-5.26743	-2.74761	1.96334
C	-4.68947	-3.60924	-0.59865
H	-5.40448	-2.78417	-0.59734
H	-4.12873	-3.56962	-1.53549
H	-5.25355	-4.54558	-0.57257

G = -1215.739905 Hartree

DFT calculation indicated that the SOMO of **3a** is localised on the bipyridyl unit (Figure S13), which is in agreement with previous calculations on a related bipyridyl-boryl radical reported by Russel and Norman.[33]

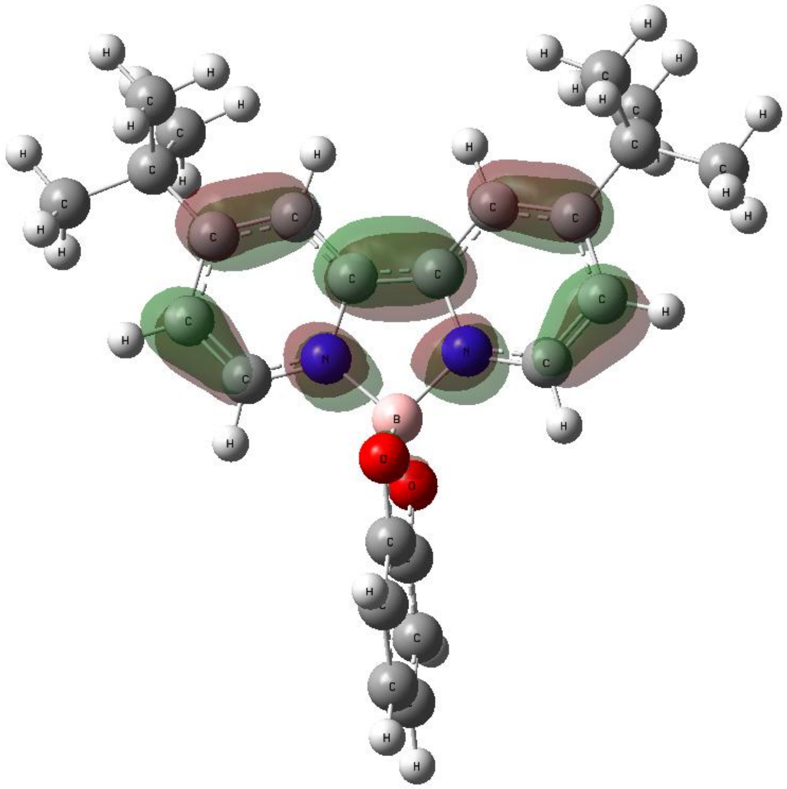

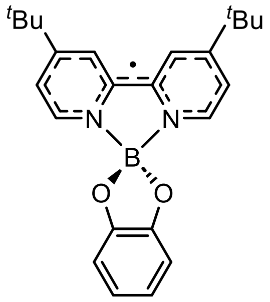

3a

Figure S13. Singly Occupied Molecular Orbital (Isovalue = 0.04)

Simulation Results (using EasySpin)[34]:

Computed EPR Hyperfine Coupling Constants:

A $H_{3/3'}$ = –2.7 MHz (–1.0 G),

A $H_{5/5'}$ = –8.3 MHz (–3.0 G),

A $H_{6/6'}$ = –1.1 MHz (–0.4 G),

A $N_{1/1'}$ = 9.8 MHz (3.5 G),

A B = –8.0 MHz (–2.8 G).

Additional EPR Parameters simulation:

g = 2.017, lwpp = 0.25 mT, Exp. Center Sweep = 349.6 mT; Exp. nPoints = 1200.

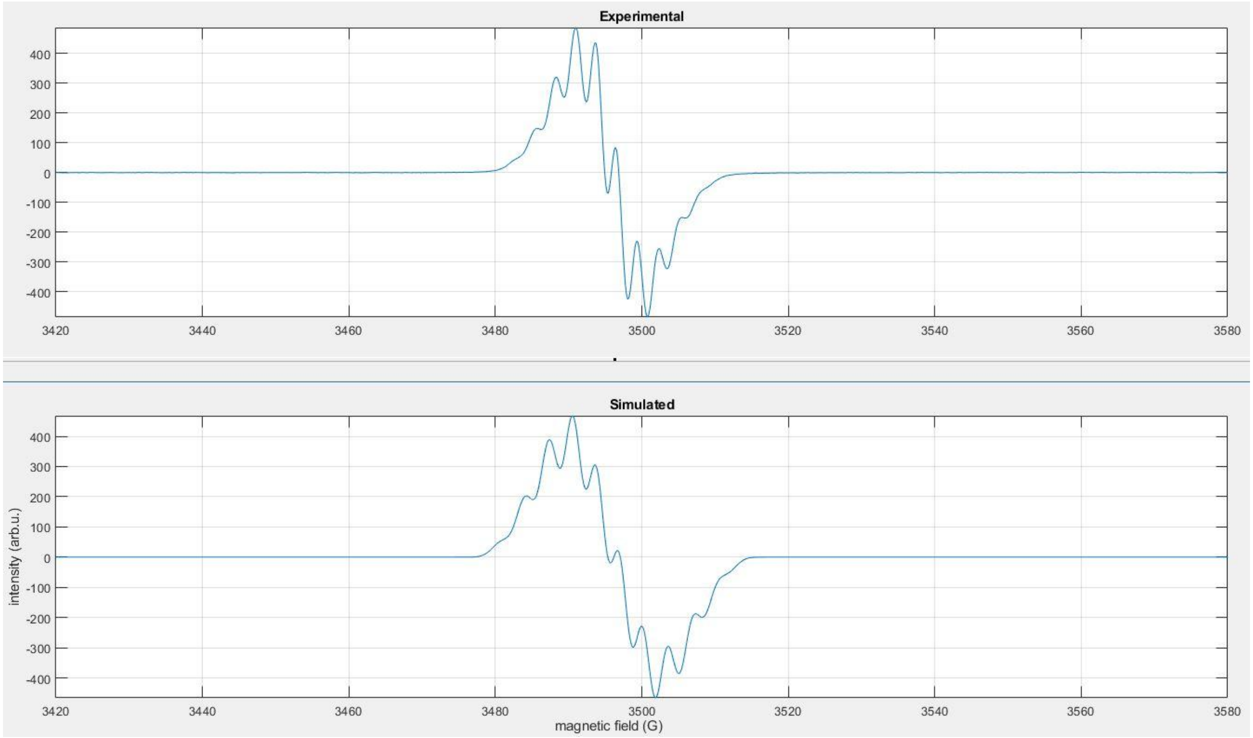

EasySpin Script (for MATLAB):

```
sys.g = 2.017;
sys.Nucs = '1H,1H,1H,1H,1H,1H,14N,14N,11B';
sys.A = [-8.34; -1.08; -2.74; -1.06; -8.25; -2.76; 9.8; 9.8; -8.0]; %mHz
sys.lwpp = 0.25; %mT
Exp.mwFreq = 9.87; %GT
Exp.CenterSweep = [349.6 20];
Exp.nPoints = 1200;
garlic(sys, Exp);
```

3.5. UV-Vis Spectroscopy

Spectrometer Details: All absorption spectra were measured using a GENESYS™ 10S UV-Visible Spectrophotometer (Thermo Scientific) in a 4.5 cm quartz cuvette (path length = 1.0 cm).

Sample preparation:

*A) dtbbpy (**2a**) with B_2cat_2:* In a glovebox, to a 7.0 mL vial equipped with a magnetic stir bar was added 4,4'-di-*tert*-butyl-2,2'-bipyridine (**2a**, 10.8 mg, 0.0402 mmol) and B_2cat_2 (9.5 mg, 0.040 mmol, 1.0 equiv). Anhydrous DMF (4.0 mL) was added, and the mixture was allowed to stir at room temperature for 1 h. Subsequently, 80 µL of this mixture was transferred to a cuvette and the sample was diluted to a total volume of 4.0 mL with dry DMF to make a 0.2 mM solution. The cuvette was sealed and removed from the glovebox for UV-Vis analysis.

*B) (dtbbpyBcat)(PF_6) (**8a**•PF_6) with Mg:* In a glovebox, to a 7.0 mL vial equipped with a magnetic stir bar was added **8a**•PF_6 (21.3 mg, 0.0400 mmol) and Mg (9.6 mg, 0.40 mmol, 10 equiv). Anhydrous DMF (1.0 mL) was added, and the mixture was allowed to stir at room temperature for 1 h. Subsequently, 40 µL of this mixture was transferred to a cuvette and the sample was diluted to a total volume of 4.0 mL with dry DMF to make a 0.1 mM solution. The cuvette was sealed and removed from the glovebox for UV-Vis analysis.

The UV/Vis spectra of **8a**•PF_6 (0.1 mM), **8a**•PF_6 (0.1 mM) after reduction with Mg, and a 1:1 dtbbpy/B_2cat_2 mixture (0.2 mM) are shown in Figure S14. The spectrum of **8a**•PF_6 changed dramatically upon reduction with Mg, which is indicates the formation of boryl radical **3a**. Overlaying the normalised (using λ_{max} = 435 nm) spectrum obtained from the 1:1 dtbbpy/B_2cat_2 mixture shows almost identical absorbance features between 350 and 500 nm, which indicates the formation of the same boryl radical.

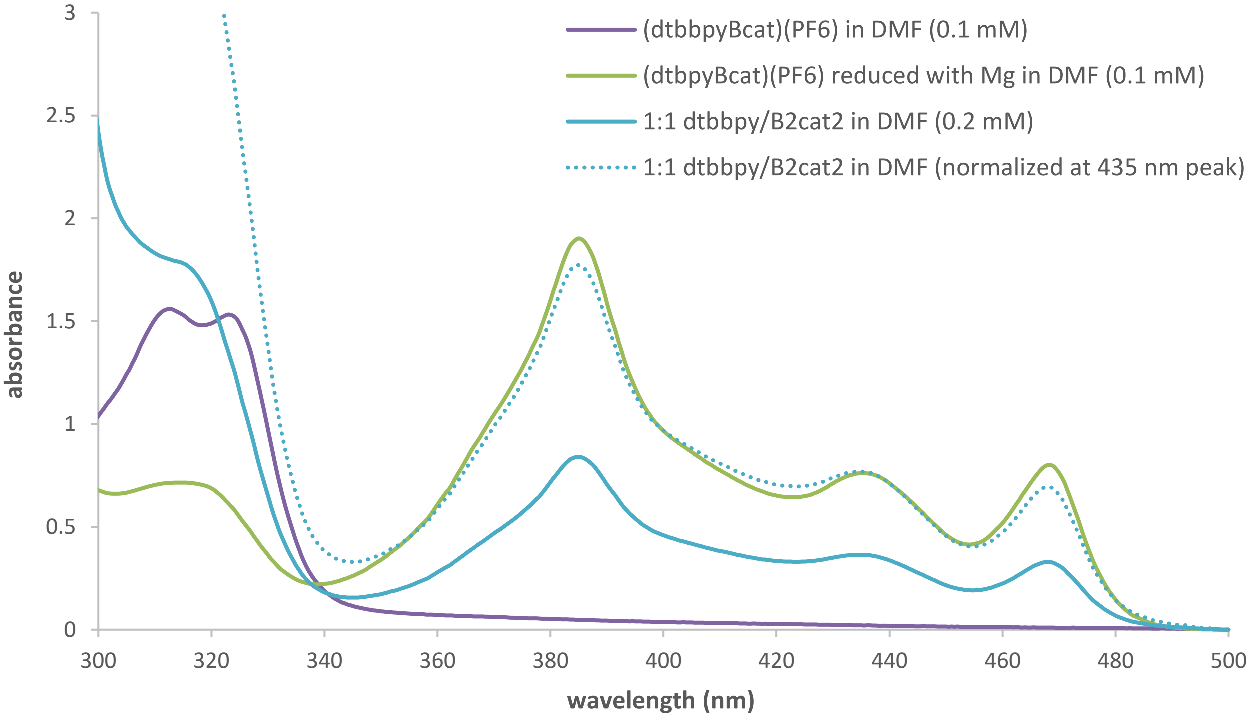

Figure S14. UV-Vis absorption spectra in DMF

3.6. Cyclic Voltammetry

Spectrometer Details: Cyclic voltammograms were measured using a PalmSens 4 with MultiTrace 4.2 software. The cell setup consisted of a Pt disk working electrode and Pt wire counter electrode. All measurement were performed under a nitrogen atmosphere in degassed MeCN or DMF with 0.1 M $TBAPF_6$ as the supporting electrolyte. Half-wave potentials ($E_{1/2}$) were normalised to the ferrocene/ferrocenium (Fc/Fc^+) redox couple and then converted to saturated calomel electrode (SCE) by adding 0.38 V.

The cyclic voltammograms for a 5.0 mM solution of (dtbbpyBcat)(PF_6) (**8a**•PF_6) in DMF [0.1 M $TBAPF_6$] at scan rates ranging from 10-300 mV are shown in Figure S15. The results showed that **8a** has 2 reduction events before –2 V. The spectra are smooth and reversible at all scan rates. The first cathodic half wave potential was determined to be $E_{1/2}^{red1}$ = –1.25 V vs. Fc/Fc^+ (–0.87 V vs. SCE) and the second was determined to be $E_{1/2}^{red2}$ = –1.81 V vs. Fc/Fc^+ (–1.43 V vs. SCE).

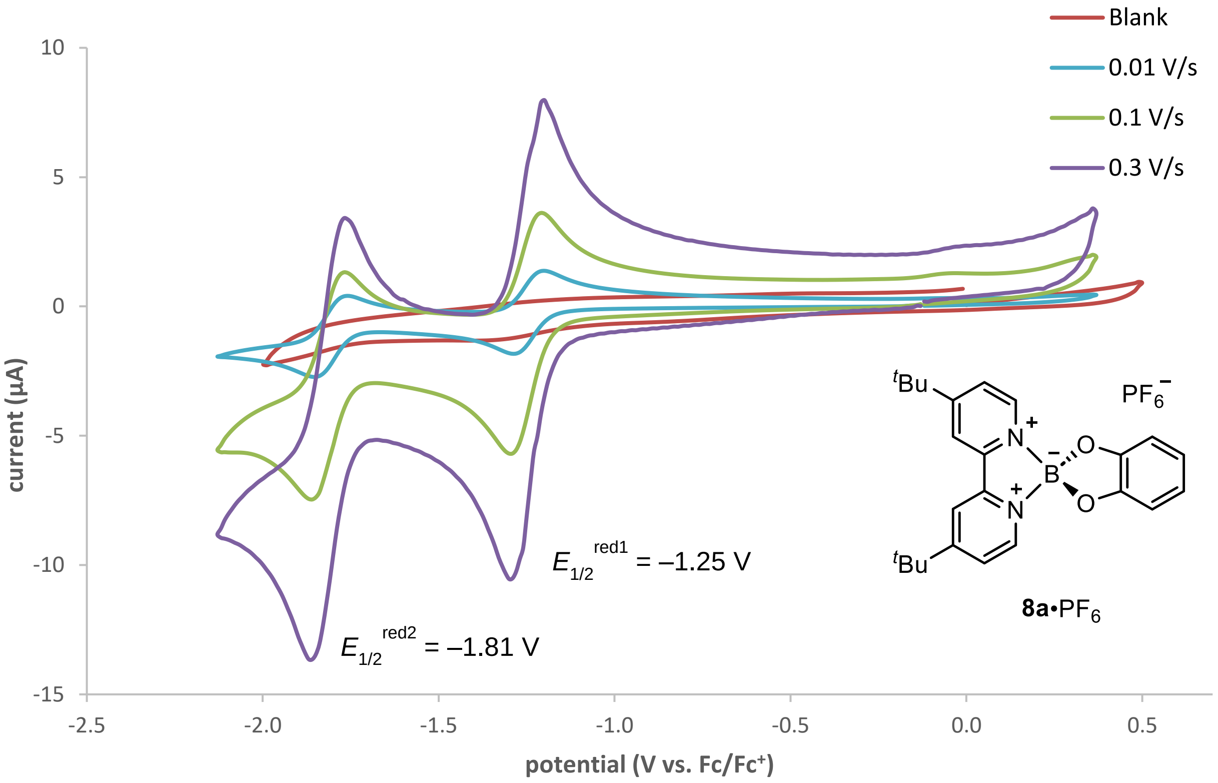

Figure S15. Cyclic voltammogram of (dtbbpyBcat)(PF_6) in DMF

The cyclic voltammograms for a 5.0 mM solution of (dtbbpyBcat)(PF_6) (**8a**•PF_6) in MeCN [0.1 M TBAPF_6] at scan rates ranging from 100-300 mV are shown in Figure S16. The first cathodic half wave potential was determined to be $E_{1/2}^{red1}$ = –1.22 V vs. Fc/Fc$^+$ (–0.84 V vs. SCE) and the second was determined to be $E_{1/2}^{red2}$ = –1.76 V vs. Fc/Fc$^+$ (–1.38 V vs. SCE).

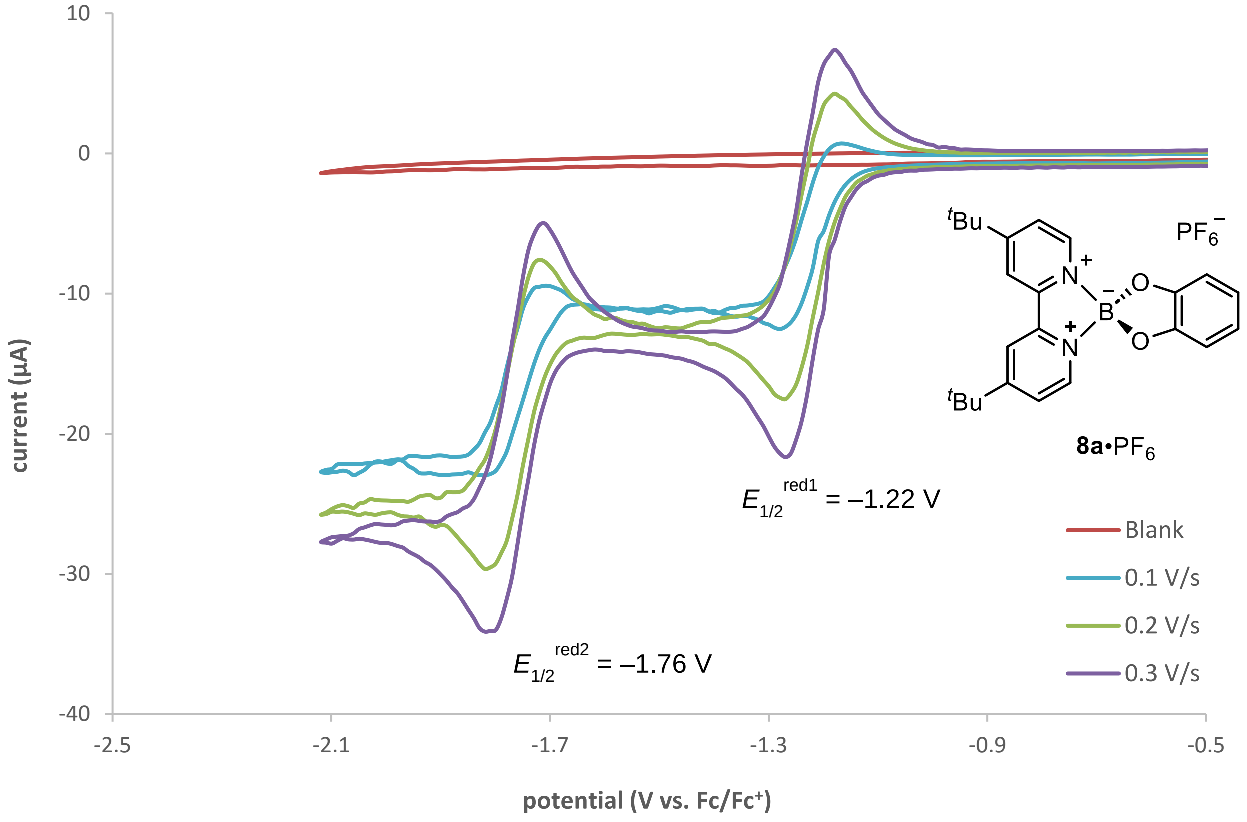

Figure S16. Cyclic voltammogram of (dtbbpyBcat)(PF_6) in MeCN

The cyclic voltammograms for a 5.0 mM solution of (bpyBcat)(PF_6) (**8d**•PF_6) in MeCN [0.1 M $TBAPF_6$] at scan rates ranging from 10-300 mV are shown in Figure S17. The first cathodic half wave potential was determined to be $E_{1/2}^{red1}$ = –1.11 V vs. Fc/Fc^+ (–0.73 V vs. SCE) and the second was determined to be $E_{1/2}^{red2}$ = –1.67 V vs. Fc/Fc^+ (–1.29 V vs. SCE).

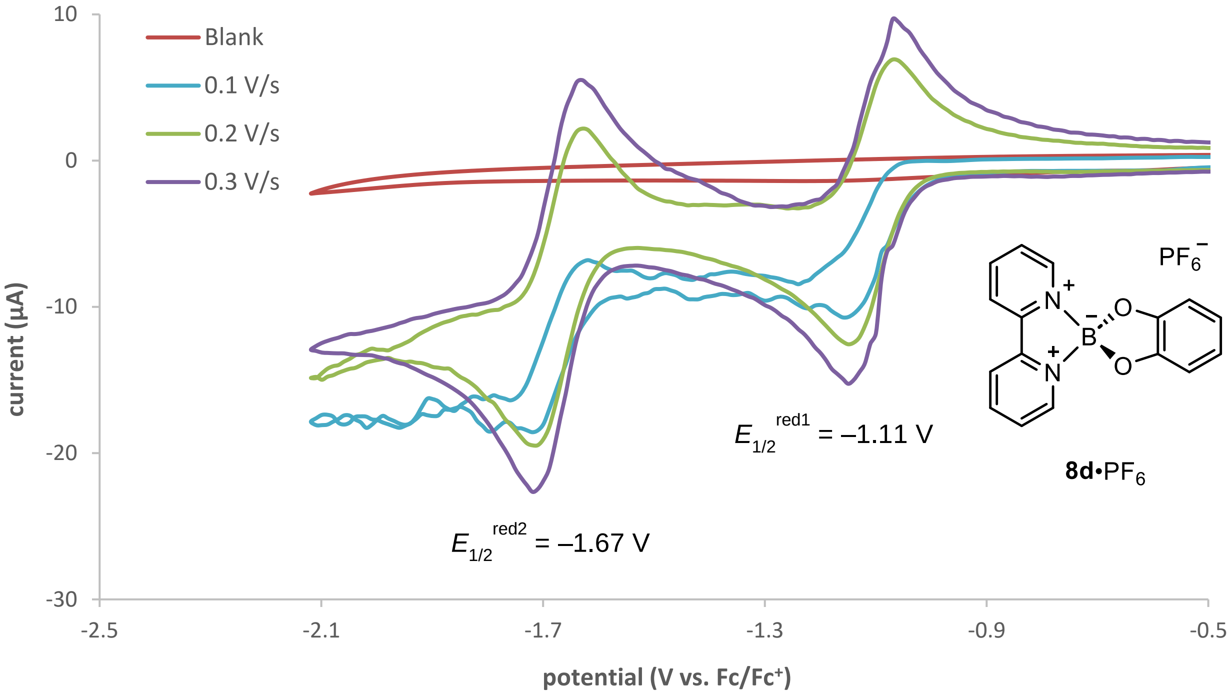

Figure S17. Cyclic voltammogram of (bpyBcat)(PF_6) in MeCN

3.7. Fluorescence Spectroscopy

Spectrometer Details: Excitation and Emission spectra were measured using a PerkinElmer LS-45 Fluorescence Spectrometer in a 4.5 cm quartz cuvette (path length = 1.0 cm).

Sample preparation: In glovebox, to a 7.0 mL vial equipped with a magnetic stir bar was added 4,4'-di-*tert*-butyl-2,2'-bipyridine (**2a**, 10.8 mg, 0.0402 mmol) and B_2cat_2 (9.5 mg, 0.040 mmol, 1.0 equiv). Anhydrous DMF (4.0 mL) was added, and the mixture was allowed to stir at room temperature for 1 h. Subsequently, 80 μL of this mixture was transferred to a cuvette and the sample was diluted to a total volume of 4.0 mL with dry DMF to make a 0.2 mM solution. The cuvette was sealed and removed from the glovebox for analysis.

Figure S18 shows the normalised absorbance, excitation and emission spectra for a 0.2 mM solution of a 1:1 dtbbpy/B_2cat_2 mixture in DMF. The excitation spectrum was obtained over the excitation range 300-495 nm while measuring the emission intensity at 500 nm. It shows a maximum excitation wavelength of 460 nm, which is close to the wavelength of the 450 nm blue LEDs used in the borylation reactions. The emission spectrum was measured at an excitation wavelength of 460 nm and shows the emission intensity over 475-600 nm. It shows a maximum emission at 506 nm.

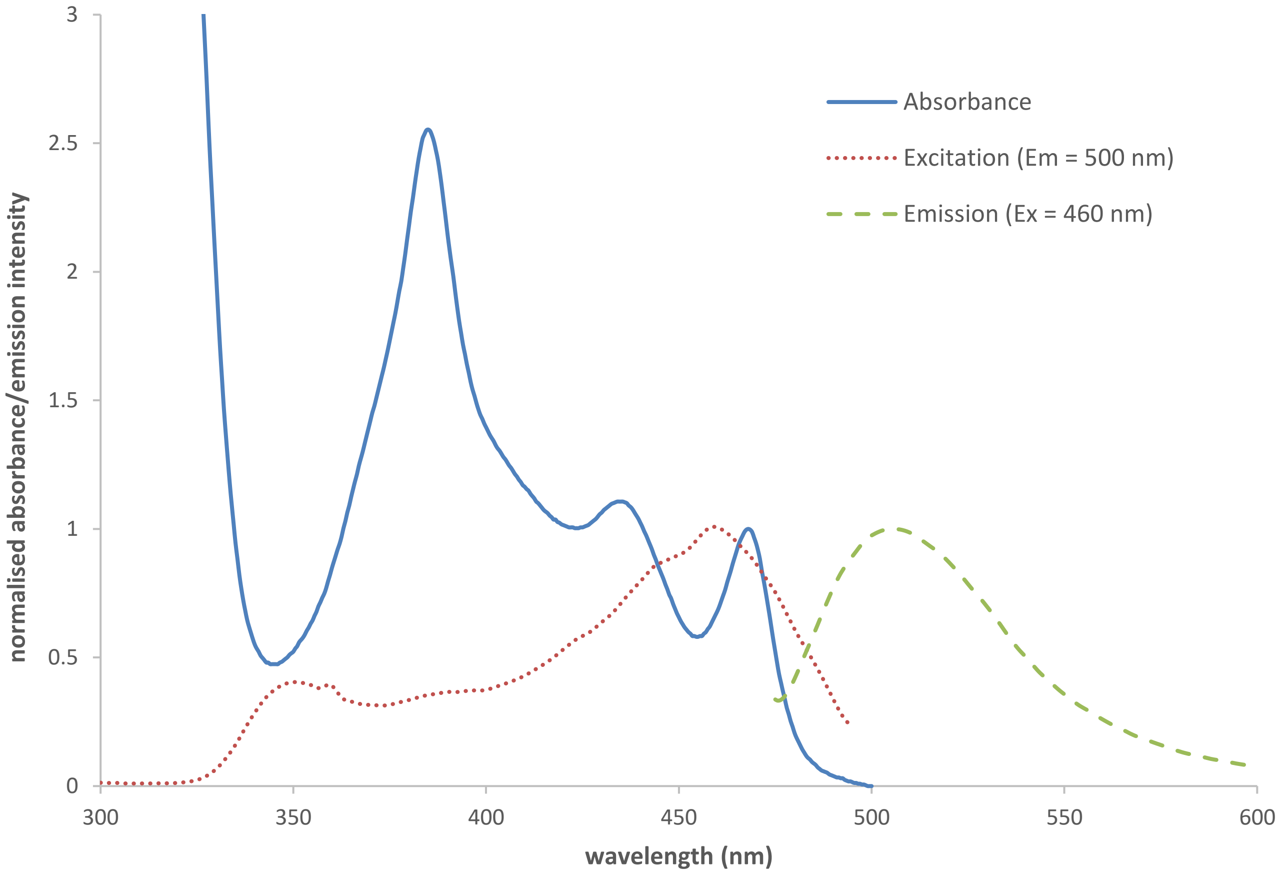

Figure S18. Normalised absorbance, excitation and emission spectra of dtbbpy/B_2cat_2 in DMF

3.8. Approximation of the Excited State Reduction Potential of the Boryl Radical

The excited state reduction potential ($E_{1/2}$*) of the dtbbpy-Bcat radical **3a** was approximated using the following equation for oxidative quenching:[35,36]

$$E_{1/2}[\mathbf{8a}/\mathbf{3a}^*] = E_{1/2}[\mathbf{8a}/\mathbf{3a}] - E_{0,0}[\mathbf{3a}^*/\mathbf{3a}]$$

Where $E_{1/2}$[**8a**/**3a**] is the ground state reduction potential and $E_{0,0}$ is the zero-zero excitation energy.

$E_{1/2}$[**8a**/**3a**] was measured by cyclic voltammetry to be –1.25 V vs. Fc/Fc^+ in DMF (Figure S15).

$E_{0,0}$[**3a***/**3a**] was estimated by using the intersection of the normalized absorbance and emission spectra of **3a**,[37] which was determined to be 478 nm (Figure S18). This corresponds to an $E_{0,0}$ value of 2.59 eV.

Therefore,

$$E_{1/2}[\mathbf{8a}/\mathbf{3a^*}] = -1.25\ \text{V} - 2.59\ \text{V}$$

$$E_{1/2}[\mathbf{8a}/\mathbf{3a^*}] = -3.84\ \text{V vs. Fc/Fc}^+\ (-3.46\ \text{V vs. SCE})\ \text{in DMF}$$

3.9. NMR study of the reaction of boronium 8a with DBU and K_2CO_3

We hypothesized that during the photocatalytic cycle, boryl radical **3** could either be regenerated by single-electron reduction of boronium ion **8**•Br (path A) or by base-mediated formation of bipyridine **2**, followed by further reaction with B_2cat_2 (path B) (Scheme S2). To investigate the possibility of path B occurring, we studied the reaction of boronium ion **8a**•PF_6 with both K_2CO_3 and DBU by ^{1}H NMR. The results showed that **8a**•PF_6 does not react with K_2CO_3 (Figure S19) and there is a slow conversion to **2a** with DBU (Figure S20). This suggests that path B is not operating when K_2CO_3 is used as the base. While path B is likely occurring when DBU is used as the base, given the relatively slow rate of conversion to **2a** observed, we believe that the predominant pathway for regeneration of **3** is via single-electron reduction of **8** (path A).

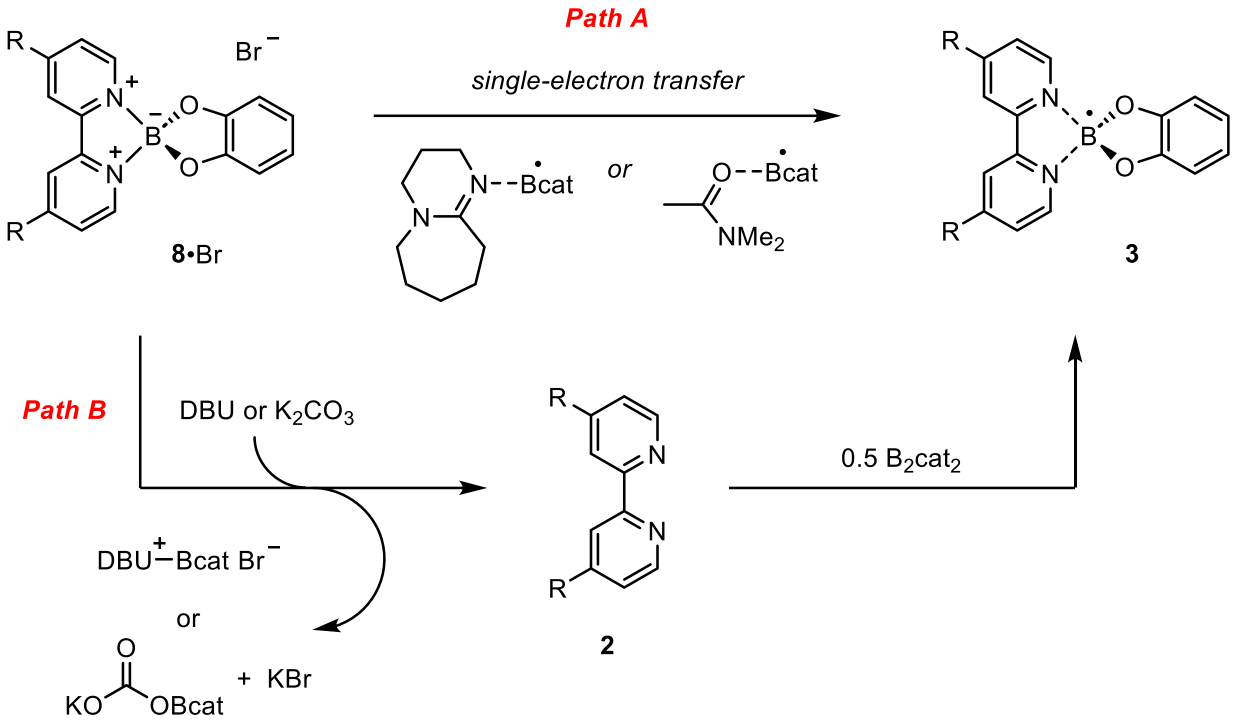

Scheme S2. Proposed pathways for regenerating the boryl radical catalyst

Sample preparation:

Sample 1: [dtbbpyBcat]PF_6 (**8a**•PF_6, 1.0 equiv., 0.005 mmol) and K_2CO_3 (4.0 equiv.) were mixed in DMF-d_7 and the ^{1}H NMR was recorded after 3 h at RT (Figure S19).

Sample 2: [dtbbpyBcat]PF_6 (**8a**•PF_6, 1.0 equiv., 0.005 mmol) and DBU (4.0 equiv.) were mixed in DMF-d_7 and the ^{1}H NMR was recorded after 3 h at RT (Figure S20).

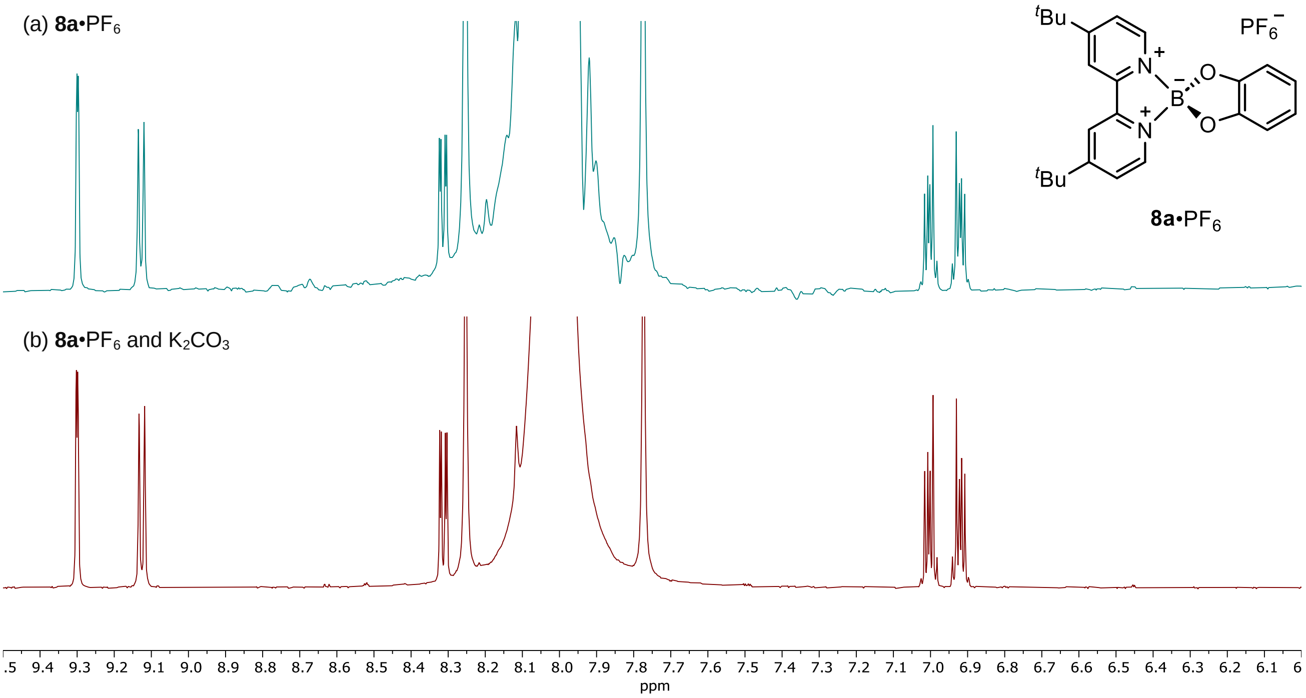

Figure S19. ^{1}H NMR of (dtbbpyBcat)PF_6 and K_2CO_3 in DMF-d_7

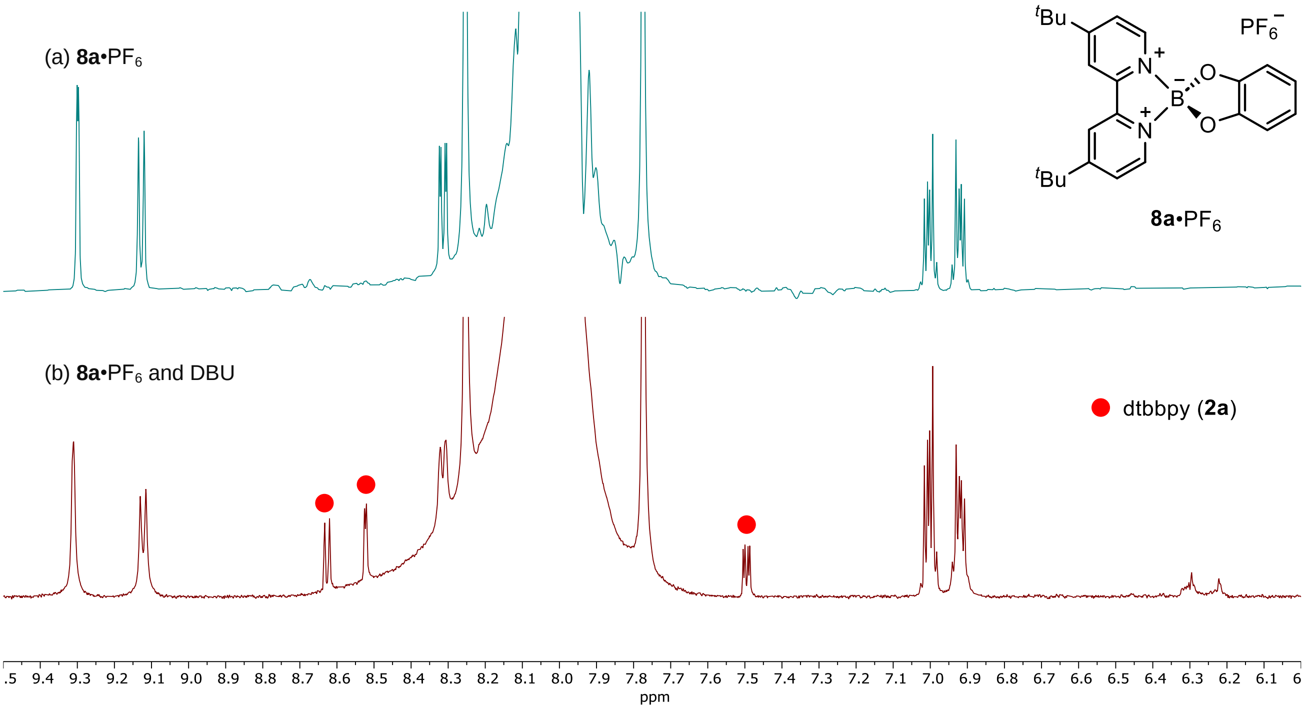

Figure S20. ^{1}H NMR study of (dtbbpyBcat)PF_6 and DBU in DMF-d_7

3.10. Quantum Yield Measurement

Determination of the Photon Flux:

The photon flux of the LED setup was determined using standard ferrioxalate actinometry[38] following a modified literature procedure.[39]

A 0.018 M ferrioxalate solution was prepared by dissolving 178 mg of potassium ferrioxalate trihydrate and 84 µL of H_2SO_4 (95–98%) in 20 mL of water. This solution was stored in an amber bottle in the dark. A buffer solution was prepared by dissolving 2.5 g of sodium acetate and 0.50 mL of H_2SO_4 (95–98%) in 50 mL of water.

While being careful to minimise exposure to background light, 0.50 mL of the 0.018 M ferrioxalate solution was added to a 7.5 mL vial. The vial was positioned in a Penn PhD Photoreactor M2 (λ_{max} = 450 nm, see Figure S1) and irradiated for between 1 and 3 s. Immediately after irradiation, 100 µL of the solution was transferred to a foil-covered 10 mL volumetric flask containing 15 mg of 1,10-phenanthroline dissolved in 3.0 mL of the buffer solution. Water was then added to the flask to make a total volume of 10 mL. The flask was shaken to ensure efficient mixing, and the solution was stored in the dark for approximately 20 min. 2.0 mL of the solution was transferred to a quartz cuvette (1.0 mL path length) and the absorbance at λ = 510 nm was measured by UV/Vis spectroscopy (Figure S21). This process was repeated for 1 s, 2 s, 3 s, and the absorbance of a non-irradiated sample was also measured.

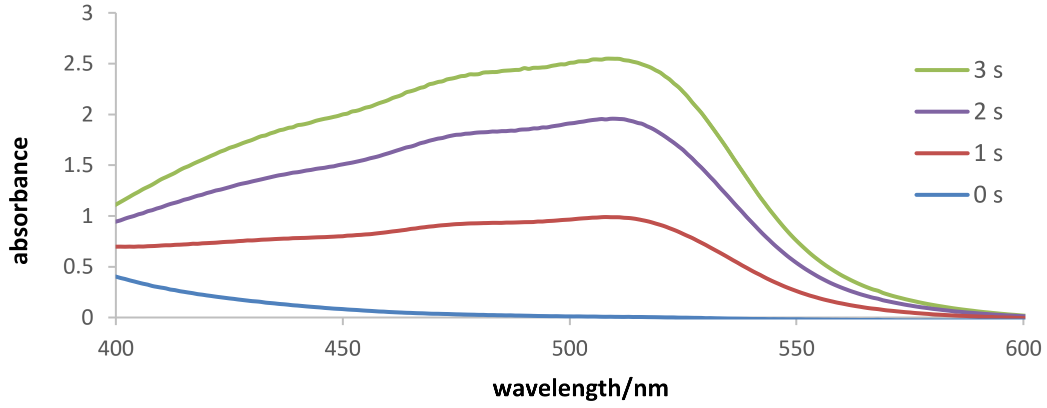

Figure S21. Actinometry: UV/Vis spectra of ferrioxalate/1,10-phenanthroline solutions Showing the change in absorbance at 510 nm after irradiation with 450 nm light for 0–3 s.

The number of moles of Fe^{2+} formed was calculated using:

$$\text{mol Fe}^{2+} = \frac{V_1 V_3 \Delta A(510\text{ nm})}{V_2 l \varepsilon(510\text{ nm})}$$

Where V_1 is the volume of ferrioxalate solution irradiated (5.0×10^{-4} L), V_2 is the volume of the aliquot taken for measurement of the concentration of Fe^{2+} ions (1.0×10^{-4} L), V_3 is the final volume after complexation with 1,10-phenanthroline (1.0×10^{-2} L), ΔA(510 nm) is the difference in absorbance at λ = 510 nm between the irradiated and non-irradiated ferrioxalate/1,10-phenanthroline solutions, l is the optical path length of the irradiation cell (1.0 cm), and ε(510 nm) is the molar absorptivity of the $Fe(phen)_3^{2+}$ complex at λ = 510 nm (11,100 L mol^{-1} cm^{-1}).

The moles of Fe^{2+} were plotted as a function of time (Fig. S22):

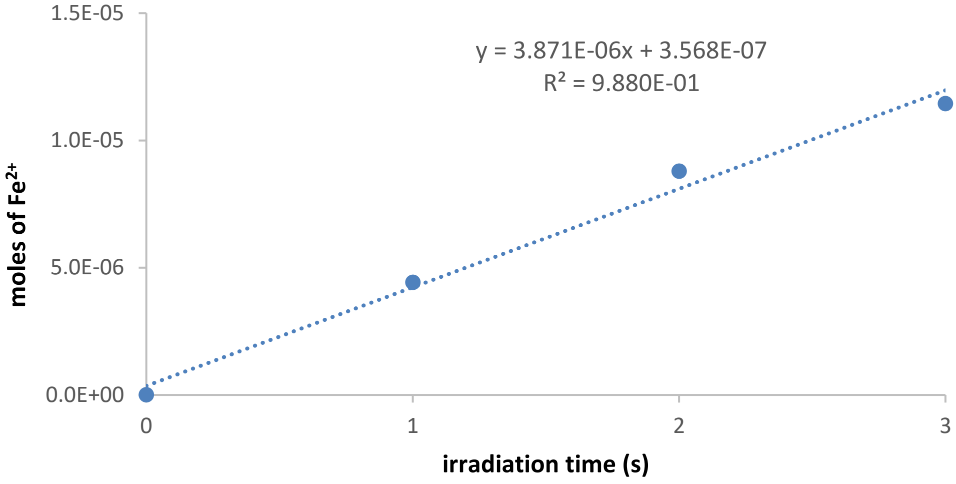

Figure S22. Actinometry: Moles of Fe^{2+} formed vs. irradiation time

The photon flux was then calculated using:

$$\text{photon flux} = \frac{\text{mol Fe}^{2+}}{\Phi t f}$$

Where Φ is the quantum yield of the ferrioxalate actinometer (1.10 at λ = 458 nm),[38] t is the time, and f is the fraction of absorbed light at λ = 450 nm, where $f = 1 - 10^{-A}$. The absorbance (A) of the 0.018 M ferrioxalate solution at λ = 450 nm was measured in a quartz cuvette (path length 1 cm) by UV/Vis spectroscopy to be 0.349, therefore f = 0.552.

$$\text{photon flux} = \frac{3.87 \times 10^{-6}}{1.10 \times 0.552} = \ 6.38 \times 10^{-6}\ \text{einstein s}^{-1}$$

Determination of the Quantum Yield:

The quantum yield was measured for the synthesis of boronic ester **7a** by borylation of **6a** using dtbbpy (**2a**) as the catalyst.

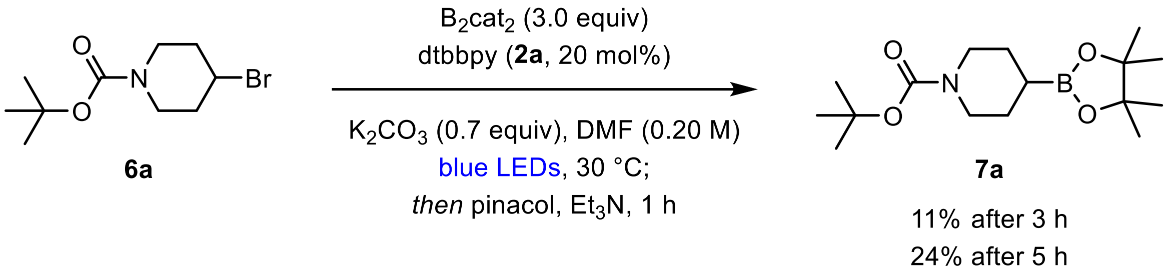

tert-Butyl 4-bromopiperidine-1-carboxylate (**6a**) (26 mg, 0.10 mmol, 1.0 equiv), B_2cat_2 (71 mg, 0.30 mmol, 3.0 equiv), dtbbpy catalyst **2a** (5.4 mg, 0.020 mmol, 20 mol%) and K_2CO_3 (9.7 mg, 0.070 mmol, 0.70 equiv) were added into an oven-dried 7 mL vial containing a small magnetic stirrer bar. The vial was sealed with a septum and anhydrous DMF (0.5 mL, 0.20 M) was added. The headspace of the vial was purged with a gentle stream of N_2 for approximately 1 min. The vial was tightly sealed with parafilm, then placed in the Penn PhD Photoreactor M2 before stirring under irradiation for 3 h (see Figure S1 for experimental setup). The vial was removed from the photoreactor, a solution of pinacol (71 mg, 0.60 mmol, 6.0 equiv) in Et_3N (0.15 mL) was added to the reaction and it was stirred at RT for another 1 h. Water (10 mL) was added and the reaction mixture was extracted with EtOAc (30 + 15 mL). The combined organic extracts were washed with water (20 mL) and brine (30 mL), dried over $MgSO_4$, filtered, and concentrated under reduced pressure. The yield of boronic ester **7a** was determined by GC analysis, using mesitylene as an internal standard, to be 11% (1.1×10^{-5} mol).

The quantum yield (Φ) was then calculated using:

$$\Phi = \frac{\text{mol product}}{\text{photon flux} \bullet t \bullet f}$$

Where t is the time (10800 s) and f is the fraction of light absorbed by the reaction mixture at λ = 450 nm, where $f = 1 - 10^{-A}$ (the absorbance of the reaction mixture (A) at λ = 450 nm was measured in a quartz cuvette (path length 1 cm) by UV/Vis spectroscopy be 3.90, thus f = 1.00).

$$\Phi = \frac{1.1 \times 10^{-5}}{6.38 \times 10^{-6} \bullet 10800 \bullet 1.00} = 1.59 \times 10^{-4}$$

The reaction was repeated with an irradiation time of 5 h (18000 s) to give 24% (2.4×10^{-5} mol) yield.

$$\Phi = \frac{2.4 \times 10^{-5}}{6.38 \times 10^{-6} \bullet 18000 \bullet 1.00} = 2.09 \times 10^{-4}$$

We believe the low quantum yield results from the short lifetime of the excited state boryl radical and the inefficient (18%) generated of the longer-lived (1.2 ns) excited state species (see Section 3.12).

3.11. Time Correlated Single Photon Counting

Sample preparation: In glovebox, to a 7.0 mL vial equipped with a magnetic stir bar was added 4,4'-di-*tert*-butyl-2,2'-bipyridine (**2a**, 10.8 mg, 0.0402 mmol) and B_2cat_2 (9.5 mg, 0.040 mmol, 1.0 equiv). Anhydrous DMF (4.0 mL) was added, and the mixture was allowed to stir at room temperature for 1 h. Subsequently, 100 µL of this mixture was transferred to cavity microscope slide and sealed with a cover slip, which was held in place using silicone grease.

Equipment Details: Fluorescence lifetime images were acquired using a Leica SP8 CLSM system attached to a DMi8 inverted microscope (*Leica Microsystems*). Excitation was provided by the 488 nm line from a pulsed white light laser with a repetition rate of 80 MHz. Images were acquired using a 10x/0.3NA objective, with fluorescence collected within the 495-600 nm window, a notch filter centred at 488 nm minimised any laser scatter into the detector. Time resolved data was acquired using a PicoHarp 300 time correlated single photon counting (TCSPC) module (*PicoQuant*) controlled through SymPhoTime software (*PicoQuant)*. Fluorescence-lifetime imaging microscopy (FLIM) images were acquired with 128 x 128 pixels, 4096 time bins and at a scan speed of 10 Hz. Total integration time was 473 s. Data analysis was carried out using the SymPhoTime fitting software tool with the pixel-wise fitting algorithm. The data were spatially binned to a single decay. The data was cropped between 500-7500 ps to exclude scattered marginal data and was fitted with a triple exponential model.

The results of the TCSPC measurement are shown in Figure S23. Three components of the fluorescence were identified: (1) 49.3 ± 0.2 ps (could result from laser scatter); (2) 388 ± 8 ps; and (3) 3.01 ± 0.05 ns.

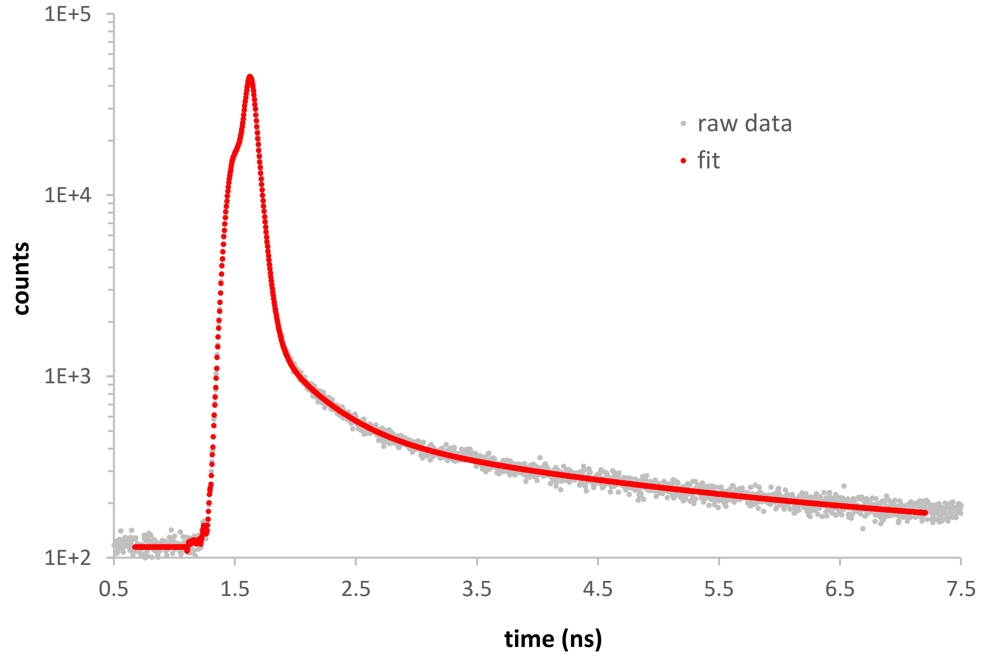

Figure S23. Fluorescence lifetime measurement using TCSPC
Red circles are the exponential fit to the raw data (grey circles)

3.12. Transient Absorption Spectroscopy

Sample preparation: In glovebox, to a 7.0 mL vial equipped with a magnetic stir bar was added 4,4'-di-*tert*-butyl-2,2'-bipyridine (**2a**, 10.8 mg, 0.0402 mmol) and B_2cat_2 (9.5 mg, 0.040 mmol, 1.0 equiv). Anhydrous DMF (4.0 mL) was added, and the mixture was allowed to stir at room temperature for 1 h. Subsequently, the mixture was transferred to a 2 mm pathlength cuvette, which was sealed with parafilm before removal from the glovebox.

Equipment Details: Transient electronic absorption (TA) spectra were recorded at the University of Bristol using an ultrafast laser setup. A detailed description of the system is available in the Supporting Information of reference 40. Briefly, femtosecond laser pulses were generated by a Coherent Astrella regenerative amplifier system (1 kHz, 800 nm, 7 W, 35 fs pulse width), seeded by a Ti:Sapphire oscillator. The pump beam at 470 nm was produced via an optical parametric amplifier (OPA, Coherent OPerA Solo), delivering approximately 490 nJ of energy at the sample. White-light continuum (WLC) probe pulses were generated by focusing the 800 nm beam into a calcium fluoride (CaF_2) plate, covering a spectral range of 350–740 nm.

The pump and probe beams were spatially and temporally overlapped at the sample position, and the transmitted probe was collected by an Andor Shamrock 163 spectrometer equipped with a 1024-element photodiode array (Entwicklungsbüro Stresing) to capture the TA spectra. The relative polarization between pump and probe was set to the magic angle (54.7°) to eliminate rotational contributions. The instrument response function (IRF) was determined to be approximately 110 fs by fitting solvent-only measurements. This setup enabled measurements over a time window from approximately 100 fs to 3.75 ns, with the time delays controlled using an optical delay stage.

The TA data were corrected for frequency chirp in the WLC and decomposed using the KOALA program.[41]

The TA spectrum is shown in Figure S24.

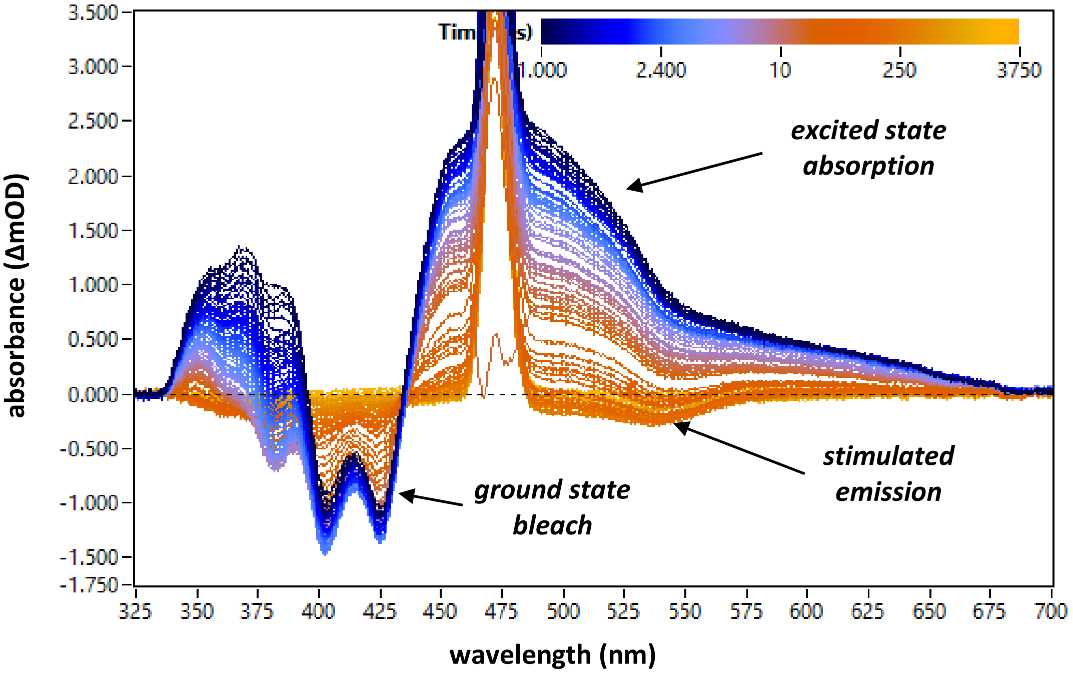

Figure S24. Transient absorption spectroscopy

Kinetic analyses of the decay of the different spectral features, including the stimulated emission (Figure S25), excited state absorption (Figure S26), and ground state bleach (Figure 27), identified two time components: one fast (10–20 ps) and one slower (~1.2 ns).

We can also conclude that the majority (~82%) of the photoexcited (at 470 nm) radicals return to the ground state with the fast time constant of 10–20 ps. The remaining ~18% return to the ground state with a ~1.2 ns time constant, and only these longer-lived radicals have sufficient time to act as photocatalysts. This inefficient generation of the longer-lived excited state species likely contributes to the low quantum yield of the reaction.

Complete recovery of the ground-state bleach features were observed within the experimental time window (0–4 ns), indicating that no other longer-lived photoexcited radicals persist beyond 4 ns.

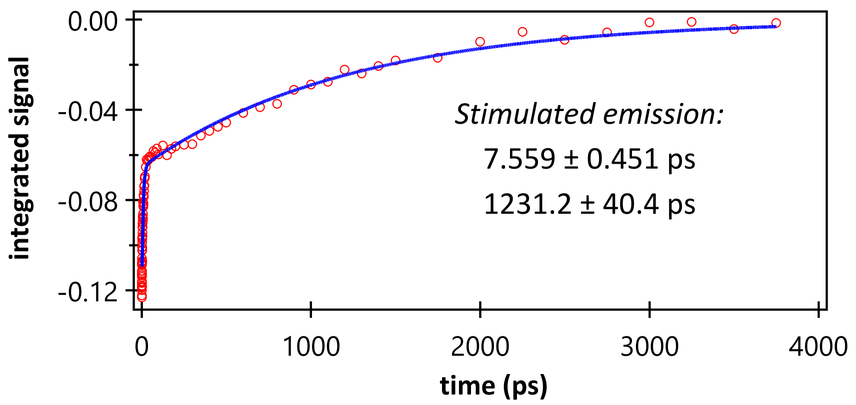

Figure S25. Kinetic analysis of the stimulated emission decay

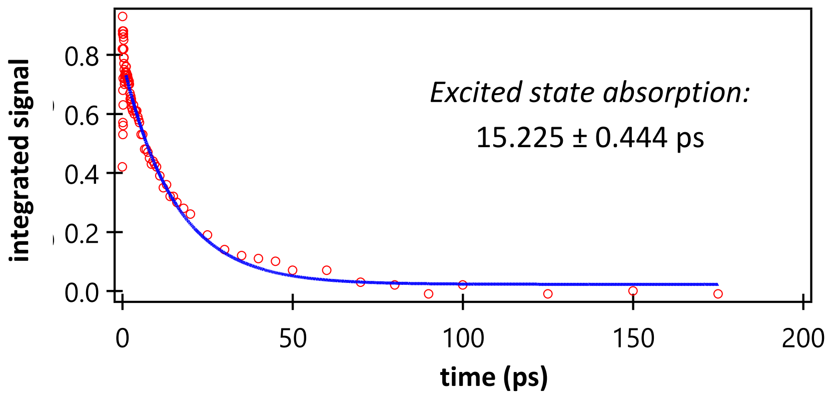

Figure S26. Kinetic analysis of the excited state absorption decay

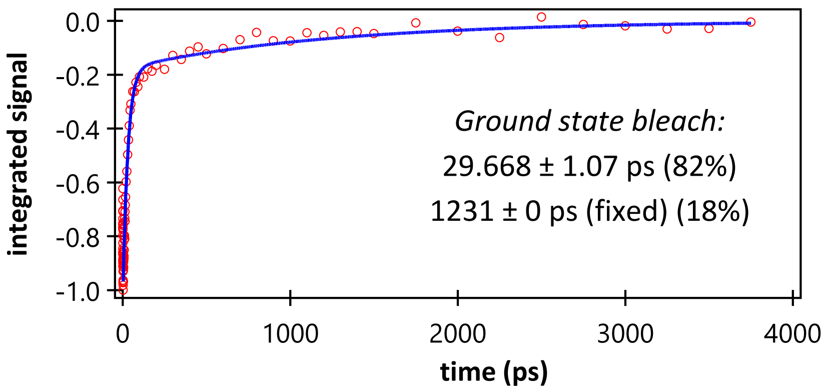

Figure S27. Kinetic analysis of the ground state bleach decay

4. X-RAY CRYSTALLOGRAPHY

X-ray diffraction experiments on **8a**•PF_6 and **8d**•PF_6 were carried out at 100(2) K on a Bruker D8 Venture diffractometer using Cu-K_α radiation (λ = 1.54178 Å). Data collections were performed using a Bruker CPAD detector. Intensities were integrated in SAINT[42] and absorption corrections based on equiv.alent reflections were applied using SADABS.[43] The structure was solved using ShelXT[44] and refined by full matrix least squares against F^{43} in ShelXL[45,46] using Olex2.[47] All of the non-hydrogen atoms were refined anisotropically. While all of the hydrogen atoms were located geometrically and refined using a riding model. The crystal structures for **8a**•PF_6 and **8d**•PF_6 are shown in Figures S28 and S29, respectively, and the refinement data are given in Table S4. Crystallographic data for compounds **8a**•PF_6 and **8d**•PF_6 has been deposited with the Cambridge Crystallographic Data Centre as supplementary publications CCDC 2126965 and CCDC 2126964, respectively. Copies of the data can be obtained free of charge on application to CCDC, 12 Union Road, Cambridge CB2 1EZ, UK [fax(+44) 1223 336033, e-mail: deposit@ccdc.cam.ac.uk].

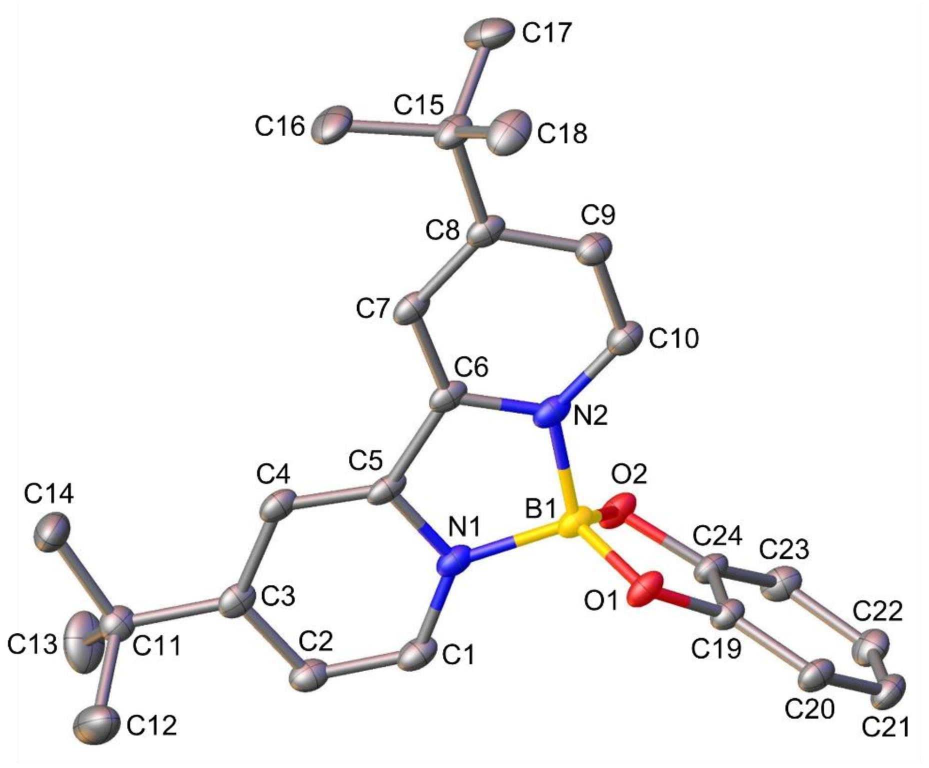

Figure S28. Crystal structure of **8a**•PF_6

The anisotropic displacement parameters are depicted at the 50% probability level. The hydrogens, PF_6 counterion and an acetone solvent molecule are omitted for clarity.

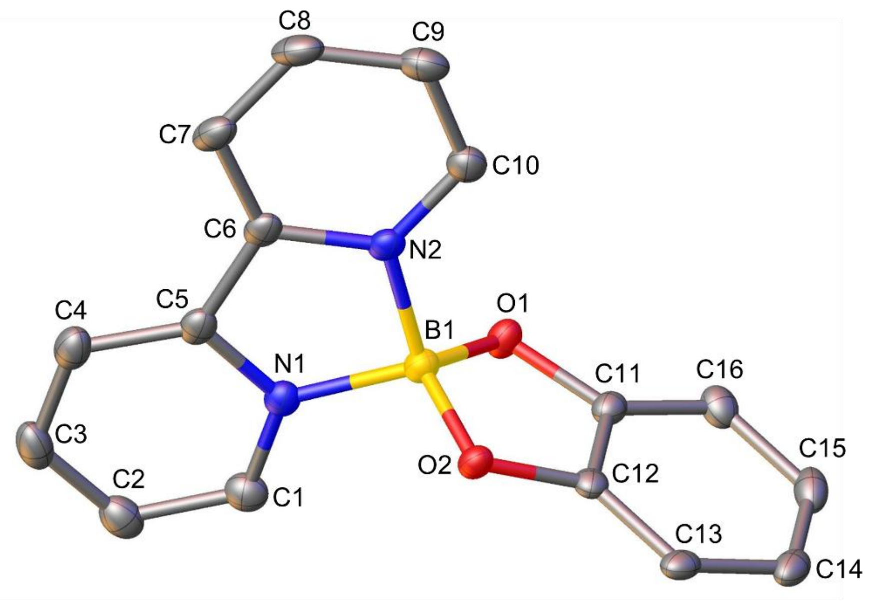

Figure S29. Crystal structure of **8d**•PF_6

The anisotropic displacement parameters are depicted at the 50% probability level. The hydrogens and PF_6 counterion are omitted for clarity.

Table S4. Crystal data and structure refinement for **8a**•PF_6 and **8d**•PF_6

Complex	**8a**•PF_6	**8d**•PF_6
CCDC number	2126965	2126964
Empirical formula	$C_{27}H_{34}BN_2O_3F_6P$	$C_{16}H_{12}BN_2O_2F_6P$
Formula weight	590.34	420.06
Temperature/K	100.0	100.0
Crystal system	monoclinic	monoclinic
Space group	$P2_1/c$	$P2_1/n$
a/Å	10.0262(3)	12.8586(4)
b/Å	27.8505(9)	8.8296(2)
c/Å	10.3457(3)	15.0072(4)
α/°	90	90
β/°	94.9740(10)	97.8680(10)
γ/°	90	90
Volume/$Å^3$	2878.00(15)	1687.82(8)
Z	4	4
$\rho_{calc}g/cm^3$	1.362	1.653
μ/mm^{-1}	1.476	2.206
F(000)	1232.0	848.0
Crystal size/mm^3	0.579 × 0.255 × 0.06	0.46 × 0.29 × 0.06
Radiation	CuKα (λ = 1.54178)	CuKα (λ = 1.54178)
2Θ range for data collection/°	6.346 to 140.134	8.5 to 136.486
Index ranges	$-12 \leq h \leq 12$<br>$-32 \leq k \leq 33$<br>$-12 \leq l \leq 12$	$-15 \leq h \leq 15$<br>$-10 \leq k \leq 10$<br>$-18 \leq l \leq 18$
Reflections collected	44332	20926
Independent reflections	5466 [R_{int} = 0.0359, R_{sigma} = 0.0183]	3084 [R_{int} = 0.0330, R_{sigma} = 0.0230]
Data/restraints/parameters	5466/0/369	3084/0/253
Goodness-of-fit on F^2	1.137	1.048
Final R indexes [I>=2σ (I)]	R_1 = 0.0636<br>wR_2 = 0.2071	R_1 = 0.0311<br>wR_2 = 0.0797
Final R indexes [all data]	R_1 = 0.0655<br>wR_2 = 0.2093	R_1 = 0.0322<br>wR_2 = 0.0806
Largest diff. peak/hole / e $Å^{-3}$	0.63/-0.96	0.21/-0.37

5. SPECTROSCOPIC DATA

^{1}H NMR (400 MHz, $CDCl_3$) of catalyst **2b** (*see procedure*)

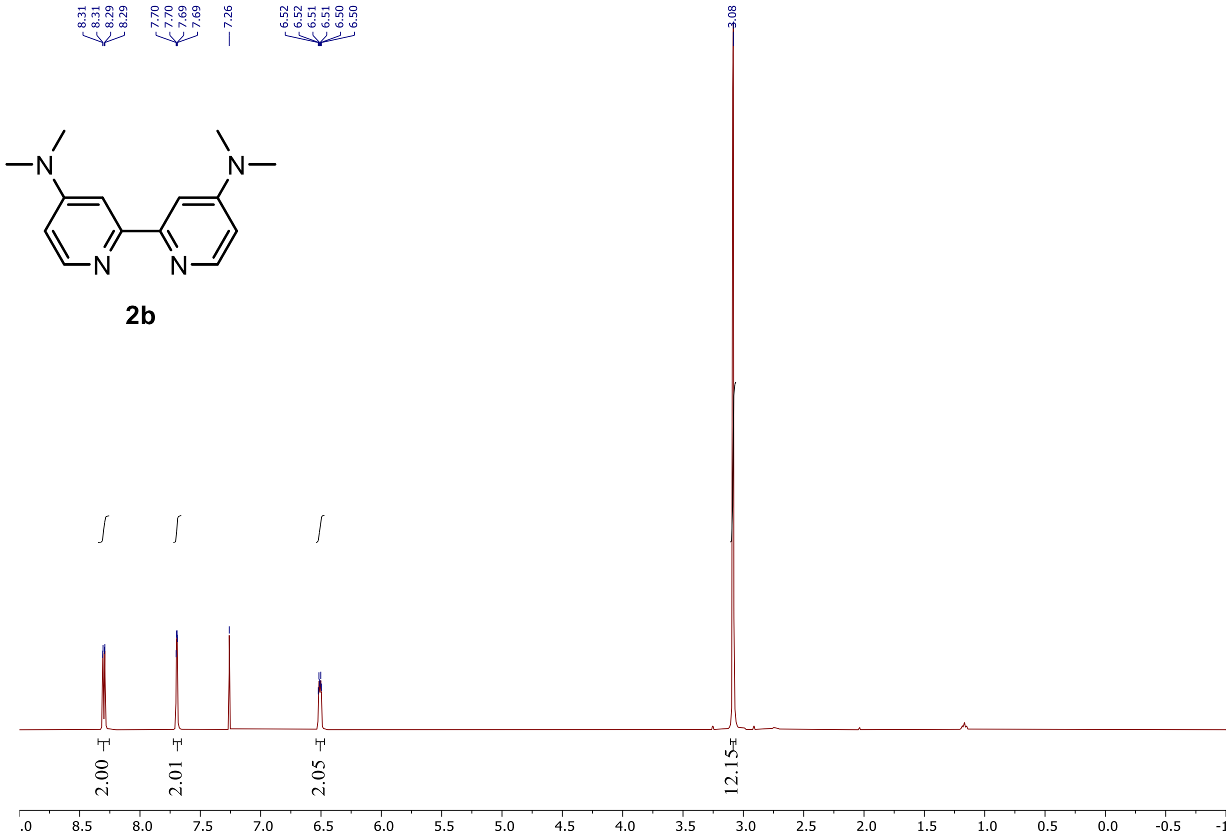

^{13}C NMR (101 MHz, $CDCl_3$) of catalyst **2b**

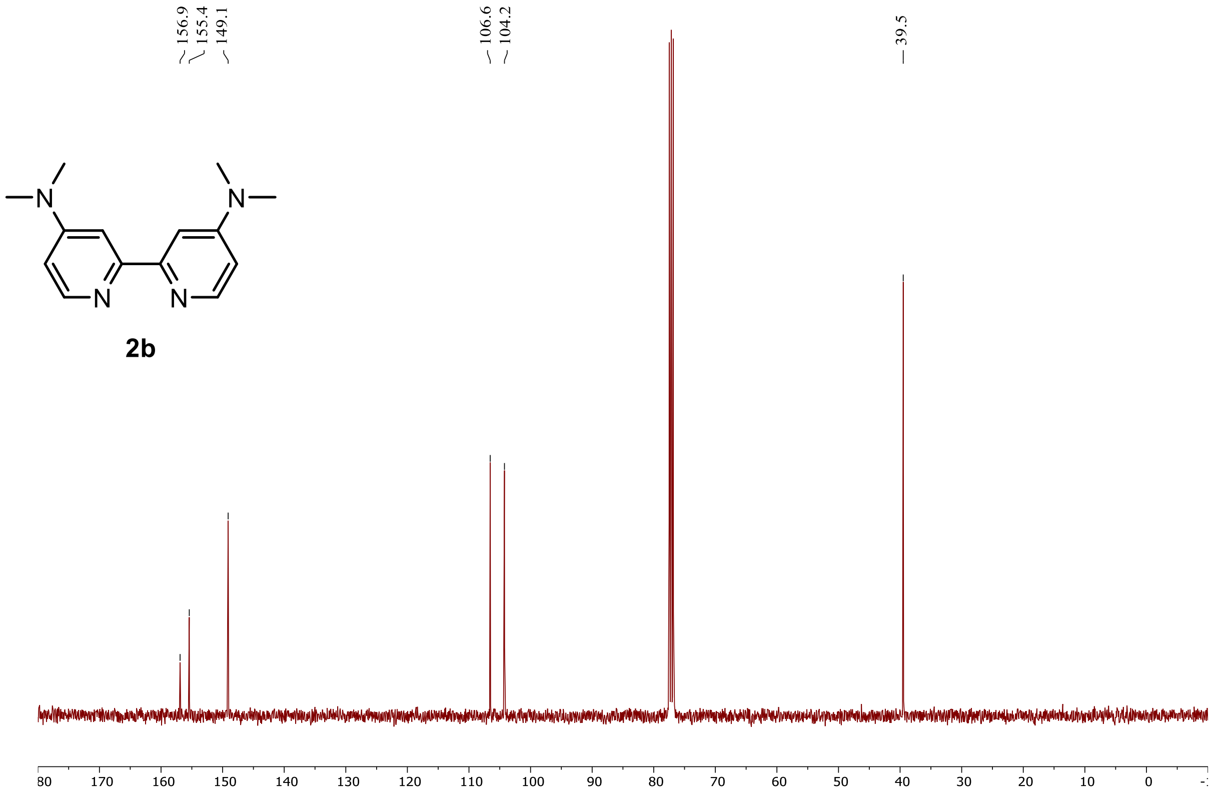

^{1}H NMR (500 MHz, DMF-d_7) of catalyst **2b** (*see procedure*)

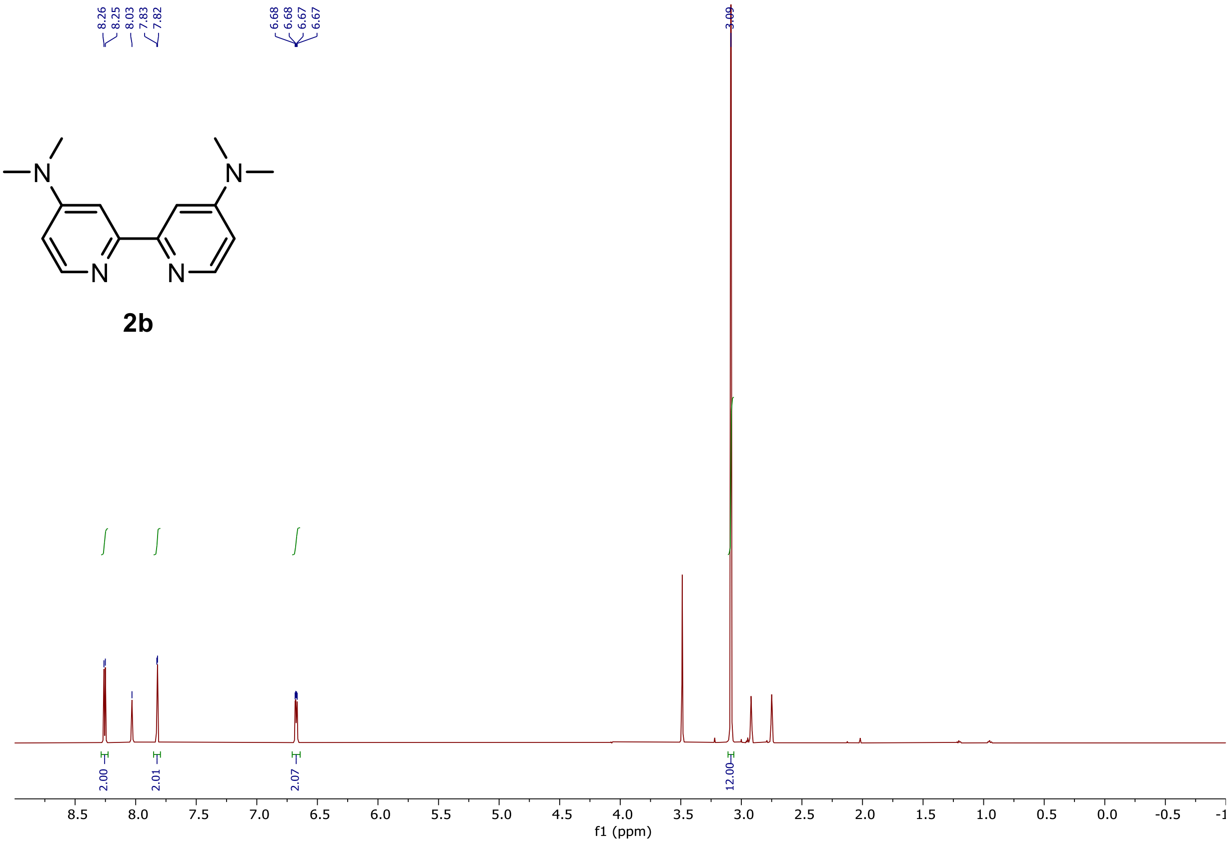

^{13}C NMR (126 MHz, DMF-d_7) of catalyst **2b**

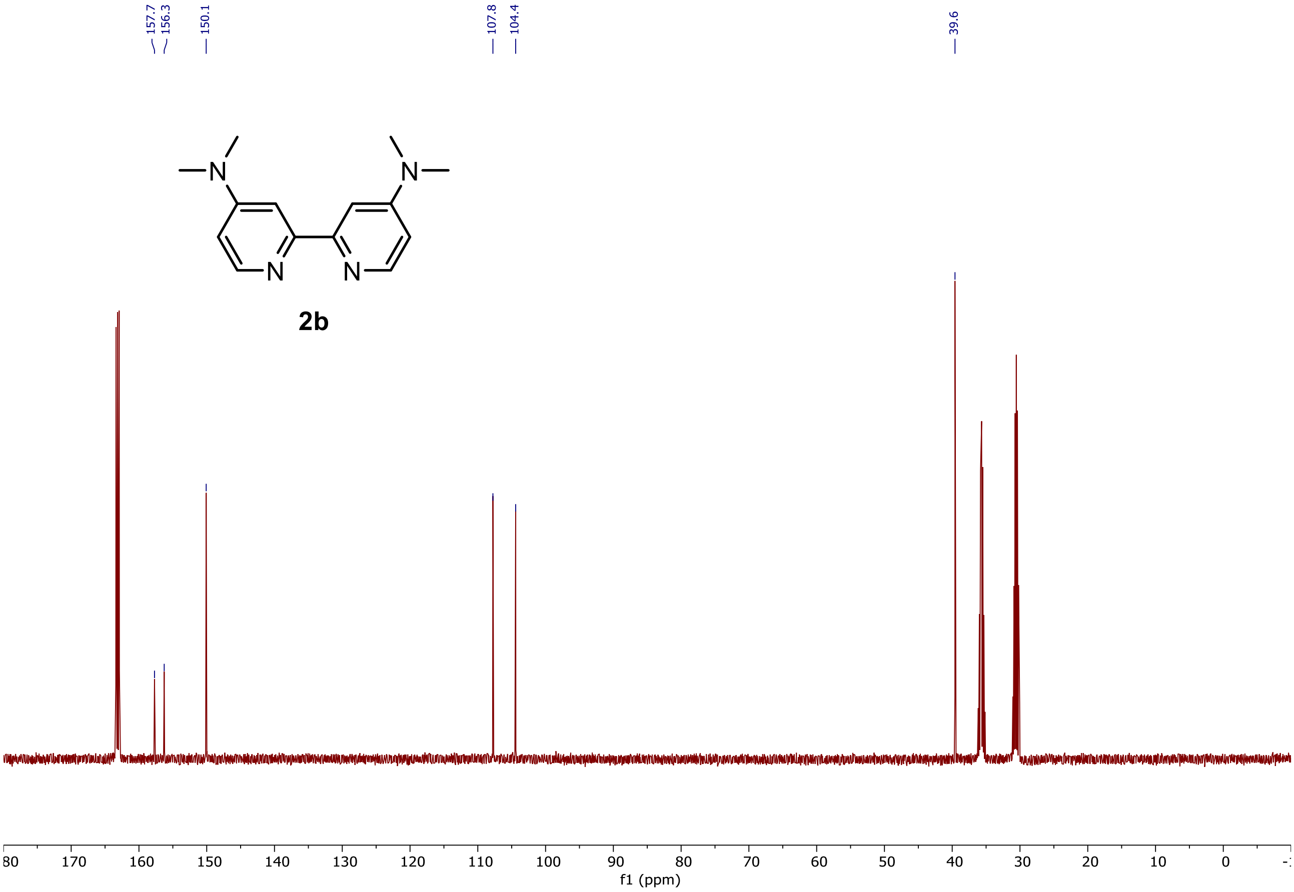

^{1}H NMR (400 MHz, $CDCl_3$) of **6f** (*see procedure*)

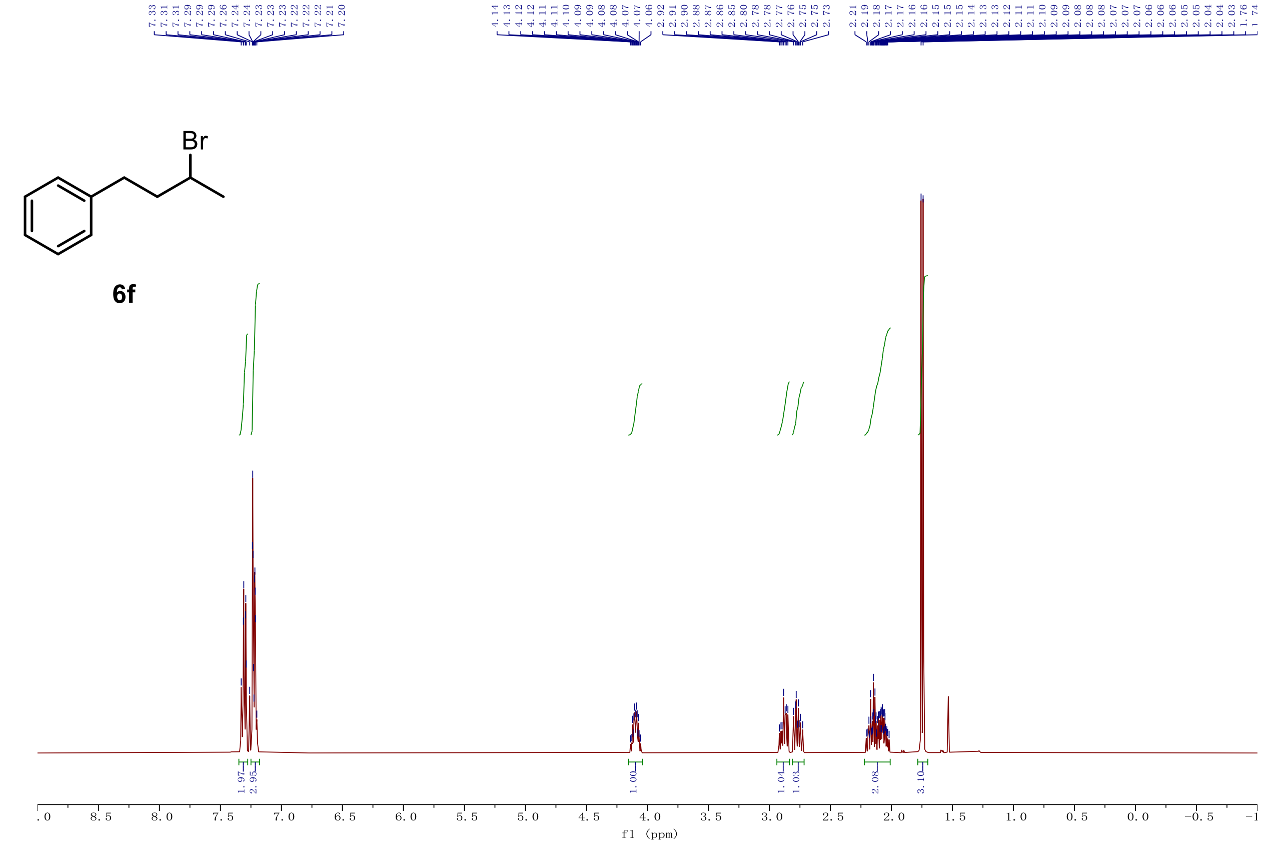

^{13}C NMR (101 MHz, $CDCl_3$) of **6f**

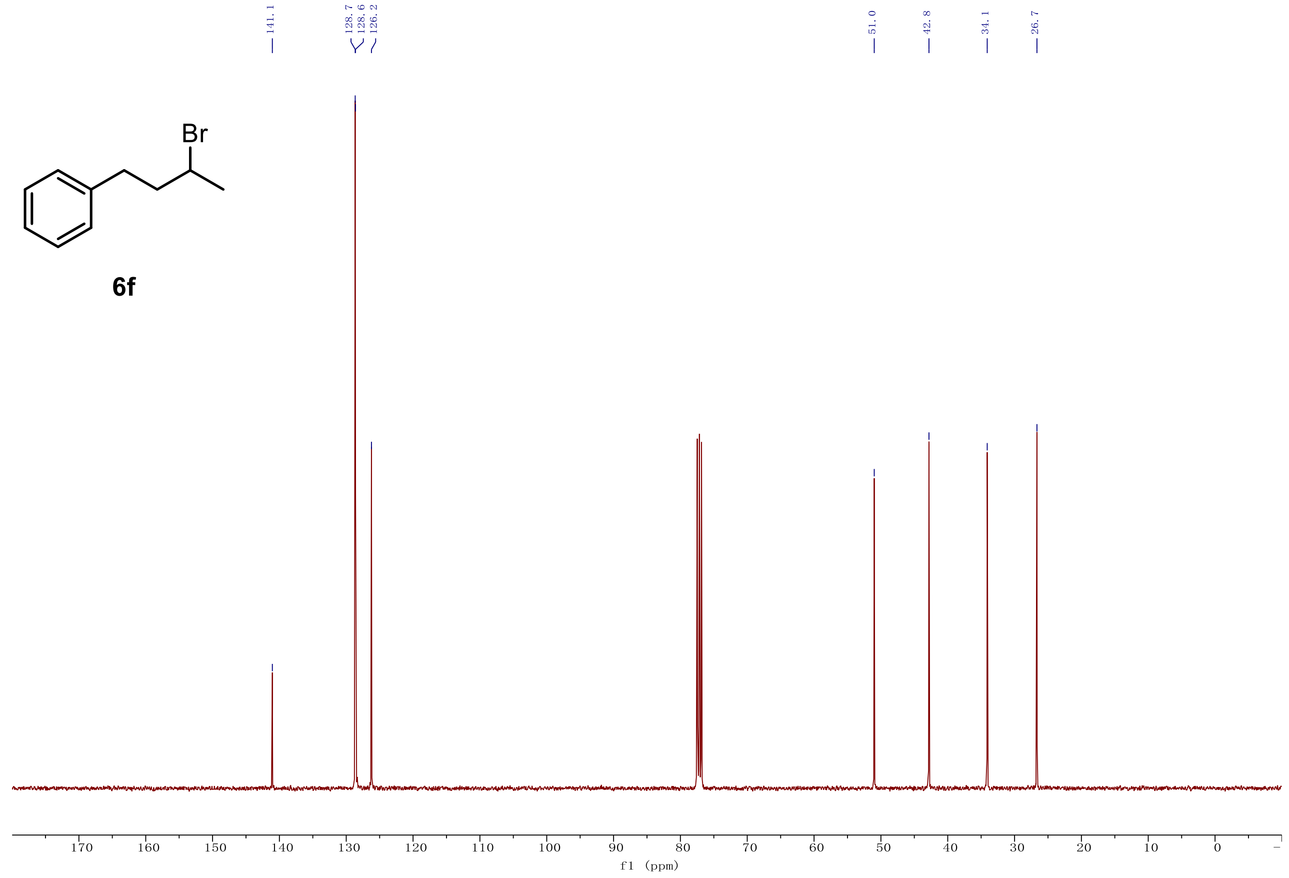

^{1}H NMR (400 MHz, $CDCl_3$) of **6n** (see procedure)

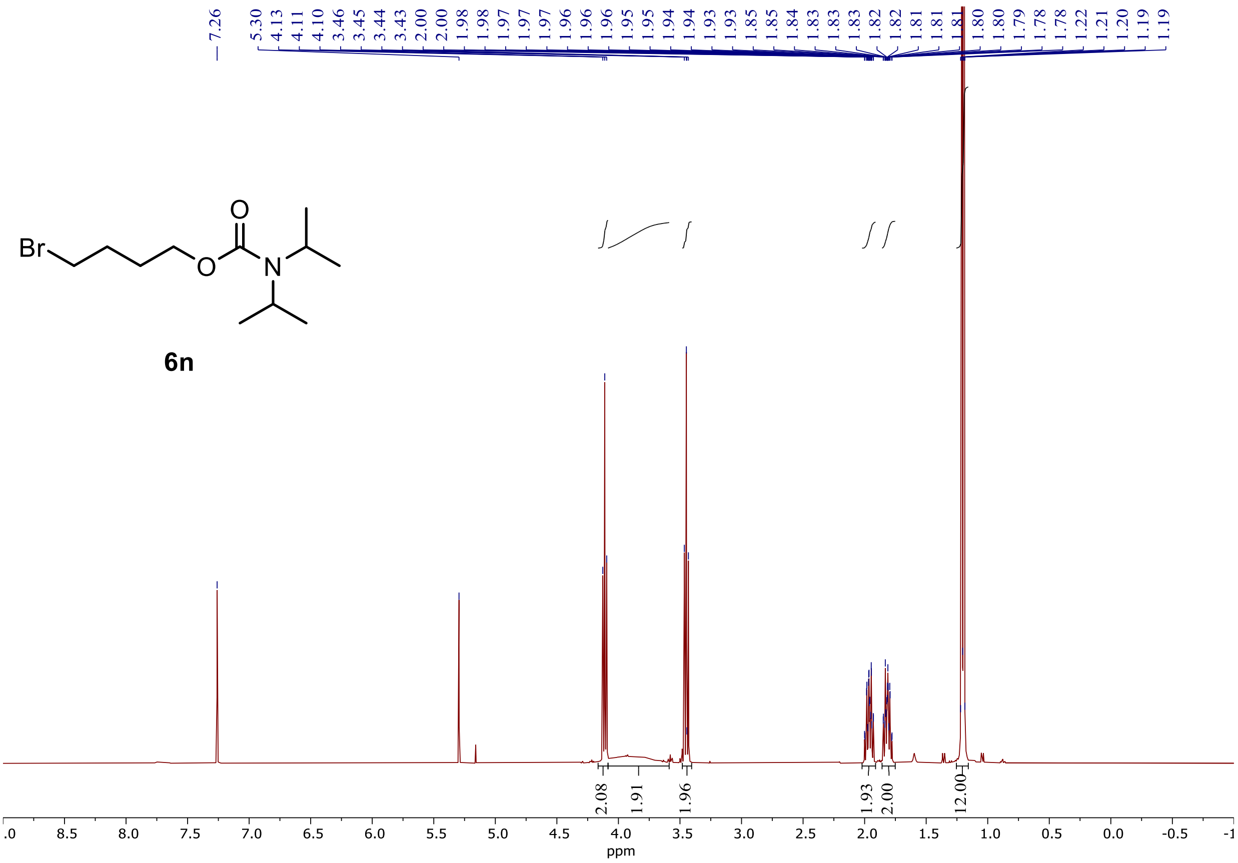

^{13}C NMR (101 MHz, $CDCl_3$) of **6n**

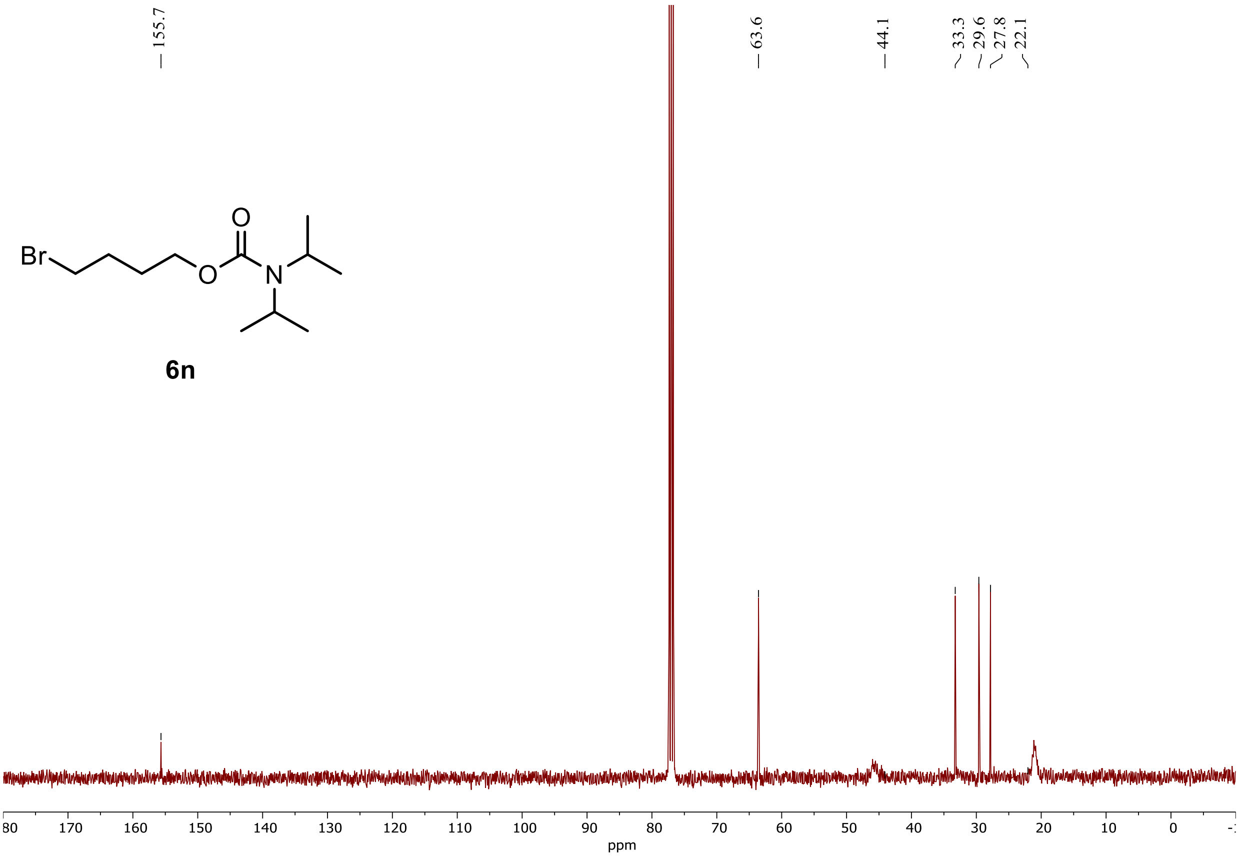

^{1}H NMR (400 MHz, $CDCl_3$) of **6o** (see procedure)

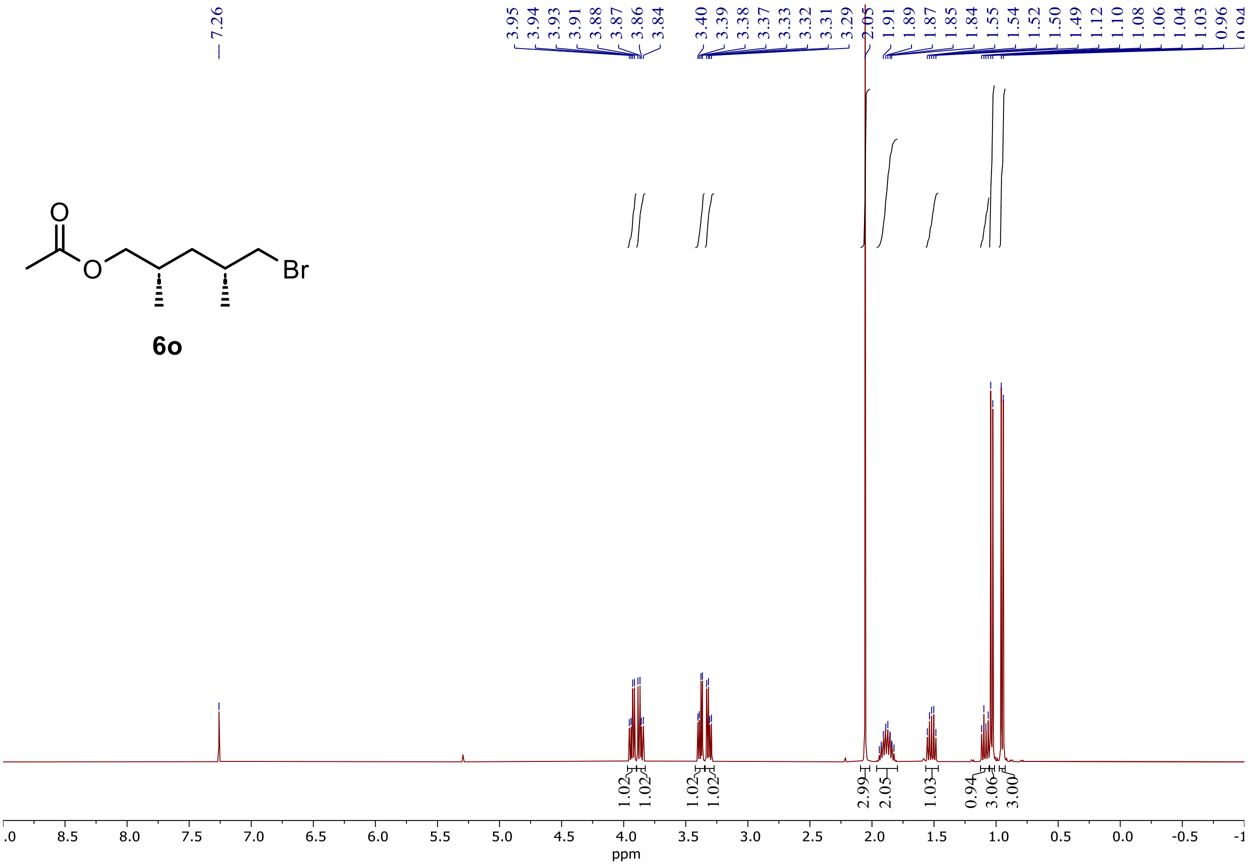

^{13}C NMR (101 MHz, $CDCl_3$) of **6o**

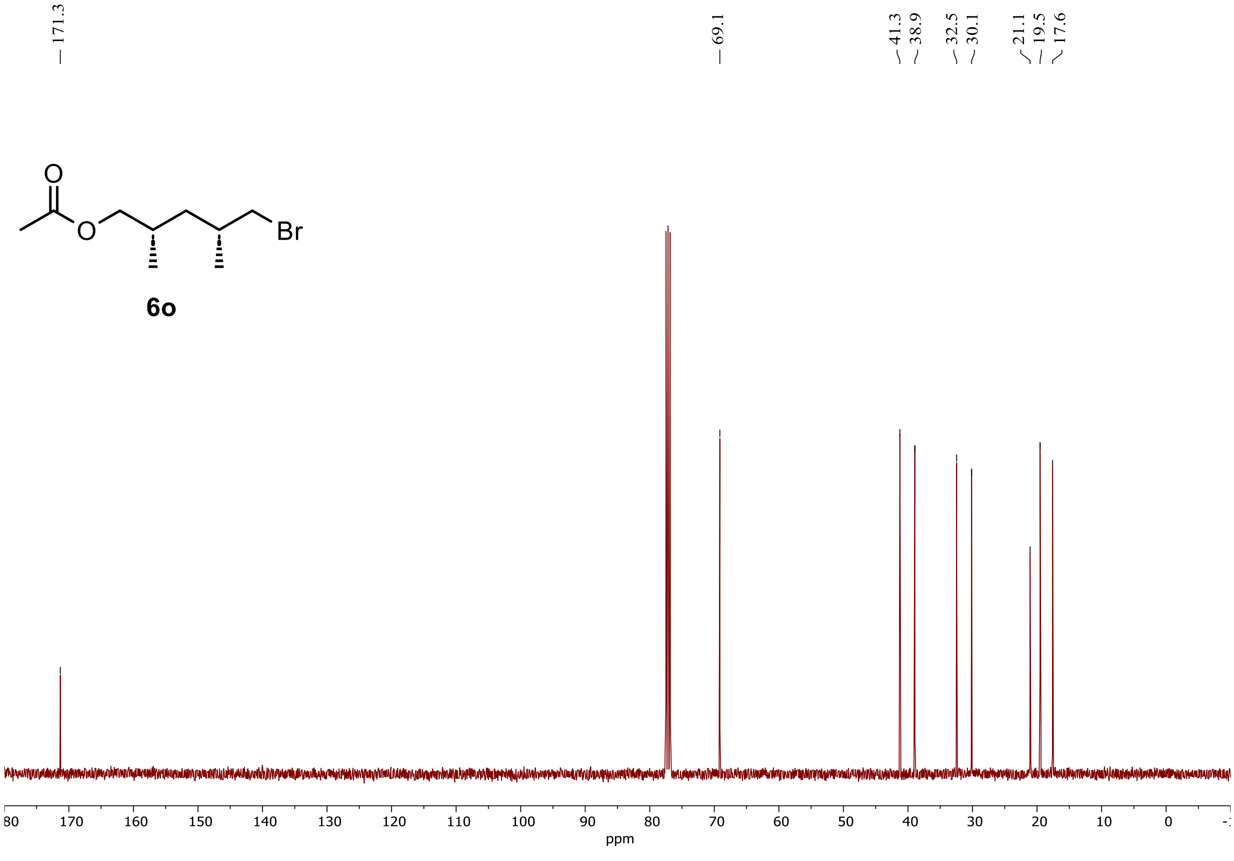

^{1}H NMR (400 MHz, CDCl3) of **6r** (*see procedure*)

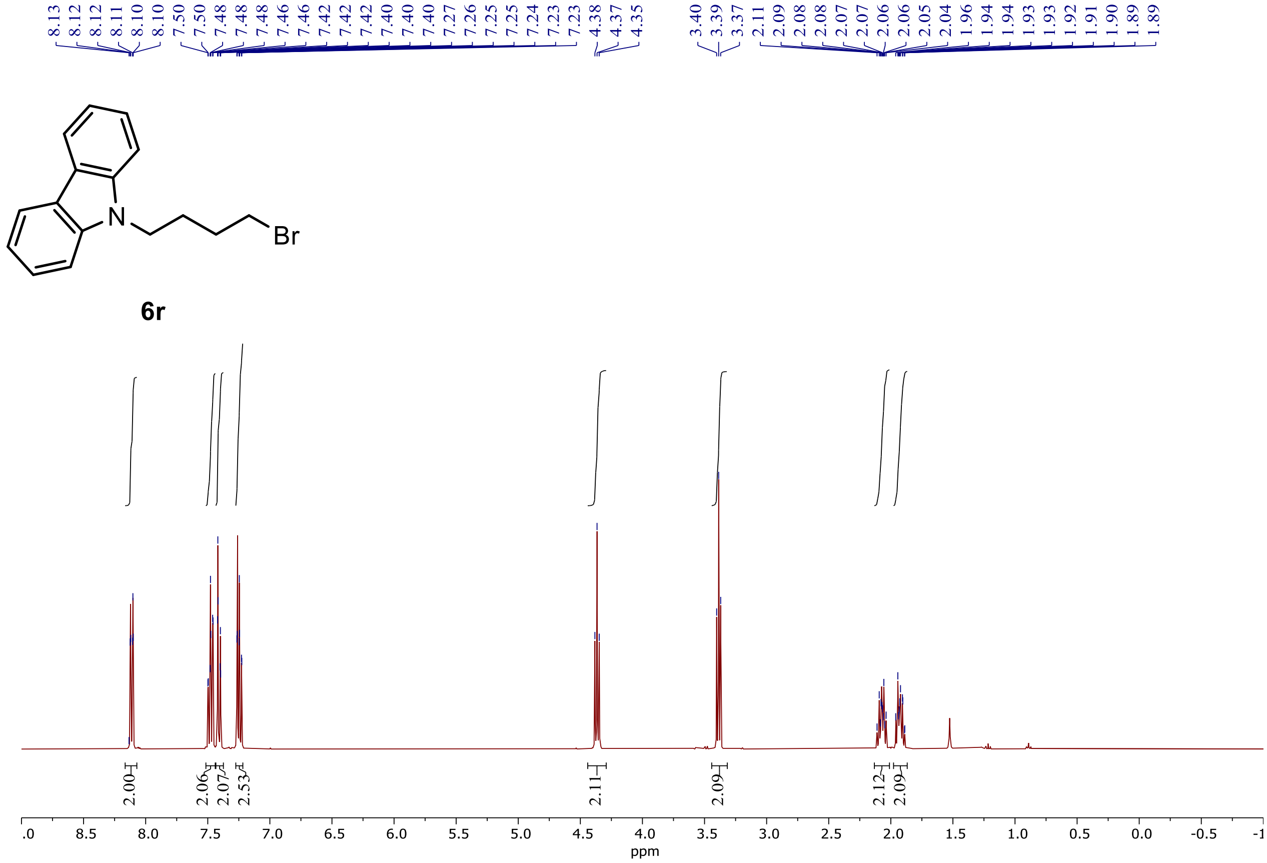

6r

^{13}C NMR (101 MHz, CDCl3) of **6r**

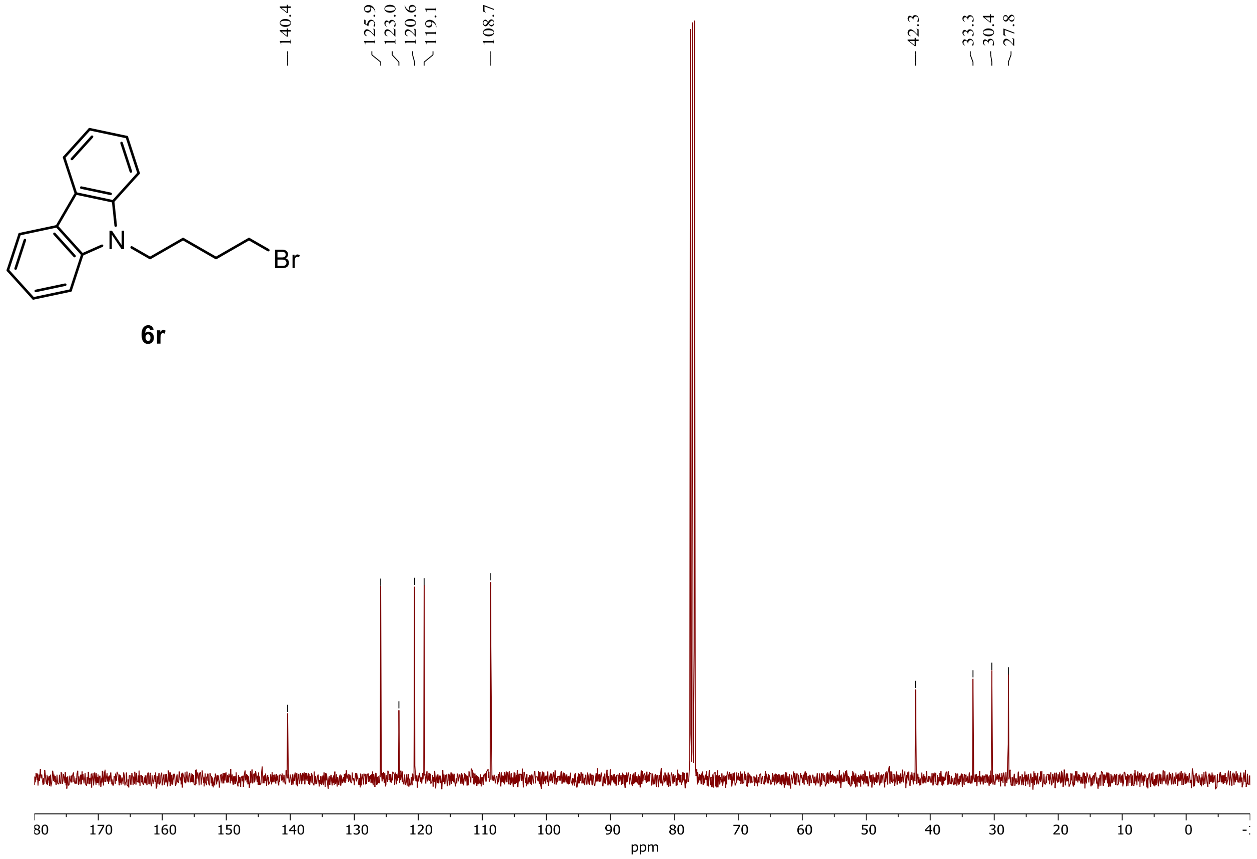

6r

^{1}H NMR (400 MHz, $CDCl_3$) of **6w** (*see procedure*)

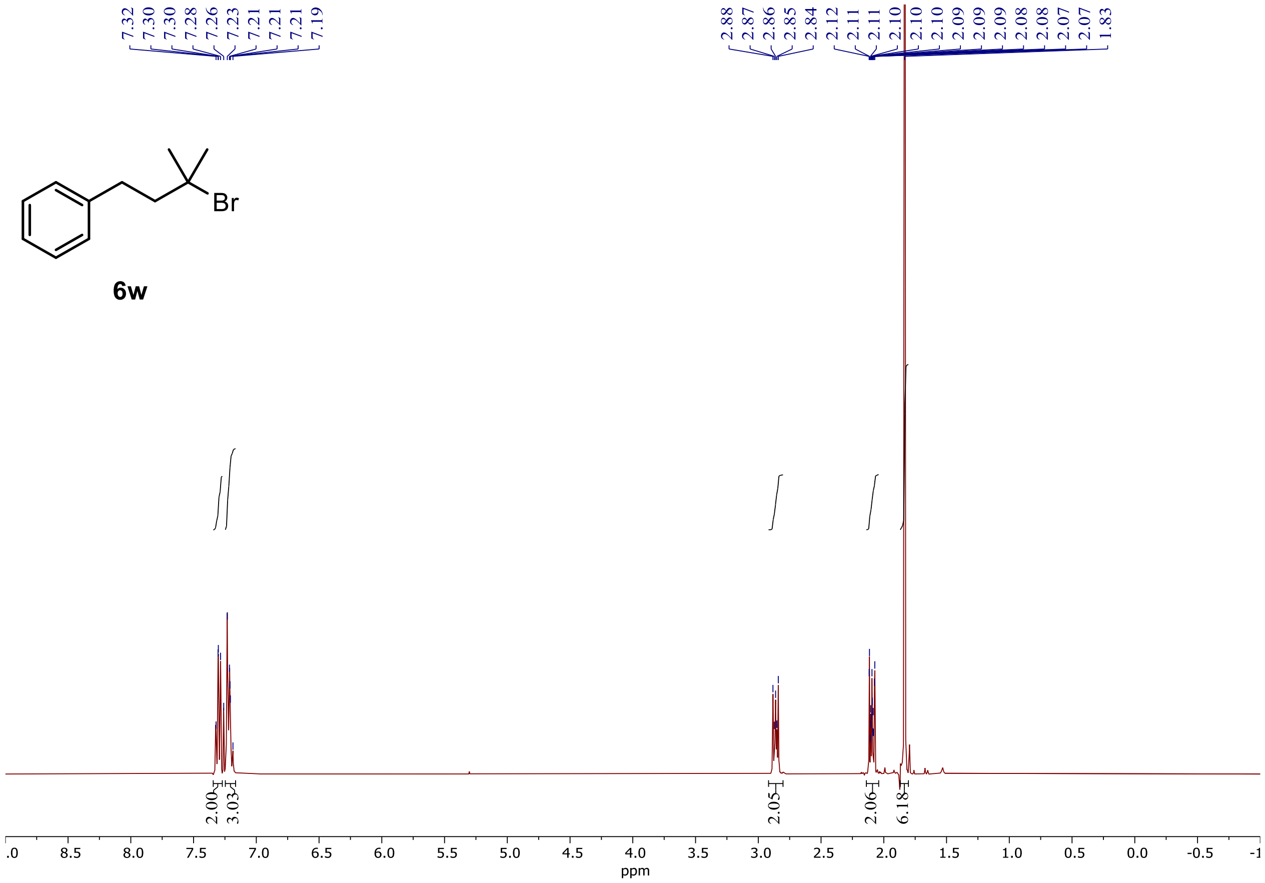

^{13}C NMR (101 MHz, $CDCl_3$) of **6w**

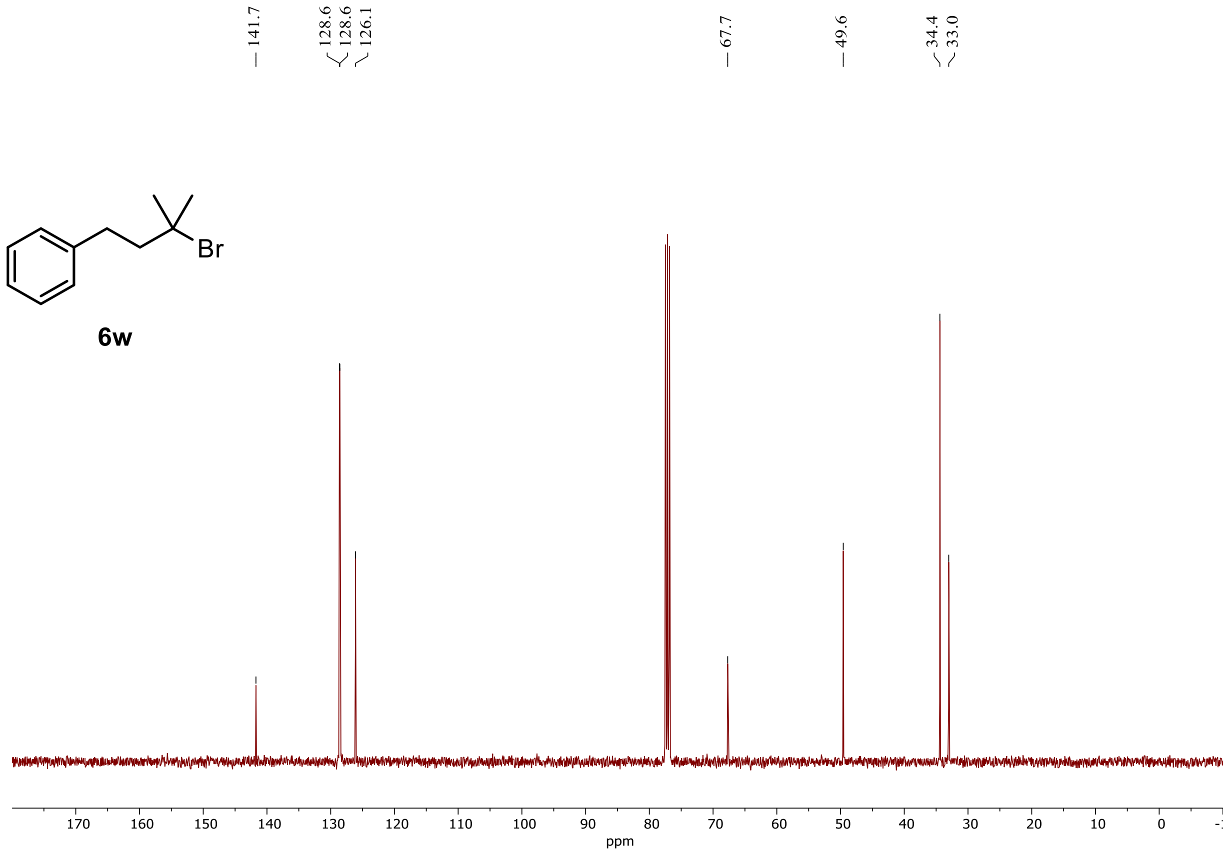

^{1}H NMR (400 MHz, $CDCl_3$) of **6x** (*see procedure*)

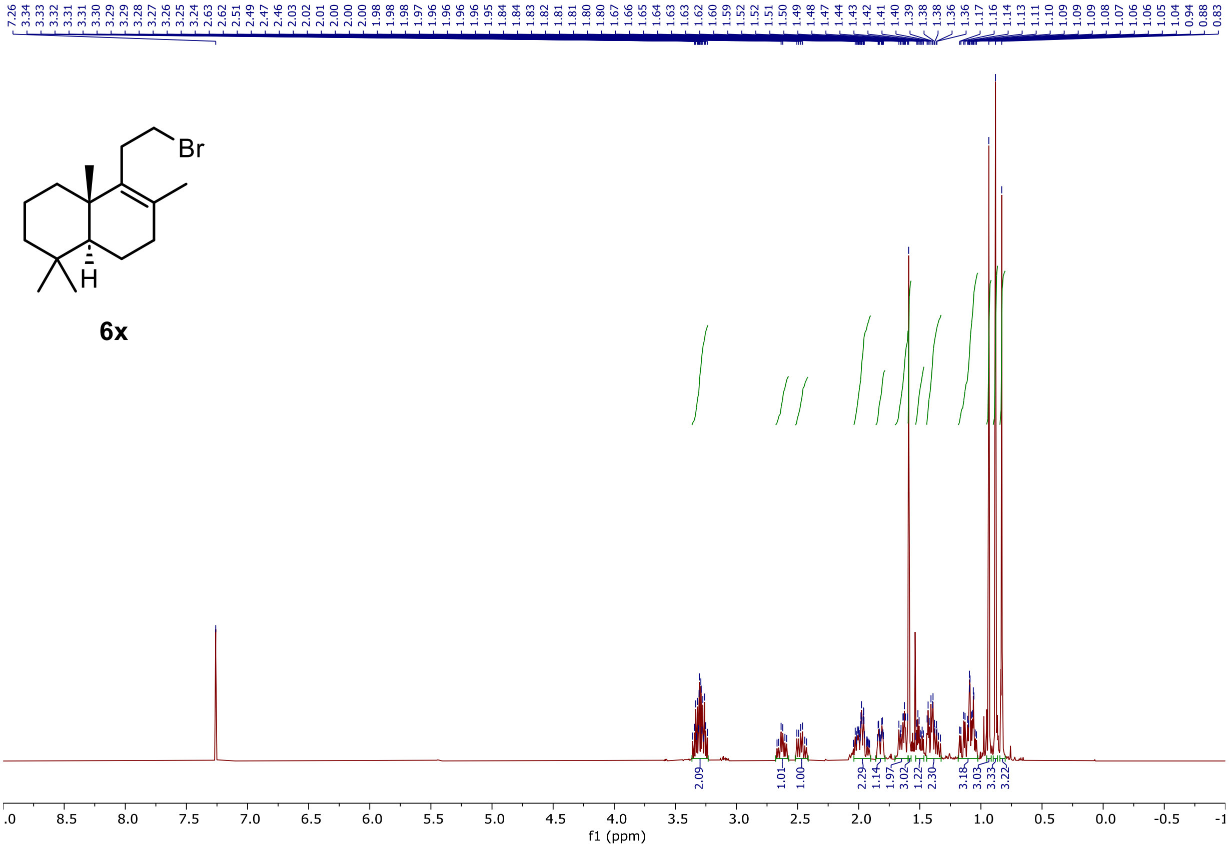

^{13}C NMR (126 MHz, $CDCl_3$) of **6x**

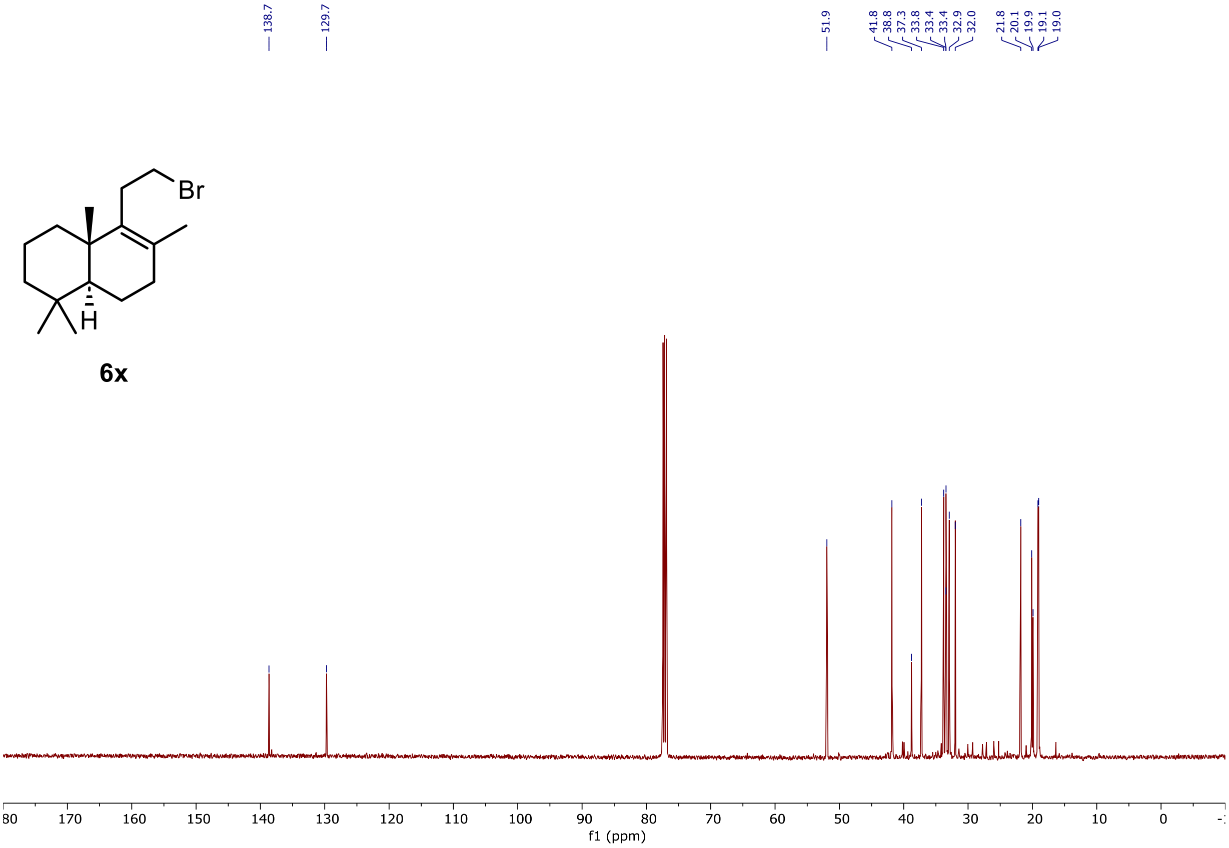

^{1}H NMR (400 MHz, $CDCl_3$) of **6y** (see procedure)

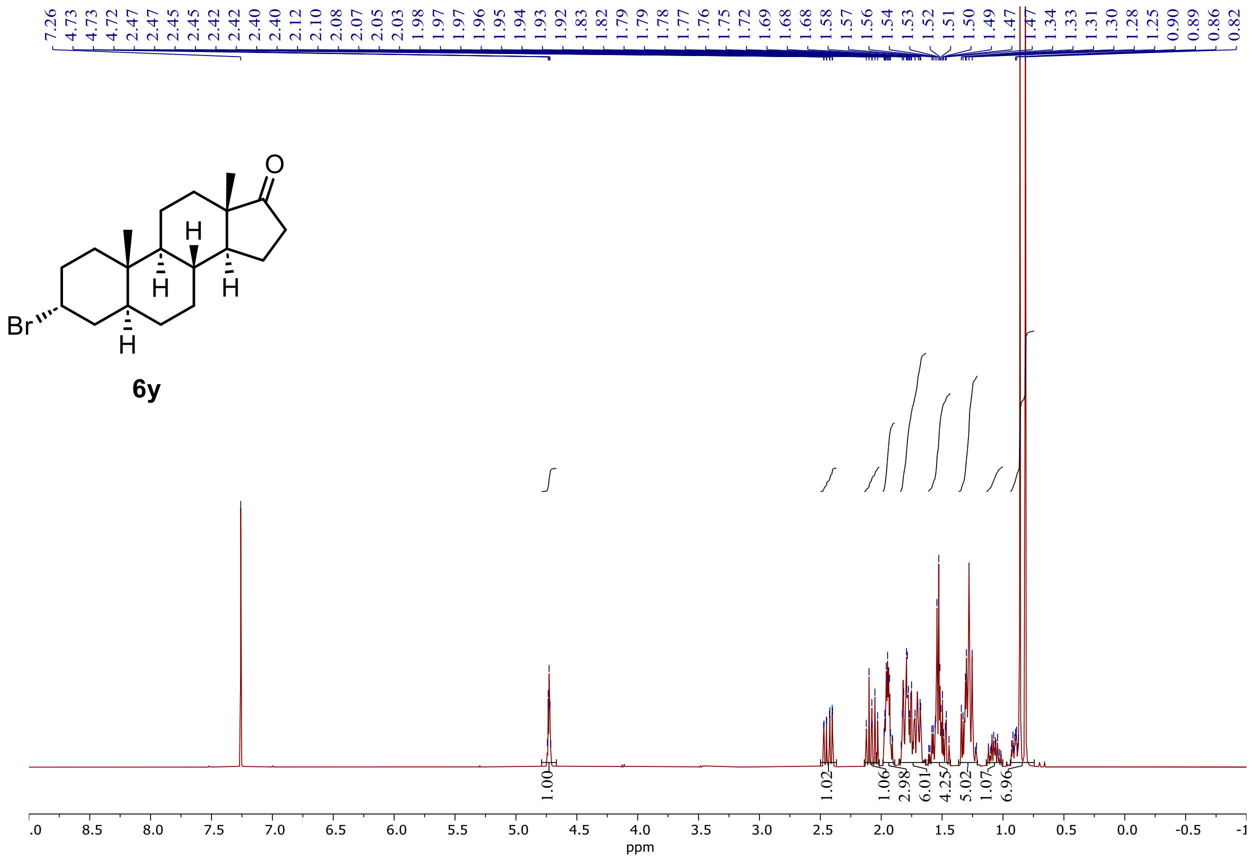

6y

^{13}C NMR (101 MHz, $CDCl_3$) of **6y**

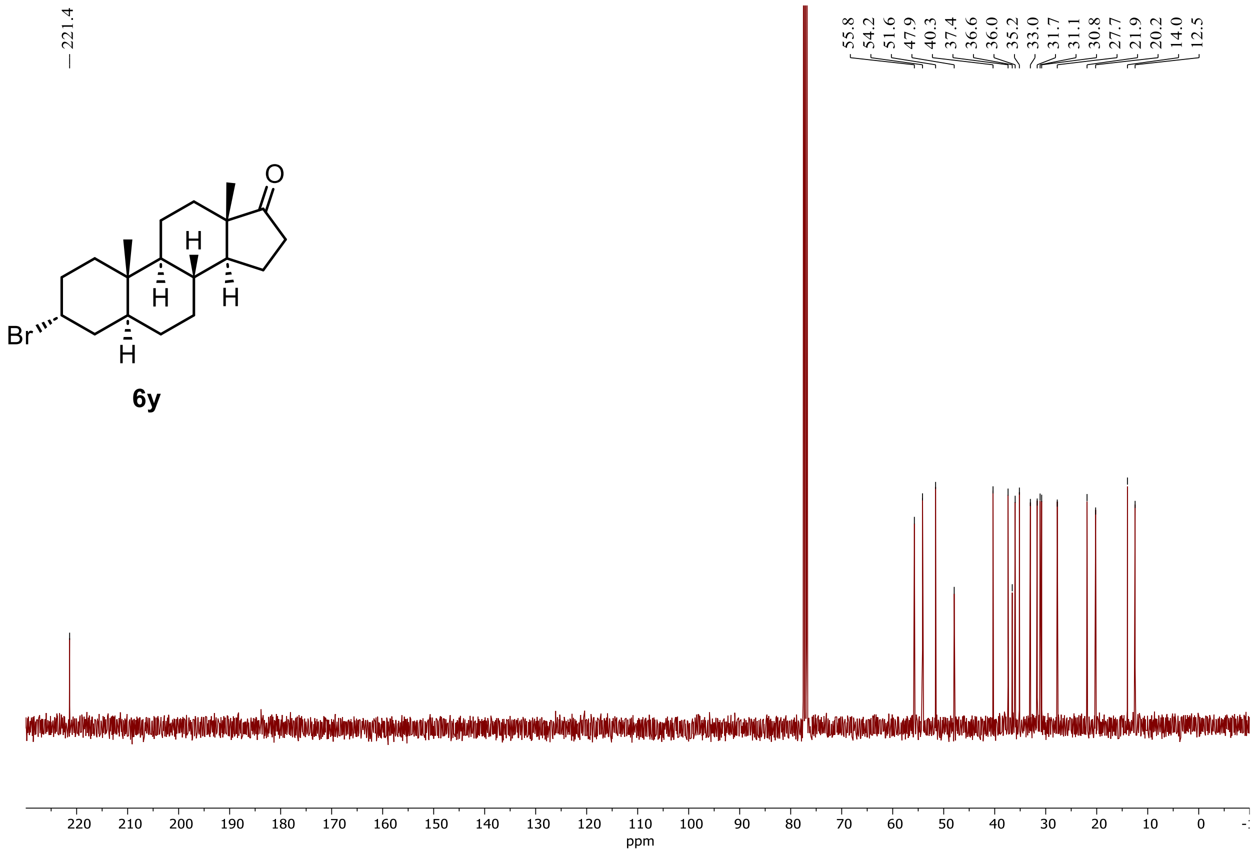

6y

^{1}H NMR (400 MHz, CDCl$_3$) of **6z** (*see procedure*)

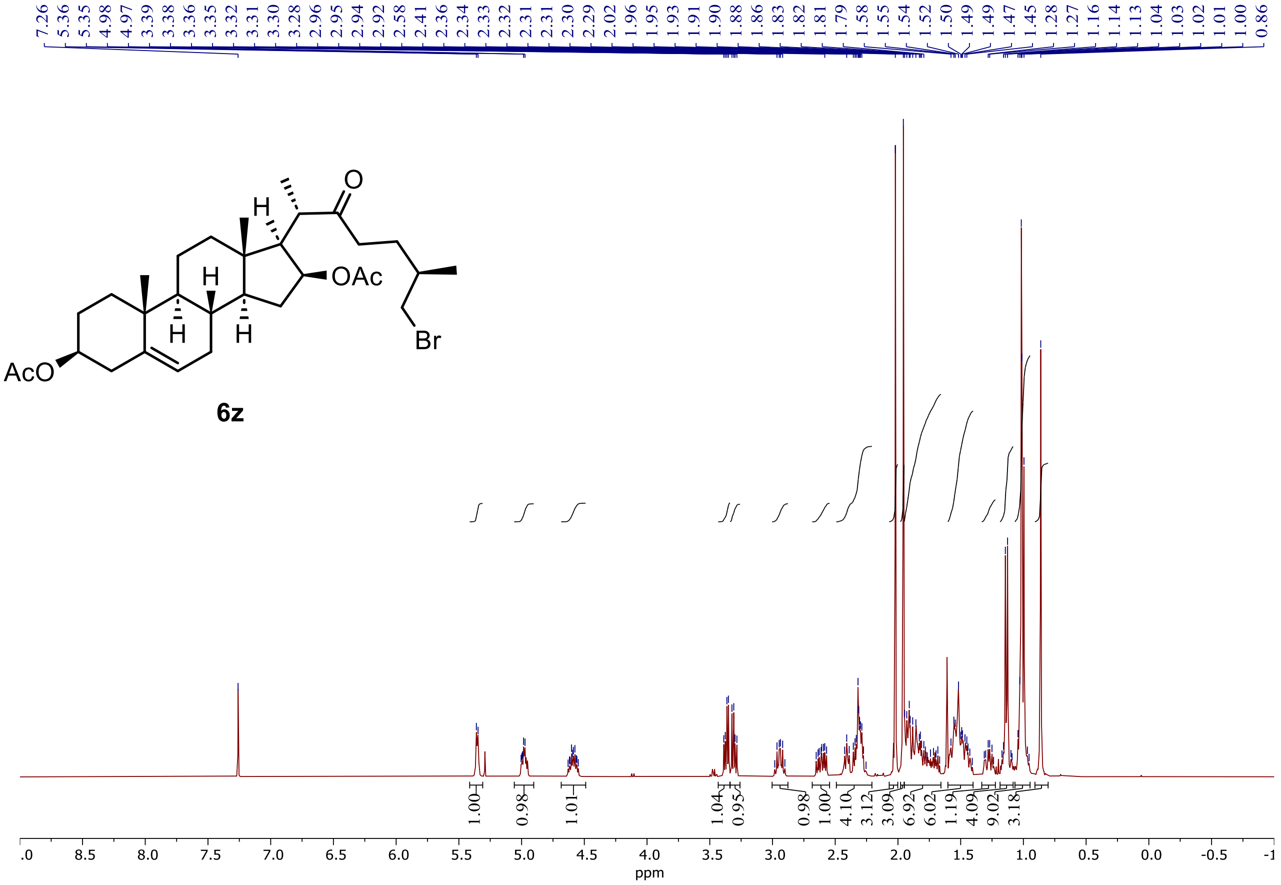

^{13}C NMR (101 MHz, CDCl$_3$) of **6z**

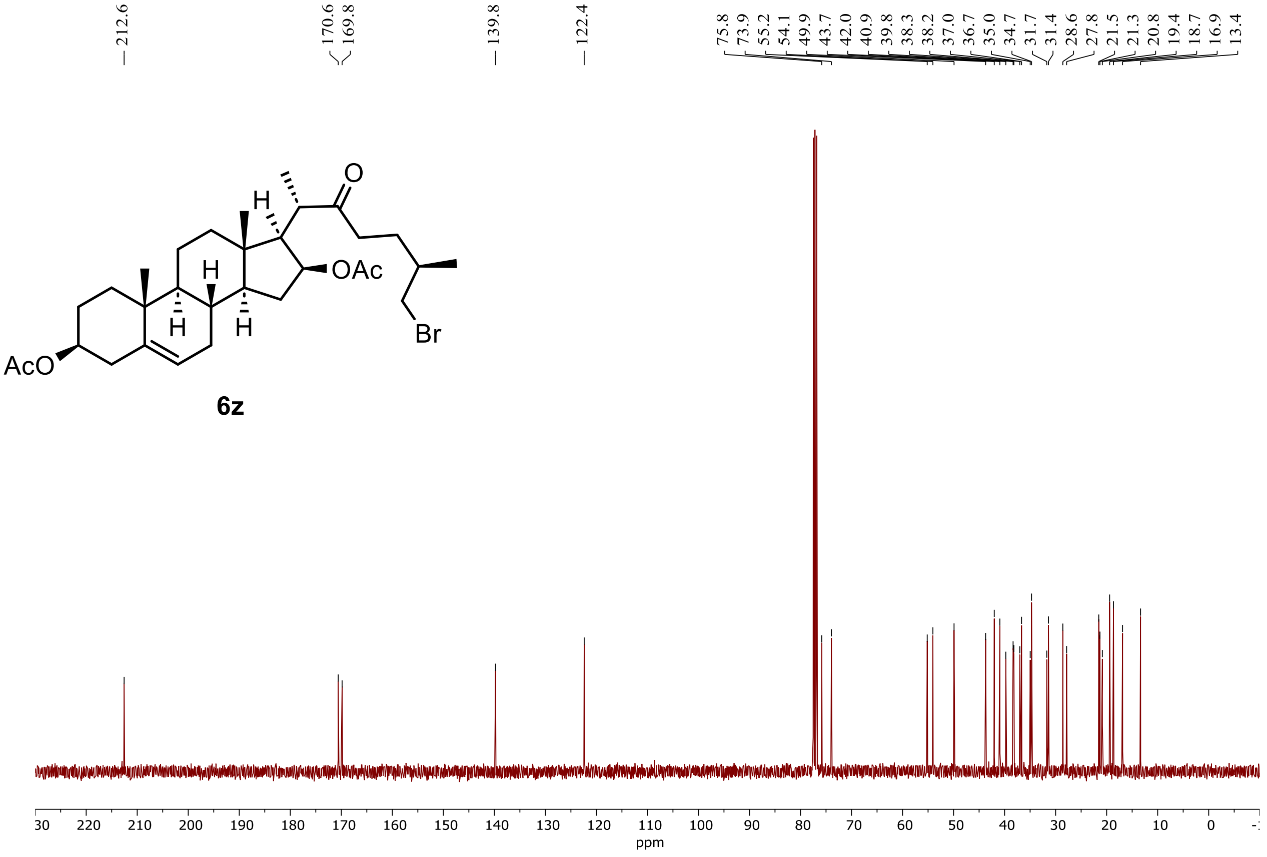

^{1}H NMR (500 MHz, $CDCl_3$) of **6aa** (*see procedure*)

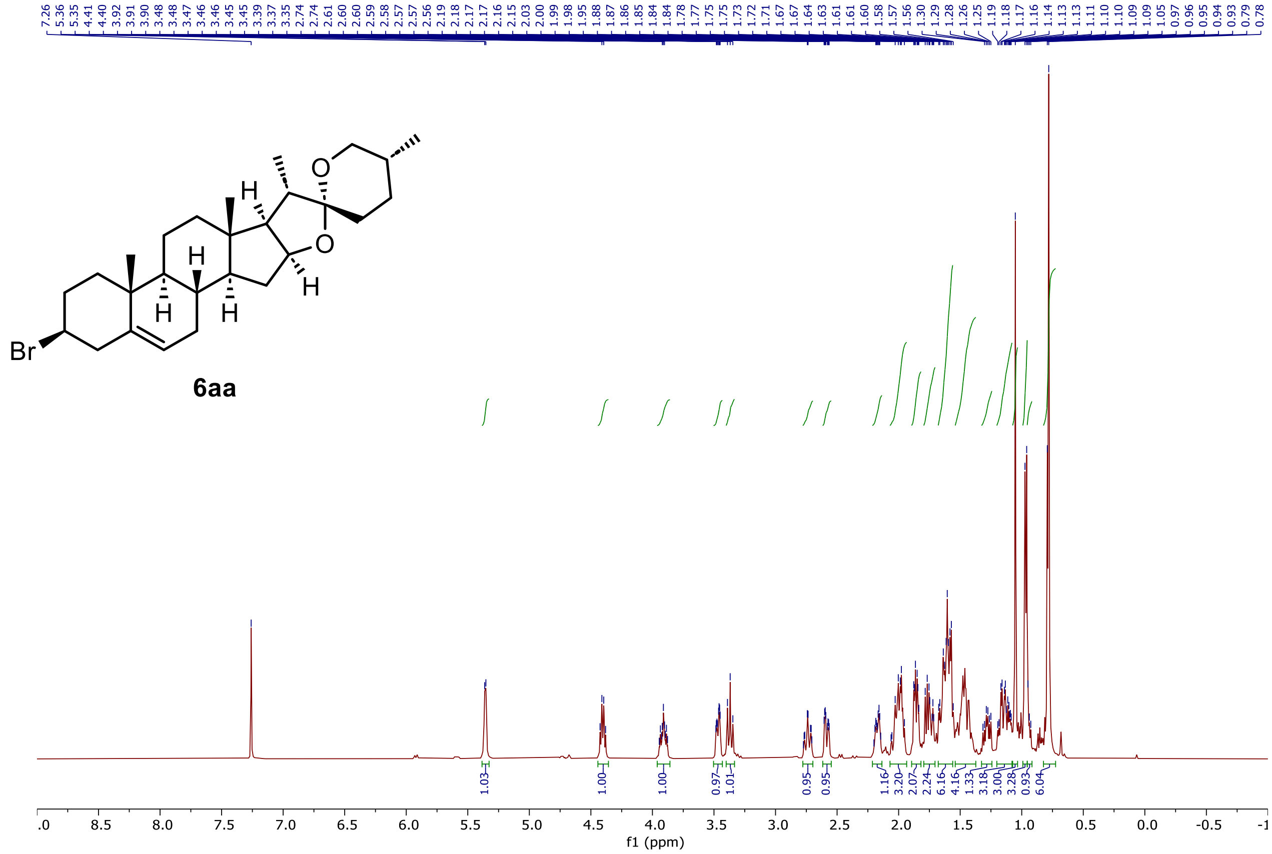

^{13}C NMR (126 MHz, $CDCl_3$) of **6aa**

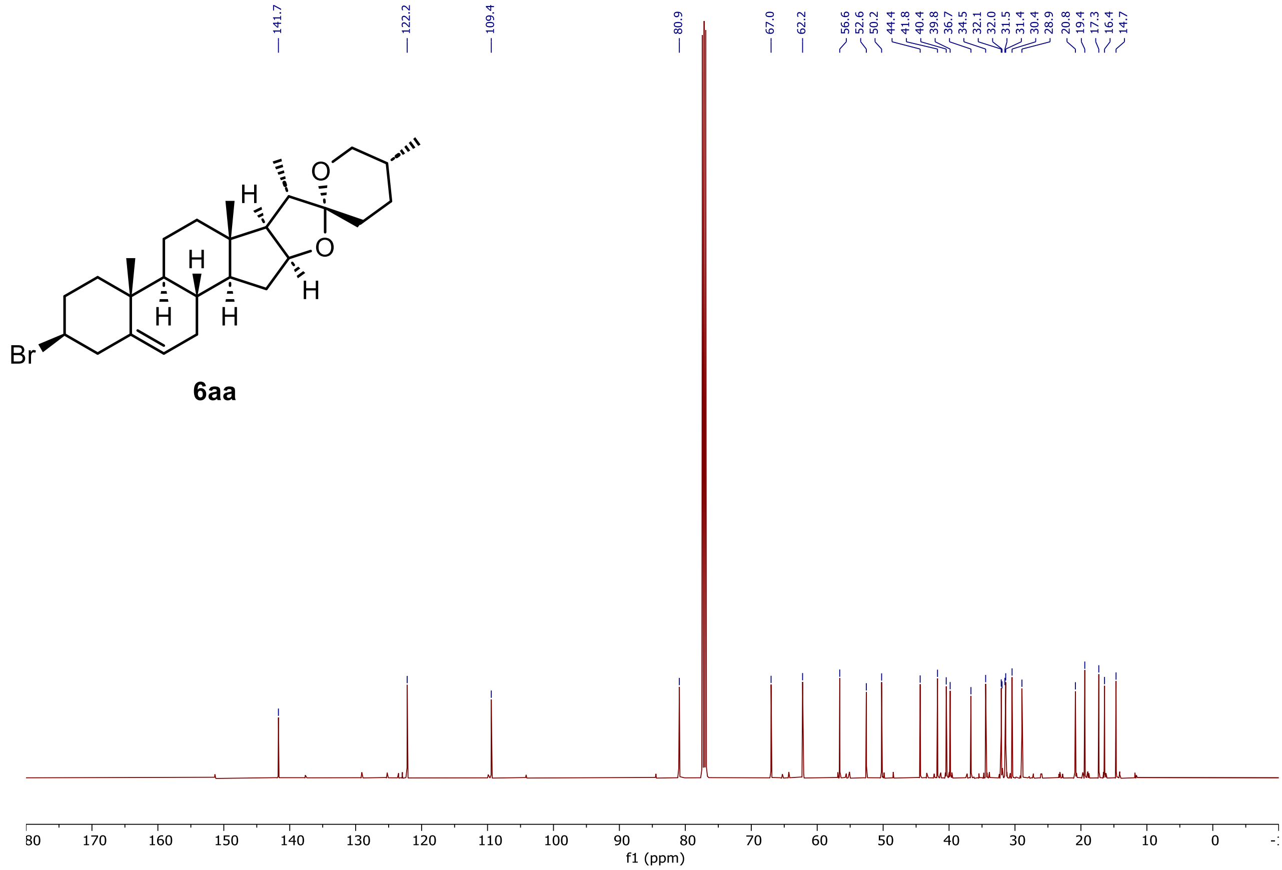

HSQC of **6aa**

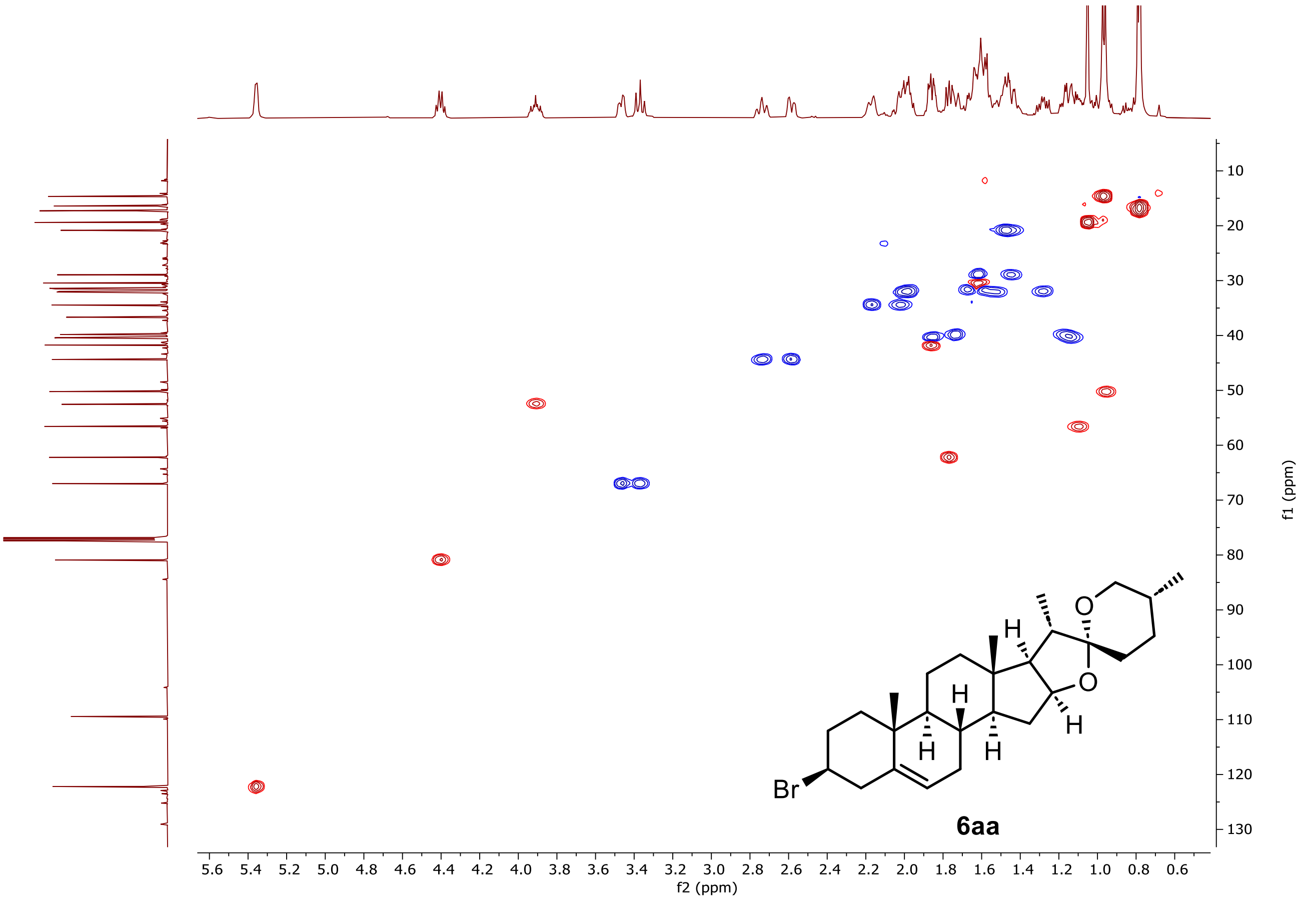

^{1}H NMR (500 MHz, $CDCl_3$) of **6ab** (see procedure)

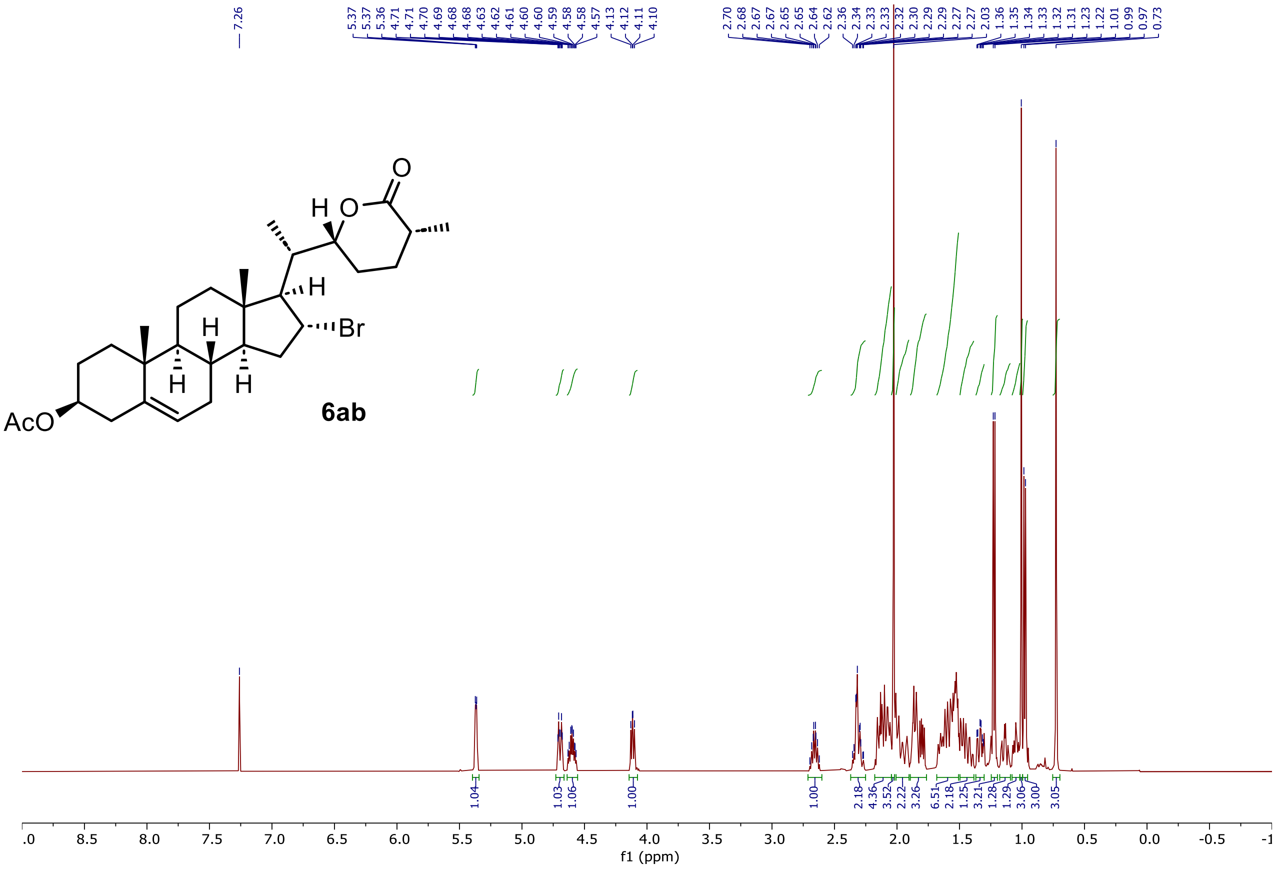

^{13}C NMR (126 MHz, $CDCl_3$) of **6ab**

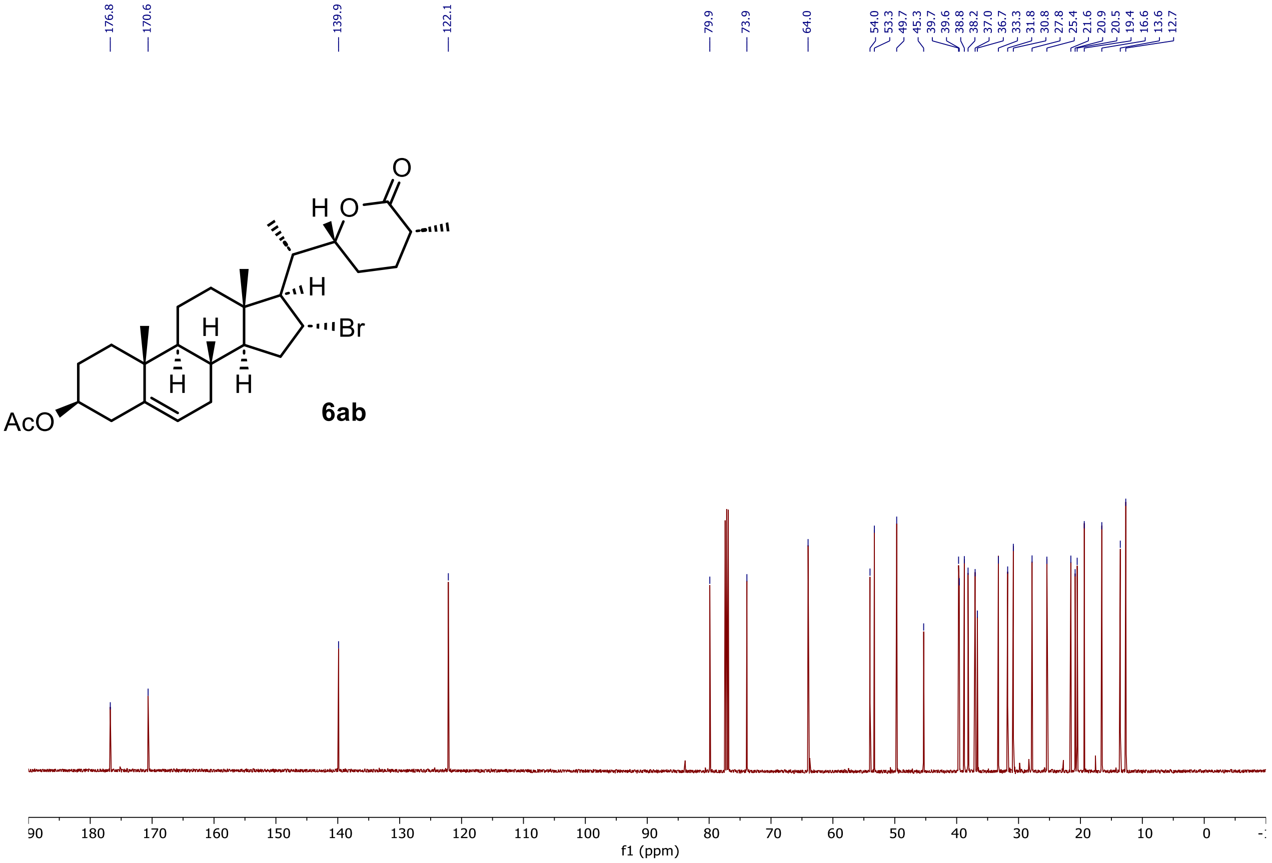

HSQC of **6ab**

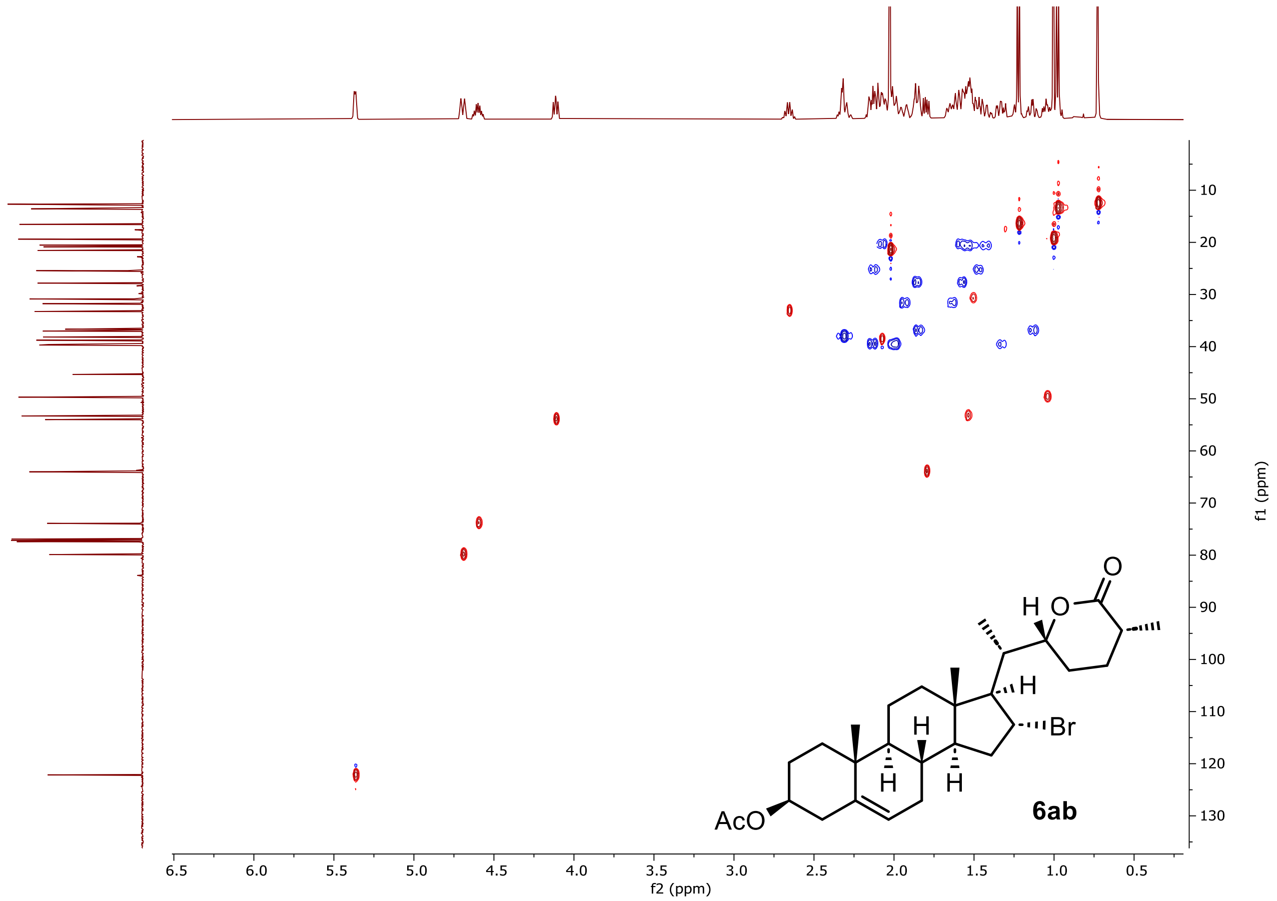

^{1}H NMR (500 MHz, $CDCl_3$) of **6ac** (*see procedure*)

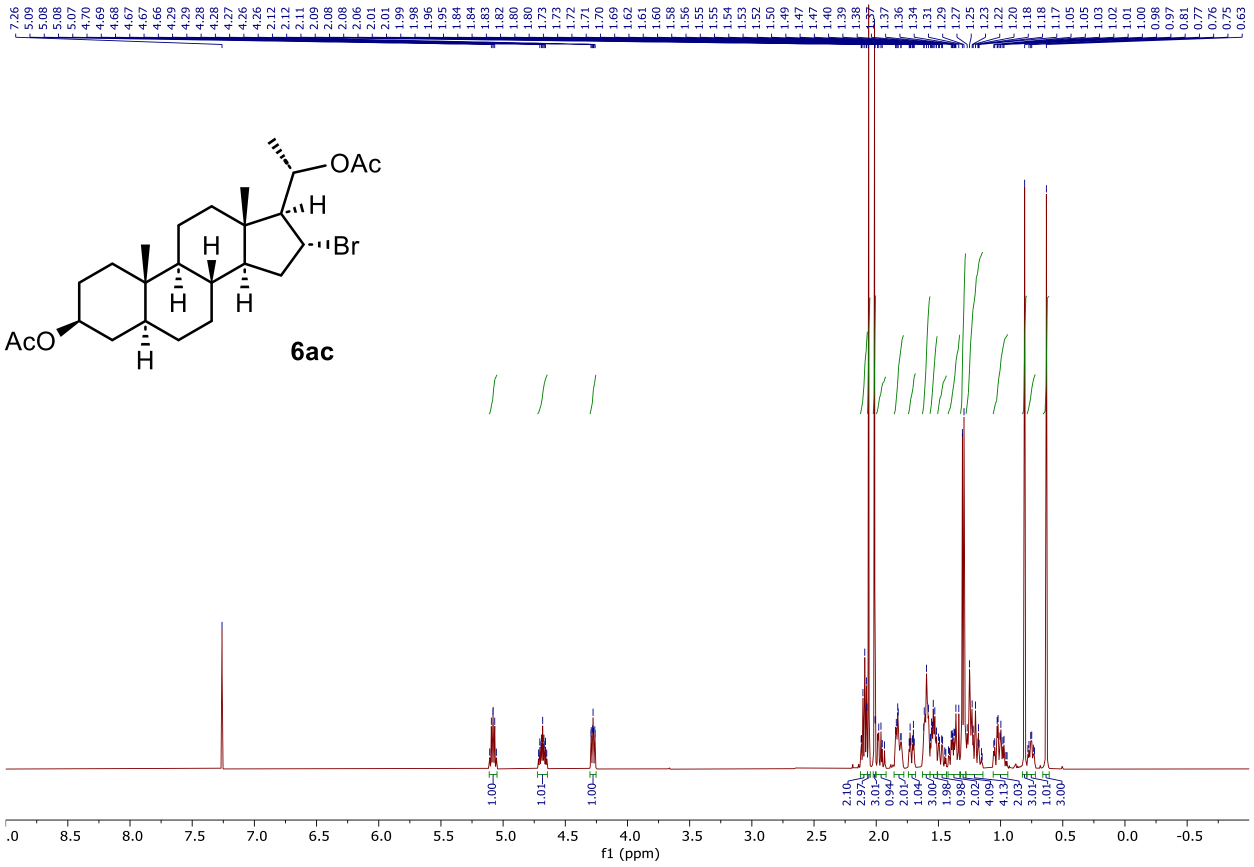

^{13}C NMR (126 MHz, $CDCl_3$) of **6ac**

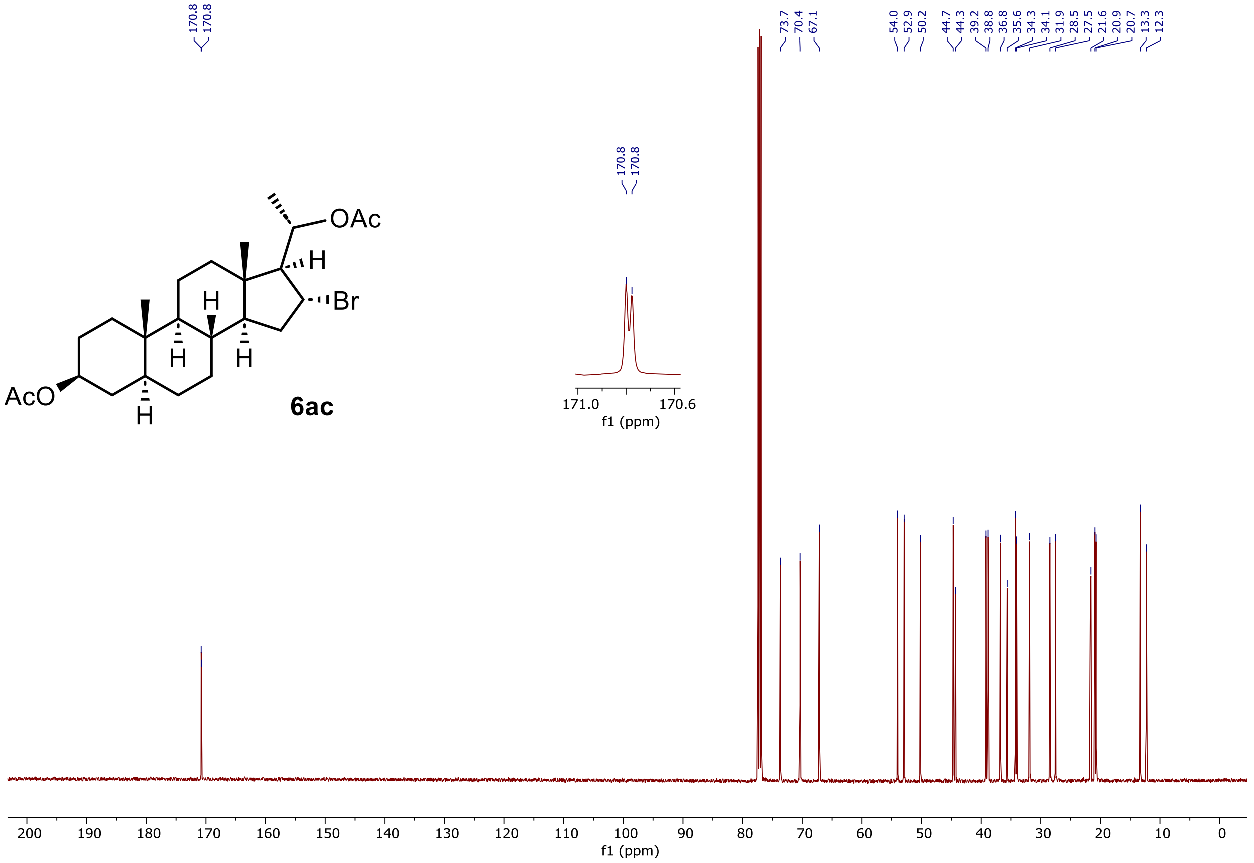

HSQC of **6ac**

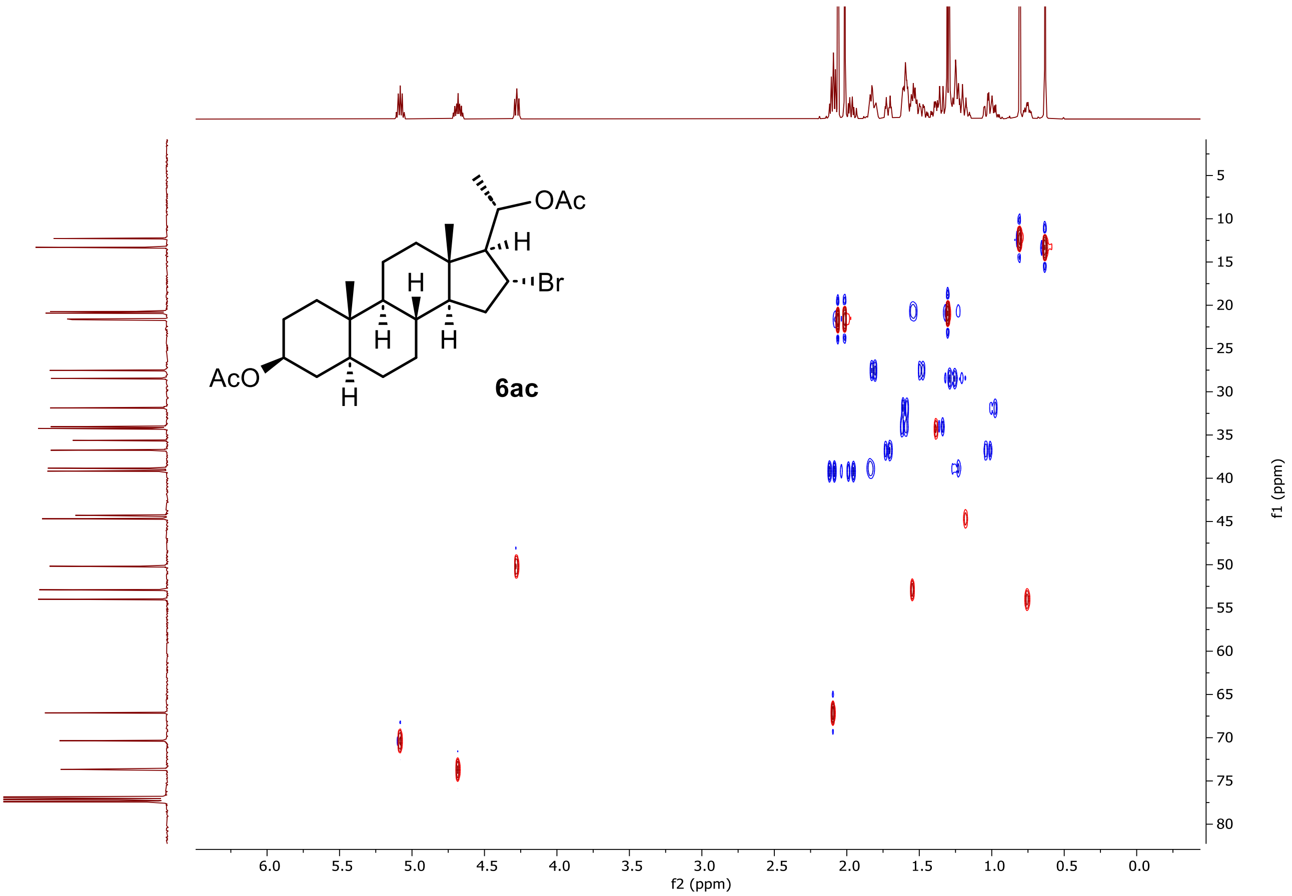
OAc
H
Br
H
H
H
AcO
H
6ac
6.0
5.5
5.0
4.5
4.0
3.5
3.0
2.5
2.0
1.5
1.0
0.5
0.0
f2 (ppm)
5
10
15
20
25
30
35
40
45
50
55
60
65
70
75
80
f1 (ppm)

^{1}H NMR (500 MHz, $CDCl_3$) of **6ad** (*see procedure*)

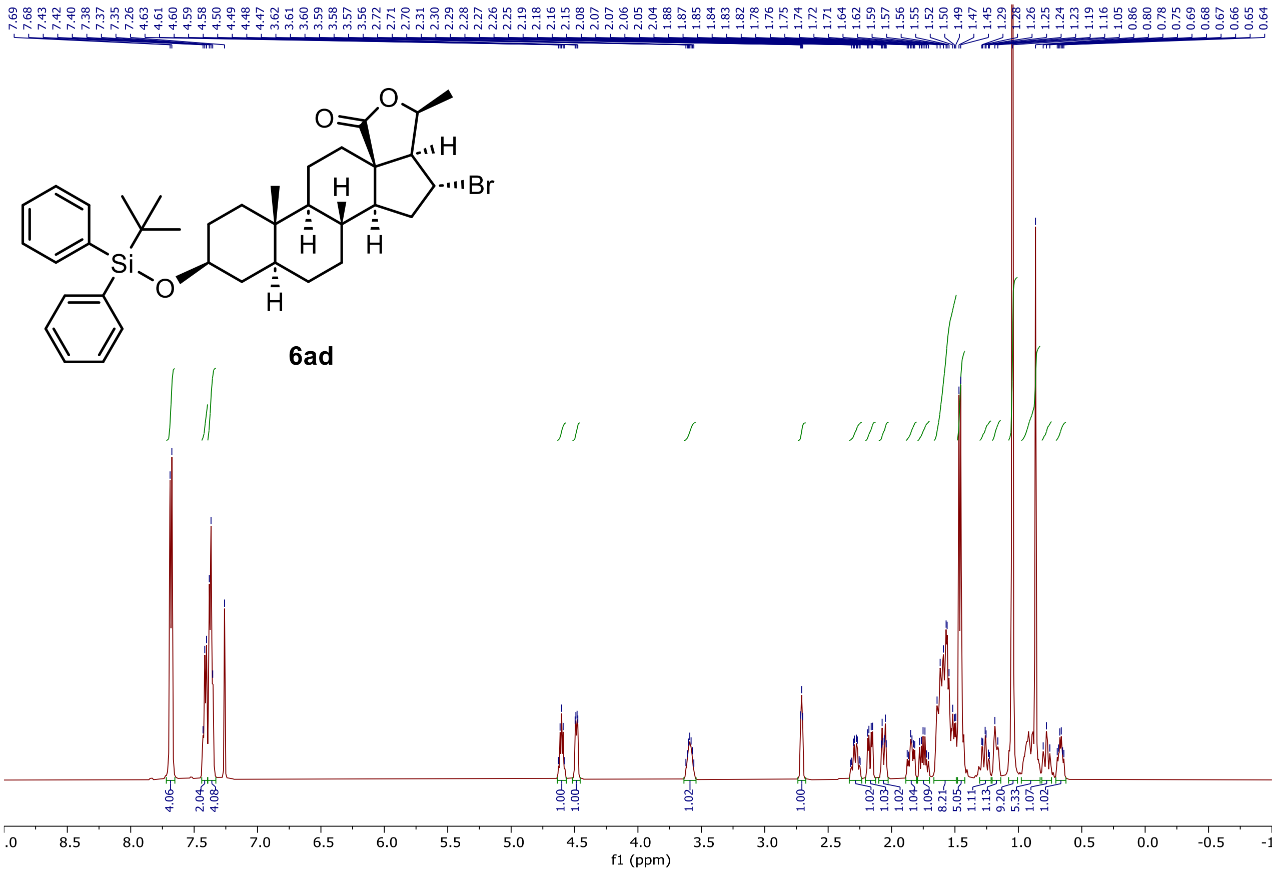

^{13}C NMR (126 MHz, $CDCl_3$) of **6ad**

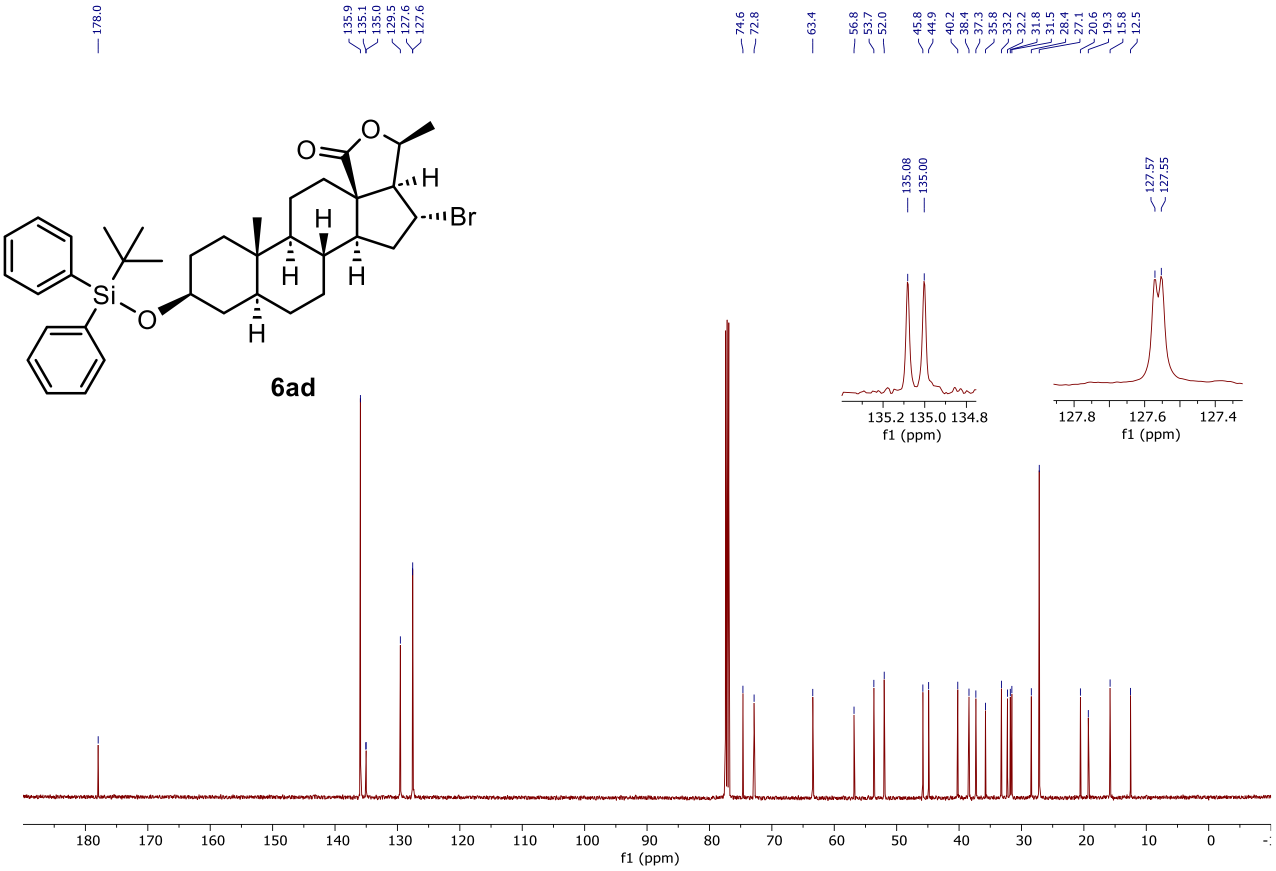

HSQC of **6ad**

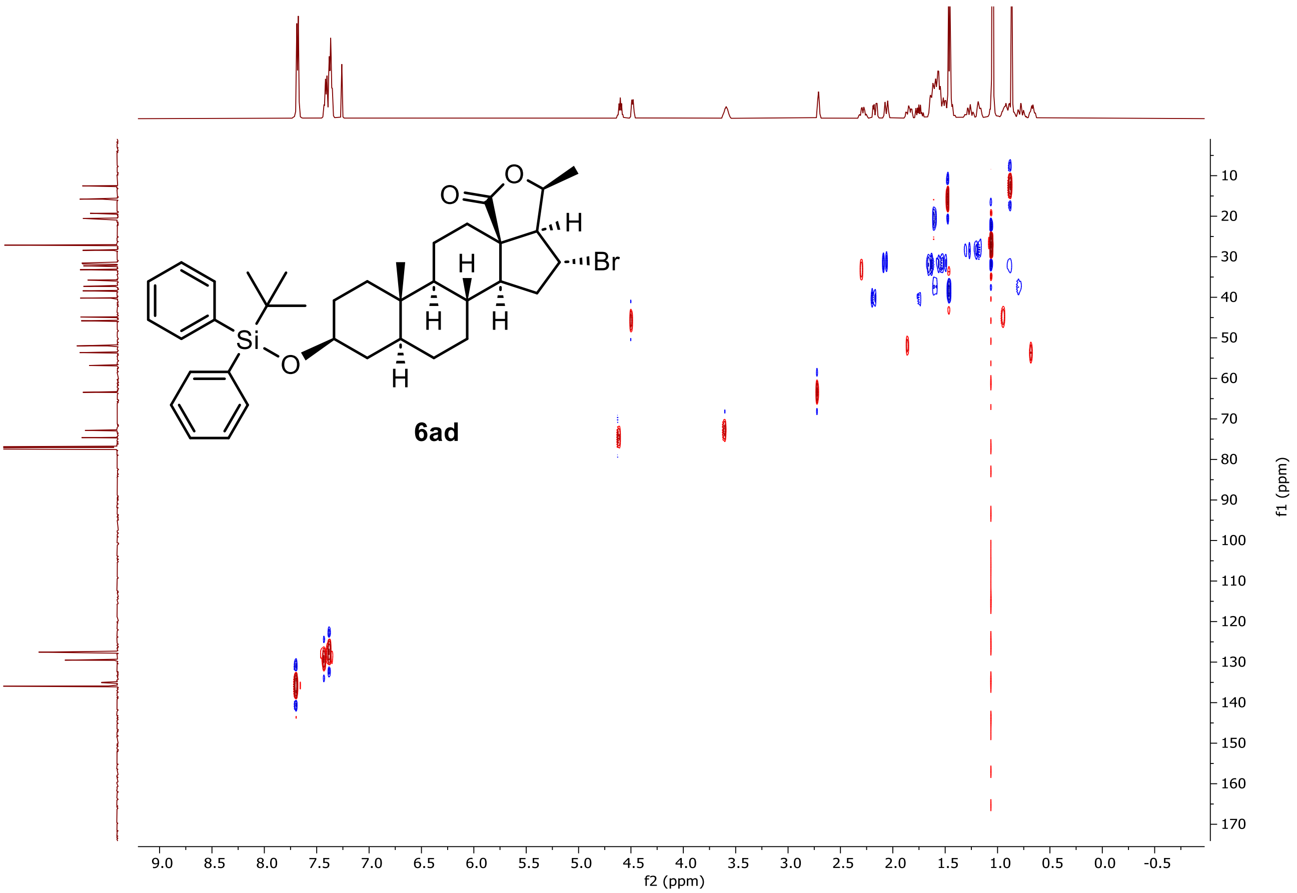

O
O
H
Br
H
H
H
Si
O
H
6ad
9.0
8.5
8.0
7.5
7.0
6.5
6.0
5.5
5.0
4.5
4.0
3.5
3.0
2.5
2.0
1.5
1.0
0.5
0.0
-0.5
f2 (ppm)
10
20
30
40
50
60
70
80
90
100
110
120
130
140
150
160
170
f1 (ppm)

^{1}H NMR (500 MHz, $CDCl_3$) of **6ae** (*see procedure*)

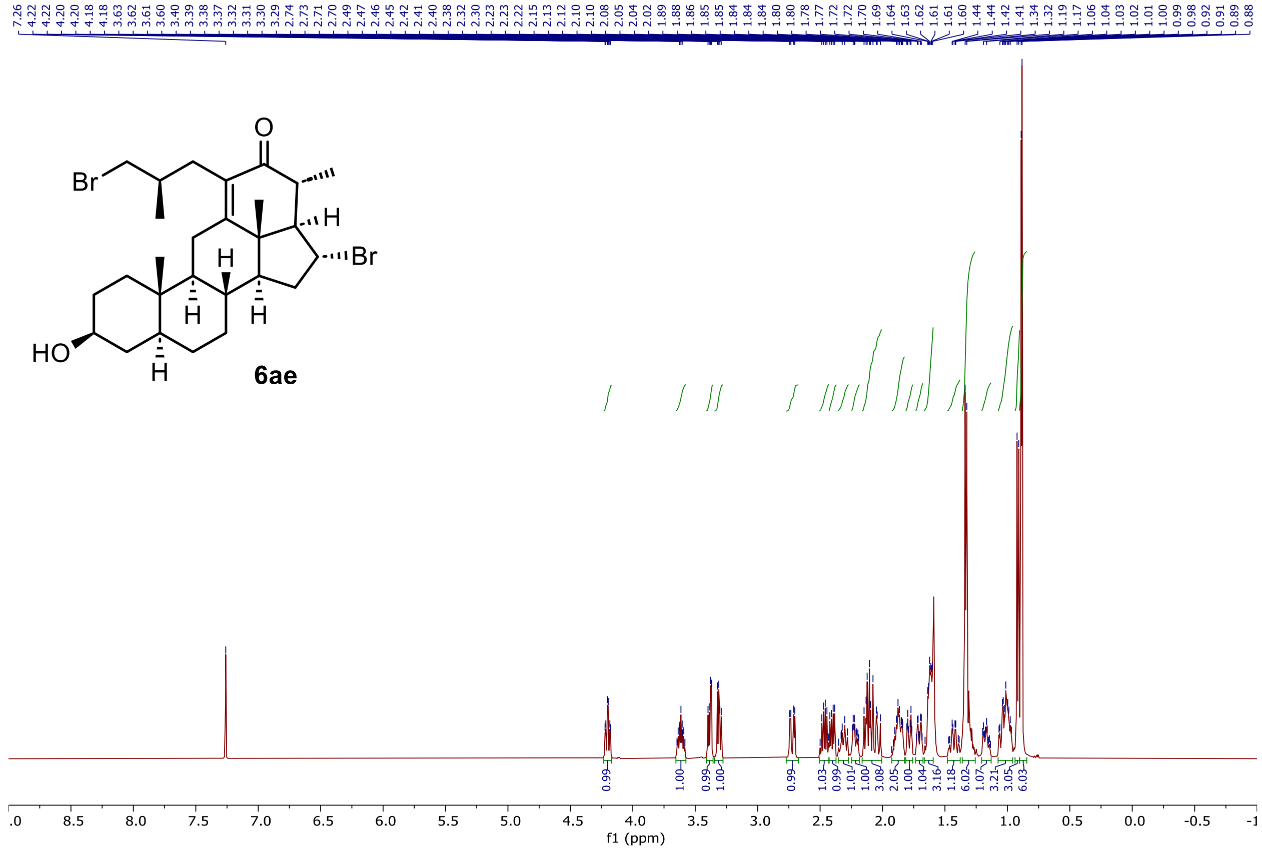

^{13}C NMR (126 MHz, $CDCl_3$) of **6ae**

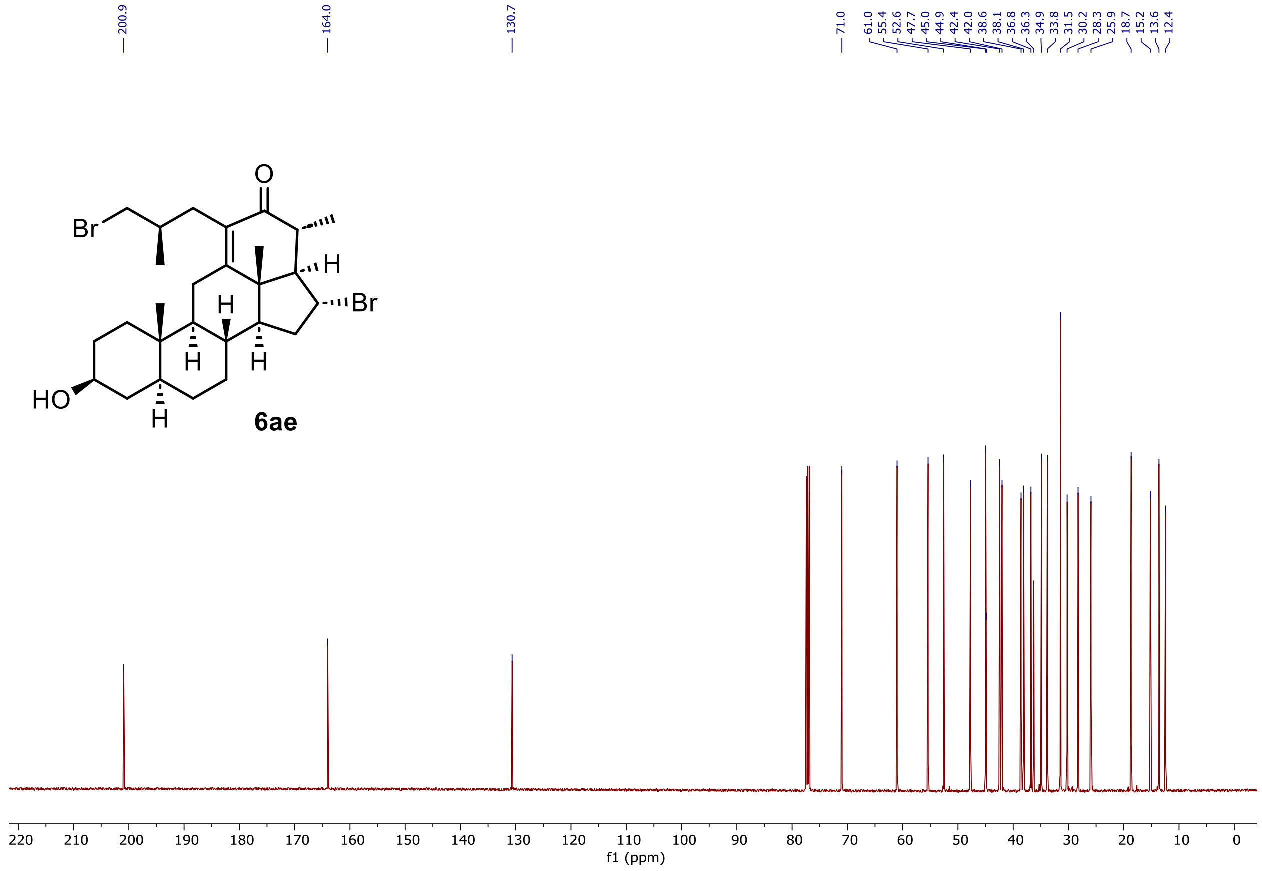

^{1}H NMR (500 MHz, $CDCl_3$) of **7a** (*see procedure*)

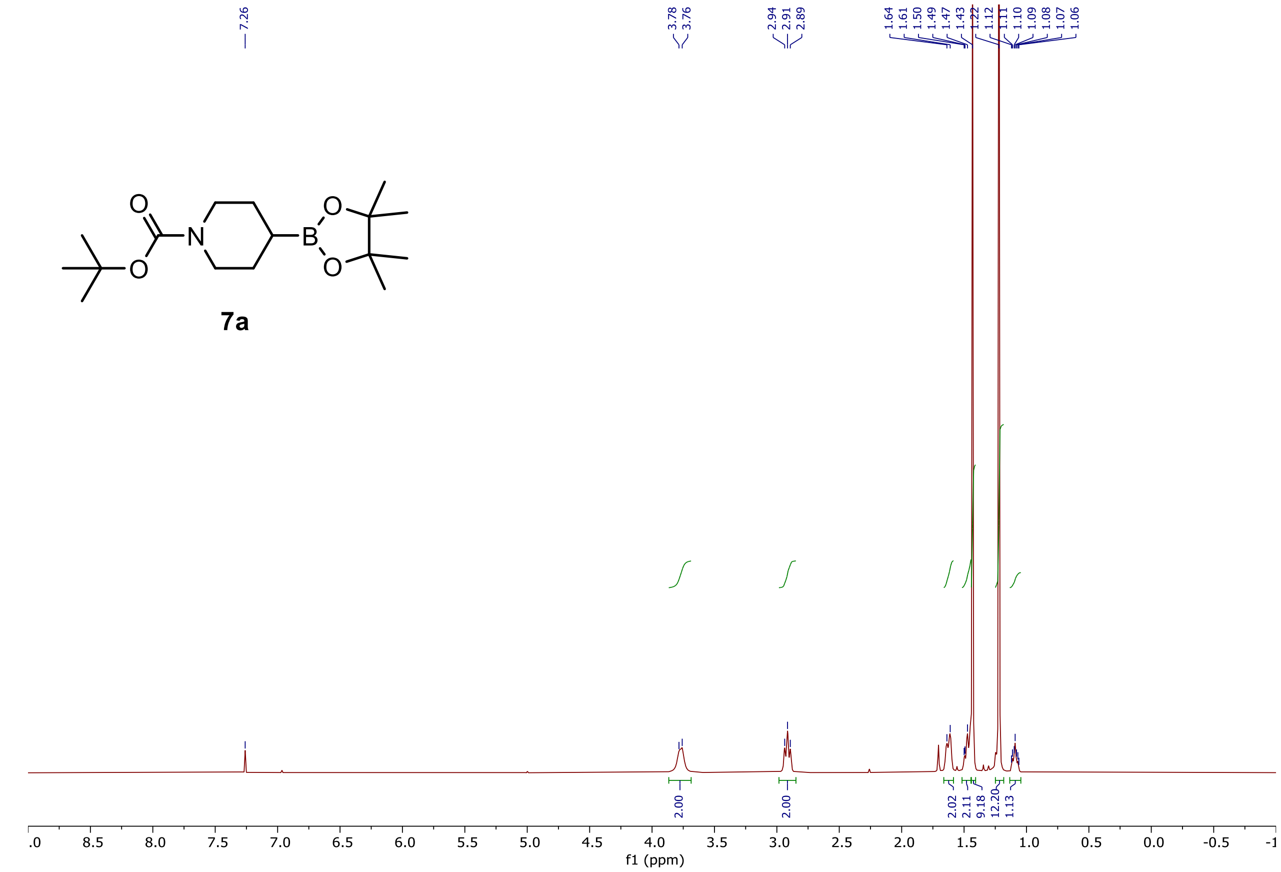

^{13}C NMR (126 MHz, $CDCl_3$) of **7a**

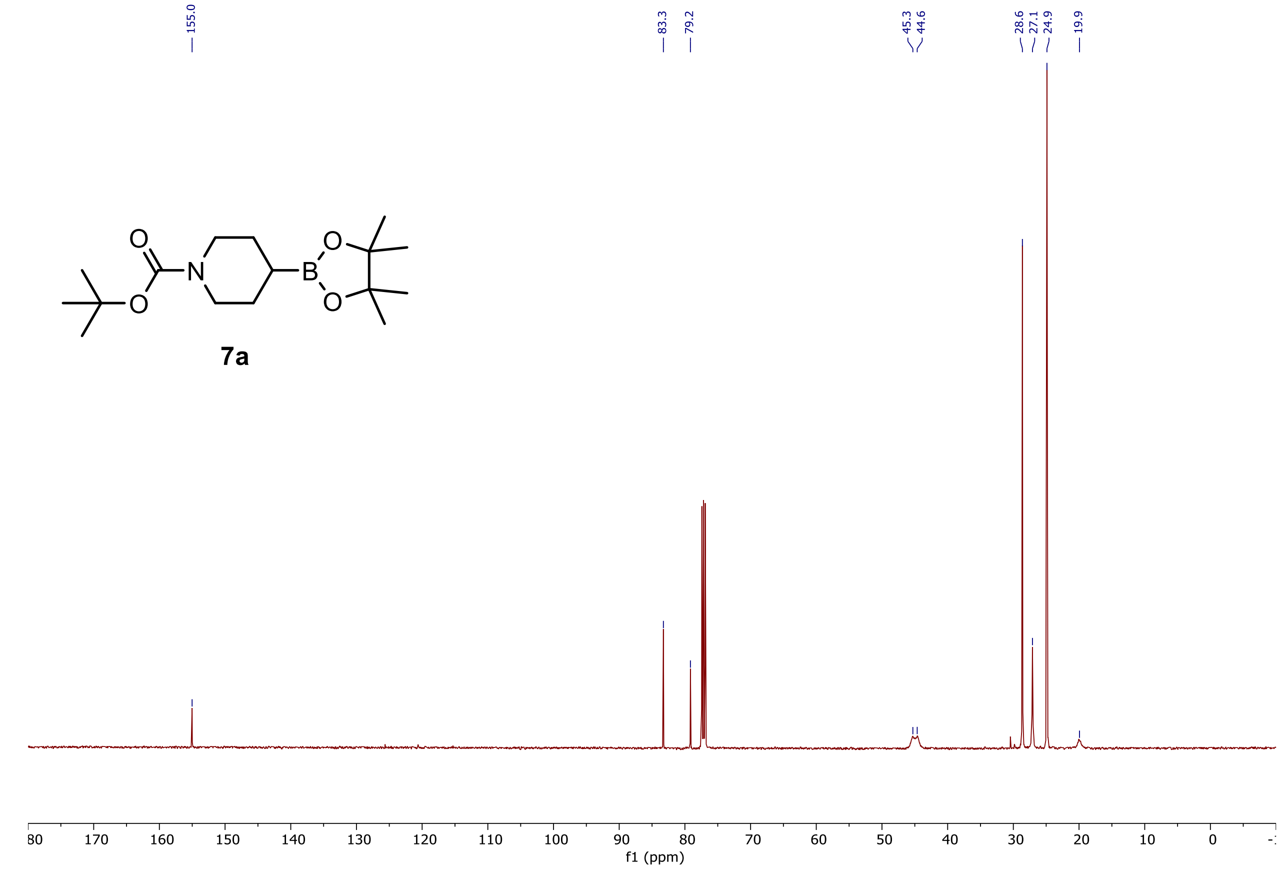

^{1}H NMR (500 MHz, $CDCl_3$) of **7b** (*see procedure*)

7b

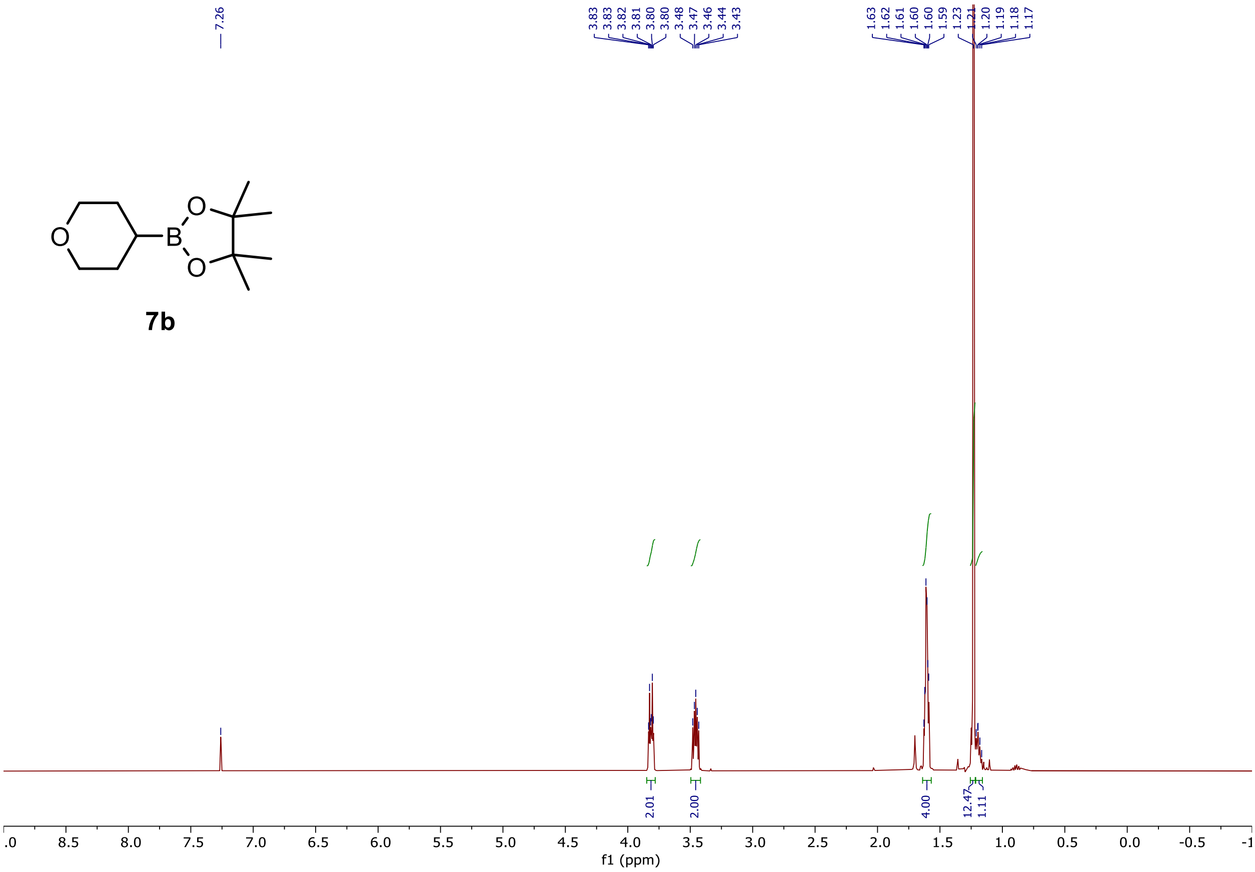

^{13}C NMR (126 MHz, $CDCl_3$) of **7b**

7b

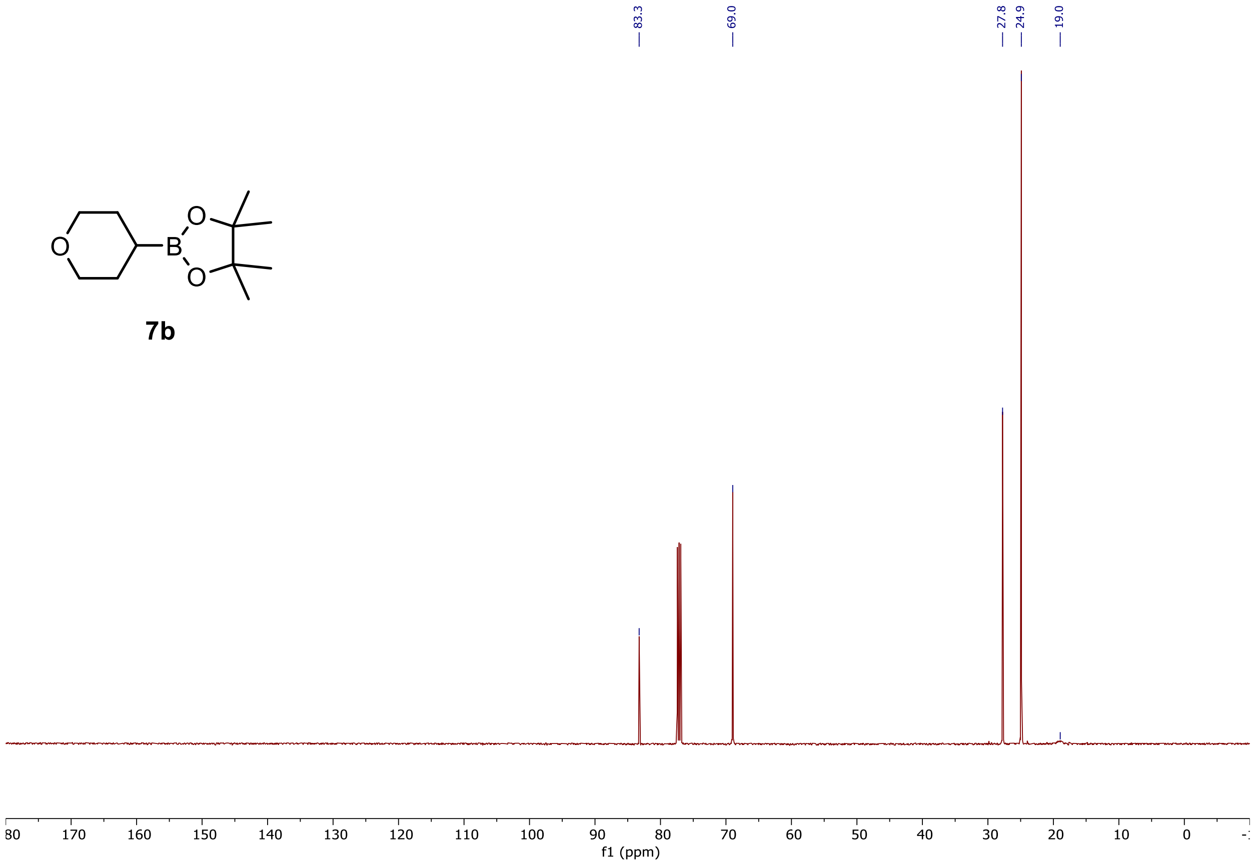

^{1}H NMR (400 MHz, $CDCl_3$) of **7c** (see procedure)

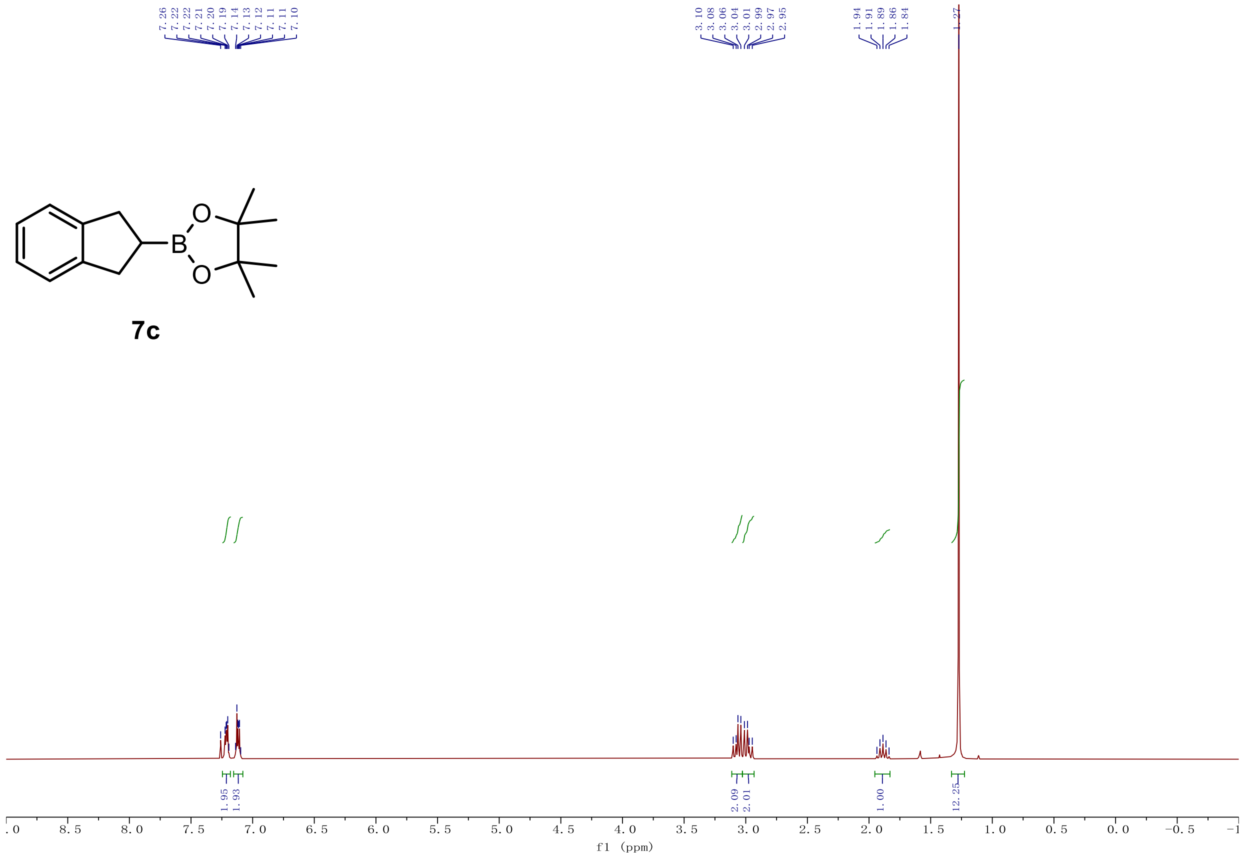

7c

^{13}C NMR (101 MHz, $CDCl_3$) of **7c**

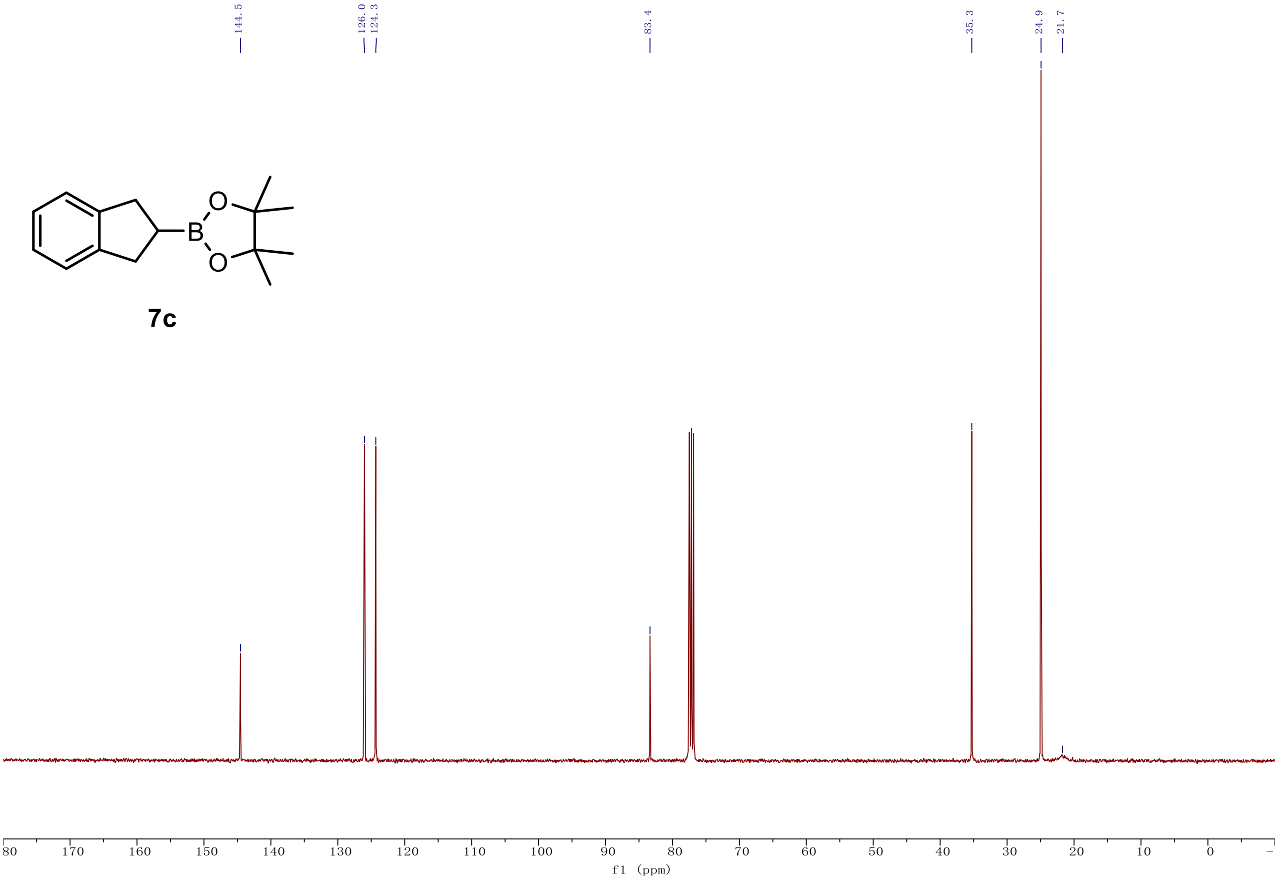

7c

^{1}H NMR (500 MHz, $CDCl_3$) of **7d** (*see procedure*)

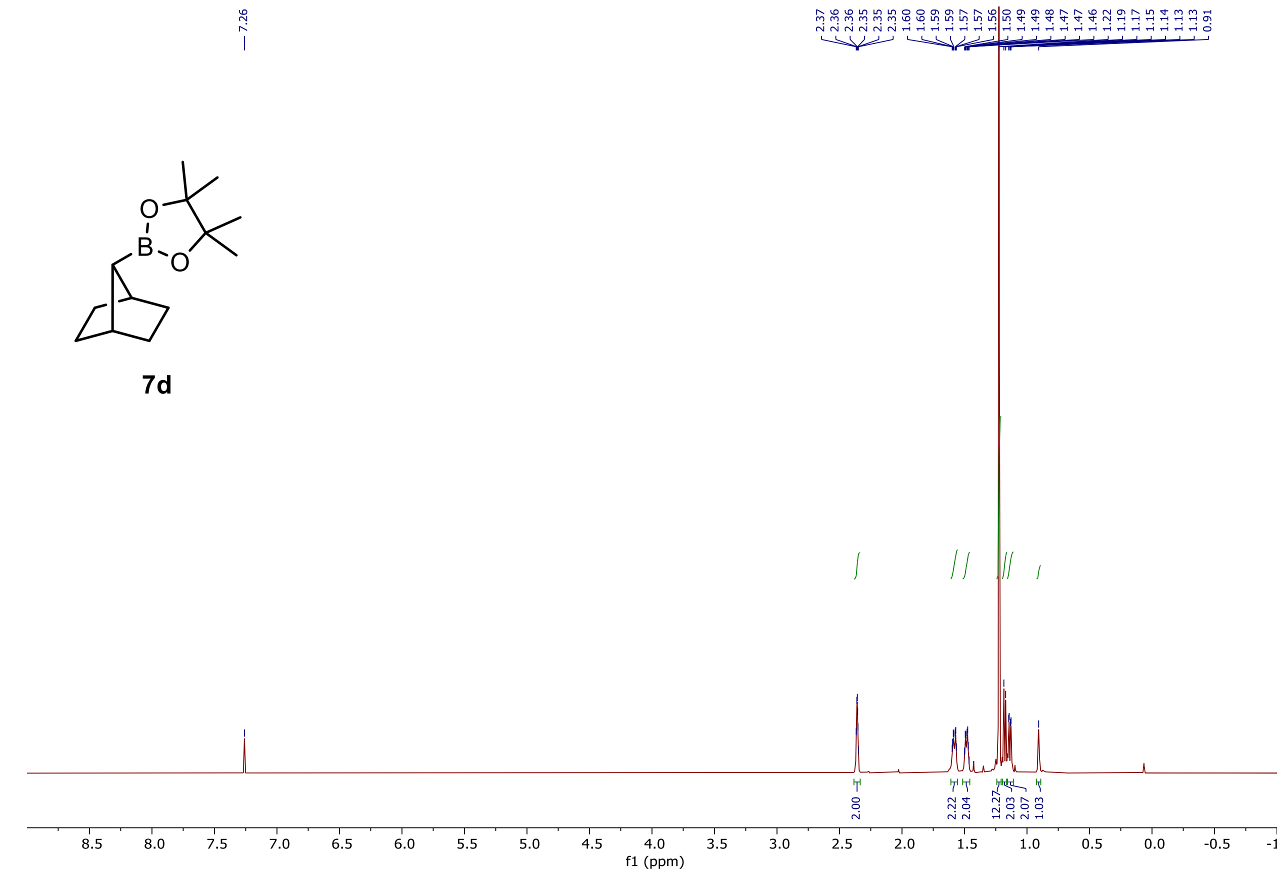

^{13}C NMR (126 MHz, $CDCl_3$) of **7d**

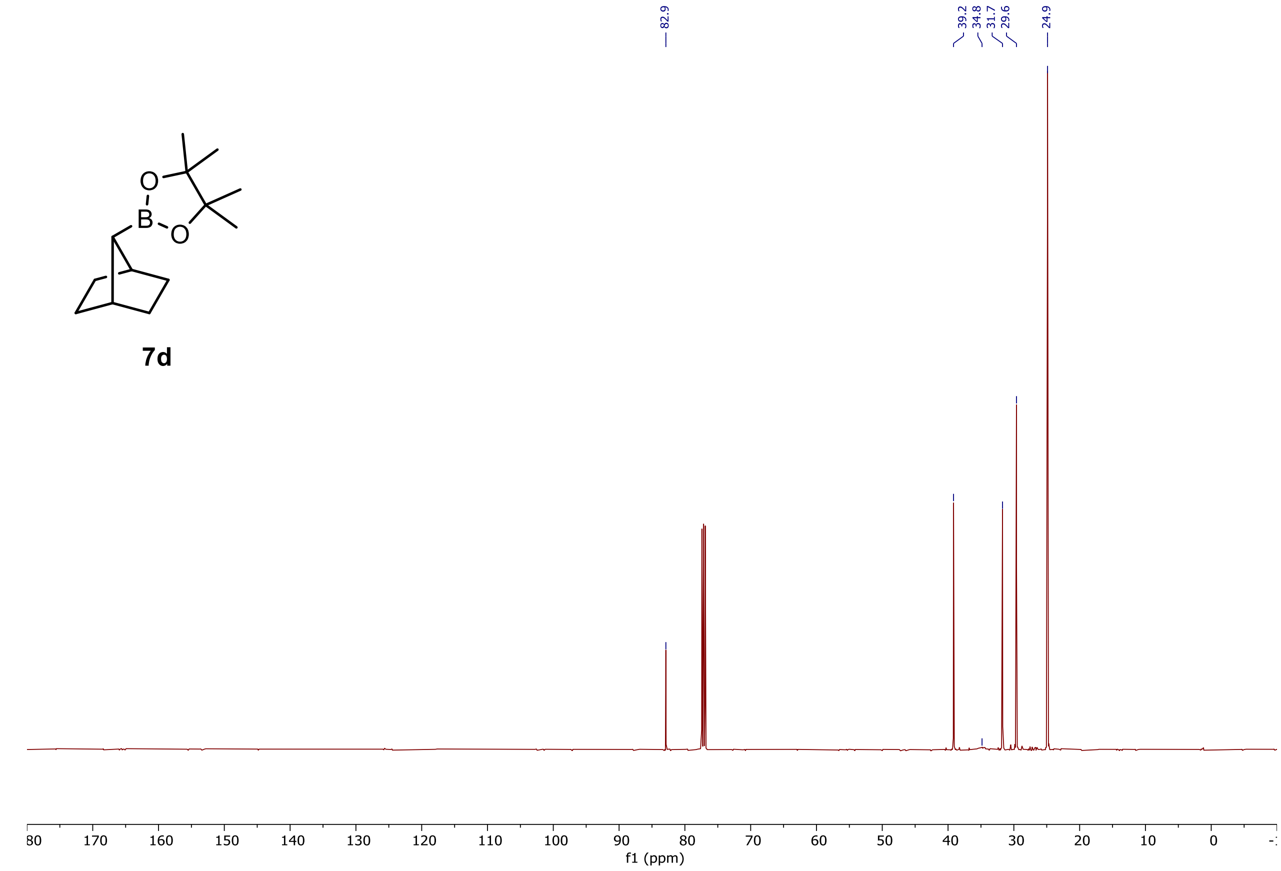

^{11}B NMR (128 MHz, $CDCl_3$) of **7d**

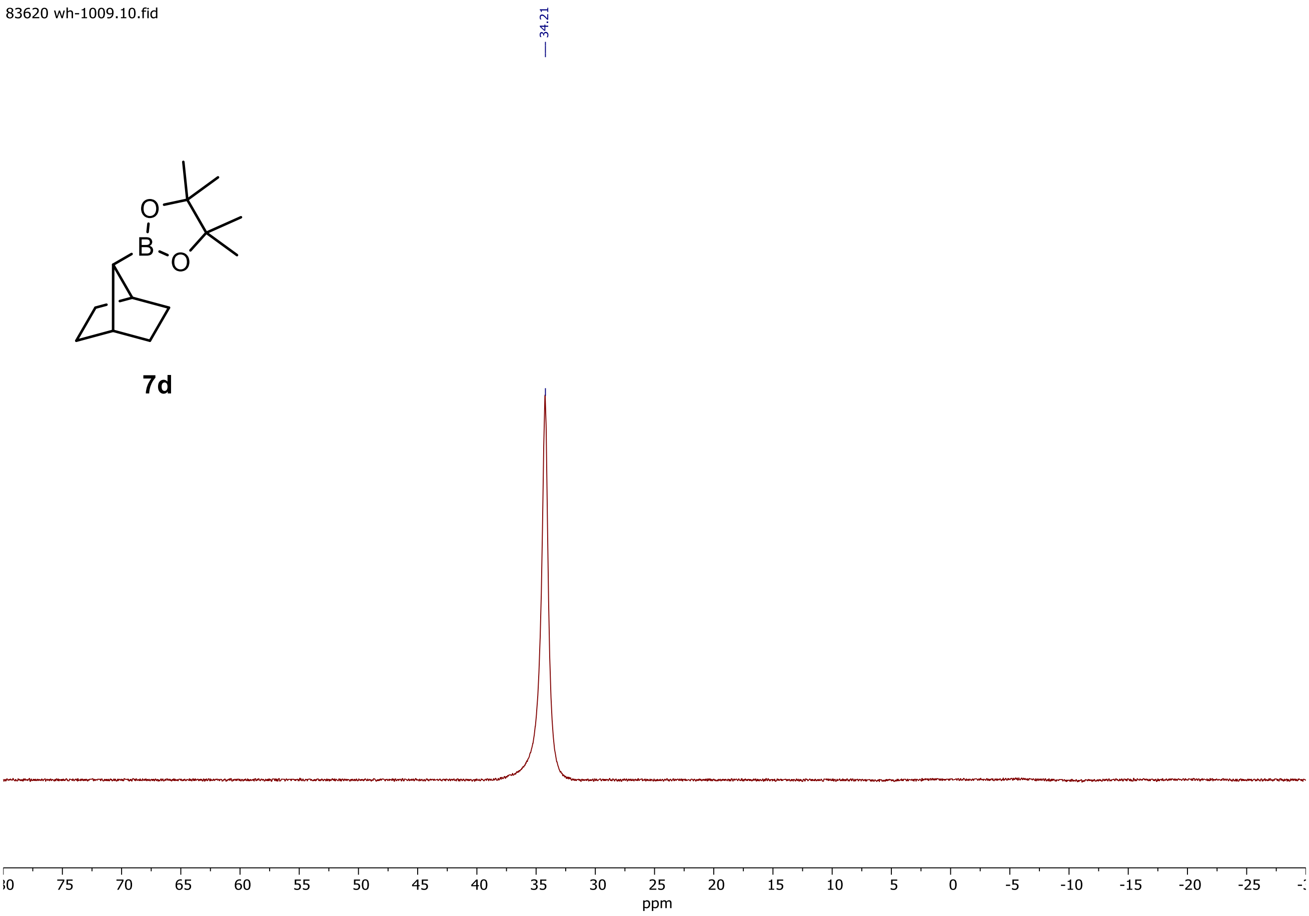

^{1}H NMR (500 MHz, $CDCl_3$) of **7e** (*see procedure*)

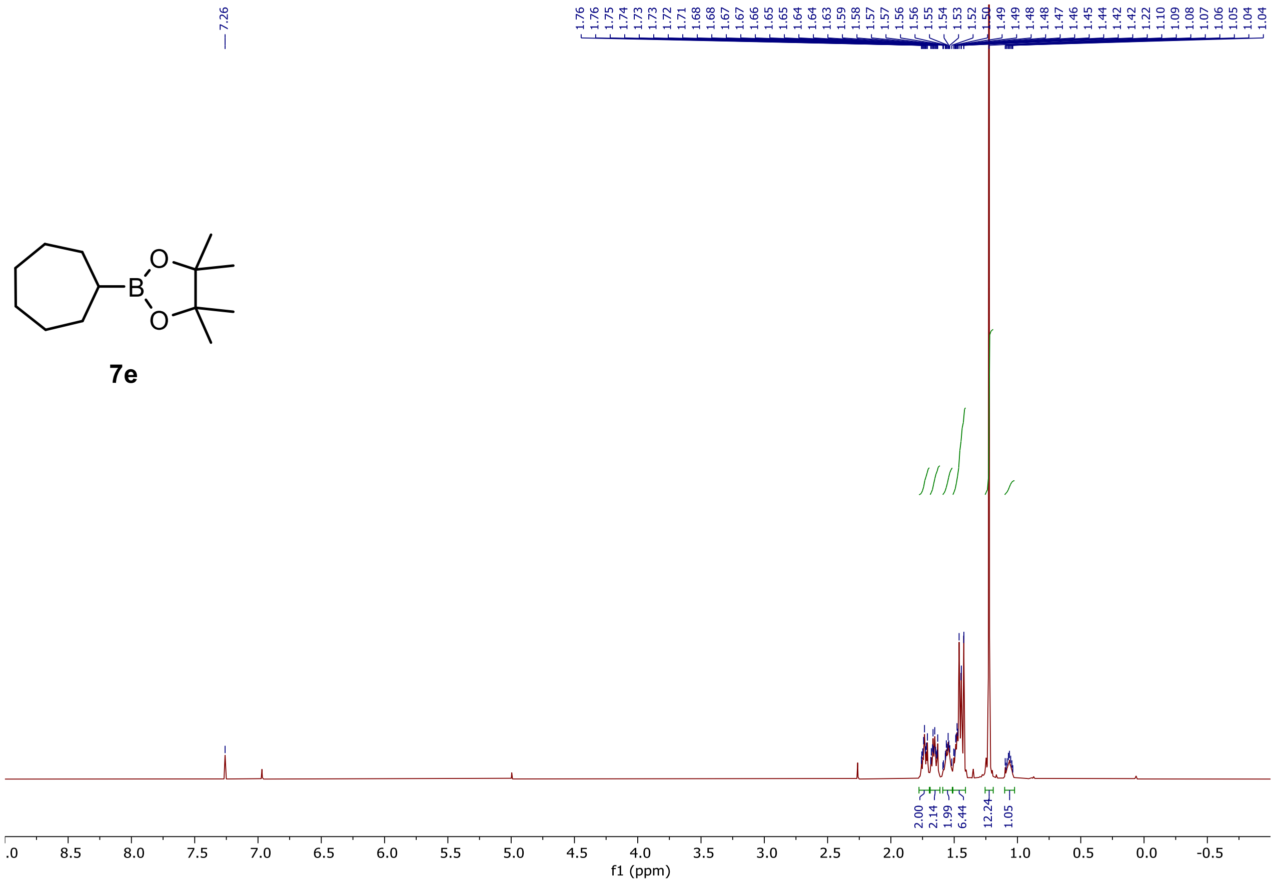

^{13}C NMR (126 MHz, $CDCl_3$) of **7e**

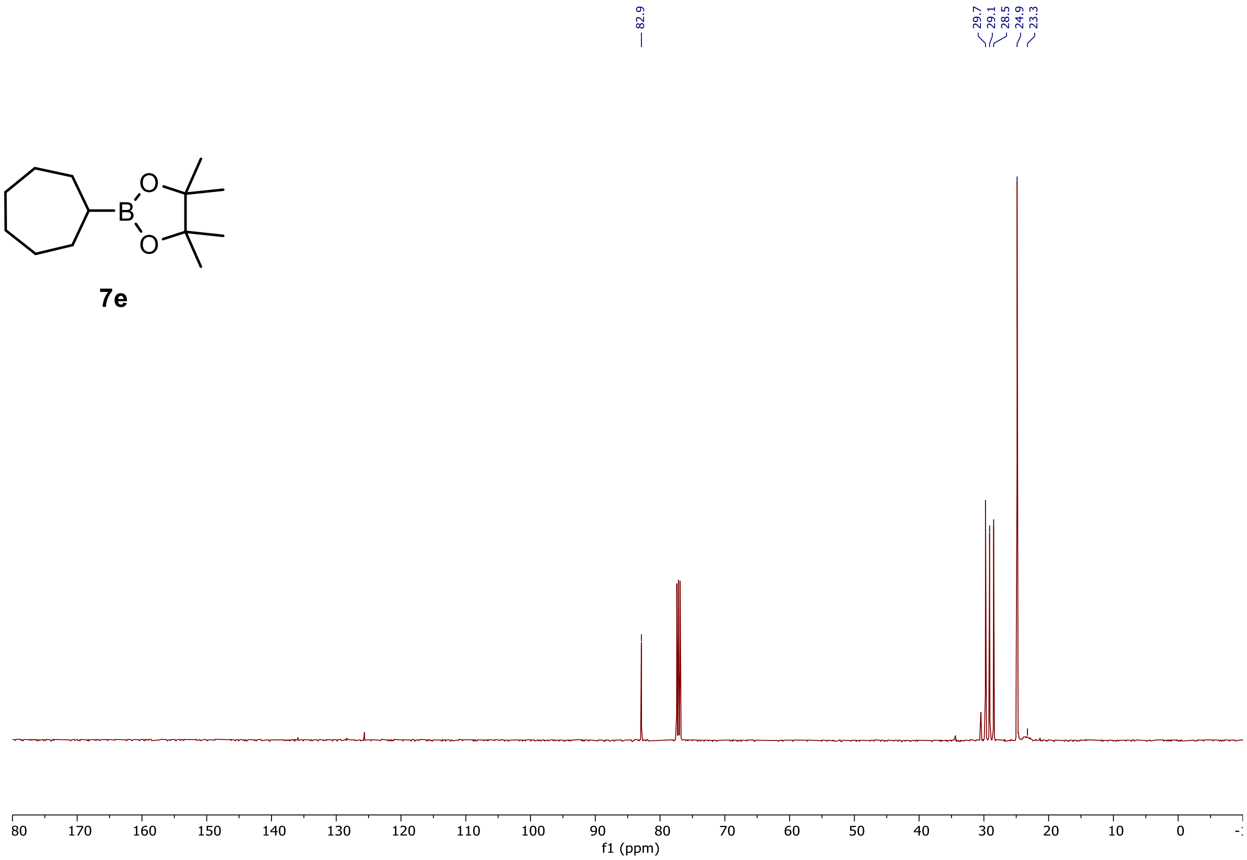

^{1}H NMR (500 MHz, $CDCl_3$) of **7f** (*see procedure*)

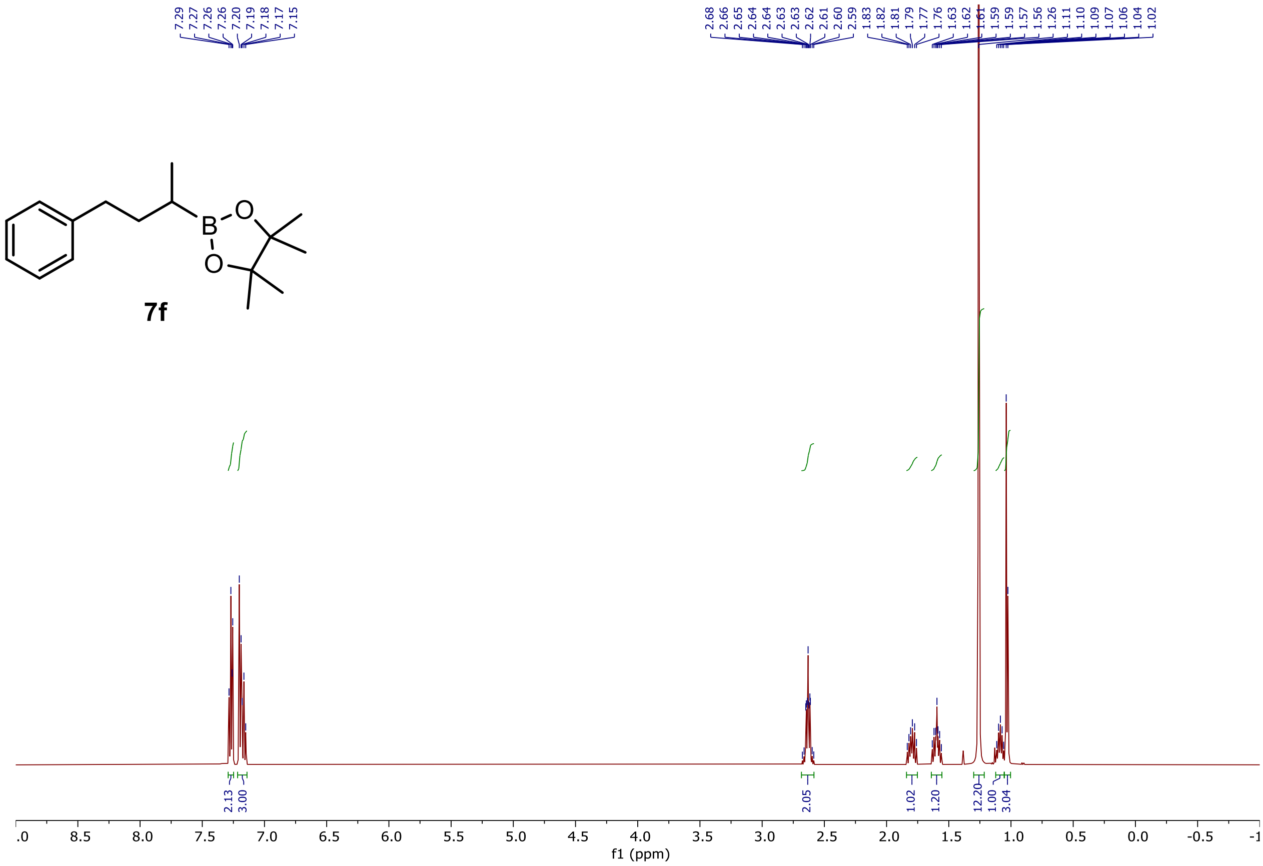

^{13}C NMR (126 MHz, $CDCl_3$) of **7f**

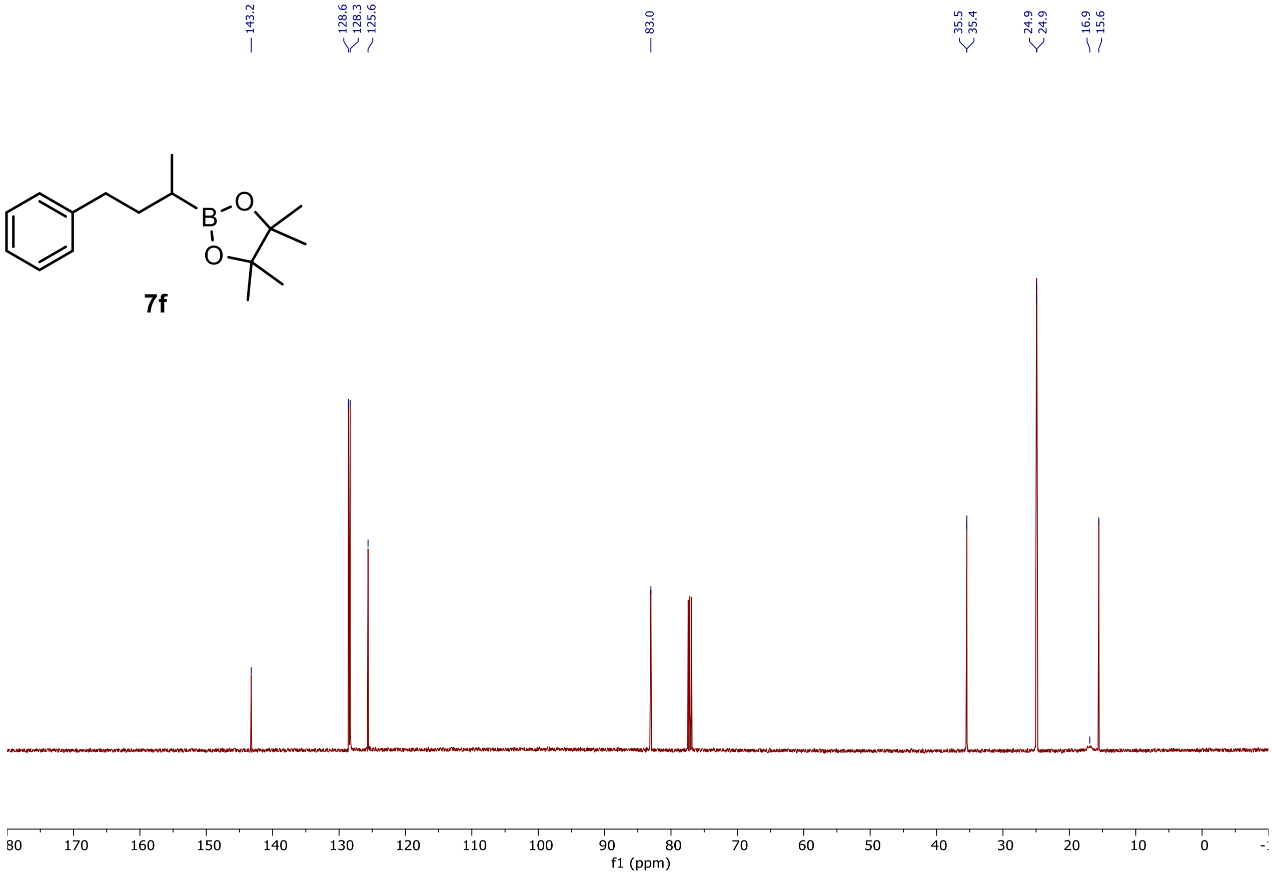

^{1}H NMR (500 MHz, $CDCl_3$) of **7g** (<u>*see procedure*</u>)

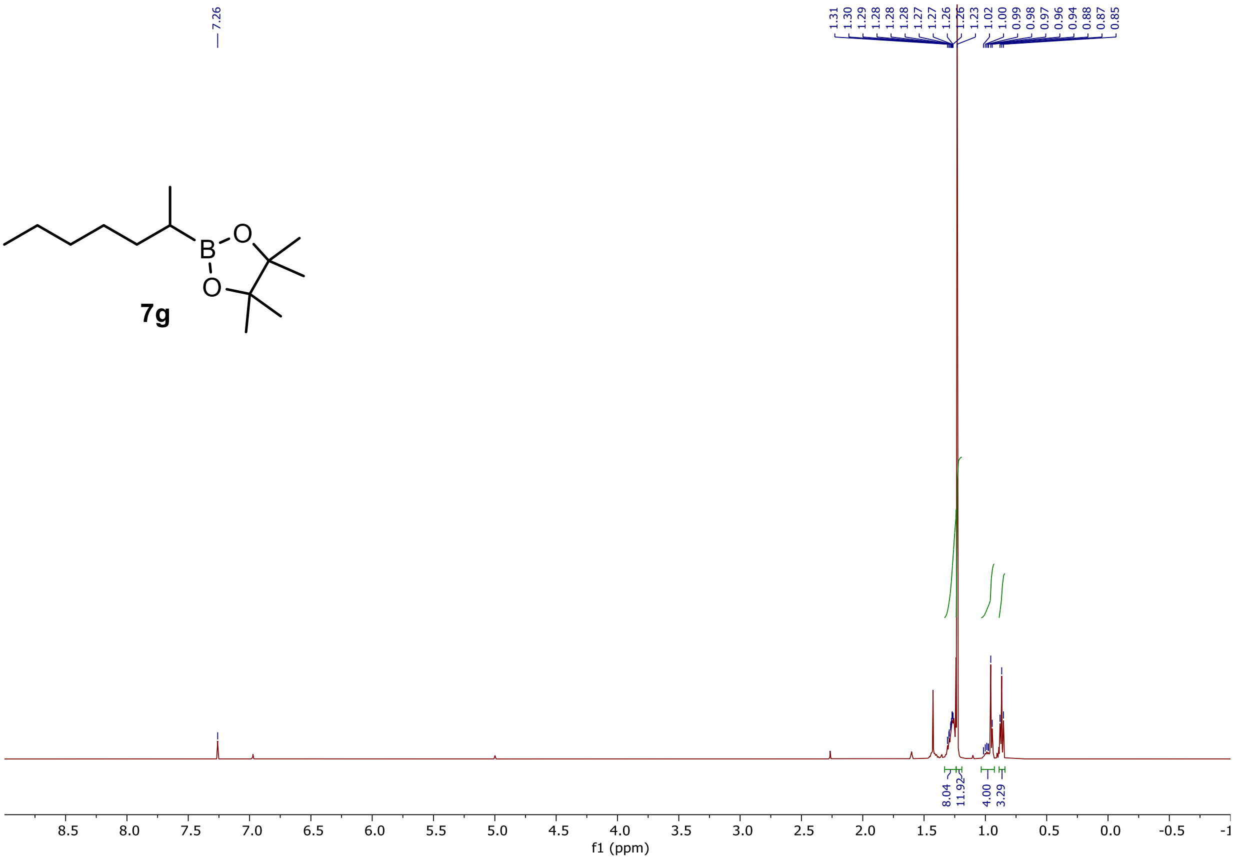

^{13}C NMR (101 MHz, $CDCl_3$) of **7g**

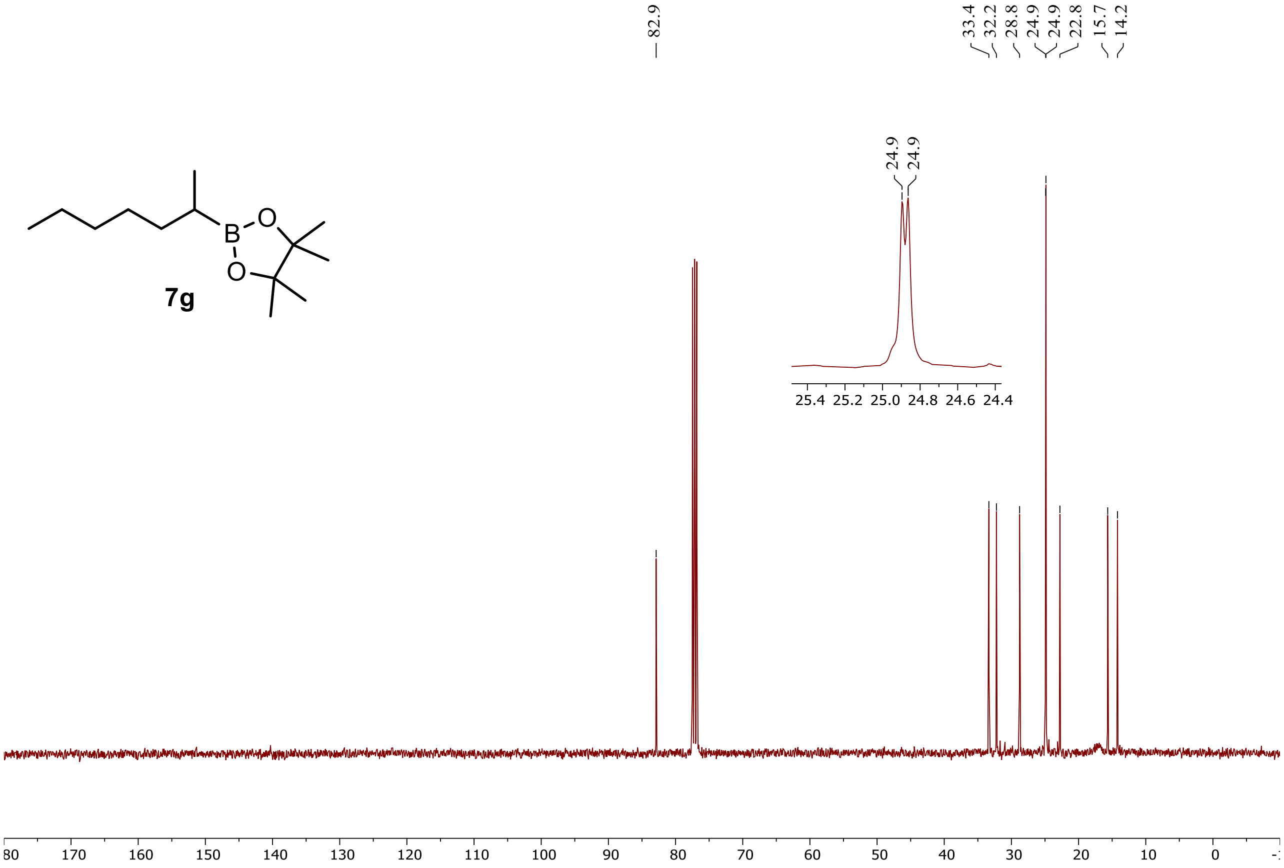

^{11}B NMR (128 MHz, $CDCl_3$) of **7g**

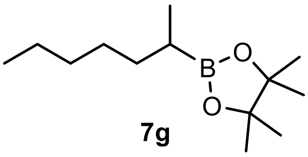

7g

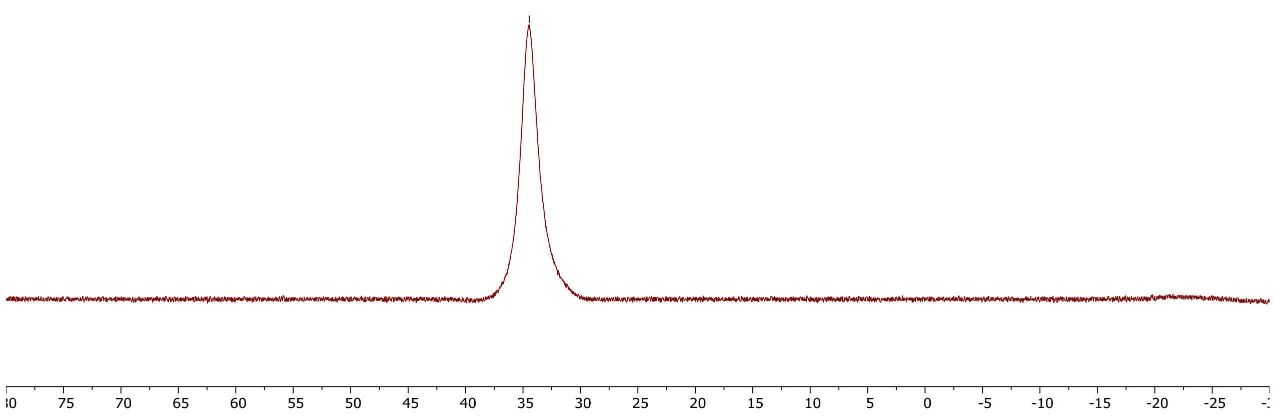

^{1}H NMR (400 MHz, $CDCl_3$) of **7h** (*see procedure*)

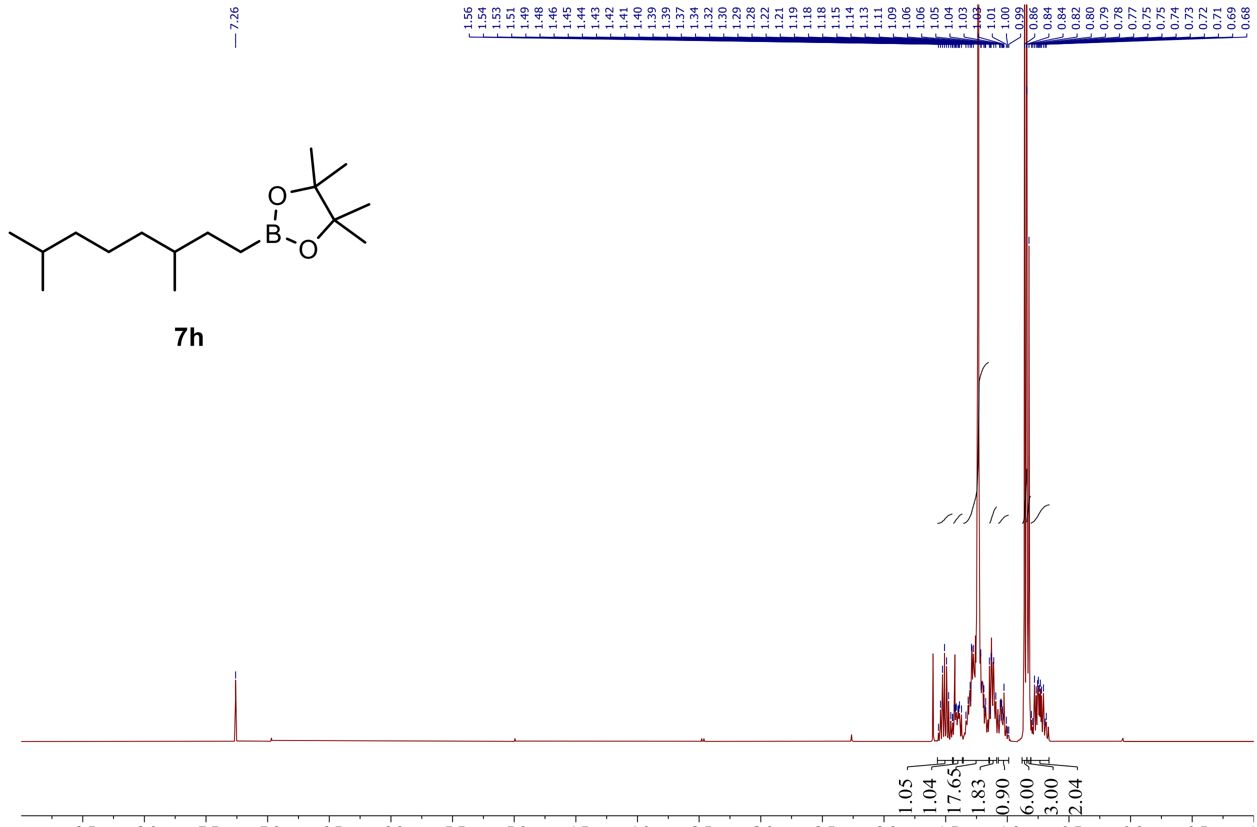

^{13}C NMR (101 MHz, $CDCl_3$) of **7h**

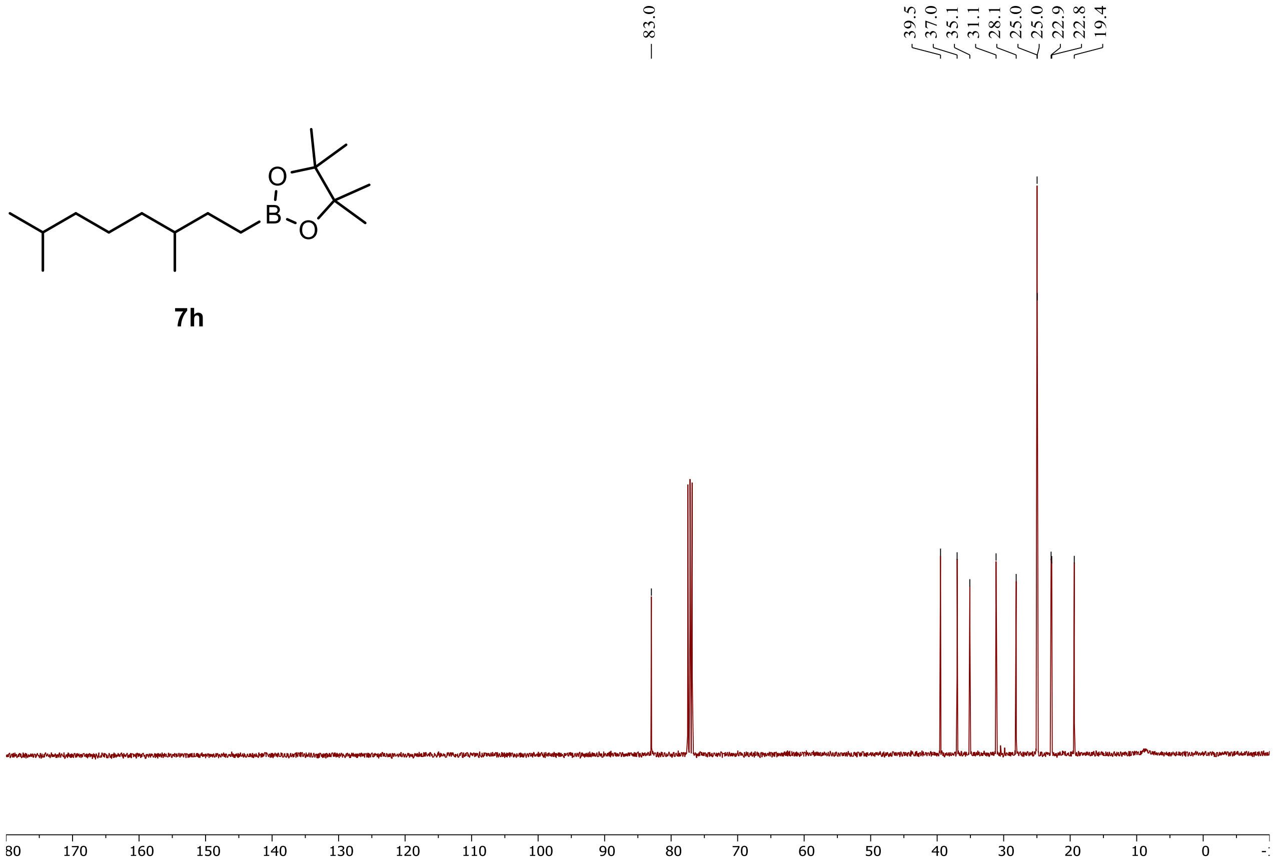

^{11}B NMR (128 MHz, $CDCl_3$) of **7h**

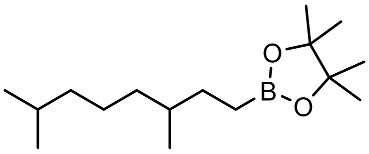

7h

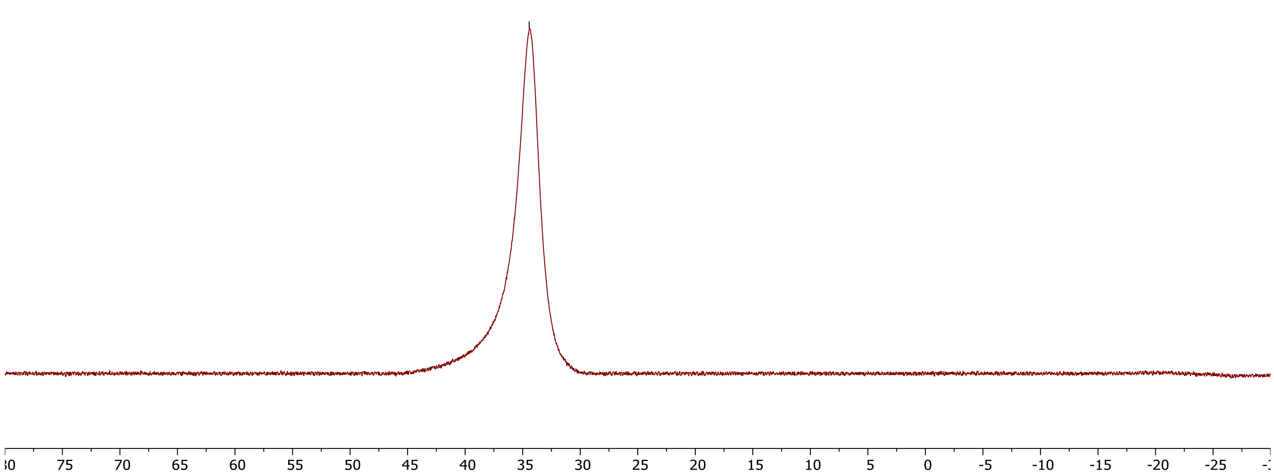

^{1}H NMR (500 MHz, $CDCl_3$) of **7i** (*see procedure*)

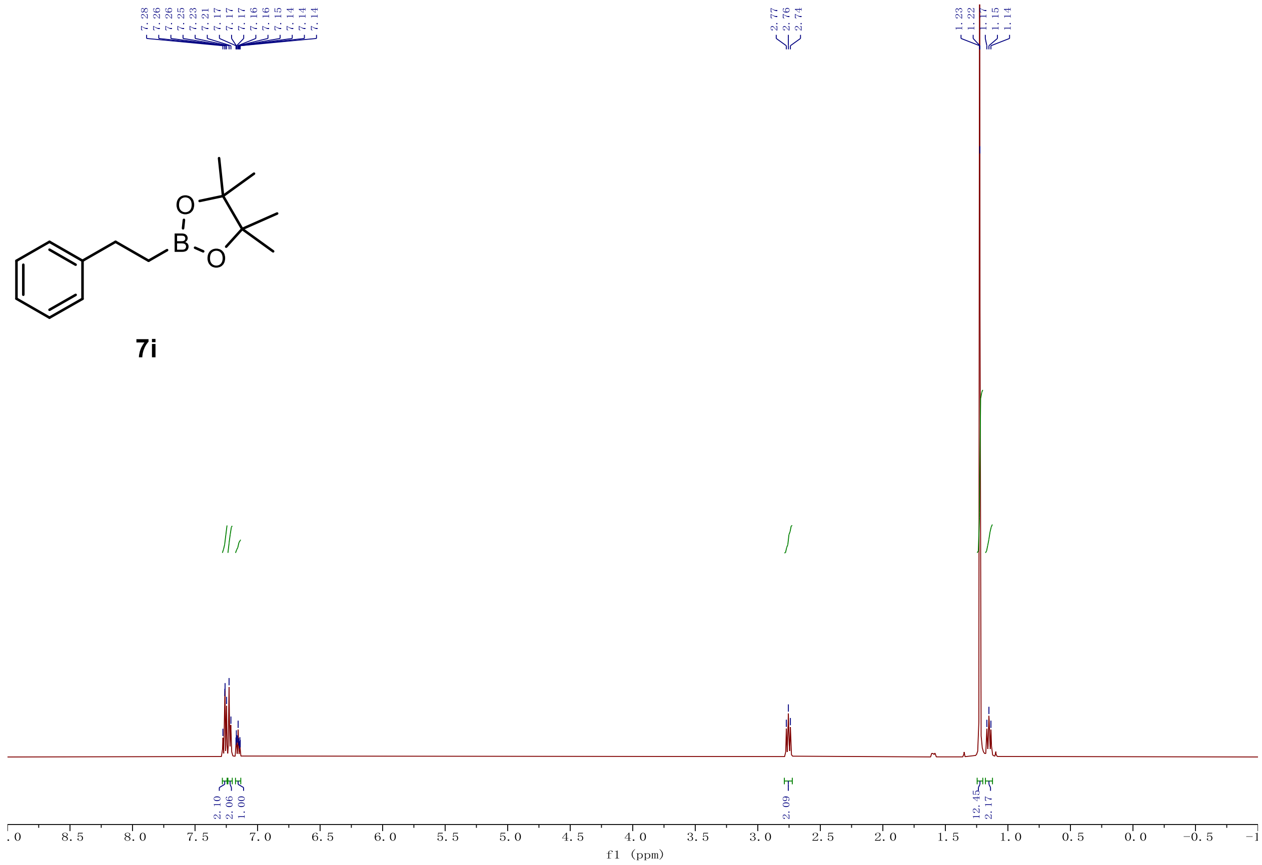

^{13}C NMR (126 MHz, $CDCl_3$) of **7i**

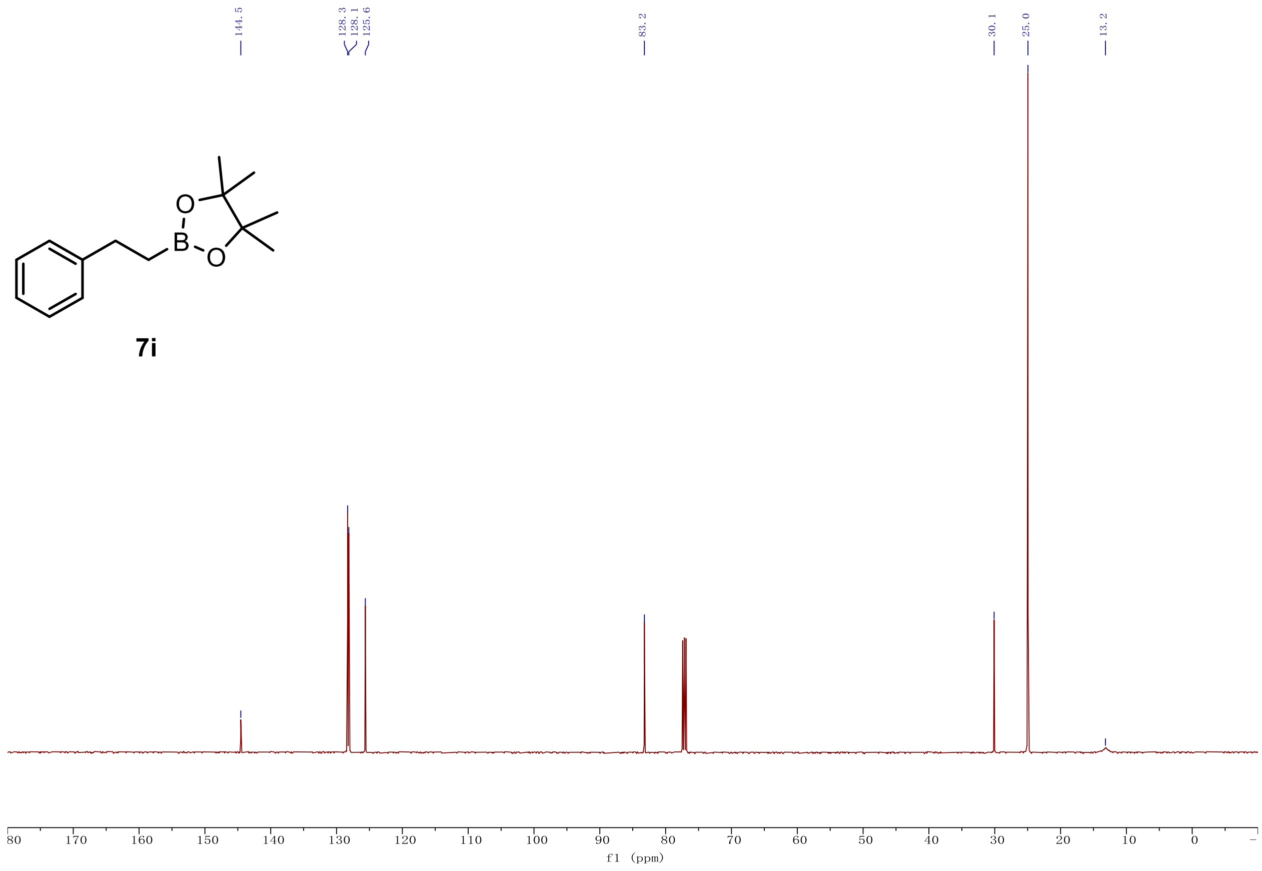

^{1}H NMR (400 MHz, $CDCl_3$) of **7j** (*see procedure*)

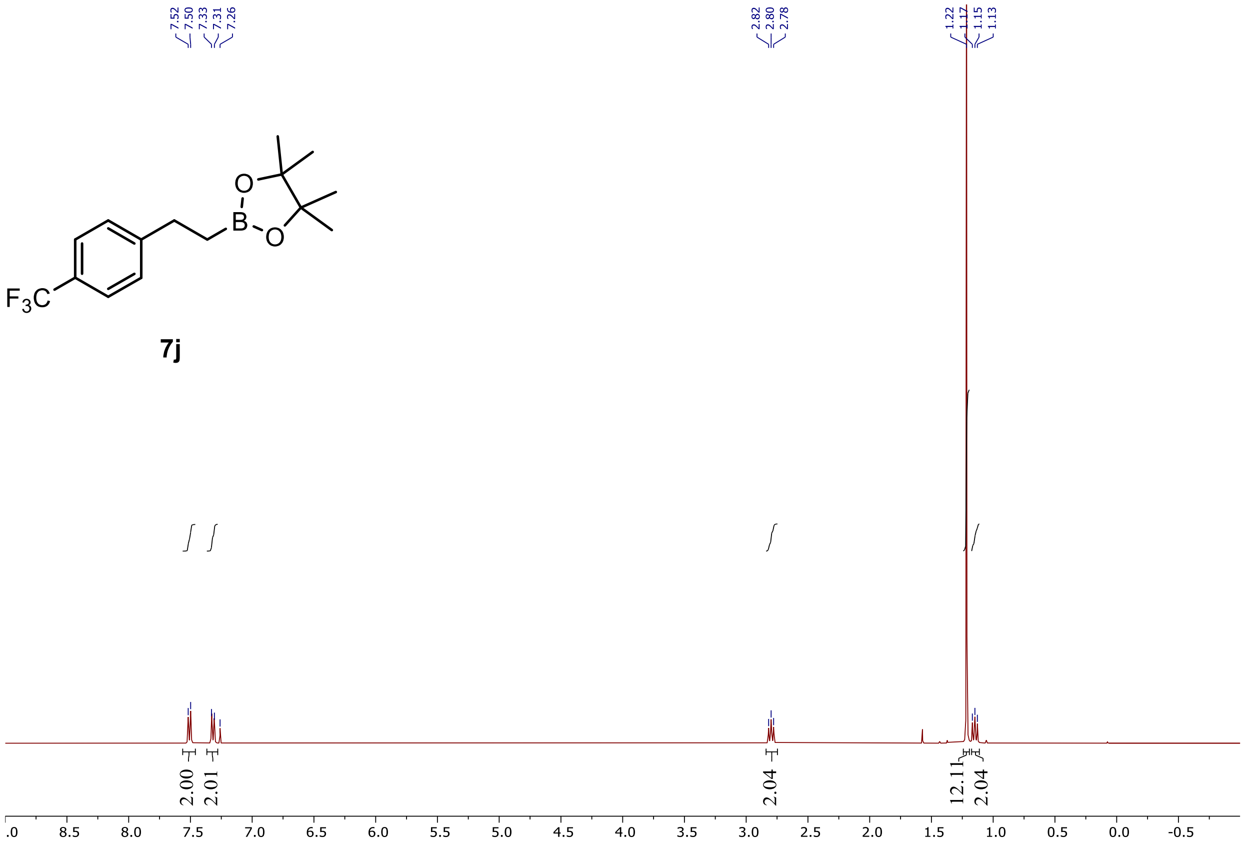

^{13}C NMR (101 MHz, $CDCl_3$) of **7j**

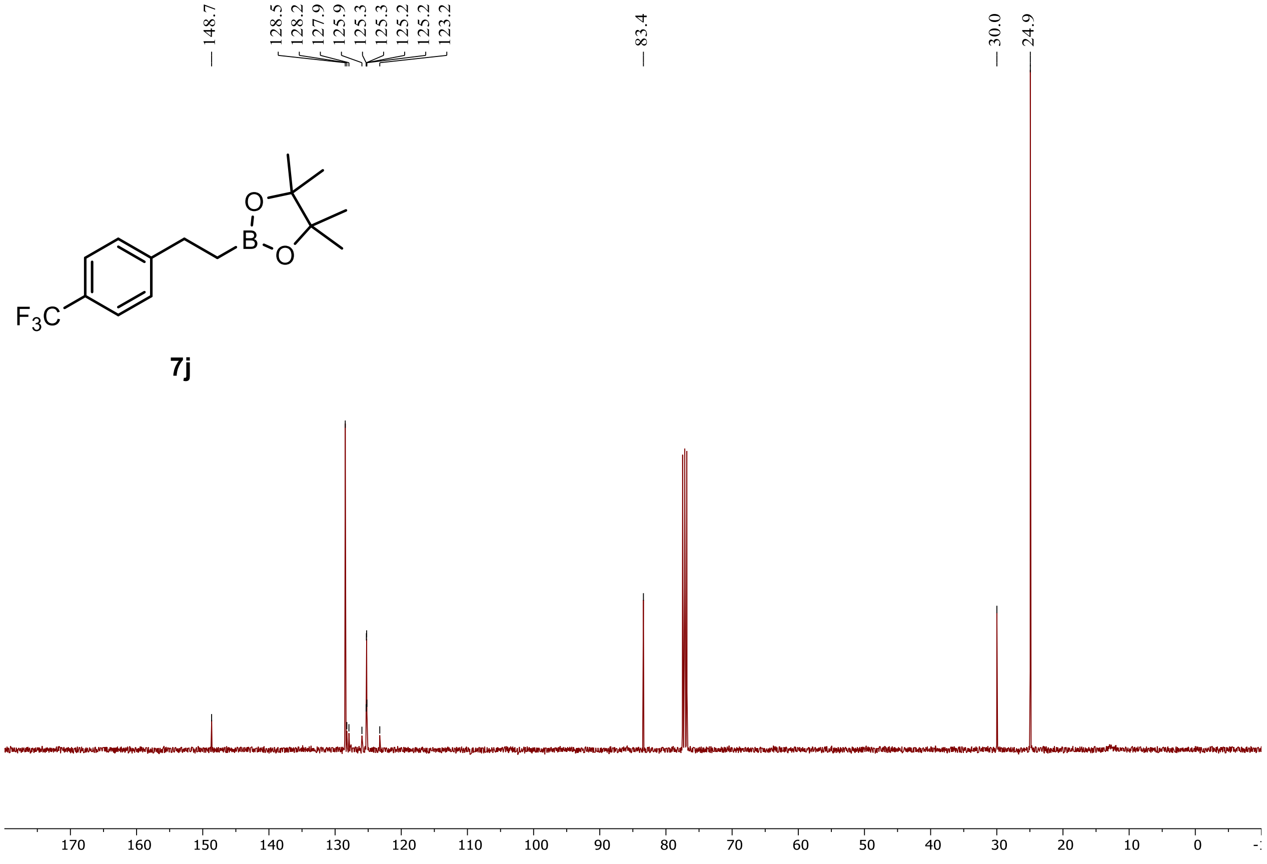

^{19}F NMR (377 MHz, $CDCl_3$) of **7j**

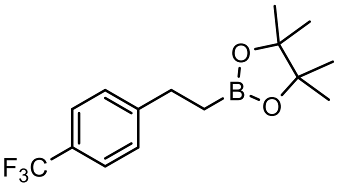

7j

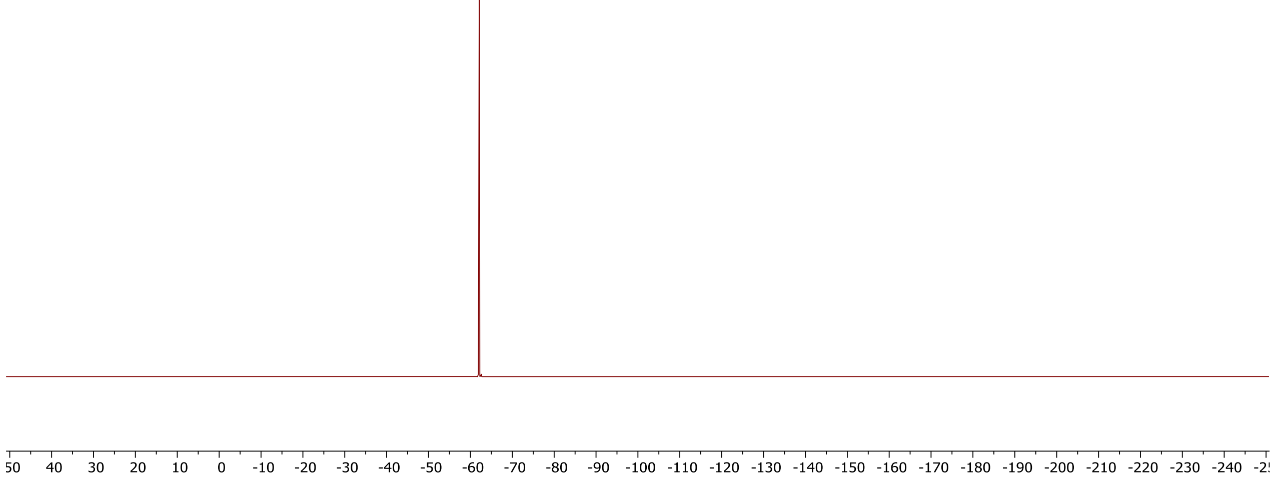

^{11}B NMR (128 MHz, $CDCl_3$) of **7j**

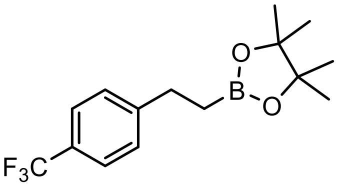

7j

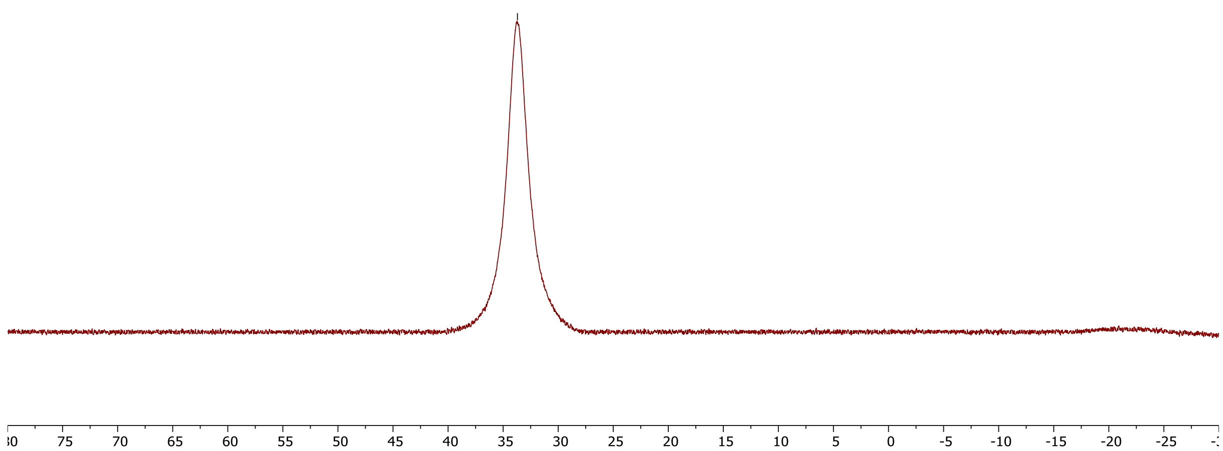

^{1}H NMR (400 MHz, $CDCl_3$) of **7k** (*see procedure*)

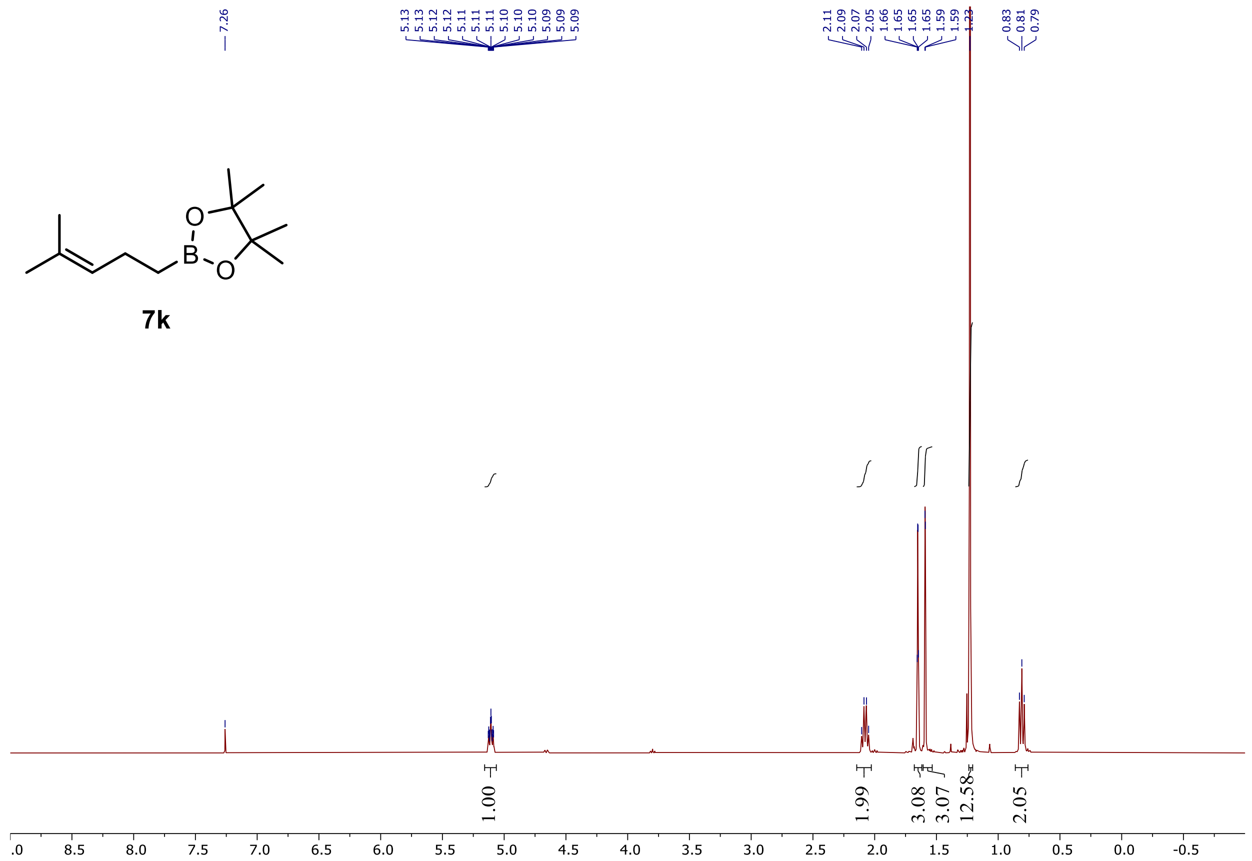

^{13}C NMR (101 MHz, $CDCl_3$) of **7k**

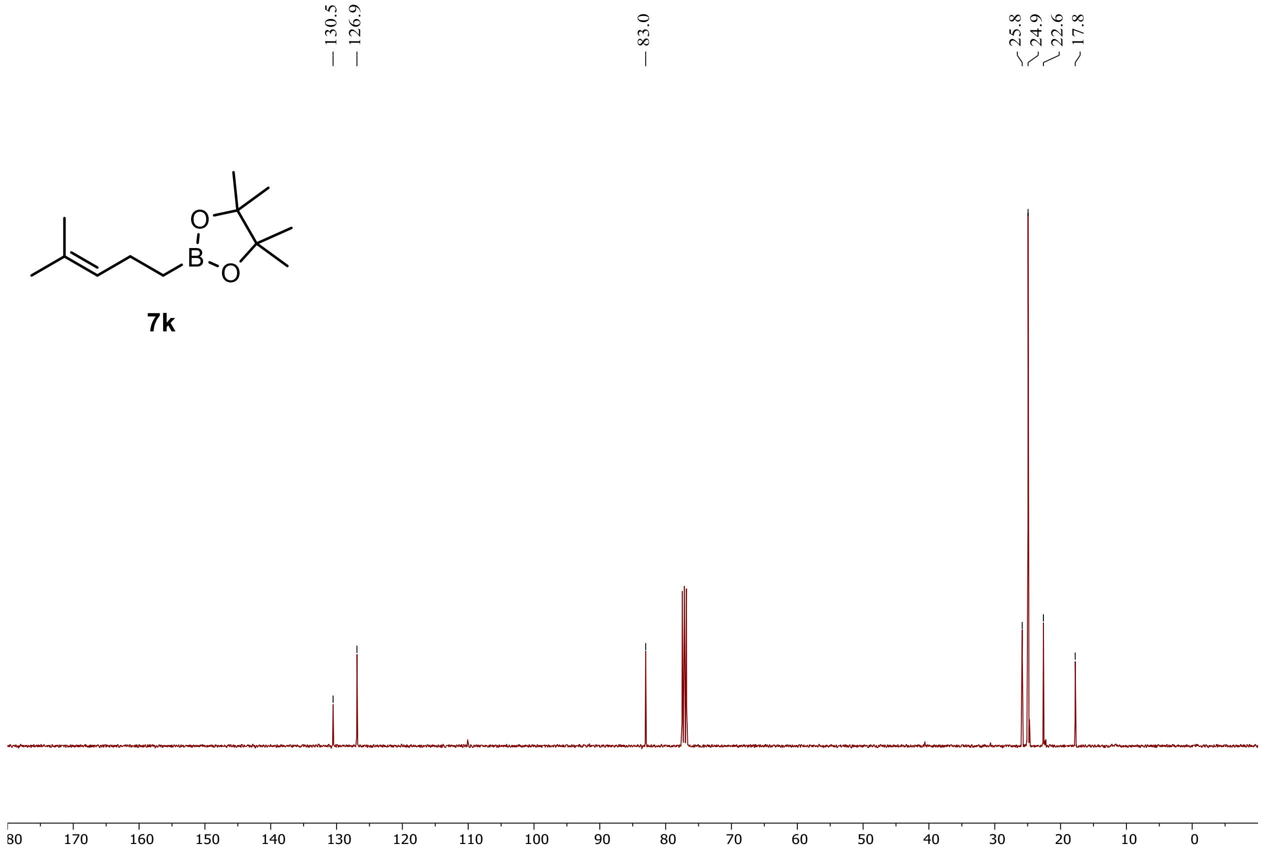

^{11}B NMR (128 MHz, $CDCl_3$) of **7k**

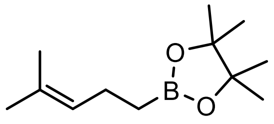

7k

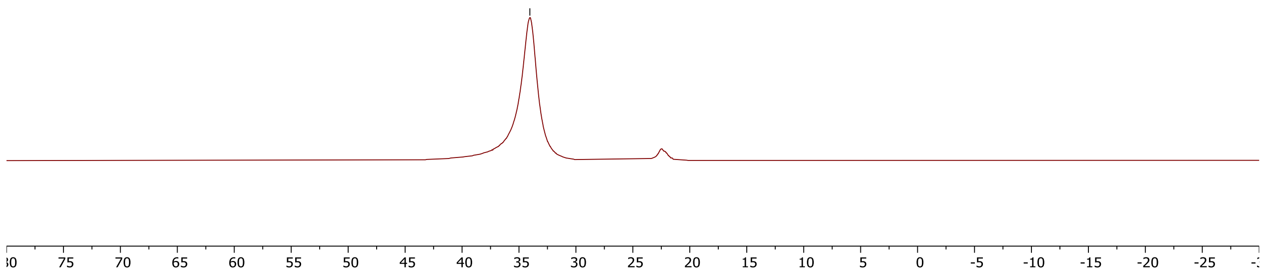

^{1}H NMR (400 MHz, $CDCl_3$) of **7l** (*see procedure*)

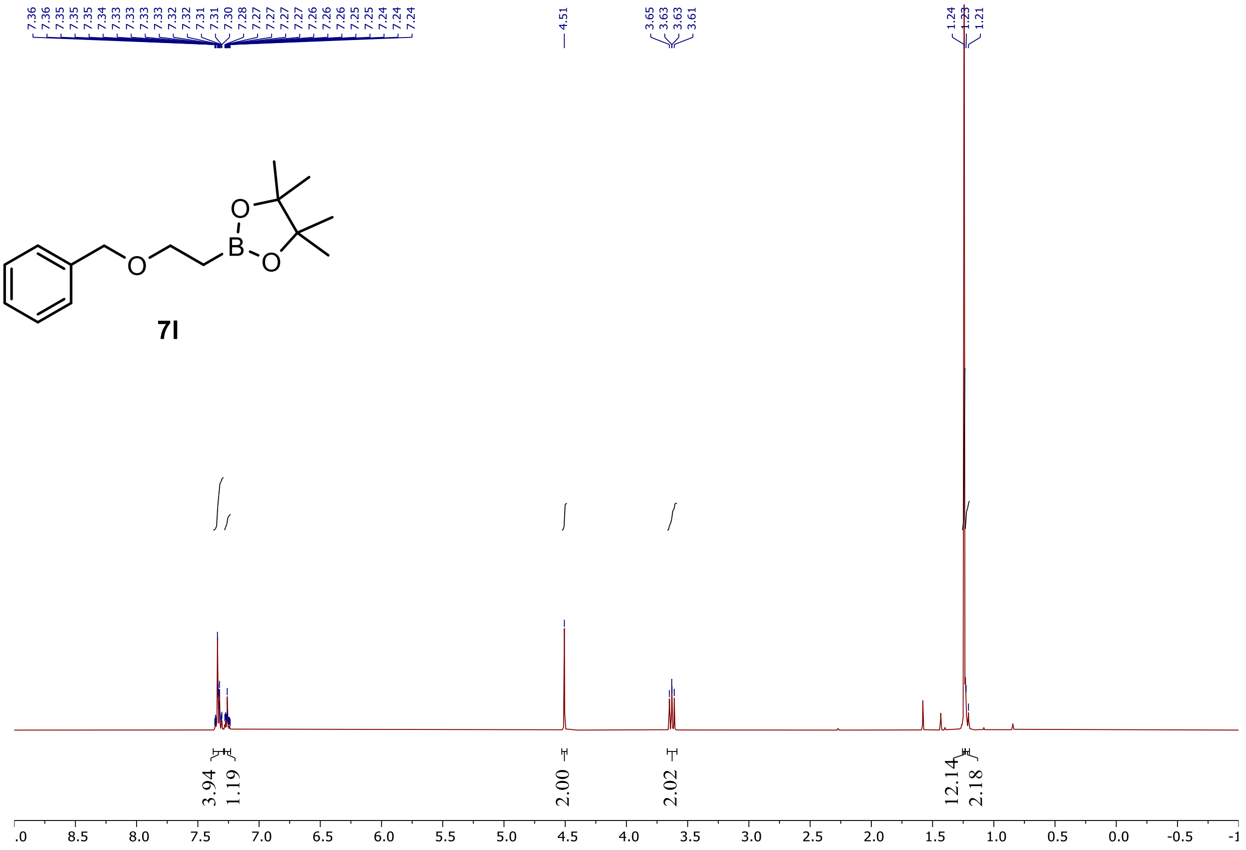

^{13}C NMR (101 MHz, $CDCl_3$) of **7l**

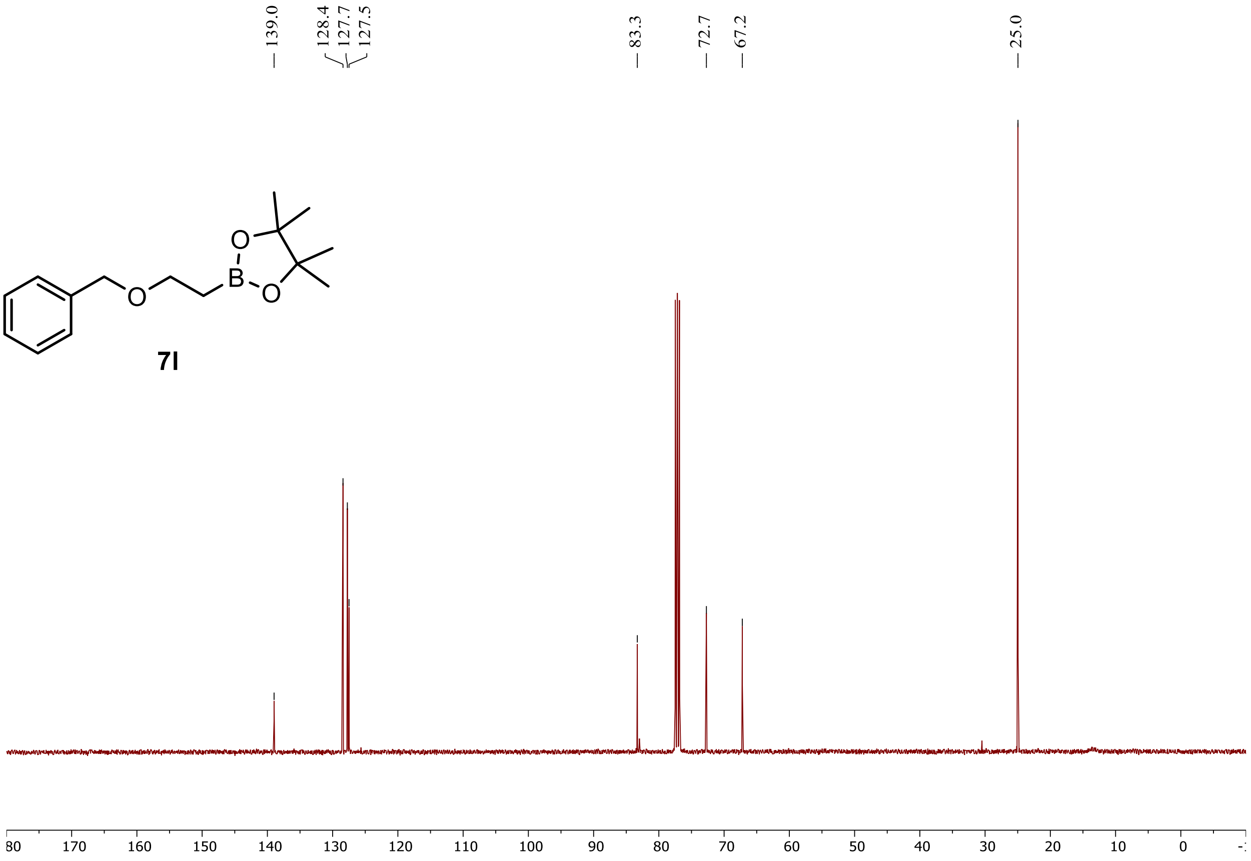

^{11}B NMR (128 MHz, $CDCl_3$) of **7l**

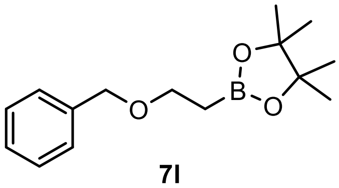

7l

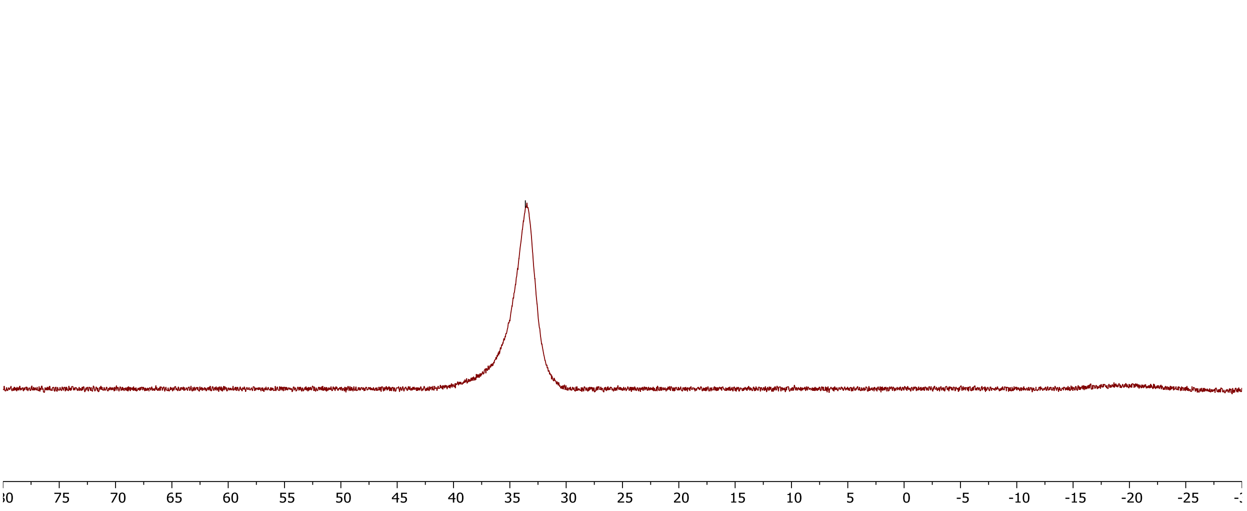

^{1}H NMR (400 MHz, $CDCl_3$) of **7m** (*see procedure*)

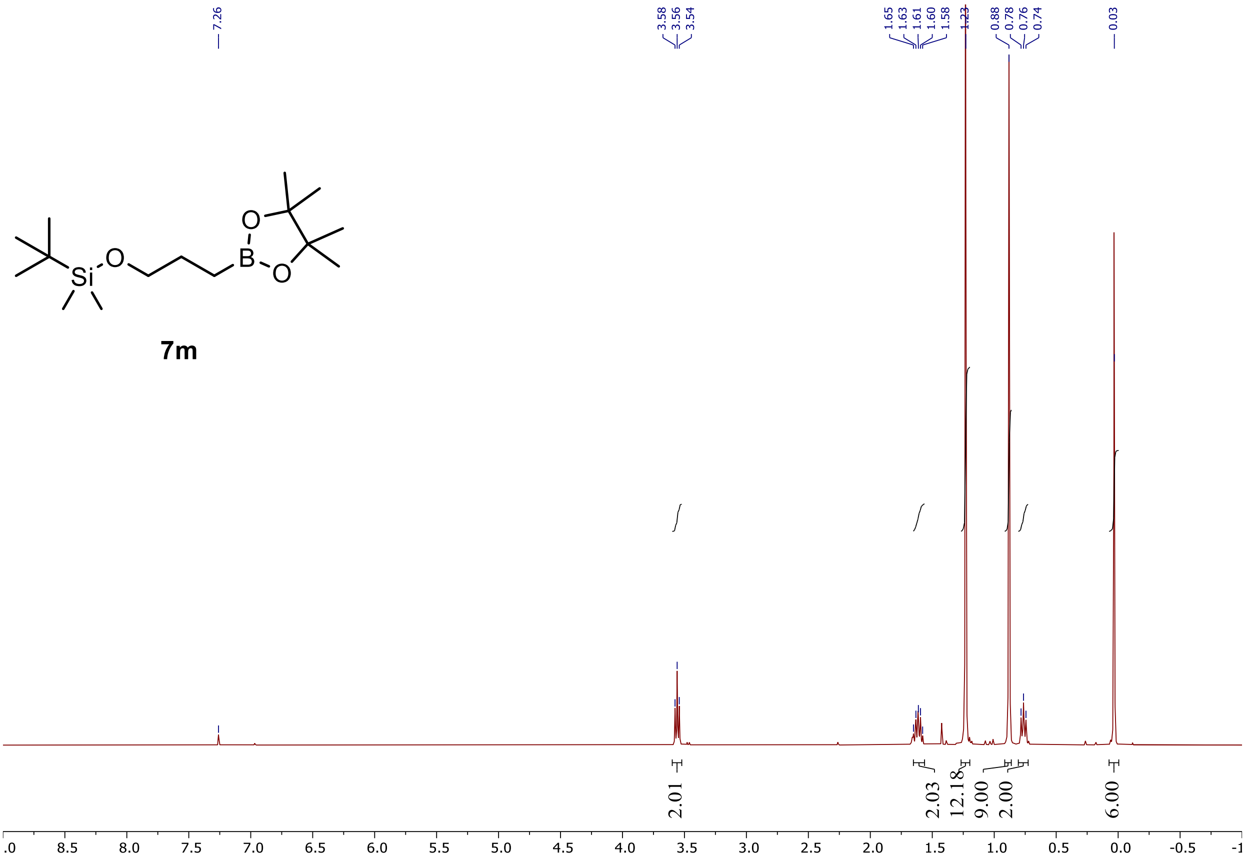

^{13}C NMR (101 MHz, $CDCl_3$) of **7m**

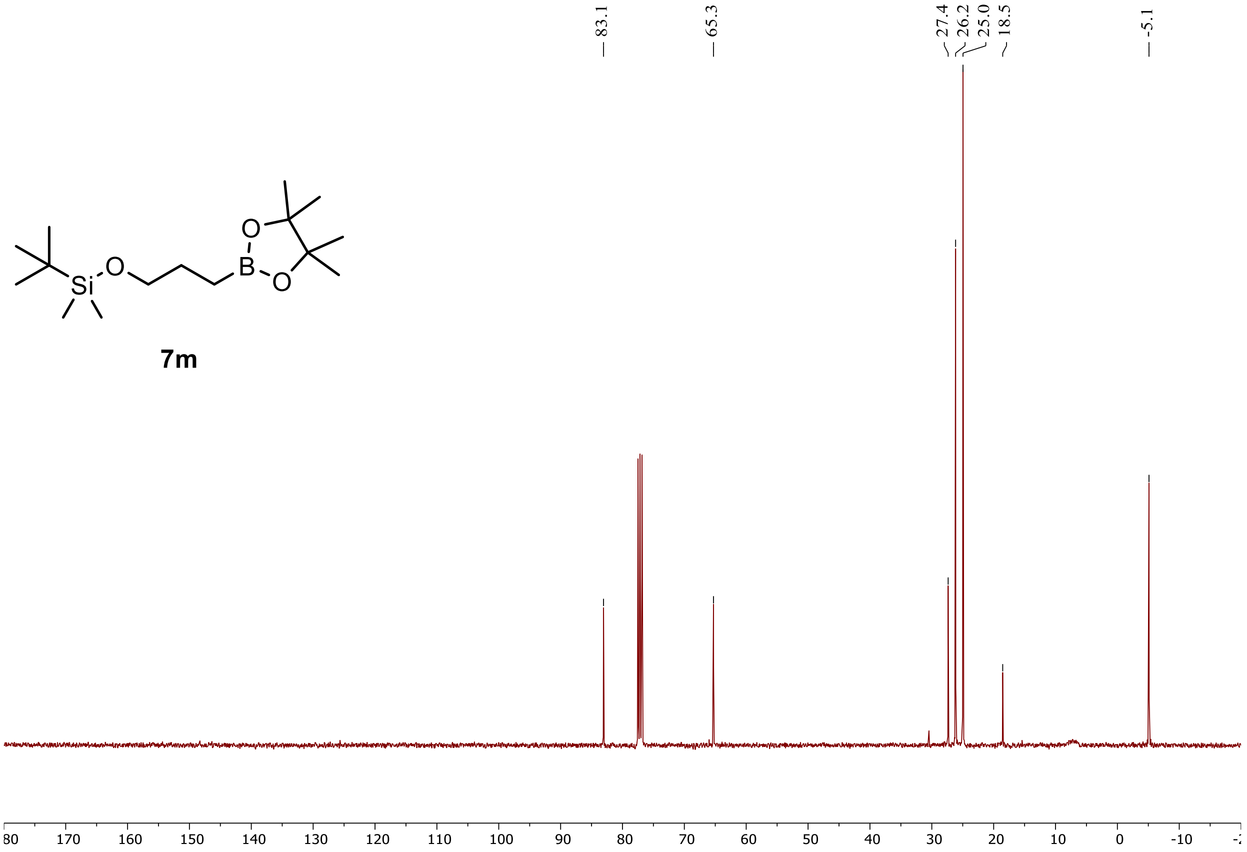

^{1}H NMR (400 MHz, $CDCl_3$) of **7n** (<u>see procedure</u>)

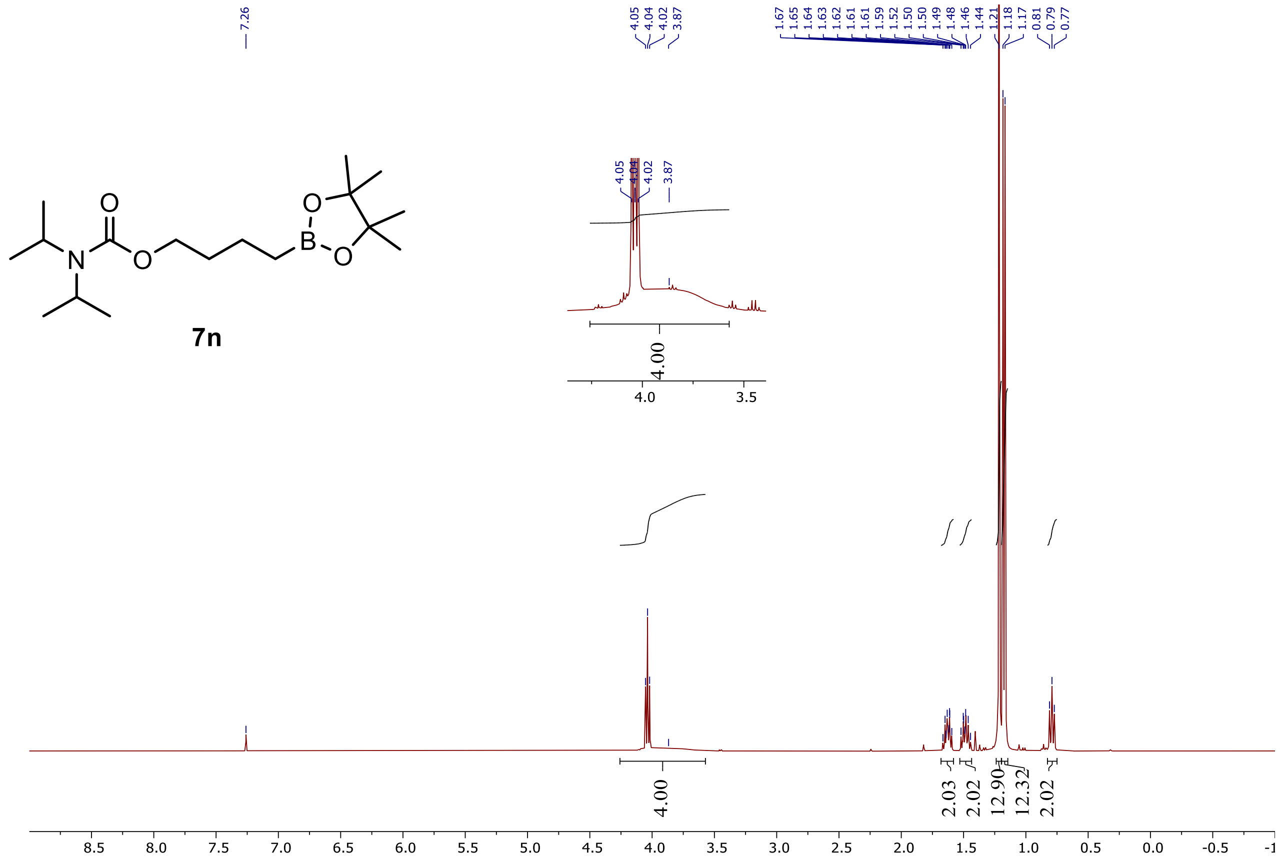

^{13}C NMR (101 MHz, $CDCl_3$) of **7n**

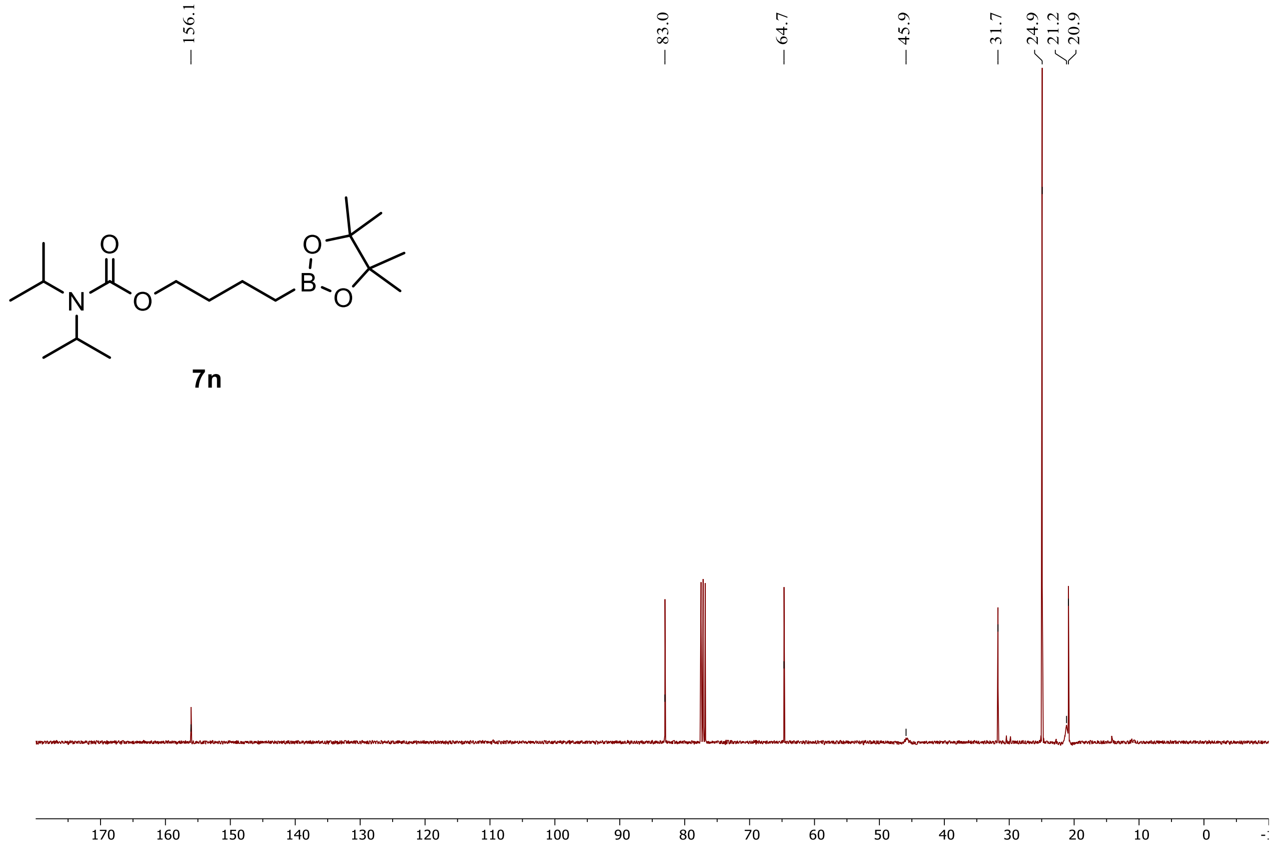

^{11}B NMR (128 MHz, $CDCl_3$) of **7n**

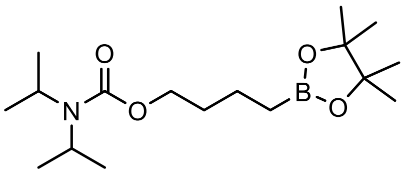

7n

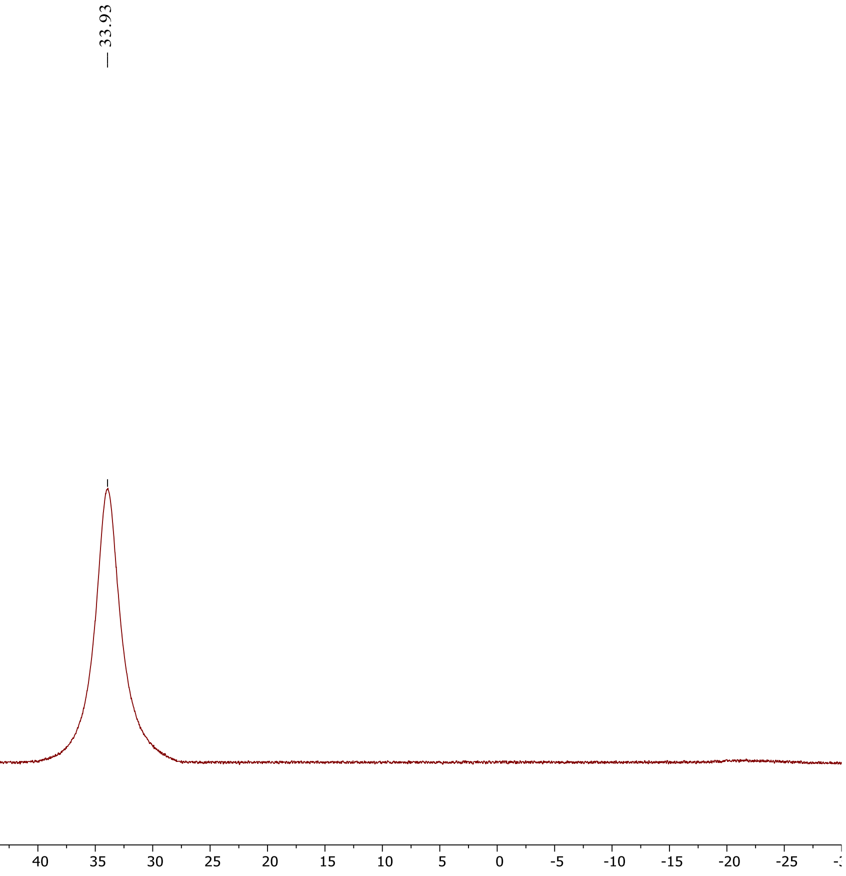

^{1}H NMR (400 MHz, $CDCl_3$) of **7o** (*see procedure*)

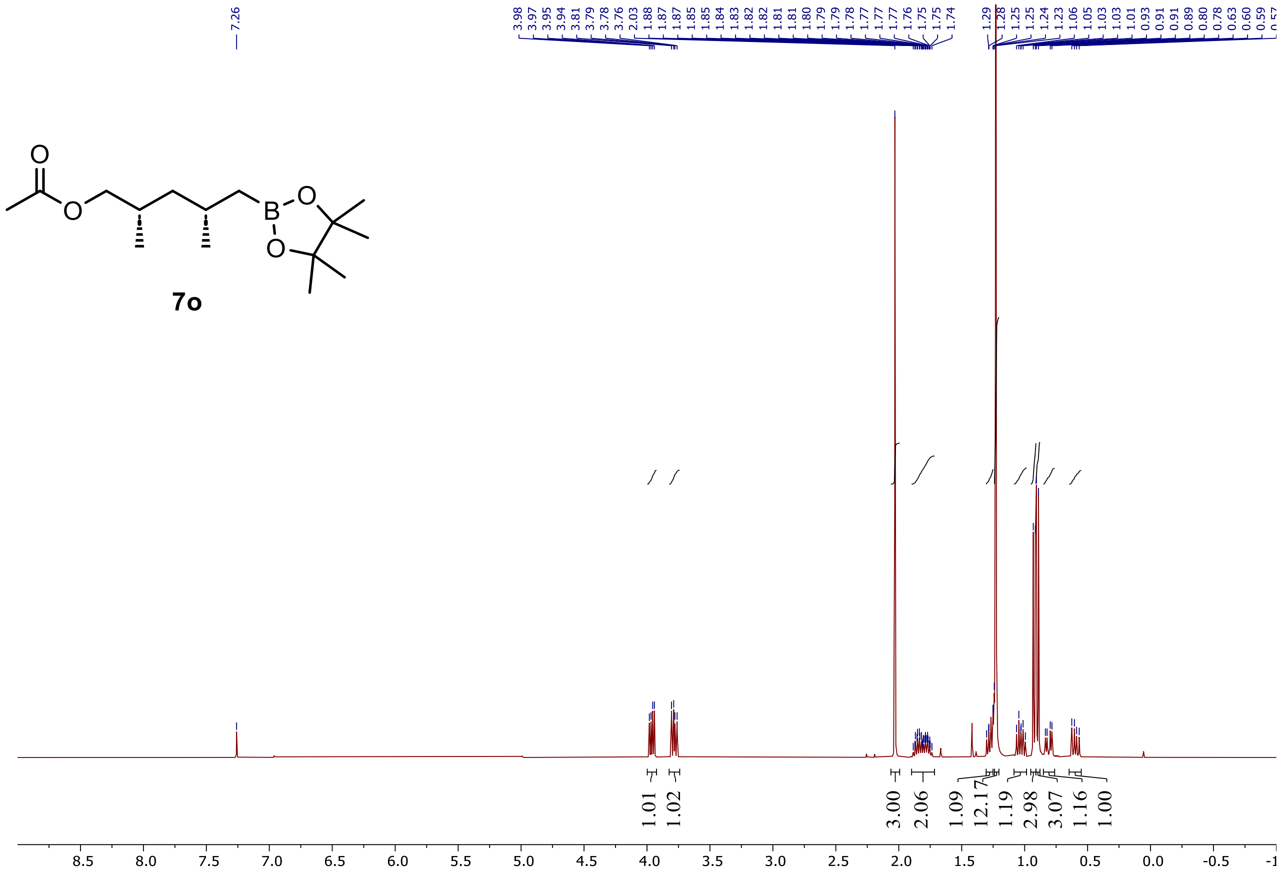

7o

^{13}C NMR (126 MHz, $CDCl_3$) of **7o**

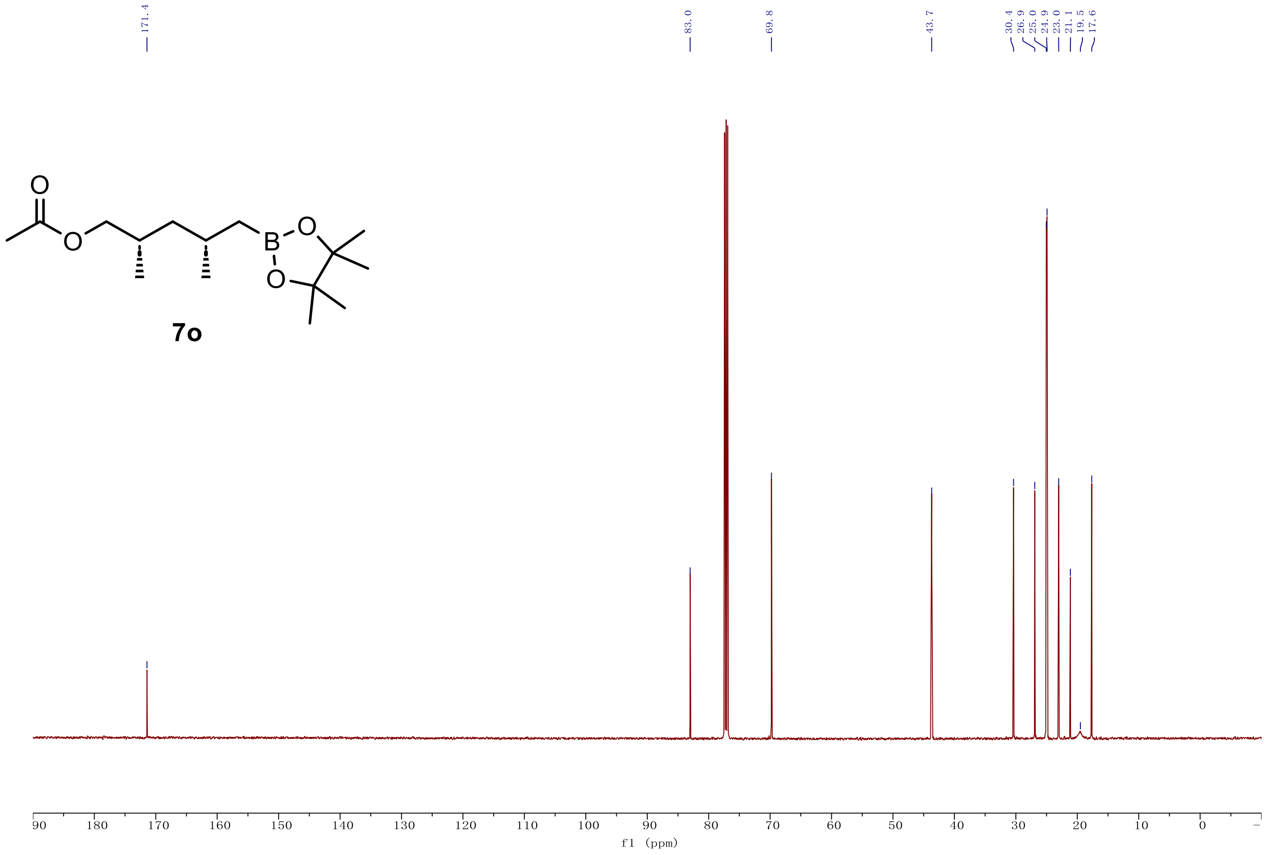

7o

^{11}B NMR (128 MHz, $CDCl_3$) of **7o**

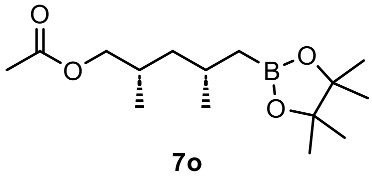

7o

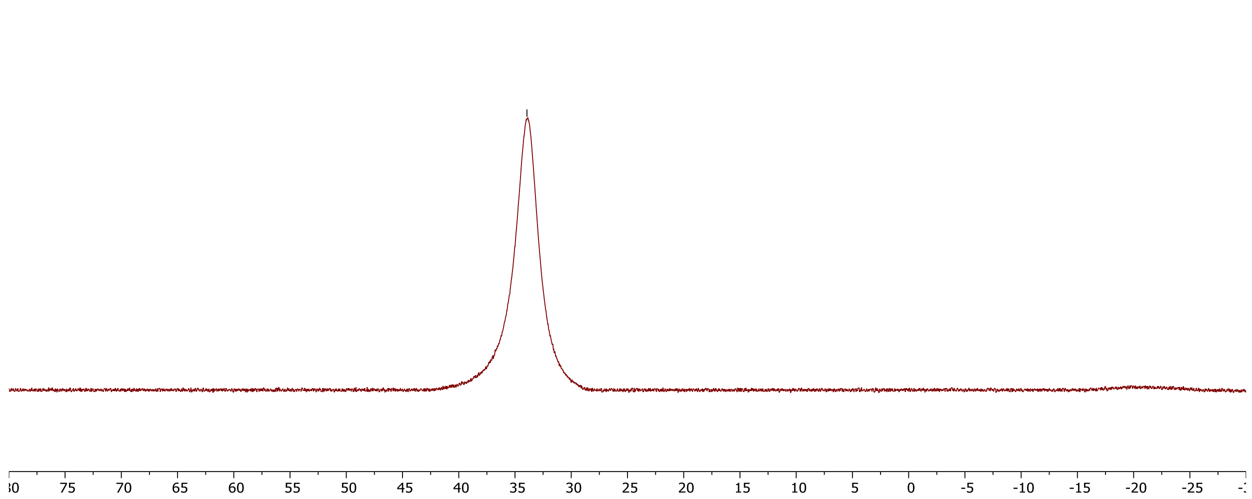

^{1}H NMR (400 MHz, $CDCl_3$) of **7p** (see procedure)

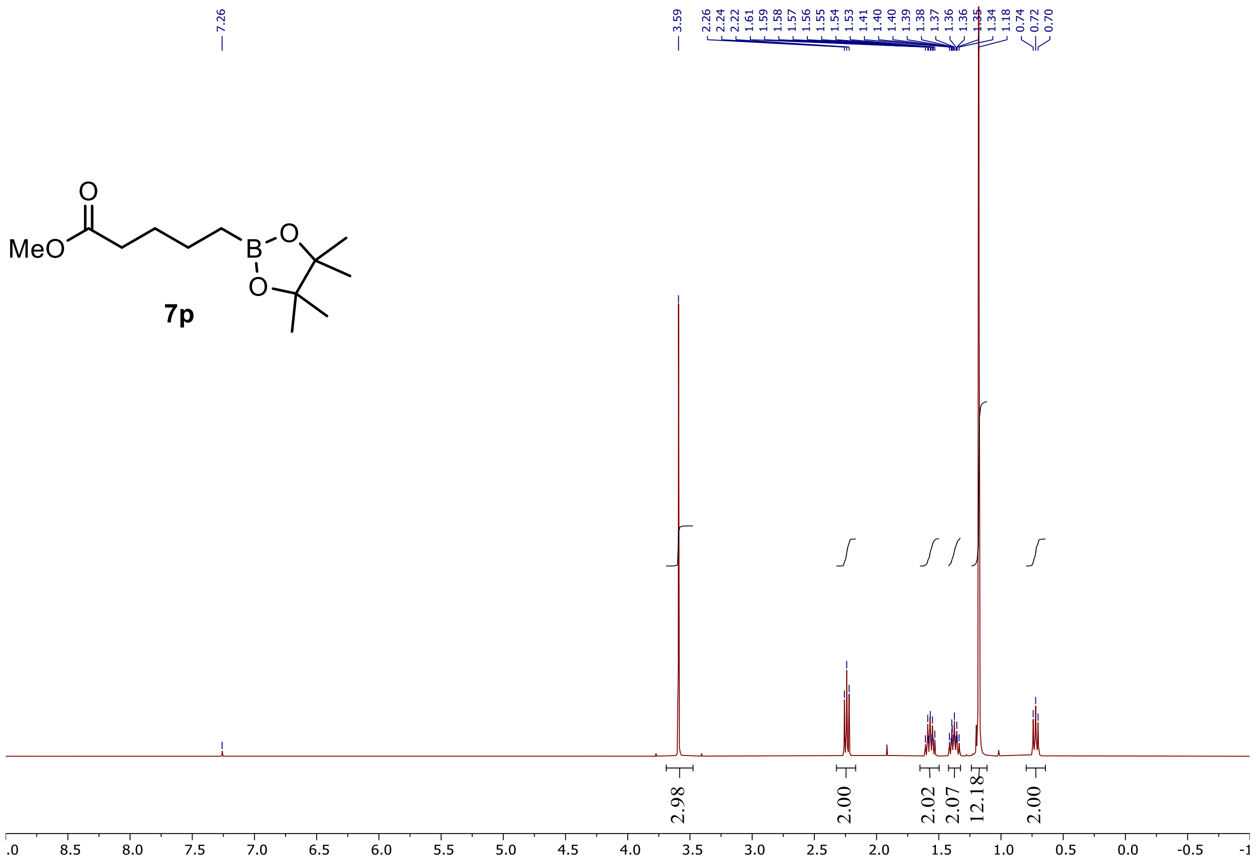

^{13}C NMR (101 MHz, $CDCl_3$) of **7p**

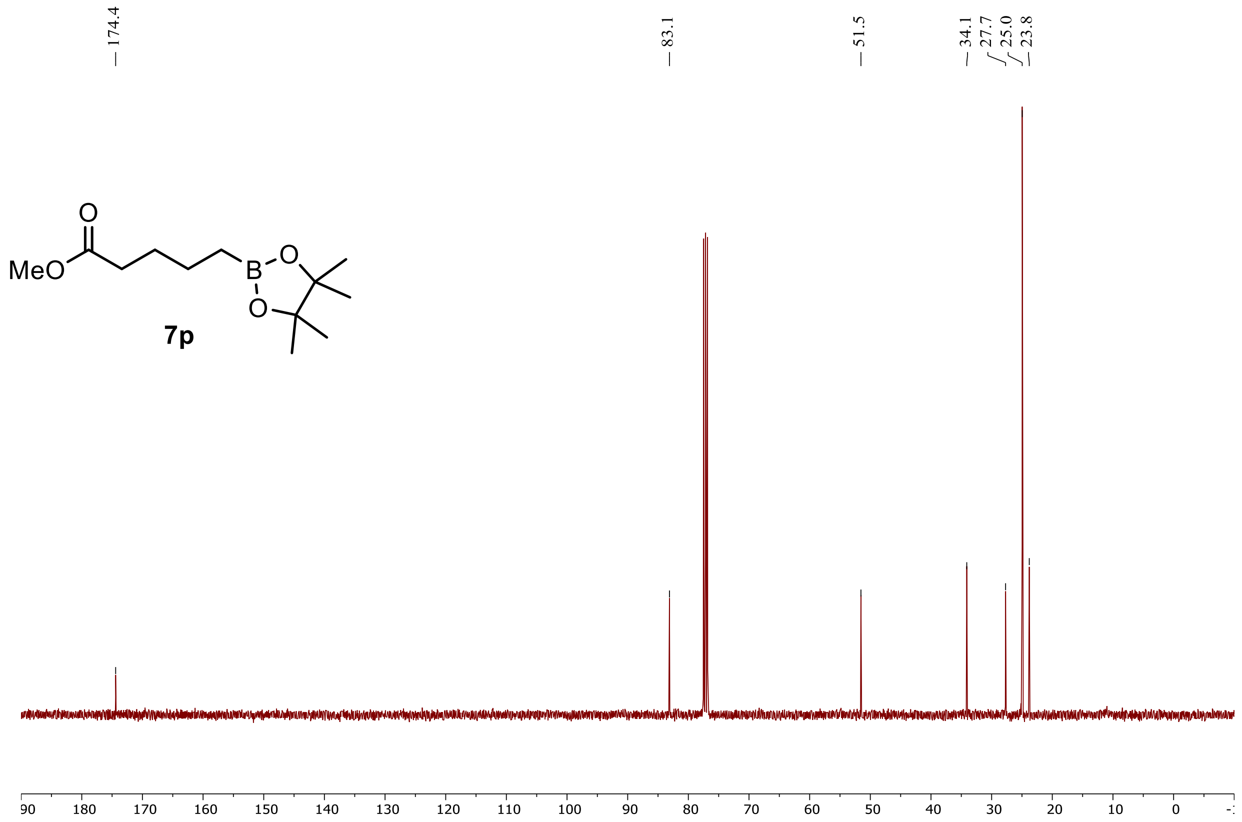

^{11}B NMR (128 MHz, $CDCl_3$) of **7p**

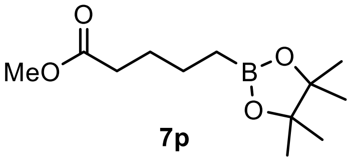

7p

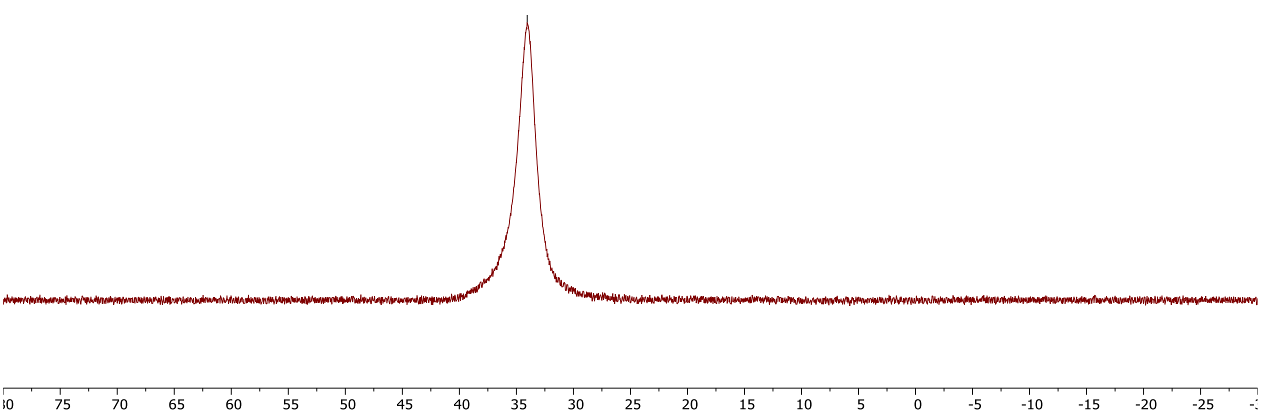

^{1}H NMR (400 MHz, $CDCl_3$) of **7q** (*see procedure*)

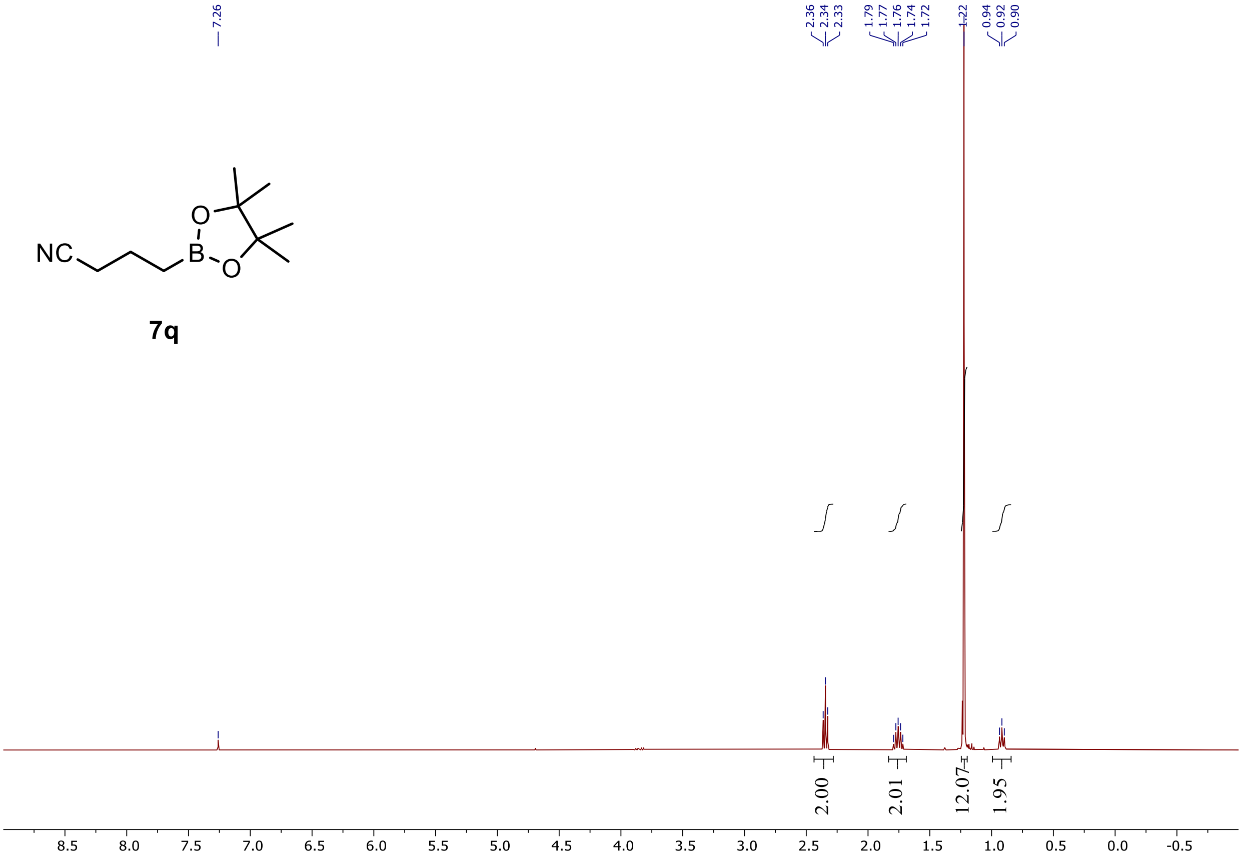

^{13}C NMR (101 MHz, $CDCl_3$) of **7q**

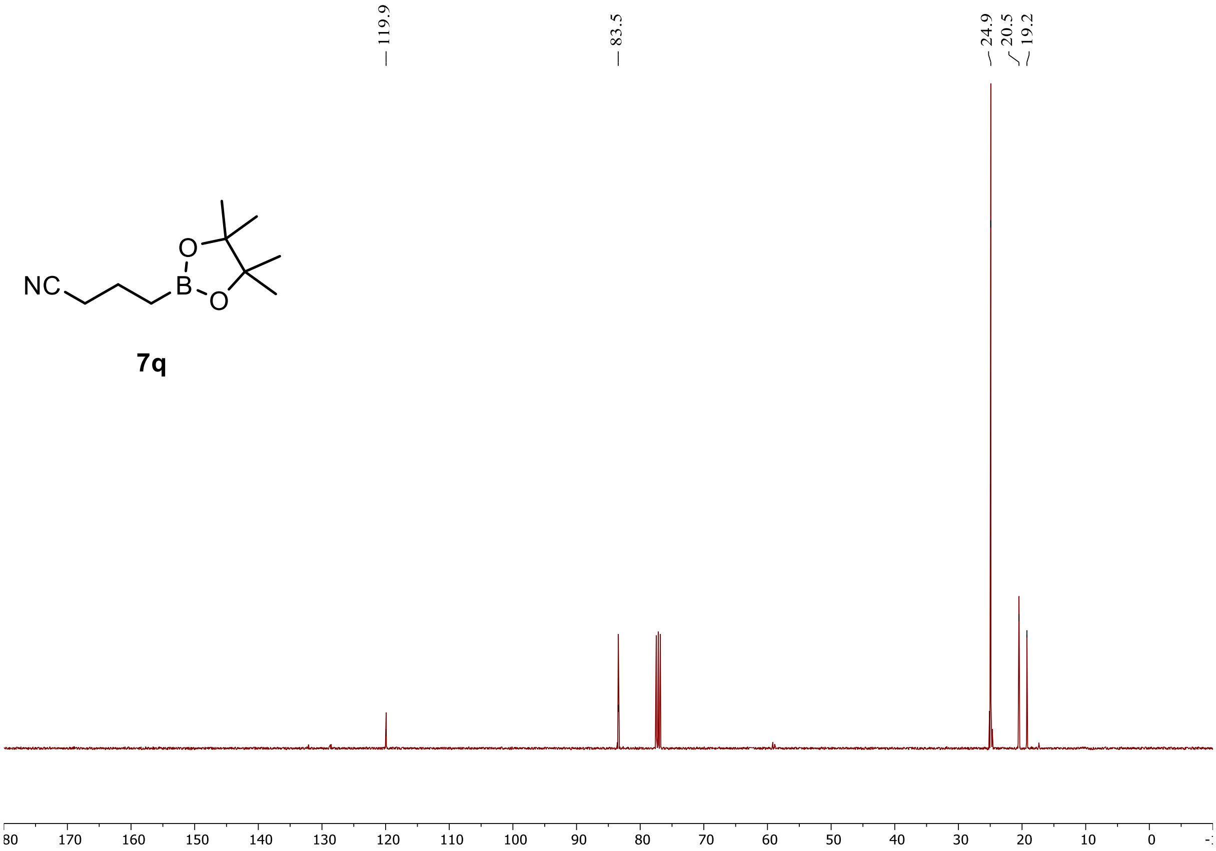

^{11}B NMR (128 MHz, $CDCl_3$) of **7q**

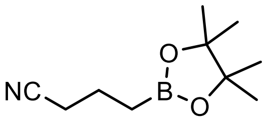

7q

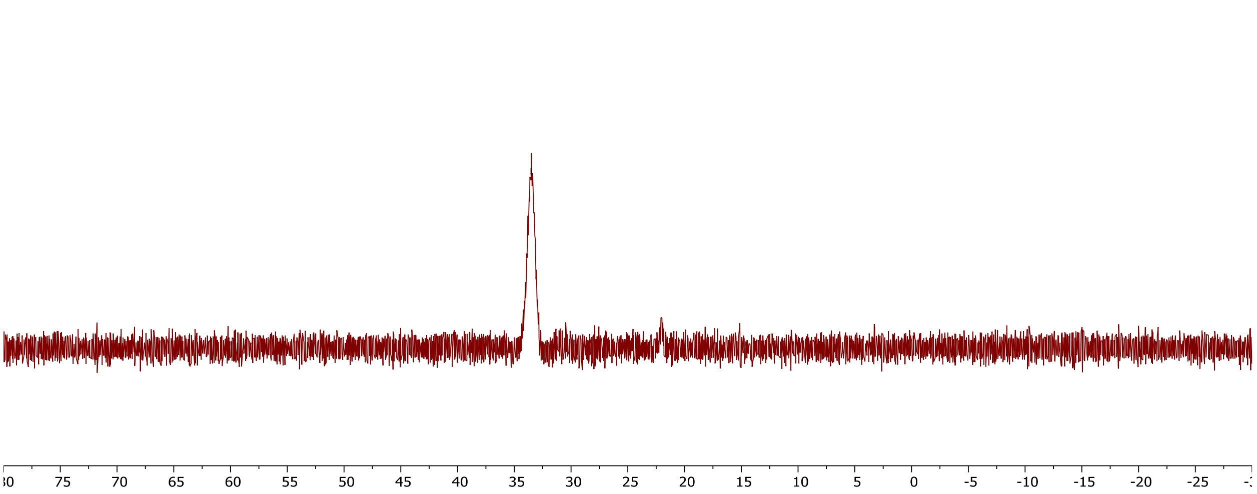

^{1}H NMR (400 MHz, $CDCl_3$) of **7r** (*see procedure*)

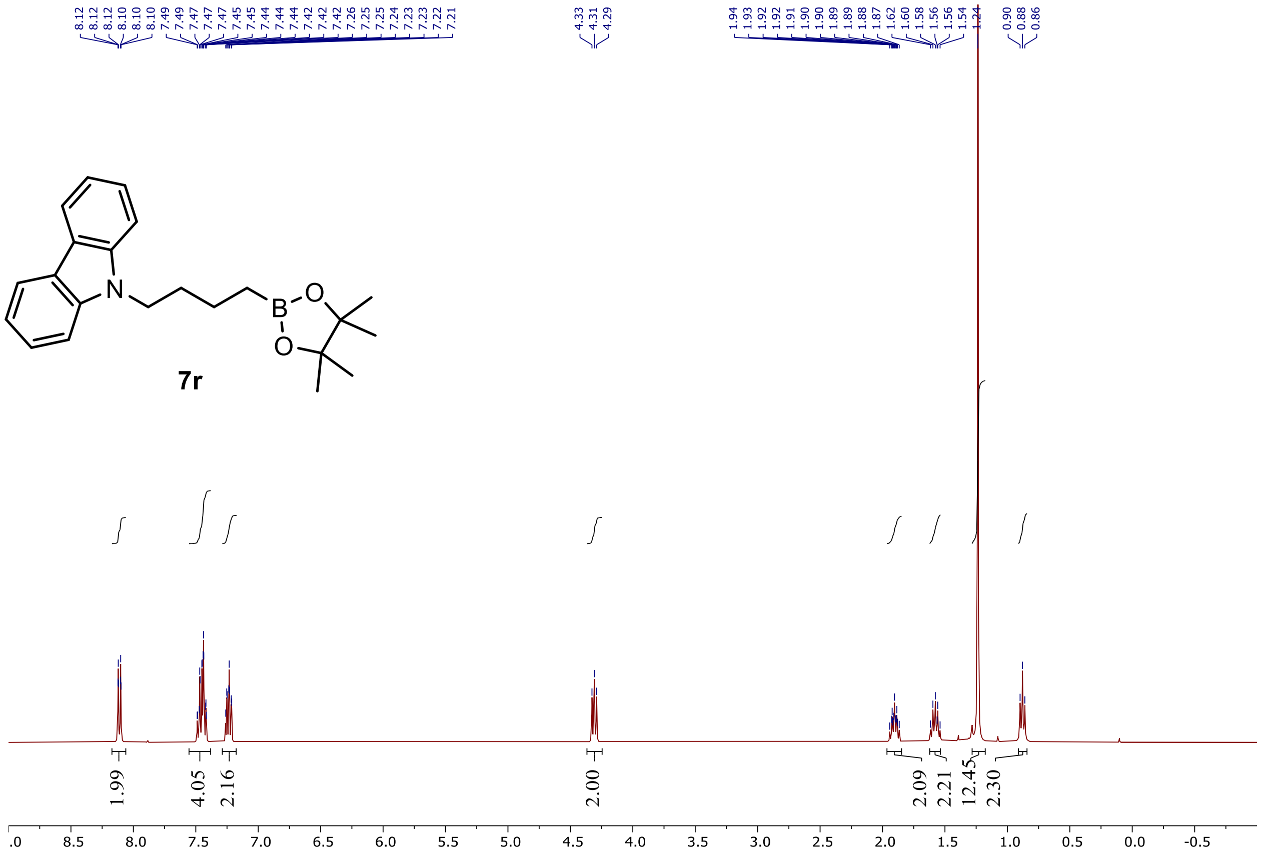

^{13}C NMR (101 MHz, $CDCl_3$) of **7r**

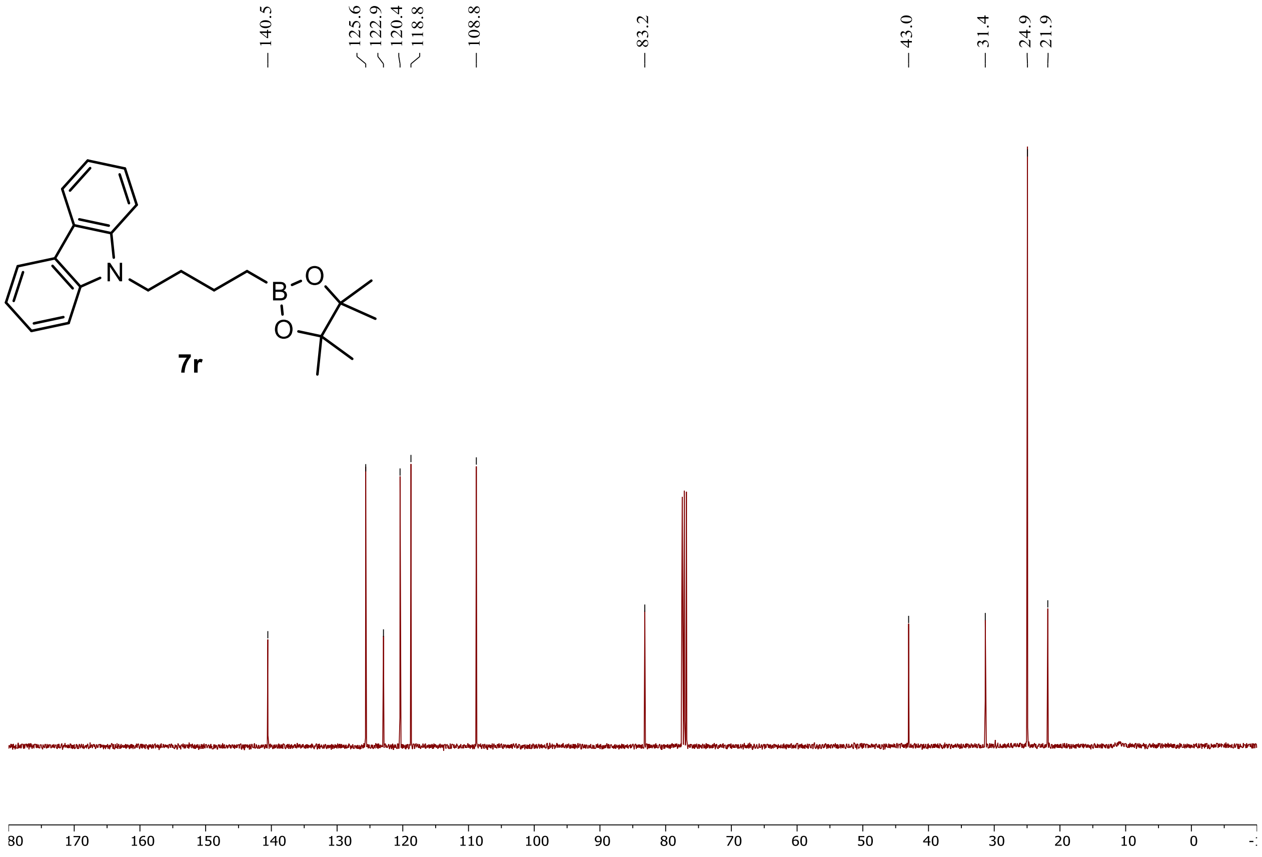

^{11}B NMR (128 MHz, $CDCl_3$) of **7r**

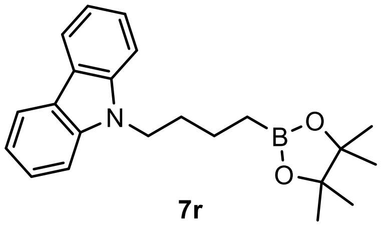

7r

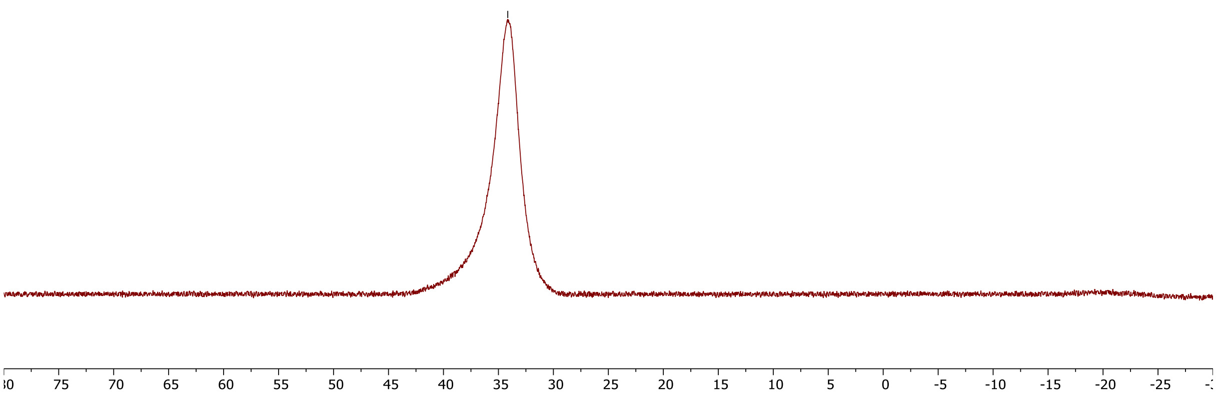

^{1}H NMR (500 MHz, $CDCl_3$) of **7s** (*see procedure*)

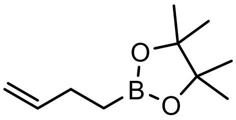

7s

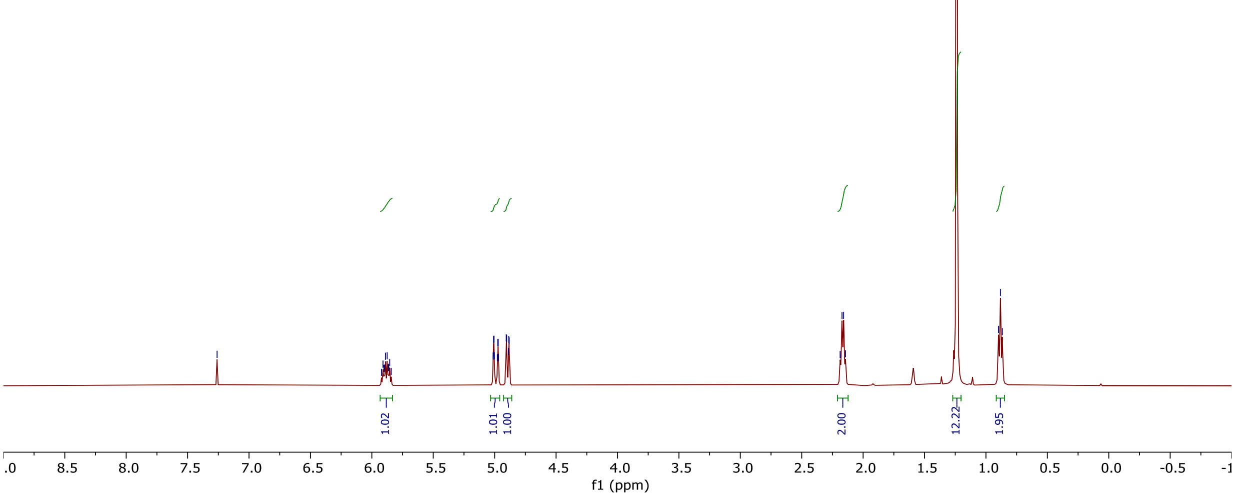

^{13}C NMR (126 MHz, $CDCl_3$) of **7s**

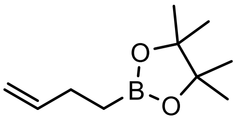

7s

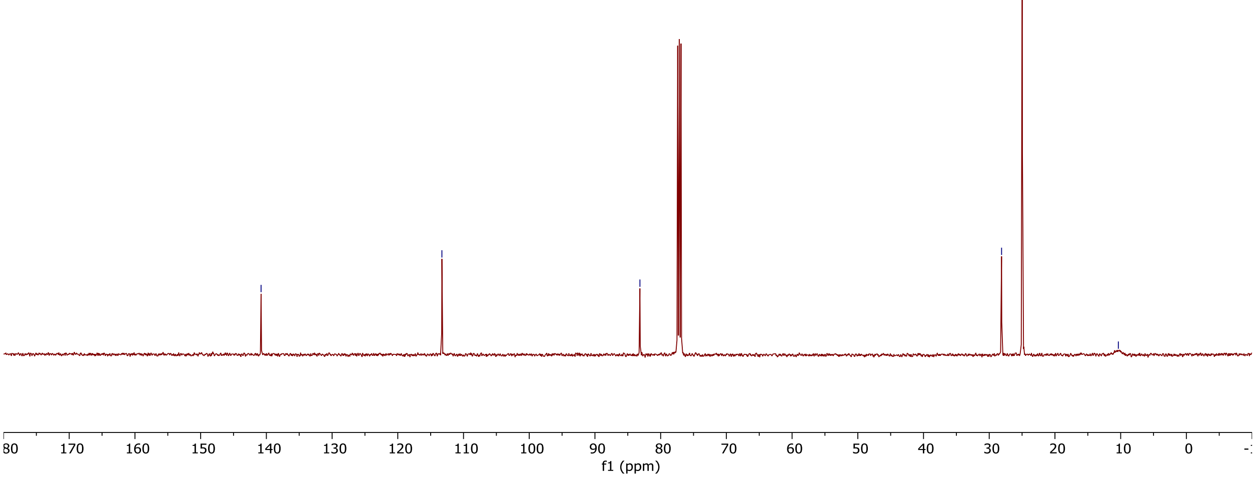

^{1}H NMR (400 MHz, $CDCl_3$) of **7t** (*see procedure*)

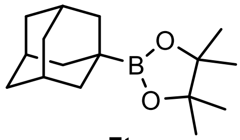

7t

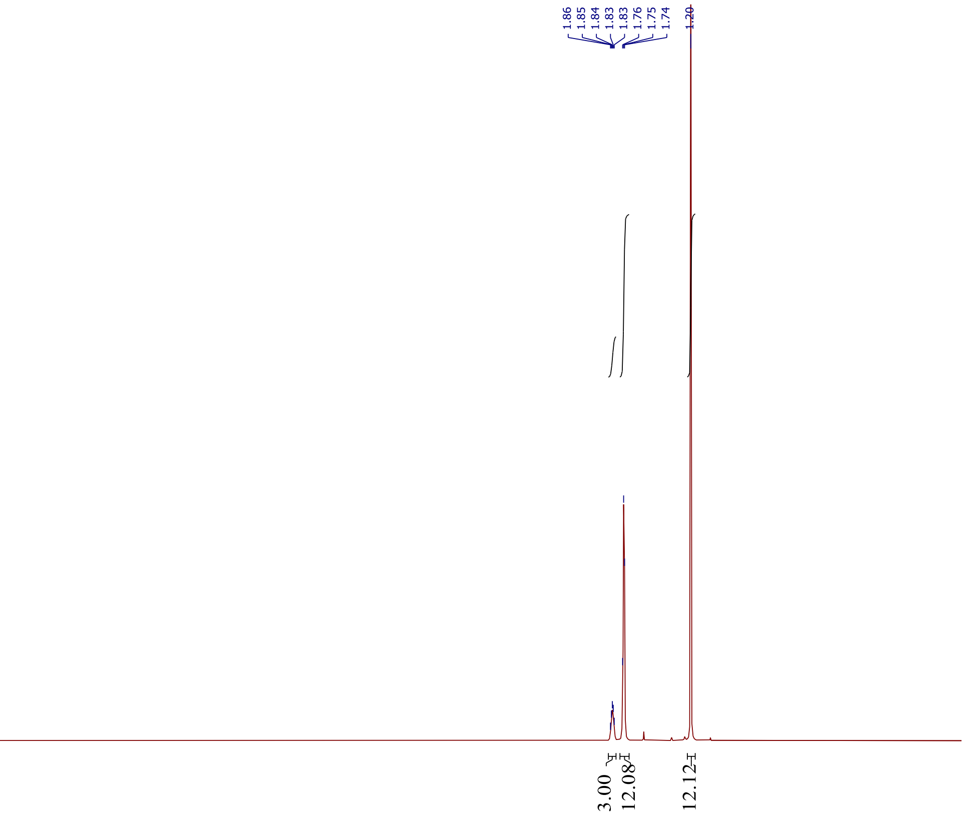

^{13}C NMR (101 MHz, $CDCl_3$) of **7t**

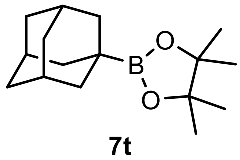

7t

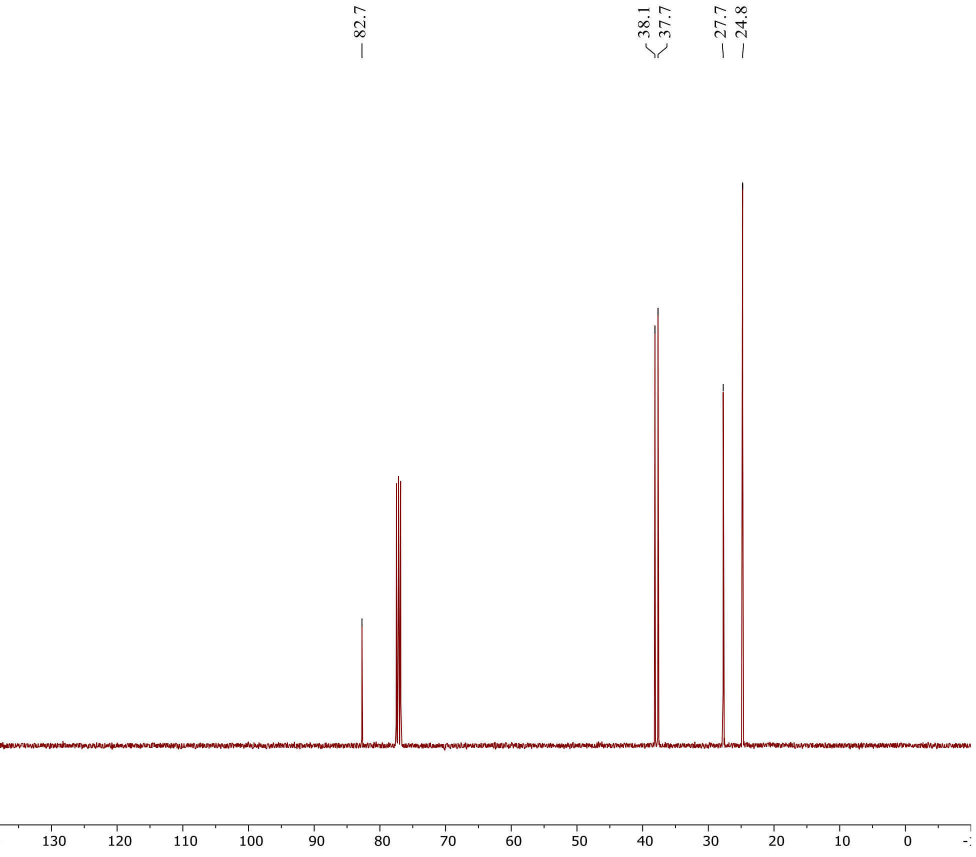

^{11}B NMR (128 MHz, $CDCl_3$) of **7t**

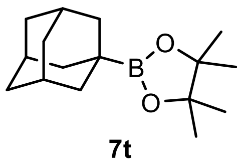

7t

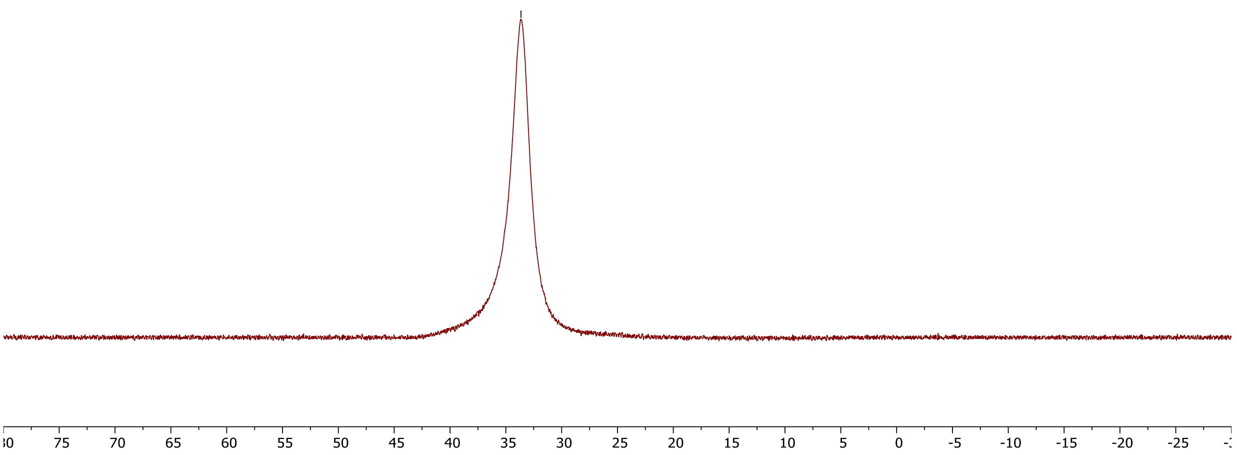

^{1}H NMR (400 MHz, $CDCl_3$) of **7u** (see procedure)

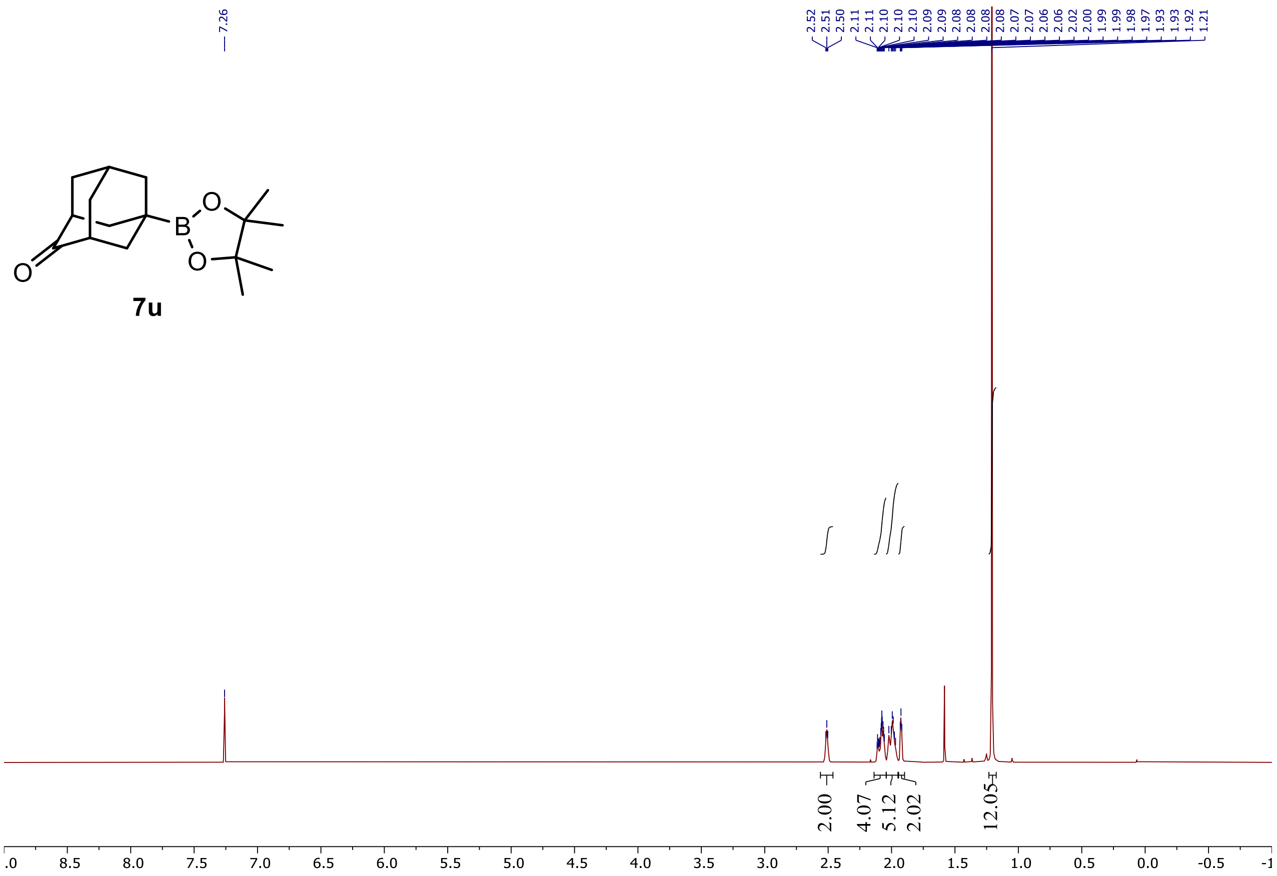

^{13}C NMR (101 MHz, $CDCl_3$) of **7u**

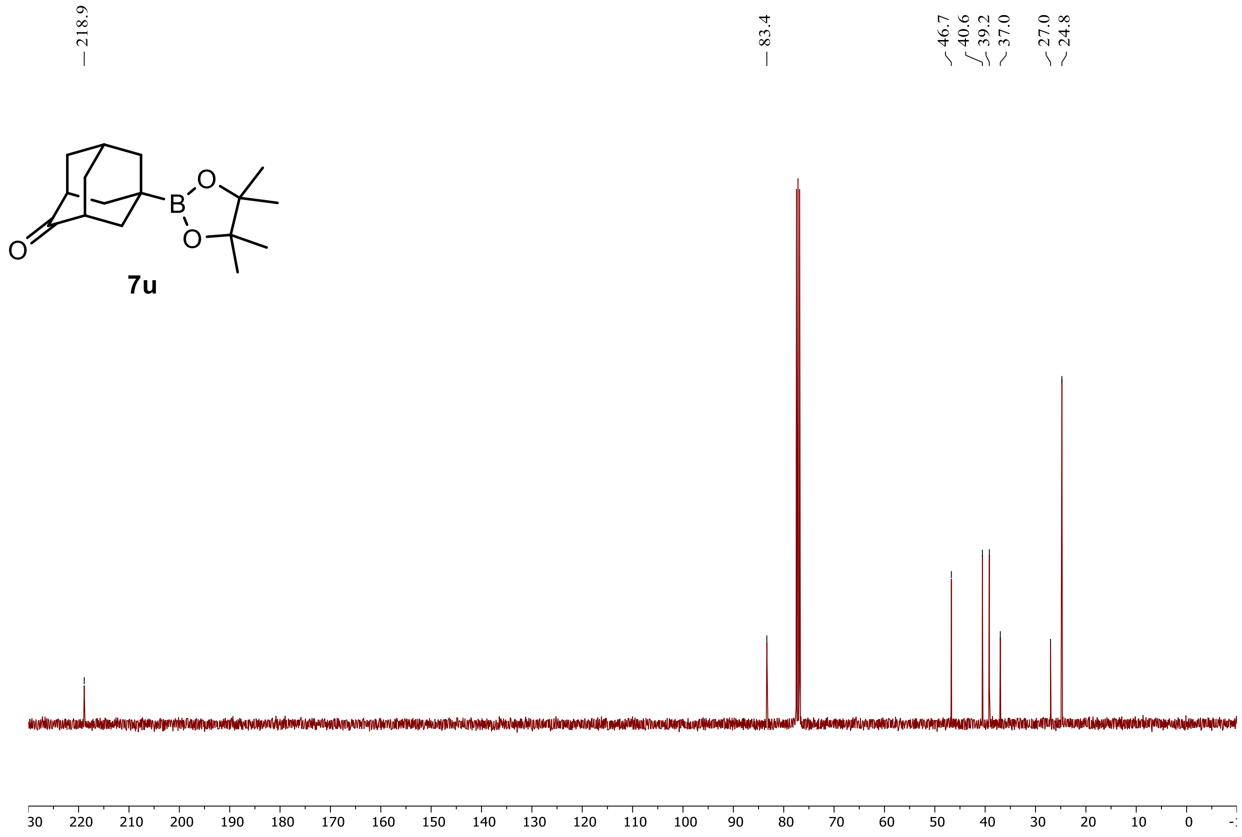

^{11}B NMR (128 MHz, $CDCl_3$) of **7u**

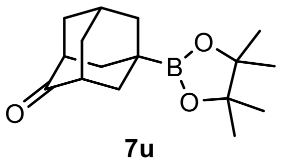

7u

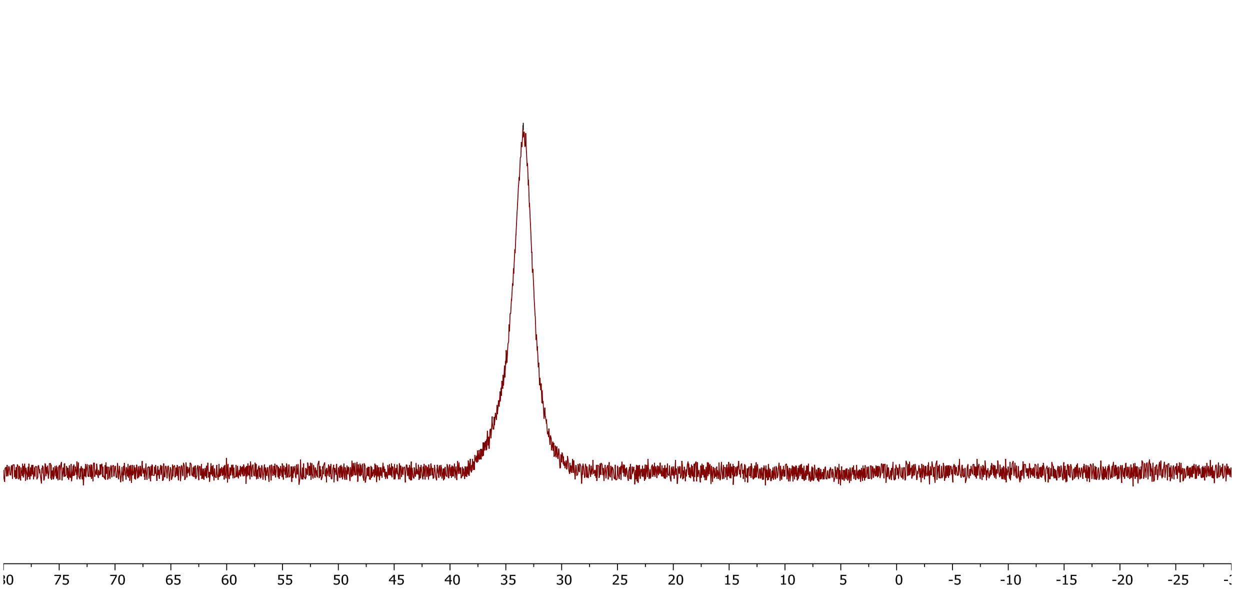

^{1}H NMR (400 MHz, $CDCl_3$) of **7v** (see procedure)

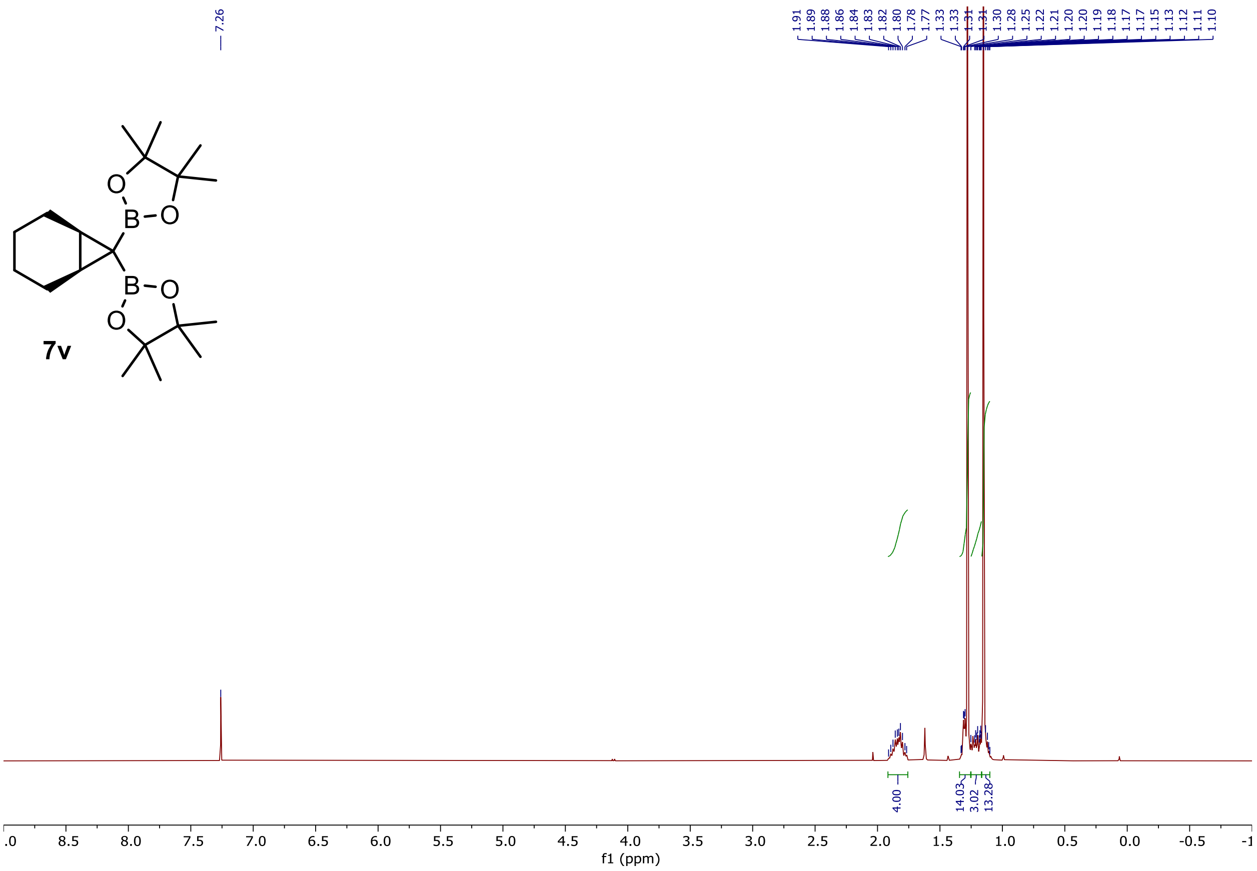

^{13}C NMR (101 MHz, $CDCl_3$) of **7v**

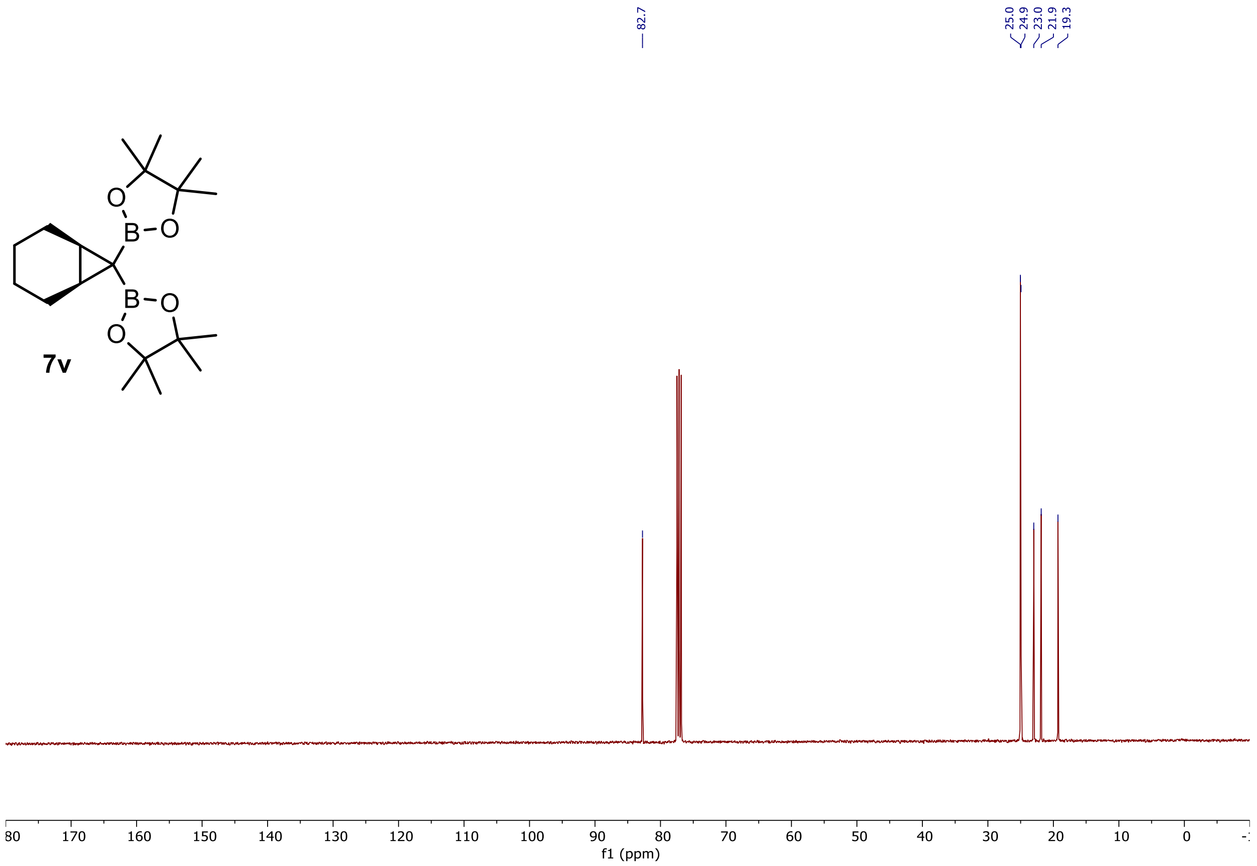

HSQC of **7v**

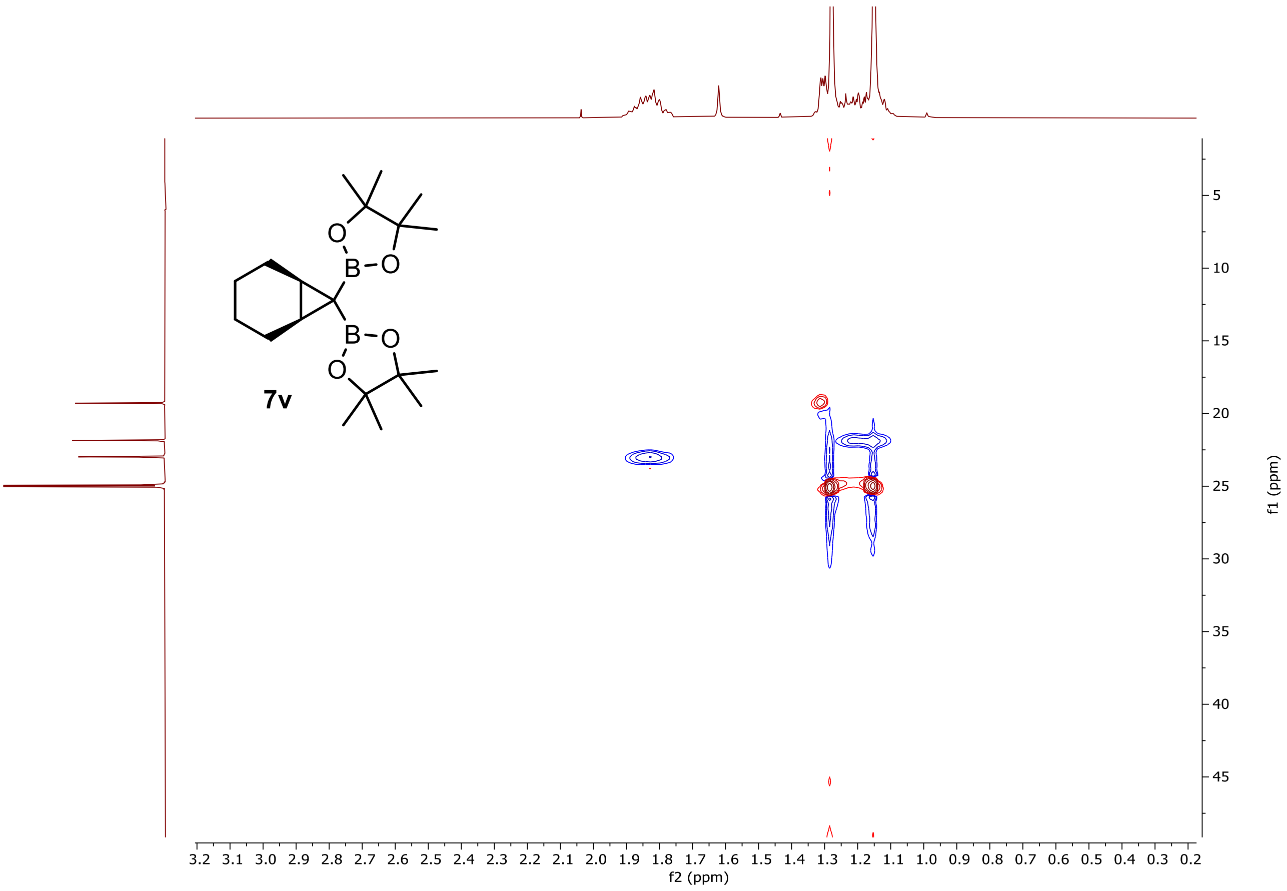

^{1}H NMR (400 MHz, $CDCl_3$) of **7w** (*see procedure*)

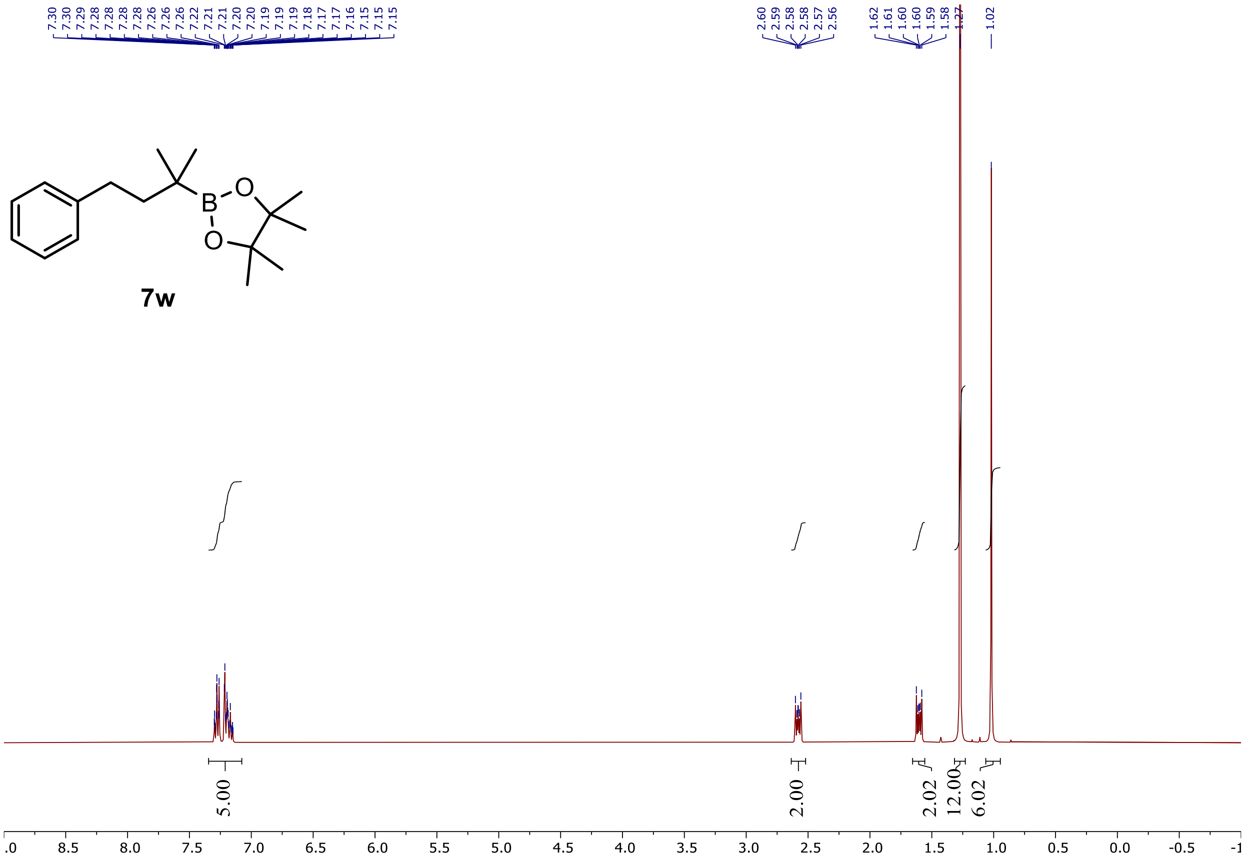

7w

^{13}C NMR (101 MHz, $CDCl_3$) of **7w**

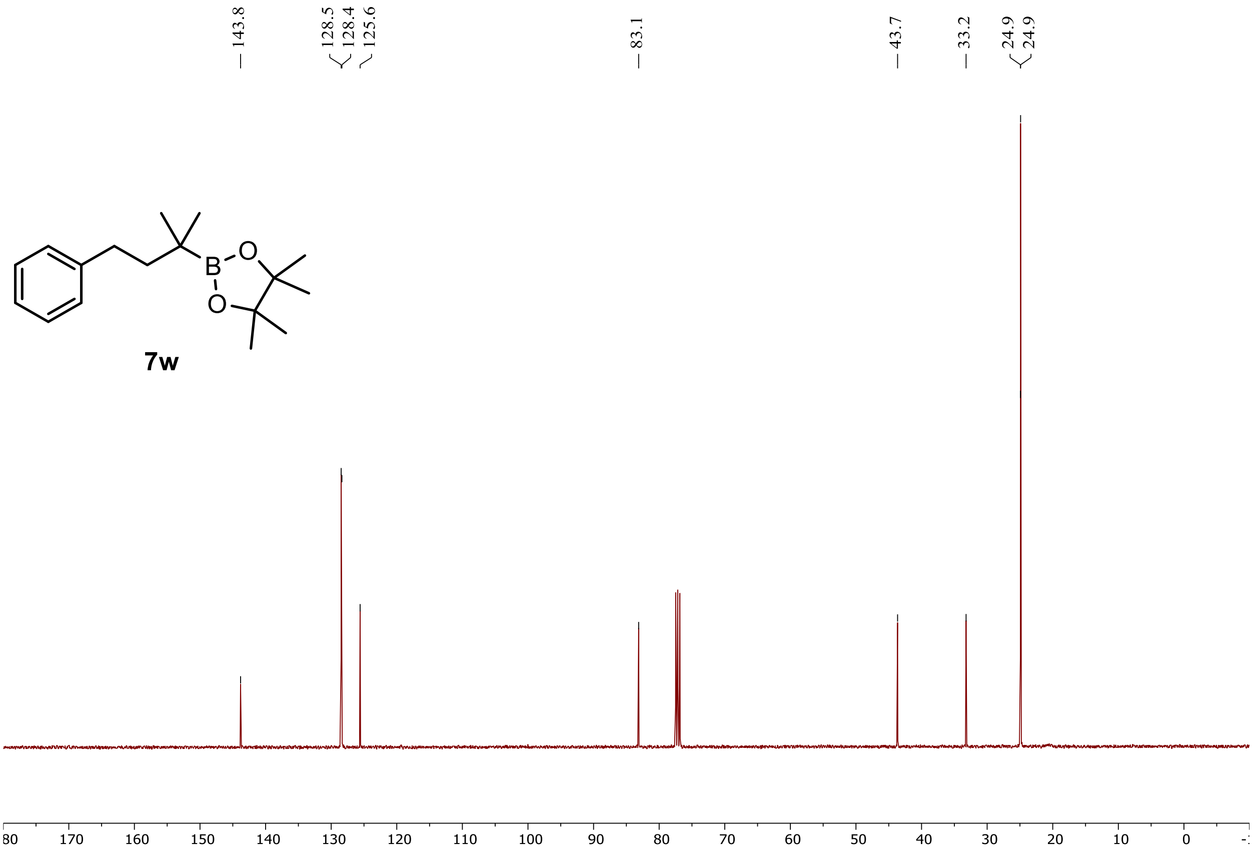

7w

^{11}B NMR (128 MHz, $CDCl_3$) of **7w**

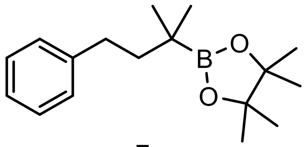

7w

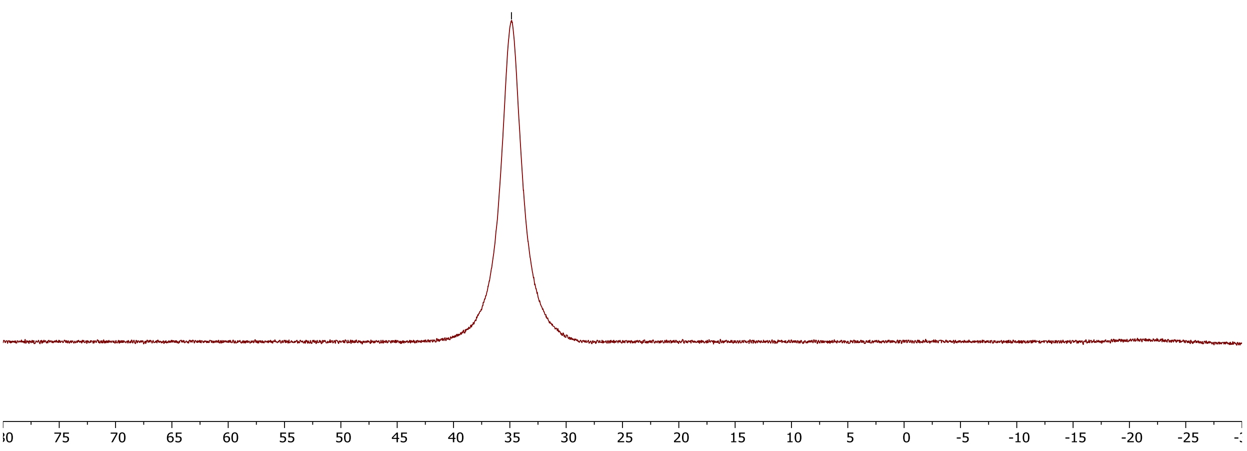

^{1}H NMR (500 MHz, $CDCl_3$) of **7x** (*see procedure*)

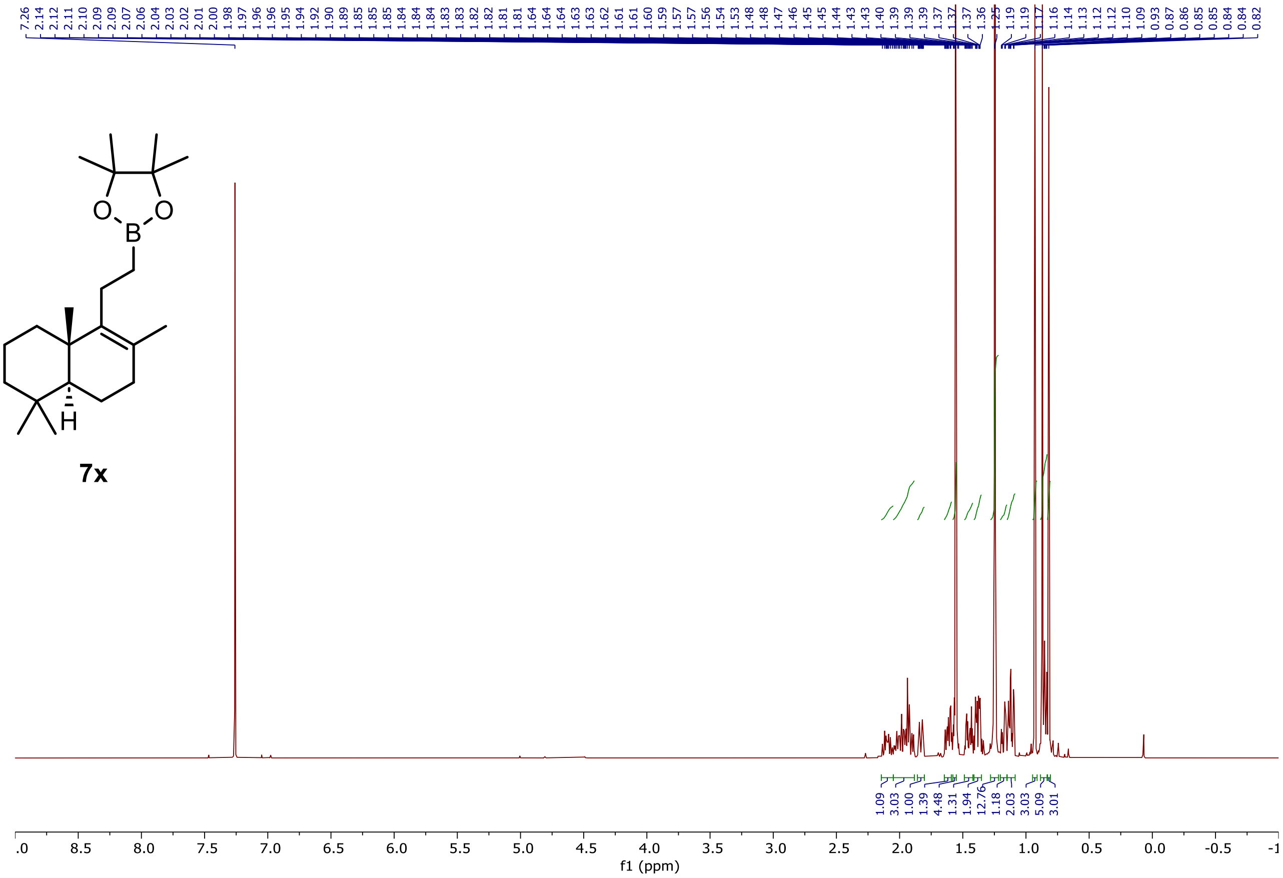

^{13}C NMR (126 MHz, $CDCl_3$) of **7x**

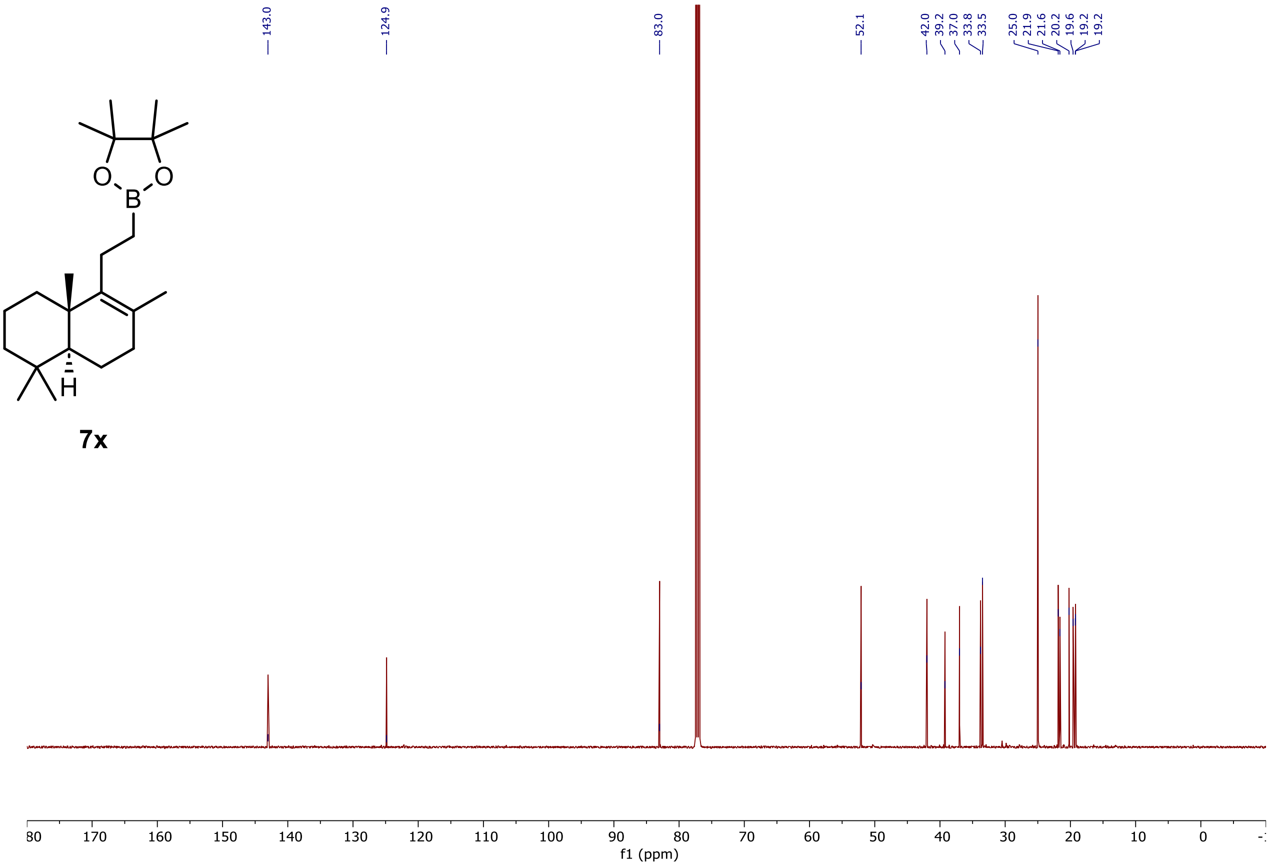

HSQC of **7x**

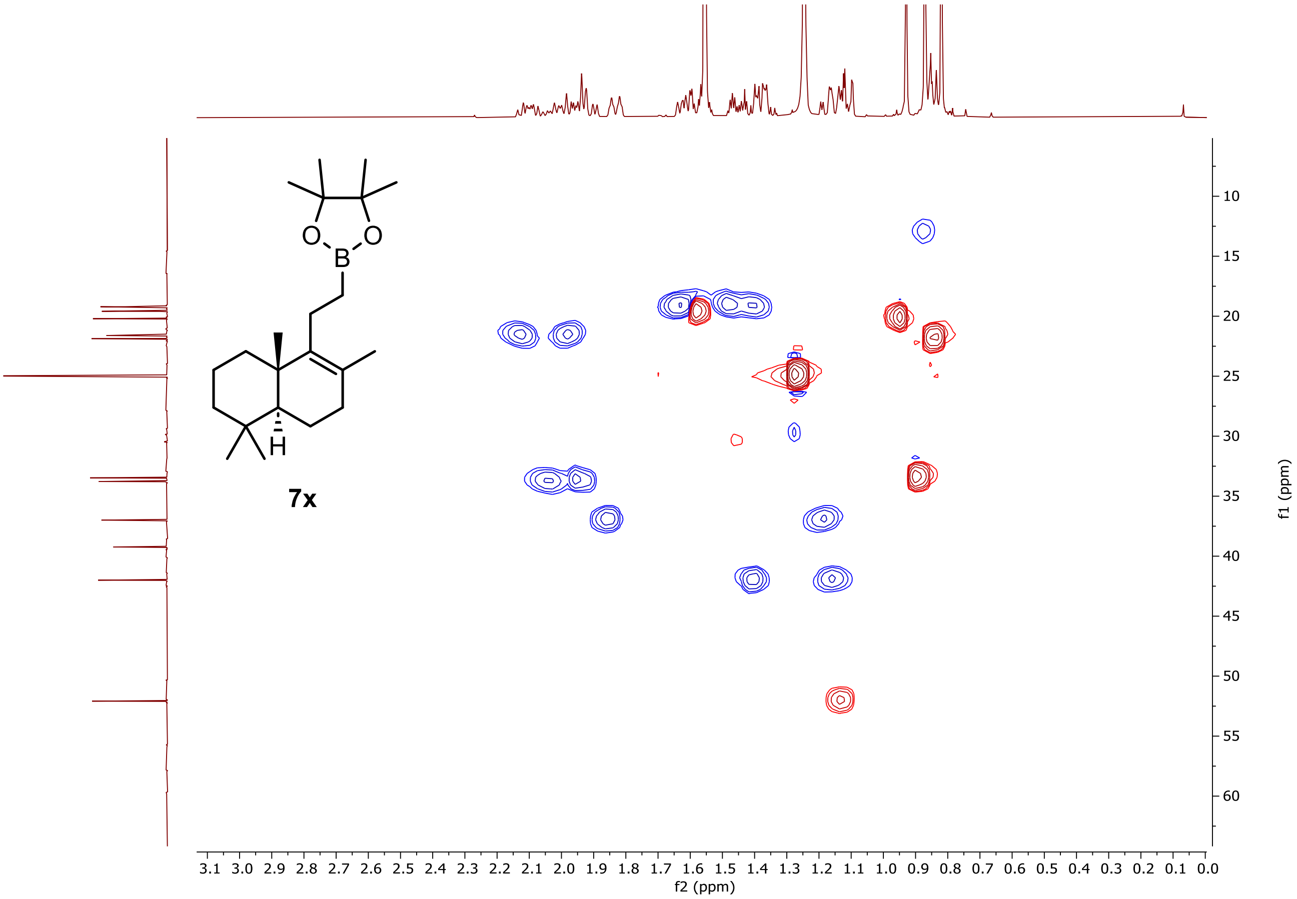

^{1}H NMR (400 MHz, $CDCl_3$) of **7y** (see procedure)

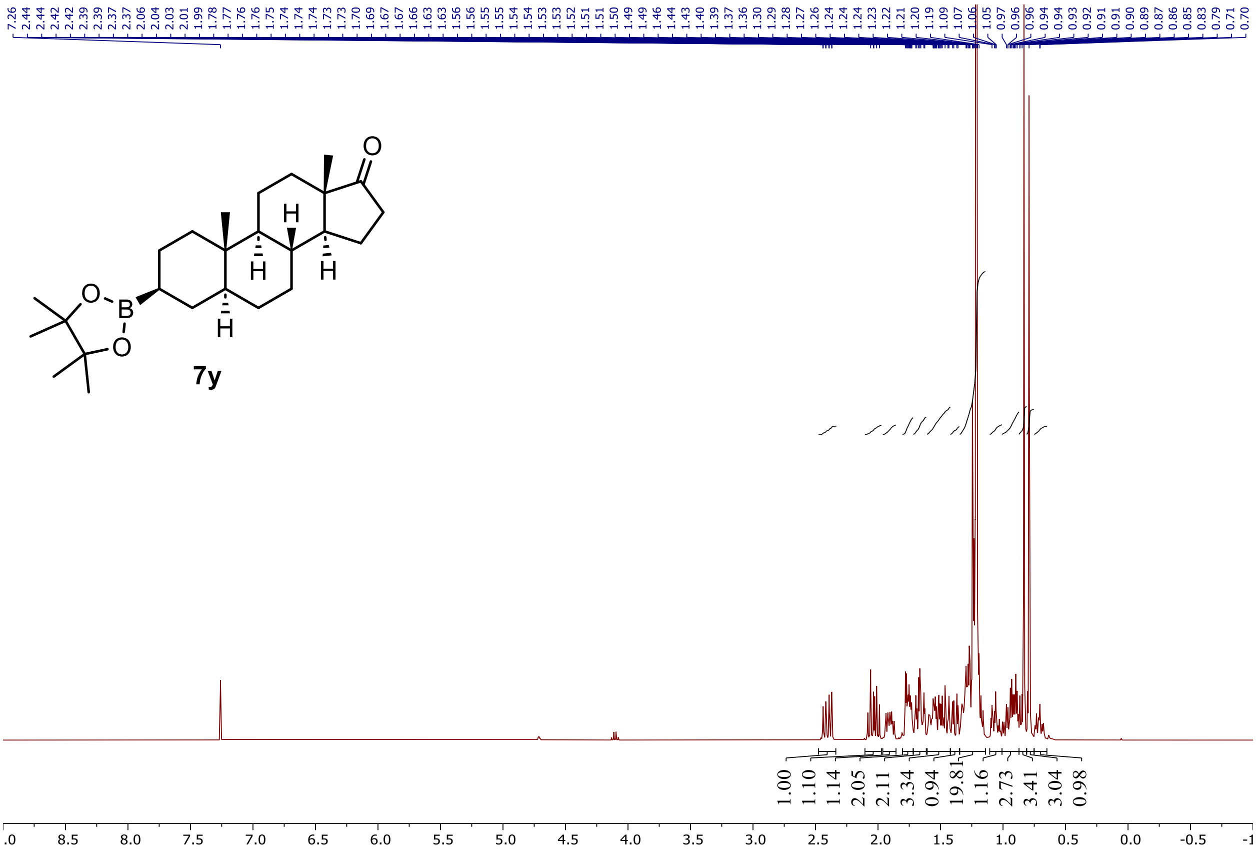

^{13}C NMR (101 MHz, $CDCl_3$) of **7y**

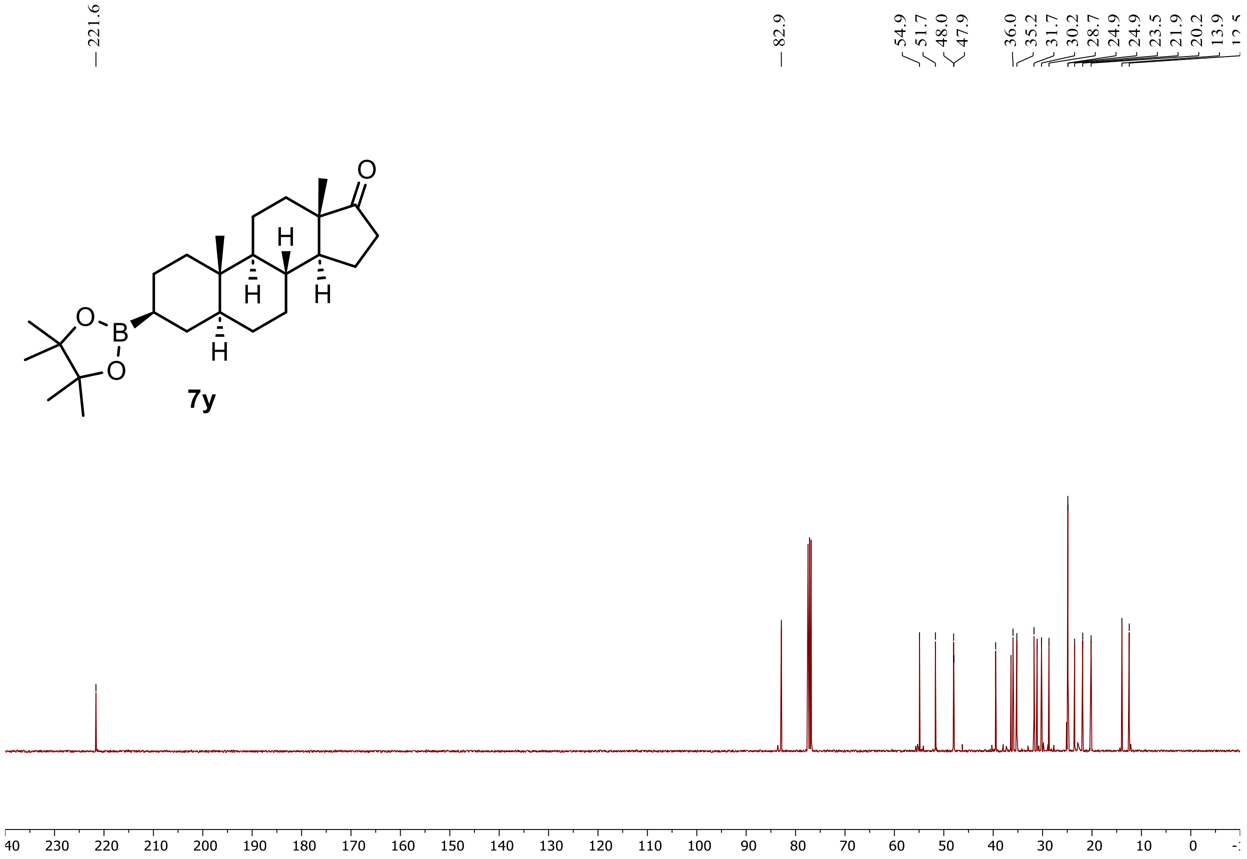

^{11}B NMR (128 MHz, $CDCl_3$) of **7y**

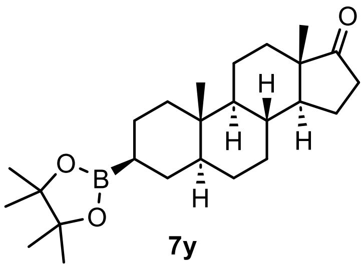

7y

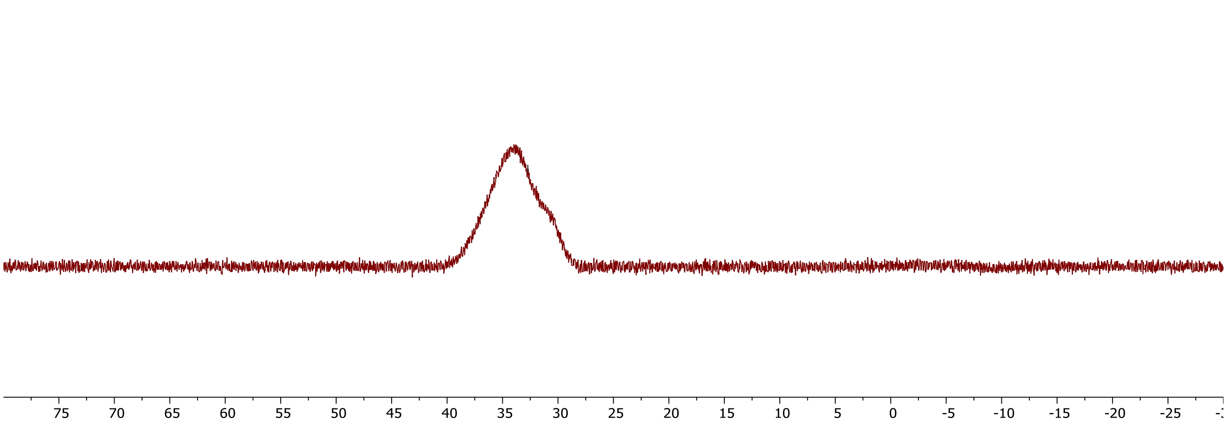

^{1}H NMR (400 MHz, $CDCl_3$) of **7z** (<u>*see procedure*</u>)

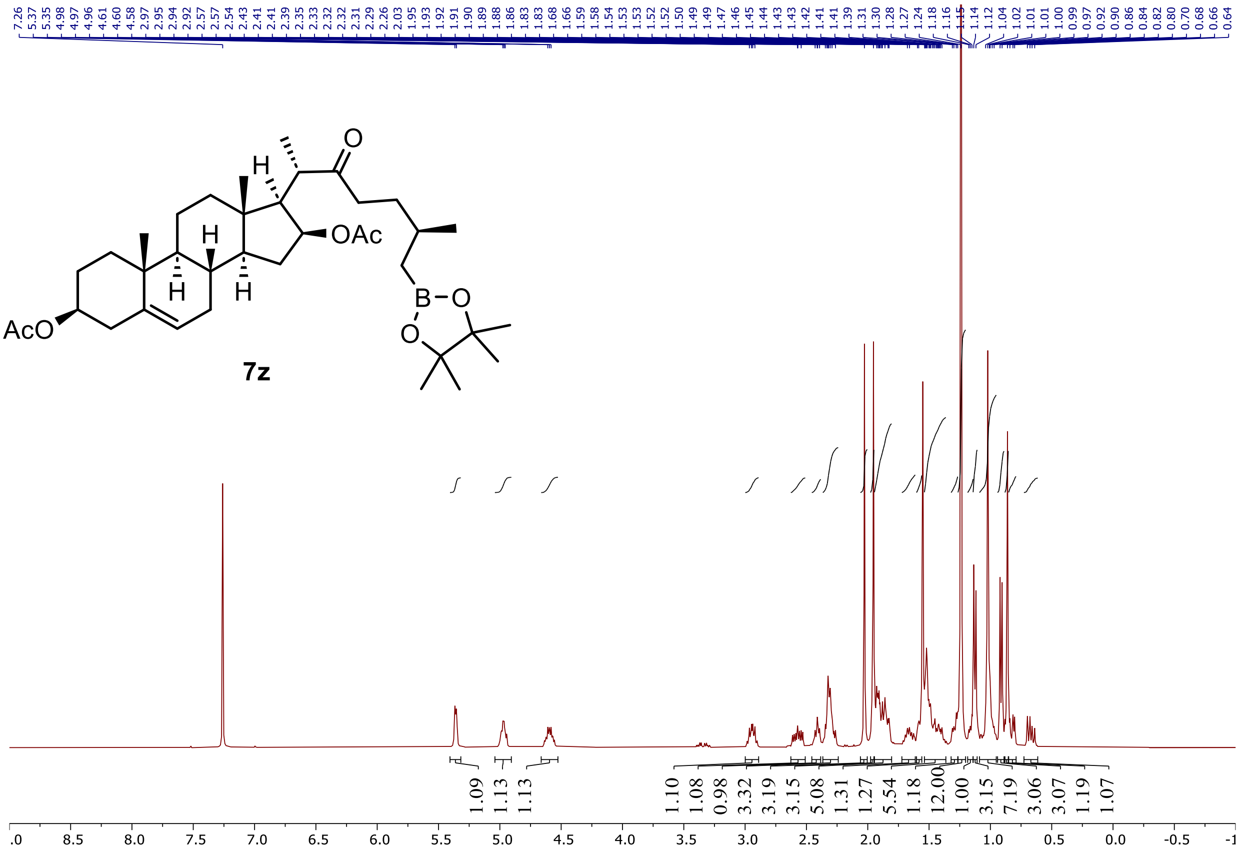

^{13}C NMR (101 MHz, $CDCl_3$) of **7z**

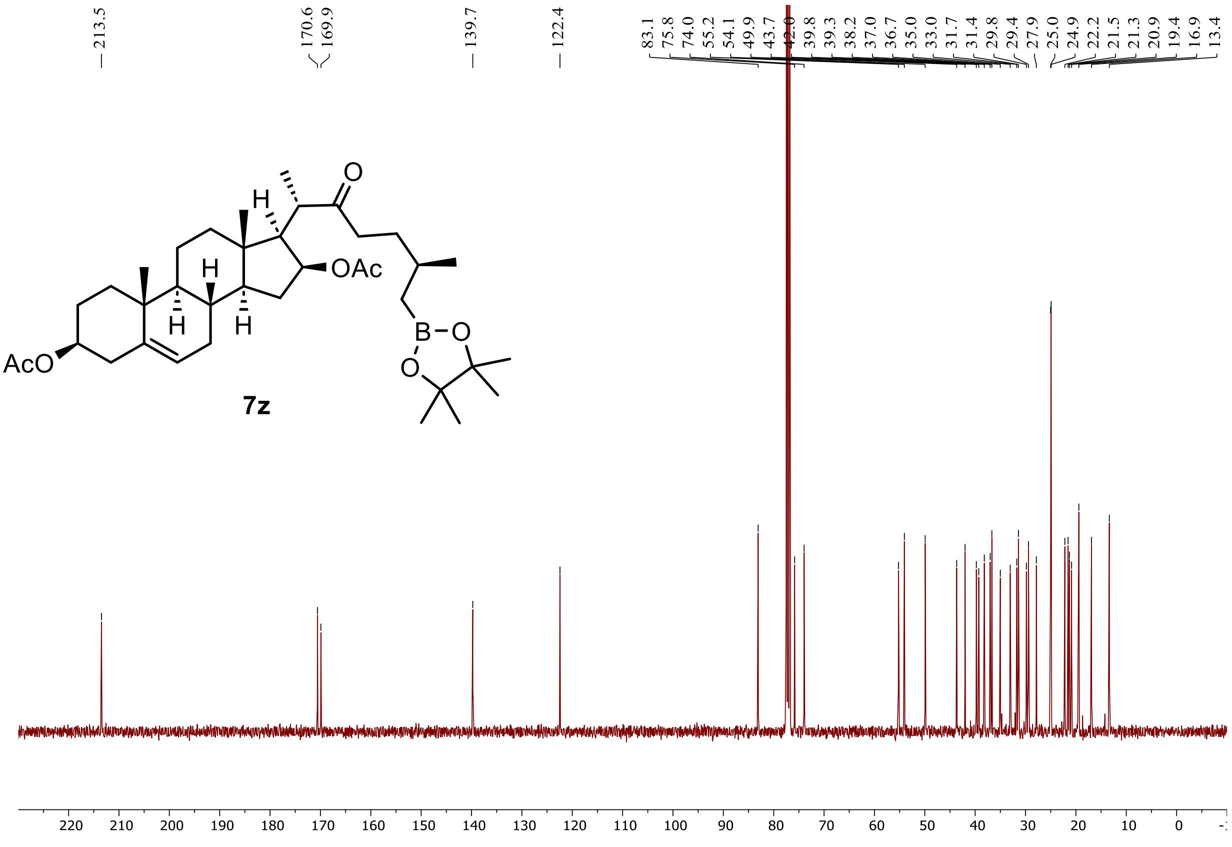

^{11}B NMR (128 MHz, $CDCl_3$) of **7z**

34.66

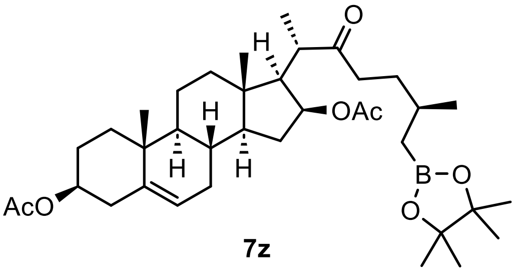

7z

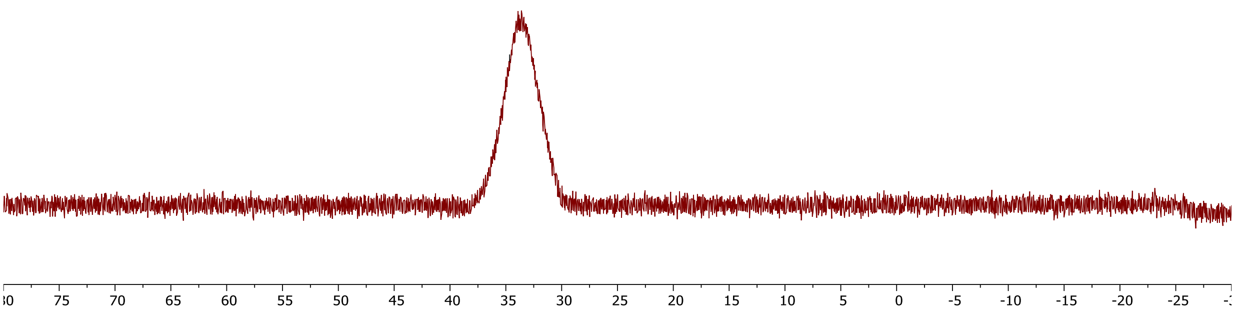

^{1}H NMR (500 MHz, $CDCl_3$) of **7aa** (*see procedure*)

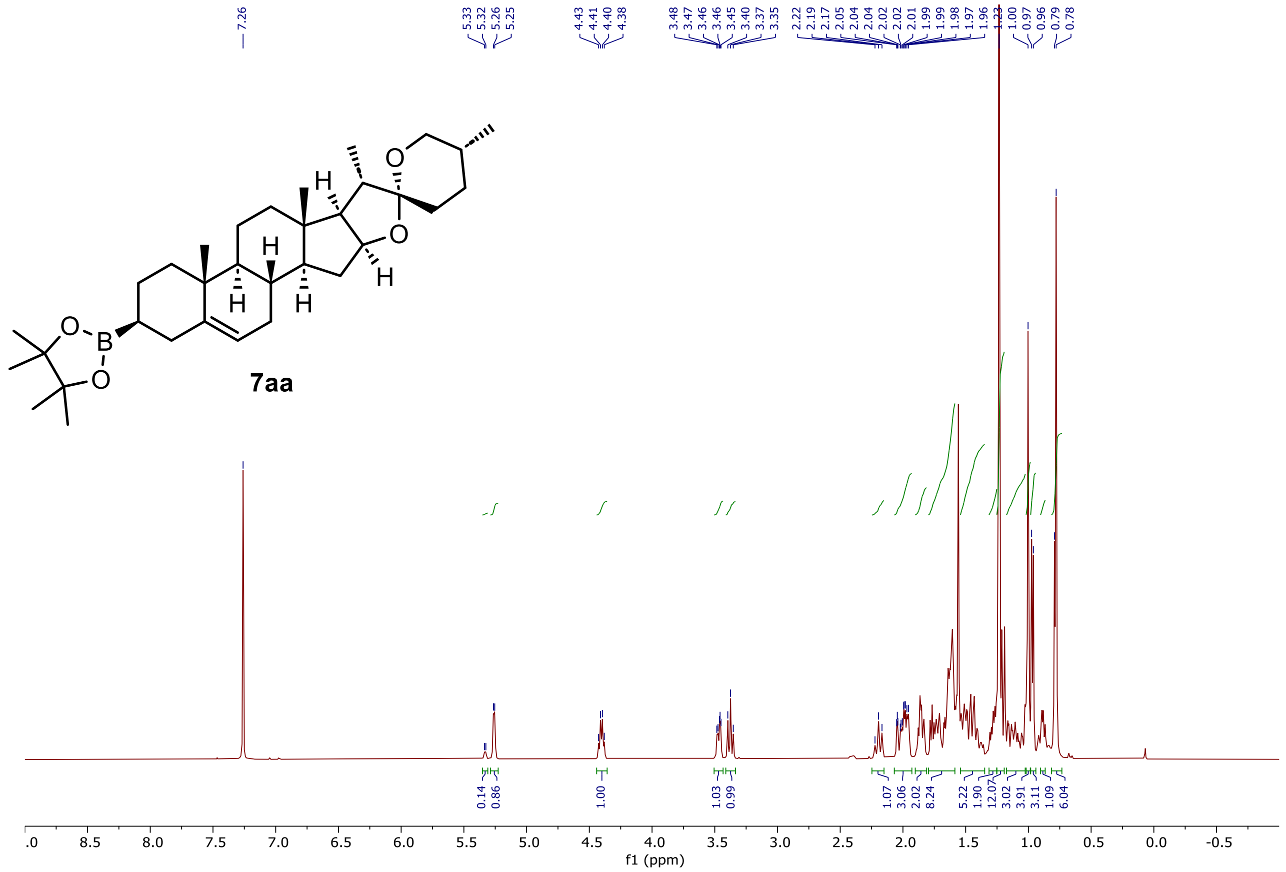

^{13}C NMR (126 MHz, $CDCl_3$) of **7aa**

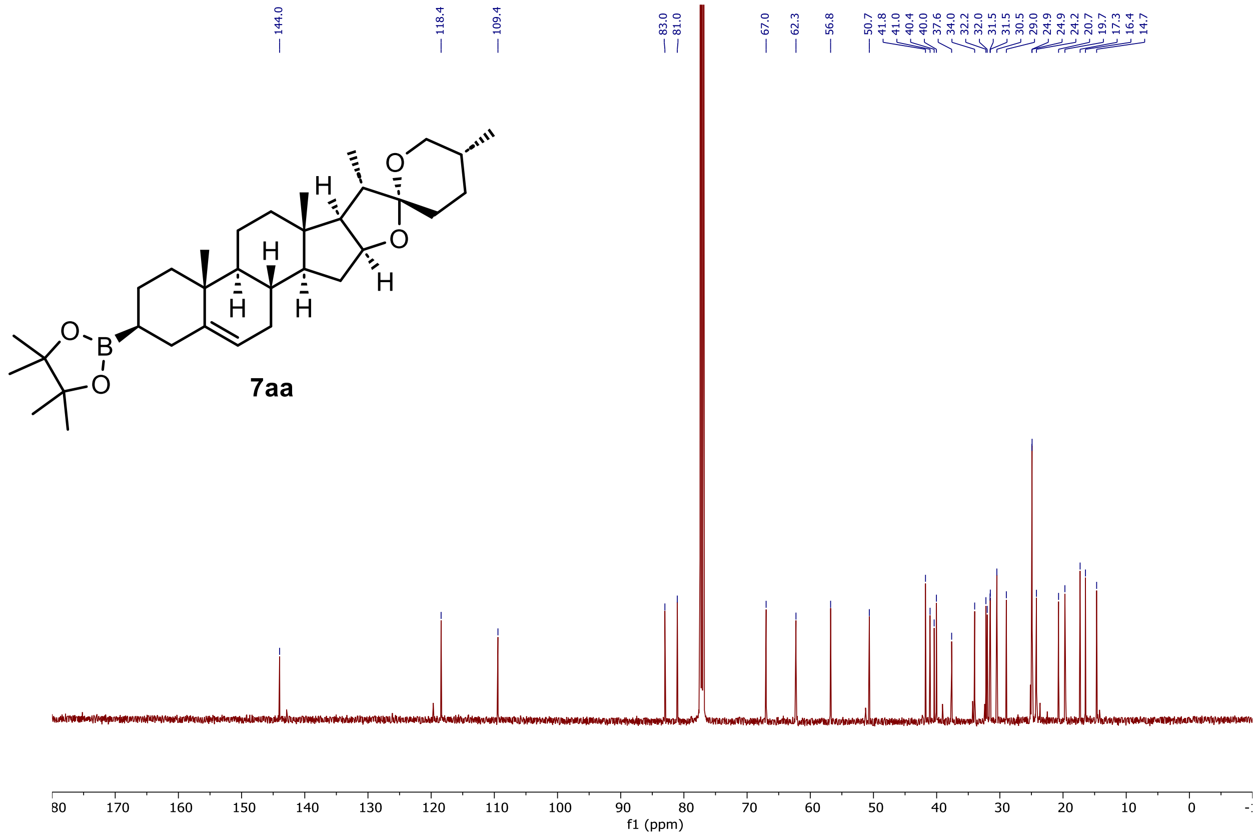

HSQC of **7aa**

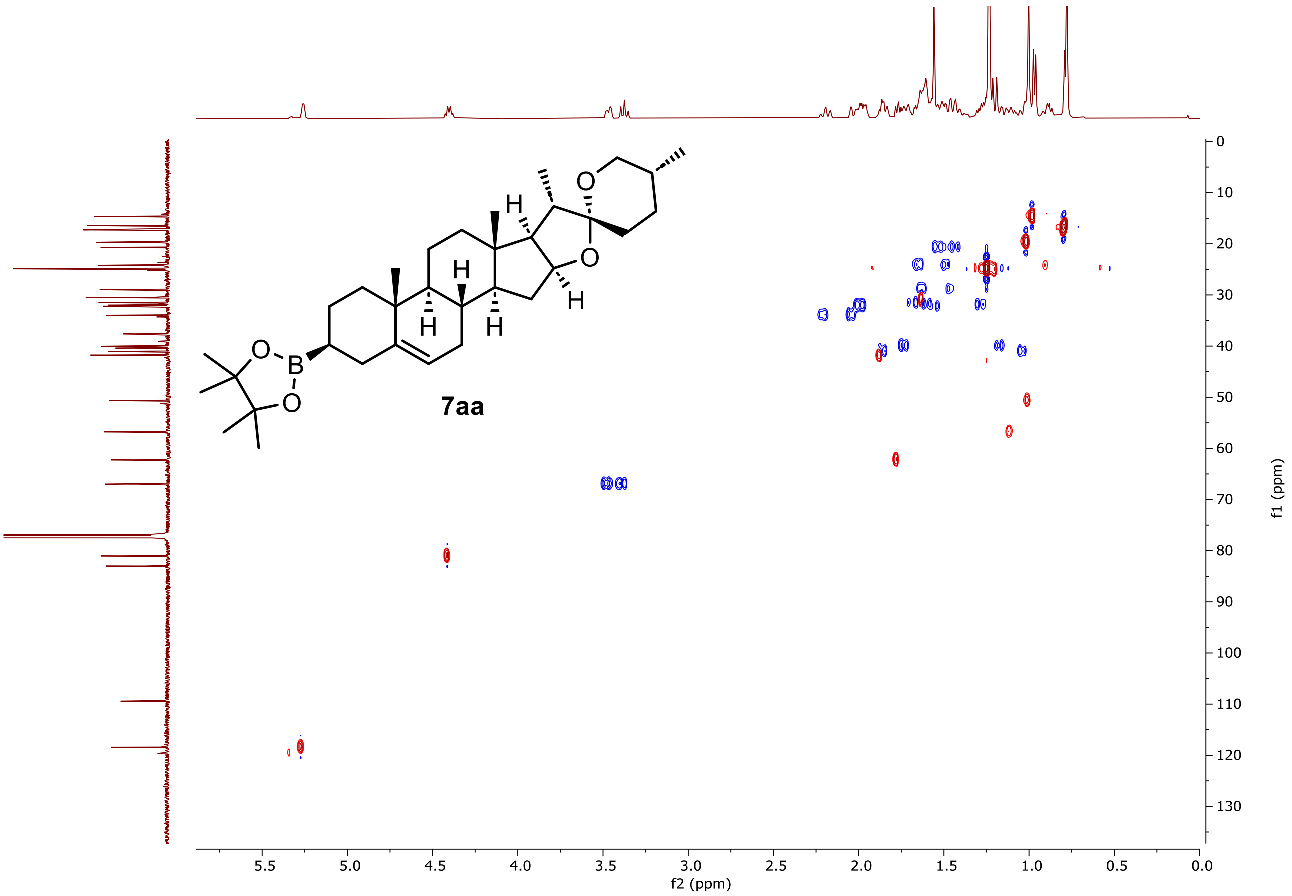

^{1}H NMR (500 MHz, $CDCl_3$) of **7ab** (see procedure)

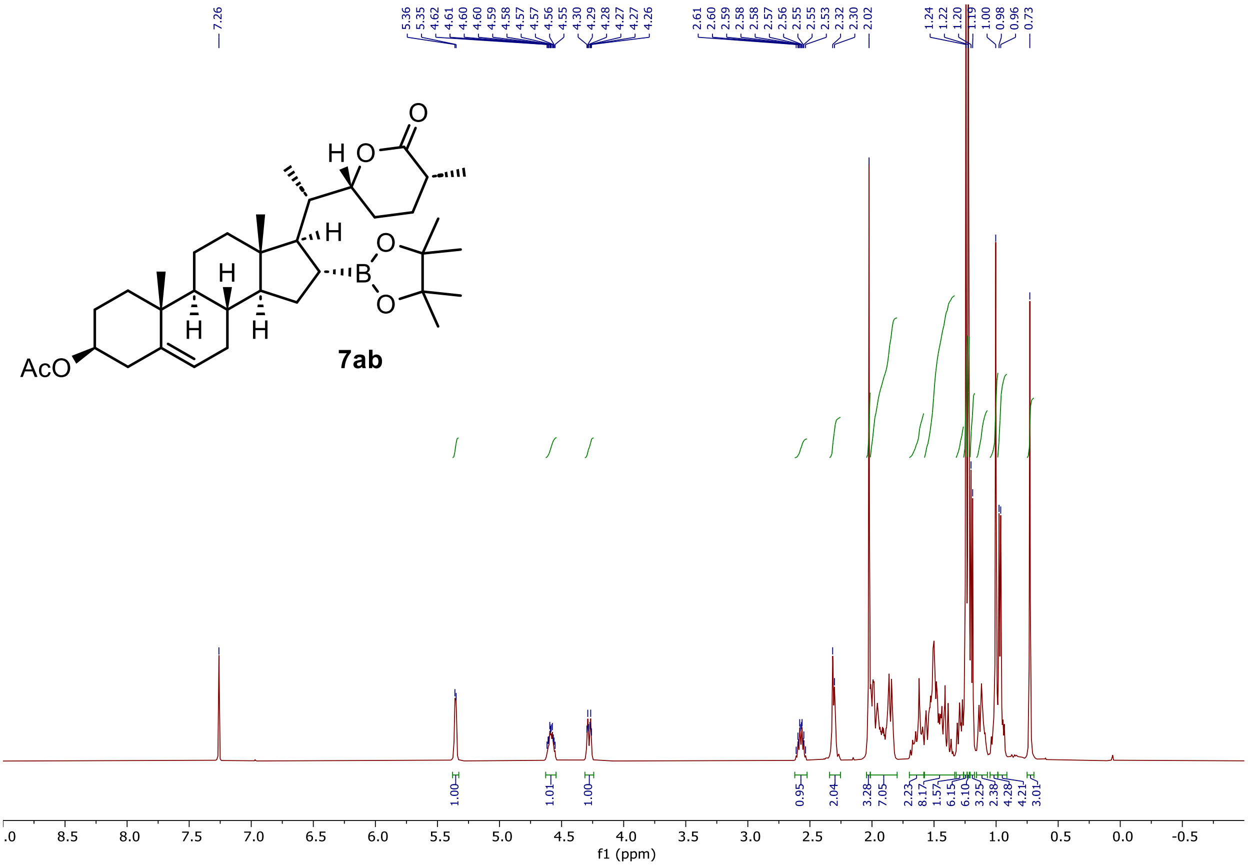

^{13}C NMR (126 MHz, $CDCl_3$) of **7ab**

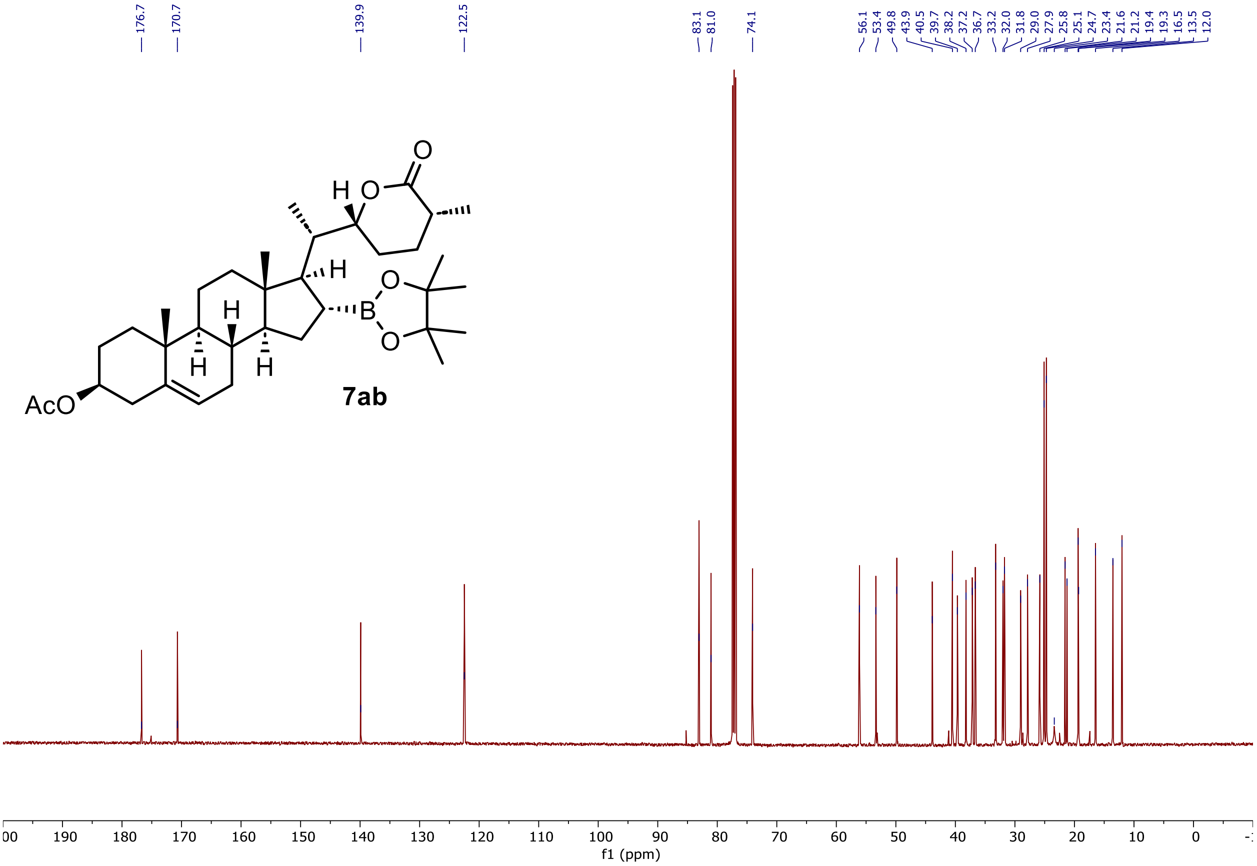

HSQC of **7ab**

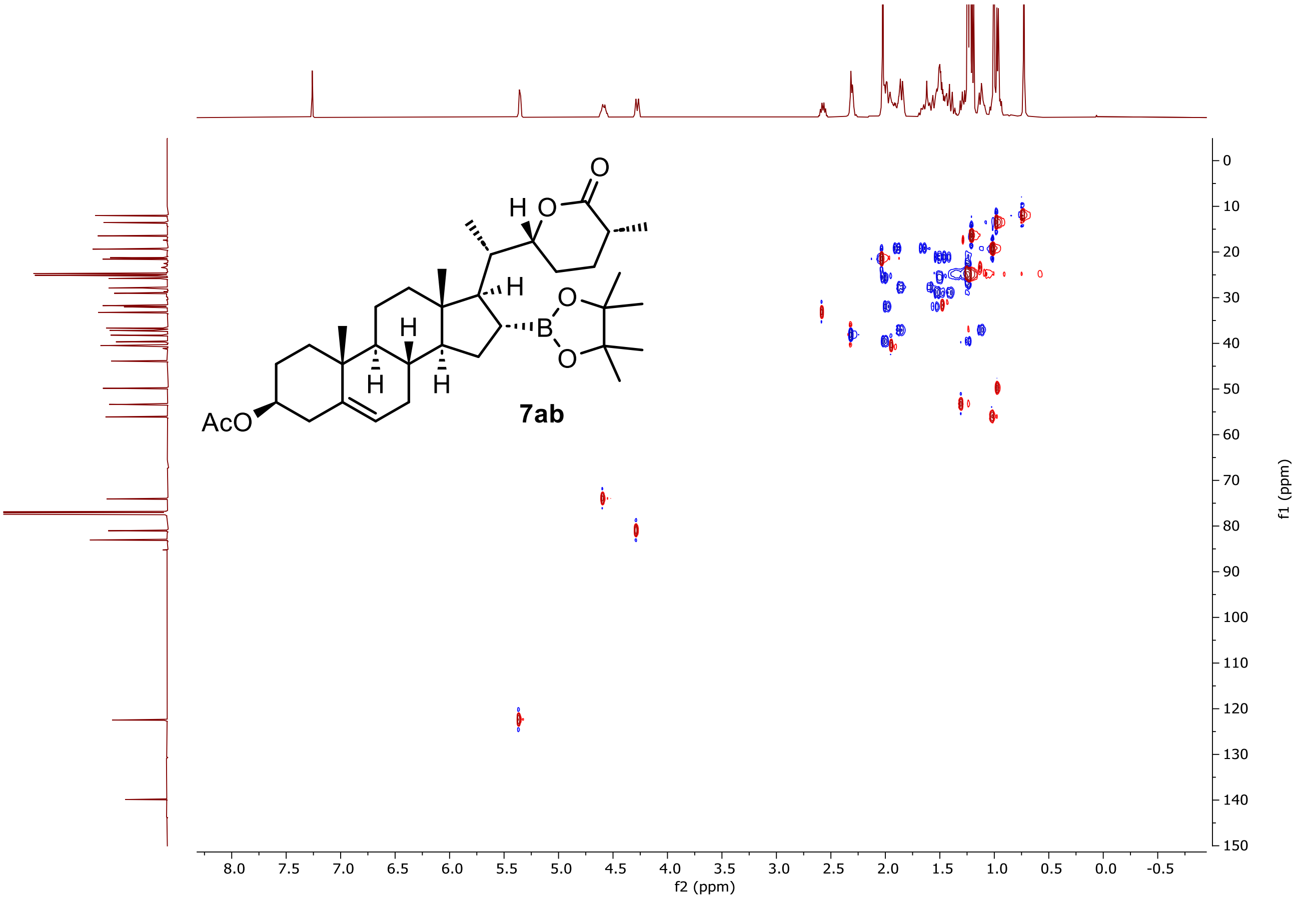
AcO
H
H
H
H
H O
O
O
B
O
7ab
8.0
7.5
7.0
6.5
6.0
5.5
5.0
4.5
4.0
3.5
3.0
2.5
2.0
1.5
1.0
0.5
0.0
-0.5
f2 (ppm)
0
10
20
30
40
50
60
70
80
90
100
110
120
130
140
150
f1 (ppm)

^{1}H NMR (500 MHz, $CDCl_3$) of **S1ab** (*see procedure*)

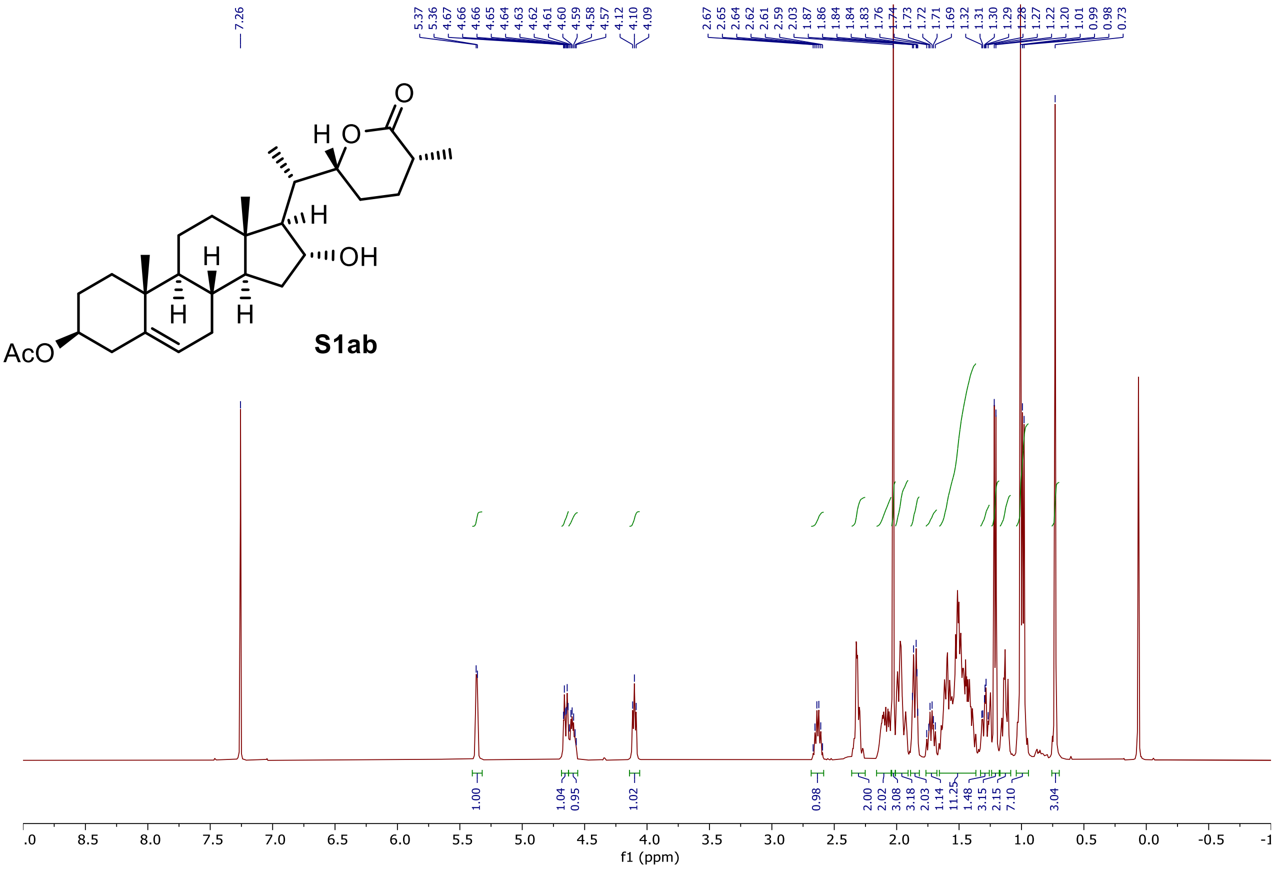

^{13}C NMR (126 MHz, $CDCl_3$) of **S1ab**

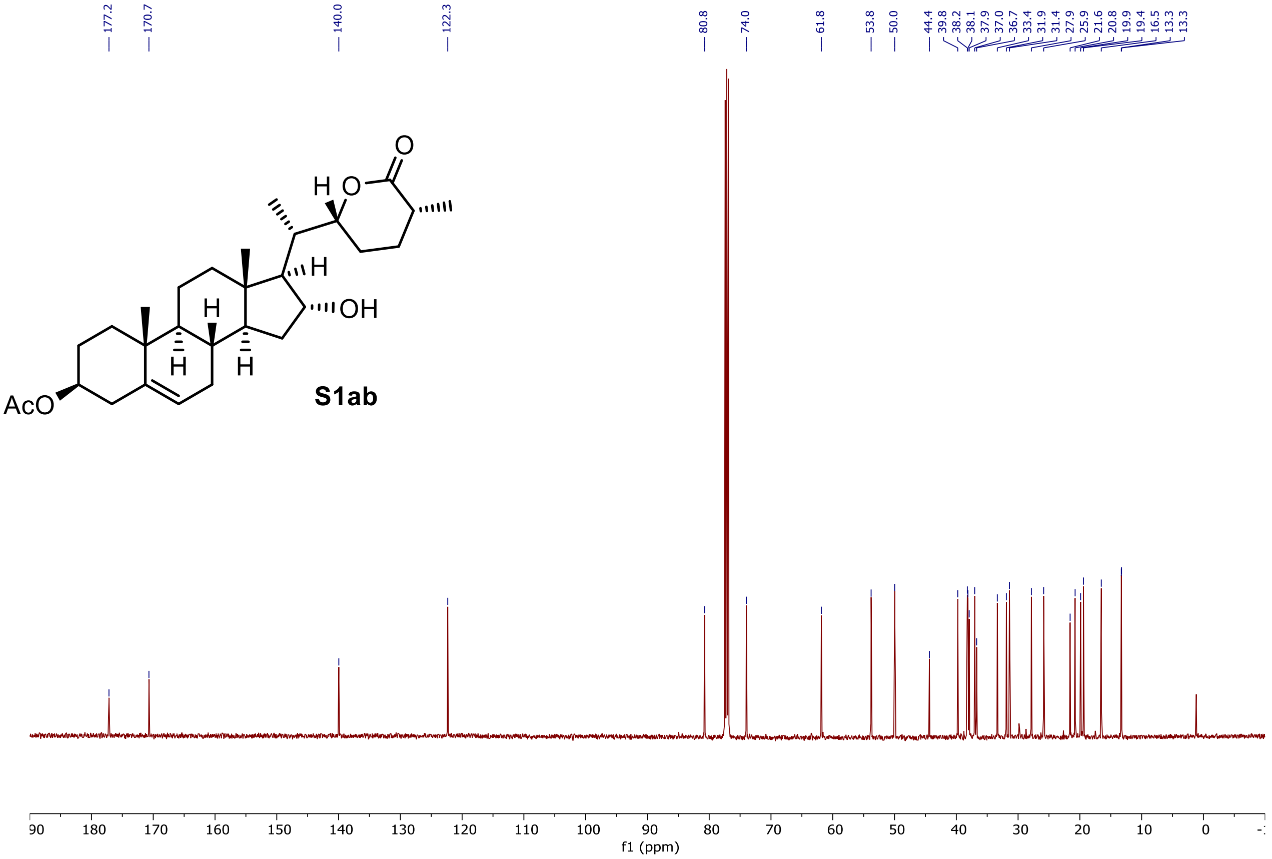

HSQC of **S1ab**

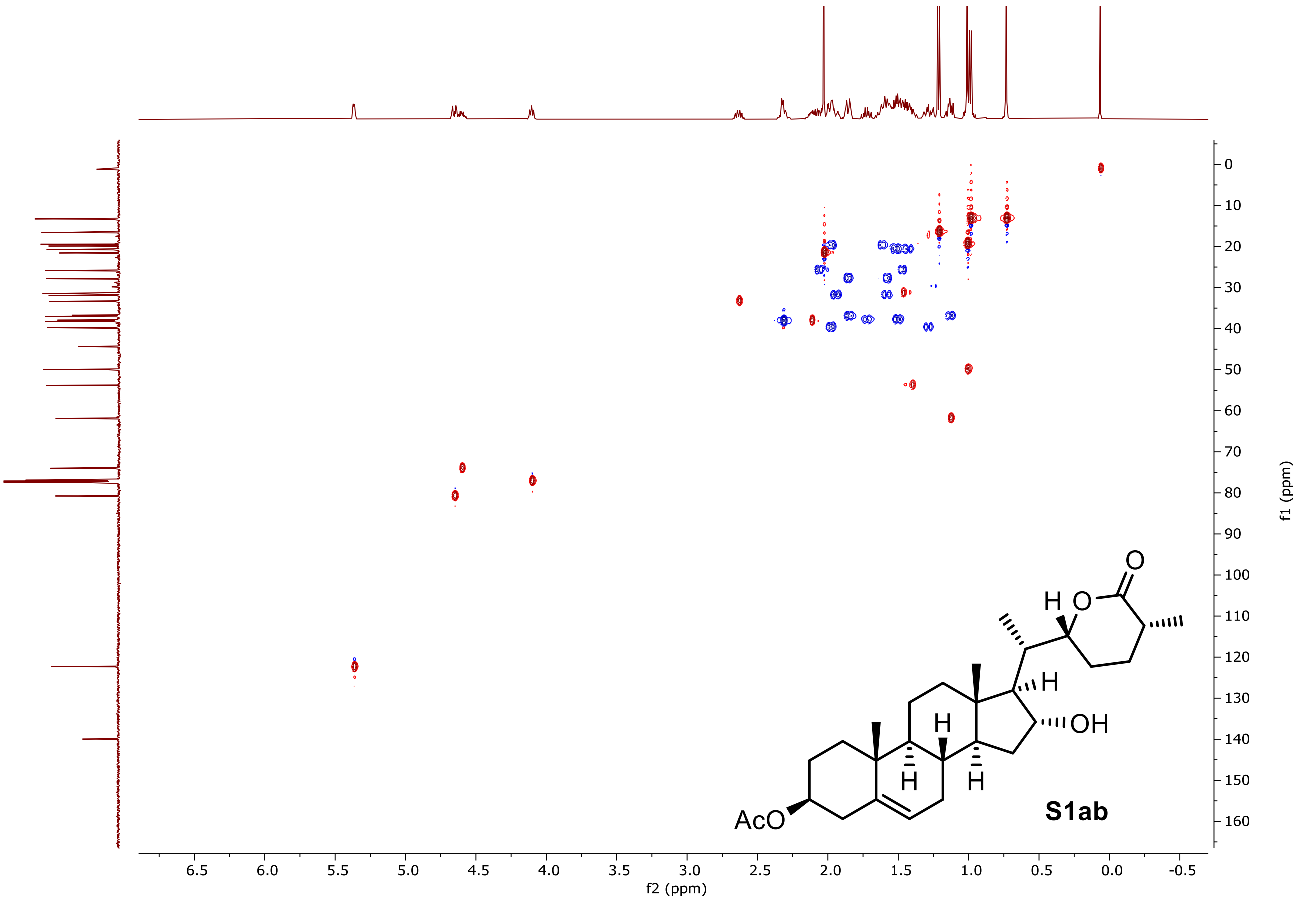

^{1}H NMR (500 MHz, $CDCl_3$) of **7ac** (*see procedure*)

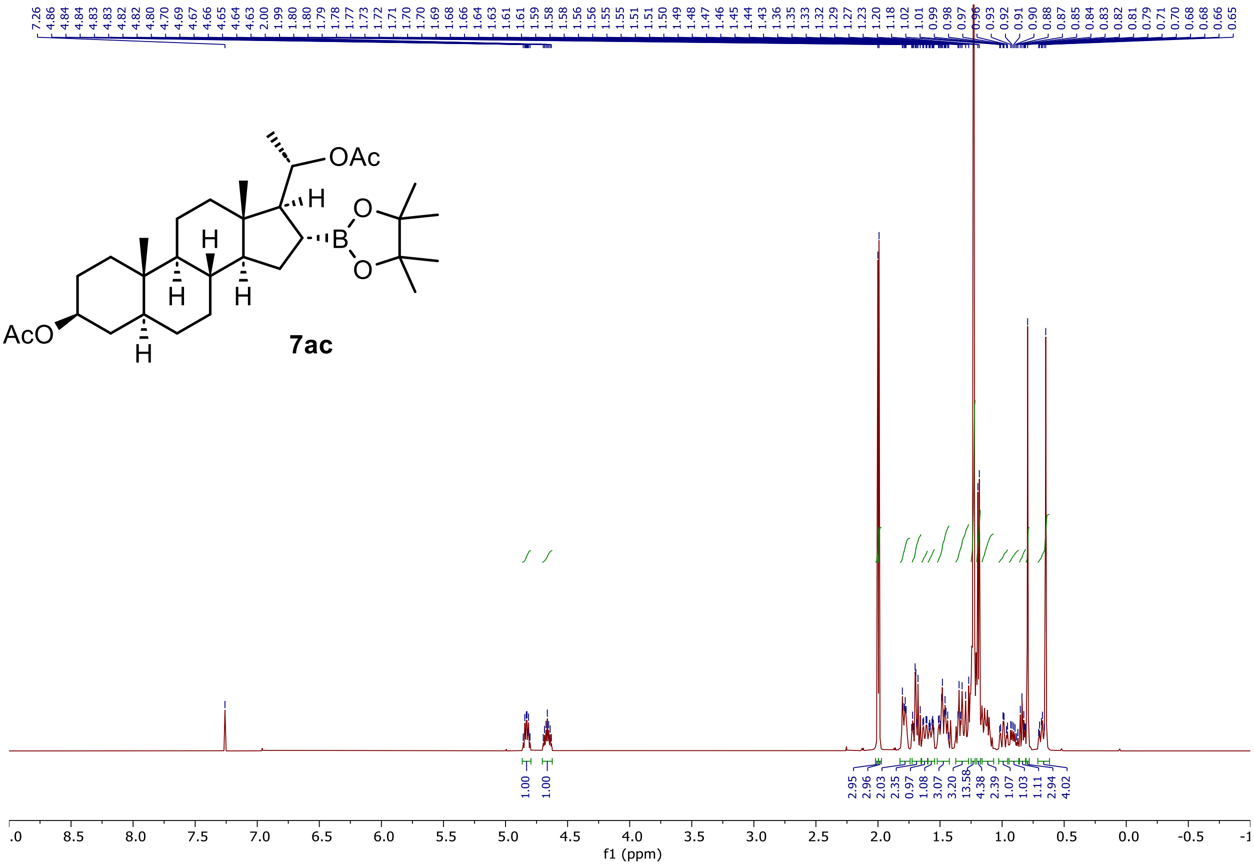

^{13}C NMR (126 MHz, $CDCl_3$) of **7ac**

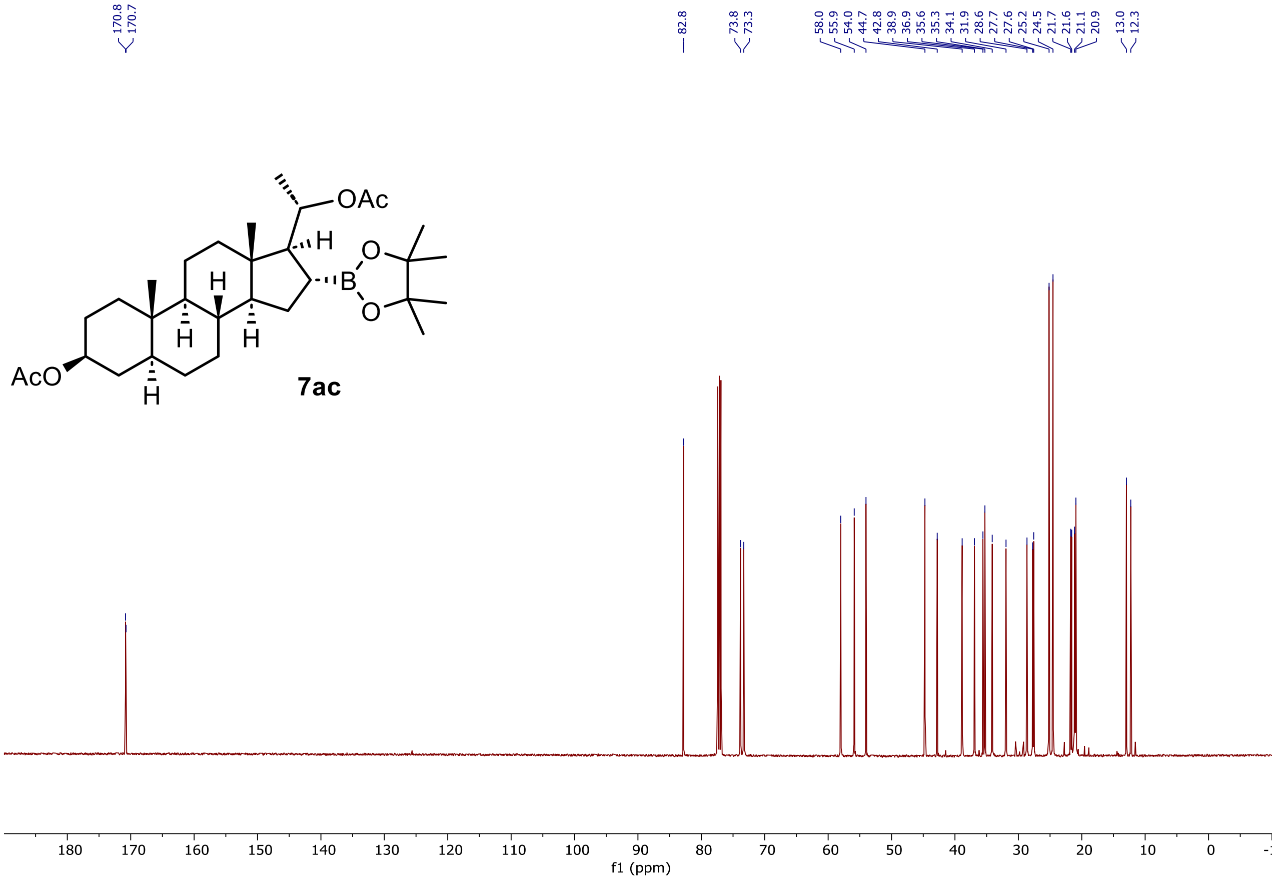

HSQC of **7ac**

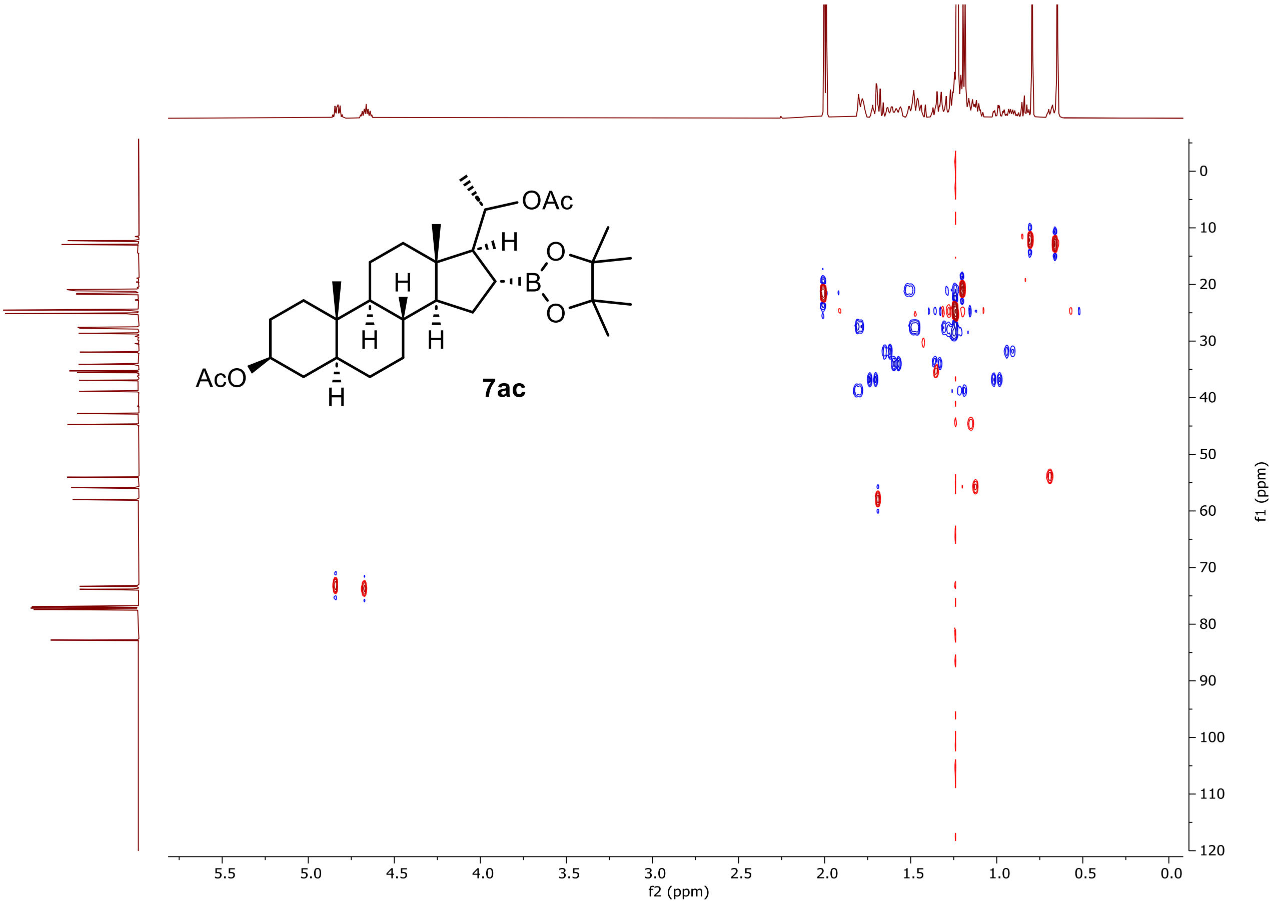

^{1}H NMR (500 MHz, $CDCl_3$) of **S1ac′** (see procedure)

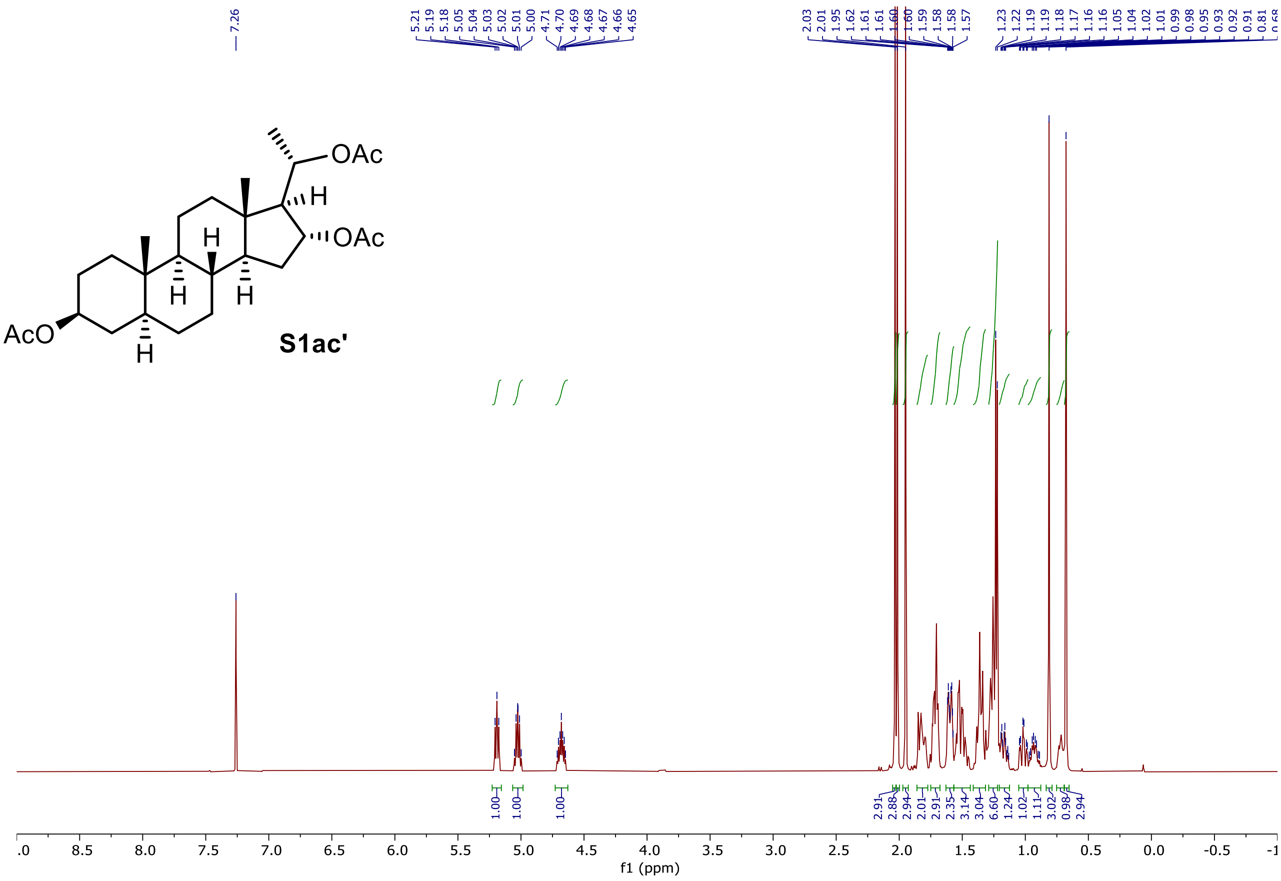

^{13}C NMR (126 MHz, $CDCl_3$) of **S1ac′**

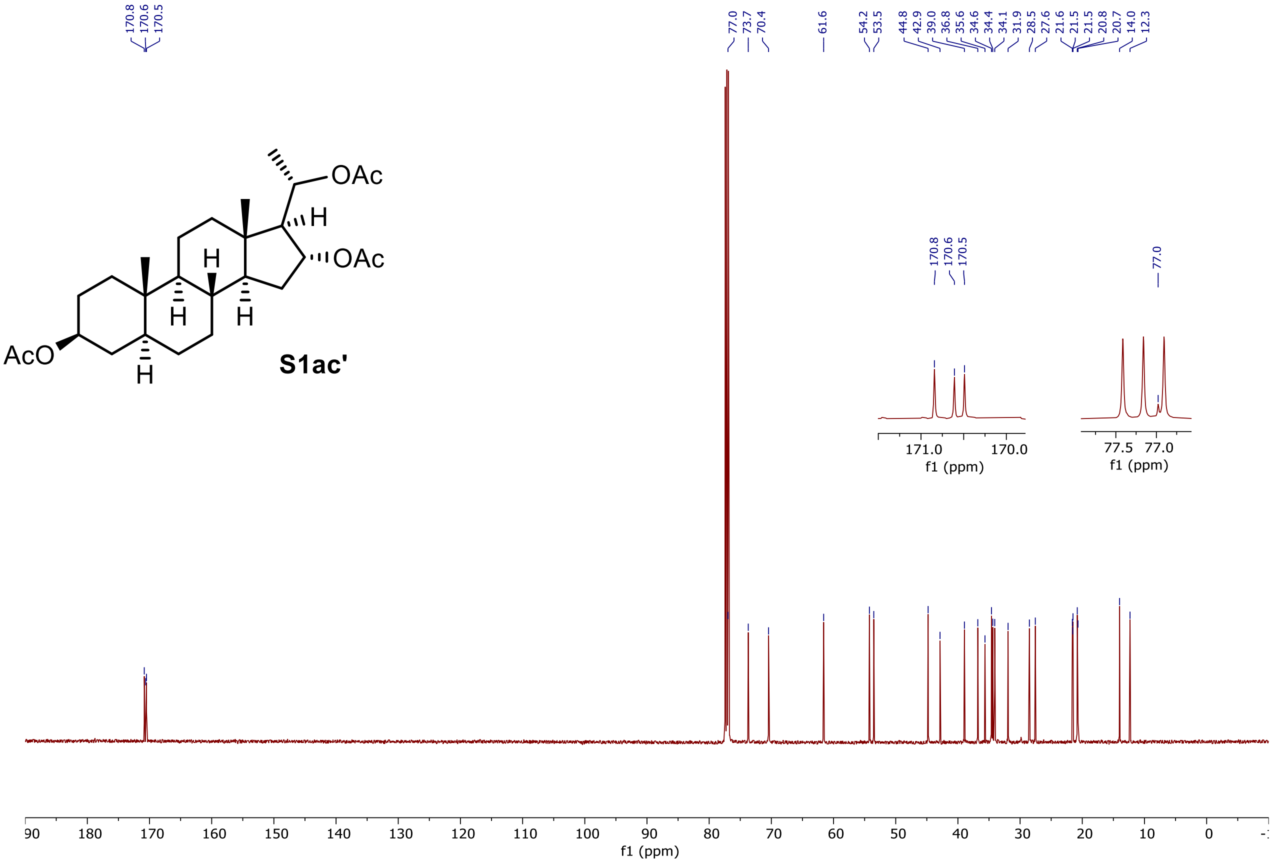

HSQC of **S1ac′**

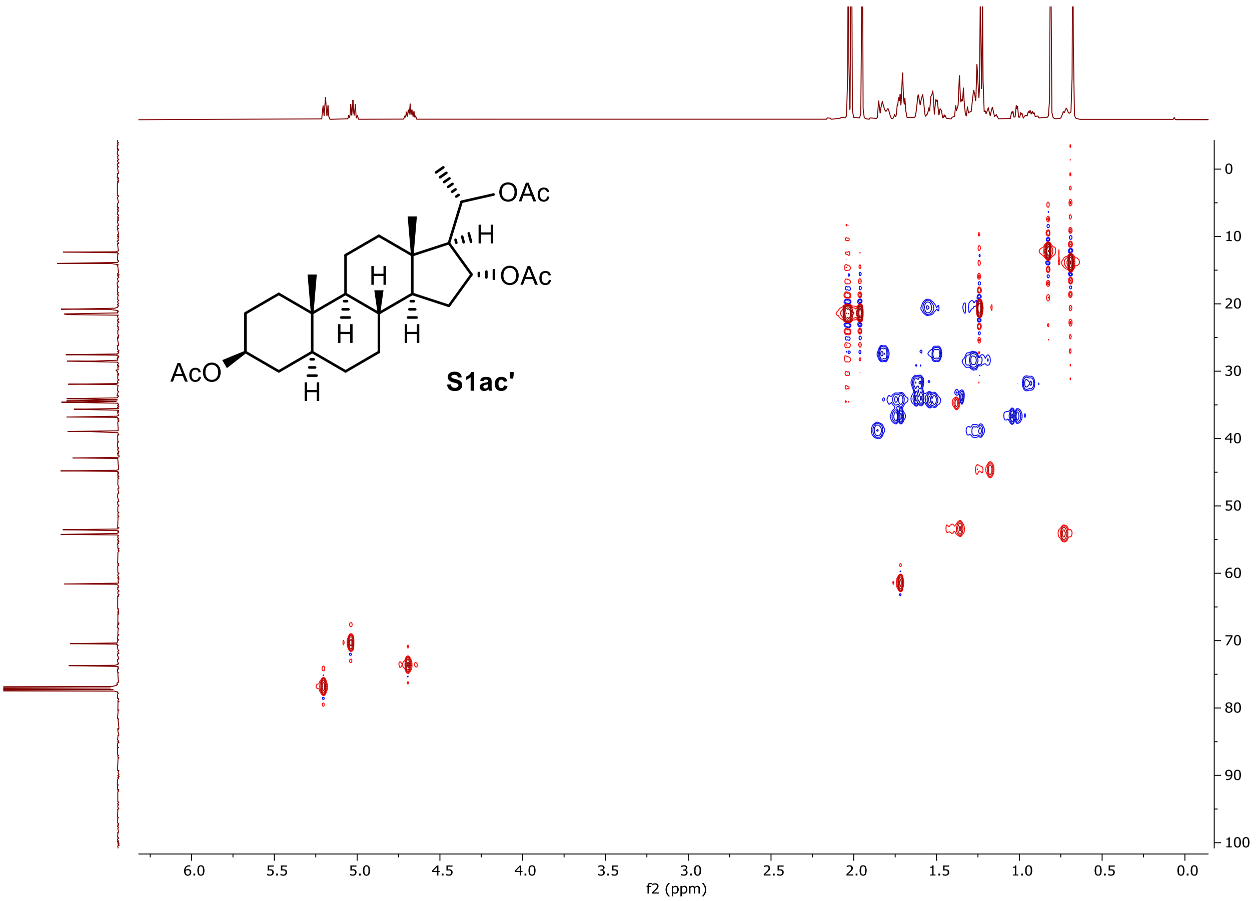

^{1}H NMR (500 MHz, $CDCl_3$) of **7ad** (see procedure)

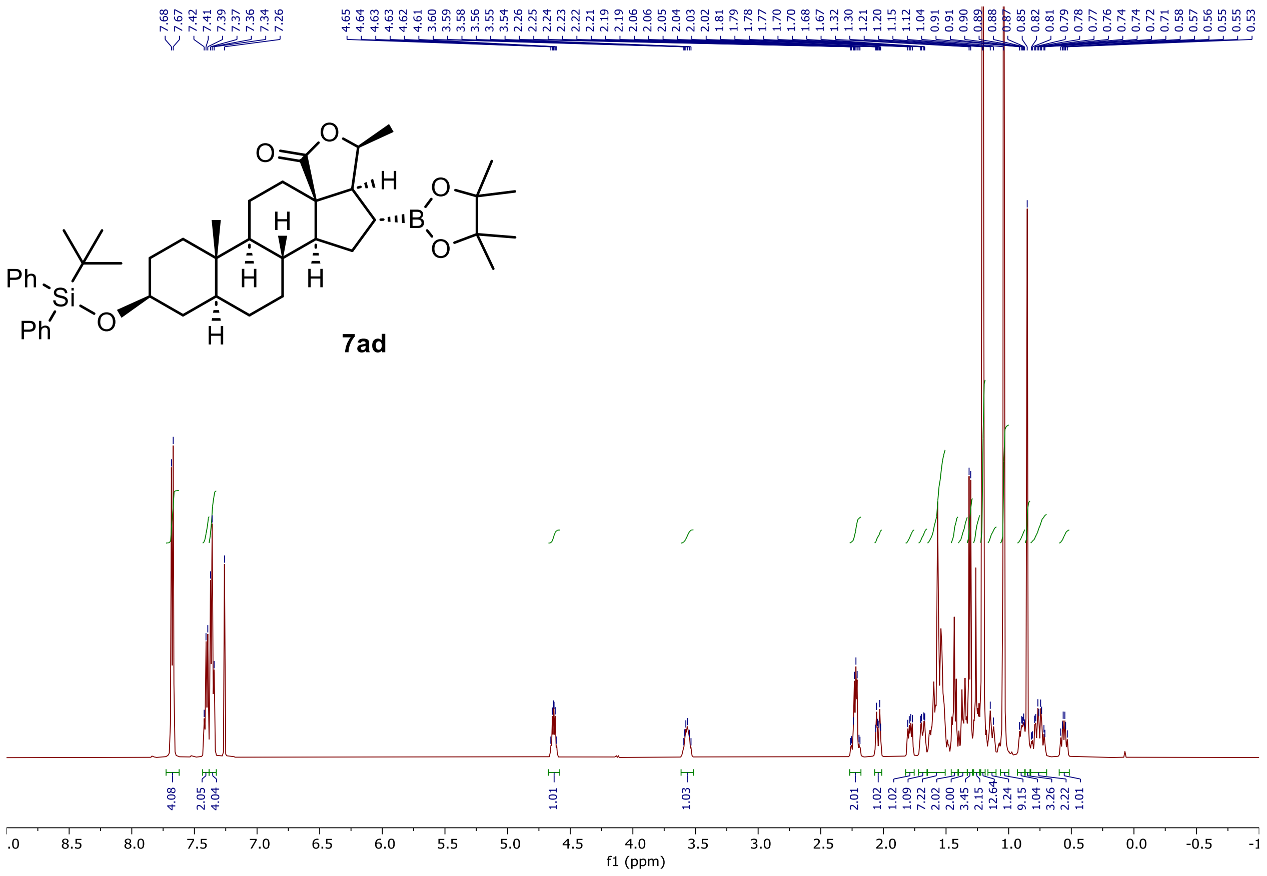

^{13}C NMR (126 MHz, $CDCl_3$) of **7ad**

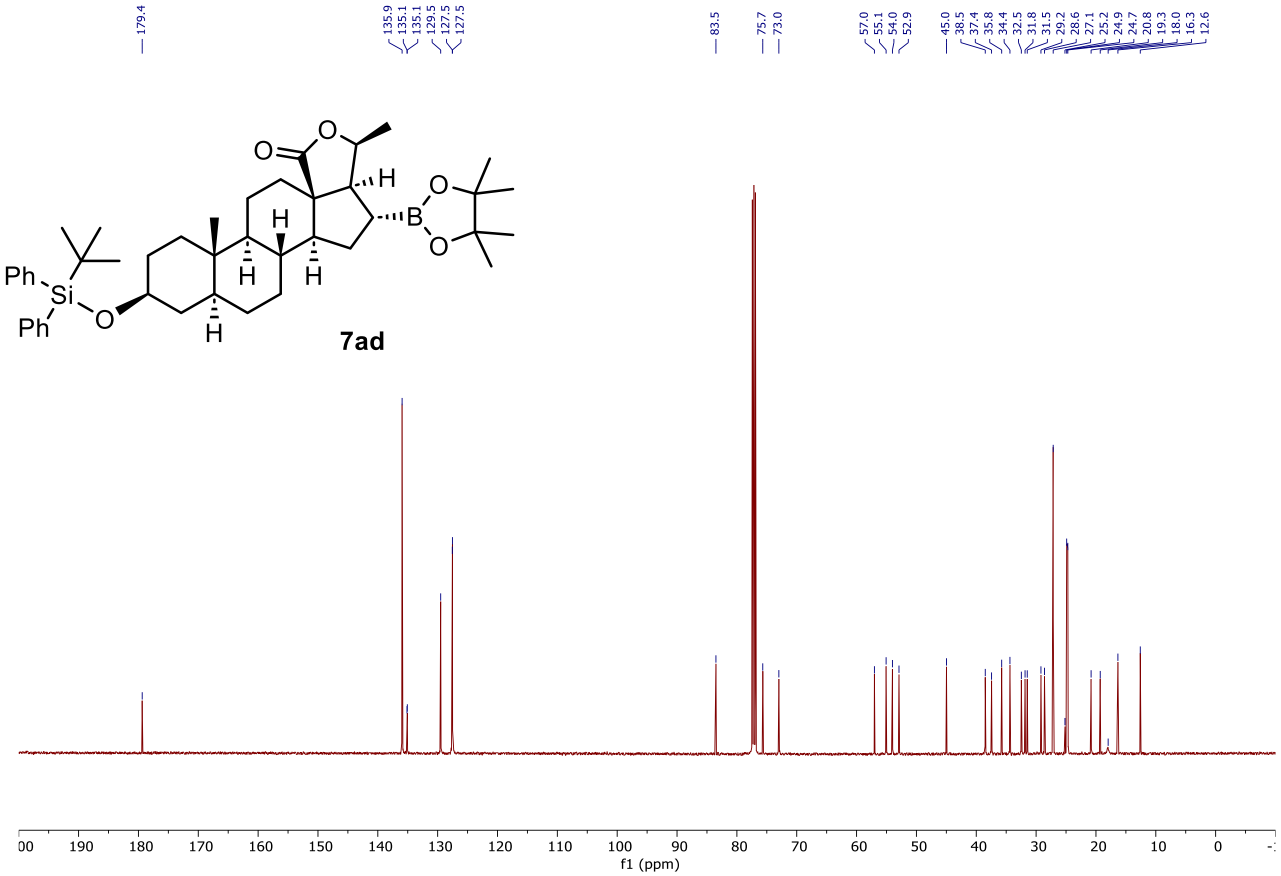

HSQC of **7ad**

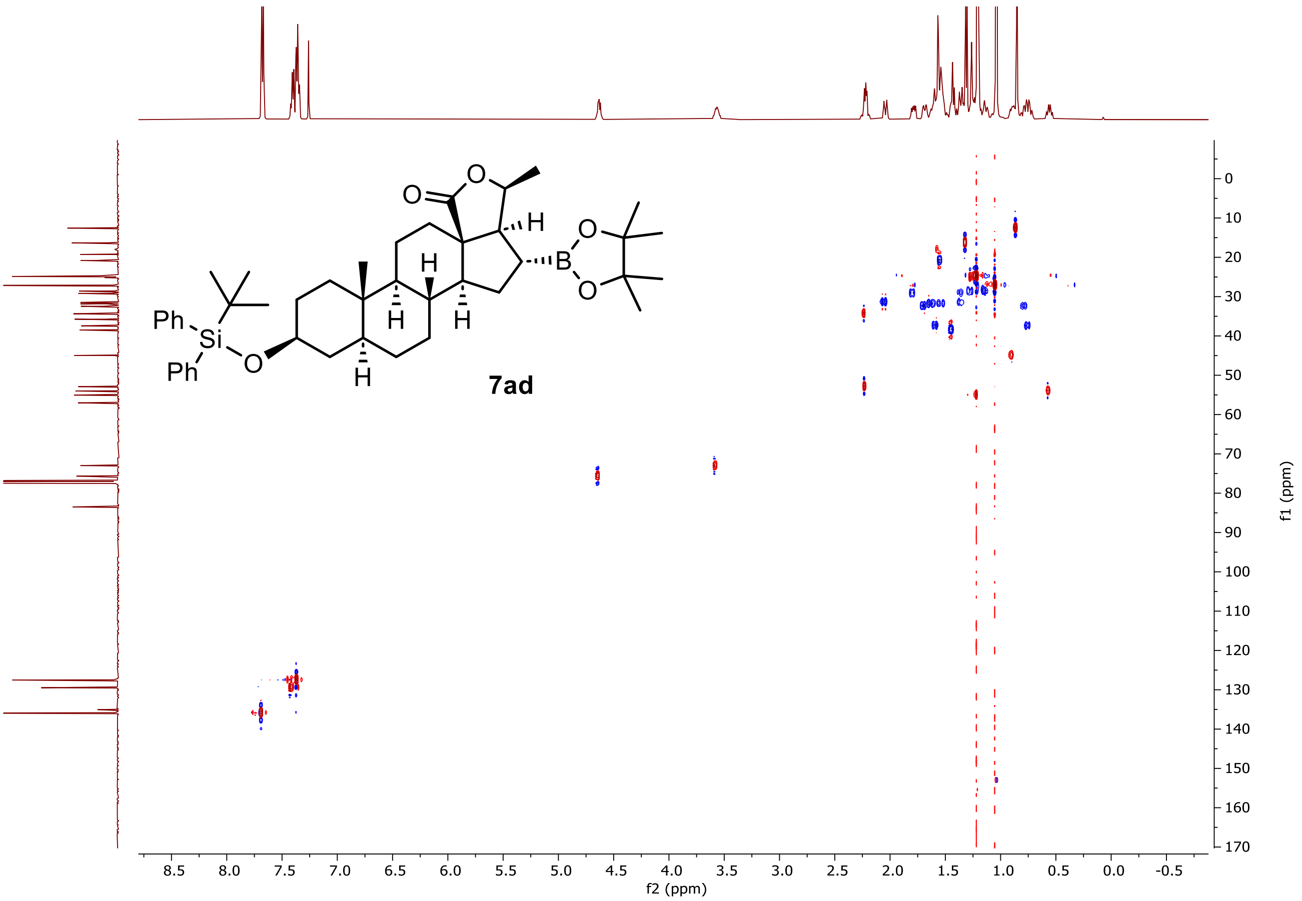
Ph
Si
Ph
O
H
H
H
H
O
O
H
B
O
O
7ad
f1 (ppm)
f2 (ppm)
0
10
20
30
40
50
60
70
80
90
100
110
120
130
140
150
160
170
8.5
8.0
7.5
7.0
6.5
6.0
5.5
5.0
4.5
4.0
3.5
3.0
2.5
2.0
1.5
1.0
0.5
0.0
-0.5

^{1}H NMR (500 MHz, $CDCl_3$) of **7ae** (<u>*see procedure*</u>)

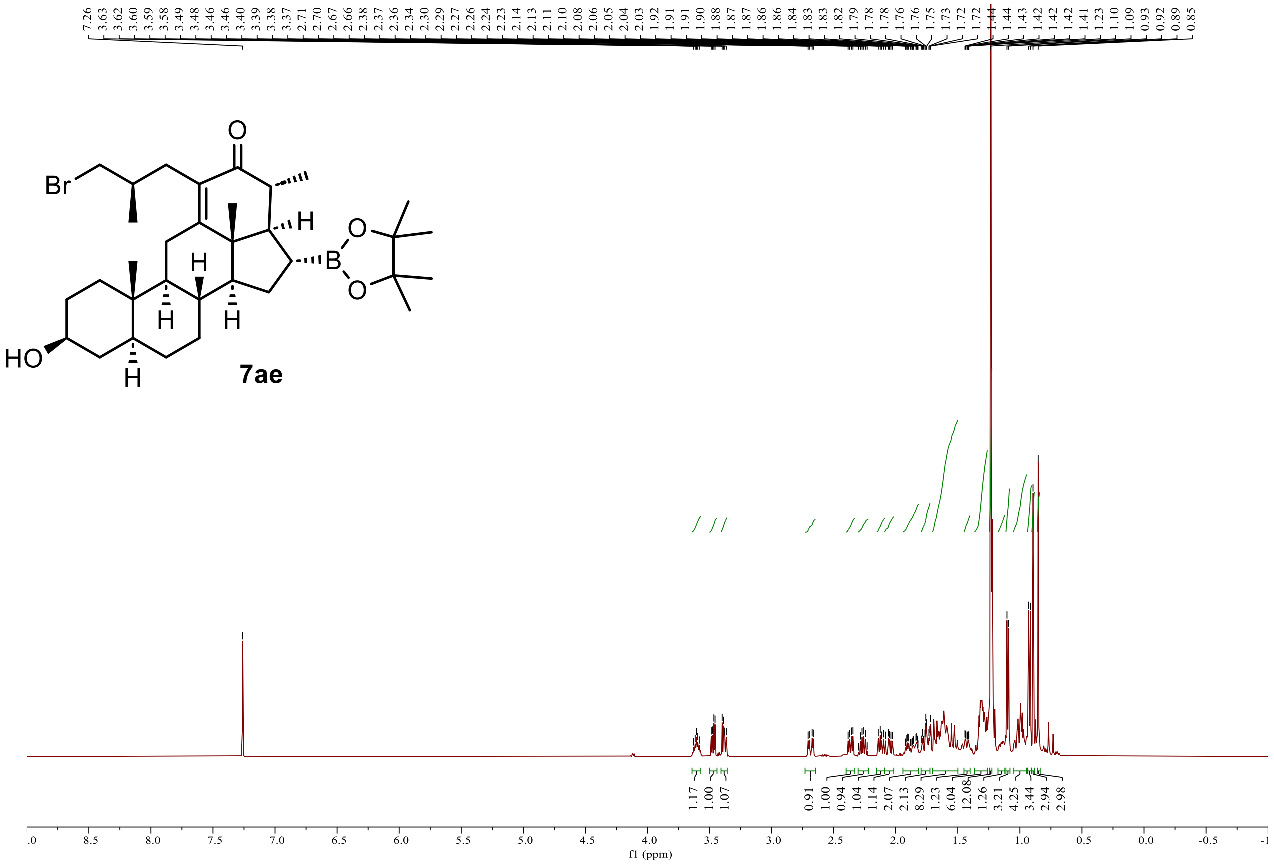

^{13}C NMR (126 MHz, $CDCl_3$) of **7ae**

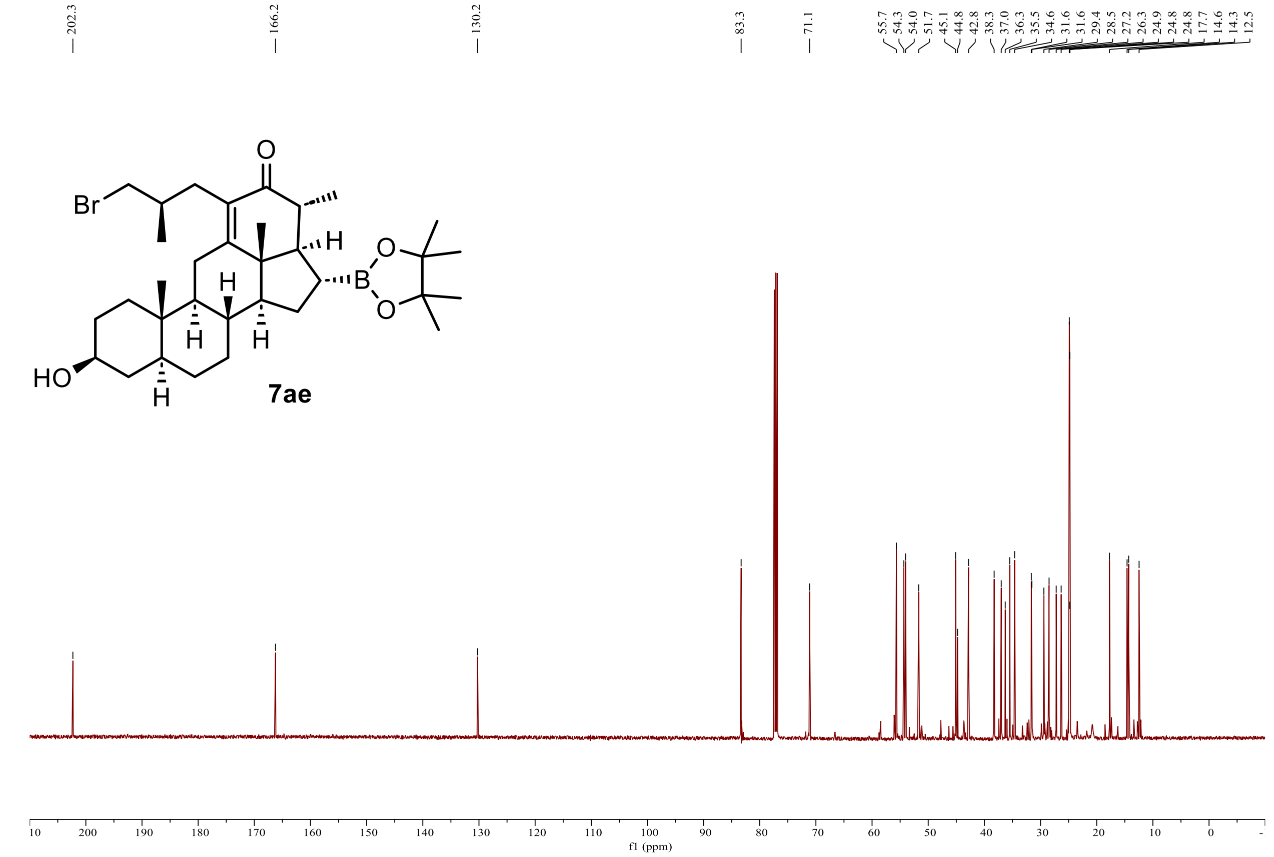

HSQC of **7ae**

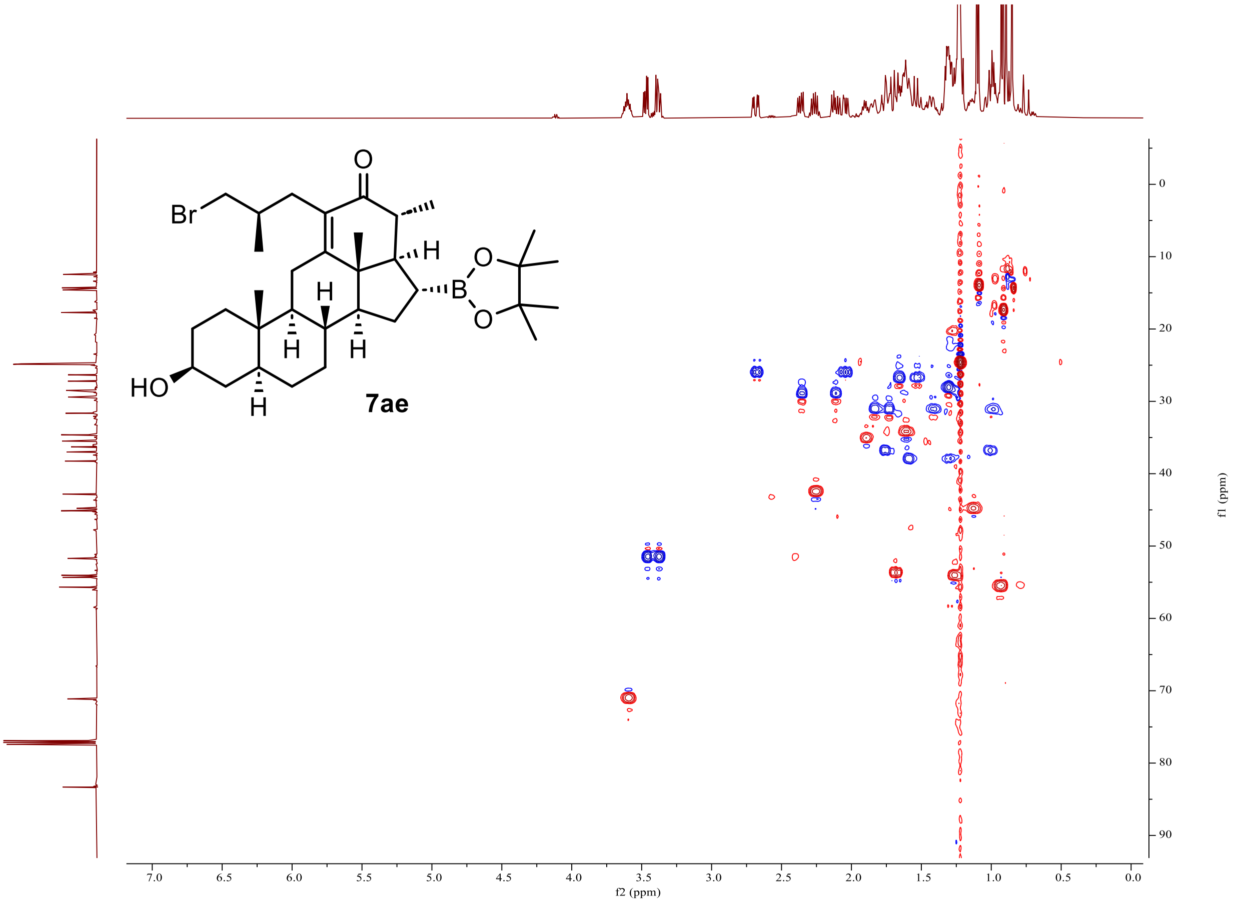

^{1}H NMR (500 MHz, $CDCl_3$) of **S1ae′** (<u>*see procedure*</u>)

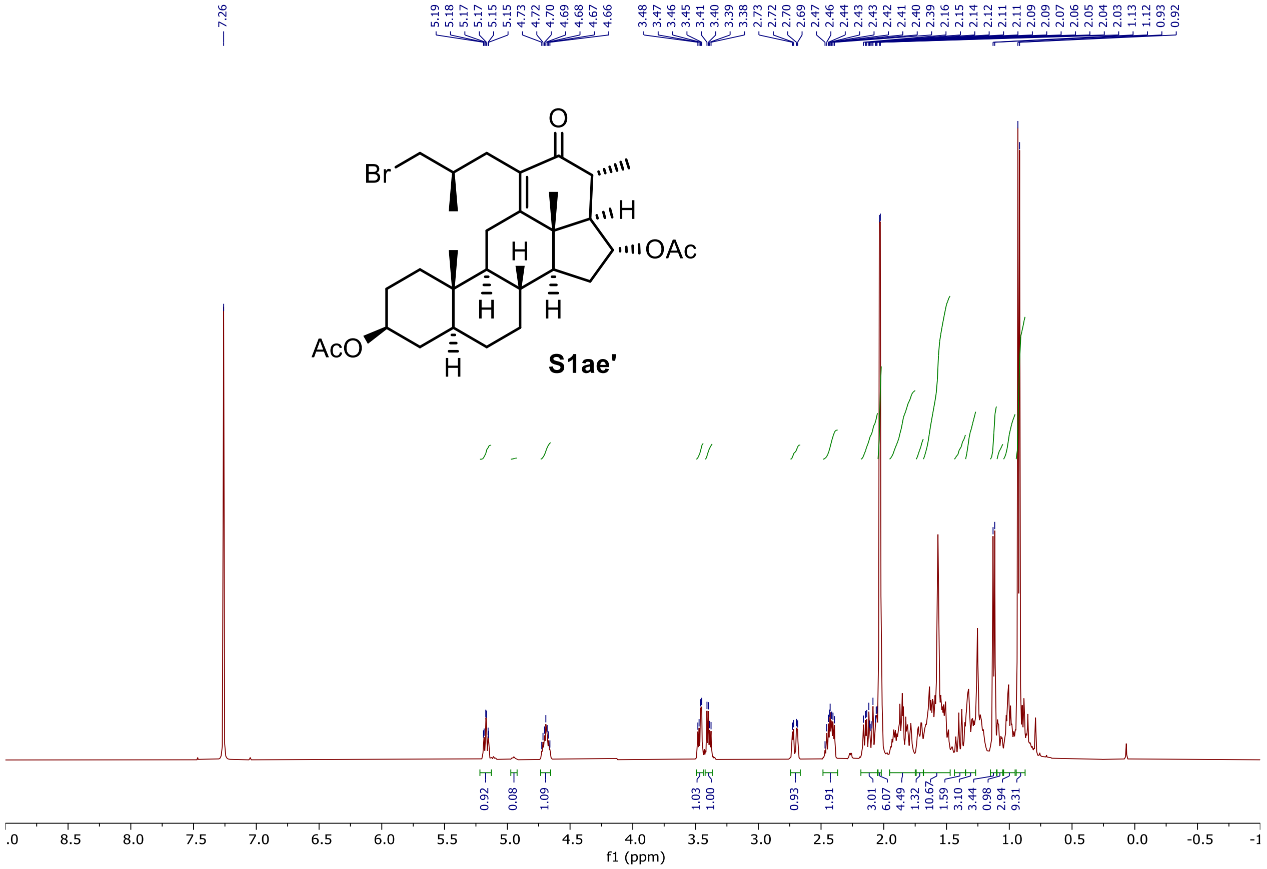

^{13}C NMR (126 MHz, $CDCl_3$) of **S1ae′**

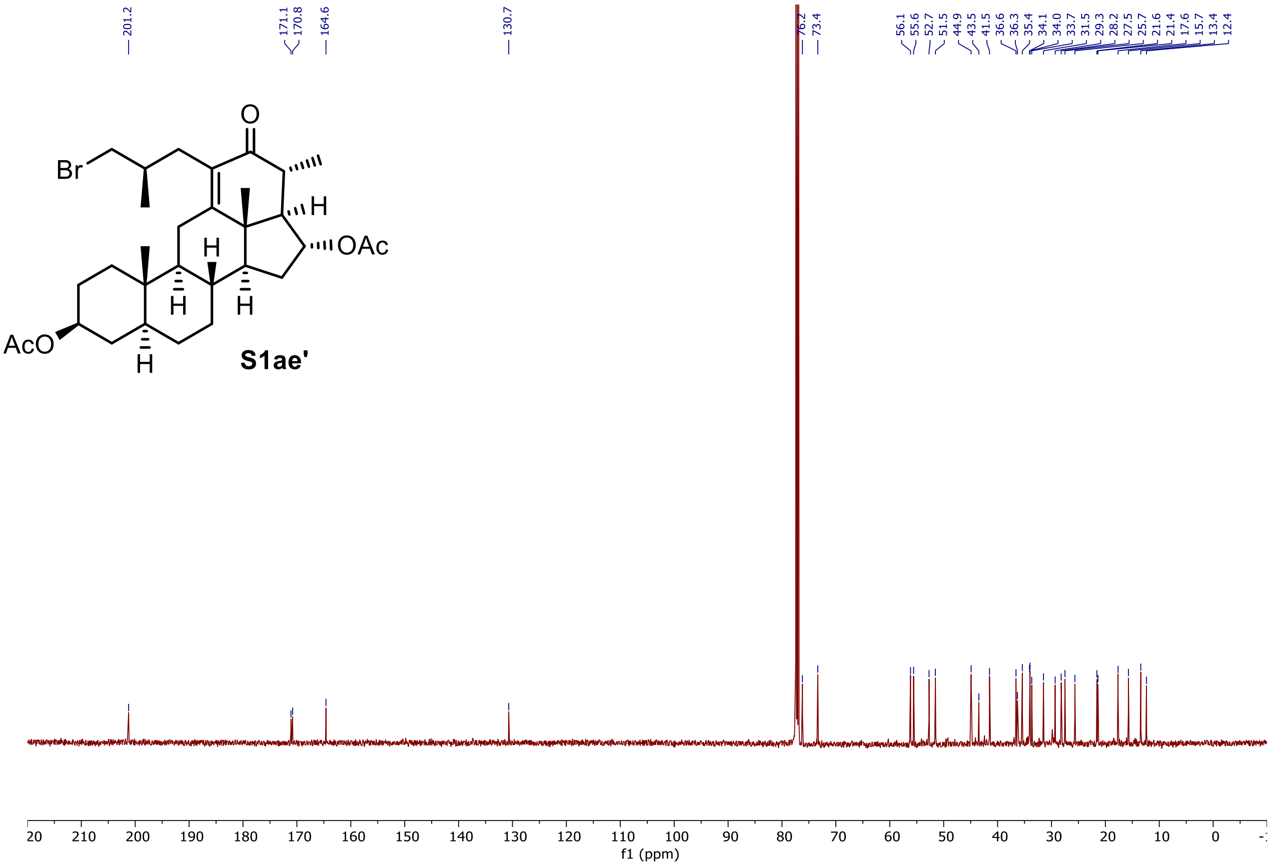

HSQC of **S1ae′**

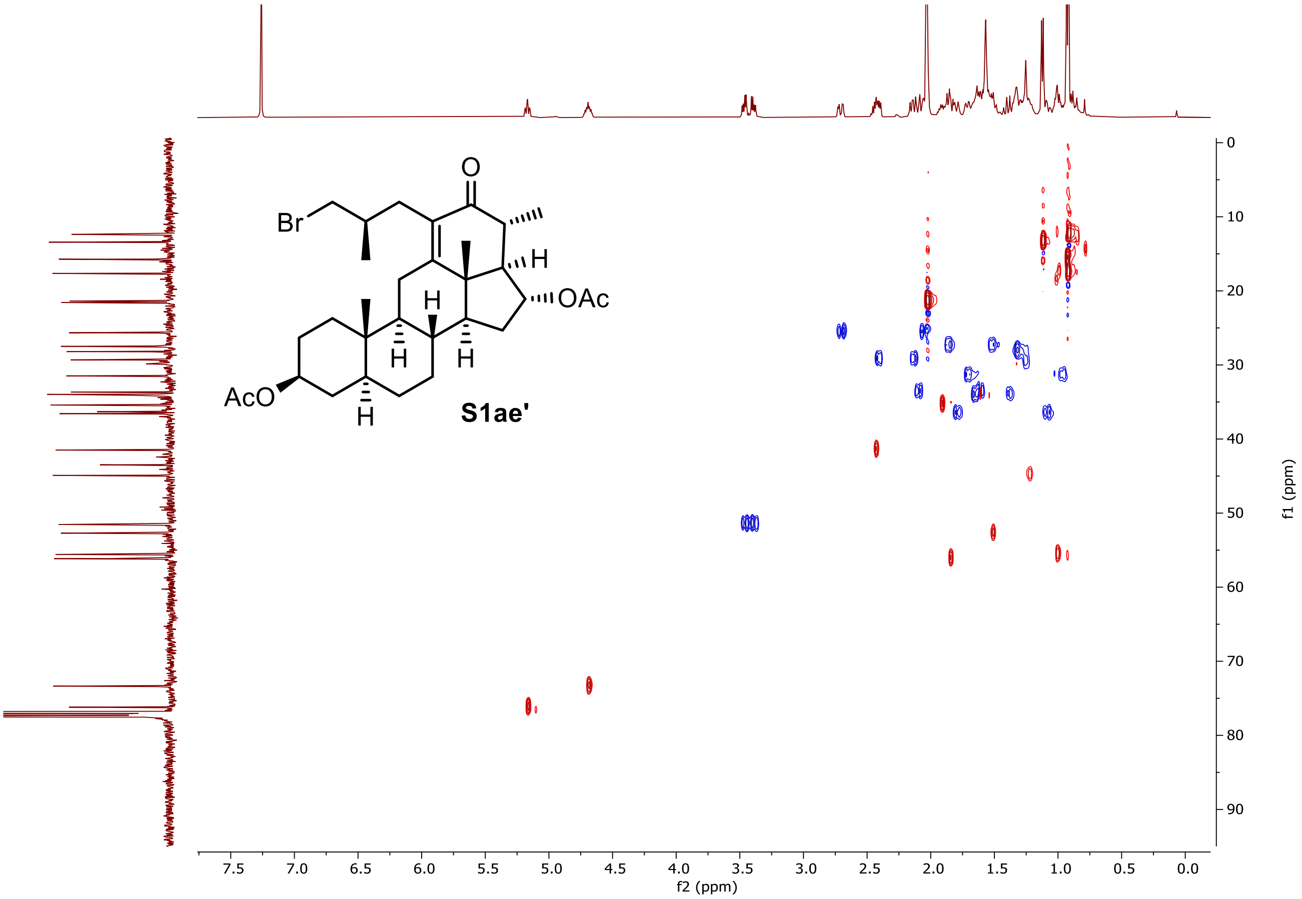
Br
O
H
OAc
H
H
H
AcO
H
S1ae'
7.5
7.0
6.5
6.0
5.5
5.0
4.5
4.0
3.5
3.0
2.5
2.0
1.5
1.0
0.5
0.0
f2 (ppm)
0
10
20
30
40
50
60
70
80
90
f1 (ppm)

^{1}H NMR (500 MHz, $CDCl_3$) of **7ae-2** (*see procedure*)

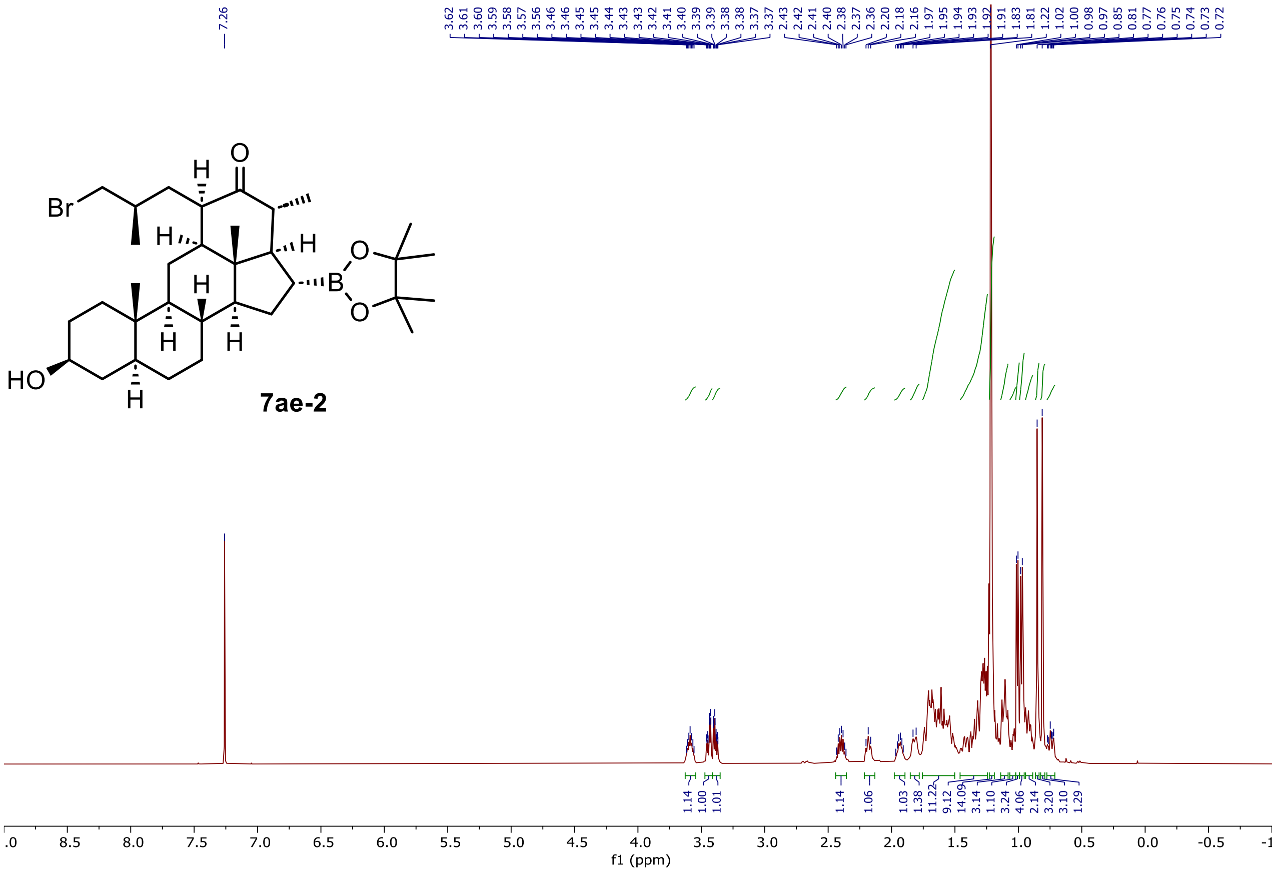

^{13}C NMR (126 MHz, $CDCl_3$) of **7ae-2**

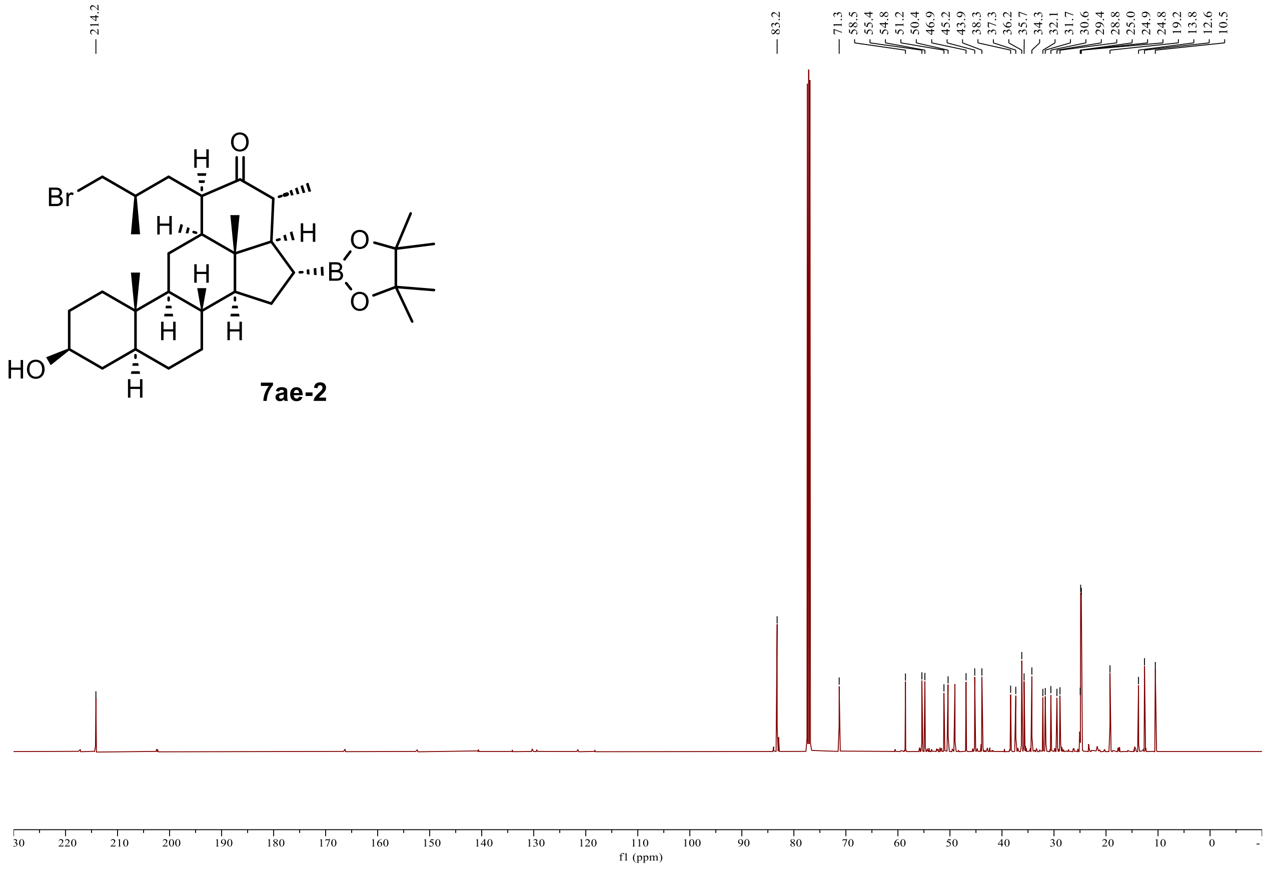

HSQC of **7ae-2**

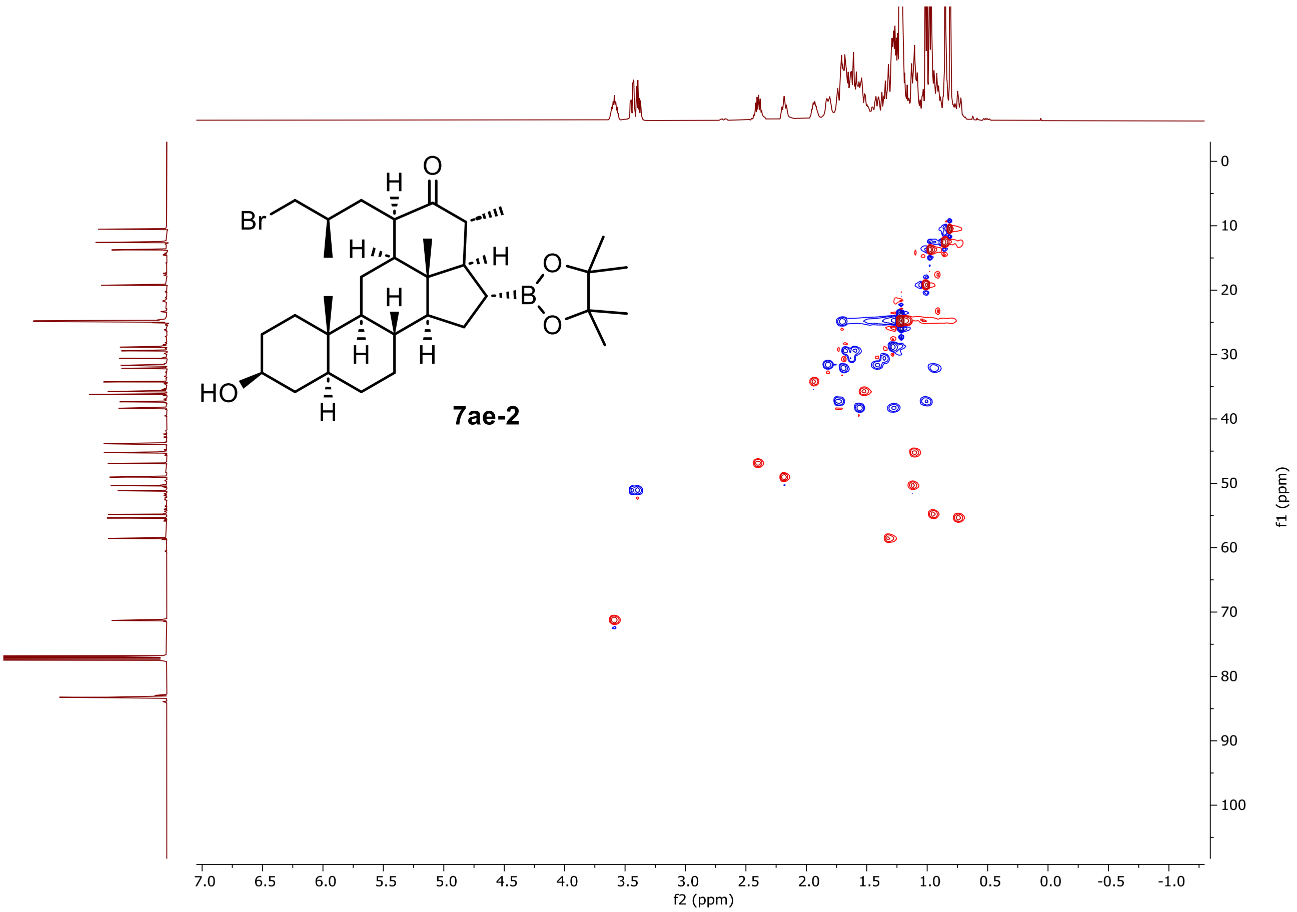

^{1}H NMR (500 MHz, $CDCl_3$) of **7ae-3** (*see procedure*)

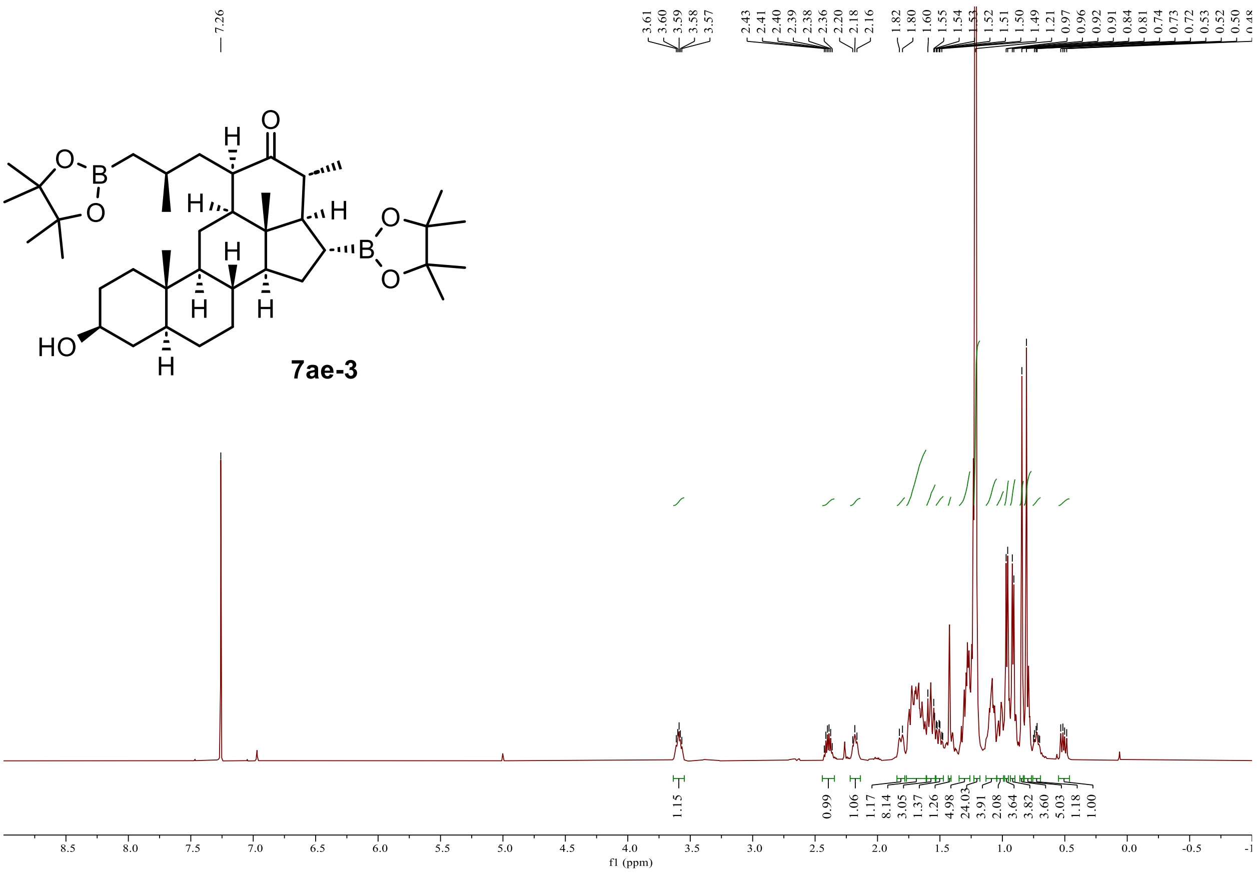

^{13}C NMR (126 MHz, $CDCl_3$) of **7ae-3**

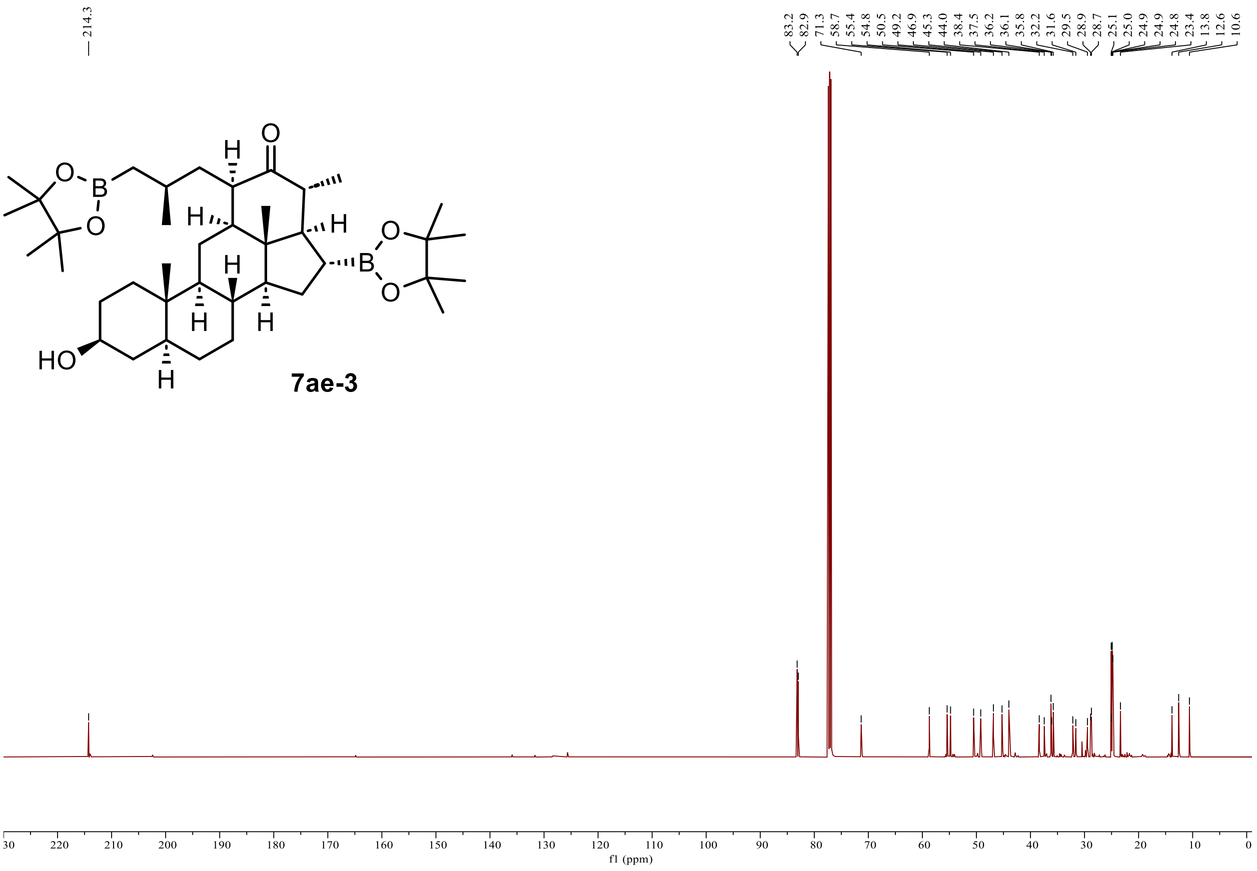

HSQC of **7ae-3**

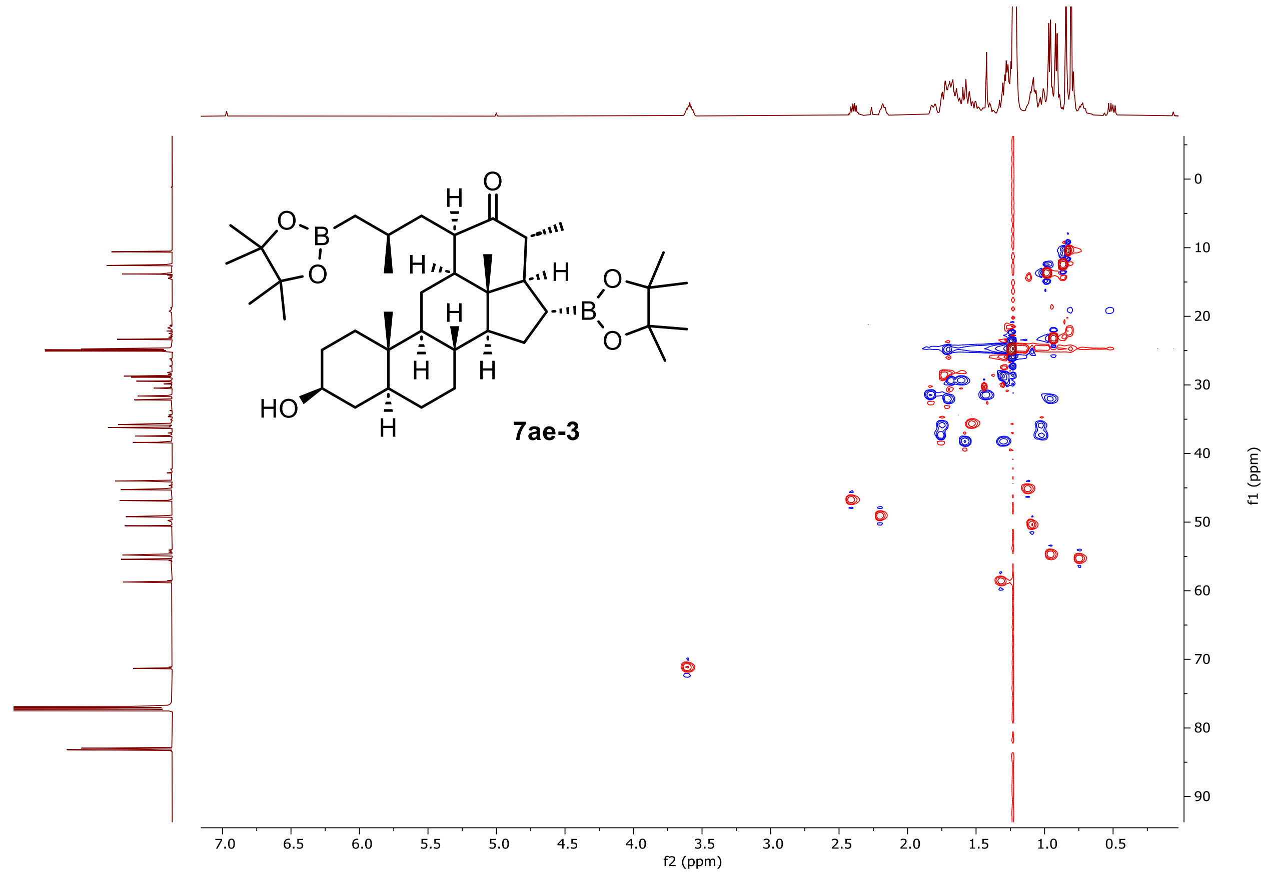

NOSEY of **7ae-3**

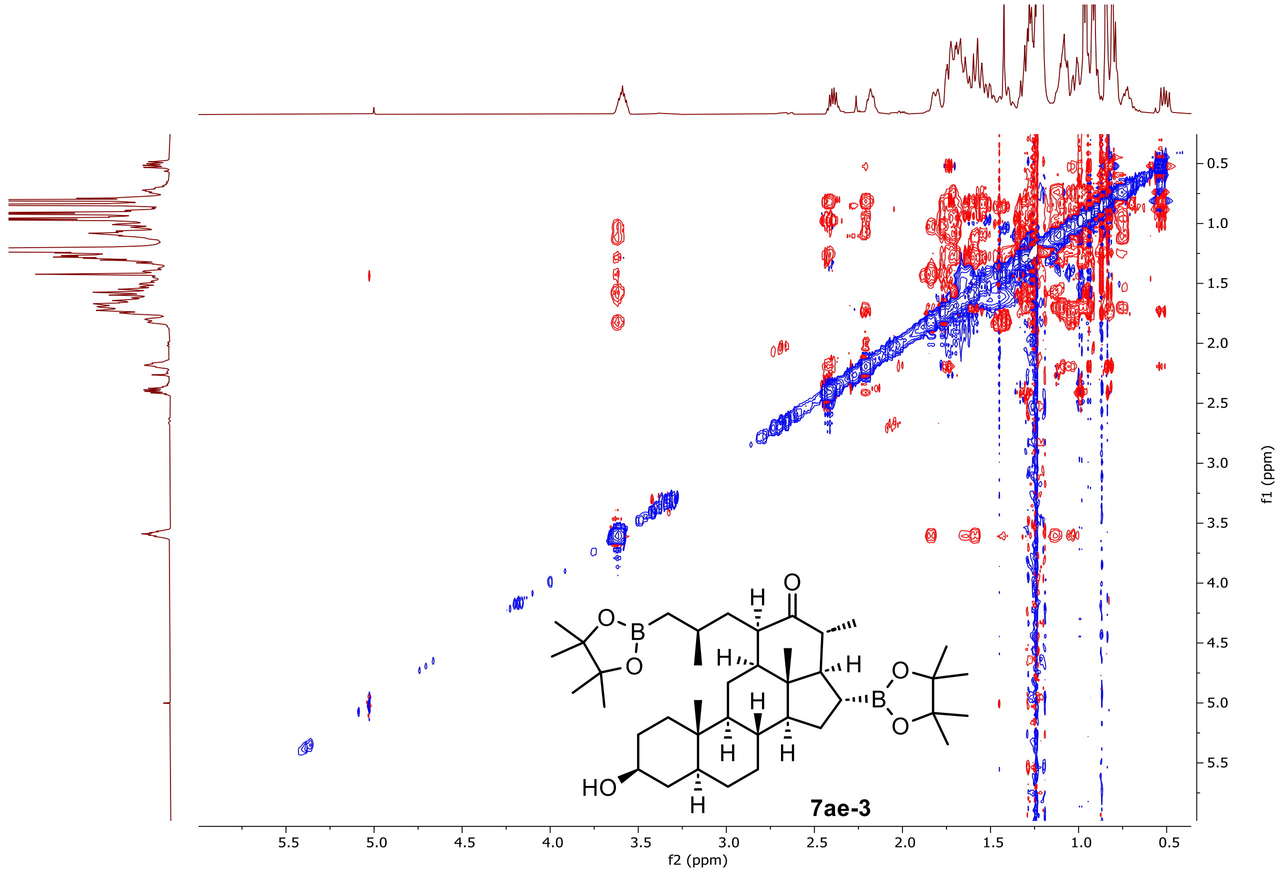

^{1}H NMR (500 MHz, $CDCl_3$) of **18** (*see procedure*)

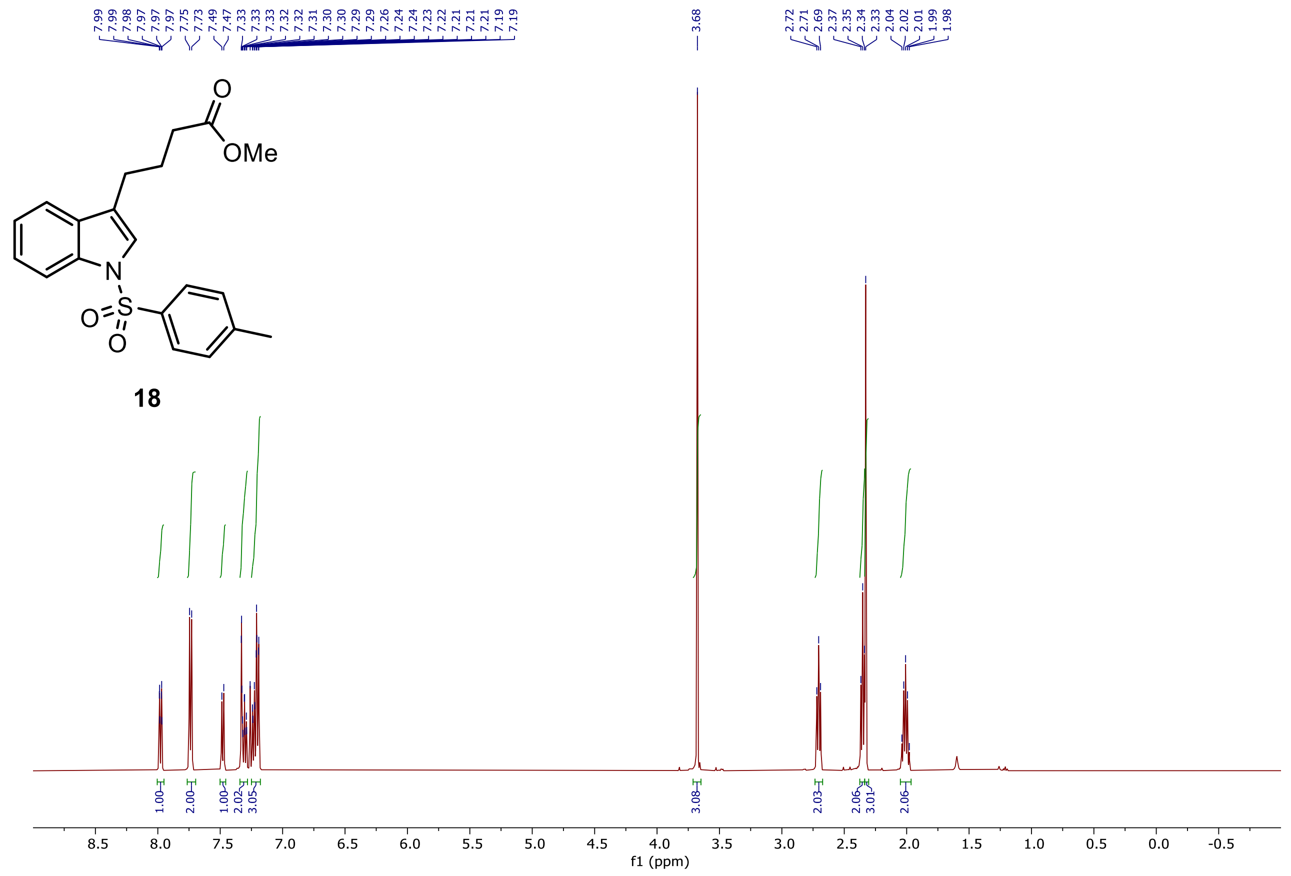

^{13}C NMR (126 MHz, $CDCl_3$) of **18**

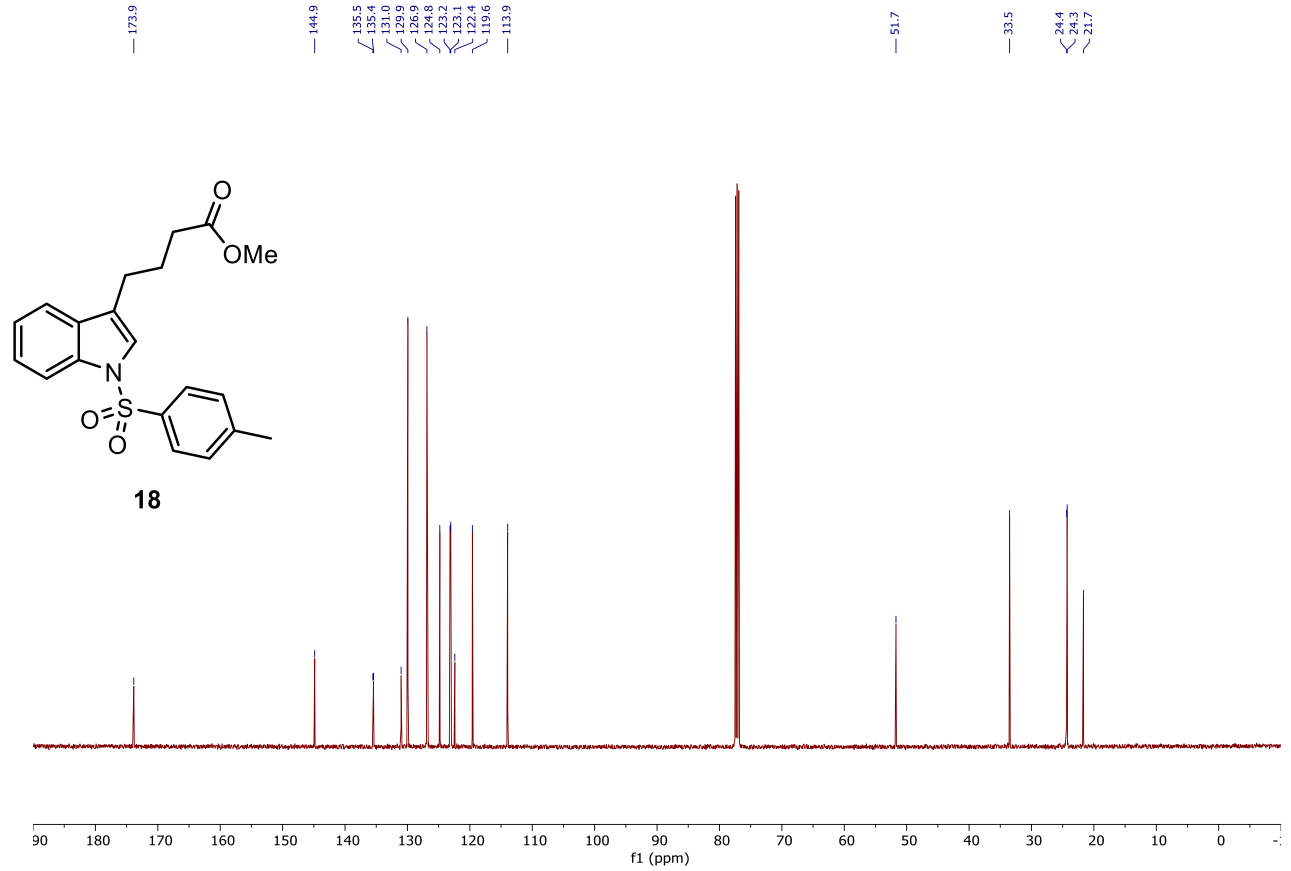

^{1}H NMR (400 MHz, $CDCl_3$) of **19** (*see procedure*)

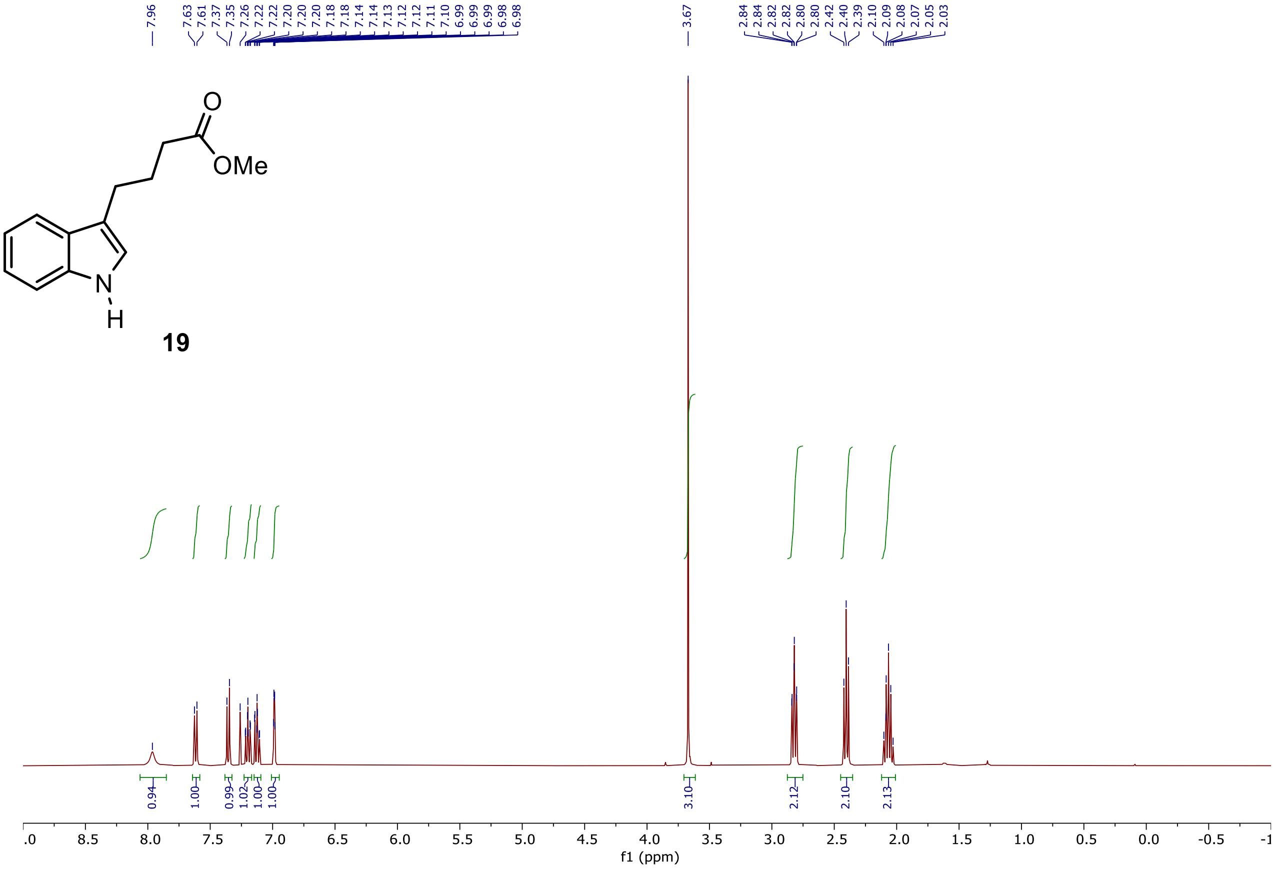

^{13}C NMR (101 MHz, $CDCl_3$) of **19**

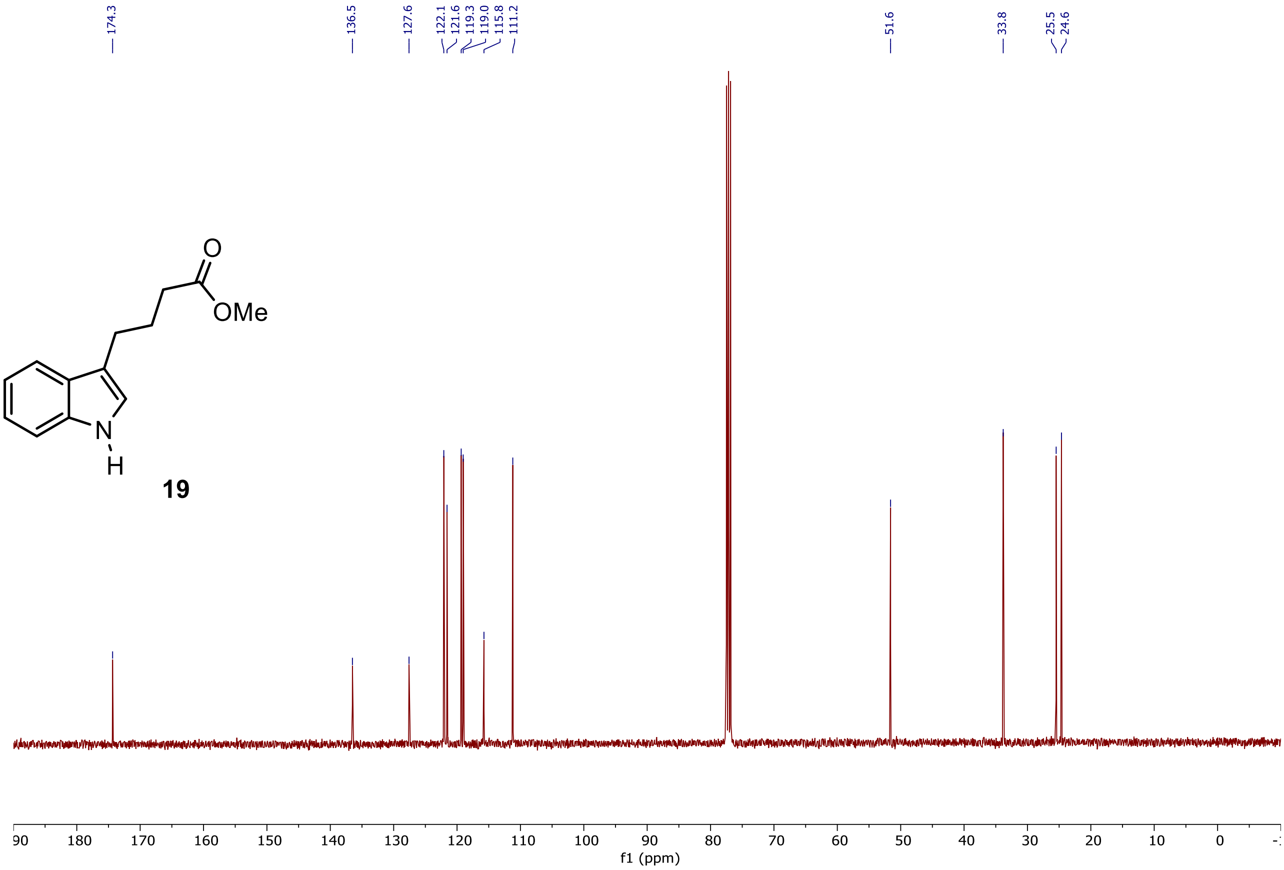

^{1}H NMR (400 MHz, acetone-d_6) of **8a•**PF_6 (see procedure)

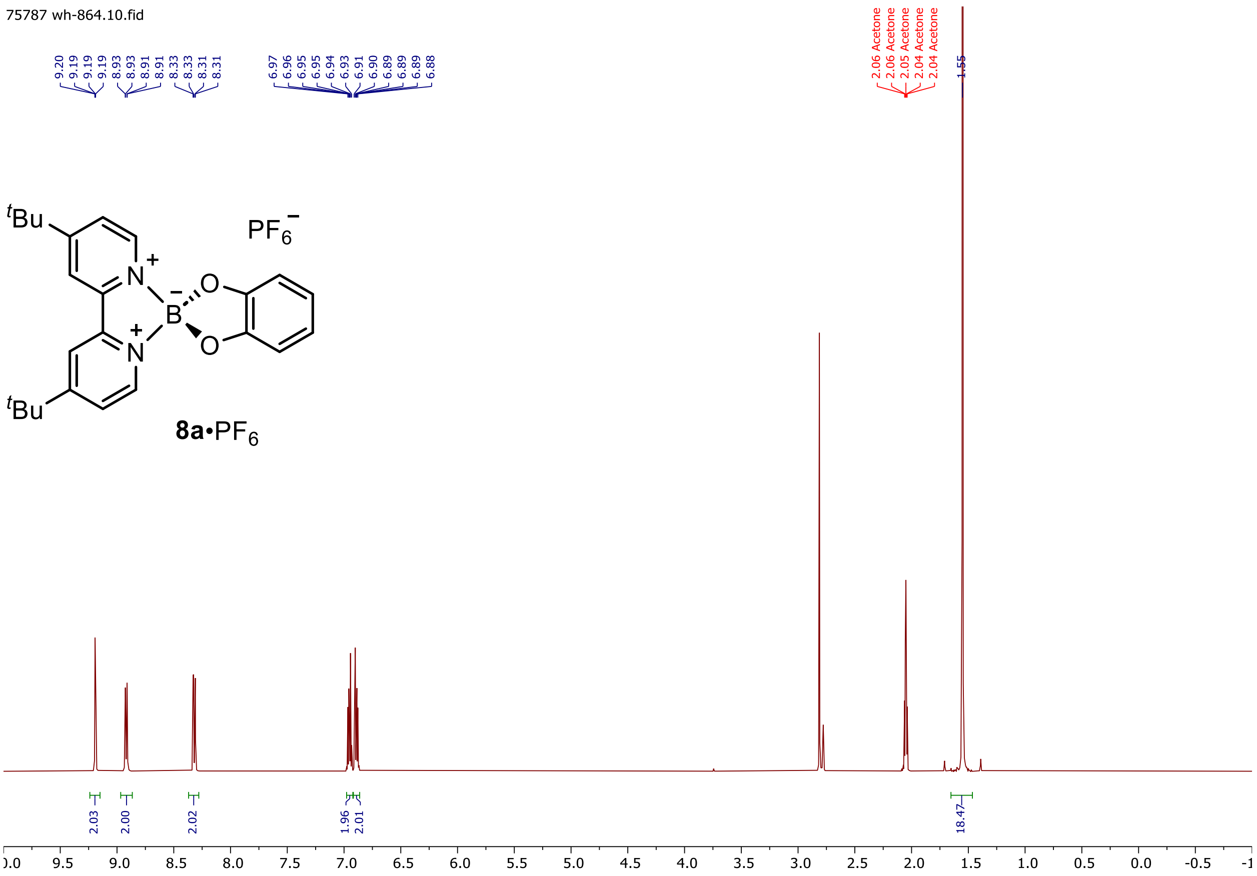

^{13}C NMR (101 MHz, acetone-d_6) of **8a•**PF_6

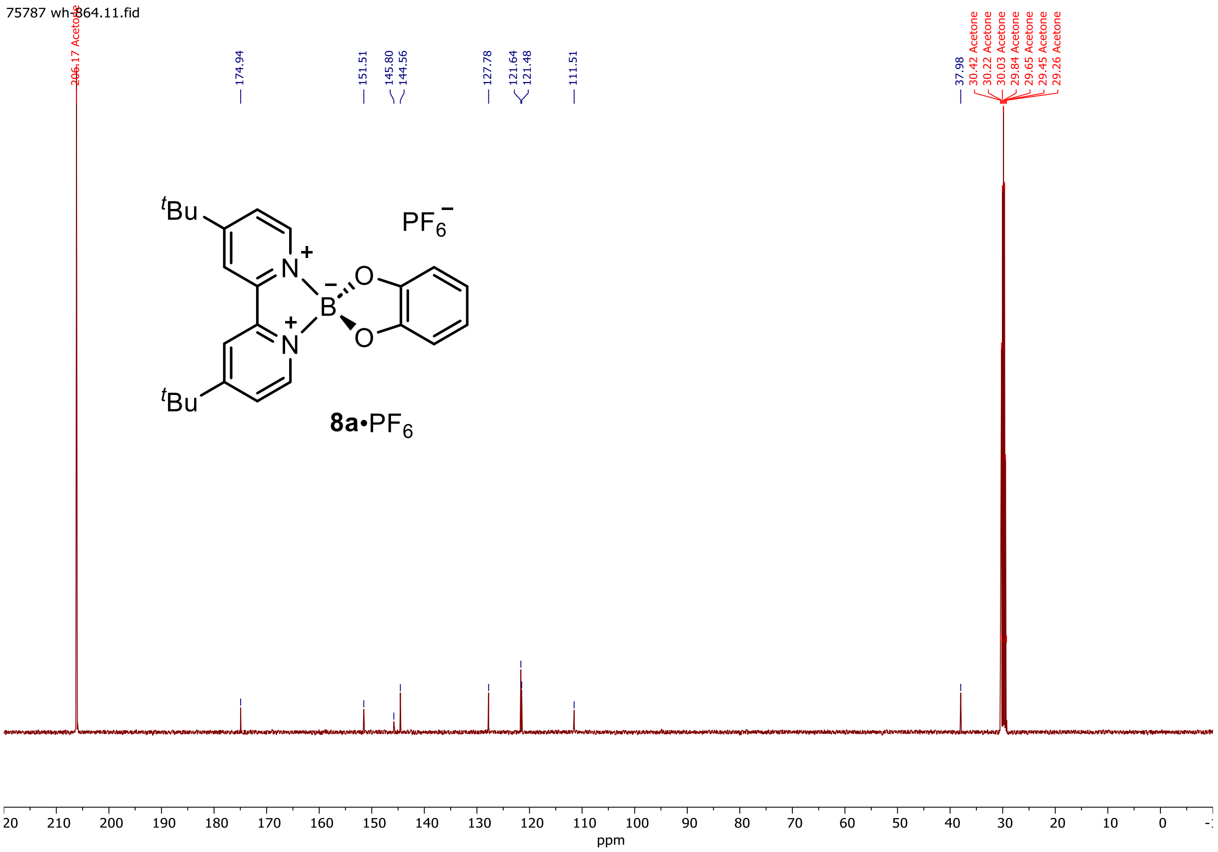

^{31}P NMR (162 MHz, acetone-d_6) of **8a**•PF_6

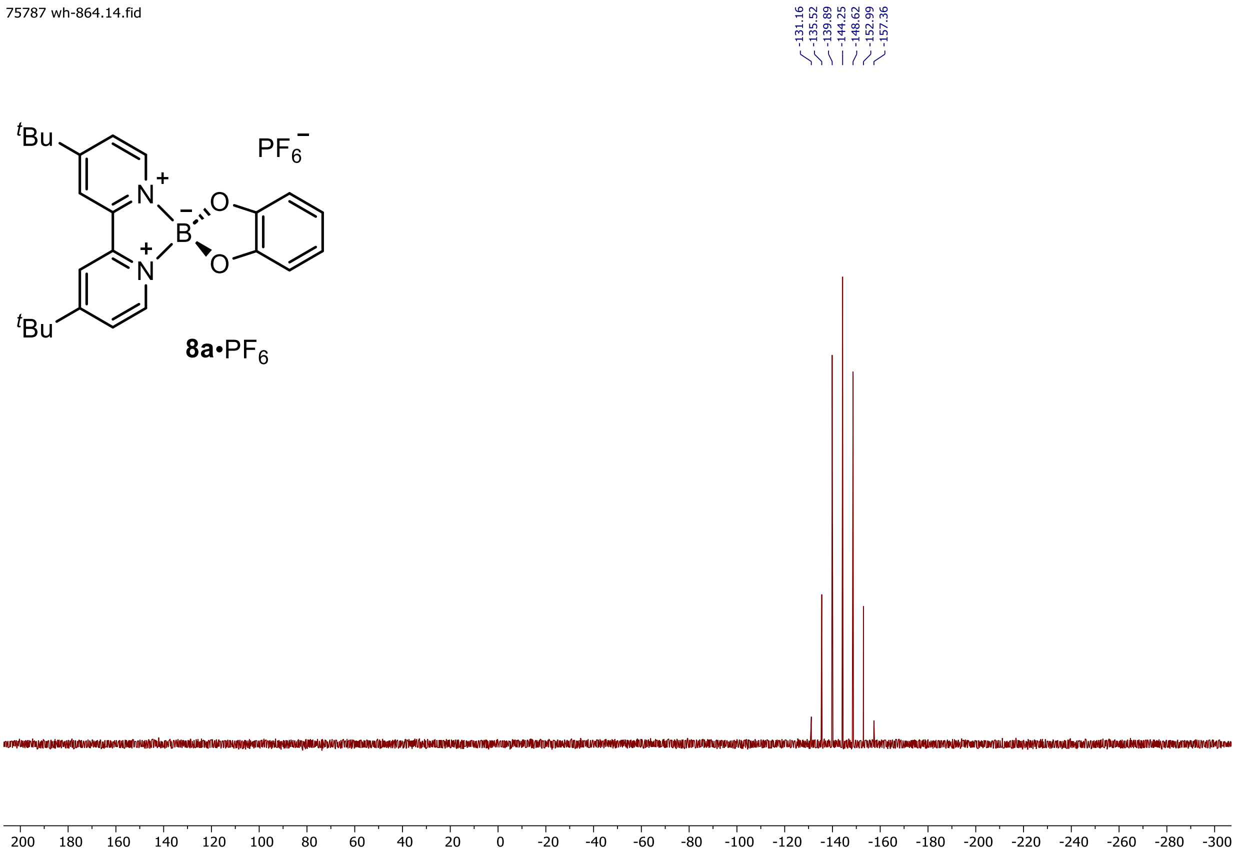

^{19}F NMR (377 MHz, acetone-d_6) of **8a**•PF_6

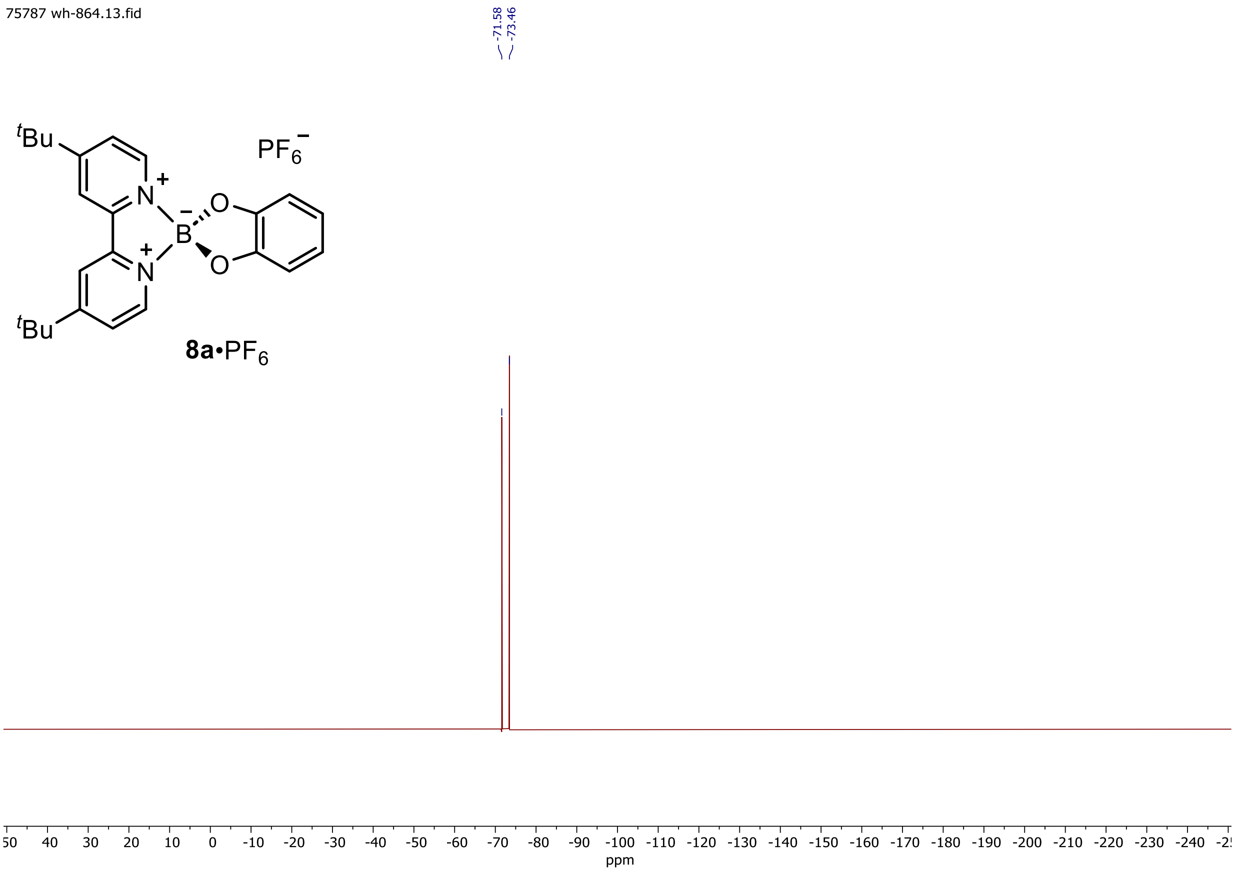

^{11}B NMR (128 MHz, acetone-d_6) of **8a**•PF_6

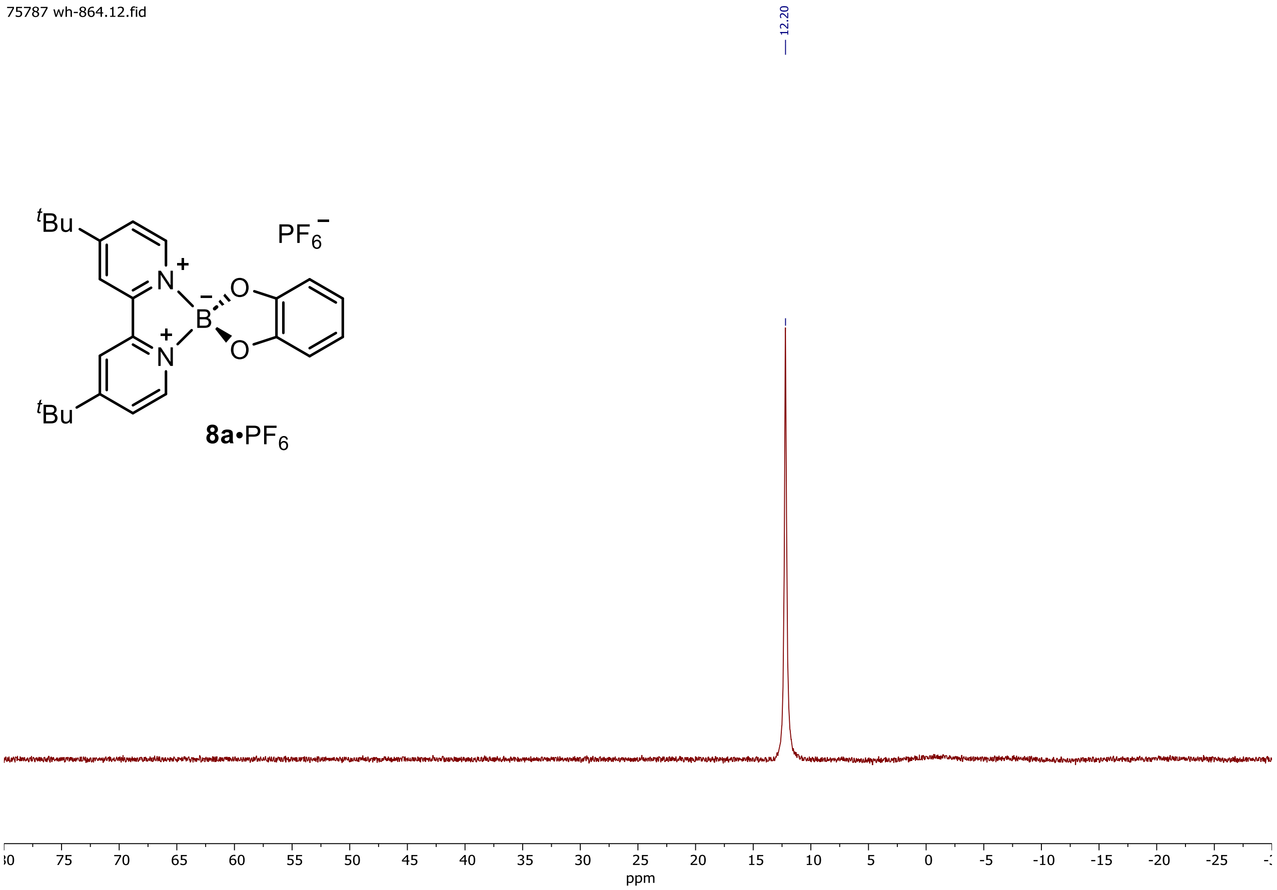

^{1}H NMR (400 MHz, acetone-d_6) of **8d**•PF_6 (see procedure)

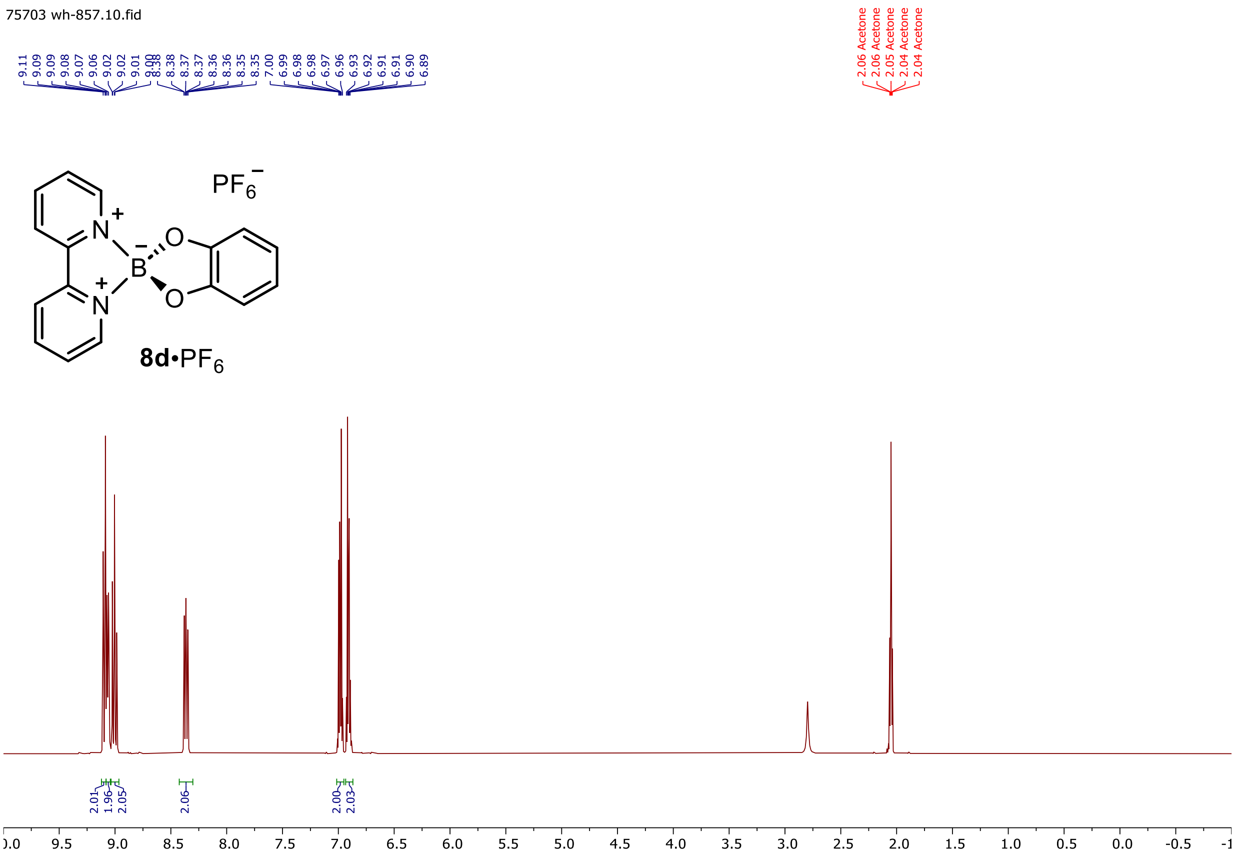

^{13}C NMR (101 MHz, acetone-d_6) of **8d**•PF_6

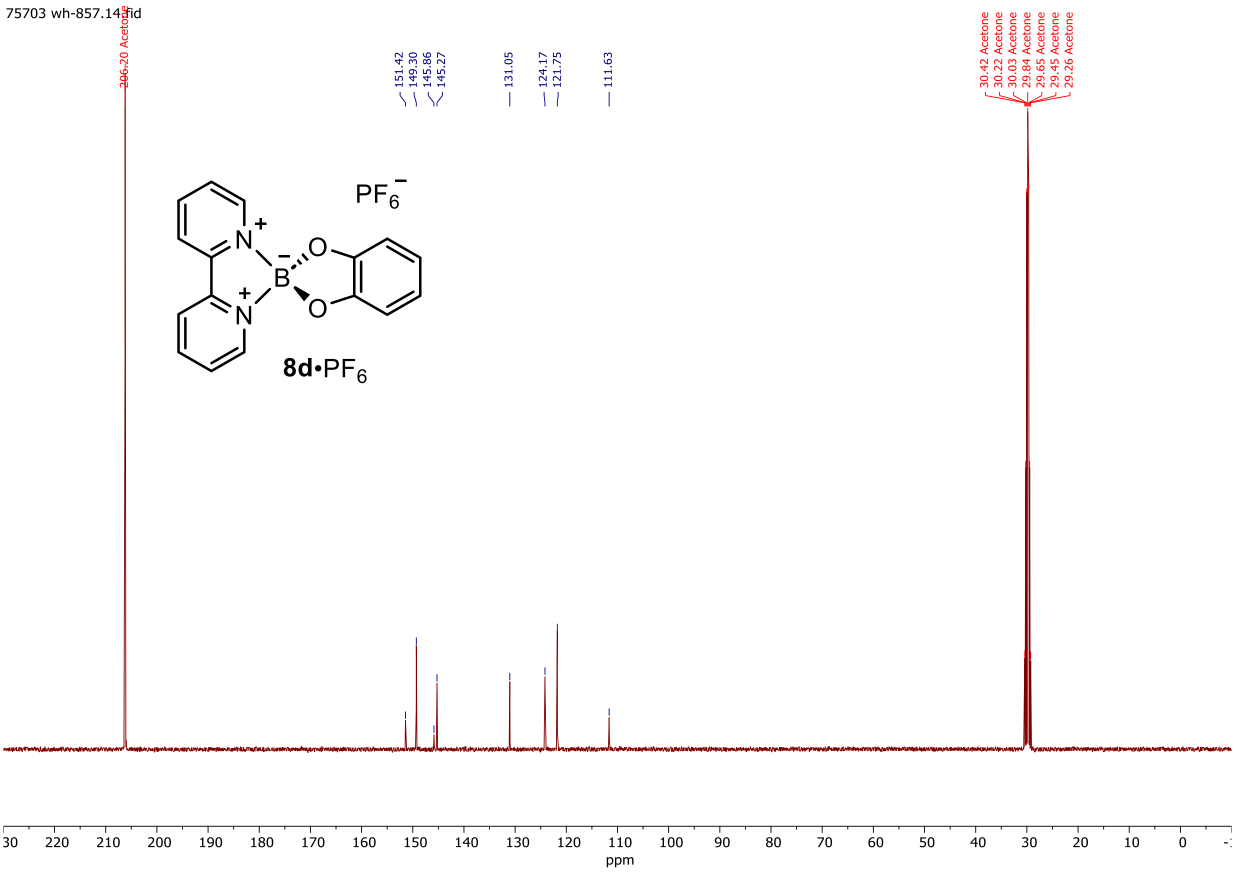

^{31}P NMR (162 MHz, acetone-d_6) of **8d**•PF_6

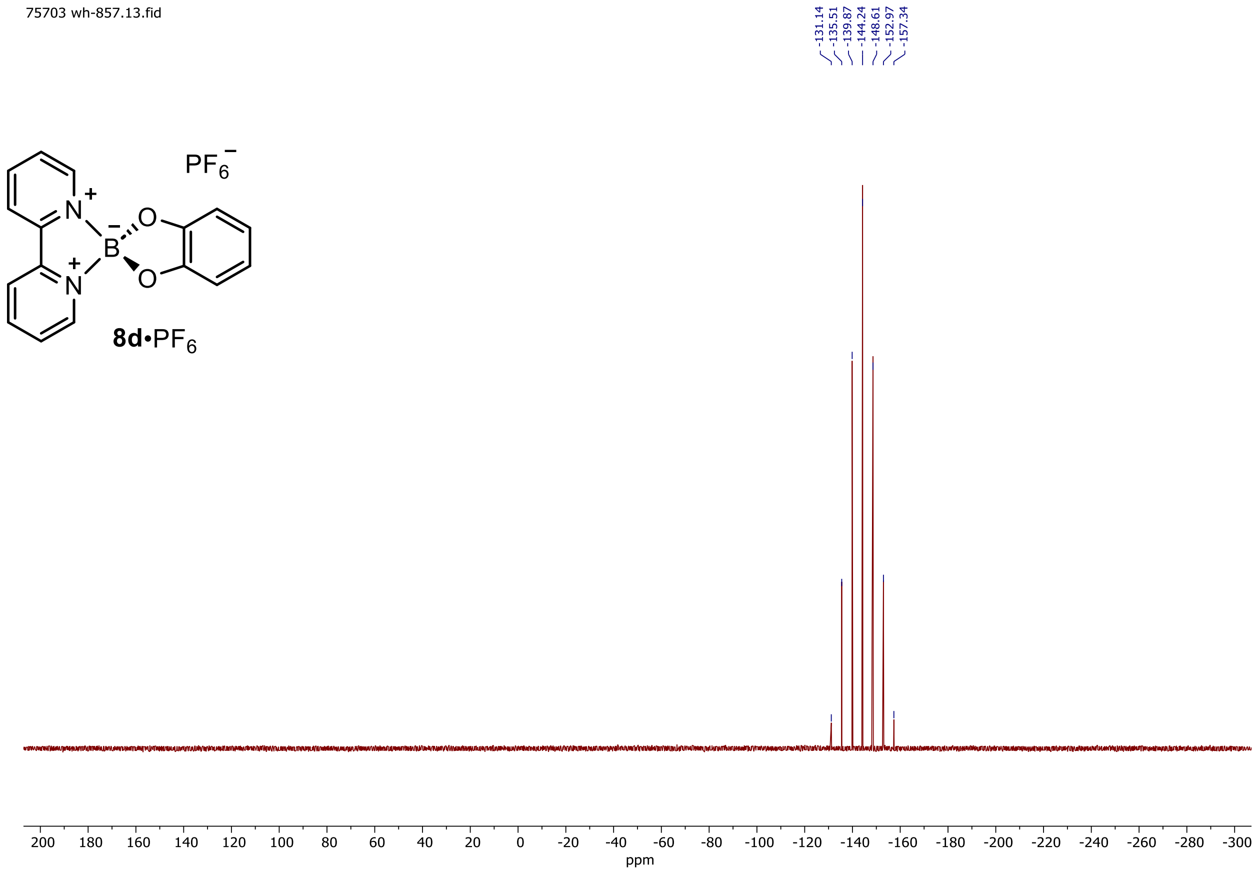

^{19}F NMR (377 MHz, acetone-d_6) of **8d**•PF_6

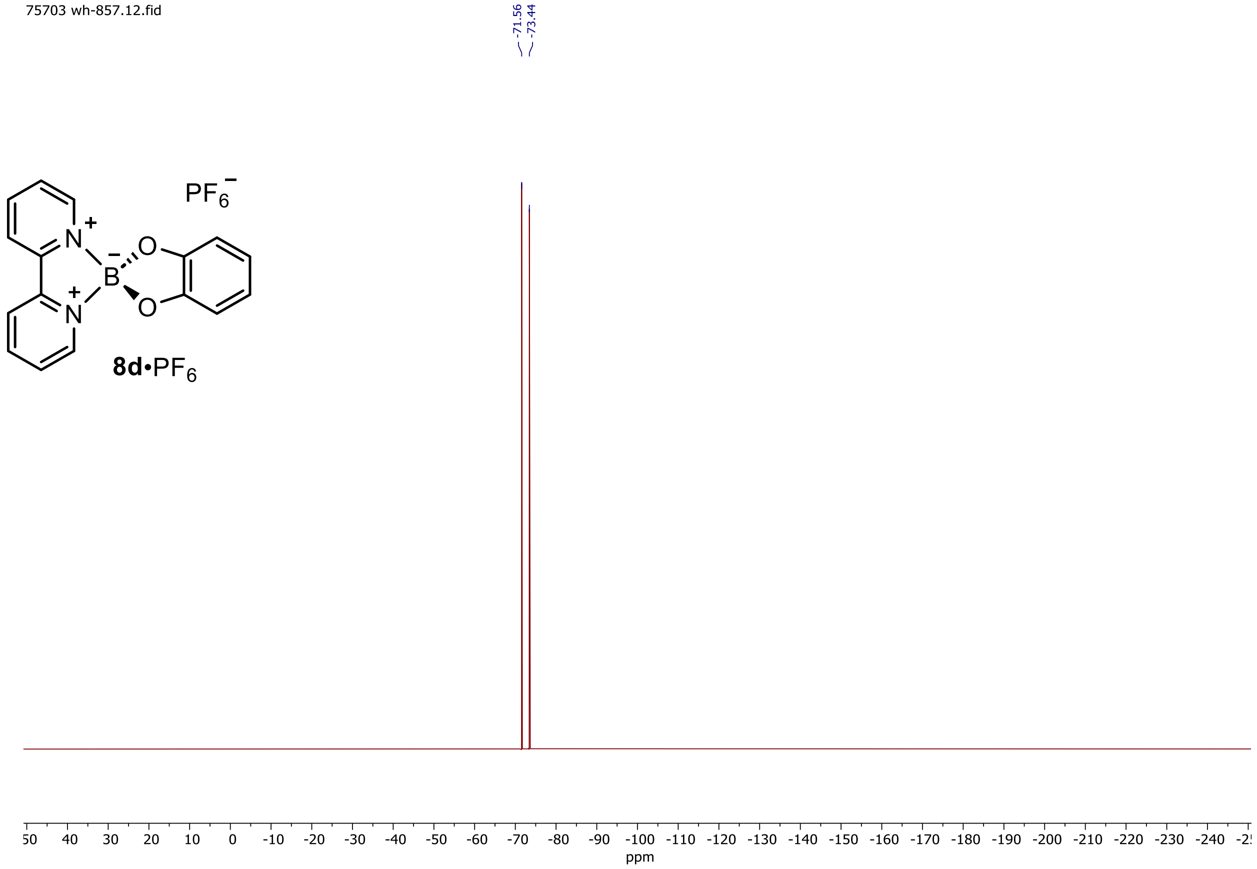

^{11}B NMR (128 MHz, acetone-d_6) of **8d**•PF_6

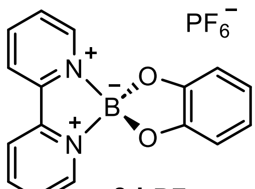

8d•PF_6

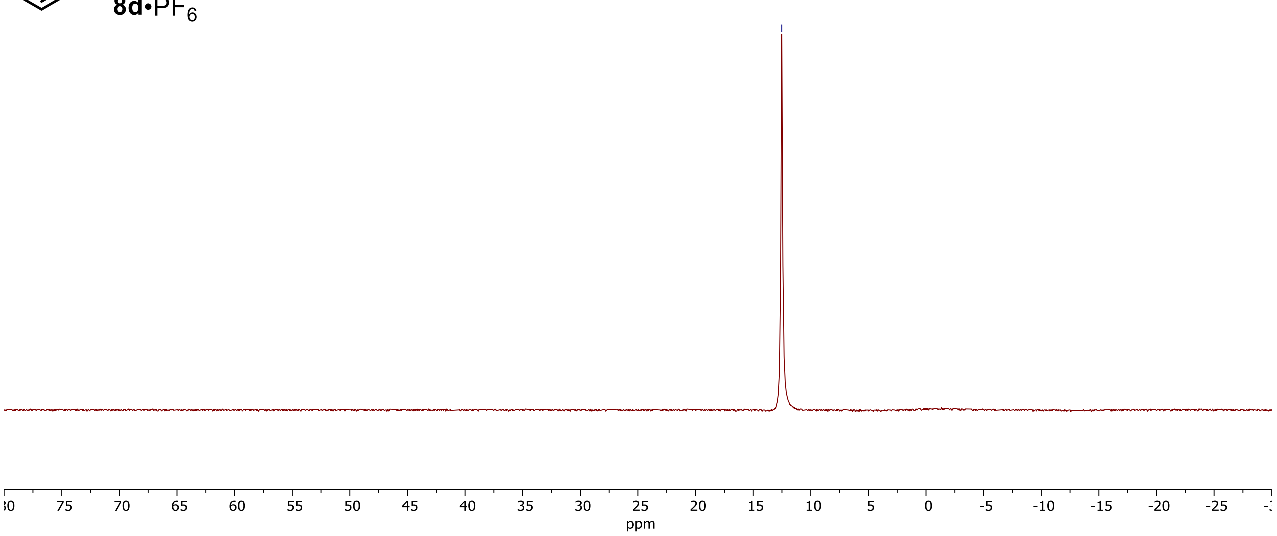

6. REFERENCES

1) Pinyou, P.; Ruff, A.; Pöller, S.; Ma, S.; Ludwig, R.; Schuhmann, W. *Chem. Eur. J.* **2016**, *22*, 5319–5326.

2) Dai, C.; Narayanam, J. M. R.; Stephenson, C. R. J. *Nat. Chem.* **2011**, *3*, 140–145.

3) Meyers, A.; Kimyonok, A.; Weck, M. *Macromolecules* **2005**, *38*, 8671–8678.

4) Atack, T. C.; Lecker, R. M.; Cook, S. P. *J. Am. Chem. Soc.* **2014**, *136*, 9521–9523.

5) Someya, H.; Yorimitsu, H.; Oshima, K. *Tetrahedron* **2010**, *66*, 5993–5999.

6) Schinzer, D.; Müller, N.; Fischer, A. K.; Prieß, J. W. *Synlett* **2000**, *2000*, 1265–1268.

7) Wang, G.-Z.; Shang, R.; Cheng, W.-M.; Fu, Y. *J. Am. Chem. Soc.* **2017**, *139*, 18307–18312.

8) Valiullina, Z. R.; Khasanova, L. S.; Selezneva, N. K.; Gimalova, F. A.; Pivnitsky, K. K.; Miftakhov, M. S. *Mendeleev Commun.* **2014**, *24*, 272–273.

9) Wu, J.-J.; Shi, Y.; Tian, W.-S. *Chem. Commun.* **2016**, *52*, 1942–1944.

10) Gui, J.; Wang, D.; Tian, W. *Angew. Chem. Int. Ed.* **2011**, *50*, 7093–7096.

11) (a) Wu, J.-J.; Shi, Y.; Tian, W.-S. *Tetrahedron Lett.* **2015**, *56*, 1215–1217. (b) Zhu, L.-C.; Yang, D.-L.; Shi, Y. *Org. Lett.* **2022**, *24*, 5825−5828.

12) Yang, C.-T.; Zhang, Z.-Q.; Tajuddin, H.; Wu, C.-C.; Liang, J.; Liu, J.-H.; Fu, Y.; Czyzewska, M.; Steel, P. G.; Marder, T. B.; Liu, L. *Angew. Chem. Int. Ed.* **2012**, *51*, 528–532.

13) Yi, J.; Liu, J.-H.; Liang, J.; Dai, J.-J.; Yang, C.-T.; Fu, Y.; Liu, L. *Adv. Synth. Catal.* **2012**, *354*, 1685–1691.

14) Fawcett, A.; Pradeilles, J.; Wang, Y.; Mutsuga, T.; Myers, E. L.; Aggarwal, V. K. *Science* **2017**, *357*, 283–286.

15) Larouche-Gauthier, R.; Elford, T. G.; Aggarwal, V. K. *J. Am. Chem. Soc.* **2011**, *133*, 16794–16797.

16) Auerhammer, N.; Schulz, A.; Schmiedel, A.; Holzapfel, M.; Hoche, J.; Röhr, M. I. S.; Mitric, R.; Lambert, C. *Phys. Chem. Chem. Phys.* **2019**, *21*, 9013–9025.

17) Bismuto, A.; Cowley, M. J.; Thomas, S. P. *ACS Catal.* **2018**, *8*, 2001–2005.

18) Elford, T. G.; Nave, S.; Sonawane, R. P.; Aggarwal, V. K. *J. Am. Chem. Soc.* **2011**, *133*, 16798–16801.

19) Zhang, L.; Wu, Z.-Q.; Jiao, L. *Angew. Chem. Int. Ed.* **2020**, *59*, 2095–2099.

20) Bull, J. A.; Charette, A. B. *J. Am. Chem. Soc.* **2010**, *132*, 1895–1902.

21) Wu, J.; Bär, R. M.; Guo, L.; Noble, A.; Aggarwal, V. K. *Angew. Chem. Int. Ed.* **2019**, *58*, 18830–18834.

22) Bose, S. K.; Brand, S.; Omoregie, H. O.; Haehnel, M.; Maier, J.; Bringmann, G.; Marder, T. B. *ACS Catal.* **2016**, *6*, 8332–8335.

23) Zhang, J.-J.; Duan, X.-H.; Wu, Y.; Yang, J.-C.; Guo, L.-N. *Chem. Sci.* **2019**, *10*, 161–166.

24) Cheng, Y.; Mück-Lichtenfeld, C.; Studer, A. *Angew. Chem. Int. Ed.* **2018**, *57*, 16832–16836.

25) Bose, S. K.; Fucke, K.; Liu, L.; Steel, P. G.; Marder, T. B. *Angew. Chem. Int. Ed.* **2014**, *53*, 1799–1803.

26) Dudnik, A. S.; Fu, G. C. *J. Am. Chem. Soc.* **2012**, *134*, 10693–10697.

27) Fasano, V.; Winter, N.; Noble, A.; Aggarwal, V. K. *Angew. Chem. Int. Ed.* **2020**, *59*, 8502–8506.

28) Zheng, Y.; Zhao, Y.; Tao, S.; Li, X.; Cheng, X.; Jiang, G.; Wan, X. *Eur. J. Org. Chem.* **2021**, *2021*, 2713–2718.

29) Dai, C.; Johnson, S. M.; Lawlor, F. J.; Lightfoot, P.; Marder, T. D.; Norman, N. C.; Orpen, A. G.; Pickett, N.

L.; Quayle, M. J.; Rice, C. R. *Polyhedron* **1998**, *17*, 4139–4143.

30) Hünig, S.; Wehner, I. *Heterocycles* **1989,** *28*, 359–363.

31) Frisch, M. J.; Trucks, G. W.; Schlegel, H. B.; Scuseria, G. E.; Robb, M. A.; Cheeseman, J. R.; Scalmani, G.; Barone, V.; Mennucci, B.; Petersson, G. A.; Nakatsuji, H.; Caricato, M.; Li, X.; Hratchian, H. P.; Izmaylov, A. F.; Bloino, J.; Zheng, G.; Sonnenberg, J. L.; Hada, M.; Ehara, M.; Toyota, K.; Fukuda, R.; Hasegawa, J.; Ishida, M.; Nakajima, T.; Honda, Y.; Kitao, O.; Nakai, H.; Vreven, T.; Montgomery, J., J. A.; Peralta, J. E.; Ogliaro, F.; Bearpark, M.; Heyd, J. J.; Brothers, E.; Kudin, K. N.; Staroverov, V. N.; Kobayashi, R.; Normand, J.; Raghavachari, K.; Rendell, A.; Burant, J. C.; Iyengar, S. S.; Tomasi, J.; Cossi, M.; Rega, N.; Millam, J. M.; Klene, M.; Knox, J. E.; Cross, J. B.; Bakken, V.; Adamo, C.; Jaramillo, J.; Gomperts, R.; Stratmann, R. E.; Yazyev, O.; Austin, A. J.; Cammi, R.; Pomelli, C.; Ochterski, J. W.; Martin, R. L.; Morokuma, K.; Zakrzewski, V. G.; Voth, G. A.; Salvador, P.; Dannenberg, J. J.; Dapprich, S.; Daniels, A. D.; Farkas, Ö.; Foresman, J. B.; Ortiz, J. V.; Cioslowski, J.; Fox, D. J., Gaussian 09, Revision C1, Gaussian, Inc., Wallingford CT, **2009**.

32) Barone, V., Structure, Magnetic Properties and Reactivities of Open-Shell Species from Density Functional and Self-Consistent Hybrid Methods. In Recent Advances in Density Functional Methods, Part I, Chong, D. P., Ed. World Scientific Publishing Co. Pte. Ltd.: Singapore, **1995**; Vol. 1, Recent Advances in Computational Chemistry, pp 287–334.

33) Mansell, S. M.; Adams, C. J.; Bramham, G.; Haddow, M. F.; Kaim, W.; Norman, N. C.; McGrady, J. E.; Russell, C. A.; Udeena, S. J. *Chem. Commun.* **2010**, *46*, 5070–5072

34) Stoll, S.; Schweiger, A. *J. Magn. Reson.* **2006**, *178*, 42–55.

35) Tucker, J. W.; Stephenson, C. R. J. *J. Org. Chem.* **2012**, *77*, 1617–1622.

36) Rehm, D.; Weller, A. *Isr. J. Chem.* **1970**, *8*, 259–271.

37) Romero, N. A.; Nicewicz, D. A. *Chem. Rev.* **2016**, *116*, 10075–10166.

38) Montalti, M., Credi, A., Prodi, L., Gandolfi, M. T. Handbook of Photochemistry (CRC/Taylor & Francis, Boca Raton, FL, ed. 3, 2006).

39) Tlahuext-Aca, A., Garza-Sanchez, R. A., Schäfer, M., Glorius, F. *Org. Lett.* **2018**, *20*, 1546–1549.

40) Greene, H. J. M.; Ghosh, D.; Sazanovich, I. V.; Phelps, R.; Curchod, B. F. E.; Orr-Ewing, A. J. *J. Phys. Chem. Lett.* **2024**, *15*, 9153−9159.

41) Grubb, M. P.; Orr-Ewing, A. J.; Ashfold, M. N. R. *Rev. Sci. Instrumen.* **2014**, *84*, 064104

42) Bruker, SAINT+ v8.39.0 Integration Engine, Data Reduction Software, Bruker Analytical X-ray Instruments Inc., Madison, WI, USA, **2018**.

43) Bruker, SADABS 2018, Bruker AXS Area Detector Scaling and Absorption Correction, Bruker Analytical X-ray Instruments Inc., Madison, Wisconsin, USA, **2018**.

44) Sheldrick, G. *Acta Cryst., Sect. A* **2015**, *71*, 3–8.

45) Sheldrick, G. *Acta Cryst., Sect. A* **2008**, *64*, 112–122.

46) Sheldrick, G. *Acta Cryst., Sect. C* **2015**, *71*, 3–8

47) Dolomanov, O. V.; Bourhis, L. J.; Gildea, R. J.; Howard, J. A. K.; Puschmann, H. *J. Appl. Crystallogr.* **2009**, *42*, 339–341.
